# Optimising treatment of cardiovascular risk factors in cerebral small vessel disease using genetics

**DOI:** 10.1093/brain/awae399

**Published:** 2025-06-03

**Authors:** Fatemeh Koohi, Eric L. Harshfield, Dipender Gill, Wenjing Ge, Stephen Burgess, Hugh S. Markus

**Affiliations:** 1 Stroke Research Group, Department of Clinical Neurosciences, University of Cambridge, Cambridge, UK; 2 Department of Epidemiology and Biostatistics, School of Public Health, Imperial College London, London, UK; 3 MRC Biostatistics Unit, University of Cambridge, Cambridge, UK

**Keywords:** cerebral small vessel disease, lacunar stroke, small vessel stroke, Mendelian randomization

## Abstract

Cerebral small vessel disease (cSVD) causes lacunar stroke (LS), intracerebral haemorrhage, and is the most common pathology underlying vascular dementia. However, there are few trials examining whether treating conventional cardiovascular risk factors reduce stroke risk in cSVD, as opposed to stroke as a whole. We used Mendelian randomization techniques to investigate which risk factors are causally related to cSVD and to evaluate whether specific drugs may be beneficial in cSVD prevention.

We identified genetic proxies for blood pressure traits, lipids, glycaemic markers, anthropometry measures, smoking, alcohol consumption, and physical activity from large-scale genome-wide association studies of European ancestry. We also selected genetic variants as proxies for drug target perturbation in hypertension, dyslipidaemia, hyperglycaemia, and obesity. Mendelian randomization was performed to assess their associations with LS from the GIGASTROKE Consortium (*n* = 6811) and in a sensitivity analysis in a cohort of patients with MRI-confirmed LS (*n* = 3306). We also investigated associations with three neuroimaging features of cSVD, namely, white matter hyperintensities (*n* = 552 91), fractional anisotropy (*n* = 36 460), and mean diffusivity (*n* = 36 012).

Genetic predisposition to higher systolic and diastolic blood pressure was associated with LS and cSVD imaging markers. Genetically predicted liability to diabetes, obesity, smoking, higher triglyceride levels, and the ratio of triglycerides to high density lipoprotein (HDL) also showed detrimental associations with LS risk, while genetic predisposition to higher HDL concentrations and moderate-to-vigorous physical activity showed protective associations. Genetically proxied blood pressure-lowering through calcium channel blockers (CCBs) was associated with cSVD imaging markers, while genetically proxied HDL-raising through Cholesteryl Ester Transfer Protein (CETP) inhibitors, triglyceride-lowering through lipoprotein lipase (LPL), and weight-lowering through gastric inhibitory polypeptide receptor (GIPR) were associated with lower risk of LS.

Our findings highlight the importance of some conventional cardiovascular risk factors, including blood pressure and BMI, in cSVD, but not other e.g. LDL. The findings further demonstrate the potential beneficial effects of CCBs on cSVD imaging markers and CETP inhibitors, LPL enhancement, and GIPR obesity-targeted drugs on LS. They provide useful information for initiating future clinical trials examining secondary prevention strategies in cSVD.

## Introduction

Cerebral small vessel disease (cSVD) causes lacunar stroke (LS), which comprises a quarter of all ischaemic strokes, as well as intracerebral haemorrhage, and is the most common pathology underlying vascular dementia. Characteristic MRI features include lacunar infarcts and white matter hyperintensities (WMH).^1^ Despite its global importance,^2^ there are few proven treatments for cSVD partly due to the lack of understanding of the underlying disease mechanisms.^3^

Even for conventional risk factors, there is incomplete data about which are causally related to cSVD, for which outcomes could be improved through risk reduction or modification. Many studies have looked at the relationship of common cardiovascular risk factors to LS risk, both in comparison to stroke-free controls and to other stroke subtypes. However, interpretation is complicated because stroke subtyping has often been performed based on diagnosis of a clinical lacunar syndrome combined with CT brain imaging. CT imaging may not show lacunar infarcts, particularly in the first 24 hours. When more rigorous subtyping is performed using MRI, which enables more accurate diagnosis of lacunar infarction, it has been shown that as many as half of all patients classified as LS using CT imaging do not have cSVD.^4^ There are few large risk factor studies for LS where MRI has been performed. An alternative approach is to study risk factor associations with MRI markers of cSVD in large cohorts, often population based. This allows accurate phenotyping of the features of cSVD but provides information on the more chronic features of cSVD, rather than risk factors for acute infarction.

There is also limited clinical trial data as to whether secondary prevention of cardiovascular risk factors improves outcome in LS, as secondary prevention strategies have been mostly inferred from studies of ischemic stroke in general, the majority of which did not specifically examine efficacy in LS.^3^ The limitations of stroke subtyping in such studies make it difficult to have confidence whether treatments are specifically effective in LS. High quality clinical trial data in well-subtyped LS is only available for hypertension treatment.^5^ This has led to recent European Stroke Organisation guidelines on LS concluding that there is “little direct evidence, mostly of low quality” to guide secondary prevention of cSVD.^6^

A useful approach to investigate whether risk factors are causally related to cSVD is Mendelian randomization (MR). This is an analytical method that uses genetic variants as instrumental variables for risk factors.^7^ It can overcome a major limitation of evidence from observational studies, namely unmeasured confounding. Exposures can be any factor robustly associated with genetic variation, for which the genetic variation mimics an intervention in the factor.^7^ A particular advantage of MR in cSVD is that genetic data obtained from cohorts with well-subtyped LS can be used to overcome the difficulties in LS subtyping described above. A related technique is drug target MR. Proteins represent the majority of drug targets; therefore, genetic variants affecting the function or expression of genes encoding these proteins can be used as proxies for investigating the effect of pharmacologically perturbing the corresponding protein drug target.^8,9^ This technique is increasingly being used to prioritise potential drugs interventions prior to definitive clinical trials.

We applied these techniques to cSVD to provide insights into optimal approaches to treat conventional cardiovascular risk factors in the secondary prevention of cSVD. First, we assessed evidence for whether traditional cardiovascular risk factors are causally related to both LS and neuroimaging markers of cSVD. Second, using drug target MR we examined whether existing drug targets to treat these conditions (antihypertensives, lipid-lowering drugs, antidiabetic drugs, and anti-obesity drug targets) are likely to be effective for treatment of LS and cSVD. We also included a second cohort of MRI-subtyped LS patients as a sensitivity analysis.

## Materials and methods

### Study Design

**Figure 1:**
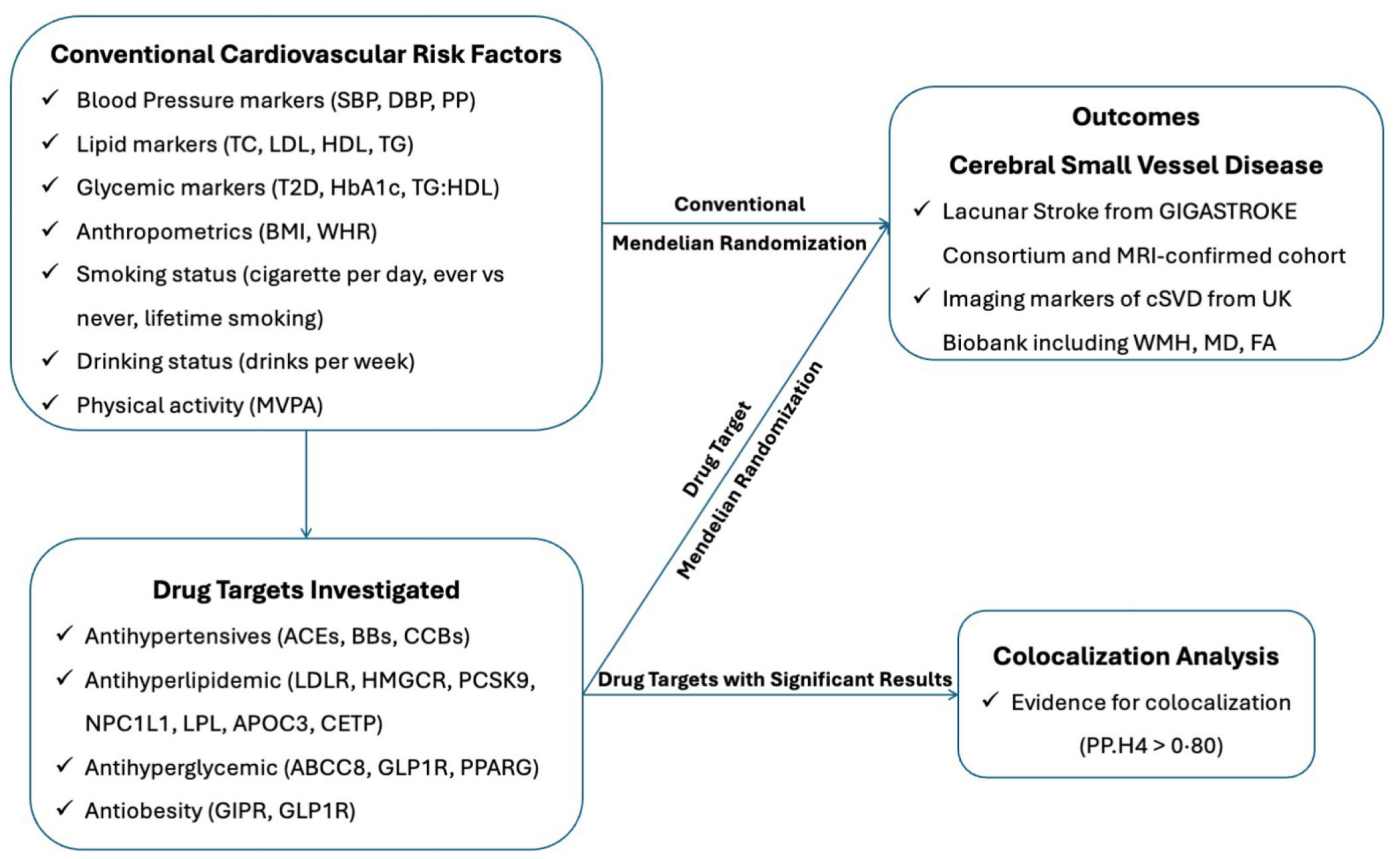
Outline of the study design. Abbreviations: SBP, systolic blood pressure; DBP, diastolic blood pressure; PP, pulse pressure; TC, total cholesterol; LDL, low density lipoprotein; HDL, high density lipoprotein; TG, triglycerides; T2D, type 2 diabetes; HbA1c, haemoglobin A1C; TG:HDL, triglycerides to high density lipoprotein ratio; BMI, body mass index; WHR, waist to hip ratio; MVPA, moderate-to-vigorous intensity physical activity during leisure time; ACEs, angiotensin-converting enzyme inhibitors; BBs, beta blockers; CCBs, calcium channel blockers; *LDLR*, LDL Receptor; HMGCR, HMG-CoA reductase inhibitors; PCSK9, proprotein convertase subtilisin/kexin type 9 inhibitors; NPC1L1, NPC1 Like Intracellular Cholesterol Transporter 1 inhibitors; *LPL*, lipoprotein lipase; APOC3, Apolipoprotein C-III inhibitors; CETP, Cholesteryl Ester Transfer Protein inhibitors; ABCC8, ATP binding cassette subfamily C member 8; GLP1R, glucagon like peptide 1 receptor; PPARG, peroxisome proliferator activated receptor gamma; GIPR, gastric inhibitory polypeptide receptor.

The current study conducted using a two-sample MR design, which utilizes exposure and outcome summary association data estimated in two independent studies, allowing increased statistical power to infer causal relationships. An outline of the study design is shown in Fig. 1. We followed the Strengthening the Reporting of Observational Studies in Epidemiology Using Mendelian Randomization (STROBE-MR) reporting guidelines.^10^

### Data Sources and ethics approval

Data sources were based on publicly available summary-level data from GWAS studies, and ethics approval for each study can be found in the original publications.

### Genetic associations with cardiovascular risk factors

Summary-level data for 17 cardiovascular risk factors including blood pressure markers [diastolic blood pressure (DBP), systolic blood pressure (SBP), pulse pressure (PP)], lipids [low-density lipoprotein (LDL), high-density lipoprotein (HDL), triglycerides (TG), total cholesterol (TC)], glycaemic markers [type 2 diabetes (T2D), haemoglobin A_1C_ (HbA_1c_), TG to HDL ratio as an insulin resistance marker), anthropometrics [body mass index (BMI), waist to hip ratio (WHR)], smoking status (ever vs never, cigarettes smoked per day, life time smoking index), drinking status (drinks per week), and physical activity [moderate-to-vigorous intensity physical activity during leisure time (MVPA)] were obtained from recent large European GWAS. Detailed information on these datasets is summarised in Table 1.

### Genetic associations with lacunar stroke

Summary genetic associations with LS were extracted from the GIGASTROKE Consortium,^11^ restricted to individuals of European ancestry, including 6811 cases and 1 234 808 controls. Given that LS subtyping in GIGASTROKE was primarily conducted using CT imaging, which has only a modest level of accuracy and reduced power to identify genetic associations,^12^ we also utilized GWAS data from a second dataset consisting of patients with MRI-confirmed LS. This dataset comprised 3306 cases and 19 955 controls from the UK DNA Lacunar studies 1 and 2,^12^ as well as other studies within the International Stroke Genomics Consortium.^12^ This dataset was analysed in our previous MR study.^13^

### Genetic associations with imaging markers of cSVD

For WMH, we updated a GWAS performed in our previous MR study^13^ on UK Biobank, increasing the sample size to 37 355, and meta-analysed this with data from the CHARGE Consortium (*N* = 17 936) to obtain a total sample size of *n* = 55 291. For diffusion tensor imaging (DTI) metrics of white matter tracts, we updated our previous GWAS to include all available participants with DTI measures in UK Biobank; the final cohort consisted of *n* = 36 012 for mean diffusivity (MD) and *n* = 36 460 for fractional anisotropy (FA).^14^

### Genetic instrument selection

Using summary-level data obtained for each cardiovascular risk factor, independent genetic variants associated with each risk factor at genome-wide significance (*P* < 5 × 10^−8^) were selected as genetic instruments for estimating the causal effect of each risk factor. These variants were clumped to a linkage disequilibrium (LD) threshold of *R*^*2*^ < 0.001 with a window size of 1000 kb in PLINK2^15^ using the 1000 Genomes Phase 3 reference panel, build 37.^16^

To generate genetic instruments as proxies for drug target perturbation in hypertension, dyslipidaemia, hyperglycaemia, and obesity, common drug classes for managing these risk factors and their target genes were obtained using approaches similar to previous published studies.^17–22^

For antihypertensive drugs, genes encoding drug targets for angiotensin-converting enzyme (ACE) inhibitors, angiotensin receptor blockers (ARBs), β-blockers (BBs), calcium channel blockers (CCBs), and thiazide diuretic agents were obtained from a study by Gill et al.^17^ Given that SBP is relatively more important in the management of hypertension compared to DBP,^23^ genetic variants within ±100 kb windows from identified gene regions associated with SBP at genome-wide significance (*P* < 5×10^−8^) were obtained from a GWAS meta-analysis of 757 601 individuals of European ancestry in UK Biobank and the International Consortium of Blood Pressure-Genome Wide Association Studies (ICBP).^24^ Since no genetic variants within the target genes for ARBs and thiazide diuretic agents were found to be associated with SBP, they were not considered further.

For lipid-lowering drugs, genetic variants associated with LDL, TG, or HDL concentrations at genome-wide significance (*P* < 5×10^−8^) and located within ±100 kb windows of gene regions corresponding to seven drug targets including *HMGCR* (a proxy for statin treatment), *PCSK9* (a proxy for PCSK9 inhibitors), *LDLR* (a proxy for inhibition of the LDL receptor), *NPC1L1* (a proxy for ezetimibe), *APOC3* (a proxy for APOC3 inhibitors), *LPL* (proxy for lipoprotein lipase inhibition), and *CETP* locus (a proxy for CETP inhibitors) were obtained from the recent GWAS meta-analysis from the Global Lipids Genetics Consortium involving 1 320 000 individuals of European ancestry.^25^

For glucose-lowering drugs, variants associated with type 2 diabetes and located within ±100 kb windows from gene regions corresponding to five approved type 2 diabetes drugs with known mechanisms of action, such as sulfonylurea receptor 1 [ATP binding cassette subfamily C member 8 (*ABCC8*)], *PPARG, SGLT2, DPP4*, and *GLP1R* were obtained from a recent GWAS meta-analysis of type 2 diabetes in the Million Veterans Program, comprising 148 726 cases and 965 732 controls of European ancestry.^26^ There were no genome-wide significant variants within ±100 kb windows from *SLC5A2* and *DPP4*, so these were excluded from further analyses. Because the mechanism(s) of action of metformin remain largely unknown,^27^ reliable genetic proxies could not be obtained.

For anti-obesity targets, variants associated with BMI at genome-wide significance (*P* < 5×10^-8^) within ±100 kb windows from *GLP1R* and *GIPR* gene regions were extracted from a recent GWAS meta-analysis of the UK Biobank, the GIANT Consortium, and the Million Veteran Program (MVP) involving 1 122 049 individuals of European ancestry.^28^

Independent instruments as proxies for drug targets were also constructed in PLINK2^15^ by obtaining variants with a weak LD threshold of *R*^*2*^ < 0.10 within a window size of 500 kb using the 1000 Genomes Phase 3 reference panel, build 37.^16^

Details for drug classes, target genes, and genomic regions of each encoding gene screened for selecting genetic proxies for each drug target are provided in Table 2.

### Statistical Analysis

Causal associations were estimated for our primary analysis method using the Wald ratio test for a single genetic instrument and the random-effects inverse variance-weighted (IVW) method for multiple genetic instruments.

The validity of MR results relies on three main assumptions: the instrumental variables (1) must be related to the exposure (relevance assumption), (2) must not be associated with any confounders that influence exposure or outcome (independence assumption), and (3) must affect the outcome solely through the exposure, not through any other pathway (exclusion restriction assumption).^7^ Sensitivity analyses were conducted to assess the robustness of MR results against potential violations of these assumptions. To evaluate the relevance assumption, the strength of each genetic variant was measured using F-statistics, with an F-statistic value of 10 or higher indicating the absence of weak instrument bias. Additionally, the variance explained by each genetic instrument (R^2^) was estimated. Statistical power was also calculated using the online web tool (https://sb452.shinyapps.io/power/) to ensure sufficient statistical power. To statistically test for and correct potential horizontal pleiotropy, which violates the independence assumption, the MR-Egger regression was employed. The Egger intercept provides a statistical test for the presence of horizontal pleiotropy, and the slope gives an unbiased causal estimate even in the presence of pleiotropy.^29^ For uncorrelated horizontal pleiotropy, we applied the weighted median estimator as an additional sensitivity analysis, which provides consistent estimates if at least 50% of the genetic variants (or 50% of the weight in a weighted analysis) are valid IVs.^30^ Furthermore, we assessed heterogeneity among genetic instruments through the Cochran Q test. To assess the exclusion restriction assumption, colocalization analysis was conducted for drug targets that demonstrated significant associations with any outcomes, utilizing the 'coloc' package.^31^ This method evaluates the probability (PP.H4) that variants associated with both the drug target and related outcome share the same causal variant at a given locus, and the probability (PP.H3) that drug targets and related outcome are influenced by distinct causal variants that are in LD with each other.^31^ A posterior probability greater than 0.80 supported the tested configuration.^31^ Prior probabilities were set according to the default options. Drug targets that strongly colocalised with an outcome (PP.H4 > 0.80) were considered potential target genes. To address the issue of multiple testing, a false discovery rate-adjusted *P*-value < 0.05 was used.

All statistical analyses were performed in R 4.4.0 (R Core Team, 2021). MR analyses were conducted using the 'TwoSampleMR' package.

## Results

Characteristics of genetic variants used to instrument cardiovascular risk factors and drug targets are presented in Supplementary Tables 1-5. All estimated *F*-statistics were greater than 10, indicating low risk of weak instrument bias. The statistical power of the MR analyses is presented in Supplementary Table 6.

### Genetically predicted cardiovascular risk factors and cSVD

**Figure 2:**
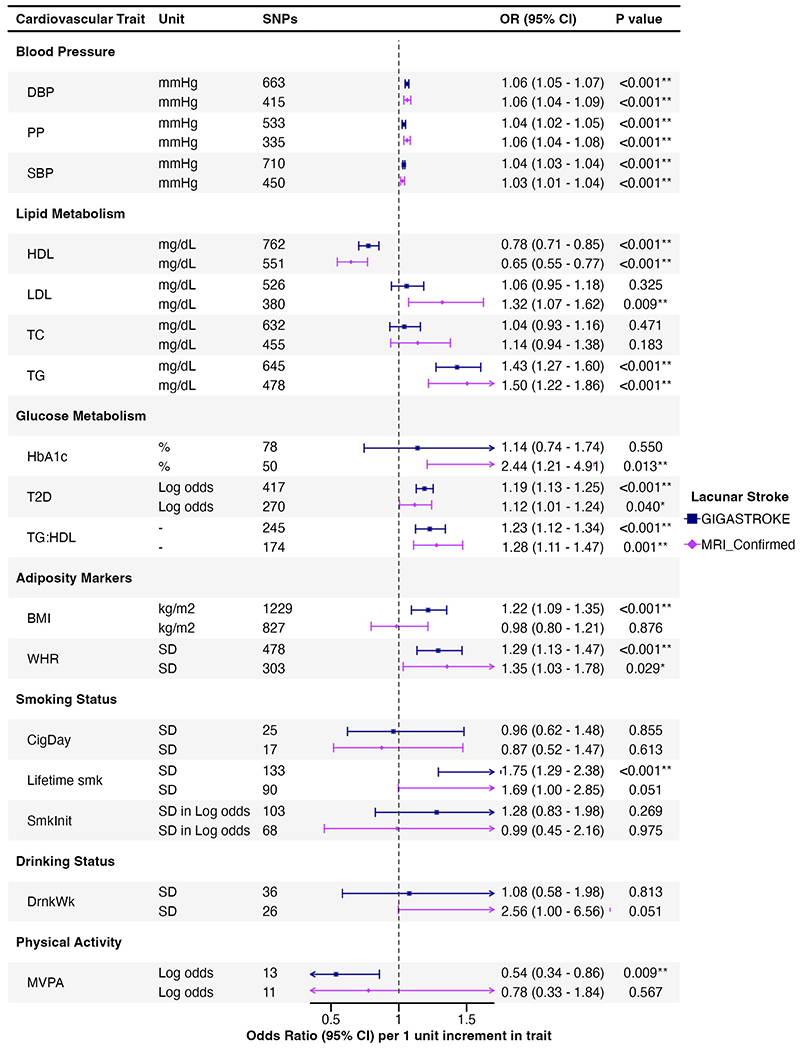
Associations of genetically predicted cardiovascular risk factors with lacunar stroke. Results are presented using GWAS data from LS in GIGASTROKE, and in a sensitivity analysis in a cohort of MRI-confirmed LS; SNPs, single nucleotide polymorphisms; DBP, diastolic blood pressure; PP, pulse pressure; SBP, systolic blood pressure; HDL, high density lipoprotein; LDL, low density lipoprotein; TC, total cholesterol; TG, triglycerides; T2D, type 2 diabetes; HbA1c, haemoglobin A1C; TG:HDL, triglycerides to high density lipoprotein ratio; BMI, body mass index; WHR, waist to hip ratio; Lifetime smk, life time smoking index; SmkInit, smoking initiation; DrnkWk, drinks per week; and MVPA, moderate-to-vigorous intensity physical activity during leisure time. *Results with a significant *P* value < 0.05. **Results with a significant *P* value after false discovery rate multiple testing correction.

**Figure 3:**
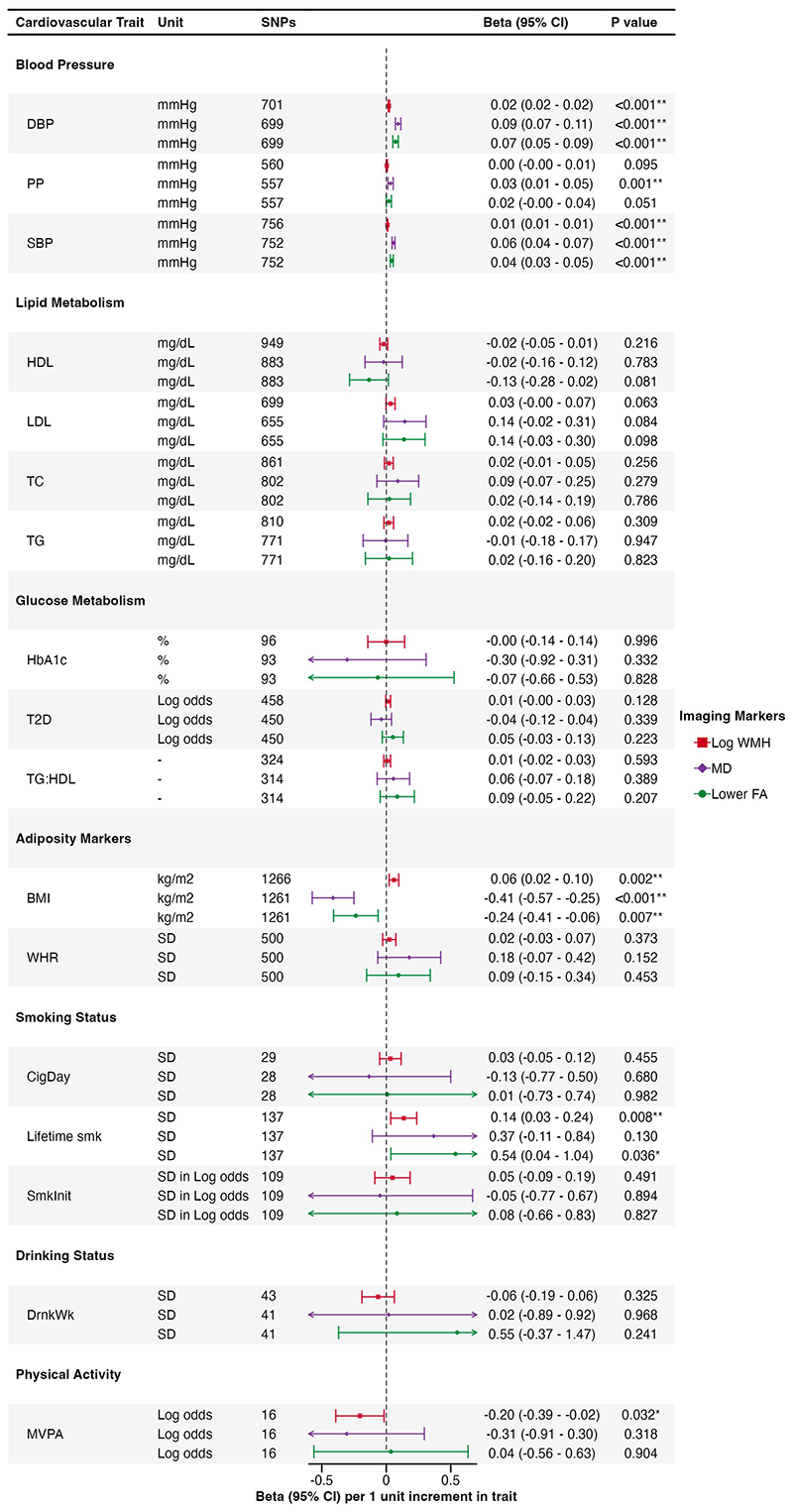
Associations of genetically predicted cardiovascular risk factors with imaging markers of cSVD. WMH indicates white matter hyperintensity; FA, fractional anisotropy; MD, mean diffusivity; SNPs, single nucleotide polymorphisms; DBP, diastolic blood pressure; PP, pulse pressure; SBP, systolic blood pressure; HDL, high density lipoprotein; LDL, low density lipoprotein; TC, total cholesterol; TG, triglycerides; T2D, type 2 diabetes; HbA1c, haemoglobin A1C; TG:HDL, triglycerides to high density lipoprotein ratio; BMI, body mass index; WHR, waist to hip ratio; Lifetime smk, life time smoking index; SmkInit, smoking initiation; DrnkWk, drinks per week; and MVPA, moderate-to-vigorous intensity physical activity during leisure time. *Results with a significant *P* value < 0.05. **Results with a significant *P* value after false discovery rate multiple testing correction.

Associations of genetically predicted cardiovascular risk factors with LS are shown in Fig. 2, and with neuroimaging features of cSVD in Fig. 3.

### Blood pressure

IVW MR analysis showed that genetically predicted SBP, DBP, and PP were associated with a higher risk of LS in both the GIGASTROKE and MRI-confirmed cohorts (Fig. 2). Genetically predicted SBP and DBP were associated with all three cSVD markers after FDR correction (Fig. 3). Pulse pressure was associated solely with MD.

### Lipids

There was no association of genetically predicted LDL levels with LS in GIGASTROKE, although an association was found for MRI-confirmed LS. In contrast, there was a consistent association between genetically predicted higher TG and LS (GIGASTROKE: OR, 1.43 [95% CI, 1.27–1.60]; MR-confirmed LS: OR, 1.50 [95% CI, 1.22–1.86] per 1 mg/dL increase in TG levels), and a protective association for genetically predicted higher levels of HDL (GIGASTROKE: OR, 0.78 [95% CI, 0.71–0.85]; MRI-confirmed LS: OR, 0.65 [95% CI, 0.55–0.77] per 1 mg/dL increase in HDL levels) across both cohorts which persisted after FDR correction. There were no significant associations between lipids with any neuroimaging marker of cSVD.

### Glycaemic markers

We found associations with several different glycaemic markers. Genetical liability to T2D was associated with LS in both GIGASTROKE (OR, 1.19 [95% CI, 1.13–1.25] per one-unit increase in log odds of T2D) and MRI-confirmed LS (OR, 1.12 [95% CI, 1.01–1.24] per one-unit increase in log odds of T2D) but the later did not survive FDR correction. Genetically predicted HbA_1c_ was associated with LS in MRI-confirmed cohort (OR, 2.44 [95% CI, 1.21–4.91] per 1 percentage increase in HbA_1c_) but not in GIGASTROKE. Genetically predicted TG:HDL ratio, a marker of insulin resistance, was associated with LS in both cohorts after FDR (GIGASTROKE: OR, 1.23 [95% CI, 1.12–1.34]; MRI-confirmed LS: OR, 1.28 [95% CI, 1.11–1.47] per 1 unit increase in TG:HDL ratio). There were no significant associations between any genetically predicted glycaemic marker with any neuroimaging marker of cSVD.

### Obesity

Genetically predicted WHR was associated with LS in both cohorts, and genetically predicted BMI was associated with LS in the GIGASTROKE (OR, 1.22 [95% CI, 1.09–1.35] per 1 kg/m^2^ increase in BMI), but not with MRI-confirmed LS. Genetically predicted BMI was associated with all three cSVD markers (WMH: β, 0.06 [95% CI, 0.02 to 0.10]; MD: β, -0.41 [95% CI, -0.57 to -0.25]; FA: β, -0.24 [95% CI, -0.41 to -0.06] per 1 kg/m^2^ increase in BMI).

### Smoking

Genetically predicted lifetime smoking index was associated with LS in GIGASTROKE (OR, 1.75 [95% CI, 1.29–2.38] per 1-SD increase in lifetime smoking index) but not in the MRI-confirmed cohort. There were no associations with other smoking markers. Genetically predicted lifetime smoking was associated with higher WMH and lower FA (WMH: β, 0.14 [95% CI, 0.03 to 0.24]; FA: β, 0.54 [95% CI, 0.04 to 1.04] per 1-SD increase in lifetime smoking index). However, the association with FA was not statistically significant after FDR correction.

### Alcohol

There were no significant associations between genetically predicted alcohol intake and either LS or any neuroimaging marker of cSVD.

### Exercise

Genetically predicted moderate-to-vigorous intensity physical activity during leisure time (MVPA) showed a significant association with a lower risk of LS in GIGASTROKE (OR, 0.54 [95% CI, 0.34–0.86] per 1 log odds increase in MVPA) but not with MRI-confirmed LS. Genetically predicted MVPA showed a nominal association with lower WMH but not with MD or FA.

### Sensitivity analyses

Associations were generally consistent across alternative MR methods, although the 95% confidence intervals were wider in the weighted median and MR-Egger regression analyses, and only blood pressure markers passed the significance threshold in the weighted median method. Full results are presented in Supplementary Table 7 for LS and Supplementary Table 8 for imaging markers of cSVD. Despite some evidence of heterogeneity among variants (*P* < 0.05; Supplementary Tables 7 and 8), the MR-Egger intercept test revealed no evidence of horizontal pleiotropy, which strengthens causal inferences (*P* for the Egger intercept > 0.05; Supplementary Tables 7 and 8). We had sufficient power (mostly 100%) to detect the relationships between risk factors and outcomes, except for smoking initiation with MRI-confirmed LS and cigarettes per day with FA, where the power might be insufficient (5.80% and 45.3%, respectively; Supplementary Table 6).

### Genetically proxied drug targets perturbation and cSVD

**Figure 4:**
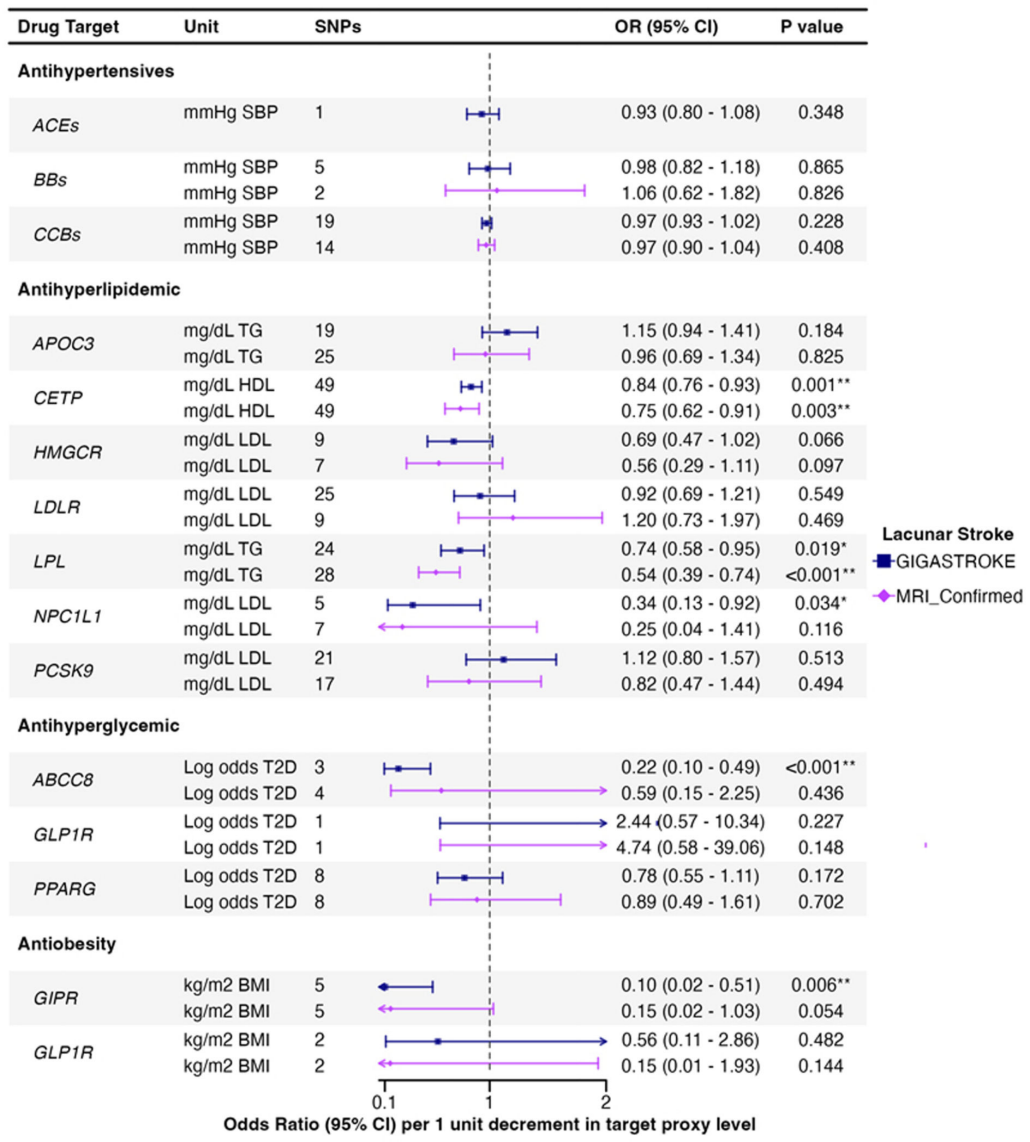
Associations of genetically proxied antihypertensive, antihyperlipidemic, antihyperglycemic, and antiobesity therapies with lacunar stroke. Results are presented using GWAS data from LS in GIGASTROKE, and in a sensitivity analysis in a cohort of MRI-confirmed LS; SNPs, single nucleotide polymorphisms; ACEs, angiotensin-converting enzyme inhibitors; BBs, beta blockers; CCBs, calcium channel blockers; APOC3, Apolipoprotein C-III inhibitors; CETP, Cholesteryl Ester Transfer Protein inhibitors; HMGCR, HMG-CoA reductase inhibitors; *LDLR*, LDL Receptor; *LPL*, lipoprotein lipase; NPC1L1, NPC1 Like Intracellular Cholesterol Transporter 1 inhibitors; PCSK9, proprotein convertase subtilisin/kexin type 9 inhibitors; ABCC8, ATP binding cassette subfamily C member 8; GLP1R, glucagon like peptide 1 receptor; PPARG, peroxisome proliferator activated receptor gamma; GIPR, gastric inhibitory polypeptide receptor. *Results with a significant *P* value < 0.05. **Results with a significant *P* value < 0.05 after false discovery rate multiple testing correction.

**Figure 5:**
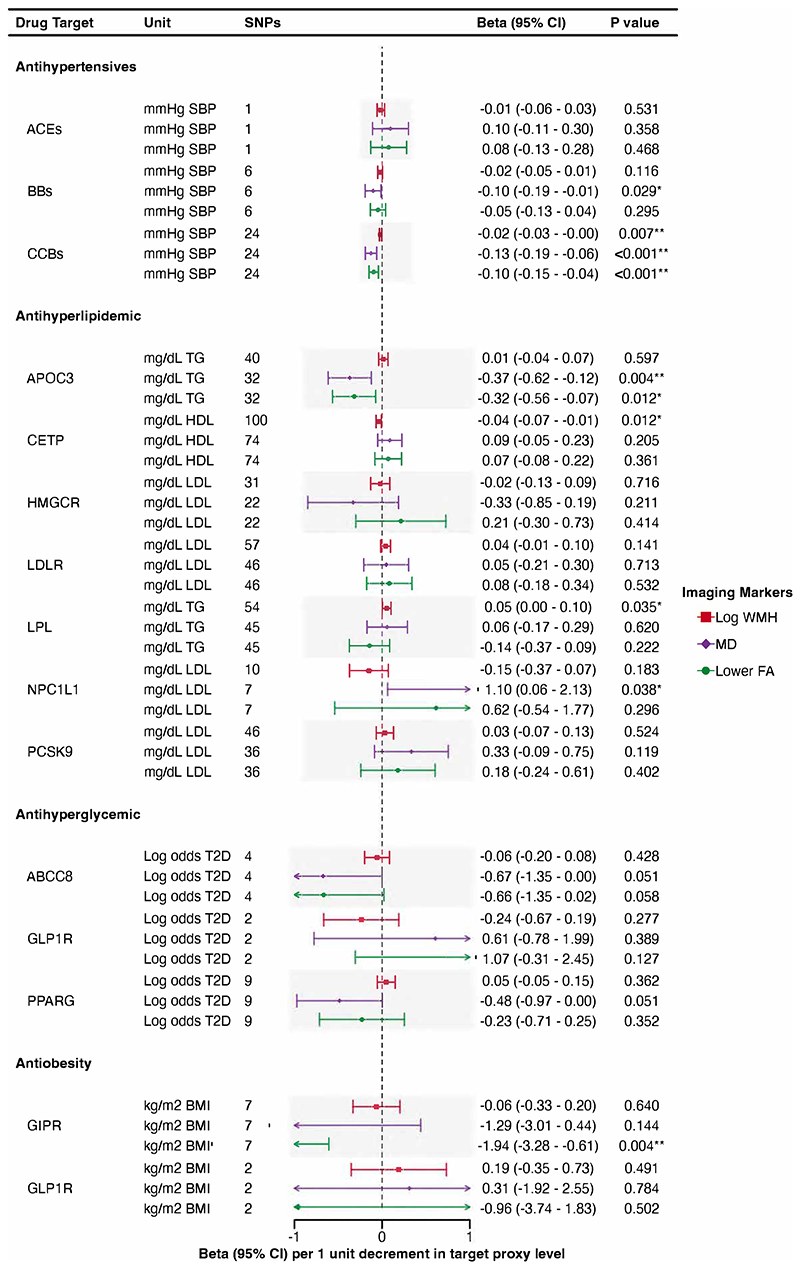
Associations of genetically proxied antihypertensive, antihyperlipidemic, antihyperglycemic, and antiobesity therapies with imaging markers of cSVD. WMH indicates white matter hyperintensity; FA, fractional anisotropy; MD, mean diffusivity; SNPs, single nucleotide polymorphisms; ACEs, angiotensin-converting enzyme inhibitors; BBs, beta blockers; CCBs, calcium channel blockers; APOC3, Apolipoprotein C-III inhibitors; CETP, Cholesteryl Ester Transfer Protein inhibitors; HMGCR, HMG-CoA reductase inhibitors; *LDLR*, LDL Receptor; *LPL*, lipoprotein lipase; NPC1L1, NPC1 Like Intracellular Cholesterol Transporter 1 inhibitors; PCSK9, proprotein convertase subtilisin/kexin type 9 inhibitors; ABCC8, ATP binding cassette subfamily C member 8; GLP1R, glucagon like peptide 1 receptor; PPARG, peroxisome proliferator activated receptor gamma; GIPR, gastric inhibitory polypeptide receptor. *Results with a significant *P* value < 0.05. **Results with a significant *P* value < 0.05 after false discovery rate multiple testing correction.

Associations of genetic proxies for the effects of antihypertensive, antihyperlipidemic, antihyperglycemic, and anti-obesity drug targets on LS are shown in Fig. 4, and on imaging markers of cSVD in Fig. 5.

### Antihypertensive drugs

IVW drug MR analysis showed that genetically proxied inhibition of CCBs, equivalent to a 1-mmHg reduction in SBP, were associated with lower WMH and MD, and higher FA (WMH: β, -0.02 [95% CI, -0.03 to -0.00]; MD: β, -0.13 [95% CI, -0.19 to -0.06]; FA: β, -0.10 [95% CI, -0.15 to -0.04]). In contrast, no similar associations were found for ACEs or BBs with imaging markers and there was no evidence of any association of antihypertensive drug targets (ACEs, BBs, CCBs) on LS.

### Antihyperlipidemic drugs

The IVW drug MR analysis indicated that genetically proxied inhibition of CETP, equivalent to a 1-mg/dL increase in HDL, was significantly associated with a lower risk of LS (GIGASTROKE: OR, 0.84 [95% CI, 0.76–0.93]; MRI confirmed: OR, 0.75 [95% CI, 0.62–0.91]), although the association with MRI-confirmed LS did not remain significant after FDR correction (Fig. 4). We also found a nominally significant association with lower WMH (β, -0.04 [95% CI, -0.07 to -0.01]).

Genetic mimicry of LPL enhancement equivalent to a 1-mg/dL reduction in TG was significantly associated with a lower risk of LS (GIGASTROKE: OR, 0.74 [95% CI, 0.58–0.95]; MRI-confirmed: OR, 0.54 [95% CI, 0.39–0.74]). However, the association with LS from GIGASTROKE did not pass FDR correction (Fig. 4). We also found a nominally association with WMH (β, 0.05 [95% CI, 0.00 to 0.10]).

Genetically proxied inhibition of NPC1L1, equivalent to a 1-mg/dL reduction in LDL was nominally associated with a lower risk of LS in GIGASTROKE but not in MRI-confirmed cohort (GIGASTROKE: OR, 0.34 [95% CI, 0.13–0.92]; MRI confirmed: OR, 0.25 [95% CI, 0.04–1.41]). We also found a nominally significant association with higher MD (β, 1.10 [95% CI, 0.06 to 2.13]).

Genetically proxied APOC3 perturbation, equivalent to a 1-mg/dL reduction in TG, was associated with MD and FA (MD: β, -0.37 [95% CI, -0.62 to -0.12]; FA: β, -0.32 [95% CI, -0.56 to -0.07]). However, the association with FA did not pass FDR correction (Fig. 5). No association was found with LS.

### Antihyperglycemic drugs

Genetically proxied inhibition of ABCC8, equivalent to a 1-unit reduction in log odds of type 2 diabetes, was significantly associated with a lower risk of LS in GIGASTROKE but not in the MRI-confirmed cohort (GIGASTROKE: OR, 0.22 [95% CI, 0.10–0.49]; MRI-confirmed: OR, 0.59 [95% CI, 0.15–2.25]).

There were no associations for anti-hyperglycaemic drug targets and MRI markers of cSVD.

### Antiobesity drugs

Genetically proxied GIPR perturbation equivalent to a 1-kg/m^2^ reduction in BMI was associated with a lower risk of LS in GIGASTROKE (OR, 0.10 [95% CI, 0.02–0.51]) but not in the MRI-confirmed LS cohort. We also found a significant association with higher FA (β, -1.94 [95% CI, -3.28 to -0.61]).

### Sensitivity analyses

Similar results were observed using MR-Egger and the weighted median-based method (Supplementary Tables 8 and 9). There was no strong evidence of horizontal pleiotropy (*P* for the Egger intercept > 0.05; Supplementary Tables 9 and 10).

Colocalization analysis suggested that *LPL* (as a TG-lowering drug target), *CETP* (as an HDL-raising drug target), *ABCC8* (as a glucose-lowering drug target), and *GIPR* (as an anti-obesity drug target) were unlikely to share a causal variant with LS (Supplementary Table 11).

Colocalization analysis for MRI markers of cSVD indicated that CCBs and MD associations had a 91% posterior probability of sharing a causal variant within the *CACNB2* locus, while CCBs and WMH associations had an 83% posterior probability of being influenced by distinct causal variants that are in LD with each other within the same locus. There was little evidence of a shared causal variant for *CETP, NPC1L1*, and *APOC3* (Supplementary Table 11). For antihypertensive targets with LS, there might not be sufficient power (Supplementary Table 6).

## Discussion

In this study we used Mendelian randomization to provide a comprehensive assessment of which conventional cardiovascular risk factors show evidence of being causally related to cSVD, in order to guide optimal preventative approaches. We found strong evidence supporting causal effects of blood pressure traits on both LS and cSVD imaging markers. Additionally, genetic evidence supported a detrimental effect of type 2 diabetes, obesity, smoking, higher TG levels, TG to HDL ratio on LS risk, while higher HDL concentrations and moderate-to-vigorous physical activity showed protective effects. We also found evidence of detrimental effects of BMI and smoking, but the protective effect of moderate-to-vigorous physical activity on WMH. No strong evidence supported the effects of most drug targets tested, but some evidence suggested a benefit of blood pressure lowering through CCBs, HDL raising through CETP inhibitors, TG lowering through LPL targeted drugs, and weight loss through GIPR inhibitors.

Hypertension is a key modifiable risk factor for cSVD,^32,33^ and intensive blood pressure lowering has been associated with reduced progression of WMH in the SPRINT trial.^34^ Consistent with this, our findings showed strong associations between all blood pressure markers and both LS and imaging markers of cSVD, with systolic and diastolic blood pressure showing stronger associations than pulse pressure. Secondary analyses of data from randomised controlled trials (RCTs) has suggested differential effect of antihypertensive drug classes on the risk of stroke,^35,36^ possibly due to their impact on blood pressure variability, an independent risk factor for stroke,^37^ and WMH.^38^ A meta-analysis of RCTs of BP-lowering drugs found that blood pressure variability was reduced by CCBs and increased by ACE inhibitors, angiotensin-receptor blockers, and beta-blockers.^39^ Consistent with this, our results suggest a benefit of blood pressure lowering through CCBs on cSVD imaging markers. However, the associations with LS were not significant, in contrast to a previous study using LS data from a multiethnic GWAS meta-analysis using MEGASTROKE data.^33^ This may reflect the lower power of the smaller European LS cohorts compared to larger GWAS of cSVD imaging markers.

Unlike blood pressure, our results showed little support for a causal association between total cholesterol or LDL with cSVD. Previous epidemiological studies have shown conflicting results with some showing possible associations between cholesterol and LDL with cSVD^32^ and others showing no association.^40^ Our results suggest LDL does not play a major casual role of cSVD.

We found a protective effect of higher HDL levels, and a detrimental effect of TG on LS but not on cSVD markers, consistent with previous studies suggesting that higher HDL levels lower the risk of LS in MEGASTROKE and WMH.^40^ Genetic variants increasing HDL at the CETP locus were also associated with reduced LS and WMH risk, which is consistent with a previous study in the MEGASTROKE.^40^ Although CETP inhibitors have shown limited cardiovascular benefits in trials, extended follow-up from the REVEAL trial suggests potential for further development.^41^ However, their impact on LS and cSVD remains unclear.

Our finding that genetic predisposition to higher TG levels is linked to a higher risk of LS aligns with a previous study in MEGASTROKE.^32^ TG-lowering variants in the LPL locus were associated with a lower risk of MRI-confirmed LS and nominally with WMH. In recent studies, LPL enhancement was associated with a lower risk of coronary disease and type 2 diabetes independently of LDL–lowering genetic mechanisms.^42,43^ While medications like fibrates, omega-3s, and metformin modulate LPL,^44^ recent interest focuses on developing drugs that directly target LPL activation for cardiovascular prevention.^44^

Consistent with epidemiological data implicating T2D as a risk factor for LS, we found genetic associations between several glycaemic markers (T2D, HbA1c, TG:HDL ratio, a marker of insulin resistance) and LS, but not with cSVD neuroimaging markers. This suggest that T2D may be a more important risk factor for LS than the more chronic features of cSVD seen on MRI. Our drug target MR analysis showed glucose-lowering variants in the ABCC8 locus (sulfonylureas target) were associated to lower LS risk from the GIGASTROKE. A previous study reported that the use of sulfonylureas before and during the acute phase of cerebral ischemia may have a significant, clinically meaningful effect on stroke outcomes in patients with type 2 diabetes.^45^ However, the improvement was not observed in the subgroup of patients with LS, which may be explained by the small size of lacunar infarcts, especially in the treatment group.^45^ Although we did not find a significant proxy in the *SLC5A2* locus, recent evidence suggests SGLT2 inhibition may reduce LS risk and WMH volumes.^46^

Despite the conflicting epidemiological evidence,^32,47,48^ we found support for causal associations of BMI with both LS and cSVD markers, and WMR with LS, supporting a role for obesity in cSVD. BMI-lowering variants in the GIPR locus were also associated to lower LS risk from GIGASTROKE and FA. Although the exact mechanism by which GIP inhibition might lower the risk of LS and FA is unclear, evidence suggests that GIP may play a protective role in vascular damage by reducing circulating inflammatory cytokines and endothelial inflammation.^49^ Additionally, dual GIPR antagonist and GLP-1R agonist therapies have shown greater efficacy in weight loss and glucose control.^50,51^

Inconsistent results on smoking and cSVD may be partly due to non-standardized smoking definitions.^52–54^ Our study examined smoking initiation, cigarettes per day, and the lifetime smoking index—a composite smoking index derived from smoking duration, smoking heaviness, and smoking cessation.^55^ While we found some evidence of a causal association of the lifetime smoking index with LS and WMH, Taylor-Bateman et al.^32^ reported a causal relationship of smoking initiation with WMH. Regarding alcohol consumption, this study was unable to definitively establish any causal links, which is in line with results reported by Taylor-Bateman et al.^32^

Lastly, our study found support for a protective effect of physical activity on LS and WMH. However, a recent review found inconsistent results regarding physical activity’s neuroprotective role in cSVD.^56^ While some studies reported an association between physical activity and cSVD features,^57,58^ others found no significant associations.^59–63^ This could be partially attributed to the inherent limitations of the study design, where healthier cSVD profiles might result from factors that are either independent of or additional to physical activity, which makes it challenging to isolate physical activity as the sole contributing factor to improved cerebrovascular health.^56^

Some risk factors and drug targets in our study did not show significant associations with LS or cSVD imaging markers, which may suggest these factors have limited causal effects or that focusing on these drug targets alone may not be effective for preventing cSVD. However, the null findings could also be influenced by methodological factors, such as insufficient statistical power or disease misclassification. Although our study was adequately powered to detect most expected effects, some associations may have been too subtle to detect, particularly for antihypertensive targets where the power was insufficient. Additionally, LS subtypes in the GIGASTROKE dataset were primarily defined using CT imaging, which may have introduced some misclassification. However, further analysis using MRI-confirmed data did not significantly alter the null results for LS.

Strengths of our study include validation of our findings by replicating them in an MRI-confirmed LS cohort, which is important given that LS subtyping can be inaccurate, especially when using CT-based diagnostic algorithms. However, the number of MRI-confirmed LS cases may have been insufficient to identify some associations and replication in larger cohorts is important. We also examined associations with chronic cSVD features using data from GWAS of a large sample size from the neuroimaging dataset, which is currently the largest available in the UK Biobank.

However, some potential limitations should also be considered when interpreting the results. Firstly, the MR approach examines the effects of small, lifelong changes in genetically predicted levels of a risk factor on an outcome, which is different from the effects of a clinical intervention that may have a more significant impact over a shorter duration. This method also cannot determine the dose-response relationship between drug targets and outcomes. MR analysis is more useful for determining the existence and the direction of associations than for quantifying their magnitude. Secondly, this approach is vulnerable to bias from genetic variants that may have pleiotropic effects on the outcome through unrelated pathways to the exposure being studied. Despite performing a range of sensitivity analyses to mitigate confounding, the possibility of such bias cannot be completely ruled out. Thirdly, there was partial sample overlap among GWAS data sets, such as the UK Biobank, which might increase the type I error rate. However, *F*-statistics for the genetic variants selected as IVs were above 10, suggesting that the bias from this overlap should be negligible. Additionally, with relatively large sample sizes, the potential bias from sample overlap is expected to be minimal.^64^ Fourthly, based on our selection criteria, we did not identify significant genetic proxies for some drug targets, including angiotensin-receptor blockers, thiazide diuretics, dipeptidyl peptidase 4 (DPP-4) inhibitors, and sodium-glucose co-transporter 2 (SGLT2) inhibitors. Future studies using larger GWAS datasets for SBP and diabetes/HbA_1c_ might identify such proxies and thus offer deeper insights into the effects of these drug classes on cSVD. Lastly, the analyses were predominantly conducted on individuals of European ancestry, limiting the generalisability to other ethnic groups.

In summary, we conducted a comprehensive investigation into how conventional cardiovascular risk factors and some of their pharmacological treatments impact LS and imaging markers of cSVD. Our findings highlight the importance of blood pressure, type 2 diabetes and hyperglycaemia, obesity, smoking, and low physical activity as risk factors for cSVD, although some risk factors had stronger associations with LS than with more chronic imaging features of cSVD. These findings strengthen the evidence for targeting these factors in clinical practice to reduce the risk of cSVD progression. Additionally, we observed the potential beneficial effects of CCB-targeted drugs on cSVD imaging markers as well as CETP-targeted, LPL-targeted, and GIPR obesity-targeted drugs on LS. Although these results do not immediately justify changes to current clinical guidelines, they could help researchers prioritise treatments to test in future clinical trials. Furthermore, future research that includes diverse populations would be beneficial to enhance the generalizability of our findings.

## Supplementary Material

Supplementary material

## Figures and Tables

**Table 1:** Descriptive characteristics of the genome-wide association summary statistics included in this Mendelian randomization study.

Study stage	Phenotype(s)	Unit	Dataset	Sample size	Ancestry	Reference
**Instrument Selection**						
**Blood pressure**	SBP, DBP, PP	mmHg	ICBP; UK Biobank	757 601	European	Evangelou et al.^24^
**Lipids**	TC, LDL, HDL, TG	mg/dL	Meta-analysis of 201 studies	1 320 000	European	Graham et al.^25^
	Type 2 diabetes	Log odds	Meta-analysis of 8 studies	148 726 cases; 965 732 controls	European	Vujkovic et al.^27^
**Blood glucose**	HbA1c	percentage	Meta-analysis in MAGIC Consortium	146 806	European	Chen et al.^70^
	TG to HDL ratio, as an insulin resistance marker	-	UK Biobank	402 398	European	Oliveri et al.^71^
**Anthropometrics**	BMI	kg/m^2^	UK Biobank; GAINT Consortium; MVP	1 122 049	European	Huang et al.^29^
	WHR	SD	UK Biobank; GIANT Consortium	694 649	European	Pulit et al.^72^
	ever vs never	SD in Log odds	GSCAN Consortium (>30 cohorts)	1 232 091	European	Liu et al.^73^
**Smoking status**	cigarettes smoked per day	SD	GSCAN Consortium (>30 cohorts)	337 334	European	Liu et al.^73^
	Life-time smoking index	SD	UK Biobank	502 647	European	Wootton et al.^55^
**Drinking status**	drinks per week	SD	GSCAN Consortium (>30 cohorts)	941 280	European	Liu et al.^73^
**Physical activity**	MVPA	Log odds	Meta-analysis of 51 studies	608 595	European	Wang et al.^74^
**Outcomes**						
	LS	Log odds	GIGASTROKE	6811 cases; 1234 808 controls	European	Mishra et al.^75^
**Lacunar stroke**	MRI-confirmed LS	Log odds	UK DNA LS studies 1 and 2, ISGC	3306 cases and 19 955 controls	European	Koohi et al.^13^
	WMH	Log mm^3^	UK Biobank; CHARGE Consortium	55 291	European	Koohi et al.^13^
**Imaging markers of SVD**						
	MD	First PCA	UK Biobank	36 012	European	
	FA	First PCA	UK Biobank	36 460	European	

**Table 2:** Drug classes, their target genes, and genomic regions.

Primary pharmacological action	Drug class	Target genes	Gene region (GRCh37/hg19 by Ensembl)
	Angiotensin I Converting Enzyme Inhibitors	ACE	chr17:61554422-61599205
	Beta Blockers	ADRB1	chr10:115803806-115806667
		CACNA1C	chr12:2079952-2807115
		CACNB2	chr10:18429606-18830798
		CACNB3	chr12:49207577-49222726
		CACNA1D	chr3:53528683-53847760
**Antihypertensive**	Calcium Channel Blockers	CACNA1S	chr1:201008640-201081694
		CACNA2D1	chr7:81575760-82073114
		CACNA2D2	chr3:50400230-50541675
		CACNB1	chr17:37329709-37353956
		CACNB4	chr2:152689285-152955593
		CACNG1	chr17:65040652-65052913
	Angiotensin II Receptor Blockers	AGTR1	chr3:148415571-148460795
	Thiazide Diuretics	SLC12A3	chr16:56899119-56949762
	LDL Receptor	LDLR	chr19:11200038-11244492
	HMG-CoA reductase inhibitors	HMGCR	chr5:74632154-74657929
**Reduced LDL-C**	Proprotein Convertase Subtilisin/Kexin Type 9 (PCSK9) inhibitors	PCSK9	chr1:55505221-55530525
	Cholesterol absorption inhibitors (Target of ezetimibe)	NPC1L1	chr7:44552134-44580914
**Reduced TG**	Lipoprotein Lipase	LPL	chr8:19759228-19824769
	Apolipoprotein C3 inhibitors	APOC3	chr11:116700422-116703788
**Raised HDL-C**	Cholesteryl Ester Transfer Protein (CETP) inhibitors	CETP	chr16:56995762-57017757
	Sulfonylureas	ABCC8	Chr11:17414432–17498449
	Thiazolidinediones	PPARG	Chr3:12328867–12475855
	Glucagon-like peptide-1 (GLP-1) analogues	GLP1R	Chr6:39016574–39055519
**Antihyperglycemic**	Dipeptidyl peptidase 4 (DPP-4) inhibitors	DPP4	chr2:162848751-162931052
	Sodium-glucose co-transporter 2 (SGLT2) inhibitors	SLC5A2	chr16:31494323-31502181
	Glucagon-like peptide-1 (GLP-1) analogues	GLP1R	Chr6: 39016574–39055519
**Anti-obesity**	Gastric Inhibitory Polypeptide Receptor	GIPR	chr19:46171502-46186982

## Data Availability

Individual-level data from the UK Biobank are available through application at http://www.ukbiobank.ac.uk/using-the-resource/. Individual-level data from the NINDS Stroke Genetics Network Study are available to researchers through the Database of Genotypes and Phenotypes (dbGaP). The new GWAS analyses on imaging markers from UK Biobank have not been previously published. The summary statistics will be made available from the GWAS Catalog (https://www.ebi.ac.uk/gwas/; accession number to be provided after manuscript is accepted). The summary statistics for WMH from the Cohorts for Heart and Aging Research in the Genomic Epidemiology (CHARGE) Consortium, Type 2 diabetes, and BMI can be obtained directly from dbGaP (https://www.ncbi.nlm.nih.gov/gap/). The summary statistics for lacunar stroke from the GIGASTROKE Consortium, blood pressure traits, HbA1C, TG to HDL ratio, smoking behaviour and initiation, drinking, and physical activity can be obtained from the GWAS Catalog (https://www.ebi.ac.uk/gwas/), summary statistics for lipid traits can be obtain from the Global Lipids Genetics Consortium Results (https://csg.sph.umich.edu/willer/public/glgc-lipids2021/), and lifetime smoking GWAS summary data can be obtained from the University of Bristol Research Data Repository (https://data.bris.ac.uk/data/dataset/10i96zb8gm0j81yz0q6ztei23d).
