# Supplementary material for: Optimizing treatment of cardiovascular risk factors in cerebral small vessel disease using genetics
Source: Brain. 2024 Dec 11;148(6):1936–49. doi: 10.1093/brain/awae399 (PMC7617411; doi:10.1093/brain/awae399)
Supplement: awae399_Supplementary_Data [file awae399_supplementary_data.pdf]

SUPPLEMENTARY ONLINE CONTENT

Optimising treatment of cardiovascular risk factors in cerebral small vessel disease using genetics

Table of Contents

Supplementary Table 1 Genetic variants used as instruments for cardiovascular risk factors **2**

Supplementary Table 2 Genetic variants used as instruments to proxy SBP-lowering drug targets **172**

Supplementary Table 3 Genetic variants used as instruments to proxy lipid-lowering drug targets **173**

Supplementary Table 4 Genetic variants used as instruments to proxy glucose-lowering drug targets **186**

Supplementary Table 5 Genetic variants used as instruments to proxy anti-obesity drug targets **187**

Supplementary Table 6 Statistical power estimates for MR analyses **188**

Supplementary Table 7 Sensitivity analyses for the Mendelian randomization associations of genetically predicted cardiovascular risk factors with lacunar stroke **189**

Supplementary Table 8 Sensitivity analyses for the Mendelian randomization associations of genetically predicted cardiovascular risk factors with imaging markers of cSVD **190**

Supplementary Table 9 Sensitivity analyses for the Mendelian randomization associations of genetically proxied antihypertensive, antihyperlipidemic, antihyperglycemic, and antiobesity therapies with lacunar stroke **191**

Supplementary Table 10 Sensitivity analyses for the Mendelian randomization associations of genetically proxied antihypertensive, antihyperlipidemic, antihyperglycemic, and antiobesity therapies with imaging markers of cSVD **192**

Supplementary Table 11 Results of colocalization analysis **193**

Supplementary Table 1 Genetic variants used as instruments for cardiovascular risk factors

Phenotype	Trait	SNP	Chr	Position (GRCh37/hg19)	EA	NEA	BETA	SE	P-value	R2	F
Blood Pressure	SBP	rs7796	1	1684169	C	G	0.338	0.031	5.0e-27	1.6e-04	116.2
Blood Pressure	SBP	rs451061	1	2075068	C	G	-0.193	0.031	6.9e-10	5.2e-05	38.1
Blood Pressure	SBP	rs2493291	1	3329384	T	C	0.421	0.044	1.8e-21	1.3e-04	90.6
Blood Pressure	SBP	rs9661802	1	6678864	A	C	0.224	0.032	2.7e-12	6.6e-05	48.9
Blood Pressure	SBP	rs2252865	1	8422676	T	C	0.202	0.032	1.7e-10	5.6e-05	40.7
Blood Pressure	SBP	rs1815614	1	9441838	A	G	0.235	0.031	3.7e-14	7.9e-05	57.4
Blood Pressure	SBP	rs17035646	1	10796547	A	G	0.513	0.032	1.4e-56	3.4e-04	250.8
Blood Pressure	SBP	rs6699618	1	11881441	C	G	0.911	0.041	1.7e-109	6.7e-04	494.2
Blood Pressure	SBP	rs12411044	1	11914074	A	G	-0.421	0.037	6.4e-30	1.7e-04	128.9
Blood Pressure	SBP	rs6666373	1	15939884	A	T	0.337	0.034	1.1e-23	1.4e-04	100.4
Blood Pressure	SBP	rs698893	1	16302982	A	G	0.225	0.030	1.8e-13	7.4e-05	54.4
Blood Pressure	SBP	rs79932633	1	22282824	A	C	-0.274	0.049	3.1e-08	4.2e-05	30.7
Blood Pressure	SBP	rs2807337	1	22577371	T	C	0.186	0.031	2.8e-09	4.8e-05	35.2
Blood Pressure	SBP	rs72654647	1	25022314	A	G	0.211	0.035	2.6e-09	4.8e-05	35.4
Blood Pressure	SBP	rs4320727	1	25351581	A	G	0.234	0.031	9.9e-14	7.5e-05	55.4
Blood Pressure	SBP	rs79598313	1	27284913	T	C	0.665	0.100	2.4e-11	6.1e-05	44.6
Blood Pressure	SBP	rs11247642	1	27699012	T	C	-0.273	0.043	2.2e-10	5.6e-05	40.4
Blood Pressure	SBP	rs4908348	1	28706949	T	G	0.237	0.033	8.1e-13	7.0e-05	51.4
Blood Pressure	SBP	rs72661887	1	38416310	T	C	0.246	0.031	3.9e-15	8.5e-05	62.0
Blood Pressure	SBP	rs1408945	1	42364877	T	G	-0.320	0.030	8.3e-26	1.5e-04	110.5
Blood Pressure	SBP	rs839755	1	43856410	A	C	-0.266	0.031	5.4e-18	1.0e-04	74.9
Blood Pressure	SBP	rs4926923	1	48109225	T	C	0.320	0.054	2.6e-09	4.9e-05	35.5
Blood Pressure	SBP	rs11579440	1	49052423	T	C	0.267	0.043	3.2e-10	5.4e-05	39.6
Blood Pressure	SBP	rs778124	1	56606206	A	G	0.296	0.031	1.4e-21	1.2e-04	90.9
Blood Pressure	SBP	rs56186267	1	56970418	A	C	-0.312	0.052	2.4e-09	4.8e-05	35.6
Blood Pressure	SBP	rs61772592	1	56979681	A	G	-0.318	0.046	2.9e-12	6.6e-05	48.9
Blood Pressure	SBP	rs12758643	1	59669918	T	C	-0.275	0.033	1.1e-16	9.3e-05	68.6
Blood Pressure	SBP	rs12136922	1	67007389	A	G	0.203	0.030	2.7e-11	6.2e-05	44.5
Blood Pressure	SBP	rs658780	1	78555928	T	G	-0.203	0.035	5.3e-09	4.6e-05	34.2
Blood Pressure	SBP	rs2892987	1	87932732	A	G	0.322	0.054	2.9e-09	4.8e-05	35.2
Blood Pressure	SBP	rs6699416	1	88661096	A	G	0.241	0.041	3.0e-09	4.8e-05	35.1
Blood Pressure	SBP	rs786923	1	89242954	T	C	-0.308	0.031	2.8e-23	1.3e-04	98.8
Blood Pressure	SBP	rs7514579	1	94051350	A	C	0.224	0.036	5.5e-10	5.2e-05	38.6
Blood Pressure	SBP	rs10776752	1	113044328	T	G	0.821	0.058	4.6e-46	2.8e-04	203.2
Blood Pressure	SBP	rs773432	1	113541836	C	G	-0.188	0.030	6.6e-10	5.2e-05	38.1
Blood Pressure	SBP	rs59980837	1	115827266	T	G	1.100	0.116	3.3e-21	1.2e-04	89.4
Blood Pressure	SBP	rs11102919	1	115839778	T	C	-0.434	0.061	1.1e-12	6.9e-05	50.6
Blood Pressure	SBP	rs494697	1	115967523	A	T	0.488	0.079	5.3e-10	5.3e-05	38.5
Blood Pressure	SBP	rs11585169	1	150572037	A	T	0.180	0.031	5.3e-09	4.7e-05	34.0
Blood Pressure	SBP	rs76719272	1	156129796	T	C	-0.274	0.046	3.0e-09	4.8e-05	35.3
Blood Pressure	SBP	rs4573493	1	166023209	T	C	0.167	0.030	3.9e-08	4.1e-05	30.3
Blood Pressure	SBP	rs12731646	1	169090660	T	C	-0.189	0.031	7.2e-10	5.1e-05	37.9
Blood Pressure	SBP	rs1043069	1	180859368	T	G	0.234	0.031	5.3e-14	7.7e-05	56.6
Blood Pressure	SBP	rs4651224	1	184585182	T	C	0.199	0.031	9.0e-11	5.7e-05	42.1
Blood Pressure	SBP	rs1007460	1	193510827	T	C	-0.175	0.030	9.8e-09	4.5e-05	32.9
Blood Pressure	SBP	rs12042924	1	197297417	T	C	-0.181	0.030	2.6e-09	4.8e-05	35.6
Blood Pressure	SBP	rs33996239	1	203109801	T	C	-0.366	0.066	3.4e-08	4.1e-05	30.5
Blood Pressure	SBP	rs11120093	1	207211326	T	C	-0.179	0.031	5.1e-09	4.6e-05	34.1
Blood Pressure	SBP	rs2724377	1	207974818	A	G	0.194	0.030	1.3e-10	5.6e-05	41.5
Blood Pressure	SBP	rs7555285	1	209970355	C	G	0.229	0.038	1.1e-09	5.0e-05	37.2
Blood Pressure	SBP	rs1502360	1	217317714	T	G	-0.180	0.032	1.4e-08	4.4e-05	32.1
Blood Pressure	SBP	rs68085857	1	217737629	T	C	0.274	0.036	1.7e-14	8.0e-05	58.9
Blood Pressure	SBP	rs4595370	1	221298122	A	G	-0.209	0.033	1.7e-10	5.5e-05	40.7
Blood Pressure	SBP	rs72632819	1	227496564	T	G	0.186	0.033	2.4e-08	4.2e-05	31.2
Blood Pressure	SBP	rs708117	1	228199902	A	G	0.287	0.030	1.6e-21	1.2e-04	90.6

Phenotype	Trait	SNP	Chr	Position (GRCh37/hg19)	EA	NEA	BETA	SE	P-value	R2	F
Blood Pressure	SBP	rs699	1	230845794	A	G	-0.375	0.031	5.6e-34	2.1e-04	148.1
Blood Pressure	SBP	rs1565440	1	243387788	A	G	0.175	0.031	1.9e-08	4.3e-05	31.5
Blood Pressure	SBP	rs4926499	1	249155909	C	G	0.296	0.044	1.3e-11	6.4e-05	45.8
Blood Pressure	SBP	rs17760259	2	19744462	T	C	-0.265	0.030	2.3e-18	1.0e-04	76.2
Blood Pressure	SBP	rs342130	2	20715656	A	G	0.189	0.031	1.3e-09	5.0e-05	36.8
Blood Pressure	SBP	rs4665624	2	23875990	A	C	0.181	0.032	1.4e-08	4.4e-05	32.3
Blood Pressure	SBP	rs2278483	2	25040082	T	C	0.205	0.036	1.4e-08	4.4e-05	32.3
Blood Pressure	SBP	rs2384063	2	25187115	T	C	0.327	0.036	6.3e-20	1.1e-04	83.7
Blood Pressure	SBP	rs1275988	2	26914364	T	C	-0.541	0.031	4.4e-69	4.2e-04	308.5
Blood Pressure	SBP	rs12053390	2	27514116	A	G	0.218	0.037	4.2e-09	4.7e-05	34.6
Blood Pressure	SBP	rs7562	2	28635740	T	C	0.231	0.030	3.3e-14	7.8e-05	57.5
Blood Pressure	SBP	rs6734118	2	37559355	A	C	-0.323	0.037	1.4e-18	1.1e-04	77.3
Blood Pressure	SBP	rs7593932	2	37567226	A	G	-0.208	0.031	1.5e-11	6.2e-05	45.5
Blood Pressure	SBP	rs2160236	2	40557276	C	G	-0.244	0.032	1.2e-14	8.2e-05	59.7
Blood Pressure	SBP	rs4952919	2	42956896	A	G	0.170	0.030	2.1e-08	4.3e-05	31.3
Blood Pressure	SBP	rs115262049	2	43196694	A	T	0.589	0.055	1.3e-26	1.5e-04	114.0
Blood Pressure	SBP	rs6714150	2	43389409	A	G	0.247	0.032	4.4e-15	8.3e-05	61.4
Blood Pressure	SBP	rs6737690	2	43643865	A	C	-0.232	0.034	1.7e-11	6.2e-05	45.4
Blood Pressure	SBP	rs687914	2	45878760	T	G	0.248	0.035	1.9e-12	6.7e-05	49.6
Blood Pressure	SBP	rs10189186	2	53025757	A	G	0.189	0.030	3.9e-10	5.3e-05	39.3
Blood Pressure	SBP	rs13016772	2	55779476	T	C	0.252	0.035	1.2e-12	6.8e-05	50.5
Blood Pressure	SBP	rs1019264	2	60012802	A	G	-0.187	0.031	2.0e-09	4.9e-05	36.0
Blood Pressure	SBP	rs1177304	2	61388164	C	G	0.187	0.032	5.6e-09	4.6e-05	33.9
Blood Pressure	SBP	rs13015703	2	64281797	T	C	0.208	0.034	1.4e-09	5.0e-05	36.7
Blood Pressure	SBP	rs2249105	2	65287896	A	G	0.293	0.031	7.6e-21	1.2e-04	87.4
Blood Pressure	SBP	rs2300481	2	66782467	T	C	0.198	0.031	1.6e-10	5.5e-05	40.8
Blood Pressure	SBP	rs4671874	2	68355640	C	G	0.190	0.032	1.8e-09	4.9e-05	36.3
Blood Pressure	SBP	rs2311412	2	69010557	T	C	0.176	0.030	7.4e-09	4.6e-05	33.5
Blood Pressure	SBP	rs13383272	2	69639911	A	G	-0.172	0.030	1.7e-08	4.3e-05	31.8
Blood Pressure	SBP	rs2419077	2	71648425	T	G	0.197	0.034	1.1e-08	4.4e-05	32.7
Blood Pressure	SBP	rs4577304	2	73403040	T	C	-0.177	0.030	5.0e-09	4.6e-05	34.2
Blood Pressure	SBP	rs883650	2	85491747	T	C	0.188	0.030	5.2e-10	5.2e-05	38.6
Blood Pressure	SBP	rs72847885	2	86326717	A	G	0.241	0.032	3.1e-14	7.8e-05	57.6
Blood Pressure	SBP	rs72819754	2	106126438	C	G	-0.293	0.052	2.1e-08	4.3e-05	31.5
Blood Pressure	SBP	rs10207726	2	112744260	T	C	-0.214	0.033	8.1e-11	5.7e-05	42.1
Blood Pressure	SBP	rs6737318	2	114083120	A	G	0.235	0.036	1.1e-10	5.6e-05	41.6
Blood Pressure	SBP	rs6723509	2	122000745	T	C	0.253	0.044	7.6e-09	4.5e-05	33.4
Blood Pressure	SBP	rs4456714	2	124885328	T	C	-0.191	0.035	3.2e-08	4.1e-05	30.5
Blood Pressure	SBP	rs17257081	2	135630498	A	G	0.227	0.039	6.4e-09	4.7e-05	33.7
Blood Pressure	SBP	rs7606205	2	144146311	A	C	-0.184	0.033	3.1e-08	4.2e-05	30.6
Blood Pressure	SBP	rs55944332	2	145726621	A	G	-0.261	0.035	1.8e-13	7.3e-05	54.2
Blood Pressure	SBP	rs62169534	2	146897539	T	C	0.190	0.030	3.7e-10	5.3e-05	39.4
Blood Pressure	SBP	rs1234422	2	148800857	A	T	-0.212	0.039	4.6e-08	4.0e-05	29.9
Blood Pressure	SBP	rs55732192	2	162278233	T	G	-0.336	0.052	1.2e-10	5.6e-05	41.5
Blood Pressure	SBP	rs995614	2	164800581	T	C	-0.240	0.032	2.5e-14	7.9e-05	58.3
Blood Pressure	SBP	rs73029563	2	165008166	C	G	-0.514	0.030	4.2e-64	3.9e-04	285.9
Blood Pressure	SBP	rs16849569	2	165392645	A	G	0.281	0.051	4.4e-08	4.1e-05	29.9
Blood Pressure	SBP	rs1562312	2	174953595	T	C	-0.185	0.030	8.5e-10	5.1e-05	37.6
Blood Pressure	SBP	rs72914576	2	175529967	C	G	-0.292	0.039	5.4e-14	7.7e-05	56.6
Blood Pressure	SBP	rs34727427	2	177016728	T	C	-0.235	0.032	4.0e-13	7.1e-05	52.7
Blood Pressure	SBP	rs1837164	2	178716601	A	T	0.182	0.031	4.7e-09	4.7e-05	34.4
Blood Pressure	SBP	rs1486236	2	180739450	A	C	-0.234	0.032	3.0e-13	7.2e-05	53.1
Blood Pressure	SBP	rs10184839	2	181946115	A	T	-0.185	0.033	2.4e-08	4.2e-05	31.2
Blood Pressure	SBP	rs1921151	2	182993609	A	G	0.300	0.038	1.3e-15	8.7e-05	64.0
Blood Pressure	SBP	rs11679657	2	183183111	T	G	0.206	0.035	6.7e-09	4.5e-05	33.6
Blood Pressure	SBP	rs6739913	2	185033065	A	G	-0.182	0.033	4.6e-08	4.0e-05	29.8

Phenotype	Trait	SNP	Chr	Position (GRCh37/hg19)	EA	NEA	BETA	SE	P-value	R2	F
Blood Pressure	SBP	rs28558491	2	187816321	T	C	-0.211	0.034	7.5e-10	5.2e-05	38.0
Blood Pressure	SBP	rs13412750	2	191634958	A	G	-0.289	0.034	2.3e-17	9.8e-05	71.8
Blood Pressure	SBP	rs296797	2	201102905	T	C	0.216	0.031	2.2e-12	6.7e-05	49.2
Blood Pressure	SBP	rs12693982	2	204085635	T	C	0.258	0.031	7.5e-17	9.4e-05	69.4
Blood Pressure	SBP	rs11690717	2	205093360	T	G	-0.202	0.032	1.9e-10	5.5e-05	40.6
Blood Pressure	SBP	rs3845811	2	208521512	C	G	-0.294	0.031	1.9e-21	1.2e-04	90.6
Blood Pressure	SBP	rs1047891	2	211540507	A	C	-0.253	0.033	1.4e-14	8.1e-05	59.4
Blood Pressure	SBP	rs12694277	2	213188795	T	C	-0.202	0.034	1.8e-09	4.9e-05	36.3
Blood Pressure	SBP	rs112905092	2	214012225	T	C	0.706	0.129	4.2e-08	4.1e-05	30.1
Blood Pressure	SBP	rs1250259	2	216300482	A	T	-0.313	0.034	7.5e-20	1.1e-04	83.0
Blood Pressure	SBP	rs2161967	2	218680529	T	G	0.284	0.031	2.9e-20	1.2e-04	85.3
Blood Pressure	SBP	rs918948	2	218778972	T	C	-0.201	0.032	2.7e-10	5.4e-05	39.8
Blood Pressure	SBP	rs1870123	2	219187698	A	G	-0.182	0.031	5.3e-09	4.6e-05	34.2
Blood Pressure	SBP	rs2943648	2	227100490	A	G	-0.256	0.031	2.6e-16	9.1e-05	67.0
Blood Pressure	SBP	rs1044822	2	230629138	T	C	-0.248	0.042	5.2e-09	4.6e-05	34.2
Blood Pressure	SBP	rs3754944	2	231279616	A	C	0.177	0.031	9.3e-09	4.5e-05	33.0
Blood Pressure	SBP	rs139354822	2	242344695	T	C	0.612	0.098	3.5e-10	5.5e-05	39.3
Blood Pressure	SBP	rs9848170	3	11495983	C	G	0.323	0.031	7.0e-26	1.5e-04	110.8
Blood Pressure	SBP	rs1797912	3	12470239	A	C	0.174	0.032	3.8e-08	4.1e-05	30.2
Blood Pressure	SBP	rs3729931	3	12626516	A	G	-0.182	0.031	6.3e-09	4.6e-05	33.7
Blood Pressure	SBP	rs34991912	3	14926351	T	C	0.311	0.031	4.0e-24	1.4e-04	102.7
Blood Pressure	SBP	rs2117776	3	14960204	T	G	-0.234	0.037	2.0e-10	5.5e-05	40.4
Blood Pressure	SBP	rs189267552	3	20073193	A	T	-0.866	0.139	4.5e-10	5.3e-05	38.9
Blood Pressure	SBP	rs2643826	3	27562988	T	C	0.447	0.031	1.7e-48	2.9e-04	213.7
Blood Pressure	SBP	rs13073304	3	27620947	A	G	0.771	0.114	1.6e-11	6.2e-05	45.5
Blood Pressure	SBP	rs12638085	3	30405936	A	T	0.219	0.032	5.6e-12	6.5e-05	47.4
Blood Pressure	SBP	rs267517	3	37539090	A	G	-0.261	0.031	7.2e-17	9.4e-05	69.5
Blood Pressure	SBP	rs6788984	3	41107173	A	G	0.300	0.043	3.8e-12	6.5e-05	48.2
Blood Pressure	SBP	rs17252114	3	41212753	T	C	-0.170	0.030	1.8e-08	4.3e-05	31.8
Blood Pressure	SBP	rs9311344	3	43940061	A	G	0.227	0.039	7.5e-09	4.5e-05	33.3
Blood Pressure	SBP	rs139409346	3	46857327	A	G	-0.430	0.078	3.3e-08	4.1e-05	30.5
Blood Pressure	SBP	rs6771917	3	48108442	T	C	-0.379	0.035	1.4e-26	1.5e-04	114.2
Blood Pressure	SBP	rs73088122	3	49432404	T	C	-0.256	0.044	4.8e-09	4.6e-05	34.2
Blood Pressure	SBP	rs3821843	3	53558012	A	G	0.337	0.034	6.6e-24	1.4e-04	101.4
Blood Pressure	SBP	rs2633731	3	53738424	T	C	-0.196	0.031	2.2e-10	5.5e-05	40.4
Blood Pressure	SBP	rs3772219	3	56771251	A	C	0.273	0.032	3.1e-17	9.6e-05	71.2
Blood Pressure	SBP	rs74923473	3	64595506	T	C	0.272	0.047	6.8e-09	4.6e-05	33.7
Blood Pressure	SBP	rs7618284	3	66422246	C	G	-0.189	0.033	1.1e-08	4.4e-05	32.6
Blood Pressure	SBP	rs4499560	3	70920485	A	T	-0.220	0.033	1.5e-11	6.2e-05	45.5
Blood Pressure	SBP	rs9857362	3	74710462	A	C	0.173	0.031	1.6e-08	4.4e-05	31.9
Blood Pressure	SBP	rs1248850	3	84887559	T	C	-0.205	0.030	1.4e-11	6.2e-05	45.6
Blood Pressure	SBP	rs1375564	3	85656311	T	C	0.258	0.032	2.8e-16	9.1e-05	67.0
Blood Pressure	SBP	rs12637573	3	121682388	A	G	-0.173	0.030	9.9e-09	4.5e-05	32.9
Blood Pressure	SBP	rs6806529	3	123049938	A	C	0.177	0.031	1.0e-08	4.4e-05	32.7
Blood Pressure	SBP	rs6438857	3	124557643	T	C	0.274	0.030	3.1e-19	1.1e-04	80.5
Blood Pressure	SBP	rs60672471	3	128125718	T	C	0.278	0.049	1.5e-08	4.3e-05	32.1
Blood Pressure	SBP	rs9875380	3	132780356	T	C	-0.175	0.030	6.5e-09	4.6e-05	33.7
Blood Pressure	SBP	rs9880098	3	133949366	A	G	0.308	0.031	1.6e-23	1.4e-04	100.1
Blood Pressure	SBP	rs863930	3	135949737	T	G	-0.189	0.030	4.0e-10	5.3e-05	39.0
Blood Pressure	SBP	rs34905952	3	138104635	A	G	0.287	0.041	3.0e-12	6.6e-05	48.7
Blood Pressure	SBP	rs73872710	3	141106955	A	T	-0.446	0.058	2.2e-14	7.9e-05	58.5
Blood Pressure	SBP	rs62271373	3	150066540	A	T	0.520	0.066	4.9e-15	8.3e-05	61.2
Blood Pressure	SBP	rs4680113	3	153727337	T	C	0.278	0.041	1.0e-11	6.3e-05	46.4
Blood Pressure	SBP	rs79539362	3	154680449	T	C	0.400	0.050	2.1e-15	8.5e-05	63.1
Blood Pressure	SBP	rs61762319	3	154801978	A	G	0.571	0.100	1.3e-08	4.5e-05	32.4
Blood Pressure	SBP	rs73164066	3	157828781	T	C	-0.217	0.033	3.2e-11	6.0e-05	44.1

Phenotype	Trait	SNP	Chr	Position (GRCh37/hg19)	EA	NEA	BETA	SE	P-value	R2	F
Blood Pressure	SBP	rs56213443	3	160309043	A	C	-0.174	0.031	1.4e-08	4.4e-05	32.1
Blood Pressure	SBP	rs3980686	3	168697602	T	G	-0.500	0.049	1.0e-24	1.4e-04	105.3
Blood Pressure	SBP	rs1290784	3	169096900	T	C	0.412	0.030	3.0e-42	2.5e-04	185.2
Blood Pressure	SBP	rs6770911	3	169295764	A	C	-0.267	0.033	1.3e-15	8.7e-05	63.9
Blood Pressure	SBP	rs73171158	3	176927949	T	C	-0.176	0.032	3.5e-08	4.1e-05	30.4
Blood Pressure	SBP	rs262986	3	183435713	A	G	-0.237	0.030	7.7e-15	8.2e-05	60.4
Blood Pressure	SBP	rs13091418	3	185329756	C	G	-0.223	0.032	6.1e-12	6.4e-05	47.2
Blood Pressure	SBP	rs80045342	3	186177114	A	G	-0.334	0.060	2.1e-08	4.3e-05	31.4
Blood Pressure	SBP	rs9869437	3	196228360	A	C	-0.200	0.032	3.2e-10	5.4e-05	39.6
Blood Pressure	SBP	rs1290933	4	2668217	A	C	-0.285	0.033	3.2e-18	1.0e-04	75.8
Blood Pressure	SBP	rs2073505	4	3444503	A	G	0.361	0.060	1.6e-09	4.9e-05	36.4
Blood Pressure	SBP	rs2610990	4	18008232	A	G	-0.290	0.034	2.9e-17	9.7e-05	71.6
Blood Pressure	SBP	rs55924432	4	26812737	T	C	0.265	0.032	5.7e-17	9.5e-05	69.9
Blood Pressure	SBP	rs2291434	4	38387244	T	G	-0.262	0.030	5.1e-18	1.0e-04	74.9
Blood Pressure	SBP	rs62301873	4	40603821	A	G	-0.300	0.050	1.6e-09	4.9e-05	36.3
Blood Pressure	SBP	rs12511987	4	46595623	T	G	-0.233	0.040	5.4e-09	4.6e-05	34.1
Blood Pressure	SBP	rs62309747	4	48713862	A	G	-0.224	0.030	1.6e-13	7.4e-05	54.5
Blood Pressure	SBP	rs61794248	4	52680923	T	G	0.203	0.036	1.3e-08	4.4e-05	32.3
Blood Pressure	SBP	rs60991988	4	54801228	T	G	0.379	0.050	2.8e-14	7.9e-05	57.9
Blood Pressure	SBP	rs7682912	4	55085620	T	G	-0.218	0.037	2.9e-09	4.8e-05	35.3
Blood Pressure	SBP	rs525271	4	56248954	T	C	0.234	0.033	1.7e-12	6.7e-05	49.8
Blood Pressure	SBP	rs13107261	4	63768826	A	G	-0.178	0.031	1.6e-08	4.4e-05	32.1
Blood Pressure	SBP	rs10008637	4	77414144	T	C	0.216	0.030	9.2e-13	6.9e-05	51.0
Blood Pressure	SBP	rs9992370	4	81092147	T	C	0.203	0.036	2.1e-08	4.3e-05	31.4
Blood Pressure	SBP	rs12509595	4	81182554	T	C	-0.837	0.033	2.6e-138	8.5e-04	627.5
Blood Pressure	SBP	rs1379617	4	83952862	T	C	0.217	0.036	1.5e-09	5.0e-05	36.4
Blood Pressure	SBP	rs17010957	4	86719165	T	C	-0.534	0.043	1.8e-35	2.1e-04	154.2
Blood Pressure	SBP	rs13149209	4	89750668	T	C	0.281	0.037	2.0e-14	8.0e-05	58.6
Blood Pressure	SBP	rs1347345	4	95938386	A	G	-0.181	0.031	6.9e-09	4.6e-05	33.5
Blood Pressure	SBP	rs1229984	4	100239319	T	C	-0.599	0.090	2.4e-11	6.2e-05	44.6
Blood Pressure	SBP	rs1527351	4	102084331	T	C	0.208	0.032	1.0e-10	5.7e-05	41.8
Blood Pressure	SBP	rs13107325	4	103188709	T	C	-0.909	0.059	4.2e-53	3.2e-04	235.6
Blood Pressure	SBP	rs56388530	4	106910958	T	C	0.416	0.036	2.8e-31	1.8e-04	135.3
Blood Pressure	SBP	rs4245930	4	109038654	A	G	-0.202	0.031	1.0e-10	5.7e-05	41.7
Blood Pressure	SBP	rs35297017	4	111204358	A	C	0.662	0.111	2.6e-09	4.8e-05	35.5
Blood Pressure	SBP	rs1879057	4	111335722	T	C	-0.325	0.038	1.7e-17	1.0e-04	72.4
Blood Pressure	SBP	rs6533524	4	111407451	A	G	-0.206	0.031	2.0e-11	6.1e-05	45.0
Blood Pressure	SBP	rs4834792	4	120555696	A	T	0.197	0.030	7.2e-11	5.8e-05	42.4
Blood Pressure	SBP	rs3097937	4	124794644	A	T	0.223	0.038	4.9e-09	4.6e-05	34.1
Blood Pressure	SBP	rs7439567	4	138464842	T	C	0.254	0.031	2.3e-16	9.2e-05	67.4
Blood Pressure	SBP	rs72719160	4	144051276	A	T	-0.224	0.032	4.3e-12	6.5e-05	47.9
Blood Pressure	SBP	rs11933087	4	145722862	A	T	-0.211	0.032	5.4e-11	5.9e-05	43.1
Blood Pressure	SBP	rs13128814	4	146801002	A	G	-0.217	0.030	1.2e-12	6.9e-05	50.5
Blood Pressure	SBP	rs13143677	4	148419040	A	G	0.224	0.033	1.8e-11	6.1e-05	45.1
Blood Pressure	SBP	rs6823767	4	151295085	T	C	-0.213	0.034	4.4e-10	5.3e-05	39.0
Blood Pressure	SBP	rs7665985	4	153006312	T	C	0.183	0.032	1.4e-08	4.4e-05	32.2
Blood Pressure	SBP	rs7683728	4	156402654	T	C	-0.365	0.030	2.4e-33	2.0e-04	144.5
Blood Pressure	SBP	rs11732143	4	156448893	A	G	0.250	0.034	2.9e-13	7.2e-05	53.4
Blood Pressure	SBP	rs12643599	4	156639846	A	G	0.313	0.031	1.2e-23	1.4e-04	100.3
Blood Pressure	SBP	rs17035181	4	157678511	T	G	0.307	0.043	7.6e-13	7.0e-05	51.3
Blood Pressure	SBP	rs869396	4	169688000	A	C	-0.212	0.030	4.1e-12	6.5e-05	48.1
Blood Pressure	SBP	rs3932940	5	202282	T	C	-0.188	0.033	1.3e-08	4.7e-05	32.4
Blood Pressure	SBP	rs10069690	5	1279790	T	C	0.310	0.037	4.5e-17	1.0e-04	70.5
Blood Pressure	SBP	rs954767	5	3706050	A	C	-0.213	0.035	7.5e-10	5.2e-05	37.8
Blood Pressure	SBP	rs7725413	5	15695987	T	C	-0.198	0.036	3.1e-08	4.1e-05	30.6
Blood Pressure	SBP	rs1458051	5	32689472	A	G	-0.430	0.037	1.9e-31	1.8e-04	136.3

Phenotype	Trait	SNP	Chr	Position (GRCh37/hg19)	EA	NEA	BETA	SE	P-value	R2	F
Blood Pressure	SBP	rs12656497	5	32831939	T	C	-0.638	0.031	7.1e-96	5.9e-04	432.2
Blood Pressure	SBP	rs10076377	5	33204461	A	G	-0.257	0.034	1.8e-14	7.9e-05	58.6
Blood Pressure	SBP	rs767717	5	50880788	T	C	0.220	0.031	1.9e-12	6.7e-05	49.7
Blood Pressure	SBP	rs1694068	5	53283630	A	T	0.266	0.031	1.2e-17	9.9e-05	73.0
Blood Pressure	SBP	rs464605	5	55807370	T	C	0.208	0.035	2.1e-09	4.9e-05	35.8
Blood Pressure	SBP	rs13179413	5	55868097	T	C	0.224	0.035	1.1e-10	5.6e-05	41.6
Blood Pressure	SBP	rs1848510	5	57754005	A	G	0.184	0.032	5.7e-09	4.6e-05	33.9
Blood Pressure	SBP	rs12657950	5	61940569	T	C	0.455	0.059	1.3e-14	8.1e-05	59.5
Blood Pressure	SBP	rs4700636	5	63841538	A	G	-0.210	0.035	3.2e-09	4.8e-05	35.1
Blood Pressure	SBP	rs6875372	5	64079015	A	T	0.189	0.030	4.8e-10	5.2e-05	38.7
Blood Pressure	SBP	rs4286632	5	66291370	A	G	0.211	0.034	7.6e-10	5.1e-05	37.8
Blood Pressure	SBP	rs10038774	5	67696345	T	C	-0.208	0.033	2.3e-10	5.4e-05	40.2
Blood Pressure	SBP	rs246973	5	68007803	T	C	0.248	0.034	1.5e-13	7.4e-05	54.8
Blood Pressure	SBP	rs56719405	5	72637748	A	G	0.358	0.065	3.9e-08	4.1e-05	30.2
Blood Pressure	SBP	rs4704514	5	77820081	T	C	0.273	0.034	3.9e-16	9.0e-05	66.2
Blood Pressure	SBP	rs6452769	5	87389027	A	G	-0.314	0.038	7.8e-17	9.4e-05	69.5
Blood Pressure	SBP	rs6884092	5	88109590	A	C	0.278	0.051	3.8e-08	4.1e-05	30.2
Blood Pressure	SBP	rs1852732	5	90412371	C	G	0.203	0.032	1.1e-10	5.6e-05	41.4
Blood Pressure	SBP	rs77151571	5	91843369	T	C	0.574	0.089	1.2e-10	5.6e-05	41.4
Blood Pressure	SBP	rs258951	5	92053867	A	T	0.180	0.031	4.8e-09	4.6e-05	34.2
Blood Pressure	SBP	rs709668	5	96174186	A	G	-0.294	0.038	6.0e-15	8.3e-05	60.9
Blood Pressure	SBP	rs1871190	5	97953719	T	G	0.195	0.032	1.7e-09	4.9e-05	36.4
Blood Pressure	SBP	rs2112453	5	112368522	A	G	-0.188	0.034	3.7e-08	4.1e-05	30.3
Blood Pressure	SBP	rs6894014	5	114393181	T	G	0.275	0.030	1.1e-19	1.1e-04	82.3
Blood Pressure	SBP	rs9885577	5	121194226	T	C	0.231	0.032	6.5e-13	7.0e-05	51.7
Blood Pressure	SBP	rs1624823	5	122475438	A	G	0.337	0.031	4.3e-27	1.6e-04	116.0
Blood Pressure	SBP	rs9327297	5	122835051	C	G	0.275	0.032	8.1e-18	1.0e-04	74.2
Blood Pressure	SBP	rs10463839	5	127519675	A	T	0.240	0.037	7.8e-11	5.7e-05	42.2
Blood Pressure	SBP	rs6892983	5	127845030	A	C	0.343	0.031	7.1e-29	1.7e-04	124.6
Blood Pressure	SBP	rs13165038	5	131813350	T	C	-0.192	0.032	3.3e-09	4.7e-05	35.0
Blood Pressure	SBP	rs3923060	5	138775301	T	C	0.192	0.035	3.6e-08	4.1e-05	30.3
Blood Pressure	SBP	rs260728	5	139143510	A	G	0.291	0.053	5.0e-08	4.0e-05	29.7
Blood Pressure	SBP	rs702395	5	140086677	T	C	0.232	0.030	3.2e-14	7.8e-05	57.8
Blood Pressure	SBP	rs2913920	5	141726983	T	C	0.242	0.036	1.6e-11	6.2e-05	45.4
Blood Pressure	SBP	rs994446	5	148348395	A	G	-0.347	0.037	1.9e-20	1.2e-04	85.9
Blood Pressure	SBP	rs11168087	5	148487534	A	G	0.223	0.035	3.2e-10	5.4e-05	39.6
Blood Pressure	SBP	rs157678	5	156145654	A	T	-0.183	0.033	2.7e-08	4.2e-05	30.9
Blood Pressure	SBP	rs1957563	5	157474590	T	C	0.363	0.034	2.3e-26	1.5e-04	112.6
Blood Pressure	SBP	rs11960210	5	157817634	T	C	0.473	0.031	1.3e-51	3.1e-04	228.1
Blood Pressure	SBP	rs2901003	5	157899616	A	G	-0.356	0.045	4.7e-15	8.5e-05	61.5
Blood Pressure	SBP	rs1368298	5	158204425	A	G	0.308	0.030	4.8e-24	1.4e-04	102.3
Blood Pressure	SBP	rs17057329	5	159433524	A	C	0.864	0.154	2.1e-08	4.4e-05	31.4
Blood Pressure	SBP	rs3860770	5	173301427	A	G	-0.266	0.033	1.2e-15	8.7e-05	64.0
Blood Pressure	SBP	rs12153395	5	179411477	A	G	-0.330	0.049	1.1e-11	6.3e-05	46.2
Blood Pressure	SBP	rs2745599	6	1613686	A	G	0.216	0.032	9.0e-12	6.4e-05	46.6
Blood Pressure	SBP	rs1575290	6	7715689	T	C	0.197	0.030	5.6e-11	5.8e-05	43.0
Blood Pressure	SBP	rs9349379	6	12903957	A	G	0.266	0.031	1.3e-17	9.9e-05	72.9
Blood Pressure	SBP	rs179972	6	16362692	T	C	0.183	0.031	5.5e-09	4.7e-05	33.9
Blood Pressure	SBP	rs9368222	6	20686996	A	C	0.228	0.034	1.8e-11	6.1e-05	45.3
Blood Pressure	SBP	rs492044	6	22060799	T	C	0.195	0.032	5.2e-10	5.2e-05	38.5
Blood Pressure	SBP	rs7746623	6	22134989	A	T	-0.220	0.031	1.5e-12	6.8e-05	50.2
Blood Pressure	SBP	rs2744133	6	22392260	A	G	0.288	0.034	1.3e-17	9.9e-05	73.0
Blood Pressure	SBP	rs12661604	6	23181119	A	G	0.178	0.031	9.9e-09	4.4e-05	32.8
Blood Pressure	SBP	rs4712955	6	25684279	A	G	-0.190	0.031	7.4e-10	5.1e-05	37.8
Blood Pressure	SBP	rs2032451	6	26092170	T	G	0.542	0.042	1.6e-37	2.2e-04	163.7
Blood Pressure	SBP	rs74931492	6	27157050	T	C	0.516	0.084	8.6e-10	5.1e-05	37.6

Phenotype	Trait	SNP	Chr	Position (GRCh37/hg19)	EA	NEA	BETA	SE	P-value	R2	F
Blood Pressure	SBP	rs9257594	6	29152970	T	C	0.217	0.039	1.9e-08	4.7e-05	31.6
Blood Pressure	SBP	rs2743925	6	29698050	A	G	-0.266	0.042	1.9e-10	5.7e-05	40.6
Blood Pressure	SBP	rs2394419	6	31067146	A	G	0.259	0.041	2.8e-10	5.5e-05	39.8
Blood Pressure	SBP	rs6904670	6	31192797	A	G	-0.240	0.033	2.2e-13	8.2e-05	53.9
Blood Pressure	SBP	rs2753960	6	31762844	T	G	0.447	0.031	2.7e-47	2.9e-04	208.9
Blood Pressure	SBP	rs204893	6	32094593	T	C	-0.374	0.031	9.8e-34	2.0e-04	146.3
Blood Pressure	SBP	rs114472600	6	34166533	T	C	-0.465	0.077	1.3e-09	5.0e-05	36.8
Blood Pressure	SBP	rs2395622	6	35388758	T	C	-0.227	0.039	7.2e-09	4.5e-05	33.4
Blood Pressure	SBP	rs9394578	6	39165859	A	C	0.193	0.035	2.6e-08	4.2e-05	31.0
Blood Pressure	SBP	rs145211473	6	39242163	A	G	0.751	0.112	1.9e-11	6.2e-05	45.1
Blood Pressure	SBP	rs115537302	6	39242719	A	C	0.331	0.058	1.1e-08	4.4e-05	32.6
Blood Pressure	SBP	rs7763558	6	43349215	A	G	0.336	0.032	1.2e-25	1.5e-04	109.8
Blood Pressure	SBP	rs78648104	6	50683009	T	C	-0.429	0.054	2.4e-15	8.5e-05	62.8
Blood Pressure	SBP	rs7772884	6	51040926	T	C	0.168	0.030	2.2e-08	4.2e-05	31.3
Blood Pressure	SBP	rs1474698	6	56064197	T	C	-0.177	0.030	6.7e-09	4.6e-05	33.6
Blood Pressure	SBP	rs1984195	6	79657391	A	G	0.241	0.030	1.8e-15	8.7e-05	63.2
Blood Pressure	SBP	rs9449350	6	82281417	T	C	-0.219	0.032	1.2e-11	6.2e-05	45.9
Blood Pressure	SBP	rs6921291	6	97066242	T	C	0.358	0.038	1.6e-20	1.2e-04	86.2
Blood Pressure	SBP	rs9486916	6	109013930	T	C	0.266	0.038	5.4e-12	6.5e-05	47.6
Blood Pressure	SBP	rs12215904	6	109117491	T	C	-0.219	0.039	2.5e-08	4.2e-05	31.0
Blood Pressure	SBP	rs13207962	6	117817099	A	G	-0.250	0.035	1.8e-12	6.8e-05	49.8
Blood Pressure	SBP	rs62421489	6	118601980	A	G	0.397	0.055	8.3e-13	6.9e-05	51.2
Blood Pressure	SBP	rs10782230	6	126228512	A	G	0.211	0.030	2.9e-12	6.6e-05	48.6
Blood Pressure	SBP	rs2130604	6	126820561	T	G	0.199	0.036	3.2e-08	4.1e-05	30.6
Blood Pressure	SBP	rs9401913	6	127159982	A	G	0.520	0.030	3.7e-65	3.9e-04	290.9
Blood Pressure	SBP	rs9885632	6	131311909	T	C	0.236	0.034	4.4e-12	6.5e-05	47.8
Blood Pressure	SBP	rs2327429	6	134209837	T	C	0.200	0.034	3.2e-09	4.7e-05	35.0
Blood Pressure	SBP	rs7763294	6	140383733	T	G	-0.200	0.032	6.4e-10	5.2e-05	38.3
Blood Pressure	SBP	rs6570530	6	143150223	T	C	0.221	0.031	6.0e-13	7.0e-05	52.0
Blood Pressure	SBP	rs7765526	6	147713764	A	G	0.201	0.031	5.9e-11	5.8e-05	42.9
Blood Pressure	SBP	rs17080102	6	151004770	C	G	-0.808	0.059	3.5e-42	2.5e-04	185.3
Blood Pressure	SBP	rs6557155	6	151910126	T	G	0.191	0.031	1.1e-09	5.1e-05	37.1
Blood Pressure	SBP	rs75339025	6	152107155	A	G	-0.295	0.051	7.4e-09	4.6e-05	33.4
Blood Pressure	SBP	rs2077647	6	152129077	T	C	0.169	0.030	2.7e-08	4.2e-05	30.9
Blood Pressure	SBP	rs2695259	6	152859370	A	G	-0.201	0.037	4.8e-08	4.0e-05	29.8
Blood Pressure	SBP	rs509833	6	159711515	A	G	0.329	0.044	7.1e-14	7.6e-05	55.9
Blood Pressure	SBP	rs10214535	6	161720674	A	G	0.181	0.033	3.3e-08	4.1e-05	30.5
Blood Pressure	SBP	rs12661036	6	163737476	T	C	-0.210	0.037	1.8e-08	4.3e-05	31.6
Blood Pressure	SBP	rs6932812	6	166180947	C	G	0.460	0.062	7.2e-14	7.7e-05	56.0
Blood Pressure	SBP	rs9294987	6	169720237	T	C	-0.193	0.031	6.3e-10	5.4e-05	38.2
Blood Pressure	SBP	rs75102927	7	1142862	C	G	0.202	0.035	7.1e-09	4.6e-05	33.6
Blood Pressure	SBP	rs17826049	7	1222859	T	G	-0.621	0.077	1.1e-15	9.2e-05	64.3
Blood Pressure	SBP	rs6959688	7	1966831	A	G	-0.234	0.031	4.2e-14	7.8e-05	57.2
Blood Pressure	SBP	rs10282122	7	2529623	T	C	-0.302	0.033	2.5e-20	1.2e-04	85.3
Blood Pressure	SBP	rs755179	7	2557266	T	C	0.196	0.032	1.5e-09	5.2e-05	36.5
Blood Pressure	SBP	rs73049928	7	4669949	A	G	-0.238	0.039	1.2e-09	5.0e-05	36.9
Blood Pressure	SBP	rs3807925	7	18543250	A	G	-0.186	0.032	5.4e-09	4.6e-05	34.0
Blood Pressure	SBP	rs2107595	7	19049388	A	G	0.418	0.042	7.4e-24	1.4e-04	101.3
Blood Pressure	SBP	rs2069833	7	22767664	T	C	-0.199	0.031	8.3e-11	5.8e-05	42.2
Blood Pressure	SBP	rs12979	7	24738164	C	G	0.274	0.045	1.1e-09	5.0e-05	37.2
Blood Pressure	SBP	rs2290263	7	25887278	A	G	0.268	0.035	3.1e-14	7.8e-05	57.8
Blood Pressure	SBP	rs6977848	7	25989520	T	G	-0.185	0.031	2.1e-09	4.9e-05	35.8
Blood Pressure	SBP	rs3735533	7	27245893	T	C	-0.910	0.058	5.3e-56	3.4e-04	248.7
Blood Pressure	SBP	rs12700814	7	27566315	A	T	-0.218	0.031	1.2e-12	6.9e-05	50.5
Blood Pressure	SBP	rs977184	7	28650761	T	C	-0.184	0.031	4.9e-09	4.7e-05	34.3
Blood Pressure	SBP	rs17171710	7	40440233	T	C	-0.409	0.049	1.1e-16	9.4e-05	68.7

Phenotype	Trait	SNP	Chr	Position (GRCh37/hg19)	EA	NEA	BETA	SE	P-value	R2	F
Blood Pressure	SBP	rs11977526	7	46008110	A	G	-0.321	0.031	6.6e-25	1.4e-04	106.1
Blood Pressure	SBP	rs12668436	7	47548893	T	C	-0.215	0.035	7.9e-10	5.1e-05	37.8
Blood Pressure	SBP	rs10950289	7	71429308	A	G	0.234	0.041	1.5e-08	4.3e-05	32.0
Blood Pressure	SBP	rs6963105	7	75097488	A	G	-0.189	0.032	3.8e-09	5.1e-05	34.7
Blood Pressure	SBP	rs848445	7	77572461	T	C	-0.202	0.034	2.3e-09	4.8e-05	35.7
Blood Pressure	SBP	rs7797824	7	90272877	T	C	-0.172	0.031	2.1e-08	4.2e-05	31.3
Blood Pressure	SBP	rs10254757	7	90320790	A	C	0.181	0.031	4.3e-09	4.7e-05	34.5
Blood Pressure	SBP	rs42377	7	92243672	A	G	-0.315	0.033	1.7e-21	1.2e-04	90.7
Blood Pressure	SBP	rs11983783	7	96463812	T	C	0.176	0.031	9.5e-09	4.5e-05	32.9
Blood Pressure	SBP	rs3808356	7	100492322	T	C	0.197	0.034	4.9e-09	4.7e-05	34.3
Blood Pressure	SBP	rs2392929	7	106414069	T	G	-0.751	0.038	2.0e-87	5.3e-04	392.3
Blood Pressure	SBP	rs6466878	7	123272278	T	C	0.196	0.032	1.5e-09	4.9e-05	36.5
Blood Pressure	SBP	rs3757387	7	128576086	T	C	0.190	0.030	4.0e-10	5.3e-05	39.2
Blood Pressure	SBP	rs11556924	7	129663496	T	C	-0.210	0.032	3.4e-11	6.0e-05	43.8
Blood Pressure	SBP	rs10244142	7	130427388	T	C	0.222	0.033	2.0e-11	6.1e-05	44.9
Blood Pressure	SBP	rs35680304	7	130973495	T	C	0.269	0.031	3.8e-18	1.0e-04	75.5
Blood Pressure	SBP	rs114792874	7	131310080	A	G	-0.538	0.078	5.9e-12	6.6e-05	47.4
Blood Pressure	SBP	rs1790998	7	134138380	A	C	-0.209	0.031	9.1e-12	6.3e-05	46.5
Blood Pressure	SBP	rs141212865	7	139404666	A	C	0.301	0.039	7.7e-15	8.2e-05	60.5
Blood Pressure	SBP	rs73727605	7	149474622	A	G	0.362	0.062	6.6e-09	4.6e-05	33.7
Blood Pressure	SBP	rs1960024	7	150281362	A	G	0.187	0.031	2.0e-09	4.9e-05	36.0
Blood Pressure	SBP	rs3918226	7	150690176	T	C	0.664	0.058	8.5e-31	1.8e-04	133.4
Blood Pressure	SBP	rs10224002	7	151415041	A	G	-0.367	0.034	1.3e-27	1.6e-04	118.7
Blood Pressure	SBP	rs1870735	7	155744303	C	G	0.206	0.031	3.6e-11	6.0e-05	43.9
Blood Pressure	SBP	rs4875958	8	1721090	A	G	0.226	0.034	1.9e-11	6.2e-05	45.1
Blood Pressure	SBP	rs907183	8	8729761	C	G	0.319	0.031	2.2e-25	1.5e-04	108.4
Blood Pressure	SBP	rs35141208	8	9738443	A	G	0.270	0.032	1.0e-16	9.4e-05	69.1
Blood Pressure	SBP	rs1821002	8	10640065	C	G	0.379	0.031	5.2e-35	2.1e-04	152.7
Blood Pressure	SBP	rs2686187	8	11654796	A	G	0.298	0.031	3.3e-22	1.3e-04	94.1
Blood Pressure	SBP	rs2872717	8	23403424	T	C	-0.264	0.036	1.0e-13	7.6e-05	55.2
Blood Pressure	SBP	rs7821832	8	25889446	T	G	0.422	0.035	6.7e-34	2.0e-04	147.2
Blood Pressure	SBP	rs2976281	8	26027462	A	G	0.212	0.032	5.8e-11	5.8e-05	42.8
Blood Pressure	SBP	rs2979470	8	30288272	T	C	0.199	0.030	4.6e-11	5.9e-05	43.5
Blood Pressure	SBP	rs1906672	8	38130025	A	G	0.297	0.036	1.2e-16	9.3e-05	68.6
Blood Pressure	SBP	rs2978456	8	42324765	T	C	-0.180	0.031	9.0e-09	4.5e-05	33.0
Blood Pressure	SBP	rs4873492	8	51947549	T	C	0.343	0.040	1.6e-17	9.8e-05	72.5
Blood Pressure	SBP	rs2354862	8	64501744	A	C	0.251	0.032	2.4e-15	8.6e-05	62.5
Blood Pressure	SBP	rs13253358	8	68920135	T	C	0.213	0.033	1.1e-10	5.6e-05	41.5
Blood Pressure	SBP	rs4738141	8	72469742	A	G	-0.193	0.035	3.1e-08	4.2e-05	30.6
Blood Pressure	SBP	rs2126474	8	76878957	T	G	-0.260	0.031	1.9e-17	9.8e-05	72.2
Blood Pressure	SBP	rs16939357	8	77690563	T	C	0.311	0.042	1.4e-13	7.5e-05	54.7
Blood Pressure	SBP	rs72688070	8	81393697	T	C	-0.270	0.041	2.8e-11	6.0e-05	44.4
Blood Pressure	SBP	rs28464834	8	82797356	A	T	0.248	0.035	8.0e-13	7.0e-05	51.4
Blood Pressure	SBP	rs7009170	8	92149429	T	C	-0.215	0.032	3.0e-11	6.0e-05	44.1
Blood Pressure	SBP	rs10956808	8	92775372	T	G	-0.175	0.031	1.2e-08	4.4e-05	32.5
Blood Pressure	SBP	rs2613203	8	95253197	A	T	-0.268	0.039	5.8e-12	6.4e-05	47.5
Blood Pressure	SBP	rs481887	8	95967838	A	G	-0.197	0.031	1.4e-10	5.6e-05	41.2
Blood Pressure	SBP	rs2978098	8	101676675	A	C	0.223	0.031	3.8e-13	7.2e-05	52.6
Blood Pressure	SBP	rs142449193	8	102750597	T	C	-0.455	0.074	7.9e-10	5.1e-05	37.8
Blood Pressure	SBP	rs35783704	8	105966258	A	G	-0.462	0.051	8.8e-20	1.1e-04	83.0
Blood Pressure	SBP	rs2957468	8	106325360	A	G	0.247	0.032	1.6e-14	8.0e-05	59.0
Blood Pressure	SBP	rs3110053	8	110090956	C	G	0.198	0.031	1.4e-10	5.6e-05	41.2
Blood Pressure	SBP	rs2470004	8	120358445	T	C	-0.345	0.039	1.3e-18	1.1e-04	77.6
Blood Pressure	SBP	rs62523863	8	126520544	A	G	0.269	0.037	2.9e-13	7.3e-05	53.4
Blood Pressure	SBP	rs2608029	8	129170126	C	G	0.180	0.032	1.6e-08	4.3e-05	31.8
Blood Pressure	SBP	rs4598218	8	129483956	T	C	0.191	0.031	1.0e-09	5.1e-05	37.3

Phenotype	Trait	SNP	Chr	Position (GRCh37/hg19)	EA	NEA	BETA	SE	P-value	R2	F
Blood Pressure	SBP	rs7012866	8	135616959	T	G	-0.232	0.030	1.2e-14	8.1e-05	59.7
Blood Pressure	SBP	rs7830900	8	141119151	A	G	-0.228	0.034	1.3e-11	6.2e-05	45.9
Blood Pressure	SBP	rs4961293	8	141812374	T	C	0.227	0.030	7.4e-14	7.6e-05	56.0
Blood Pressure	SBP	rs76735299	8	142396481	A	G	0.414	0.057	2.6e-13	7.3e-05	53.5
Blood Pressure	SBP	rs7463212	8	143991858	A	T	-0.275	0.030	1.8e-19	1.1e-04	81.5
Blood Pressure	SBP	rs520015	9	211762	C	G	0.200	0.030	2.8e-11	5.9e-05	44.3
Blood Pressure	SBP	rs12004440	9	756796	A	G	0.222	0.037	1.8e-09	4.8e-05	36.2
Blood Pressure	SBP	rs927315	9	4117713	T	C	0.169	0.030	2.4e-08	4.2e-05	31.1
Blood Pressure	SBP	rs28558845	9	4334791	C	G	-0.257	0.042	1.2e-09	5.0e-05	37.1
Blood Pressure	SBP	rs1547288	9	9310461	A	G	-0.172	0.031	2.2e-08	4.2e-05	31.3
Blood Pressure	SBP	rs1332813	9	9350706	T	C	0.220	0.031	2.3e-12	6.6e-05	49.2
Blood Pressure	SBP	rs7874646	9	13899209	T	C	-0.206	0.038	4.2e-08	4.0e-05	30.0
Blood Pressure	SBP	rs9886665	9	22942770	T	C	0.205	0.034	2.5e-09	4.8e-05	35.7
Blood Pressure	SBP	rs4553000	9	34223553	T	C	-0.203	0.030	1.1e-11	6.2e-05	46.0
Blood Pressure	SBP	rs76452347	9	35906471	T	C	-0.297	0.040	7.1e-14	7.5e-05	56.1
Blood Pressure	SBP	rs10746963	9	77238558	A	G	-0.218	0.039	2.1e-08	4.2e-05	31.5
Blood Pressure	SBP	rs10820855	9	94201341	T	C	-0.190	0.033	1.0e-08	4.4e-05	32.6
Blood Pressure	SBP	rs7045409	9	95201540	A	T	-0.186	0.031	2.5e-09	4.8e-05	35.4
Blood Pressure	SBP	rs59400568	9	112757346	A	G	0.224	0.038	2.9e-09	4.7e-05	35.3
Blood Pressure	SBP	rs10980408	9	113249071	T	C	-0.761	0.083	3.8e-20	1.1e-04	84.6
Blood Pressure	SBP	rs13290326	9	116696625	T	C	-0.212	0.030	1.5e-12	6.7e-05	50.0
Blood Pressure	SBP	rs4978572	9	116943302	C	G	-0.174	0.031	1.6e-08	4.3e-05	31.9
Blood Pressure	SBP	rs2093324	9	119359745	A	G	-0.180	0.031	9.7e-09	4.4e-05	32.9
Blood Pressure	SBP	rs34025993	9	123516572	A	G	0.223	0.031	4.7e-13	7.0e-05	52.4
Blood Pressure	SBP	rs733329	9	125624164	T	C	0.302	0.045	3.1e-11	6.0e-05	44.2
Blood Pressure	SBP	rs7861040	9	127044135	C	G	-0.189	0.031	1.2e-09	5.0e-05	37.1
Blood Pressure	SBP	rs7858935	9	127986904	A	T	-0.318	0.043	9.5e-14	7.4e-05	55.6
Blood Pressure	SBP	rs7023828	9	128498594	T	C	-0.260	0.031	2.2e-17	9.7e-05	71.9
Blood Pressure	SBP	rs1891730	9	130309028	T	C	-0.181	0.031	7.7e-09	4.5e-05	33.3
Blood Pressure	SBP	rs6271	9	136522274	T	C	-0.555	0.061	1.2e-19	1.1e-04	82.4
Blood Pressure	SBP	rs11145807	9	139520789	A	G	0.214	0.032	3.5e-11	6.1e-05	44.0
Blood Pressure	SBP	rs6560653	9	139894003	A	G	0.179	0.033	4.1e-08	4.0e-05	30.0
Blood Pressure	SBP	rs11252324	10	4124568	T	G	-0.416	0.057	3.6e-13	7.2e-05	52.8
Blood Pressure	SBP	rs11257593	10	12241815	A	G	0.176	0.030	8.6e-09	4.5e-05	33.2
Blood Pressure	SBP	rs2488135	10	18333144	T	C	0.220	0.033	4.1e-11	5.9e-05	43.7
Blood Pressure	SBP	rs1888693	10	18440444	A	G	0.386	0.032	4.7e-34	2.0e-04	148.1
Blood Pressure	SBP	rs142073748	10	18691733	T	C	0.821	0.148	3.2e-08	4.2e-05	30.6
Blood Pressure	SBP	rs12258967	10	18727959	C	G	0.633	0.034	1.1e-78	4.8e-04	352.5
Blood Pressure	SBP	rs7895467	10	18762948	A	G	0.258	0.032	8.3e-16	8.8e-05	64.8
Blood Pressure	SBP	rs3858217	10	24690604	C	G	0.197	0.035	2.6e-08	4.2e-05	31.0
Blood Pressure	SBP	rs3802517	10	28233469	A	T	0.253	0.030	4.6e-17	9.5e-05	70.5
Blood Pressure	SBP	rs813412	10	32284825	T	C	-0.210	0.035	1.9e-09	4.9e-05	36.1
Blood Pressure	SBP	rs4948643	10	45379759	T	C	0.226	0.034	2.4e-11	6.1e-05	44.6
Blood Pressure	SBP	rs34130368	10	48411796	T	G	-0.302	0.050	1.3e-09	5.0e-05	36.8
Blood Pressure	SBP	rs4245599	10	60365755	A	G	-0.179	0.030	4.0e-09	4.7e-05	34.6
Blood Pressure	SBP	rs1177701	10	61624697	A	G	0.172	0.030	1.5e-08	4.3e-05	32.1
Blood Pressure	SBP	rs10761530	10	62390726	T	C	0.200	0.030	2.5e-11	6.0e-05	44.6
Blood Pressure	SBP	rs57946343	10	63499951	T	C	0.716	0.043	2.1e-63	3.8e-04	282.5
Blood Pressure	SBP	rs2236295	10	64564892	T	G	-0.303	0.031	1.0e-22	1.3e-04	96.0
Blood Pressure	SBP	rs10761788	10	65354080	A	G	0.214	0.033	5.8e-11	5.8e-05	43.0
Blood Pressure	SBP	rs17712705	10	69623271	A	G	0.250	0.032	5.7e-15	8.3e-05	61.1
Blood Pressure	SBP	rs2177843	10	75409877	T	C	0.439	0.043	2.8e-24	1.4e-04	103.5
Blood Pressure	SBP	rs2131957	10	75866929	A	C	-0.206	0.031	2.0e-11	6.1e-05	44.9
Blood Pressure	SBP	rs2788293	10	82020788	A	T	0.210	0.030	2.7e-12	6.6e-05	49.0
Blood Pressure	SBP	rs77413490	10	89681688	T	G	0.449	0.076	4.3e-09	4.7e-05	34.5
Blood Pressure	SBP	rs11187142	10	94468685	T	C	0.331	0.050	2.5e-11	6.0e-05	44.6

Phenotype	Trait	SNP	Chr	Position (GRCh37/hg19)	EA	NEA	BETA	SE	P-value	R2	F
Blood Pressure	SBP	rs2797983	10	95899646	C	G	-0.413	0.030	3.9e-42	2.5e-04	184.6
Blood Pressure	SBP	rs2274224	10	96039597	C	G	-0.452	0.030	6.0e-50	3.0e-04	220.8
Blood Pressure	SBP	rs1079504	10	96220393	A	G	-0.201	0.031	6.0e-11	5.8e-05	42.7
Blood Pressure	SBP	rs56085433	10	97326325	A	G	-0.251	0.044	8.2e-09	4.5e-05	33.3
Blood Pressure	SBP	rs603424	10	102075479	A	G	0.233	0.040	6.7e-09	4.6e-05	33.6
Blood Pressure	SBP	rs1006545	10	102553647	T	G	0.685	0.048	3.5e-46	2.8e-04	203.4
Blood Pressure	SBP	rs10883667	10	103384849	A	T	-0.230	0.037	3.6e-10	5.3e-05	39.4
Blood Pressure	SBP	rs77361397	10	103843830	A	G	-0.814	0.129	2.6e-10	5.5e-05	39.9
Blood Pressure	SBP	rs11191580	10	104906211	T	C	1.099	0.055	7.7e-89	5.4e-04	399.6
Blood Pressure	SBP	rs2067831	10	105643223	C	G	-0.238	0.034	2.3e-12	6.7e-05	49.2
Blood Pressure	SBP	rs191784289	10	106894942	T	C	1.229	0.143	9.2e-18	1.0e-04	73.6
Blood Pressure	SBP	rs56087297	10	114799172	A	G	-0.227	0.034	1.6e-11	6.2e-05	45.5
Blood Pressure	SBP	rs151591	10	115713566	A	G	-0.255	0.036	1.3e-12	6.8e-05	50.5
Blood Pressure	SBP	rs1801253	10	115805056	C	G	0.463	0.034	2.8e-41	2.4e-04	180.8
Blood Pressure	SBP	rs11197813	10	118523933	A	G	-0.182	0.033	3.5e-08	4.1e-05	30.4
Blood Pressure	SBP	rs72842207	10	121433675	T	C	-0.203	0.037	3.1e-08	4.1e-05	30.6
Blood Pressure	SBP	rs11592107	10	122968964	A	G	0.302	0.033	1.5e-20	1.2e-04	86.0
Blood Pressure	SBP	rs10788159	10	123030785	A	G	-0.240	0.035	6.4e-12	6.4e-05	47.1
Blood Pressure	SBP	rs72834453	10	124235226	T	G	-0.325	0.046	3.0e-12	6.6e-05	48.7
Blood Pressure	SBP	rs1133400	10	134459388	A	G	-0.298	0.038	2.5e-15	8.7e-05	62.6
Blood Pressure	SBP	rs569550	11	1887068	T	G	-0.577	0.032	1.3e-73	4.6e-04	328.7
Blood Pressure	SBP	rs4930047	11	1995414	T	G	-0.233	0.031	3.0e-14	7.9e-05	57.9
Blood Pressure	SBP	rs117212336	11	2116314	T	C	0.481	0.082	5.1e-09	4.8e-05	34.1
Blood Pressure	SBP	rs3763917	11	8819808	A	C	0.212	0.038	1.8e-08	4.3e-05	31.7
Blood Pressure	SBP	rs1392026	11	9791644	T	C	0.288	0.032	5.3e-20	1.1e-04	83.8
Blood Pressure	SBP	rs10840364	11	10114943	A	C	-0.214	0.035	7.9e-10	5.1e-05	37.7
Blood Pressure	SBP	rs111230791	11	10270621	T	C	-0.478	0.039	5.2e-35	2.1e-04	152.8
Blood Pressure	SBP	rs10832013	11	13295353	T	G	0.190	0.033	5.9e-09	4.6e-05	34.0
Blood Pressure	SBP	rs10832300	11	14788963	A	G	-0.191	0.031	6.2e-10	5.2e-05	38.2
Blood Pressure	SBP	rs2014408	11	16365282	T	C	0.517	0.037	1.3e-43	2.6e-04	192.0
Blood Pressure	SBP	rs7926335	11	16917869	T	C	0.314	0.034	2.5e-20	1.2e-04	85.5
Blood Pressure	SBP	rs12146652	11	17384542	A	C	-0.358	0.031	9.0e-31	1.8e-04	133.1
Blood Pressure	SBP	rs10766533	11	19224677	A	T	0.210	0.034	4.7e-10	5.3e-05	38.8
Blood Pressure	SBP	rs1514086	11	22530162	A	G	-0.433	0.059	3.0e-13	7.2e-05	53.2
Blood Pressure	SBP	rs7125719	11	27288359	C	G	-0.196	0.031	3.5e-10	5.3e-05	39.3
Blood Pressure	SBP	rs11030119	11	27728102	A	G	-0.222	0.033	1.8e-11	6.2e-05	45.2
Blood Pressure	SBP	rs871004	11	28512458	A	G	0.234	0.032	1.6e-13	7.4e-05	54.3
Blood Pressure	SBP	rs11031051	11	30355707	A	C	-0.224	0.033	7.7e-12	6.3e-05	46.8
Blood Pressure	SBP	rs56334134	11	31040126	A	G	0.173	0.032	3.7e-08	4.1e-05	30.3
Blood Pressure	SBP	rs7110547	11	32447347	C	G	-0.215	0.031	6.8e-12	6.5e-05	47.1
Blood Pressure	SBP	rs11604310	11	45351420	T	C	-0.278	0.041	1.5e-11	6.2e-05	45.7
Blood Pressure	SBP	rs72910057	11	46331362	T	G	0.402	0.050	7.0e-16	8.8e-05	65.1
Blood Pressure	SBP	rs2301216	11	47356870	A	T	0.190	0.032	2.9e-09	4.8e-05	35.4
Blood Pressure	SBP	rs7107356	11	47676170	A	G	-0.460	0.030	1.6e-52	3.2e-04	233.3
Blood Pressure	SBP	rs9667596	11	48691193	T	C	0.505	0.045	3.1e-29	1.7e-04	125.9
Blood Pressure	SBP	rs6486003	11	49693773	A	T	-0.438	0.045	1.4e-22	1.3e-04	95.7
Blood Pressure	SBP	rs7107202	11	51519430	A	T	0.394	0.040	1.9e-22	1.3e-04	95.2
Blood Pressure	SBP	rs75905900	11	55113534	A	C	0.417	0.045	1.4e-20	1.2e-04	86.4
Blood Pressure	SBP	rs694455	11	56197898	A	G	-0.391	0.046	1.3e-17	9.9e-05	72.9
Blood Pressure	SBP	rs11229115	11	57521122	A	G	-0.260	0.036	5.0e-13	7.1e-05	52.1
Blood Pressure	SBP	rs2276153	11	58407740	C	G	0.330	0.035	6.6e-21	1.2e-04	88.2
Blood Pressure	SBP	rs7125196	11	61272565	T	C	0.442	0.047	7.3e-21	1.2e-04	87.8
Blood Pressure	SBP	rs4980515	11	63744609	T	C	0.225	0.030	9.7e-14	7.5e-05	55.3
Blood Pressure	SBP	rs1662185	11	64100776	A	G	0.212	0.033	1.9e-10	5.6e-05	40.5
Blood Pressure	SBP	rs2306363	11	65405600	T	G	-0.436	0.038	5.2e-31	1.8e-04	134.3
Blood Pressure	SBP	rs139437879	11	67904333	A	G	-0.716	0.123	6.0e-09	4.7e-05	33.8

Phenotype	Trait	SNP	Chr	Position (GRCh37/hg19)	EA	NEA	BETA	SE	P-value	R2	F
Blood Pressure	SBP	rs314779	11	68097826	T	G	-0.212	0.033	1.8e-10	5.5e-05	40.7
Blood Pressure	SBP	rs7394746	11	69262428	T	C	0.228	0.030	9.1e-14	7.5e-05	55.7
Blood Pressure	SBP	rs72931748	11	69825414	A	G	0.397	0.053	6.4e-14	7.6e-05	56.2
Blood Pressure	SBP	rs10501410	11	72088806	A	G	0.412	0.061	1.1e-11	6.2e-05	46.1
Blood Pressure	SBP	rs7927515	11	76125330	A	C	0.227	0.032	1.0e-12	6.9e-05	50.7
Blood Pressure	SBP	rs2289124	11	89224477	A	G	-0.308	0.042	1.1e-13	7.5e-05	55.1
Blood Pressure	SBP	rs78107596	11	100425787	C	G	-0.231	0.036	2.1e-10	5.5e-05	40.5
Blood Pressure	SBP	rs604723	11	100610546	T	C	-0.655	0.034	2.5e-83	5.1e-04	373.3
Blood Pressure	SBP	rs4754196	11	107096777	A	G	-0.349	0.030	1.5e-30	1.8e-04	132.4
Blood Pressure	SBP	rs12363917	11	111664401	T	C	0.200	0.034	2.5e-09	4.8e-05	35.6
Blood Pressure	SBP	rs7128707	11	112855354	A	T	-0.225	0.034	7.3e-11	5.7e-05	42.4
Blood Pressure	SBP	rs17119370	11	116097136	A	T	0.221	0.033	2.2e-11	6.1e-05	44.7
Blood Pressure	SBP	rs1076485	11	116772441	T	C	0.339	0.046	1.2e-13	7.5e-05	55.0
Blood Pressure	SBP	rs3897568	11	120403333	T	C	0.216	0.038	1.9e-08	4.3e-05	31.5
Blood Pressure	SBP	rs2156805	11	122610568	A	G	0.178	0.030	4.6e-09	4.7e-05	34.3
Blood Pressure	SBP	rs11222084	11	130273230	A	T	-0.336	0.032	1.8e-26	1.5e-04	113.3
Blood Pressure	SBP	rs7944927	11	130490917	T	C	0.224	0.039	1.2e-08	4.4e-05	32.5
Blood Pressure	SBP	rs78998485	12	434755	C	G	-0.245	0.035	1.5e-12	6.7e-05	50.1
Blood Pressure	SBP	rs2239046	12	2434419	A	G	0.208	0.032	9.6e-11	5.6e-05	41.8
Blood Pressure	SBP	rs2417189	12	12626050	T	G	0.243	0.031	1.0e-14	8.0e-05	59.8
Blood Pressure	SBP	rs2024385	12	12888438	A	T	-0.264	0.031	5.9e-18	1.0e-04	74.5
Blood Pressure	SBP	rs28621435	12	13860990	A	G	-0.298	0.048	6.5e-10	5.1e-05	38.1
Blood Pressure	SBP	rs73067632	12	19888834	A	G	-0.295	0.052	1.7e-08	4.3e-05	31.7
Blood Pressure	SBP	rs11044907	12	20012531	C	G	0.261	0.035	5.3e-14	7.6e-05	56.6
Blood Pressure	SBP	rs2417849	12	20276513	T	C	0.275	0.031	1.8e-18	1.0e-04	76.6
Blood Pressure	SBP	rs73075659	12	20373541	A	G	0.396	0.032	5.5e-35	2.0e-04	152.3
Blood Pressure	SBP	rs11045237	12	20579083	A	C	0.240	0.038	1.6e-10	5.5e-05	41.0
Blood Pressure	SBP	rs7976167	12	24210599	T	C	0.178	0.032	3.8e-08	4.1e-05	30.3
Blood Pressure	SBP	rs2129869	12	26457650	A	T	-0.264	0.036	2.4e-13	7.2e-05	53.6
Blood Pressure	SBP	rs56389811	12	48205358	T	C	-0.280	0.036	4.5e-15	8.2e-05	61.3
Blood Pressure	SBP	rs117913411	12	48254353	A	T	0.556	0.084	3.8e-11	5.9e-05	43.7
Blood Pressure	SBP	rs4760701	12	48698518	A	G	-0.293	0.037	2.0e-15	8.6e-05	63.1
Blood Pressure	SBP	rs150857355	12	49209340	C	G	0.941	0.112	5.2e-17	9.6e-05	70.3
Blood Pressure	SBP	rs10875907	12	49389410	A	G	-0.716	0.122	4.3e-09	4.8e-05	34.5
Blood Pressure	SBP	rs12426261	12	50573037	A	G	0.378	0.031	2.3e-34	2.0e-04	149.3
Blood Pressure	SBP	rs61926302	12	51164363	T	C	0.614	0.077	2.0e-15	8.5e-05	63.1
Blood Pressure	SBP	rs7315980	12	53449321	A	G	0.480	0.056	7.4e-18	9.9e-05	74.1
Blood Pressure	SBP	rs2280446	12	53451952	T	C	-0.216	0.040	4.6e-08	4.0e-05	29.9
Blood Pressure	SBP	rs7134677	12	54441498	T	C	-0.385	0.033	4.5e-31	1.8e-04	134.5
Blood Pressure	SBP	rs10437954	12	58003922	A	G	-0.410	0.053	1.6e-14	7.9e-05	59.0
Blood Pressure	SBP	rs7306710	12	66376091	T	C	-0.243	0.030	1.0e-15	8.6e-05	64.3
Blood Pressure	SBP	rs4143175	12	67782397	T	C	0.219	0.035	5.1e-10	5.2e-05	38.6
Blood Pressure	SBP	rs111983646	12	69838702	A	G	0.253	0.042	2.3e-09	4.8e-05	35.7
Blood Pressure	SBP	rs11178008	12	70364874	T	C	0.225	0.035	1.8e-10	5.4e-05	40.6
Blood Pressure	SBP	rs7963801	12	79685226	T	C	-0.236	0.031	2.9e-14	7.7e-05	57.7
Blood Pressure	SBP	rs4842313	12	79895819	T	C	-0.320	0.045	8.5e-13	6.9e-05	51.2
Blood Pressure	SBP	rs143285018	12	89932434	A	G	0.865	0.124	2.9e-12	6.6e-05	48.8
Blood Pressure	SBP	rs17249754	12	90060586	A	G	-0.845	0.040	1.2e-97	5.9e-04	439.2
Blood Pressure	SBP	rs5742643	12	102837863	T	C	-0.223	0.035	1.5e-10	5.5e-05	40.9
Blood Pressure	SBP	rs11112548	12	105871914	A	T	0.509	0.077	3.3e-11	5.9e-05	44.0
Blood Pressure	SBP	rs9634314	12	110406386	A	G	-0.392	0.057	4.4e-12	6.4e-05	47.9
Blood Pressure	SBP	rs11611512	12	111718382	A	G	-0.590	0.105	1.8e-08	4.3e-05	31.7
Blood Pressure	SBP	rs17626956	12	111735697	A	G	-0.579	0.092	2.7e-10	5.4e-05	39.9
Blood Pressure	SBP	rs7310615	12	111865049	C	G	0.585	0.031	1.3e-81	5.0e-04	365.5
Blood Pressure	SBP	rs11066309	12	112883476	A	G	0.469	0.031	5.0e-51	3.1e-04	226.2
Blood Pressure	SBP	rs73193163	12	113013907	A	T	-0.575	0.097	2.7e-09	4.8e-05	35.4

Phenotype	Trait	SNP	Chr	Position (GRCh37/hg19)	EA	NEA	BETA	SE	P-value	R2	F
Blood Pressure	SBP	rs7964325	12	115368787	C	G	-0.348	0.033	2.9e-26	1.5e-04	112.3
Blood Pressure	SBP	rs35444	12	115552437	A	G	0.437	0.031	3.5e-45	2.7e-04	198.5
Blood Pressure	SBP	rs11067612	12	115915783	A	T	0.300	0.033	7.6e-20	1.1e-04	83.3
Blood Pressure	SBP	rs11067765	12	116212862	A	T	-0.292	0.050	7.1e-09	4.5e-05	33.5
Blood Pressure	SBP	rs12368199	12	121472167	A	G	-0.234	0.042	3.2e-08	4.1e-05	30.5
Blood Pressure	SBP	rs1169078	12	122416254	C	G	-0.197	0.033	1.7e-09	4.9e-05	36.3
Blood Pressure	SBP	rs35754956	12	122511116	A	G	-0.290	0.048	1.1e-09	5.1e-05	37.1
Blood Pressure	SBP	rs117206641	12	133086888	T	C	0.315	0.050	2.7e-10	5.5e-05	40.0
Blood Pressure	SBP	rs9578336	13	21546513	C	G	-0.294	0.048	7.9e-10	5.1e-05	37.8
Blood Pressure	SBP	rs9552466	13	22262231	T	C	-0.182	0.032	1.2e-08	4.4e-05	32.4
Blood Pressure	SBP	rs606950	13	22298923	A	G	0.270	0.031	3.2e-18	1.0e-04	75.6
Blood Pressure	SBP	rs61948066	13	25255212	T	G	0.280	0.047	2.1e-09	4.8e-05	35.9
Blood Pressure	SBP	rs1331012	13	27115424	T	G	0.204	0.034	1.5e-09	4.9e-05	36.5
Blood Pressure	SBP	rs4771092	13	27877792	C	G	-0.183	0.032	1.3e-08	4.3e-05	32.4
Blood Pressure	SBP	rs9508495	13	30146201	T	C	-0.356	0.035	6.3e-24	1.4e-04	101.5
Blood Pressure	SBP	rs9532243	13	32191408	A	C	0.224	0.030	8.2e-14	7.5e-05	55.8
Blood Pressure	SBP	rs4274337	13	41967193	A	G	-0.297	0.041	2.5e-13	7.2e-05	53.4
Blood Pressure	SBP	rs73187291	13	42740063	T	C	0.261	0.047	3.1e-08	4.1e-05	30.6
Blood Pressure	SBP	rs912434	13	47189928	T	G	0.234	0.035	2.5e-11	6.0e-05	44.4
Blood Pressure	SBP	rs9526707	13	51489186	A	G	-0.204	0.032	2.8e-10	5.4e-05	39.8
Blood Pressure	SBP	rs75961402	13	56398286	A	G	0.266	0.042	1.9e-10	5.4e-05	40.5
Blood Pressure	SBP	rs17245822	13	73131694	A	C	-0.190	0.031	1.2e-09	5.0e-05	37.0
Blood Pressure	SBP	rs78474310	13	73826901	A	G	-0.470	0.073	1.5e-10	5.5e-05	41.0
Blood Pressure	SBP	rs1073943	13	74205174	A	G	0.168	0.030	2.8e-08	4.1e-05	30.8
Blood Pressure	SBP	rs1215469	13	80707408	A	C	-0.212	0.036	6.2e-09	4.6e-05	33.9
Blood Pressure	SBP	rs9549328	13	113636156	T	C	0.292	0.036	7.9e-16	8.7e-05	64.8
Blood Pressure	SBP	rs7987213	13	114968246	T	C	-0.183	0.033	3.7e-08	4.1e-05	30.3
Blood Pressure	SBP	rs7331680	13	115000650	T	G	0.410	0.042	3.4e-22	1.3e-04	94.0
Blood Pressure	SBP	rs12050260	14	23761094	T	C	0.228	0.032	1.7e-12	6.7e-05	49.6
Blood Pressure	SBP	rs365990	14	23861811	A	G	0.225	0.031	6.0e-13	7.0e-05	52.0
Blood Pressure	SBP	rs17115145	14	30122409	T	C	0.178	0.031	7.4e-09	4.5e-05	33.4
Blood Pressure	SBP	rs2273171	14	31381351	T	C	-0.167	0.030	2.6e-08	4.1e-05	30.9
Blood Pressure	SBP	rs8904	14	35871217	A	G	0.306	0.031	1.7e-22	1.3e-04	95.0
Blood Pressure	SBP	rs2415299	14	36052290	A	G	-0.300	0.047	2.0e-10	5.7e-05	40.4
Blood Pressure	SBP	rs34983854	14	39858442	A	G	-0.206	0.031	2.1e-11	6.0e-05	44.9
Blood Pressure	SBP	rs72683923	14	50735947	T	C	0.959	0.110	3.1e-18	1.0e-04	75.8
Blood Pressure	SBP	rs74707969	14	53266966	A	G	0.300	0.051	3.0e-09	4.7e-05	35.2
Blood Pressure	SBP	rs35413927	14	53420358	A	G	-0.300	0.033	5.2e-20	1.1e-04	83.8
Blood Pressure	SBP	rs57140819	14	68018247	C	G	0.242	0.040	1.3e-09	4.9e-05	36.8
Blood Pressure	SBP	rs7146217	14	69250915	T	C	0.175	0.030	5.6e-09	4.5e-05	33.9
Blood Pressure	SBP	rs11847049	14	69259406	C	G	-0.227	0.036	4.4e-10	5.2e-05	39.0
Blood Pressure	SBP	rs227426	14	70456664	T	G	0.186	0.030	8.6e-10	5.1e-05	37.7
Blood Pressure	SBP	rs221902	14	71601079	T	G	0.172	0.032	4.8e-08	4.0e-05	29.7
Blood Pressure	SBP	rs11623535	14	72462381	A	G	0.210	0.034	1.0e-09	5.0e-05	37.3
Blood Pressure	SBP	rs3815460	14	73422259	C	G	-0.285	0.050	1.2e-08	4.4e-05	32.5
Blood Pressure	SBP	rs11159091	14	75074316	A	G	0.198	0.030	6.8e-11	5.8e-05	42.6
Blood Pressure	SBP	rs7156903	14	94466910	T	G	-0.194	0.031	5.4e-10	5.2e-05	38.6
Blood Pressure	SBP	rs7154723	14	98590629	A	G	0.253	0.031	2.7e-16	9.0e-05	67.0
Blood Pressure	SBP	rs12050196	14	100139434	T	C	0.213	0.031	6.3e-12	6.3e-05	47.1
Blood Pressure	SBP	rs8014182	14	103859962	T	C	-0.334	0.044	5.2e-14	7.6e-05	56.6
Blood Pressure	SBP	rs11629850	15	40317075	A	G	0.230	0.030	2.3e-14	7.8e-05	58.2
Blood Pressure	SBP	rs28866311	15	41442195	T	G	-0.276	0.030	5.5e-20	1.1e-04	83.6
Blood Pressure	SBP	rs4924570	15	41974660	T	C	-0.211	0.031	1.8e-11	6.1e-05	45.2
Blood Pressure	SBP	rs4775769	15	48939888	T	G	-0.416	0.052	7.8e-16	8.7e-05	64.8
Blood Pressure	SBP	rs3098186	15	50810621	T	C	-0.242	0.030	1.4e-15	8.6e-05	63.9
Blood Pressure	SBP	rs17730281	15	53907948	A	G	-0.200	0.036	2.4e-08	4.2e-05	31.2

Phenotype	Trait	SNP	Chr	Position (GRCh37/hg19)	EA	NEA	BETA	SE	P-value	R2	F
Blood Pressure	SBP	rs2652812	15	63406170	T	C	-0.252	0.035	1.0e-12	6.8e-05	50.8
Blood Pressure	SBP	rs7165200	15	66937421	A	G	0.201	0.030	2.9e-11	6.0e-05	44.3
Blood Pressure	SBP	rs11636952	15	75114322	T	C	0.531	0.033	4.2e-59	3.6e-04	262.4
Blood Pressure	SBP	rs72730585	15	75353906	A	G	-0.246	0.033	5.2e-14	7.6e-05	56.6
Blood Pressure	SBP	rs2627316	15	81042816	A	G	-0.333	0.030	2.8e-28	1.6e-04	121.4
Blood Pressure	SBP	rs7496012	15	85599660	A	G	-0.202	0.030	2.0e-11	6.0e-05	45.0
Blood Pressure	SBP	rs11632436	15	86295286	C	G	0.222	0.030	2.0e-13	7.4e-05	54.0
Blood Pressure	SBP	rs78550103	15	90027296	A	G	-0.284	0.043	4.5e-11	5.9e-05	43.5
Blood Pressure	SBP	rs28611491	15	90641809	T	C	0.334	0.058	6.5e-09	4.5e-05	33.7
Blood Pressure	SBP	rs4932373	15	91429287	A	C	-0.635	0.033	2.5e-83	5.2e-04	374.8
Blood Pressure	SBP	rs12904445	15	91480010	A	C	-0.374	0.053	1.9e-12	6.7e-05	49.6
Blood Pressure	SBP	rs11074093	15	93222897	T	C	-0.192	0.031	4.6e-10	5.2e-05	38.8
Blood Pressure	SBP	rs12906962	15	95312071	T	C	-0.265	0.032	3.3e-16	9.0e-05	66.6
Blood Pressure	SBP	rs2246754	15	96767312	T	C	0.250	0.037	1.2e-11	6.2e-05	46.1
Blood Pressure	SBP	rs12901664	15	98338524	T	C	0.182	0.032	9.6e-09	4.4e-05	32.9
Blood Pressure	SBP	rs4965529	15	100145224	A	C	-0.273	0.040	1.3e-11	6.2e-05	45.8
Blood Pressure	SBP	rs6600135	16	1362199	A	G	-0.273	0.046	3.1e-09	4.7e-05	35.1
Blood Pressure	SBP	rs28590346	16	2080653	A	T	-0.271	0.033	3.4e-16	9.2e-05	66.6
Blood Pressure	SBP	rs2379829	16	3538873	C	G	-0.268	0.034	4.5e-15	8.3e-05	61.3
Blood Pressure	SBP	rs74551774	16	4038528	A	G	0.337	0.059	1.0e-08	4.4e-05	32.8
Blood Pressure	SBP	rs7203473	16	4140231	C	G	-0.327	0.049	2.2e-11	6.0e-05	44.7
Blood Pressure	SBP	rs75345455	16	4419854	T	C	-0.494	0.074	3.3e-11	6.3e-05	44.0
Blood Pressure	SBP	rs13333419	16	4878707	A	G	-0.333	0.054	5.5e-10	5.2e-05	38.6
Blood Pressure	SBP	rs12446456	16	4922201	T	C	-0.300	0.030	3.0e-23	1.3e-04	98.9
Blood Pressure	SBP	rs7198817	16	11945778	A	C	-0.182	0.031	6.0e-09	4.6e-05	34.0
Blood Pressure	SBP	rs3915499	16	15910743	A	G	-0.206	0.032	1.4e-10	5.5e-05	41.1
Blood Pressure	SBP	rs77924615	16	20392332	A	G	-0.408	0.039	1.1e-25	1.5e-04	109.5
Blood Pressure	SBP	rs7186298	16	21088031	T	C	-0.232	0.030	1.9e-14	7.9e-05	58.8
Blood Pressure	SBP	rs6497759	16	24801737	A	G	-0.327	0.038	8.8e-18	1.0e-04	73.8
Blood Pressure	SBP	rs6565174	16	30111904	A	C	-0.288	0.048	2.6e-09	4.8e-05	35.5
Blood Pressure	SBP	rs10468291	16	49768046	A	C	-0.187	0.030	9.4e-10	5.1e-05	37.5
Blood Pressure	SBP	rs34941092	16	50550137	A	G	-0.322	0.043	3.2e-14	7.7e-05	57.6
Blood Pressure	SBP	rs12596721	16	51539762	A	T	-0.352	0.056	3.4e-10	5.3e-05	39.4
Blood Pressure	SBP	rs34869093	16	51757242	A	G	-0.220	0.031	1.8e-12	6.6e-05	49.6
Blood Pressure	SBP	rs2060664	16	60652439	T	C	0.216	0.034	4.1e-10	5.3e-05	39.2
Blood Pressure	SBP	rs4496141	16	65270392	T	G	0.180	0.030	2.3e-09	4.8e-05	35.6
Blood Pressure	SBP	rs3843712	16	66934153	T	C	-0.340	0.051	1.9e-11	6.2e-05	45.1
Blood Pressure	SBP	rs12149704	16	69789516	A	G	0.588	0.068	8.6e-18	9.9e-05	73.8
Blood Pressure	SBP	rs62053102	16	71654365	A	T	-0.569	0.075	3.4e-14	7.8e-05	57.4
Blood Pressure	SBP	rs62051555	16	72830539	C	G	-0.484	0.073	2.6e-11	6.0e-05	44.4
Blood Pressure	SBP	rs62055084	16	73097845	T	C	0.297	0.037	5.9e-16	9.0e-05	65.3
Blood Pressure	SBP	rs1012089	16	74171973	C	G	-0.192	0.030	1.9e-10	5.4e-05	40.4
Blood Pressure	SBP	rs4888408	16	75432824	A	G	0.365	0.031	1.4e-32	1.9e-04	141.6
Blood Pressure	SBP	rs56844452	16	80864776	T	C	-0.451	0.060	4.0e-14	7.7e-05	57.2
Blood Pressure	SBP	rs10493891	16	81510742	T	C	-0.277	0.034	1.3e-16	9.2e-05	68.4
Blood Pressure	SBP	rs4889341	16	81604251	T	C	-0.209	0.031	1.0e-11	6.2e-05	46.2
Blood Pressure	SBP	rs7500448	16	83045790	A	G	0.223	0.035	2.0e-10	5.5e-05	40.5
Blood Pressure	SBP	rs7187540	16	85318302	A	C	-0.195	0.034	1.0e-08	4.4e-05	32.7
Blood Pressure	SBP	rs3851018	16	86437811	C	G	0.192	0.031	5.4e-10	5.2e-05	38.5
Blood Pressure	SBP	rs6540125	16	87993889	T	G	0.204	0.032	1.2e-10	5.6e-05	41.5
Blood Pressure	SBP	rs908951	16	89697625	T	C	-0.226	0.032	7.1e-13	7.0e-05	51.5
Blood Pressure	SBP	rs11650511	17	1337960	T	C	0.243	0.031	1.1e-14	8.2e-05	59.9
Blood Pressure	SBP	rs4480845	17	1958609	T	C	0.316	0.032	1.8e-23	1.3e-04	99.7
Blood Pressure	SBP	rs930526	17	6473353	A	C	0.221	0.031	4.9e-13	7.0e-05	52.3
Blood Pressure	SBP	rs113086489	17	7171356	T	C	0.325	0.031	3.8e-26	1.5e-04	112.0
Blood Pressure	SBP	rs9899183	17	7452977	T	C	-0.289	0.034	1.9e-17	9.7e-05	72.4

Phenotype	Trait	SNP	Chr	Position (GRCh37/hg19)	EA	NEA	BETA	SE	P-value	R2	F
Blood Pressure	SBP	rs62059712	17	7740170	T	C	0.494	0.058	9.9e-18	9.9e-05	73.6
Blood Pressure	SBP	rs4925159	17	18185510	A	G	0.217	0.030	9.7e-13	6.9e-05	50.8
Blood Pressure	SBP	rs7218708	17	19926836	A	G	-0.178	0.030	4.4e-09	4.7e-05	34.5
Blood Pressure	SBP	rs704	17	26694861	A	G	-0.169	0.030	1.9e-08	4.3e-05	31.7
Blood Pressure	SBP	rs9899540	17	30777924	A	T	0.201	0.032	1.9e-10	5.4e-05	40.5
Blood Pressure	SBP	rs4793069	17	40801187	A	G	0.374	0.066	1.7e-08	4.3e-05	31.9
Blood Pressure	SBP	rs7502079	17	42713882	T	C	-0.391	0.051	2.2e-14	7.8e-05	58.3
Blood Pressure	SBP	rs7213273	17	43155914	A	G	-0.400	0.032	6.2e-37	2.2e-04	161.2
Blood Pressure	SBP	rs56319902	17	43871982	T	C	-0.261	0.038	5.5e-12	6.6e-05	47.5
Blood Pressure	SBP	rs17608766	17	45013271	T	C	-0.690	0.043	2.5e-57	3.4e-04	254.2
Blood Pressure	SBP	rs118127175	17	46188183	T	G	0.725	0.080	7.7e-20	1.1e-04	83.1
Blood Pressure	SBP	rs72827810	17	46355550	A	G	0.434	0.050	7.5e-18	9.9e-05	74.1
Blood Pressure	SBP	rs7209108	17	46863898	T	C	-0.207	0.034	1.8e-09	4.9e-05	36.2
Blood Pressure	SBP	rs12944684	17	47509486	C	G	0.310	0.032	5.2e-22	1.3e-04	93.2
Blood Pressure	SBP	rs2611774	17	56732304	A	G	0.219	0.032	6.8e-12	6.4e-05	47.0
Blood Pressure	SBP	rs78638044	17	57974476	A	G	0.280	0.049	1.2e-08	4.4e-05	32.5
Blood Pressure	SBP	rs11079404	17	59025721	A	G	0.247	0.040	9.7e-10	5.1e-05	37.4
Blood Pressure	SBP	rs1000423	17	59475642	T	C	0.414	0.035	6.5e-33	1.9e-04	143.0
Blood Pressure	SBP	rs56288724	17	60767135	A	G	-0.218	0.031	2.0e-12	6.6e-05	49.4
Blood Pressure	SBP	rs8077276	17	61547562	A	G	-0.295	0.031	5.1e-21	1.2e-04	88.2
Blood Pressure	SBP	rs6504213	17	62381714	T	C	-0.298	0.031	1.2e-21	1.2e-04	91.3
Blood Pressure	SBP	rs1991401	17	62502435	A	G	0.251	0.033	2.9e-14	7.9e-05	57.7
Blood Pressure	SBP	rs112260610	17	64252393	T	C	0.259	0.044	2.7e-09	4.8e-05	35.4
Blood Pressure	SBP	rs3744009	17	73839366	T	C	-0.246	0.035	1.6e-12	6.7e-05	49.8
Blood Pressure	SBP	rs1436138	17	75316880	A	G	0.312	0.032	4.7e-23	1.3e-04	98.0
Blood Pressure	SBP	rs9302885	17	76799898	A	G	0.224	0.030	1.0e-13	7.4e-05	55.1
Blood Pressure	SBP	rs11655604	17	79365861	T	C	-0.203	0.033	1.1e-09	5.3e-05	37.3
Blood Pressure	SBP	rs34413141	18	777282	A	T	-0.353	0.039	2.5e-19	1.1e-04	80.7
Blood Pressure	SBP	rs62082230	18	22676071	A	T	-0.188	0.034	4.7e-08	4.0e-05	29.8
Blood Pressure	SBP	rs1154214	18	24546824	T	G	-0.203	0.031	3.3e-11	5.9e-05	44.1
Blood Pressure	SBP	rs56407827	18	42179819	T	C	0.360	0.034	2.8e-26	1.5e-04	112.3
Blood Pressure	SBP	rs11874246	18	42596789	T	C	0.286	0.033	3.2e-18	1.0e-04	75.8
Blood Pressure	SBP	rs16978387	18	43083393	A	T	-0.227	0.035	5.4e-11	5.8e-05	43.0
Blood Pressure	SBP	rs61148001	18	48133241	T	C	-0.258	0.037	2.1e-12	6.6e-05	49.5
Blood Pressure	SBP	rs11872627	18	48287818	T	C	-0.295	0.044	1.3e-11	6.1e-05	45.7
Blood Pressure	SBP	rs72915163	18	48792829	T	C	0.202	0.035	1.1e-08	4.4e-05	32.7
Blood Pressure	SBP	rs1657880	18	51866974	T	C	-0.287	0.050	7.4e-09	4.5e-05	33.4
Blood Pressure	SBP	rs613872	18	53210302	T	G	-0.224	0.040	1.7e-08	4.3e-05	31.9
Blood Pressure	SBP	rs10048404	18	54578482	T	C	-0.261	0.032	1.9e-16	9.1e-05	67.6
Blood Pressure	SBP	rs6567160	18	57829135	T	C	0.224	0.036	3.3e-10	5.3e-05	39.4
Blood Pressure	SBP	rs12454712	18	60845884	T	C	0.191	0.033	5.8e-09	4.8e-05	33.8
Blood Pressure	SBP	rs10460108	18	73034151	A	G	0.214	0.030	1.1e-12	6.8e-05	50.6
Blood Pressure	SBP	rs2289286	19	1822668	T	C	0.224	0.038	2.4e-09	4.9e-05	35.6
Blood Pressure	SBP	rs55678414	19	2177625	T	G	-0.689	0.063	1.6e-27	1.6e-04	118.1
Blood Pressure	SBP	rs1982468	19	4953351	T	G	0.174	0.032	3.9e-08	4.0e-05	30.1
Blood Pressure	SBP	rs2613765	19	5066330	A	G	-0.235	0.030	5.3e-15	8.2e-05	61.1
Blood Pressure	SBP	rs12971628	19	7230675	T	G	-0.254	0.032	4.2e-15	8.3e-05	61.6
Blood Pressure	SBP	rs12978472	19	7257990	C	G	0.845	0.049	1.2e-66	4.1e-04	297.4
Blood Pressure	SBP	rs56718386	19	7296283	T	C	0.327	0.051	1.0e-10	5.8e-05	41.7
Blood Pressure	SBP	rs72999517	19	7522549	A	C	0.256	0.035	2.1e-13	7.2e-05	53.9
Blood Pressure	SBP	rs10409243	19	10332988	T	C	-0.308	0.031	8.1e-23	1.3e-04	96.6
Blood Pressure	SBP	rs34333186	19	11284891	A	T	0.404	0.057	1.4e-12	6.8e-05	50.2
Blood Pressure	SBP	rs118081085	19	11419844	T	C	0.485	0.080	1.2e-09	5.1e-05	36.9
Blood Pressure	SBP	rs167479	19	11526765	T	G	-0.564	0.033	7.2e-67	4.4e-04	297.7
Blood Pressure	SBP	rs7246865	19	17219105	A	G	0.276	0.034	1.0e-15	8.6e-05	64.5
Blood Pressure	SBP	rs34518929	19	18455444	A	G	-0.220	0.034	1.8e-10	5.5e-05	40.7

Phenotype	Trait	SNP	Chr	Position (GRCh37/hg19)	EA	NEA	BETA	SE	P-value	R2	F
Blood Pressure	SBP	rs143333049	19	19800289	A	G	-0.542	0.087	4.1e-10	5.4e-05	39.0
Blood Pressure	SBP	rs4319878	19	21924452	T	C	0.169	0.031	3.8e-08	4.1e-05	30.2
Blood Pressure	SBP	rs1848994	19	22111366	A	G	0.201	0.033	1.8e-09	4.9e-05	36.3
Blood Pressure	SBP	rs3786516	19	30100319	A	T	0.231	0.036	2.1e-10	5.5e-05	40.4
Blood Pressure	SBP	rs60138042	19	30350005	C	G	0.340	0.062	4.1e-08	4.1e-05	30.1
Blood Pressure	SBP	rs28572357	19	31867447	A	C	-0.273	0.031	6.3e-19	1.1e-04	78.7
Blood Pressure	SBP	rs10421783	19	32457846	A	C	0.194	0.032	1.4e-09	4.9e-05	36.6
Blood Pressure	SBP	rs7256564	19	33889593	A	G	0.196	0.032	1.5e-09	4.9e-05	36.4
Blood Pressure	SBP	rs256741	19	37855839	A	C	0.168	0.031	4.5e-08	4.0e-05	30.0
Blood Pressure	SBP	rs7412	19	45412079	T	C	-0.434	0.057	4.0e-14	7.9e-05	57.1
Blood Pressure	SBP	rs7255933	19	45766729	A	G	0.231	0.034	2.4e-11	6.0e-05	44.7
Blood Pressure	SBP	rs7250497	19	46358425	A	G	0.205	0.032	1.2e-10	5.6e-05	41.4
Blood Pressure	SBP	rs8103840	19	49254955	T	C	-0.210	0.031	1.8e-11	6.2e-05	45.3
Blood Pressure	SBP	rs73046792	19	49605705	A	G	-0.355	0.043	7.2e-17	9.4e-05	69.6
Blood Pressure	SBP	rs1764975	20	4101290	A	T	0.282	0.038	1.1e-13	7.4e-05	55.3
Blood Pressure	SBP	rs6054139	20	6327810	A	G	0.209	0.031	8.2e-12	6.3e-05	46.8
Blood Pressure	SBP	rs6039211	20	8616588	A	G	0.283	0.031	1.9e-19	1.1e-04	81.5
Blood Pressure	SBP	rs6040076	20	10658882	C	G	-0.323	0.031	1.0e-25	1.5e-04	110.0
Blood Pressure	SBP	rs974266	20	10882552	A	C	0.268	0.030	7.7e-19	1.1e-04	78.7
Blood Pressure	SBP	rs6108787	20	10967214	T	G	-0.427	0.030	5.4e-46	2.7e-04	203.0
Blood Pressure	SBP	rs8118496	20	10996231	A	G	-0.196	0.033	2.3e-09	4.8e-05	35.7
Blood Pressure	SBP	rs78057287	20	11023553	A	C	-0.959	0.149	1.3e-10	5.6e-05	41.3
Blood Pressure	SBP	rs6078084	20	11151237	A	G	0.173	0.030	1.5e-08	4.3e-05	32.1
Blood Pressure	SBP	rs1998107	20	17877073	A	G	-0.190	0.030	2.2e-10	5.4e-05	40.3
Blood Pressure	SBP	rs17812022	20	19007099	T	C	-0.361	0.052	5.6e-12	6.4e-05	47.4
Blood Pressure	SBP	rs3790227	20	19469002	A	C	0.187	0.033	2.1e-08	4.2e-05	31.5
Blood Pressure	SBP	rs6137201	20	20957669	A	T	0.267	0.047	1.6e-08	4.3e-05	31.9
Blood Pressure	SBP	rs6058088	20	30139886	T	G	0.283	0.042	1.1e-11	6.2e-05	46.1
Blood Pressure	SBP	rs6141766	20	31222769	A	G	-0.314	0.042	9.9e-14	7.4e-05	55.5
Blood Pressure	SBP	rs4812536	20	40258709	A	G	0.271	0.032	4.1e-17	9.5e-05	70.6
Blood Pressure	SBP	rs58442973	20	42770972	A	G	0.332	0.046	4.8e-13	7.0e-05	52.2
Blood Pressure	SBP	rs6031431	20	42795152	A	G	-0.262	0.030	7.0e-18	1.0e-04	74.1
Blood Pressure	SBP	rs6012506	20	47285637	A	G	0.173	0.030	1.1e-08	4.4e-05	32.6
Blood Pressure	SBP	rs6090907	20	47410231	A	G	-0.385	0.043	1.3e-19	1.1e-04	82.2
Blood Pressure	SBP	rs6021247	20	50108980	A	G	0.233	0.030	9.8e-15	8.1e-05	59.8
Blood Pressure	SBP	rs2801008	20	51788718	T	G	-0.188	0.032	7.4e-09	4.5e-05	33.5
Blood Pressure	SBP	rs749209	20	57088189	T	C	-0.171	0.031	3.4e-08	4.1e-05	30.5
Blood Pressure	SBP	rs234615	20	57498784	C	G	-0.214	0.033	1.6e-10	5.5e-05	40.9
Blood Pressure	SBP	rs163780	20	57561525	C	G	0.235	0.031	5.5e-14	7.6e-05	56.6
Blood Pressure	SBP	rs6026744	20	57742388	A	T	-0.713	0.046	7.0e-54	3.2e-04	239.3
Blood Pressure	SBP	rs28374392	20	61189717	T	C	0.192	0.034	1.2e-08	4.8e-05	32.4
Blood Pressure	SBP	rs6062324	20	62446351	A	G	-0.329	0.036	1.2e-19	1.1e-04	82.3
Blood Pressure	SBP	rs117267354	20	62583838	A	G	0.221	0.039	1.5e-08	4.4e-05	32.0
Blood Pressure	SBP	rs35213536	20	62694319	T	G	0.294	0.036	1.9e-16	9.3e-05	67.7
Blood Pressure	SBP	rs2254107	21	16324417	A	G	-0.199	0.032	2.6e-10	5.4e-05	40.1
Blood Pressure	SBP	rs1882961	21	16556367	T	C	0.244	0.033	6.7e-14	7.5e-05	56.2
Blood Pressure	SBP	rs2823263	21	16790213	T	C	-0.184	0.033	2.7e-08	4.2e-05	30.9
Blood Pressure	SBP	rs2833834	21	33814378	A	C	0.218	0.034	1.2e-10	5.6e-05	41.5
Blood Pressure	SBP	rs149487184	21	35605863	T	C	-0.634	0.105	1.5e-09	4.9e-05	36.6
Blood Pressure	SBP	rs12627651	21	44760603	A	G	0.350	0.034	1.0e-24	1.4e-04	105.2
Blood Pressure	SBP	rs4818861	21	45123807	A	G	-0.227	0.031	3.0e-13	7.1e-05	53.2
Blood Pressure	SBP	rs2238787	22	19976406	A	G	0.255	0.033	1.5e-14	7.9e-05	59.1
Blood Pressure	SBP	rs12321	22	29453193	C	G	-0.229	0.030	3.8e-14	7.7e-05	57.2
Blood Pressure	SBP	rs12627866	22	30202984	T	C	-0.418	0.069	1.7e-09	4.9e-05	36.3
Blood Pressure	SBP	rs8142376	22	32001037	T	C	0.168	0.030	2.2e-08	4.2e-05	31.2
Blood Pressure	SBP	rs28578714	22	50727921	T	C	0.207	0.033	2.5e-10	5.6e-05	39.9

Phenotype	Trait	SNP	Chr	Position (GRCh37/hg19)	EA	NEA	BETA	SE	P-value	R2	F
Blood Pressure	DBP	rs34645159	1	1724366	A	G	-0.133	0.017	2.1e-14	7.7e-05	58.4
Blood Pressure	DBP	rs2493288	1	3330884	A	G	0.254	0.025	2.0e-23	1.3e-04	99.7
Blood Pressure	DBP	rs4908670	1	7722446	A	T	-0.108	0.018	1.2e-09	4.9e-05	36.8
Blood Pressure	DBP	rs2252865	1	8422676	T	C	0.119	0.018	5.5e-11	5.8e-05	43.2
Blood Pressure	DBP	rs17035646	1	10796547	A	G	0.260	0.018	1.3e-44	2.6e-04	197.1
Blood Pressure	DBP	rs55857306	1	11895795	A	G	-0.522	0.024	5.1e-109	6.5e-04	494.2
Blood Pressure	DBP	rs7516453	1	15972558	C	G	0.124	0.020	1.8e-10	5.3e-05	40.4
Blood Pressure	DBP	rs848309	1	16308447	T	C	-0.138	0.017	2.8e-15	8.3e-05	62.6
Blood Pressure	DBP	rs6686889	1	25030470	T	C	0.192	0.020	6.9e-22	1.2e-04	92.9
Blood Pressure	DBP	rs311427	1	25358706	A	G	0.131	0.019	1.0e-11	6.1e-05	46.5
Blood Pressure	DBP	rs12728150	1	27268737	A	G	-0.204	0.032	1.3e-10	5.5e-05	41.4
Blood Pressure	DBP	rs1565716	1	29549216	A	G	0.214	0.034	3.5e-10	5.2e-05	39.3
Blood Pressure	DBP	rs61776678	1	38377021	A	G	-0.100	0.018	2.7e-08	4.1e-05	31.1
Blood Pressure	DBP	rs72663521	1	40005903	A	G	0.126	0.022	1.4e-08	4.3e-05	32.3
Blood Pressure	DBP	rs1138293	1	41486245	T	C	-0.128	0.022	7.1e-09	4.4e-05	33.6
Blood Pressure	DBP	rs34538877	1	42383670	T	C	-0.124	0.018	2.1e-12	6.6e-05	49.2
Blood Pressure	DBP	rs710249	1	43869235	C	G	0.150	0.017	6.4e-18	9.8e-05	74.4
Blood Pressure	DBP	rs56810307	1	44973546	T	C	-0.155	0.027	1.3e-08	4.3e-05	32.2
Blood Pressure	DBP	rs12142296	1	46541679	T	G	-0.164	0.025	8.9e-11	5.6e-05	42.2
Blood Pressure	DBP	rs12143164	1	47974381	T	C	-0.100	0.018	4.6e-08	4.0e-05	30.0
Blood Pressure	DBP	rs4926923	1	48109225	T	C	0.192	0.031	4.7e-10	5.2e-05	38.8
Blood Pressure	DBP	rs116287796	1	51457442	T	C	-0.423	0.066	1.0e-10	5.5e-05	41.8
Blood Pressure	DBP	rs61772592	1	56979681	A	G	-0.151	0.026	7.4e-09	4.4e-05	33.4
Blood Pressure	DBP	rs12730750	1	59613905	A	G	0.112	0.019	1.9e-09	4.8e-05	35.9
Blood Pressure	DBP	rs10493408	1	66992054	A	C	0.158	0.025	5.1e-10	5.1e-05	38.6
Blood Pressure	DBP	rs34517439	1	78450517	A	C	-0.251	0.028	2.0e-19	1.1e-04	81.2
Blood Pressure	DBP	rs786921	1	89286673	A	G	-0.114	0.018	8.6e-11	5.7e-05	42.3
Blood Pressure	DBP	rs17396055	1	94730954	A	G	-0.115	0.018	4.1e-10	5.2e-05	39.1
Blood Pressure	DBP	rs10776752	1	113044328	T	G	0.457	0.033	1.2e-43	2.5e-04	192.0
Blood Pressure	DBP	rs11466111	1	115829178	T	C	0.668	0.067	1.9e-23	1.3e-04	99.4
Blood Pressure	DBP	rs568032	1	115976048	A	G	-0.267	0.039	6.3e-12	6.3e-05	47.1
Blood Pressure	DBP	rs1886914	1	119541452	C	G	-0.111	0.018	3.0e-10	5.2e-05	39.5
Blood Pressure	DBP	rs72704264	1	145713305	C	G	0.117	0.021	3.6e-08	4.0e-05	30.5
Blood Pressure	DBP	rs1819663	1	154025891	A	G	0.115	0.017	4.6e-11	5.8e-05	43.5
Blood Pressure	DBP	rs11578696	1	156056968	A	G	0.146	0.026	1.6e-08	4.2e-05	32.0
Blood Pressure	DBP	rs2171690	1	164740099	T	C	0.118	0.017	1.2e-11	6.1e-05	46.1
Blood Pressure	DBP	rs7524019	1	167367193	T	C	0.104	0.017	2.6e-09	4.7e-05	35.5
Blood Pressure	DBP	rs12405515	1	172357441	T	G	-0.170	0.017	1.9e-22	1.3e-04	95.2
Blood Pressure	DBP	rs3766694	1	175143207	T	C	0.110	0.018	4.1e-10	5.2e-05	39.3
Blood Pressure	DBP	rs3795320	1	176591291	T	G	-0.118	0.020	4.4e-09	4.5e-05	34.3
Blood Pressure	DBP	rs150816167	1	179571862	T	C	-0.287	0.045	1.2e-10	5.5e-05	41.5
Blood Pressure	DBP	rs3789368	1	180842196	T	C	-0.114	0.018	1.3e-10	5.4e-05	41.2
Blood Pressure	DBP	rs41475048	1	183058452	A	G	-0.123	0.020	9.9e-10	4.9e-05	37.3
Blood Pressure	DBP	rs4651224	1	184585182	T	C	0.110	0.018	3.4e-10	5.2e-05	39.7
Blood Pressure	DBP	rs882624	1	201735913	T	C	-0.157	0.018	2.3e-17	9.6e-05	72.1
Blood Pressure	DBP	rs33996239	1	203109801	T	C	-0.251	0.038	2.9e-11	5.8e-05	44.2
Blood Pressure	DBP	rs6700051	1	204071890	C	G	-0.110	0.019	3.4e-09	4.6e-05	34.8
Blood Pressure	DBP	rs2169137	1	204497913	C	G	0.159	0.019	3.2e-16	8.8e-05	67.0
Blood Pressure	DBP	rs2629665	1	207220800	A	C	-0.119	0.018	1.5e-11	6.1e-05	45.4
Blood Pressure	DBP	rs1502358	1	217324932	A	G	-0.113	0.018	1.1e-09	4.9e-05	37.1
Blood Pressure	DBP	rs68085857	1	217737629	T	C	0.191	0.020	9.8e-21	1.1e-04	86.8
Blood Pressure	DBP	rs35981664	1	218549354	A	T	-0.161	0.019	2.0e-17	9.5e-05	72.2
Blood Pressure	DBP	rs9431431	1	221358796	A	G	-0.134	0.019	1.7e-12	6.6e-05	49.7
Blood Pressure	DBP	rs2670441	1	227434681	T	C	-0.137	0.020	1.1e-11	6.1e-05	46.1
Blood Pressure	DBP	rs2760061	1	228191075	A	T	0.177	0.018	1.0e-23	1.3e-04	101.3
Blood Pressure	DBP	rs699	1	230845794	A	G	-0.236	0.018	1.3e-40	2.4e-04	177.6

Phenotype	Trait	SNP	Chr	Position (GRCh37/hg19)	EA	NEA	BETA	SE	P-value	R2	F
Blood Pressure	DBP	rs1885018	1	236316575	A	C	0.132	0.023	1.8e-08	4.2e-05	31.7
Blood Pressure	DBP	rs3943093	1	243458502	T	C	0.248	0.018	3.9e-41	2.4e-04	181.2
Blood Pressure	DBP	rs4926499	1	249155909	C	G	0.169	0.025	9.4e-12	6.4e-05	46.7
Blood Pressure	DBP	rs112393817	2	9807226	C	G	0.116	0.021	3.8e-08	4.0e-05	30.2
Blood Pressure	DBP	rs1373780	2	19501029	C	G	0.125	0.022	2.6e-08	4.1e-05	30.9
Blood Pressure	DBP	rs824522	2	19707726	A	G	0.169	0.021	9.5e-16	8.5e-05	64.5
Blood Pressure	DBP	rs342130	2	20715656	A	G	0.110	0.018	6.2e-10	5.1e-05	38.4
Blood Pressure	DBP	rs2384061	2	25135620	A	G	-0.174	0.018	2.3e-23	1.3e-04	99.0
Blood Pressure	DBP	rs1275988	2	26914364	T	C	-0.294	0.018	1.9e-62	3.7e-04	276.8
Blood Pressure	DBP	rs6547850	2	28629343	T	G	-0.111	0.017	2.1e-10	5.4e-05	40.5
Blood Pressure	DBP	rs57361399	2	37528311	T	C	-0.165	0.021	1.2e-15	8.5e-05	64.2
Blood Pressure	DBP	rs3731851	2	37880919	A	T	-0.134	0.022	2.1e-09	4.8e-05	35.9
Blood Pressure	DBP	rs12611608	2	40577162	C	G	-0.142	0.017	1.7e-16	9.0e-05	67.8
Blood Pressure	DBP	rs76326501	2	43167878	A	C	0.362	0.030	2.2e-32	1.9e-04	140.7
Blood Pressure	DBP	rs4952668	2	43386568	A	G	-0.192	0.018	1.1e-26	1.5e-04	113.8
Blood Pressure	DBP	rs12712893	2	43877550	A	G	0.108	0.018	1.8e-09	4.8e-05	36.1
Blood Pressure	DBP	rs10170954	2	44206799	A	C	-0.105	0.018	6.9e-09	4.4e-05	33.5
Blood Pressure	DBP	rs687914	2	45878760	T	G	0.169	0.020	6.5e-17	9.2e-05	69.8
Blood Pressure	DBP	rs10495928	2	46353166	A	G	0.125	0.018	7.7e-12	6.2e-05	47.0
Blood Pressure	DBP	rs1454393	2	53002225	A	C	-0.101	0.017	4.5e-09	4.5e-05	34.3
Blood Pressure	DBP	rs2586970	2	55829967	A	G	-0.149	0.018	1.6e-17	9.8e-05	72.8
Blood Pressure	DBP	rs72816333	2	60096560	A	T	0.143	0.023	4.7e-10	5.1e-05	38.8
Blood Pressure	DBP	rs7608483	2	61836235	A	C	0.117	0.018	2.8e-11	5.9e-05	44.3
Blood Pressure	DBP	rs13014371	2	64217786	T	C	-0.118	0.018	1.7e-11	6.0e-05	45.2
Blood Pressure	DBP	rs13026184	2	64930786	C	G	-0.113	0.020	2.3e-08	4.1e-05	31.3
Blood Pressure	DBP	rs12052761	2	69065841	A	G	-0.123	0.018	4.2e-12	6.4e-05	48.2
Blood Pressure	DBP	rs1876490	2	73052351	A	G	0.136	0.019	1.2e-12	6.7e-05	50.5
Blood Pressure	DBP	rs6546810	2	73389716	T	C	-0.120	0.018	3.2e-11	5.8e-05	44.0
Blood Pressure	DBP	rs311564	2	86293498	A	G	-0.133	0.018	4.2e-13	7.0e-05	52.8
Blood Pressure	DBP	rs62155750	2	96491456	A	G	-0.218	0.020	8.3e-29	1.6e-04	123.4
Blood Pressure	DBP	rs78381888	2	97500775	A	G	-0.198	0.024	9.7e-17	9.2e-05	69.0
Blood Pressure	DBP	rs35098301	2	98580012	T	C	-0.108	0.018	1.4e-09	4.8e-05	36.7
Blood Pressure	DBP	rs28377357	2	112769721	A	G	-0.124	0.019	6.0e-11	5.7e-05	42.8
Blood Pressure	DBP	rs62158170	2	114082175	A	G	0.164	0.021	6.6e-15	8.0e-05	60.8
Blood Pressure	DBP	rs10864859	2	121440218	T	G	0.196	0.032	1.5e-09	4.8e-05	36.4
Blood Pressure	DBP	rs13001283	2	127183454	A	G	0.152	0.024	1.9e-10	5.4e-05	40.6
Blood Pressure	DBP	rs4954192	2	135632981	T	C	-0.122	0.018	8.1e-12	6.3e-05	46.8
Blood Pressure	DBP	rs7606205	2	144146311	A	C	-0.128	0.019	2.4e-11	5.9e-05	44.6
Blood Pressure	DBP	rs55944332	2	145726621	A	G	-0.236	0.020	3.3e-31	1.8e-04	134.4
Blood Pressure	DBP	rs62169544	2	146950908	A	G	-0.121	0.018	5.0e-12	6.3e-05	47.6
Blood Pressure	DBP	rs12990959	2	148572160	T	C	-0.127	0.019	1.1e-11	6.1e-05	46.2
Blood Pressure	DBP	rs1220128	2	158499902	C	G	0.192	0.025	6.2e-15	8.0e-05	60.7
Blood Pressure	DBP	rs72936986	2	159429379	A	C	0.114	0.019	3.3e-09	4.7e-05	34.9
Blood Pressure	DBP	rs16848690	2	164456639	T	C	0.183	0.029	1.9e-10	5.3e-05	40.5
Blood Pressure	DBP	rs73029563	2	165008166	C	G	-0.259	0.017	5.8e-50	2.9e-04	221.9
Blood Pressure	DBP	rs34357955	2	165731664	A	G	0.102	0.018	9.8e-09	4.4e-05	33.0
Blood Pressure	DBP	rs6758859	2	173965056	T	C	0.121	0.018	1.5e-11	6.0e-05	45.8
Blood Pressure	DBP	rs12623637	2	177573878	A	G	-0.102	0.018	6.0e-09	4.5e-05	33.8
Blood Pressure	DBP	rs17362588	2	179721046	A	G	0.333	0.030	9.3e-28	1.6e-04	119.2
Blood Pressure	DBP	rs10184839	2	181946115	A	T	-0.140	0.019	2.1e-13	7.2e-05	54.1
Blood Pressure	DBP	rs12693302	2	183211443	A	G	-0.238	0.018	2.2e-39	2.3e-04	172.6
Blood Pressure	DBP	rs7576060	2	188073163	T	C	-0.102	0.018	2.1e-08	4.2e-05	31.6
Blood Pressure	DBP	rs7592578	2	191439591	T	G	-0.200	0.022	4.7e-19	1.1e-04	79.6
Blood Pressure	DBP	rs4675297	2	203696607	A	G	-0.128	0.018	3.2e-12	6.5e-05	48.7
Blood Pressure	DBP	rs2162003	2	205077128	T	C	0.128	0.018	3.2e-12	6.5e-05	48.8
Blood Pressure	DBP	rs1263671	2	207996447	T	C	-0.139	0.024	4.7e-09	4.5e-05	34.3

Phenotype	Trait	SNP	Chr	Position (GRCh37/hg19)	EA	NEA	BETA	SE	P-value	R2	F
Blood Pressure	DBP	rs4675682	2	208402750	T	C	-0.141	0.017	4.5e-16	8.8e-05	66.3
Blood Pressure	DBP	rs1047891	2	211540507	A	C	-0.141	0.019	8.2e-14	7.4e-05	55.9
Blood Pressure	DBP	rs1035673	2	218675533	T	C	0.162	0.018	3.0e-20	1.1e-04	85.2
Blood Pressure	DBP	rs1996992	2	219651349	T	G	-0.297	0.039	4.7e-14	7.5e-05	56.7
Blood Pressure	DBP	rs13002079	2	220339466	T	C	-0.106	0.018	8.4e-09	4.4e-05	33.2
Blood Pressure	DBP	rs10804330	2	227185749	T	C	0.133	0.018	4.6e-14	7.6e-05	57.2
Blood Pressure	DBP	rs1044822	2	230629138	T	C	-0.133	0.024	4.1e-08	4.0e-05	30.1
Blood Pressure	DBP	rs4507125	2	239864732	A	C	-0.124	0.021	3.6e-09	4.6e-05	34.8
Blood Pressure	DBP	rs347585	3	11286220	T	C	0.151	0.019	1.6e-15	8.4e-05	63.5
Blood Pressure	DBP	rs1687295	3	14889756	T	C	0.206	0.019	3.0e-26	1.5e-04	112.9
Blood Pressure	DBP	rs6442608	3	16588273	T	C	-0.134	0.023	7.5e-09	4.5e-05	33.3
Blood Pressure	DBP	rs4634143	3	23163749	T	C	0.116	0.019	7.9e-10	5.0e-05	37.7
Blood Pressure	DBP	rs2643826	3	27562988	T	C	0.186	0.018	2.8e-26	1.5e-04	112.6
Blood Pressure	DBP	rs72851229	3	29374219	C	G	-0.136	0.023	3.6e-09	4.6e-05	34.9
Blood Pressure	DBP	rs56401299	3	30422563	T	C	0.100	0.018	4.5e-08	4.0e-05	30.1
Blood Pressure	DBP	rs7427249	3	37572489	A	G	-0.110	0.018	4.3e-10	5.1e-05	38.9
Blood Pressure	DBP	rs4973930	3	41306063	A	C	-0.103	0.017	2.7e-09	4.7e-05	35.6
Blood Pressure	DBP	rs114714860	3	41882905	C	G	0.330	0.024	1.4e-44	2.6e-04	195.5
Blood Pressure	DBP	rs113134141	3	46861939	A	G	-0.161	0.029	2.5e-08	4.1e-05	31.2
Blood Pressure	DBP	rs6442105	3	48182326	A	G	-0.248	0.018	3.1e-41	2.4e-04	180.4
Blood Pressure	DBP	rs36022378	3	49913705	T	C	-0.176	0.022	8.6e-16	8.6e-05	65.0
Blood Pressure	DBP	rs6775938	3	51006237	T	C	0.146	0.026	1.4e-08	4.2e-05	32.2
Blood Pressure	DBP	rs35593046	3	53553923	T	G	-0.152	0.020	4.4e-14	7.6e-05	57.2
Blood Pressure	DBP	rs2680663	3	53735299	A	G	-0.178	0.018	7.3e-22	1.2e-04	92.5
Blood Pressure	DBP	rs3772219	3	56771251	A	C	0.175	0.018	2.9e-21	1.2e-04	89.9
Blood Pressure	DBP	rs17801943	3	57899366	T	C	-0.160	0.019	1.3e-17	9.7e-05	73.1
Blood Pressure	DBP	rs3774702	3	63856870	A	G	0.147	0.023	1.2e-10	5.5e-05	41.6
Blood Pressure	DBP	rs9870517	3	64708600	A	C	0.145	0.018	1.9e-16	9.0e-05	67.5
Blood Pressure	DBP	rs4499560	3	70920485	A	T	-0.114	0.019	1.1e-09	4.9e-05	37.2
Blood Pressure	DBP	rs7623706	3	74712754	A	G	0.098	0.018	2.8e-08	4.1e-05	30.7
Blood Pressure	DBP	rs11923343	3	85668570	A	G	-0.114	0.018	3.1e-10	5.2e-05	39.5
Blood Pressure	DBP	rs11923667	3	101268080	A	T	0.117	0.018	3.1e-11	5.8e-05	44.1
Blood Pressure	DBP	rs28675079	3	111500002	A	G	-0.144	0.022	8.3e-11	5.6e-05	42.3
Blood Pressure	DBP	rs9834975	3	122117663	A	T	-0.105	0.017	1.4e-09	4.8e-05	36.5
Blood Pressure	DBP	rs4141663	3	124551967	T	C	-0.150	0.018	1.4e-17	9.7e-05	73.1
Blood Pressure	DBP	rs62264113	3	127292333	A	G	0.153	0.028	4.7e-08	3.9e-05	29.9
Blood Pressure	DBP	rs4077158	3	133942941	T	C	-0.183	0.017	3.1e-26	1.5e-04	112.1
Blood Pressure	DBP	rs6807945	3	138052754	T	C	-0.187	0.024	1.8e-15	8.3e-05	63.1
Blood Pressure	DBP	rs74734425	3	141096216	T	C	0.404	0.042	3.5e-22	1.2e-04	93.8
Blood Pressure	DBP	rs9844972	3	150097635	C	G	0.225	0.035	8.0e-11	5.6e-05	42.4
Blood Pressure	DBP	rs35288962	3	153732022	A	G	0.148	0.020	7.7e-14	7.4e-05	55.8
Blood Pressure	DBP	rs78809139	3	154674943	A	G	-0.228	0.029	2.6e-15	8.3e-05	62.7
Blood Pressure	DBP	rs78151625	3	158316726	T	C	-0.187	0.023	1.0e-15	8.5e-05	64.3
Blood Pressure	DBP	rs16853198	3	168840179	A	G	0.339	0.033	4.4e-25	1.4e-04	107.2
Blood Pressure	DBP	rs1528293	3	169154511	A	T	0.276	0.017	1.5e-57	3.4e-04	255.3
Blood Pressure	DBP	rs62294352	3	169197122	T	C	-0.160	0.022	6.1e-13	7.0e-05	51.8
Blood Pressure	DBP	rs4894808	3	171833266	C	G	0.106	0.018	6.8e-09	4.4e-05	33.5
Blood Pressure	DBP	rs7611674	3	179169230	T	G	0.158	0.022	1.7e-12	6.6e-05	49.9
Blood Pressure	DBP	rs12630496	3	183416341	T	C	-0.116	0.018	1.8e-10	5.4e-05	40.8
Blood Pressure	DBP	rs6779368	3	185298868	A	G	-0.179	0.018	2.3e-22	1.3e-04	94.7
Blood Pressure	DBP	rs147501096	3	186180253	C	G	-0.196	0.034	9.9e-09	4.3e-05	32.9
Blood Pressure	DBP	rs6776964	3	189304602	A	G	0.097	0.017	1.9e-08	4.2e-05	31.6
Blood Pressure	DBP	rs1706003	3	194299967	T	G	0.124	0.018	5.8e-12	6.3e-05	47.2
Blood Pressure	DBP	rs4244200	3	196226059	C	G	-0.122	0.019	3.2e-10	5.2e-05	39.6
Blood Pressure	DBP	rs6777317	3	197070959	A	G	0.125	0.020	1.5e-10	5.5e-05	41.0
Blood Pressure	DBP	rs61789369	4	2265295	A	G	-0.304	0.044	3.1e-12	6.4e-05	48.6

Phenotype	Trait	SNP	Chr	Position (GRCh37/hg19)	EA	NEA	BETA	SE	P-value	R2	F
Blood Pressure	DBP	rs16896276	4	18015156	A	T	-0.131	0.020	3.8e-11	5.8e-05	43.7
Blood Pressure	DBP	rs28667801	4	26785356	A	T	-0.162	0.018	1.9e-19	1.1e-04	81.2
Blood Pressure	DBP	rs1878825	4	36091370	C	G	-0.107	0.018	4.6e-09	4.6e-05	34.3
Blood Pressure	DBP	rs11721984	4	38343935	T	C	-0.141	0.018	1.9e-15	8.5e-05	63.4
Blood Pressure	DBP	rs62301873	4	40603821	A	G	-0.173	0.028	1.1e-09	4.9e-05	37.3
Blood Pressure	DBP	rs4998802	4	48800910	A	G	-0.107	0.020	4.4e-08	4.0e-05	29.9
Blood Pressure	DBP	rs871606	4	54799245	T	C	-0.184	0.028	8.6e-11	5.6e-05	42.2
Blood Pressure	DBP	rs11945489	4	56463775	T	C	-0.139	0.019	4.0e-13	6.9e-05	52.6
Blood Pressure	DBP	rs6551716	4	63575696	A	T	0.148	0.025	3.9e-09	4.6e-05	34.7
Blood Pressure	DBP	rs34587285	4	73349491	T	C	0.098	0.018	3.3e-08	4.0e-05	30.5
Blood Pressure	DBP	rs6838416	4	77378829	A	G	-0.121	0.019	8.2e-11	5.5e-05	42.0
Blood Pressure	DBP	rs12509595	4	81182554	T	C	-0.497	0.019	1.6e-148	8.9e-04	670.6
Blood Pressure	DBP	rs72976750	4	86725684	T	C	-0.172	0.025	7.4e-12	6.2e-05	46.8
Blood Pressure	DBP	rs1807870	4	89947671	A	G	0.103	0.018	5.9e-09	4.5e-05	34.0
Blood Pressure	DBP	rs7694000	4	95324968	A	T	-0.096	0.018	3.5e-08	4.0e-05	30.4
Blood Pressure	DBP	rs1527351	4	102084331	T	C	0.166	0.018	2.1e-19	1.1e-04	81.1
Blood Pressure	DBP	rs13107325	4	103188709	T	C	-0.675	0.034	3.7e-88	5.2e-04	396.1
Blood Pressure	DBP	rs1607095	4	104225289	T	C	-0.135	0.018	2.5e-14	7.7e-05	58.3
Blood Pressure	DBP	rs56388530	4	106910958	T	C	0.229	0.021	7.4e-29	1.6e-04	123.7
Blood Pressure	DBP	rs7694643	4	109017528	A	G	-0.132	0.018	3.1e-13	7.0e-05	53.0
Blood Pressure	DBP	rs35982905	4	111137737	A	T	-0.408	0.069	3.3e-09	4.7e-05	35.0
Blood Pressure	DBP	rs1879056	4	111335604	T	C	-0.216	0.022	6.2e-23	1.3e-04	97.2
Blood Pressure	DBP	rs66887589	4	120509279	T	C	-0.161	0.017	1.8e-20	1.1e-04	85.6
Blood Pressure	DBP	rs9286351	4	138441530	A	G	-0.141	0.018	1.6e-15	8.4e-05	63.6
Blood Pressure	DBP	rs72719149	4	144043336	T	C	-0.128	0.019	6.3e-12	6.3e-05	47.3
Blood Pressure	DBP	rs4292285	4	145271954	A	T	-0.107	0.018	1.3e-09	4.9e-05	36.7
Blood Pressure	DBP	rs10434099	4	148518051	A	C	0.105	0.018	4.4e-09	4.5e-05	34.2
Blood Pressure	DBP	rs4240358	4	151070611	A	G	0.126	0.021	4.2e-09	4.5e-05	34.4
Blood Pressure	DBP	rs1123037	4	156514725	A	T	-0.161	0.017	1.6e-20	1.1e-04	86.4
Blood Pressure	DBP	rs13139571	4	156645513	A	C	-0.241	0.020	2.3e-32	1.9e-04	140.7
Blood Pressure	DBP	rs1425486	4	157683685	T	C	-0.133	0.019	1.1e-12	6.8e-05	50.7
Blood Pressure	DBP	rs10069690	5	1279790	T	C	0.162	0.021	1.4e-14	8.1e-05	59.1
Blood Pressure	DBP	rs954767	5	3706050	A	C	-0.150	0.020	4.2e-14	7.6e-05	57.1
Blood Pressure	DBP	rs2921604	5	14867948	T	C	-0.096	0.018	4.5e-08	3.9e-05	29.8
Blood Pressure	DBP	rs1177764	5	32829975	C	G	-0.307	0.018	1.4e-67	4.0e-04	300.2
Blood Pressure	DBP	rs10941043	5	33194751	T	G	-0.127	0.019	2.5e-11	5.9e-05	44.6
Blood Pressure	DBP	rs2940928	5	42423905	A	G	-0.126	0.022	7.6e-09	4.4e-05	33.3
Blood Pressure	DBP	rs6875967	5	50878292	A	G	0.134	0.018	1.2e-13	7.3e-05	55.1
Blood Pressure	DBP	rs1694068	5	53283630	A	T	0.129	0.018	5.0e-13	6.9e-05	52.3
Blood Pressure	DBP	rs10054208	5	55688992	T	C	0.119	0.018	1.5e-10	5.4e-05	41.2
Blood Pressure	DBP	rs1035514	5	56037878	A	G	0.161	0.026	8.2e-10	5.0e-05	37.6
Blood Pressure	DBP	rs13167059	5	56362024	T	C	-0.108	0.018	7.9e-10	5.0e-05	37.7
Blood Pressure	DBP	rs12515541	5	57095011	T	G	0.116	0.018	6.2e-11	5.6e-05	42.7
Blood Pressure	DBP	rs1848510	5	57754005	A	G	0.126	0.018	4.1e-12	6.4e-05	48.2
Blood Pressure	DBP	rs10062049	5	61553881	T	C	0.221	0.025	4.5e-18	1.0e-04	75.0
Blood Pressure	DBP	rs6889240	5	66320940	A	G	0.120	0.020	1.0e-09	4.9e-05	37.3
Blood Pressure	DBP	rs10066799	5	67708390	T	G	0.138	0.019	9.4e-13	6.7e-05	51.0
Blood Pressure	DBP	rs9291932	5	68043243	T	C	-0.118	0.019	1.1e-09	4.9e-05	37.0
Blood Pressure	DBP	rs2307111	5	75003678	T	C	-0.174	0.018	1.6e-22	1.3e-04	95.8
Blood Pressure	DBP	rs4704514	5	77820081	T	C	0.109	0.019	1.7e-08	4.2e-05	31.7
Blood Pressure	DBP	rs10042590	5	87773318	A	G	-0.226	0.032	6.6e-13	6.8e-05	51.6
Blood Pressure	DBP	rs62380354	5	89484911	A	C	0.182	0.029	3.7e-10	5.2e-05	39.3
Blood Pressure	DBP	rs6887553	5	91845809	A	G	0.233	0.041	9.8e-09	4.4e-05	32.9
Blood Pressure	DBP	rs7731530	5	91983354	A	C	0.136	0.019	6.4e-13	6.8e-05	51.5
Blood Pressure	DBP	rs55770741	5	96220087	T	C	-0.128	0.018	2.2e-13	7.1e-05	53.6
Blood Pressure	DBP	rs1871190	5	97953719	T	G	0.108	0.019	6.6e-09	4.4e-05	33.6

Phenotype	Trait	SNP	Chr	Position (GRCh37/hg19)	EA	NEA	BETA	SE	P-value	R2	F
Blood Pressure	DBP	rs9326869	5	112349070	T	C	0.110	0.020	4.0e-08	4.0e-05	30.0
Blood Pressure	DBP	rs6894014	5	114393181	T	G	0.152	0.017	1.9e-18	1.0e-04	76.5
Blood Pressure	DBP	rs335170	5	122482243	A	C	0.113	0.018	1.6e-10	5.4e-05	40.8
Blood Pressure	DBP	rs1582931	5	122657199	A	G	0.216	0.018	4.5e-35	2.0e-04	152.5
Blood Pressure	DBP	rs6892983	5	127845030	A	C	0.200	0.018	9.5e-30	1.7e-04	128.6
Blood Pressure	DBP	rs1030271	5	130632655	C	G	0.132	0.019	1.6e-12	6.9e-05	49.8
Blood Pressure	DBP	rs736801	5	131833599	T	C	-0.156	0.018	7.0e-18	9.9e-05	74.4
Blood Pressure	DBP	rs66827546	5	132388960	A	G	-0.126	0.020	3.2e-10	5.2e-05	39.5
Blood Pressure	DBP	rs1212061	5	141723403	C	G	0.128	0.020	7.9e-11	5.6e-05	42.3
Blood Pressure	DBP	rs3776299	5	142507651	A	G	0.127	0.018	5.1e-13	7.0e-05	52.3
Blood Pressure	DBP	rs1800888	5	148206885	T	C	0.464	0.076	1.2e-09	5.0e-05	36.9
Blood Pressure	DBP	rs994446	5	148348395	A	G	-0.221	0.021	6.0e-25	1.4e-04	106.8
Blood Pressure	DBP	rs3117736	5	157462999	T	C	0.237	0.020	9.7e-34	1.9e-04	146.7
Blood Pressure	DBP	rs11960210	5	157817634	T	C	0.247	0.018	3.4e-43	2.5e-04	188.9
Blood Pressure	DBP	rs13358657	5	157938070	A	G	-0.224	0.025	1.7e-18	1.0e-04	77.2
Blood Pressure	DBP	rs17717527	5	158466283	A	T	-0.124	0.018	2.0e-12	6.6e-05	49.3
Blood Pressure	DBP	rs2546963	5	159522488	T	C	0.097	0.018	3.7e-08	4.1e-05	30.4
Blood Pressure	DBP	rs6555948	5	170804832	A	G	0.096	0.018	4.7e-08	3.9e-05	29.8
Blood Pressure	DBP	rs114503346	5	172192350	T	C	-0.268	0.043	3.1e-10	5.2e-05	39.5
Blood Pressure	DBP	rs55993676	5	173303392	T	G	-0.210	0.019	3.8e-28	1.6e-04	120.5
Blood Pressure	DBP	rs28362590	5	176731452	T	G	0.124	0.020	8.7e-10	5.0e-05	37.4
Blood Pressure	DBP	rs2569882	6	1620147	T	C	0.120	0.018	4.3e-11	5.7e-05	43.4
Blood Pressure	DBP	rs9406076	6	8023804	T	C	0.101	0.018	4.6e-08	3.9e-05	29.8
Blood Pressure	DBP	rs2282823	6	15478336	T	C	0.135	0.024	3.0e-08	4.0e-05	30.6
Blood Pressure	DBP	rs61408836	6	17302857	T	C	-0.128	0.022	5.7e-09	4.5e-05	34.0
Blood Pressure	DBP	rs35261542	6	20675792	A	C	0.120	0.020	9.3e-10	5.0e-05	37.6
Blood Pressure	DBP	rs1543310	6	22110418	T	C	-0.139	0.018	7.3e-15	8.1e-05	60.6
Blood Pressure	DBP	rs2744133	6	22392260	A	G	0.144	0.019	1.2e-13	7.3e-05	55.3
Blood Pressure	DBP	rs9467545	6	25638464	A	T	-0.254	0.024	7.4e-27	1.5e-04	115.3
Blood Pressure	DBP	rs198851	6	26104632	T	G	0.389	0.024	2.9e-57	3.4e-04	254.0
Blood Pressure	DBP	rs3846843	6	26427063	A	G	0.168	0.024	5.0e-12	6.4e-05	47.7
Blood Pressure	DBP	rs146754195	6	27177604	A	G	0.313	0.056	1.9e-08	4.2e-05	31.5
Blood Pressure	DBP	rs149866169	6	27441723	A	T	-0.262	0.027	3.8e-22	1.2e-04	93.6
Blood Pressure	DBP	rs11966966	6	28655388	T	C	0.251	0.020	3.1e-36	2.3e-04	158.6
Blood Pressure	DBP	rs3129039	6	29658864	A	G	-0.224	0.021	3.6e-26	1.5e-04	111.4
Blood Pressure	DBP	rs2245822	6	31230800	A	G	-0.186	0.021	1.3e-18	1.2e-04	77.3
Blood Pressure	DBP	rs1077393	6	31610529	A	G	-0.248	0.018	2.3e-43	2.6e-04	191.3
Blood Pressure	DBP	rs116479312	6	32408500	T	G	0.466	0.068	9.9e-12	7.0e-05	46.3
Blood Pressure	DBP	rs3129716	6	32657436	T	C	0.349	0.027	5.5e-39	2.5e-04	170.7
Blood Pressure	DBP	rs115245297	6	34244132	T	C	-0.312	0.044	1.1e-12	6.7e-05	50.8
Blood Pressure	DBP	rs4714224	6	39186743	C	G	-0.136	0.020	3.8e-12	6.4e-05	48.0
Blood Pressure	DBP	rs6905288	6	43758873	A	G	0.176	0.018	7.8e-23	1.3e-04	96.6
Blood Pressure	DBP	rs9472135	6	43809802	T	C	0.155	0.019	4.3e-16	8.8e-05	66.4
Blood Pressure	DBP	rs9369465	6	44169237	A	G	-0.110	0.018	5.9e-10	5.2e-05	38.5
Blood Pressure	DBP	rs78648104	6	50683009	T	C	-0.241	0.031	8.1e-15	8.0e-05	60.2
Blood Pressure	DBP	rs56351548	6	51581548	T	C	-0.162	0.026	5.7e-10	5.1e-05	38.3
Blood Pressure	DBP	rs1114347	6	51834297	A	G	-0.179	0.017	3.3e-25	1.4e-04	107.3
Blood Pressure	DBP	rs62413470	6	55999537	A	G	-0.166	0.024	9.1e-12	6.2e-05	46.6
Blood Pressure	DBP	rs504691	6	72206620	A	C	-0.118	0.018	3.1e-11	5.9e-05	44.2
Blood Pressure	DBP	rs1984195	6	79657391	A	G	0.174	0.017	1.4e-23	1.3e-04	100.7
Blood Pressure	DBP	rs7753695	6	80818531	T	C	0.103	0.018	4.8e-09	4.5e-05	34.3
Blood Pressure	DBP	rs7763102	6	82279237	A	C	0.112	0.020	2.6e-08	4.1e-05	31.0
Blood Pressure	DBP	rs16875357	6	85652904	T	G	-0.120	0.020	2.7e-09	4.7e-05	35.2
Blood Pressure	DBP	rs3798293	6	97033370	A	G	-0.133	0.021	2.7e-10	5.3e-05	40.0
Blood Pressure	DBP	rs9496614	6	100613551	T	C	0.131	0.020	1.6e-10	5.4e-05	41.0
Blood Pressure	DBP	rs72613227	6	106320771	A	T	-0.188	0.028	3.9e-11	6.3e-05	43.7

Phenotype	Trait	SNP	Chr	Position (GRCh37/hg19)	EA	NEA	BETA	SE	P-value	R2	F
Blood Pressure	DBP	rs9386780	6	109584410	A	G	0.098	0.017	1.5e-08	4.3e-05	32.2
Blood Pressure	DBP	rs3822857	6	116313931	C	G	-0.124	0.018	6.1e-12	6.2e-05	47.3
Blood Pressure	DBP	rs2693560	6	117523671	A	G	-0.150	0.018	1.1e-16	9.1e-05	68.8
Blood Pressure	DBP	rs17574630	6	117819718	A	G	-0.125	0.022	9.7e-09	4.3e-05	32.8
Blood Pressure	DBP	rs11153730	6	118667522	T	C	0.155	0.017	2.6e-19	1.1e-04	80.4
Blood Pressure	DBP	rs9375033	6	121787498	A	T	-0.123	0.018	6.7e-12	6.2e-05	47.0
Blood Pressure	DBP	rs13215166	6	127164360	A	G	-0.309	0.017	1.8e-70	4.2e-04	316.2
Blood Pressure	DBP	rs12192720	6	134195719	A	G	-0.109	0.019	1.2e-08	4.3e-05	32.6
Blood Pressure	DBP	rs9399136	6	135402339	T	C	0.117	0.020	3.8e-09	4.6e-05	34.9
Blood Pressure	DBP	rs668459	6	139835689	T	C	-0.113	0.018	1.0e-10	5.5e-05	41.8
Blood Pressure	DBP	rs645144	6	141180780	T	C	0.101	0.018	4.1e-08	4.0e-05	30.0
Blood Pressure	DBP	rs13195550	6	143159284	T	C	0.132	0.019	1.7e-12	6.6e-05	49.8
Blood Pressure	DBP	rs9399437	6	143636526	A	G	0.125	0.020	2.1e-10	5.3e-05	40.4
Blood Pressure	DBP	rs588273	6	147591371	A	G	-0.115	0.018	7.8e-11	5.6e-05	42.5
Blood Pressure	DBP	rs62434124	6	150999751	T	C	-0.485	0.034	7.8e-47	2.7e-04	206.2
Blood Pressure	DBP	rs9478282	6	152398669	T	C	-0.199	0.028	8.7e-13	6.8e-05	51.1
Blood Pressure	DBP	rs9479509	6	153427265	A	G	-0.115	0.019	1.2e-09	4.9e-05	36.8
Blood Pressure	DBP	rs9456648	6	161712235	T	C	-0.117	0.018	2.8e-10	5.2e-05	39.7
Blood Pressure	DBP	rs12665161	6	163749986	A	G	0.121	0.018	1.6e-11	6.0e-05	45.2
Blood Pressure	DBP	rs4709746	6	164133001	T	C	-0.145	0.026	2.2e-08	4.1e-05	31.2
Blood Pressure	DBP	rs11961593	6	166164137	T	C	-0.316	0.035	1.5e-19	1.1e-04	81.9
Blood Pressure	DBP	rs1322639	6	169587103	A	G	-0.158	0.021	3.9e-14	7.6e-05	57.4
Blood Pressure	DBP	rs73033340	7	1195692	A	G	0.531	0.052	5.1e-24	1.4e-04	102.4
Blood Pressure	DBP	rs6959688	7	1966831	A	G	-0.127	0.018	1.0e-12	6.7e-05	50.8
Blood Pressure	DBP	rs2906152	7	2523003	A	G	-0.187	0.018	5.5e-25	1.4e-04	107.1
Blood Pressure	DBP	rs1468520	7	7290732	A	G	-0.164	0.023	2.5e-12	6.5e-05	49.0
Blood Pressure	DBP	rs13240040	7	14375977	A	G	0.119	0.019	4.0e-10	5.2e-05	39.0
Blood Pressure	DBP	rs818508	7	16719917	T	C	-0.120	0.021	1.5e-08	4.3e-05	32.3
Blood Pressure	DBP	rs11543651	7	18590659	T	C	-0.116	0.019	6.6e-10	5.0e-05	38.1
Blood Pressure	DBP	rs4507656	7	22156538	C	G	-0.149	0.020	8.7e-14	7.8e-05	55.8
Blood Pressure	DBP	rs896312	7	25935030	T	C	0.149	0.018	5.7e-16	8.7e-05	65.7
Blood Pressure	DBP	rs1725074	7	27144921	T	C	0.141	0.026	3.9e-08	4.0e-05	30.2
Blood Pressure	DBP	rs3735533	7	27245893	T	C	-0.487	0.033	6.3e-49	2.9e-04	216.5
Blood Pressure	DBP	rs6961048	7	27328187	C	G	-0.273	0.029	1.3e-21	1.2e-04	91.0
Blood Pressure	DBP	rs33916666	7	35306718	T	C	0.123	0.018	2.1e-12	6.6e-05	49.6
Blood Pressure	DBP	rs342989	7	35467896	A	G	0.163	0.021	3.0e-15	8.3e-05	62.1
Blood Pressure	DBP	rs7777835	7	45124008	T	C	-0.104	0.018	1.3e-08	4.3e-05	32.4
Blood Pressure	DBP	rs2854746	7	45960645	C	G	0.113	0.018	3.3e-10	5.2e-05	39.4
Blood Pressure	DBP	rs17454517	7	50915776	A	G	0.122	0.017	2.7e-12	6.5e-05	48.8
Blood Pressure	DBP	rs1178979	7	72856430	T	C	0.150	0.022	1.0e-11	6.2e-05	46.3
Blood Pressure	DBP	rs3807101	7	80393418	T	C	-0.174	0.026	4.6e-11	5.7e-05	43.3
Blood Pressure	DBP	rs39281	7	89778489	A	G	0.128	0.018	4.0e-13	6.9e-05	52.5
Blood Pressure	DBP	rs1947228	7	96461649	T	C	-0.145	0.018	2.6e-16	9.0e-05	66.9
Blood Pressure	DBP	rs7788746	7	99612405	T	G	-0.164	0.018	3.2e-19	1.1e-04	80.7
Blood Pressure	DBP	rs7797740	7	100618993	A	G	0.174	0.027	9.3e-11	5.6e-05	42.0
Blood Pressure	DBP	rs12536606	7	107907875	T	C	-0.123	0.020	3.2e-10	5.3e-05	39.8
Blood Pressure	DBP	rs2402668	7	123296605	A	T	-0.107	0.019	1.0e-08	4.3e-05	32.9
Blood Pressure	DBP	rs11556924	7	129663496	T	C	-0.181	0.018	1.8e-23	1.3e-04	100.0
Blood Pressure	DBP	rs10228513	7	131069461	A	T	0.138	0.017	2.6e-15	8.2e-05	62.4
Blood Pressure	DBP	rs75511781	7	131323710	A	G	-0.372	0.047	2.5e-15	8.6e-05	62.7
Blood Pressure	DBP	rs12535680	7	131343205	A	G	-0.110	0.018	2.8e-09	4.7e-05	35.4
Blood Pressure	DBP	rs1722886	7	134215259	A	T	0.122	0.018	3.7e-12	6.4e-05	48.2
Blood Pressure	DBP	rs141212865	7	139404666	A	C	0.148	0.022	2.5e-11	5.9e-05	44.4
Blood Pressure	DBP	rs3735318	7	149152906	A	G	-0.101	0.017	4.7e-09	4.5e-05	34.4
Blood Pressure	DBP	rs1044608	7	150502016	C	G	-0.202	0.034	2.8e-09	4.7e-05	35.4
Blood Pressure	DBP	rs3918226	7	150690176	T	C	0.612	0.033	5.3e-77	4.6e-04	345.7

Phenotype	Trait	SNP	Chr	Position (GRCh37/hg19)	EA	NEA	BETA	SE	P-value	R2	F
Blood Pressure	DBP	rs4726006	7	150878803	A	G	0.134	0.020	2.4e-11	5.9e-05	44.8
Blood Pressure	DBP	rs6464165	7	151413124	T	C	-0.217	0.020	7.3e-29	1.6e-04	123.8
Blood Pressure	DBP	rs9638084	7	156311745	A	G	0.115	0.018	8.5e-11	5.6e-05	42.0
Blood Pressure	DBP	rs11136373	8	1212030	C	G	-0.106	0.019	1.3e-08	4.3e-05	32.3
Blood Pressure	DBP	rs2922895	8	6379932	C	G	0.132	0.018	5.9e-14	7.5e-05	56.6
Blood Pressure	DBP	rs7387360	8	8922464	T	C	0.146	0.018	1.0e-16	9.1e-05	69.3
Blood Pressure	DBP	rs35091929	8	10693492	T	C	0.183	0.018	6.5e-25	1.4e-04	106.7
Blood Pressure	DBP	rs13269417	8	11791962	T	C	0.145	0.018	1.4e-16	9.0e-05	68.2
Blood Pressure	DBP	rs56335308	8	17419461	A	G	0.355	0.057	6.3e-10	5.1e-05	38.3
Blood Pressure	DBP	rs62503324	8	23400615	T	C	0.203	0.020	2.1e-23	1.3e-04	99.3
Blood Pressure	DBP	rs951914	8	25878995	C	G	0.190	0.019	5.1e-23	1.3e-04	97.3
Blood Pressure	DBP	rs4284049	8	25987666	T	G	-0.125	0.022	2.4e-08	4.1e-05	31.1
Blood Pressure	DBP	rs17321041	8	26445194	T	C	0.231	0.036	1.8e-10	5.4e-05	40.6
Blood Pressure	DBP	rs1842646	8	26743933	A	C	0.098	0.018	2.6e-08	4.1e-05	30.9
Blood Pressure	DBP	rs2979470	8	30288272	T	C	0.102	0.017	4.6e-09	4.6e-05	34.5
Blood Pressure	DBP	rs1906672	8	38130025	A	G	0.140	0.020	8.5e-12	6.2e-05	46.8
Blood Pressure	DBP	rs72639047	8	49415598	A	G	-0.153	0.026	2.3e-09	4.7e-05	35.7
Blood Pressure	DBP	rs4873492	8	51947549	T	C	0.140	0.023	1.3e-09	4.9e-05	36.8
Blood Pressure	DBP	rs11778153	8	64503942	T	C	0.119	0.018	5.8e-11	5.7e-05	42.9
Blood Pressure	DBP	rs7817000	8	68919463	A	C	0.107	0.019	1.8e-08	4.2e-05	31.8
Blood Pressure	DBP	rs7843475	8	72475748	C	G	-0.115	0.020	7.2e-09	4.5e-05	33.6
Blood Pressure	DBP	rs4007357	8	77245410	A	G	0.102	0.019	4.2e-08	3.9e-05	29.9
Blood Pressure	DBP	rs148401029	8	81386066	A	C	-0.312	0.049	1.3e-10	5.4e-05	41.3
Blood Pressure	DBP	rs56345595	8	82814156	A	G	0.133	0.018	5.2e-14	7.5e-05	56.4
Blood Pressure	DBP	rs2142141	8	90940205	C	G	-0.105	0.018	3.7e-09	4.6e-05	34.7
Blood Pressure	DBP	rs843078	8	92616140	A	G	-0.107	0.018	1.3e-09	4.9e-05	37.0
Blood Pressure	DBP	rs11781001	8	96044076	A	G	0.155	0.024	1.1e-10	5.5e-05	41.5
Blood Pressure	DBP	rs2978098	8	101676675	A	C	0.155	0.018	1.3e-18	1.0e-04	77.4
Blood Pressure	DBP	rs142449193	8	102750597	T	C	-0.257	0.043	1.5e-09	4.8e-05	36.5
Blood Pressure	DBP	rs2513877	8	103883630	A	G	-0.129	0.022	4.2e-09	4.6e-05	34.6
Blood Pressure	DBP	rs2957468	8	106325360	A	G	0.138	0.018	8.4e-14	7.3e-05	55.4
Blood Pressure	DBP	rs722783	8	120442287	A	G	-0.209	0.021	9.0e-24	1.3e-04	101.3
Blood Pressure	DBP	rs13270541	8	124794849	A	G	0.120	0.021	1.3e-08	4.3e-05	32.3
Blood Pressure	DBP	rs12114418	8	126521465	A	G	-0.148	0.021	1.1e-12	6.7e-05	50.8
Blood Pressure	DBP	rs4380895	8	129383675	A	T	0.121	0.021	1.8e-08	4.2e-05	31.8
Blood Pressure	DBP	rs4909314	8	135623798	A	T	0.134	0.018	3.4e-14	7.6e-05	57.2
Blood Pressure	DBP	rs11167039	8	142245646	A	G	-0.178	0.030	3.6e-09	4.6e-05	34.9
Blood Pressure	DBP	rs60818561	8	142368160	C	G	-0.306	0.040	1.4e-14	7.8e-05	59.3
Blood Pressure	DBP	rs6983488	8	143455701	A	C	-0.102	0.018	2.5e-08	4.1e-05	31.0
Blood Pressure	DBP	rs3802230	8	143992864	A	C	-0.160	0.017	2.8e-20	1.1e-04	85.1
Blood Pressure	DBP	rs11782138	8	144965498	A	G	-0.276	0.045	1.1e-09	5.1e-05	37.1
Blood Pressure	DBP	rs1536608	9	223613	T	G	0.106	0.018	1.7e-09	4.8e-05	36.3
Blood Pressure	DBP	rs12216886	9	2493751	T	G	0.129	0.022	4.8e-09	4.5e-05	34.2
Blood Pressure	DBP	rs1332812	9	9350986	A	T	-0.114	0.018	2.7e-10	5.3e-05	40.0
Blood Pressure	DBP	rs35287509	9	10594635	T	C	-0.108	0.018	4.2e-09	4.6e-05	34.6
Blood Pressure	DBP	rs4615669	9	21818674	A	G	-0.114	0.017	6.1e-11	5.7e-05	42.9
Blood Pressure	DBP	rs1243876	9	35693104	T	C	-0.106	0.019	2.1e-08	4.1e-05	31.3
Blood Pressure	DBP	rs76452347	9	35906471	T	C	-0.225	0.023	9.4e-23	1.3e-04	96.2
Blood Pressure	DBP	rs11141731	9	89888472	T	C	-0.126	0.021	1.3e-09	4.9e-05	36.9
Blood Pressure	DBP	rs7020564	9	109670016	A	T	-0.111	0.019	6.7e-09	4.4e-05	33.5
Blood Pressure	DBP	rs7043304	9	112358150	T	C	0.176	0.025	1.6e-12	6.6e-05	50.0
Blood Pressure	DBP	rs10980408	9	113249071	T	C	-0.374	0.048	4.2e-15	8.1e-05	61.6
Blood Pressure	DBP	rs10759697	9	117172307	A	G	0.131	0.017	3.9e-14	7.6e-05	57.2
Blood Pressure	DBP	rs112332688	9	119083371	A	G	-0.114	0.021	4.4e-08	3.9e-05	29.9
Blood Pressure	DBP	rs1861881	9	119312256	T	G	0.115	0.019	6.5e-10	5.1e-05	38.2
Blood Pressure	DBP	rs937481	9	128194443	C	G	0.139	0.018	2.7e-15	8.3e-05	62.7

Phenotype	Trait	SNP	Chr	Position (GRCh37/hg19)	EA	NEA	BETA	SE	P-value	R2	F
Blood Pressure	DBP	rs4837127	9	129792141	T	C	0.117	0.021	2.6e-08	4.1e-05	31.1
Blood Pressure	DBP	rs507666	9	136149399	A	G	-0.285	0.022	2.3e-37	2.2e-04	163.8
Blood Pressure	DBP	rs6271	9	136522274	T	C	-0.431	0.035	1.7e-34	2.0e-04	150.1
Blood Pressure	DBP	rs11145807	9	139520789	A	G	0.155	0.018	4.1e-17	9.6e-05	71.0
Blood Pressure	DBP	rs11252324	10	4124568	T	G	-0.234	0.033	1.0e-12	6.7e-05	50.9
Blood Pressure	DBP	rs10906391	10	13523937	T	C	0.129	0.019	7.6e-12	6.2e-05	46.8
Blood Pressure	DBP	rs6602177	10	17167141	T	C	-0.120	0.021	6.5e-09	4.5e-05	33.8
Blood Pressure	DBP	rs1623474	10	18471794	T	C	0.223	0.018	6.2e-34	1.9e-04	147.4
Blood Pressure	DBP	rs12258967	10	18727959	C	G	0.354	0.019	3.3e-75	4.4e-04	336.4
Blood Pressure	DBP	rs3802517	10	28233469	A	T	0.129	0.017	9.3e-14	7.3e-05	55.3
Blood Pressure	DBP	rs1265842	10	28924901	T	C	0.111	0.017	1.7e-10	5.4e-05	40.9
Blood Pressure	DBP	rs2478835	10	30317949	T	C	-0.109	0.018	5.0e-10	5.2e-05	38.6
Blood Pressure	DBP	rs72816405	10	31350083	A	G	0.130	0.022	4.7e-09	4.5e-05	34.3
Blood Pressure	DBP	rs76164690	10	32590362	T	G	-0.154	0.025	7.2e-10	5.0e-05	37.9
Blood Pressure	DBP	rs4948643	10	45379759	T	C	0.159	0.019	2.3e-16	8.9e-05	67.3
Blood Pressure	DBP	rs34130368	10	48411796	T	G	-0.203	0.028	8.8e-13	6.7e-05	50.9
Blood Pressure	DBP	rs4948550	10	60588553	T	C	-0.111	0.019	1.0e-08	4.4e-05	33.0
Blood Pressure	DBP	rs10761530	10	62390726	T	C	0.117	0.017	1.1e-11	6.1e-05	46.3
Blood Pressure	DBP	rs72831343	10	63515681	T	G	0.494	0.025	4.8e-88	5.3e-04	396.1
Blood Pressure	DBP	rs2236295	10	64564892	T	G	-0.207	0.018	1.4e-31	1.8e-04	136.8
Blood Pressure	DBP	rs35732435	10	65120962	T	C	0.150	0.019	1.7e-15	8.3e-05	63.2
Blood Pressure	DBP	rs28564120	10	70330410	A	G	-0.125	0.022	1.2e-08	4.3e-05	32.4
Blood Pressure	DBP	rs12247028	10	75410052	A	G	-0.140	0.019	1.2e-13	7.4e-05	55.1
Blood Pressure	DBP	rs7098414	10	82214586	A	C	0.112	0.020	1.0e-08	4.3e-05	32.8
Blood Pressure	DBP	rs303217	10	91153699	T	C	-0.096	0.018	4.1e-08	4.1e-05	30.1
Blood Pressure	DBP	rs2274224	10	96039597	C	G	-0.279	0.017	1.2e-57	3.4e-04	256.7
Blood Pressure	DBP	rs1006545	10	102553647	T	G	0.363	0.028	8.0e-40	2.3e-04	174.5
Blood Pressure	DBP	rs10883560	10	102673707	C	G	0.119	0.018	1.0e-11	6.1e-05	46.1
Blood Pressure	DBP	rs72845603	10	103835807	T	C	0.282	0.043	4.1e-11	5.8e-05	43.7
Blood Pressure	DBP	rs12414028	10	104957629	A	T	-0.514	0.031	1.6e-60	3.6e-04	269.8
Blood Pressure	DBP	rs11191614	10	104977603	T	C	0.202	0.025	1.1e-15	8.5e-05	64.3
Blood Pressure	DBP	rs11191801	10	105532165	A	C	-0.119	0.019	4.7e-10	5.1e-05	38.6
Blood Pressure	DBP	rs7082671	10	106781426	A	G	0.497	0.069	5.5e-13	7.0e-05	52.1
Blood Pressure	DBP	rs111777102	10	111965826	T	C	0.214	0.035	1.6e-09	4.8e-05	36.5
Blood Pressure	DBP	rs11196549	10	115707298	A	G	0.445	0.045	2.9e-23	1.3e-04	98.8
Blood Pressure	DBP	rs2484294	10	115792062	A	G	0.316	0.020	1.2e-58	3.4e-04	260.8
Blood Pressure	DBP	rs72842207	10	121433675	T	C	-0.211	0.021	1.1e-23	1.3e-04	100.2
Blood Pressure	DBP	rs11592107	10	122968964	A	G	0.120	0.019	1.2e-10	5.5e-05	41.4
Blood Pressure	DBP	rs10490923	10	124214251	A	G	0.153	0.026	5.0e-09	4.5e-05	34.2
Blood Pressure	DBP	rs9419374	10	133729749	A	G	0.116	0.018	3.4e-10	5.2e-05	39.6
Blood Pressure	DBP	rs1133400	10	134459388	A	G	-0.132	0.021	8.3e-10	5.1e-05	37.6
Blood Pressure	DBP	rs28570096	11	1616088	T	C	0.140	0.019	1.1e-13	7.3e-05	55.1
Blood Pressure	DBP	rs79889784	11	1702117	T	G	-0.394	0.072	3.9e-08	4.4e-05	30.2
Blood Pressure	DBP	rs569550	11	1887068	T	G	-0.269	0.018	1.2e-49	3.0e-04	220.5
Blood Pressure	DBP	rs74048200	11	2120298	A	G	-0.197	0.033	1.5e-09	5.0e-05	36.7
Blood Pressure	DBP	rs17224476	11	4673788	A	G	0.160	0.028	7.4e-09	4.4e-05	33.5
Blood Pressure	DBP	rs4315061	11	8247020	T	C	0.125	0.018	2.2e-12	6.5e-05	49.0
Blood Pressure	DBP	rs2173030	11	9802442	C	G	0.237	0.028	2.1e-17	9.5e-05	72.0
Blood Pressure	DBP	rs117828113	11	9966605	T	C	0.234	0.035	3.5e-11	5.8e-05	43.9
Blood Pressure	DBP	rs10840378	11	10273128	T	C	0.278	0.022	3.3e-36	2.1e-04	158.2
Blood Pressure	DBP	rs2098839	11	10676987	T	C	0.175	0.018	1.3e-21	1.2e-04	91.3
Blood Pressure	DBP	rs10832013	11	13295353	T	G	0.152	0.019	4.5e-16	8.7e-05	66.2
Blood Pressure	DBP	rs10832300	11	14788963	A	G	-0.130	0.018	2.1e-13	7.1e-05	53.9
Blood Pressure	DBP	rs10832586	11	16304089	A	C	-0.308	0.022	2.5e-46	2.7e-04	203.7
Blood Pressure	DBP	rs7926335	11	16917869	T	C	0.180	0.020	2.0e-20	1.1e-04	85.6
Blood Pressure	DBP	rs11026586	11	22515533	A	G	0.290	0.034	2.7e-17	9.4e-05	71.4

Phenotype	Trait	SNP	Chr	Position (GRCh37/hg19)	EA	NEA	BETA	SE	P-value	R2	F
Blood Pressure	DBP	rs10835149	11	27295678	T	C	-0.115	0.019	2.2e-09	4.8e-05	35.8
Blood Pressure	DBP	rs962369	11	27734420	T	C	0.168	0.019	6.0e-19	1.1e-04	79.4
Blood Pressure	DBP	rs61879810	11	31821467	A	G	0.164	0.024	1.4e-11	6.0e-05	45.8
Blood Pressure	DBP	rs72910057	11	46331362	T	G	0.240	0.028	3.4e-17	9.4e-05	70.9
Blood Pressure	DBP	rs10838702	11	47410888	T	G	0.238	0.018	1.3e-40	2.4e-04	178.0
Blood Pressure	DBP	rs12280469	11	48701823	A	G	0.240	0.026	3.4e-20	1.2e-04	85.1
Blood Pressure	DBP	rs13328893	11	50272145	T	C	-0.195	0.024	1.7e-15	8.4e-05	63.2
Blood Pressure	DBP	rs11246486	11	51292160	T	C	-0.225	0.027	1.4e-16	9.5e-05	68.2
Blood Pressure	DBP	rs11228953	11	55042898	T	G	-0.215	0.027	1.3e-15	8.6e-05	64.1
Blood Pressure	DBP	rs7927898	11	56195905	T	C	-0.143	0.019	1.1e-13	7.3e-05	55.2
Blood Pressure	DBP	rs509564	11	57448117	T	C	0.148	0.021	1.4e-12	6.6e-05	50.3
Blood Pressure	DBP	rs7927966	11	58376276	T	C	-0.153	0.020	2.9e-14	7.6e-05	57.7
Blood Pressure	DBP	rs751984	11	61278246	T	C	0.394	0.028	1.4e-46	2.7e-04	205.0
Blood Pressure	DBP	rs35927325	11	63882495	T	C	0.222	0.036	1.0e-09	4.9e-05	37.2
Blood Pressure	DBP	rs2306363	11	65405600	T	G	-0.264	0.022	1.6e-34	2.0e-04	149.7
Blood Pressure	DBP	rs67976715	11	68023742	C	G	0.133	0.021	9.8e-11	5.5e-05	41.9
Blood Pressure	DBP	rs11605229	11	69075538	T	C	-0.247	0.028	2.4e-18	1.0e-04	76.5
Blood Pressure	DBP	rs586459	11	69461374	C	G	-0.103	0.018	5.7e-09	4.5e-05	33.9
Blood Pressure	DBP	rs875106	11	70005641	A	G	-0.133	0.017	1.7e-14	7.8e-05	59.0
Blood Pressure	DBP	rs504217	11	72006086	T	C	0.274	0.034	2.5e-16	9.0e-05	67.1
Blood Pressure	DBP	rs2298807	11	73068571	T	C	0.123	0.021	4.9e-09	4.5e-05	34.1
Blood Pressure	DBP	rs4420291	11	74374950	A	G	0.097	0.017	2.2e-08	4.1e-05	31.1
Blood Pressure	DBP	rs7115331	11	76218590	T	G	-0.127	0.019	3.9e-11	5.7e-05	43.5
Blood Pressure	DBP	rs117980989	11	77634537	A	T	0.171	0.028	1.7e-09	4.8e-05	36.3
Blood Pressure	DBP	rs2450128	11	77940075	A	G	-0.150	0.024	3.5e-10	5.2e-05	39.3
Blood Pressure	DBP	rs2289123	11	89224718	T	G	-0.119	0.021	2.9e-08	4.1e-05	30.6
Blood Pressure	DBP	rs11021221	11	95308854	A	T	-0.188	0.023	6.9e-16	8.6e-05	64.9
Blood Pressure	DBP	rs61909958	11	96151677	C	G	0.128	0.023	2.2e-08	4.1e-05	31.3
Blood Pressure	DBP	rs604723	11	100610546	T	C	-0.385	0.019	2.3e-87	5.2e-04	393.4
Blood Pressure	DBP	rs7951348	11	107081841	T	C	0.153	0.017	1.0e-18	1.0e-04	77.9
Blood Pressure	DBP	rs11608122	11	111535105	T	C	0.124	0.020	3.0e-10	5.3e-05	39.9
Blood Pressure	DBP	rs4938235	11	116097385	A	C	0.131	0.024	3.9e-08	4.0e-05	30.2
Blood Pressure	DBP	rs7116797	11	116707338	A	G	0.184	0.028	5.2e-11	5.8e-05	43.0
Blood Pressure	DBP	rs12790943	11	120058623	T	C	-0.100	0.018	1.1e-08	4.3e-05	32.8
Blood Pressure	DBP	rs12574332	11	122521123	T	C	0.207	0.027	6.1e-15	8.0e-05	60.7
Blood Pressure	DBP	rs4421757	11	122610115	A	G	0.098	0.017	1.6e-08	4.2e-05	31.8
Blood Pressure	DBP	rs66518071	11	128762184	C	G	0.125	0.021	6.1e-09	4.5e-05	33.9
Blood Pressure	DBP	rs4936099	11	130280725	A	C	0.174	0.018	1.2e-22	1.3e-04	96.1
Blood Pressure	DBP	rs55935819	12	2521579	A	G	0.127	0.018	2.0e-12	6.5e-05	49.3
Blood Pressure	DBP	rs75507123	12	5417856	T	G	-0.143	0.026	3.9e-08	4.0e-05	30.2
Blood Pressure	DBP	rs11055034	12	12890626	A	C	-0.134	0.020	5.9e-12	6.3e-05	47.6
Blood Pressure	DBP	rs28444248	12	15259020	T	C	0.157	0.024	1.0e-10	5.6e-05	42.0
Blood Pressure	DBP	rs61912333	12	19554817	C	G	0.119	0.018	1.1e-11	6.1e-05	45.8
Blood Pressure	DBP	rs4306343	12	20190630	A	T	-0.317	0.019	8.2e-61	3.6e-04	269.8
Blood Pressure	DBP	rs6487076	12	20470857	A	G	0.174	0.021	8.7e-17	9.1e-05	69.3
Blood Pressure	DBP	rs7136518	12	24163241	T	C	-0.099	0.018	4.5e-08	4.0e-05	30.0
Blood Pressure	DBP	rs11837982	12	26470813	A	C	0.150	0.018	2.1e-16	8.9e-05	67.4
Blood Pressure	DBP	rs7965392	12	42540280	A	G	0.112	0.018	4.2e-10	5.2e-05	39.0
Blood Pressure	DBP	rs11168245	12	48204499	C	G	0.176	0.020	1.1e-17	9.7e-05	73.5
Blood Pressure	DBP	rs117913411	12	48254353	A	T	0.401	0.049	1.5e-16	9.0e-05	68.0
Blood Pressure	DBP	rs150857355	12	49209340	C	G	0.410	0.064	1.8e-10	5.5e-05	40.7
Blood Pressure	DBP	rs7967705	12	50511408	T	C	0.269	0.018	1.5e-51	3.0e-04	229.1
Blood Pressure	DBP	rs145667436	12	51574697	A	T	0.262	0.042	6.5e-10	5.1e-05	38.1
Blood Pressure	DBP	rs3782480	12	52305069	T	G	0.151	0.027	3.4e-08	4.0e-05	30.5
Blood Pressure	DBP	rs7134440	12	53450097	T	C	0.228	0.032	1.8e-12	6.6e-05	49.7
Blood Pressure	DBP	rs6580970	12	54434277	T	C	-0.166	0.019	4.0e-18	1.0e-04	75.6

Phenotype	Trait	SNP	Chr	Position (GRCh37/hg19)	EA	NEA	BETA	SE	P-value	R2	F
Blood Pressure	DBP	rs705696	12	56480648	A	G	-0.105	0.018	1.2e-08	4.3e-05	32.3
Blood Pressure	DBP	rs7137749	12	57098040	T	C	0.141	0.018	7.2e-15	8.0e-05	60.9
Blood Pressure	DBP	rs10437954	12	58003922	A	G	-0.206	0.031	2.1e-11	5.9e-05	44.8
Blood Pressure	DBP	rs7959649	12	67783108	T	C	0.117	0.020	8.1e-09	4.4e-05	33.3
Blood Pressure	DBP	rs521033	12	69951428	A	G	-0.180	0.025	1.1e-12	6.7e-05	50.7
Blood Pressure	DBP	rs710698	12	70369918	A	G	0.106	0.018	1.9e-09	4.8e-05	36.2
Blood Pressure	DBP	rs1245829	12	79648170	A	T	0.107	0.018	1.4e-09	4.9e-05	36.8
Blood Pressure	DBP	rs2681485	12	90025622	A	G	0.294	0.018	1.3e-62	3.7e-04	280.0
Blood Pressure	DBP	rs4761524	12	94144175	A	G	0.096	0.017	2.8e-08	4.1e-05	30.9
Blood Pressure	DBP	rs11108209	12	96109855	T	C	-0.190	0.030	2.4e-10	5.3e-05	40.2
Blood Pressure	DBP	rs11112548	12	105871914	A	T	0.274	0.044	5.8e-10	5.1e-05	38.3
Blood Pressure	DBP	rs141391620	12	110681099	A	G	0.301	0.038	4.5e-15	8.1e-05	61.5
Blood Pressure	DBP	rs61942582	12	110803794	A	G	-0.236	0.034	6.7e-12	6.2e-05	47.0
Blood Pressure	DBP	rs7137828	12	111932800	T	C	-0.503	0.018	4.8e-180	1.1e-03	815.8
Blood Pressure	DBP	rs3741983	12	112939853	T	C	-0.315	0.023	2.0e-43	2.6e-04	190.6
Blood Pressure	DBP	rs4766994	12	113143565	T	C	0.143	0.021	2.1e-11	6.0e-05	45.1
Blood Pressure	DBP	rs10850377	12	115201436	A	G	0.104	0.018	1.5e-08	4.3e-05	31.9
Blood Pressure	DBP	rs7316428	12	115335672	C	G	-0.157	0.020	4.6e-15	8.1e-05	61.7
Blood Pressure	DBP	rs35443	12	115552878	C	G	-0.266	0.018	1.2e-50	2.9e-04	223.5
Blood Pressure	DBP	rs10850526	12	115934758	A	G	-0.202	0.019	1.6e-26	1.5e-04	114.0
Blood Pressure	DBP	rs3898618	12	120813921	T	C	-0.268	0.038	2.5e-12	6.5e-05	49.0
Blood Pressure	DBP	rs148775688	12	122144944	T	C	0.383	0.068	1.4e-08	4.4e-05	32.1
Blood Pressure	DBP	rs11059094	12	122606837	T	C	-0.150	0.017	6.2e-18	9.8e-05	74.6
Blood Pressure	DBP	rs1727311	12	123660716	T	G	-0.203	0.022	4.0e-20	1.1e-04	84.4
Blood Pressure	DBP	rs1271309	12	124820705	A	G	-0.198	0.024	1.5e-16	9.0e-05	68.0
Blood Pressure	DBP	rs682681	13	22294062	T	C	-0.145	0.018	4.5e-15	8.2e-05	61.8
Blood Pressure	DBP	rs55641580	13	25257917	T	C	0.174	0.026	4.8e-11	5.7e-05	43.4
Blood Pressure	DBP	rs9508495	13	30146201	T	C	-0.194	0.020	1.3e-21	1.2e-04	90.8
Blood Pressure	DBP	rs9532243	13	32191408	A	C	0.134	0.017	1.1e-14	7.9e-05	59.9
Blood Pressure	DBP	rs7321585	13	38263176	C	G	0.119	0.020	1.6e-09	4.8e-05	36.5
Blood Pressure	DBP	rs56256111	13	41478963	A	G	0.193	0.026	2.6e-13	7.1e-05	53.6
Blood Pressure	DBP	rs7992292	13	41968013	A	G	0.137	0.023	3.2e-09	4.6e-05	35.0
Blood Pressure	DBP	rs59086590	13	50498925	A	C	-0.151	0.025	7.5e-10	5.0e-05	37.8
Blood Pressure	DBP	rs9526707	13	51489186	A	G	-0.122	0.019	6.6e-11	5.7e-05	42.8
Blood Pressure	DBP	rs9563529	13	58316637	T	G	0.122	0.021	1.4e-08	4.3e-05	32.3
Blood Pressure	DBP	rs3861113	13	72364382	A	C	0.213	0.032	3.9e-11	5.8e-05	43.6
Blood Pressure	DBP	rs78474310	13	73826901	A	G	-0.243	0.042	9.7e-09	4.3e-05	32.9
Blood Pressure	DBP	rs1215469	13	80707408	A	C	-0.138	0.021	5.2e-11	5.7e-05	43.0
Blood Pressure	DBP	rs55684003	13	97988689	A	G	0.122	0.019	1.0e-10	5.5e-05	41.7
Blood Pressure	DBP	rs650724	13	110804809	A	G	-0.197	0.031	1.3e-10	5.5e-05	41.3
Blood Pressure	DBP	rs4773140	13	110954237	A	G	-0.138	0.021	2.9e-11	6.0e-05	44.3
Blood Pressure	DBP	rs7491960	13	114470370	T	C	-0.129	0.018	8.4e-13	7.2e-05	51.2
Blood Pressure	DBP	rs7321688	13	115000365	A	C	0.151	0.020	2.0e-13	7.2e-05	54.0
Blood Pressure	DBP	rs7350752	14	21841154	A	G	-0.150	0.027	2.0e-08	4.2e-05	31.5
Blood Pressure	DBP	rs17880989	14	23313633	A	G	0.401	0.059	1.1e-11	6.4e-05	46.1
Blood Pressure	DBP	rs1950500	14	24830850	T	C	0.140	0.019	2.2e-13	7.1e-05	54.0
Blood Pressure	DBP	rs2273171	14	31381351	T	C	-0.098	0.017	1.3e-08	4.3e-05	32.4
Blood Pressure	DBP	rs4424827	14	35110857	T	C	-0.098	0.018	2.1e-08	4.1e-05	31.4
Blood Pressure	DBP	rs7155504	14	36158828	T	C	0.229	0.032	5.2e-13	6.9e-05	52.0
Blood Pressure	DBP	rs72683923	14	50735947	T	C	0.532	0.064	5.0e-17	9.3e-05	70.3
Blood Pressure	DBP	rs35413927	14	53420358	A	G	-0.127	0.019	1.8e-11	6.0e-05	45.4
Blood Pressure	DBP	rs11628933	14	60700903	C	G	-0.122	0.021	3.1e-09	4.7e-05	35.2
Blood Pressure	DBP	rs731681	14	68010224	C	G	-0.107	0.017	8.0e-10	5.0e-05	37.9
Blood Pressure	DBP	rs57786342	14	69260028	A	G	0.142	0.022	4.4e-11	5.7e-05	43.4
Blood Pressure	DBP	rs227426	14	70456664	T	G	0.112	0.018	1.7e-10	5.4e-05	40.9
Blood Pressure	DBP	rs2239268	14	72469591	A	G	0.110	0.019	7.4e-09	4.4e-05	33.3

Phenotype	Trait	SNP	Chr	Position (GRCh37/hg19)	EA	NEA	BETA	SE	P-value	R2	F
Blood Pressure	DBP	rs4903064	14	73279420	T	C	0.154	0.021	7.8e-14	7.4e-05	56.1
Blood Pressure	DBP	rs7151887	14	100184783	A	G	0.121	0.020	3.1e-09	4.6e-05	34.9
Blood Pressure	DBP	rs8014182	14	103859962	T	C	-0.194	0.026	3.9e-14	7.5e-05	57.1
Blood Pressure	DBP	rs10873612	15	26105602	T	C	-0.110	0.018	9.5e-10	5.0e-05	37.5
Blood Pressure	DBP	rs11070245	15	40317792	T	G	-0.129	0.017	1.6e-13	7.3e-05	54.7
Blood Pressure	DBP	rs2925345	15	41311799	T	C	0.189	0.017	1.6e-27	1.6e-04	118.0
Blood Pressure	DBP	rs17678552	15	42066190	T	C	-0.165	0.018	1.3e-19	1.1e-04	82.1
Blood Pressure	DBP	rs2927071	15	43919081	T	C	-0.139	0.019	6.4e-13	6.8e-05	51.5
Blood Pressure	DBP	rs11854184	15	49293194	A	C	0.134	0.021	2.3e-10	5.3e-05	40.1
Blood Pressure	DBP	rs7169864	15	53902901	T	C	-0.113	0.020	3.4e-08	4.0e-05	30.5
Blood Pressure	DBP	rs113631817	15	66827783	A	G	0.119	0.021	8.8e-09	4.4e-05	33.1
Blood Pressure	DBP	rs28429256	15	66931617	A	G	0.164	0.019	2.8e-18	1.0e-04	75.7
Blood Pressure	DBP	rs62004794	15	68454523	A	G	-0.096	0.017	3.4e-08	4.0e-05	30.6
Blood Pressure	DBP	rs11853359	15	71621524	A	G	-0.166	0.018	1.3e-19	1.1e-04	82.3
Blood Pressure	DBP	rs11636952	15	75114322	T	C	0.400	0.019	5.2e-99	6.0e-04	447.2
Blood Pressure	DBP	rs62012629	15	79070351	A	C	-0.186	0.021	2.4e-19	1.1e-04	80.9
Blood Pressure	DBP	rs2627316	15	81042816	A	G	-0.154	0.017	7.4e-19	1.1e-04	78.7
Blood Pressure	DBP	rs983353	15	82186535	A	G	-0.109	0.019	8.5e-09	4.4e-05	33.0
Blood Pressure	DBP	rs11259905	15	83949751	A	C	0.130	0.021	1.3e-09	4.9e-05	36.8
Blood Pressure	DBP	rs7180952	15	85162551	T	C	-0.101	0.018	9.8e-09	4.3e-05	32.8
Blood Pressure	DBP	rs7171498	15	86244247	T	C	-0.114	0.018	6.2e-11	5.6e-05	42.7
Blood Pressure	DBP	rs78550103	15	90027296	A	G	-0.181	0.025	3.5e-13	7.1e-05	53.0
Blood Pressure	DBP	rs4932373	15	91429287	A	C	-0.366	0.019	7.7e-84	5.1e-04	375.8
Blood Pressure	DBP	rs873122	15	92702020	C	G	0.121	0.020	6.5e-10	5.1e-05	38.1
Blood Pressure	DBP	rs28533980	15	94207872	T	C	0.100	0.018	2.5e-08	4.2e-05	31.2
Blood Pressure	DBP	rs12906962	15	95312071	T	C	-0.238	0.019	8.7e-37	2.1e-04	160.0
Blood Pressure	DBP	rs4984496	15	96635898	T	G	0.176	0.019	4.9e-21	1.2e-04	88.9
Blood Pressure	DBP	rs2577002	15	96796260	A	C	0.096	0.017	2.7e-08	4.1e-05	31.0
Blood Pressure	DBP	rs2570816	15	100159557	C	G	0.141	0.023	6.0e-10	5.1e-05	38.4
Blood Pressure	DBP	rs9932866	16	706067	A	G	0.115	0.018	2.9e-10	5.3e-05	39.8
Blood Pressure	DBP	rs28590346	16	2080653	A	T	-0.191	0.019	9.8e-24	1.4e-04	100.4
Blood Pressure	DBP	rs76073047	16	2222769	A	G	0.230	0.042	2.8e-08	4.2e-05	30.8
Blood Pressure	DBP	rs2240075	16	3535540	A	G	-0.148	0.020	4.3e-14	7.5e-05	56.9
Blood Pressure	DBP	rs11647570	16	4137262	A	G	0.165	0.028	3.8e-09	4.6e-05	34.6
Blood Pressure	DBP	rs12446456	16	4922201	T	C	-0.181	0.018	4.0e-25	1.4e-04	107.0
Blood Pressure	DBP	rs1126889	16	12061910	C	G	-0.106	0.018	7.6e-09	4.4e-05	33.5
Blood Pressure	DBP	rs57327054	16	14487036	T	C	-0.117	0.019	8.1e-10	5.0e-05	37.7
Blood Pressure	DBP	rs77924615	16	20392332	A	G	-0.316	0.022	3.7e-45	2.6e-04	199.4
Blood Pressure	DBP	rs9937801	16	21088130	T	C	0.155	0.017	4.8e-19	1.1e-04	79.8
Blood Pressure	DBP	rs80095680	16	30902353	A	G	-0.157	0.020	2.8e-15	8.3e-05	62.6
Blood Pressure	DBP	rs10468291	16	49768046	A	C	-0.117	0.018	3.7e-11	5.9e-05	43.9
Blood Pressure	DBP	rs12149258	16	50536826	A	G	0.152	0.024	2.6e-10	5.3e-05	40.0
Blood Pressure	DBP	rs9932220	16	51758116	A	G	-0.159	0.021	3.8e-14	7.6e-05	57.4
Blood Pressure	DBP	rs12919839	16	56859216	T	C	-0.110	0.019	1.0e-08	4.3e-05	32.7
Blood Pressure	DBP	rs2060664	16	60652439	T	C	0.109	0.020	4.6e-08	3.9e-05	29.8
Blood Pressure	DBP	rs45474499	16	66914492	T	C	0.356	0.042	8.5e-18	9.7e-05	73.7
Blood Pressure	DBP	rs28544928	16	69329268	T	G	0.154	0.020	9.1e-15	7.9e-05	60.1
Blood Pressure	DBP	rs4788444	16	71856547	A	G	-0.145	0.024	1.4e-09	4.9e-05	36.8
Blood Pressure	DBP	rs62055084	16	73097845	T	C	0.119	0.021	1.3e-08	4.4e-05	32.3
Blood Pressure	DBP	rs11859505	16	74195719	A	G	-0.104	0.018	9.8e-09	4.4e-05	32.8
Blood Pressure	DBP	rs8046697	16	75442144	T	C	-0.129	0.018	6.1e-13	6.9e-05	51.9
Blood Pressure	DBP	rs12929303	16	81602264	A	G	0.157	0.017	1.6e-19	1.1e-04	81.6
Blood Pressure	DBP	rs7500448	16	83045790	A	G	-0.130	0.020	1.1e-10	5.5e-05	41.6
Blood Pressure	DBP	rs35781150	16	86082389	A	G	-0.124	0.021	4.2e-09	4.6e-05	34.5
Blood Pressure	DBP	rs9939182	16	86566596	T	C	0.253	0.042	1.3e-09	4.9e-05	36.8
Blood Pressure	DBP	rs908951	16	89697625	T	C	-0.198	0.018	7.7e-28	1.6e-04	120.0

Phenotype	Trait	SNP	Chr	Position (GRCh37/hg19)	EA	NEA	BETA	SE	P-value	R2	F
Blood Pressure	DBP	rs4362428	17	2090341	A	C	-0.113	0.018	1.5e-10	5.4e-05	41.0
Blood Pressure	DBP	rs7215084	17	3880148	T	C	0.112	0.017	1.2e-10	5.5e-05	41.6
Blood Pressure	DBP	rs12601936	17	7172609	A	G	-0.143	0.018	1.1e-15	8.5e-05	64.5
Blood Pressure	DBP	rs138420351	17	7700063	T	C	0.557	0.085	7.1e-11	5.8e-05	42.5
Blood Pressure	DBP	rs74439044	17	7781019	T	C	-0.350	0.029	1.4e-32	1.9e-04	141.4
Blood Pressure	DBP	rs1465249	17	12639294	T	C	0.096	0.018	3.6e-08	4.0e-05	30.3
Blood Pressure	DBP	rs9893005	17	16225506	C	G	-0.120	0.018	7.9e-12	6.2e-05	46.9
Blood Pressure	DBP	rs7221807	17	18221799	T	C	-0.121	0.018	5.4e-12	6.2e-05	47.3
Blood Pressure	DBP	rs1043809	17	19239432	T	C	0.159	0.022	9.8e-13	6.8e-05	50.8
Blood Pressure	DBP	rs76954792	17	30033514	T	C	0.121	0.021	5.1e-09	4.5e-05	34.0
Blood Pressure	DBP	rs28661492	17	30609932	T	C	-0.136	0.022	9.6e-10	5.0e-05	37.5
Blood Pressure	DBP	rs7213273	17	43155914	A	G	-0.180	0.018	3.6e-23	1.3e-04	98.0
Blood Pressure	DBP	rs55938136	17	43798360	A	G	0.141	0.022	4.2e-10	5.7e-05	39.2
Blood Pressure	DBP	rs4968248	17	44993128	A	G	-0.132	0.018	1.3e-13	7.2e-05	54.6
Blood Pressure	DBP	rs45572038	17	46690322	T	C	0.356	0.051	2.3e-12	6.5e-05	49.2
Blood Pressure	DBP	rs35929648	17	47002543	A	G	-0.119	0.019	3.0e-10	5.3e-05	39.8
Blood Pressure	DBP	rs9889262	17	47398070	A	T	0.228	0.018	7.1e-37	2.1e-04	160.9
Blood Pressure	DBP	rs34430710	17	56876627	A	T	-0.129	0.019	3.8e-12	6.4e-05	48.2
Blood Pressure	DBP	rs2052074	17	59161646	A	G	-0.127	0.023	1.7e-08	4.2e-05	31.8
Blood Pressure	DBP	rs1000423	17	59475642	T	C	0.192	0.020	9.5e-22	1.2e-04	91.7
Blood Pressure	DBP	rs6504163	17	61545779	T	C	-0.184	0.018	6.3e-24	1.3e-04	101.3
Blood Pressure	DBP	rs1867624	17	62387091	T	C	0.141	0.018	2.1e-15	8.3e-05	62.9
Blood Pressure	DBP	rs12941755	17	64275523	C	G	-0.118	0.020	7.6e-09	4.4e-05	33.4
Blood Pressure	DBP	rs3744010	17	73840243	A	G	-0.143	0.020	2.0e-12	6.5e-05	49.4
Blood Pressure	DBP	rs1436138	17	75316880	A	G	0.199	0.018	7.3e-28	1.6e-04	119.7
Blood Pressure	DBP	rs78554005	17	76443843	T	G	-0.188	0.032	3.1e-09	4.7e-05	35.1
Blood Pressure	DBP	rs7217916	17	76769434	A	G	0.111	0.018	5.6e-10	5.1e-05	38.5
Blood Pressure	DBP	rs112280096	17	79367409	A	C	-0.119	0.019	6.9e-10	5.4e-05	38.2
Blood Pressure	DBP	rs11077961	17	81012749	A	G	0.107	0.019	8.5e-09	4.5e-05	33.3
Blood Pressure	DBP	rs7227492	18	772064	T	C	0.181	0.023	1.4e-15	8.4e-05	63.6
Blood Pressure	DBP	rs334414	18	7124612	T	C	-0.102	0.018	1.6e-08	4.2e-05	32.0
Blood Pressure	DBP	rs11665020	18	10879503	C	G	-0.142	0.019	2.8e-14	7.7e-05	57.9
Blood Pressure	DBP	rs4800420	18	20158965	A	G	0.119	0.019	5.2e-10	5.1e-05	38.5
Blood Pressure	DBP	rs1025655	18	24596211	A	G	-0.108	0.018	1.3e-09	4.9e-05	37.0
Blood Pressure	DBP	rs10164193	18	31161426	T	G	-0.220	0.033	1.9e-11	6.0e-05	45.1
Blood Pressure	DBP	rs11661473	18	42177123	A	G	0.201	0.020	1.5e-24	1.4e-04	104.9
Blood Pressure	DBP	rs9967367	18	42606091	T	C	0.140	0.019	2.0e-13	7.1e-05	53.8
Blood Pressure	DBP	rs57091267	18	48170771	A	G	-0.180	0.022	2.7e-16	8.8e-05	66.9
Blood Pressure	DBP	rs11082866	18	48789736	A	T	-0.121	0.019	4.2e-10	5.1e-05	39.0
Blood Pressure	DBP	rs34163044	18	51851616	A	C	0.149	0.018	9.6e-17	9.1e-05	68.9
Blood Pressure	DBP	rs12605156	18	53498114	A	T	0.142	0.022	1.5e-10	5.4e-05	41.2
Blood Pressure	DBP	rs10048404	18	54578482	T	C	-0.110	0.018	2.0e-09	4.7e-05	35.9
Blood Pressure	DBP	rs7235890	18	55732115	T	G	-0.169	0.029	4.1e-09	4.6e-05	34.5
Blood Pressure	DBP	rs12454712	18	60845884	T	C	0.105	0.019	2.7e-08	4.3e-05	31.0
Blood Pressure	DBP	rs4891258	18	72995537	A	G	-0.116	0.019	5.7e-10	5.1e-05	38.4
Blood Pressure	DBP	rs12609484	19	4970593	T	G	-0.140	0.019	1.2e-13	7.3e-05	55.3
Blood Pressure	DBP	rs12978472	19	7257990	C	G	0.478	0.028	8.5e-65	3.9e-04	289.2
Blood Pressure	DBP	rs12463045	19	7295964	T	C	0.164	0.024	1.8e-11	6.3e-05	45.1
Blood Pressure	DBP	rs2009733	19	8398714	A	G	0.122	0.018	5.1e-12	6.5e-05	47.8
Blood Pressure	DBP	rs10409243	19	10332988	T	C	-0.120	0.018	2.9e-11	5.9e-05	44.4
Blood Pressure	DBP	rs111427795	19	11272727	T	G	0.119	0.019	2.1e-10	5.4e-05	40.2
Blood Pressure	DBP	rs390938	19	11510205	A	G	0.207	0.032	1.2e-10	5.5e-05	41.5
Blood Pressure	DBP	rs167479	19	11526765	T	G	-0.362	0.019	1.7e-82	5.4e-04	370.8
Blood Pressure	DBP	rs66502847	19	12615646	A	G	0.099	0.018	1.8e-08	4.2e-05	31.8
Blood Pressure	DBP	rs3745318	19	16436262	T	C	0.140	0.021	1.3e-11	6.2e-05	45.9
Blood Pressure	DBP	rs1077795	19	17222584	A	G	0.199	0.020	1.6e-23	1.3e-04	99.7

Phenotype	Trait	SNP	Chr	Position (GRCh37/hg19)	EA	NEA	BETA	SE	P-value	R2	F
Blood Pressure	DBP	rs2288464	19	17417252	A	C	-0.168	0.029	1.0e-08	4.3e-05	32.7
Blood Pressure	DBP	rs72999033	19	19366632	T	C	0.279	0.036	5.9e-15	8.1e-05	60.9
Blood Pressure	DBP	rs6511291	19	21950402	T	C	-0.116	0.018	6.9e-11	5.7e-05	42.8
Blood Pressure	DBP	rs7257694	19	30314666	T	C	0.184	0.018	6.3e-25	1.4e-04	106.5
Blood Pressure	DBP	rs8108717	19	31911914	A	G	0.132	0.018	1.4e-13	7.3e-05	54.6
Blood Pressure	DBP	rs12983238	19	39438532	A	G	-0.127	0.020	3.2e-10	5.3e-05	39.7
Blood Pressure	DBP	rs55710016	19	40828730	A	G	0.116	0.017	2.7e-11	5.9e-05	44.2
Blood Pressure	DBP	rs11083560	19	41100406	C	G	-0.147	0.020	6.9e-14	7.4e-05	56.0
Blood Pressure	DBP	rs11882467	19	41754217	T	G	0.111	0.019	8.0e-09	4.4e-05	33.3
Blood Pressure	DBP	rs73036520	19	45749484	C	G	0.156	0.020	1.3e-14	7.9e-05	59.4
Blood Pressure	DBP	rs55706574	19	47556180	A	G	-0.114	0.019	3.2e-09	4.9e-05	35.2
Blood Pressure	DBP	rs679574	19	49206108	C	G	-0.133	0.018	3.4e-14	7.7e-05	57.4
Blood Pressure	DBP	rs73046792	19	49605705	A	G	-0.152	0.024	5.9e-10	5.1e-05	38.4
Blood Pressure	DBP	rs67535236	20	4097355	A	G	0.168	0.023	1.4e-13	7.2e-05	54.8
Blood Pressure	DBP	rs297690	20	4426656	T	G	-0.130	0.020	5.5e-11	5.7e-05	42.9
Blood Pressure	DBP	rs6076983	20	6331376	T	C	0.125	0.018	1.6e-12	6.6e-05	49.9
Blood Pressure	DBP	rs6039211	20	8616588	A	G	0.178	0.018	6.8e-23	1.3e-04	97.1
Blood Pressure	DBP	rs6077799	20	10426174	A	G	0.100	0.018	3.0e-08	4.1e-05	30.6
Blood Pressure	DBP	rs693974	20	10557252	T	C	-0.185	0.018	1.8e-25	1.4e-04	108.9
Blood Pressure	DBP	rs1327235	20	10969030	A	G	-0.302	0.017	4.8e-68	4.0e-04	304.3
Blood Pressure	DBP	rs6078393	20	11908101	T	G	0.120	0.018	7.7e-12	6.2e-05	46.9
Blood Pressure	DBP	rs2618647	20	17882452	A	G	-0.122	0.017	2.7e-12	6.5e-05	48.8
Blood Pressure	DBP	rs6046137	20	19466832	A	G	0.116	0.020	5.4e-09	4.5e-05	34.0
Blood Pressure	DBP	rs6137201	20	20957669	A	T	0.153	0.027	2.0e-08	4.2e-05	31.6
Blood Pressure	DBP	rs6060262	20	30193071	A	C	0.180	0.024	4.8e-14	7.7e-05	56.6
Blood Pressure	DBP	rs13042148	20	32298286	T	C	-0.167	0.024	7.2e-12	6.2e-05	47.1
Blood Pressure	DBP	rs4810332	20	40268334	A	T	-0.172	0.018	6.5e-21	1.2e-04	87.8
Blood Pressure	DBP	rs6031431	20	42795152	A	G	-0.115	0.018	4.9e-11	5.7e-05	43.4
Blood Pressure	DBP	rs2425757	20	44792921	T	C	-0.108	0.019	1.1e-08	4.3e-05	32.7
Blood Pressure	DBP	rs2598	20	47241618	A	G	0.139	0.018	1.9e-15	8.3e-05	62.8
Blood Pressure	DBP	rs79044887	20	47427831	C	G	0.243	0.024	4.0e-23	1.3e-04	98.1
Blood Pressure	DBP	rs6021247	20	50108980	A	G	0.130	0.017	8.2e-14	7.4e-05	55.6
Blood Pressure	DBP	rs2801008	20	51788718	T	G	-0.105	0.019	2.1e-08	4.2e-05	31.4
Blood Pressure	DBP	rs6026578	20	57463472	C	G	-0.131	0.018	7.4e-13	6.9e-05	51.6
Blood Pressure	DBP	rs6026739	20	57739469	A	T	-0.503	0.027	1.5e-79	4.7e-04	357.9
Blood Pressure	DBP	rs6062295	20	62291174	A	G	-0.120	0.018	1.2e-11	6.1e-05	45.8
Blood Pressure	DBP	rs2180631	20	62426393	T	C	0.237	0.038	3.7e-10	5.2e-05	39.4
Blood Pressure	DBP	rs35213536	20	62694319	T	G	0.204	0.020	2.5e-23	1.3e-04	99.4
Blood Pressure	DBP	rs1882961	21	16556367	T	C	0.127	0.019	1.4e-11	6.0e-05	45.8
Blood Pressure	DBP	rs71326977	21	35290975	A	C	-0.137	0.023	2.8e-09	4.7e-05	35.4
Blood Pressure	DBP	rs8128234	21	36470865	T	C	0.117	0.021	4.0e-08	4.0e-05	30.3
Blood Pressure	DBP	rs2836411	21	39819830	T	C	0.156	0.018	1.7e-17	9.5e-05	72.1
Blood Pressure	DBP	rs762384	21	40074396	T	C	0.151	0.020	2.0e-13	7.2e-05	54.3
Blood Pressure	DBP	rs1571737	21	43530671	T	C	0.130	0.023	1.5e-08	4.3e-05	32.2
Blood Pressure	DBP	rs12627514	21	44759440	C	G	-0.216	0.020	2.0e-28	1.6e-04	121.9
Blood Pressure	DBP	rs34487963	21	44838330	A	C	-0.573	0.071	8.2e-16	8.9e-05	64.9
Blood Pressure	DBP	rs7278003	21	44966069	T	C	-0.129	0.018	1.8e-13	7.1e-05	54.0
Blood Pressure	DBP	rs5992929	22	18451977	T	C	0.168	0.019	3.1e-18	1.0e-04	76.1
Blood Pressure	DBP	rs134041	22	28056338	T	C	0.122	0.018	3.1e-12	6.5e-05	48.8
Blood Pressure	DBP	rs12321	22	29453193	C	G	-0.149	0.018	1.4e-17	9.6e-05	72.7
Blood Pressure	DBP	rs12484046	22	31439650	A	G	0.102	0.018	3.7e-08	4.1e-05	30.5
Blood Pressure	DBP	rs9609429	22	32517431	T	C	0.120	0.020	6.3e-10	5.0e-05	38.1
Blood Pressure	DBP	rs240069	22	34083187	C	G	-0.133	0.024	3.5e-08	4.0e-05	30.5
Blood Pressure	DBP	rs2012831	22	36837260	A	G	0.107	0.019	1.8e-08	4.2e-05	31.6
Blood Pressure	PP	rs307359	1	1280014	A	G	-0.334	0.044	3.9e-14	8.5e-05	57.2
Blood Pressure	PP	rs7796	1	1684169	C	G	0.202	0.021	2.8e-21	1.2e-04	90.0

Phenotype	Trait	SNP	Chr	Position (GRCh37/hg19)	EA	NEA	BETA	SE	P-value	R2	F
Blood Pressure	PP	rs263532	1	2164116	T	C	0.119	0.021	1.2e-08	4.4e-05	32.5
Blood Pressure	PP	rs2493291	1	3329384	T	C	0.191	0.030	1.9e-10	5.6e-05	40.5
Blood Pressure	PP	rs11121483	1	6263792	A	G	0.148	0.022	3.1e-11	6.1e-05	44.0
Blood Pressure	PP	rs9661802	1	6678864	A	C	0.138	0.022	2.7e-10	5.4e-05	39.9
Blood Pressure	PP	rs4406655	1	9205179	A	G	-0.125	0.021	2.6e-09	4.8e-05	35.6
Blood Pressure	PP	rs1890683	1	9441477	T	C	-0.219	0.021	2.3e-25	1.5e-04	107.8
Blood Pressure	PP	rs17035646	1	10796547	A	G	0.246	0.022	4.9e-29	1.7e-04	124.9
Blood Pressure	PP	rs17037452	1	11895675	A	G	0.383	0.028	2.8e-43	2.6e-04	190.1
Blood Pressure	PP	rs7552400	1	16035892	A	G	0.217	0.023	1.7e-20	1.2e-04	86.0
Blood Pressure	PP	rs150266910	1	23442265	T	C	0.173	0.027	9.6e-11	5.7e-05	42.0
Blood Pressure	PP	rs6598886	1	27849300	T	C	0.218	0.037	3.0e-09	4.8e-05	35.3
Blood Pressure	PP	rs143167197	1	28734372	A	G	-0.307	0.042	2.5e-13	7.4e-05	53.6
Blood Pressure	PP	rs4360494	1	38455891	C	G	0.298	0.021	1.3e-43	2.6e-04	191.9
Blood Pressure	PP	rs11210568	1	42514452	T	C	0.191	0.021	2.7e-20	1.2e-04	85.5
Blood Pressure	PP	rs4660729	1	43767900	C	G	0.128	0.021	1.4e-09	5.0e-05	36.5
Blood Pressure	PP	rs512083	1	46027355	T	C	-0.126	0.020	9.5e-10	5.1e-05	37.5
Blood Pressure	PP	rs1278516	1	50900021	A	G	-0.244	0.035	2.0e-12	6.8e-05	49.5
Blood Pressure	PP	rs778124	1	56606206	A	G	0.224	0.021	2.7e-26	1.5e-04	112.6
Blood Pressure	PP	rs2492321	1	59351446	A	G	-0.143	0.025	1.5e-08	4.3e-05	32.0
Blood Pressure	PP	rs17535443	1	59646056	A	G	-0.365	0.023	7.6e-57	3.4e-04	251.7
Blood Pressure	PP	rs949827	1	59841785	T	C	0.152	0.022	2.6e-12	6.6e-05	48.8
Blood Pressure	PP	rs20354	1	67071356	T	G	0.209	0.030	3.0e-12	6.6e-05	48.6
Blood Pressure	PP	rs12034319	1	86040107	A	G	0.135	0.025	4.7e-08	4.0e-05	29.8
Blood Pressure	PP	rs385437	1	86822231	A	G	0.163	0.030	4.5e-08	4.1e-05	30.0
Blood Pressure	PP	rs1856047	1	87897907	A	G	-0.136	0.021	6.1e-11	5.9e-05	42.8
Blood Pressure	PP	rs786923	1	89242954	T	C	-0.198	0.021	4.0e-21	1.2e-04	89.3
Blood Pressure	PP	rs17516329	1	92319781	A	T	0.142	0.022	1.6e-10	5.6e-05	41.1
Blood Pressure	PP	rs10776752	1	113044328	T	G	0.374	0.039	1.5e-21	1.2e-04	90.8
Blood Pressure	PP	rs11585169	1	150572037	A	T	0.156	0.021	8.3e-14	7.6e-05	55.5
Blood Pressure	PP	rs12138150	1	169098738	T	C	-0.195	0.021	5.7e-21	1.2e-04	88.3
Blood Pressure	PP	rs12118102	1	176634724	A	G	0.356	0.046	8.4e-15	8.1e-05	60.1
Blood Pressure	PP	rs12739010	1	178910830	C	G	-0.115	0.021	4.9e-08	4.0e-05	29.6
Blood Pressure	PP	rs3753802	1	180140096	T	C	0.116	0.021	2.7e-08	4.2e-05	30.9
Blood Pressure	PP	rs1043069	1	180859368	T	G	0.124	0.021	4.8e-09	4.6e-05	34.3
Blood Pressure	PP	rs10912438	1	188525475	C	G	0.113	0.021	4.3e-08	4.1e-05	29.9
Blood Pressure	PP	rs4559481	1	193526470	A	G	-0.117	0.021	2.3e-08	4.2e-05	31.3
Blood Pressure	PP	rs1337005	1	197265641	T	C	0.112	0.020	4.8e-08	4.1e-05	29.9
Blood Pressure	PP	rs536070	1	201748800	T	C	-0.164	0.021	5.1e-15	8.3e-05	61.3
Blood Pressure	PP	rs558248	1	208047435	A	G	0.192	0.021	1.6e-19	1.1e-04	81.6
Blood Pressure	PP	rs17042848	1	216734001	A	T	0.150	0.026	1.6e-08	4.3e-05	31.9
Blood Pressure	PP	rs2820443	1	219753509	T	C	0.186	0.022	1.3e-16	9.3e-05	68.7
Blood Pressure	PP	rs57657087	1	228256020	T	G	-0.212	0.035	1.5e-09	5.0e-05	36.6
Blood Pressure	PP	rs2493134	1	230849359	T	C	-0.134	0.021	1.4e-10	5.7e-05	41.4
Blood Pressure	PP	rs2175337	2	9298590	A	C	0.166	0.021	3.5e-15	8.4e-05	62.2
Blood Pressure	PP	rs1344652	2	19731180	A	G	-0.346	0.022	3.9e-56	3.4e-04	248.9
Blood Pressure	PP	rs6531174	2	19767275	A	T	0.145	0.026	2.3e-08	4.3e-05	31.3
Blood Pressure	PP	rs62111832	2	19850924	A	G	0.237	0.041	9.8e-09	4.5e-05	32.9
Blood Pressure	PP	rs7255	2	20878820	T	C	-0.192	0.021	1.7e-20	1.2e-04	86.0
Blood Pressure	PP	rs10779936	2	23950200	A	G	-0.153	0.023	1.4e-11	6.2e-05	45.6
Blood Pressure	PP	rs2384063	2	25187115	T	C	0.160	0.024	4.5e-11	5.9e-05	43.2
Blood Pressure	PP	rs1275988	2	26914364	T	C	-0.250	0.021	5.3e-33	1.9e-04	143.2
Blood Pressure	PP	rs7562	2	28635740	T	C	0.124	0.021	1.8e-09	4.9e-05	36.1
Blood Pressure	PP	rs1545552	2	33360338	A	G	-0.127	0.023	2.0e-08	4.2e-05	31.4
Blood Pressure	PP	rs6734118	2	37559355	A	C	-0.161	0.025	9.6e-11	5.7e-05	41.8
Blood Pressure	PP	rs115262049	2	43196694	A	T	0.226	0.038	1.5e-09	4.9e-05	36.4
Blood Pressure	PP	rs6544652	2	43626212	T	C	-0.144	0.024	1.9e-09	4.9e-05	36.0

Phenotype	Trait	SNP	Chr	Position (GRCh37/hg19)	EA	NEA	BETA	SE	P-value	R2	F
Blood Pressure	PP	rs11690961	2	46363336	A	C	0.304	0.032	1.9e-21	1.2e-04	90.6
Blood Pressure	PP	rs11899888	2	56102744	A	G	0.234	0.028	2.0e-16	9.2e-05	67.8
Blood Pressure	PP	rs925484	2	60611437	C	G	-0.149	0.021	8.7e-13	7.0e-05	51.0
Blood Pressure	PP	rs6735330	2	61731478	A	G	0.159	0.029	4.3e-08	4.1e-05	30.0
Blood Pressure	PP	rs2540951	2	65276736	A	G	0.224	0.021	1.3e-26	1.5e-04	114.1
Blood Pressure	PP	rs268882	2	65502802	A	G	0.157	0.028	2.4e-08	4.2e-05	31.2
Blood Pressure	PP	rs6731373	2	68503044	A	G	0.134	0.022	1.4e-09	5.0e-05	36.5
Blood Pressure	PP	rs7578166	2	71630041	A	C	0.138	0.021	4.1e-11	5.9e-05	43.8
Blood Pressure	PP	rs11689667	2	85491365	T	C	0.203	0.021	7.4e-23	1.3e-04	97.0
Blood Pressure	PP	rs7058	2	96917588	T	G	0.180	0.021	1.9e-18	1.0e-04	76.7
Blood Pressure	PP	rs6747874	2	101578489	A	G	0.170	0.025	5.0e-12	6.5e-05	47.6
Blood Pressure	PP	rs150194832	2	106126880	C	G	-0.215	0.035	1.3e-09	5.0e-05	36.8
Blood Pressure	PP	rs4664080	2	152978341	A	G	-0.135	0.021	1.0e-10	5.7e-05	41.7
Blood Pressure	PP	rs55732192	2	162278233	T	G	-0.208	0.035	4.0e-09	4.7e-05	34.7
Blood Pressure	PP	rs72874178	2	164924573	A	G	-0.379	0.024	4.3e-55	3.3e-04	245.0
Blood Pressure	PP	rs75758489	2	165043823	T	C	-0.176	0.029	1.4e-09	5.0e-05	36.7
Blood Pressure	PP	rs560887	2	169763148	T	C	-0.190	0.022	1.6e-17	1.0e-04	72.9
Blood Pressure	PP	rs151054210	2	172381487	A	G	0.173	0.026	6.5e-11	5.8e-05	42.5
Blood Pressure	PP	rs11695888	2	174265303	A	T	-0.143	0.023	2.5e-10	5.4e-05	40.0
Blood Pressure	PP	rs72914576	2	175529967	C	G	-0.208	0.026	2.7e-15	8.5e-05	62.6
Blood Pressure	PP	rs11888604	2	177004026	T	C	0.175	0.023	6.7e-14	7.6e-05	56.2
Blood Pressure	PP	rs1837164	2	178716601	A	T	0.121	0.021	9.1e-09	4.5e-05	33.1
Blood Pressure	PP	rs80271311	2	179734144	T	C	-0.290	0.045	1.2e-10	5.6e-05	41.4
Blood Pressure	PP	rs10497529	2	179839888	A	G	-0.451	0.058	4.7e-15	8.3e-05	61.5
Blood Pressure	PP	rs6732308	2	182996188	A	G	0.168	0.026	5.3e-11	5.8e-05	43.1
Blood Pressure	PP	rs11901929	2	189643316	A	G	0.127	0.022	4.0e-09	4.7e-05	34.6
Blood Pressure	PP	rs7603849	2	191438741	A	C	-0.156	0.020	2.3e-14	8.0e-05	58.2
Blood Pressure	PP	rs295118	2	201144004	T	C	-0.137	0.021	5.1e-11	5.9e-05	43.1
Blood Pressure	PP	rs1469760	2	204125426	T	C	-0.189	0.021	1.2e-19	1.1e-04	82.7
Blood Pressure	PP	rs3845811	2	208521512	C	G	-0.161	0.021	1.6e-14	8.0e-05	59.0
Blood Pressure	PP	rs1250259	2	216300482	A	T	-0.278	0.023	8.6e-33	1.9e-04	142.6
Blood Pressure	PP	rs4674114	2	217659266	A	G	-0.212	0.026	1.3e-16	9.3e-05	68.3
Blood Pressure	PP	rs2161967	2	218680529	T	G	0.126	0.021	1.7e-09	4.9e-05	36.2
Blood Pressure	PP	rs2891076	2	219184398	T	C	-0.123	0.021	6.2e-09	4.6e-05	33.8
Blood Pressure	PP	rs13423088	2	227026864	A	T	-0.127	0.022	6.5e-09	4.6e-05	33.7
Blood Pressure	PP	rs4441458	2	228293218	T	C	-0.129	0.023	1.4e-08	4.4e-05	32.2
Blood Pressure	PP	rs12052878	2	238227594	A	G	-0.150	0.022	1.1e-11	6.3e-05	46.3
Blood Pressure	PP	rs1995496	2	242445336	A	G	-0.128	0.021	5.7e-10	5.2e-05	38.4
Blood Pressure	PP	rs9848170	3	11495983	C	G	0.193	0.021	2.4e-20	1.2e-04	85.7
Blood Pressure	PP	rs729639	3	13826854	T	C	-0.125	0.022	8.4e-09	4.5e-05	33.3
Blood Pressure	PP	rs13070927	3	14919646	T	G	-0.168	0.020	2.6e-16	9.1e-05	66.9
Blood Pressure	PP	rs189267552	3	20073193	A	T	-0.738	0.094	4.5e-15	8.4e-05	61.5
Blood Pressure	PP	rs2055120	3	27548040	A	G	-0.558	0.072	1.1e-14	8.2e-05	59.7
Blood Pressure	PP	rs75487052	3	27552361	A	T	0.262	0.021	9.5e-36	2.1e-04	156.2
Blood Pressure	PP	rs11710131	3	37282685	A	G	-0.158	0.021	7.8e-14	7.7e-05	56.0
Blood Pressure	PP	rs6801957	3	38767315	T	C	0.121	0.021	6.6e-09	4.6e-05	33.6
Blood Pressure	PP	rs6788984	3	41107173	A	G	0.188	0.029	1.3e-10	5.6e-05	41.3
Blood Pressure	PP	rs9839213	3	41918992	T	C	-0.532	0.028	4.0e-81	4.9e-04	363.0
Blood Pressure	PP	rs10433615	3	52638482	T	C	0.166	0.021	3.4e-15	8.4e-05	62.1
Blood Pressure	PP	rs2241823	3	63965093	A	C	-0.125	0.022	1.7e-08	4.3e-05	31.9
Blood Pressure	PP	rs7630745	3	66427029	T	C	0.164	0.021	2.7e-14	7.8e-05	57.9
Blood Pressure	PP	rs12634194	3	70578117	C	G	0.131	0.022	1.5e-09	5.0e-05	36.6
Blood Pressure	PP	rs729448	3	73260545	A	G	-0.130	0.021	2.8e-10	5.4e-05	39.6
Blood Pressure	PP	rs9835724	3	84964976	A	G	0.163	0.022	1.8e-13	7.3e-05	54.1
Blood Pressure	PP	rs9860290	3	99839106	A	G	-0.174	0.025	5.2e-12	6.4e-05	47.5
Blood Pressure	PP	rs1882289	3	114461208	A	G	-0.191	0.032	2.6e-09	4.8e-05	35.5

Phenotype	Trait	SNP	Chr	Position (GRCh37/hg19)	EA	NEA	BETA	SE	P-value	R2	F
Blood Pressure	PP	rs6806529	3	123049938	A	C	0.137	0.021	5.8e-11	5.9e-05	43.1
Blood Pressure	PP	rs12631961	3	124561848	T	C	-0.128	0.021	6.5e-10	5.2e-05	38.1
Blood Pressure	PP	rs60672471	3	128125718	T	C	0.192	0.033	8.0e-09	4.5e-05	33.2
Blood Pressure	PP	rs62270945	3	128201889	T	C	0.528	0.065	5.2e-16	9.1e-05	65.7
Blood Pressure	PP	rs11915143	3	133985901	A	G	0.131	0.021	4.1e-10	5.3e-05	39.0
Blood Pressure	PP	rs73230648	3	141628963	A	C	-0.160	0.028	7.6e-09	4.5e-05	33.3
Blood Pressure	PP	rs62278541	3	142631909	A	G	0.168	0.021	4.8e-15	8.3e-05	61.4
Blood Pressure	PP	rs9851265	3	149667426	A	C	0.127	0.022	7.1e-09	4.5e-05	33.5
Blood Pressure	PP	rs62271373	3	150066540	A	T	0.311	0.045	4.5e-12	6.5e-05	47.9
Blood Pressure	PP	rs677910	3	151872154	A	G	0.162	0.029	2.3e-08	4.2e-05	31.2
Blood Pressure	PP	rs56213443	3	160309043	A	C	-0.141	0.021	1.1e-11	6.3e-05	46.4
Blood Pressure	PP	rs9835962	3	168693089	A	C	-0.251	0.039	1.4e-10	5.6e-05	41.1
Blood Pressure	PP	rs1918973	3	169165173	A	G	0.125	0.020	9.9e-10	5.1e-05	37.4
Blood Pressure	PP	rs12630450	3	169480204	A	G	0.195	0.024	1.2e-16	9.4e-05	68.6
Blood Pressure	PP	rs4894535	3	171995605	T	C	0.185	0.028	4.7e-11	5.9e-05	43.3
Blood Pressure	PP	rs73171158	3	176927949	T	C	-0.129	0.022	2.8e-09	4.8e-05	35.2
Blood Pressure	PP	rs263017	3	183503017	A	G	0.136	0.020	3.2e-11	6.0e-05	44.2
Blood Pressure	PP	rs9855673	3	186207805	T	G	-0.115	0.020	1.8e-08	4.3e-05	31.8
Blood Pressure	PP	rs1773242	3	194290858	C	G	-0.119	0.022	5.0e-08	4.0e-05	29.8
Blood Pressure	PP	rs1250129	4	1254930	A	G	-0.207	0.032	1.3e-10	5.6e-05	41.3
Blood Pressure	PP	rs231708	4	2694773	C	G	-0.210	0.022	2.6e-21	1.2e-04	90.1
Blood Pressure	PP	rs2498323	4	3451109	A	G	0.296	0.035	3.1e-17	9.7e-05	71.4
Blood Pressure	PP	rs3796822	4	10093651	C	G	0.128	0.022	9.1e-09	4.5e-05	33.1
Blood Pressure	PP	rs4303974	4	15375984	A	C	-0.129	0.023	3.4e-08	4.2e-05	30.5
Blood Pressure	PP	rs11730129	4	16032948	T	C	-0.161	0.025	1.0e-10	5.7e-05	41.7
Blood Pressure	PP	rs2610990	4	18008232	A	G	-0.159	0.023	1.1e-11	6.3e-05	46.3
Blood Pressure	PP	rs28702684	4	38395515	C	G	-0.135	0.020	4.8e-11	5.9e-05	43.2
Blood Pressure	PP	rs2102397	4	48656326	A	C	0.162	0.021	1.6e-14	8.0e-05	59.2
Blood Pressure	PP	rs61794248	4	52680923	T	G	0.145	0.024	2.2e-09	4.9e-05	35.9
Blood Pressure	PP	rs60991988	4	54801228	T	G	0.529	0.034	1.4e-55	3.4e-04	246.8
Blood Pressure	PP	rs6851147	4	55056566	C	G	-0.190	0.023	2.0e-16	9.1e-05	67.5
Blood Pressure	PP	rs62308665	4	56477797	T	C	0.134	0.023	6.6e-09	4.5e-05	33.6
Blood Pressure	PP	rs28418670	4	77371434	C	G	-0.134	0.021	8.7e-11	5.7e-05	42.0
Blood Pressure	PP	rs10857147	4	81181072	A	T	-0.358	0.023	8.0e-54	3.2e-04	238.4
Blood Pressure	PP	rs6823199	4	83925895	T	C	0.157	0.024	3.1e-11	6.0e-05	44.1
Blood Pressure	PP	rs17010957	4	86719165	T	C	-0.346	0.029	2.3e-32	1.9e-04	140.1
Blood Pressure	PP	rs13149209	4	89750668	T	C	0.177	0.025	1.2e-12	6.9e-05	50.5
Blood Pressure	PP	rs4699299	4	95501166	T	C	0.128	0.022	7.3e-09	4.5e-05	33.4
Blood Pressure	PP	rs1229984	4	100239319	T	C	-0.511	0.061	3.6e-17	9.8e-05	70.9
Blood Pressure	PP	rs223408	4	103736345	A	T	0.159	0.025	1.8e-10	5.5e-05	40.7
Blood Pressure	PP	rs2726478	4	106254606	A	T	0.118	0.021	1.5e-08	4.4e-05	32.1
Blood Pressure	PP	rs56388530	4	106910958	T	C	0.183	0.024	4.2e-14	7.8e-05	57.0
Blood Pressure	PP	rs77301788	4	120935531	T	C	-0.163	0.022	1.7e-13	7.4e-05	54.6
Blood Pressure	PP	rs10010247	4	138466213	T	C	-0.116	0.021	3.3e-08	4.2e-05	30.6
Blood Pressure	PP	rs11933087	4	145722862	A	T	-0.121	0.022	2.8e-08	4.2e-05	31.0
Blood Pressure	PP	rs77866798	4	146660884	A	G	0.192	0.031	6.1e-10	5.2e-05	38.4
Blood Pressure	PP	rs4835266	4	146821725	T	C	0.164	0.021	4.2e-15	8.3e-05	61.4
Blood Pressure	PP	rs10305838	4	148400256	T	C	-0.254	0.029	4.6e-18	1.0e-04	75.3
Blood Pressure	PP	rs10002709	4	151156761	A	C	-0.124	0.021	7.1e-09	4.5e-05	33.5
Blood Pressure	PP	rs4691670	4	156403247	T	C	-0.236	0.020	1.1e-30	1.8e-04	132.4
Blood Pressure	PP	rs3796584	4	156641047	A	G	-0.121	0.021	1.2e-08	4.4e-05	32.6
Blood Pressure	PP	rs999958	4	169698873	A	C	-0.221	0.020	5.7e-27	1.6e-04	116.0
Blood Pressure	PP	rs67846163	4	174656889	A	G	0.142	0.025	1.0e-08	4.4e-05	32.7
Blood Pressure	PP	rs2242652	5	1280028	A	G	0.158	0.028	1.9e-08	4.5e-05	31.5
Blood Pressure	PP	rs7707563	5	15695932	T	C	0.154	0.024	8.8e-11	5.7e-05	42.1
Blood Pressure	PP	rs7733331	5	32828846	T	C	-0.338	0.021	6.0e-59	3.5e-04	261.2

Phenotype	Trait	SNP	Chr	Position (GRCh37/hg19)	EA	NEA	BETA	SE	P-value	R2	F
Blood Pressure	PP	rs10941043	5	33194751	T	G	-0.127	0.022	1.6e-08	4.3e-05	32.0
Blood Pressure	PP	rs10939913	5	50592825	C	G	-0.130	0.023	3.0e-08	4.2e-05	30.8
Blood Pressure	PP	rs6867399	5	52135543	A	C	0.140	0.024	7.0e-09	4.6e-05	33.7
Blood Pressure	PP	rs1694068	5	53283630	A	T	0.128	0.021	1.2e-09	5.0e-05	36.8
Blood Pressure	PP	rs464605	5	55807370	T	C	0.140	0.024	2.5e-09	4.8e-05	35.6
Blood Pressure	PP	rs9291825	5	64084524	A	G	-0.127	0.021	5.9e-10	5.2e-05	38.2
Blood Pressure	PP	rs26683	5	68024512	C	G	-0.129	0.023	1.5e-08	4.3e-05	32.1
Blood Pressure	PP	rs72761109	5	71506529	T	C	0.158	0.022	1.2e-12	6.8e-05	50.4
Blood Pressure	PP	rs4443403	5	72654304	T	C	-0.196	0.035	1.9e-08	4.3e-05	31.6
Blood Pressure	PP	rs10076149	5	77874854	C	G	0.179	0.020	2.9e-18	1.0e-04	76.1
Blood Pressure	PP	rs6452769	5	87389027	A	G	-0.159	0.026	4.6e-10	5.3e-05	38.8
Blood Pressure	PP	rs76443575	5	96211594	C	G	-0.317	0.055	1.0e-08	4.4e-05	32.8
Blood Pressure	PP	rs79409628	5	108113740	T	G	-0.309	0.037	5.2e-17	9.5e-05	70.3
Blood Pressure	PP	rs4487481	5	114381312	T	C	0.130	0.021	2.7e-10	5.4e-05	39.8
Blood Pressure	PP	rs9885577	5	121194226	T	C	0.162	0.022	1.0e-13	7.5e-05	55.3
Blood Pressure	PP	rs71594307	5	122156060	A	G	0.266	0.049	4.8e-08	4.0e-05	29.8
Blood Pressure	PP	rs1644318	5	122471989	T	C	-0.231	0.021	3.9e-28	1.6e-04	120.8
Blood Pressure	PP	rs75887402	5	122674563	T	C	0.529	0.065	4.1e-16	9.0e-05	66.3
Blood Pressure	PP	rs13189347	5	122828560	A	C	-0.141	0.021	8.1e-12	6.3e-05	46.7
Blood Pressure	PP	rs58595196	5	127558338	C	G	-0.152	0.024	3.5e-10	5.3e-05	39.4
Blood Pressure	PP	rs1435514	5	127842419	T	C	0.147	0.021	1.6e-12	6.8e-05	49.9
Blood Pressure	PP	rs702395	5	140086677	T	C	0.144	0.021	4.2e-12	6.5e-05	48.2
Blood Pressure	PP	rs853170	5	142537474	T	C	0.152	0.023	7.1e-11	5.8e-05	42.6
Blood Pressure	PP	rs2400509	5	147696018	A	G	-0.148	0.023	2.7e-10	5.4e-05	39.8
Blood Pressure	PP	rs4705324	5	148517441	A	C	-0.143	0.024	2.2e-09	4.9e-05	35.9
Blood Pressure	PP	rs157678	5	156145654	A	T	-0.146	0.022	6.8e-11	5.8e-05	42.7
Blood Pressure	PP	rs10076730	5	157816992	T	C	0.222	0.021	8.0e-26	1.5e-04	110.4
Blood Pressure	PP	rs10052777	5	158269081	T	C	-0.246	0.021	1.7e-31	1.9e-04	136.9
Blood Pressure	PP	rs193986	5	172500742	A	G	-0.122	0.021	5.1e-09	4.7e-05	34.1
Blood Pressure	PP	rs12153395	5	179411477	A	G	-0.213	0.033	1.0e-10	5.7e-05	41.8
Blood Pressure	PP	rs2569842	6	1649032	T	C	0.150	0.023	3.8e-11	5.9e-05	43.7
Blood Pressure	PP	rs6920534	6	7519183	T	C	0.274	0.036	1.6e-14	8.0e-05	58.8
Blood Pressure	PP	rs9392172	6	7723962	C	G	-0.184	0.020	2.6e-19	1.1e-04	81.0
Blood Pressure	PP	rs1630736	6	12295987	T	C	-0.131	0.021	4.7e-10	5.3e-05	38.8
Blood Pressure	PP	rs9349379	6	12903957	A	G	0.268	0.021	1.3e-36	2.2e-04	159.4
Blood Pressure	PP	rs11969405	6	16289538	A	G	0.141	0.025	3.5e-08	4.1e-05	30.4
Blood Pressure	PP	rs12216497	6	19028623	T	C	0.131	0.021	2.3e-10	5.5e-05	40.3
Blood Pressure	PP	rs2744133	6	22392260	A	G	0.152	0.023	2.7e-11	6.0e-05	44.3
Blood Pressure	PP	rs1028885	6	25434518	A	G	-0.152	0.024	2.4e-10	5.4e-05	40.0
Blood Pressure	PP	rs1056314	6	26114653	A	G	0.176	0.020	9.3e-18	9.9e-05	73.4
Blood Pressure	PP	rs200997	6	27811815	A	G	0.197	0.022	1.5e-18	1.0e-04	77.1
Blood Pressure	PP	rs736466	6	29176296	T	C	-0.175	0.022	3.2e-15	9.5e-05	62.1
Blood Pressure	PP	rs3129077	6	29610435	T	C	0.123	0.022	1.4e-08	4.4e-05	32.3
Blood Pressure	PP	rs3132555	6	31082910	C	G	-0.186	0.023	1.9e-15	8.8e-05	63.0
Blood Pressure	PP	rs9267575	6	31811850	T	C	0.130	0.021	7.9e-10	5.2e-05	37.7
Blood Pressure	PP	rs3134950	6	32127477	A	C	0.293	0.022	8.2e-40	2.4e-04	174.0
Blood Pressure	PP	rs3176336	6	36648816	A	T	0.160	0.021	5.2e-14	7.7e-05	56.5
Blood Pressure	PP	rs9471008	6	39185482	A	G	-0.184	0.030	7.6e-10	5.1e-05	38.0
Blood Pressure	PP	rs1563788	6	43308363	T	C	0.212	0.023	5.8e-21	1.2e-04	87.9
Blood Pressure	PP	rs631441	6	53994626	T	G	-0.154	0.022	3.6e-12	6.5e-05	48.3
Blood Pressure	PP	rs12201429	6	55993585	T	C	0.376	0.030	1.5e-36	2.2e-04	159.5
Blood Pressure	PP	rs12195276	6	73657714	T	C	-0.182	0.023	3.5e-15	8.4e-05	62.0
Blood Pressure	PP	rs9361836	6	82235408	T	C	0.138	0.022	3.9e-10	5.3e-05	39.1
Blood Pressure	PP	rs60255247	6	85283253	A	C	0.249	0.033	4.5e-14	7.7e-05	57.0
Blood Pressure	PP	rs2983896	6	97029871	A	G	0.213	0.025	1.3e-17	9.9e-05	73.0
Blood Pressure	PP	rs72943207	6	99522316	A	G	0.144	0.026	2.3e-08	4.2e-05	31.1

Phenotype	Trait	SNP	Chr	Position (GRCh37/hg19)	EA	NEA	BETA	SE	P-value	R2	F
Blood Pressure	PP	rs9486916	6	109013930	T	C	0.184	0.026	1.8e-12	6.8e-05	49.8
Blood Pressure	PP	rs12192632	6	117525985	C	G	-0.122	0.021	7.6e-09	4.5e-05	33.3
Blood Pressure	PP	rs4946265	6	117863175	A	G	0.155	0.020	5.4e-14	7.8e-05	56.9
Blood Pressure	PP	rs12211121	6	121733676	A	T	-0.193	0.033	4.4e-09	4.7e-05	34.5
Blood Pressure	PP	rs1919872	6	122182186	A	G	0.347	0.035	1.8e-23	1.4e-04	99.9
Blood Pressure	PP	rs10782230	6	126228512	A	G	0.112	0.020	5.0e-08	4.0e-05	29.6
Blood Pressure	PP	rs13199674	6	127185489	A	G	0.220	0.021	1.7e-26	1.5e-04	113.4
Blood Pressure	PP	rs9885632	6	131311909	T	C	0.149	0.023	1.1e-10	5.6e-05	41.6
Blood Pressure	PP	rs4896104	6	135119089	T	C	-0.124	0.021	3.2e-09	4.8e-05	35.1
Blood Pressure	PP	rs7763294	6	140383733	T	G	-0.155	0.022	1.9e-12	6.7e-05	49.7
Blood Pressure	PP	rs6941056	6	143591821	C	G	0.201	0.021	3.6e-22	1.3e-04	94.1
Blood Pressure	PP	rs17080102	6	151004770	C	G	-0.320	0.040	1.9e-15	8.6e-05	63.1
Blood Pressure	PP	rs117524059	6	152099420	A	G	-0.226	0.039	8.9e-09	4.5e-05	33.1
Blood Pressure	PP	rs9340985	6	152341587	T	C	-0.476	0.033	4.2e-47	2.8e-04	208.3
Blood Pressure	PP	rs13206305	6	152549309	T	C	-0.156	0.026	1.8e-09	4.9e-05	36.3
Blood Pressure	PP	rs2102244	6	159724297	A	G	-0.380	0.030	4.2e-37	2.2e-04	161.9
Blood Pressure	PP	rs555754	6	160769423	A	G	-0.139	0.020	1.0e-11	6.2e-05	46.1
Blood Pressure	PP	rs9766666	6	169613541	T	G	-0.280	0.023	1.1e-33	2.0e-04	146.7
Blood Pressure	PP	rs9294987	6	169720237	T	C	-0.175	0.021	1.6e-16	9.6e-05	68.1
Blood Pressure	PP	rs2969036	7	2534901	T	G	0.131	0.023	1.4e-08	4.4e-05	32.3
Blood Pressure	PP	rs2107595	7	19049388	A	G	0.444	0.028	8.2e-56	3.3e-04	247.3
Blood Pressure	PP	rs1800795	7	22766645	C	G	0.187	0.021	2.6e-19	1.1e-04	80.9
Blood Pressure	PP	rs2529055	7	24590331	A	G	0.159	0.027	2.9e-09	4.8e-05	35.3
Blood Pressure	PP	rs2290263	7	25887278	A	G	0.174	0.024	3.4e-13	7.2e-05	52.8
Blood Pressure	PP	rs1859168	7	27242359	A	C	-0.432	0.039	2.0e-28	1.7e-04	122.0
Blood Pressure	PP	rs6961048	7	27328187	C	G	-0.267	0.034	2.7e-15	8.5e-05	62.3
Blood Pressure	PP	rs977184	7	28650761	T	C	-0.195	0.021	6.9e-20	1.1e-04	83.6
Blood Pressure	PP	rs2052263	7	36225818	A	G	0.198	0.033	1.4e-09	5.0e-05	36.8
Blood Pressure	PP	rs17171710	7	40440233	T	C	-0.366	0.034	7.4e-28	1.6e-04	119.4
Blood Pressure	PP	rs1004558	7	44240407	T	C	0.179	0.027	3.1e-11	6.0e-05	44.1
Blood Pressure	PP	rs11977526	7	46008110	A	G	-0.431	0.021	1.7e-92	5.7e-04	416.9
Blood Pressure	PP	rs1486141	7	46272315	C	G	-0.149	0.021	8.5e-13	7.0e-05	51.4
Blood Pressure	PP	rs12668436	7	47548893	T	C	-0.171	0.024	6.5e-13	7.0e-05	51.6
Blood Pressure	PP	rs1091811	7	73491212	A	G	-0.174	0.028	2.3e-10	5.5e-05	40.3
Blood Pressure	PP	rs848445	7	77572461	T	C	-0.131	0.023	1.2e-08	4.4e-05	32.4
Blood Pressure	PP	rs11770630	7	89805241	T	C	0.161	0.020	3.8e-15	8.4e-05	61.8
Blood Pressure	PP	rs4475412	7	90392848	C	G	0.133	0.022	7.6e-10	5.1e-05	37.8
Blood Pressure	PP	rs42377	7	92243672	A	G	-0.318	0.022	2.4e-45	2.7e-04	199.1
Blood Pressure	PP	rs12705090	7	100467700	T	C	-0.236	0.026	2.2e-19	1.1e-04	81.2
Blood Pressure	PP	rs12536419	7	106419296	A	C	-0.699	0.028	6.3e-133	8.2e-04	600.7
Blood Pressure	PP	rs1997571	7	116198621	A	G	-0.145	0.021	3.5e-12	6.6e-05	48.5
Blood Pressure	PP	rs11770163	7	116417848	C	G	0.120	0.022	2.9e-08	4.2e-05	30.7
Blood Pressure	PP	rs3757387	7	128576086	T	C	0.116	0.021	2.4e-08	4.2e-05	31.1
Blood Pressure	PP	rs13234269	7	130429186	A	T	-0.114	0.021	3.1e-08	4.1e-05	30.6
Blood Pressure	PP	rs35680304	7	130973495	T	C	0.185	0.021	1.6e-18	1.1e-04	77.4
Blood Pressure	PP	rs10267979	7	136618188	A	T	-0.132	0.022	1.6e-09	4.9e-05	36.5
Blood Pressure	PP	rs141212865	7	139404666	A	C	0.150	0.026	1.2e-08	4.4e-05	32.4
Blood Pressure	PP	rs73727605	7	149474622	A	G	0.285	0.042	1.5e-11	6.3e-05	45.5
Blood Pressure	PP	rs73158180	7	151402852	A	C	-0.169	0.023	1.8e-13	7.4e-05	54.2
Blood Pressure	PP	rs4716908	7	155561010	T	C	-0.155	0.026	2.9e-09	4.8e-05	35.2
Blood Pressure	PP	rs7015858	8	1724307	T	C	0.126	0.023	2.7e-08	4.2e-05	30.8
Blood Pressure	PP	rs907183	8	8729761	C	G	0.168	0.021	5.2e-16	8.9e-05	65.5
Blood Pressure	PP	rs6601523	8	10635141	A	G	-0.205	0.021	7.9e-23	1.3e-04	97.1
Blood Pressure	PP	rs4840581	8	11635569	T	C	0.159	0.021	1.8e-14	8.0e-05	58.7
Blood Pressure	PP	rs13279822	8	13291527	C	G	0.162	0.026	6.1e-10	5.2e-05	38.3
Blood Pressure	PP	rs7821832	8	25889446	T	G	0.214	0.024	1.5e-19	1.1e-04	82.0

Phenotype	Trait	SNP	Chr	Position (GRCh37/hg19)	EA	NEA	BETA	SE	P-value	R2	F
Blood Pressure	PP	rs62502006	8	30319467	A	G	-0.139	0.023	2.4e-09	4.9e-05	35.8
Blood Pressure	PP	rs11991469	8	32413280	C	G	-0.133	0.021	1.1e-10	5.6e-05	41.5
Blood Pressure	PP	rs7845722	8	33309993	A	G	-0.127	0.021	1.2e-09	5.0e-05	36.9
Blood Pressure	PP	rs10101096	8	38292147	A	C	-0.169	0.024	4.7e-12	6.5e-05	47.8
Blood Pressure	PP	rs2978456	8	42324765	T	C	-0.178	0.021	5.1e-17	9.6e-05	70.6
Blood Pressure	PP	rs4873492	8	51947549	T	C	0.220	0.027	8.1e-16	8.8e-05	65.1
Blood Pressure	PP	rs2354862	8	64501744	A	C	0.127	0.021	3.1e-09	4.8e-05	35.0
Blood Pressure	PP	rs10089500	8	74617061	A	G	-0.233	0.042	2.9e-08	4.2e-05	30.7
Blood Pressure	PP	rs117403674	8	75713707	C	G	-0.559	0.085	4.1e-11	6.0e-05	43.6
Blood Pressure	PP	rs1350100	8	76054904	A	G	0.165	0.021	1.8e-15	8.6e-05	63.2
Blood Pressure	PP	rs1449544	8	76591880	A	C	0.193	0.020	6.7e-21	1.2e-04	88.4
Blood Pressure	PP	rs6985531	8	77695388	T	C	0.244	0.029	1.7e-17	1.0e-04	72.7
Blood Pressure	PP	rs7009170	8	92149429	T	C	-0.201	0.022	5.7e-20	1.1e-04	83.5
Blood Pressure	PP	rs34917849	8	95278307	C	G	0.285	0.031	2.5e-20	1.2e-04	85.4
Blood Pressure	PP	rs13263821	8	105982209	C	G	-0.199	0.026	5.9e-14	7.6e-05	56.2
Blood Pressure	PP	rs28499085	8	110107161	A	G	0.157	0.023	9.4e-12	6.3e-05	46.5
Blood Pressure	PP	rs2205260	8	116959837	A	C	0.176	0.028	1.6e-10	5.5e-05	40.9
Blood Pressure	PP	rs7341594	8	120409477	A	G	0.507	0.025	5.6e-93	5.7e-04	417.8
Blood Pressure	PP	rs4909595	8	135671718	C	G	-0.128	0.023	4.8e-08	4.1e-05	29.8
Blood Pressure	PP	rs4440615	8	141057641	A	G	-0.249	0.021	8.2e-32	1.9e-04	138.2
Blood Pressure	PP	rs7011889	8	144005260	A	C	0.117	0.021	1.6e-08	4.3e-05	31.8
Blood Pressure	PP	rs7042232	9	14533251	A	G	-0.124	0.022	9.8e-09	4.4e-05	32.8
Blood Pressure	PP	rs4977492	9	19057551	T	C	-0.125	0.022	6.7e-09	4.5e-05	33.6
Blood Pressure	PP	rs4977575	9	22124744	C	G	-0.168	0.021	2.9e-16	8.9e-05	66.7
Blood Pressure	PP	rs4553000	9	34223553	T	C	-0.146	0.020	7.5e-13	6.9e-05	51.5
Blood Pressure	PP	rs113276784	9	35225357	A	G	0.132	0.024	3.4e-08	4.1e-05	30.5
Blood Pressure	PP	rs7047650	9	38075488	C	G	-0.126	0.021	1.8e-09	4.9e-05	36.2
Blood Pressure	PP	rs34587684	9	85076420	T	C	0.145	0.025	1.1e-08	4.4e-05	32.5
Blood Pressure	PP	rs7045409	9	95201540	A	T	-0.125	0.021	3.9e-09	4.7e-05	34.6
Blood Pressure	PP	rs10988442	9	101739709	A	G	0.181	0.021	1.4e-17	9.9e-05	72.8
Blood Pressure	PP	rs76038906	9	113250200	T	G	0.396	0.057	4.6e-12	6.4e-05	47.9
Blood Pressure	PP	rs13290326	9	116696625	T	C	-0.156	0.020	2.1e-14	7.9e-05	58.6
Blood Pressure	PP	rs3827676	9	116941019	T	C	0.131	0.021	4.1e-10	5.2e-05	39.0
Blood Pressure	PP	rs10982910	9	118534500	T	G	-0.201	0.035	1.1e-08	4.4e-05	32.6
Blood Pressure	PP	rs2241003	9	123666777	C	G	-0.172	0.021	4.0e-16	8.9e-05	66.1
Blood Pressure	PP	rs7854147	9	125863350	A	G	0.278	0.031	8.2e-19	1.1e-04	78.3
Blood Pressure	PP	rs142378207	9	127825168	A	G	0.347	0.030	3.5e-30	1.7e-04	130.5
Blood Pressure	PP	rs7869756	9	131210410	A	G	-0.152	0.027	1.1e-08	4.4e-05	32.6
Blood Pressure	PP	rs3780190	9	139099073	A	G	-0.141	0.021	1.4e-11	6.1e-05	45.7
Blood Pressure	PP	rs36006409	10	10876943	T	G	-0.141	0.025	3.4e-08	4.1e-05	30.5
Blood Pressure	PP	rs11257655	10	12307894	T	C	0.139	0.025	3.7e-08	4.1e-05	30.4
Blood Pressure	PP	rs1779240	10	18476313	A	G	-0.202	0.024	5.2e-17	9.5e-05	70.1
Blood Pressure	PP	rs12258967	10	18727959	C	G	0.279	0.023	3.7e-34	2.0e-04	148.0
Blood Pressure	PP	rs11010905	10	19934813	A	T	0.135	0.020	4.3e-11	5.9e-05	43.5
Blood Pressure	PP	rs56944355	10	20544580	T	C	0.164	0.025	4.2e-11	5.9e-05	43.6
Blood Pressure	PP	rs10732433	10	21037294	T	C	0.190	0.021	5.8e-20	1.1e-04	83.4
Blood Pressure	PP	rs11006778	10	28228037	A	G	-0.122	0.021	3.0e-09	4.8e-05	35.0
Blood Pressure	PP	rs9337951	10	30317073	A	G	0.258	0.023	4.2e-30	1.8e-04	129.5
Blood Pressure	PP	rs3006576	10	31244928	T	C	0.192	0.022	9.1e-18	1.0e-04	73.7
Blood Pressure	PP	rs211359	10	32316360	A	C	-0.188	0.024	3.3e-15	8.4e-05	62.1
Blood Pressure	PP	rs4948524	10	60351224	A	C	0.154	0.020	5.8e-14	7.7e-05	56.7
Blood Pressure	PP	rs7099368	10	61566323	T	C	0.142	0.021	1.2e-11	6.2e-05	46.0
Blood Pressure	PP	rs57946343	10	63499951	T	C	0.240	0.029	8.5e-17	9.4e-05	69.3
Blood Pressure	PP	rs6415872	10	63660689	A	G	0.122	0.021	3.7e-09	4.7e-05	34.8
Blood Pressure	PP	rs4746726	10	69820644	T	C	-0.170	0.022	4.0e-15	8.4e-05	61.5
Blood Pressure	PP	rs4746150	10	75565935	A	G	-0.217	0.032	1.6e-11	6.2e-05	45.4

Phenotype	Trait	SNP	Chr	Position (GRCh37/hg19)	EA	NEA	BETA	SE	P-value	R2	F
Blood Pressure	PP	rs55947600	10	75797672	A	G	-0.190	0.021	4.1e-20	1.2e-04	84.6
Blood Pressure	PP	rs10887914	10	82215288	T	C	0.146	0.020	1.1e-12	6.9e-05	50.8
Blood Pressure	PP	rs148994653	10	89615562	A	G	-0.148	0.025	5.1e-09	4.6e-05	34.0
Blood Pressure	PP	rs12240758	10	89807339	T	C	0.133	0.023	9.8e-09	4.5e-05	32.9
Blood Pressure	PP	rs111866816	10	94441507	T	C	0.255	0.041	3.1e-10	5.4e-05	39.5
Blood Pressure	PP	rs7070115	10	95900004	A	G	-0.256	0.021	4.6e-35	2.1e-04	152.1
Blood Pressure	PP	rs11187998	10	96278395	A	G	-0.140	0.021	1.1e-11	6.3e-05	46.4
Blood Pressure	PP	rs11190709	10	102552663	A	G	0.337	0.033	8.4e-25	1.4e-04	105.6
Blood Pressure	PP	rs11191156	10	103702763	A	G	-0.157	0.021	2.7e-13	7.3e-05	53.5
Blood Pressure	PP	rs138112129	10	104716767	A	T	0.401	0.062	1.3e-10	5.7e-05	41.2
Blood Pressure	PP	rs112913898	10	104958900	A	G	-0.582	0.038	5.7e-54	3.2e-04	239.2
Blood Pressure	PP	rs10883942	10	105651386	T	C	0.128	0.020	3.4e-10	5.4e-05	39.6
Blood Pressure	PP	rs10885409	10	114808072	T	C	-0.188	0.020	5.2e-20	1.1e-04	84.0
Blood Pressure	PP	rs10787515	10	115790005	T	C	-0.135	0.021	4.7e-11	5.9e-05	43.2
Blood Pressure	PP	rs72830615	10	122988575	A	G	0.179	0.021	1.6e-17	9.9e-05	72.3
Blood Pressure	PP	rs1133400	10	134459388	A	G	-0.161	0.025	3.0e-10	5.5e-05	39.8
Blood Pressure	PP	rs72844798	11	809666	A	G	-0.195	0.026	7.1e-14	7.8e-05	56.2
Blood Pressure	PP	rs686722	11	1891722	T	C	0.317	0.022	4.2e-47	2.9e-04	208.1
Blood Pressure	PP	rs74048200	11	2120298	A	G	-0.227	0.039	6.1e-09	4.7e-05	33.8
Blood Pressure	PP	rs143435810	11	9782346	A	G	-0.412	0.067	1.0e-09	5.1e-05	37.3
Blood Pressure	PP	rs138546576	11	10194152	A	G	0.308	0.039	2.2e-15	8.5e-05	63.0
Blood Pressure	PP	rs7131442	11	16348061	A	T	-0.213	0.025	3.0e-17	9.7e-05	71.6
Blood Pressure	PP	rs10832778	11	17394073	C	G	0.221	0.021	1.4e-25	1.5e-04	108.8
Blood Pressure	PP	rs10766533	11	19224677	A	T	0.126	0.023	4.1e-08	4.1e-05	30.0
Blood Pressure	PP	rs2582908	11	28522535	C	G	-0.116	0.020	1.5e-08	4.3e-05	32.0
Blood Pressure	PP	rs11031051	11	30355707	A	C	-0.172	0.022	1.0e-14	8.1e-05	60.0
Blood Pressure	PP	rs4922591	11	32374199	T	C	-0.151	0.021	1.4e-12	6.8e-05	50.0
Blood Pressure	PP	rs10838212	11	44019715	A	G	-0.224	0.036	7.5e-10	5.2e-05	38.0
Blood Pressure	PP	rs714417	11	45247176	T	C	-0.223	0.022	2.5e-23	1.4e-04	99.0
Blood Pressure	PP	rs7107356	11	47676170	A	G	-0.234	0.020	3.2e-30	1.8e-04	130.3
Blood Pressure	PP	rs9667596	11	48691193	T	C	0.274	0.030	3.1e-19	1.1e-04	80.5
Blood Pressure	PP	rs6486003	11	49693773	A	T	-0.239	0.030	3.9e-15	8.3e-05	61.6
Blood Pressure	PP	rs7127896	11	51519416	T	C	0.214	0.027	6.9e-15	8.3e-05	60.8
Blood Pressure	PP	rs11229252	11	55129640	T	C	-0.247	0.034	1.5e-13	7.4e-05	54.5
Blood Pressure	PP	rs4939043	11	56171183	A	C	-0.209	0.031	1.5e-11	6.2e-05	45.6
Blood Pressure	PP	rs11607056	11	57496820	T	C	-0.183	0.022	4.8e-17	9.5e-05	70.4
Blood Pressure	PP	rs174566	11	61592362	A	G	0.120	0.021	2.5e-08	4.2e-05	31.2
Blood Pressure	PP	rs4980515	11	63744609	T	C	0.163	0.020	2.3e-15	8.5e-05	63.1
Blood Pressure	PP	rs588177	11	64024056	A	C	-0.132	0.022	3.1e-09	4.8e-05	35.1
Blood Pressure	PP	rs144822931	11	65398943	T	C	0.509	0.080	1.7e-10	5.5e-05	40.8
Blood Pressure	PP	rs7119612	11	65570517	T	C	-0.246	0.035	3.6e-12	6.6e-05	48.3
Blood Pressure	PP	rs7395791	11	69262916	A	G	-0.129	0.021	6.4e-10	5.2e-05	38.3
Blood Pressure	PP	rs72931748	11	69825414	A	G	0.269	0.036	8.4e-14	7.6e-05	55.8
Blood Pressure	PP	rs1687692	11	76512416	A	G	0.159	0.026	8.6e-10	5.1e-05	37.6
Blood Pressure	PP	rs2289125	11	89224453	A	C	-0.385	0.025	1.8e-51	3.1e-04	227.6
Blood Pressure	PP	rs10830963	11	92708710	C	G	-0.136	0.024	7.1e-09	4.5e-05	33.4
Blood Pressure	PP	rs11021221	11	95308854	A	T	0.181	0.028	4.8e-11	5.8e-05	43.1
Blood Pressure	PP	rs604723	11	100610546	T	C	-0.269	0.023	1.5e-31	1.9e-04	136.7
Blood Pressure	PP	rs12807220	11	102077200	A	G	-0.148	0.021	6.5e-12	6.4e-05	47.2
Blood Pressure	PP	rs4754196	11	107096777	A	G	-0.202	0.021	9.3e-23	1.3e-04	96.3
Blood Pressure	PP	rs11214503	11	112986754	T	C	0.113	0.021	3.6e-08	4.1e-05	30.2
Blood Pressure	PP	rs641620	11	117074229	T	C	-0.169	0.030	1.5e-08	4.4e-05	32.1
Blood Pressure	PP	rs573455	11	117267884	A	G	0.252	0.021	2.4e-34	2.0e-04	149.1
Blood Pressure	PP	rs78799967	11	118266523	T	C	-0.470	0.069	9.4e-12	6.4e-05	46.4
Blood Pressure	PP	rs11222084	11	130273230	A	T	-0.497	0.021	5.2e-119	7.3e-04	539.8
Blood Pressure	PP	rs10736585	11	130465785	T	C	0.194	0.022	2.8e-19	1.1e-04	80.3

Phenotype	Trait	SNP	Chr	Position (GRCh37/hg19)	EA	NEA	BETA	SE	P-value	R2	F
Blood Pressure	PP	rs11603014	11	130730825	A	G	0.173	0.026	1.9e-11	6.1e-05	45.1
Blood Pressure	PP	rs486098	12	388657	T	C	0.154	0.023	3.7e-11	6.0e-05	43.8
Blood Pressure	PP	rs3819532	12	2436837	T	C	-0.134	0.021	1.5e-10	5.5e-05	40.9
Blood Pressure	PP	rs117233107	12	4328521	A	G	-0.533	0.093	1.1e-08	4.4e-05	32.6
Blood Pressure	PP	rs11609905	12	12656760	T	C	-0.182	0.023	2.3e-15	8.4e-05	63.0
Blood Pressure	PP	rs4763297	12	12889289	A	C	-0.147	0.021	3.1e-12	6.6e-05	48.8
Blood Pressure	PP	rs7313556	12	15297359	A	G	-0.140	0.021	6.8e-11	5.7e-05	42.6
Blood Pressure	PP	rs10841389	12	20017599	A	G	-0.155	0.025	6.7e-10	5.1e-05	38.2
Blood Pressure	PP	rs10770612	12	20230639	A	G	0.342	0.026	5.8e-40	2.3e-04	174.5
Blood Pressure	PP	rs34085523	12	20760410	A	G	0.120	0.021	7.0e-09	4.5e-05	33.4
Blood Pressure	PP	rs704191	12	22015022	T	C	0.163	0.021	2.7e-15	8.4e-05	62.5
Blood Pressure	PP	rs17287293	12	24770878	A	G	-0.197	0.029	6.0e-12	6.4e-05	47.4
Blood Pressure	PP	rs10842991	12	27962103	T	C	-0.183	0.026	1.9e-12	6.6e-05	49.5
Blood Pressure	PP	rs11052722	12	33626530	A	G	0.113	0.021	4.7e-08	4.1e-05	29.9
Blood Pressure	PP	rs2261608	12	48721634	A	T	0.170	0.021	1.4e-15	8.6e-05	64.0
Blood Pressure	PP	rs150857355	12	49209340	C	G	0.527	0.076	5.5e-12	6.5e-05	47.5
Blood Pressure	PP	rs117945311	12	50015907	A	T	0.505	0.077	4.8e-11	5.9e-05	43.3
Blood Pressure	PP	rs7315980	12	53449321	A	G	0.249	0.038	5.8e-11	5.8e-05	43.0
Blood Pressure	PP	rs67772913	12	54435716	A	G	0.231	0.022	1.1e-24	1.4e-04	105.1
Blood Pressure	PP	rs7968719	12	57540751	C	G	-0.120	0.021	2.2e-08	4.2e-05	31.4
Blood Pressure	PP	rs1351394	12	66351826	T	C	-0.181	0.020	6.5e-19	1.1e-04	78.8
Blood Pressure	PP	rs4842266	12	79951566	A	G	-0.168	0.022	4.9e-14	7.8e-05	56.9
Blood Pressure	PP	rs111478946	12	90058842	A	G	-0.462	0.028	8.2e-63	3.8e-04	280.6
Blood Pressure	PP	rs10859568	12	94130967	A	T	0.142	0.021	6.7e-12	6.3e-05	46.9
Blood Pressure	PP	rs114697502	12	94677559	T	C	-0.381	0.038	5.8e-24	1.4e-04	101.8
Blood Pressure	PP	rs7977311	12	95487226	T	C	-0.199	0.032	4.9e-10	5.2e-05	38.7
Blood Pressure	PP	rs1520222	12	102797293	A	G	0.128	0.023	2.9e-08	4.1e-05	30.7
Blood Pressure	PP	rs7956131	12	110340053	A	G	0.229	0.039	4.7e-09	4.6e-05	34.2
Blood Pressure	PP	rs11065861	12	111752211	A	G	0.168	0.025	1.4e-11	6.2e-05	45.7
Blood Pressure	PP	rs35429	12	115555867	A	G	0.178	0.021	4.9e-17	9.4e-05	70.3
Blood Pressure	PP	rs6490019	12	115920472	A	G	-0.115	0.021	4.8e-08	4.0e-05	29.9
Blood Pressure	PP	rs61935847	12	116630136	T	G	-0.156	0.024	5.1e-11	5.8e-05	43.0
Blood Pressure	PP	rs606950	13	22298923	A	G	0.136	0.021	1.5e-10	5.5e-05	40.9
Blood Pressure	PP	rs7338758	13	30137828	T	C	0.158	0.024	5.5e-11	5.8e-05	43.1
Blood Pressure	PP	rs9525471	13	41886568	A	G	-0.144	0.024	3.5e-09	4.7e-05	34.9
Blood Pressure	PP	rs7491248	13	47180671	A	G	0.154	0.025	3.9e-10	5.3e-05	39.1
Blood Pressure	PP	rs4304924	13	79238925	A	G	-0.120	0.021	8.7e-09	4.5e-05	33.2
Blood Pressure	PP	rs4287430	13	94417872	A	T	-0.114	0.021	4.6e-08	4.0e-05	29.9
Blood Pressure	PP	rs3742182	13	111375132	T	C	-0.186	0.026	9.0e-13	6.8e-05	50.9
Blood Pressure	PP	rs9549328	13	113636156	T	C	0.216	0.025	1.8e-18	1.0e-04	76.8
Blood Pressure	PP	rs7331680	13	115000650	T	G	0.228	0.029	2.3e-15	8.5e-05	62.9
Blood Pressure	PP	rs365990	14	23861811	A	G	0.308	0.021	1.8e-47	2.8e-04	209.0
Blood Pressure	PP	rs696	14	35871093	T	C	0.211	0.021	7.5e-23	1.3e-04	96.9
Blood Pressure	PP	rs34983854	14	39858442	A	G	-0.137	0.021	5.6e-11	5.8e-05	43.0
Blood Pressure	PP	rs142004400	14	50829560	A	C	0.331	0.057	5.2e-09	4.6e-05	34.1
Blood Pressure	PP	rs8009633	14	53386836	C	G	0.207	0.024	2.4e-17	9.6e-05	71.5
Blood Pressure	PP	rs9323466	14	66278992	A	T	-0.120	0.021	6.9e-09	4.5e-05	33.7
Blood Pressure	PP	rs8003103	14	71451265	A	G	-0.123	0.022	1.4e-08	4.3e-05	32.1
Blood Pressure	PP	rs2215590	14	73297741	T	C	0.173	0.024	1.7e-13	7.3e-05	54.3
Blood Pressure	PP	rs11159091	14	75074316	A	G	0.129	0.021	4.2e-10	5.3e-05	39.1
Blood Pressure	PP	rs11627326	14	85785251	C	G	0.155	0.023	9.8e-12	6.2e-05	46.4
Blood Pressure	PP	rs4904503	14	89565130	T	C	0.150	0.022	2.5e-11	6.0e-05	44.4
Blood Pressure	PP	rs8023114	14	92391955	A	G	-0.156	0.022	5.1e-13	7.0e-05	52.3
Blood Pressure	PP	rs11160085	14	93112102	T	C	0.140	0.023	8.6e-10	5.0e-05	37.6
Blood Pressure	PP	rs8013933	14	94465789	T	C	0.147	0.022	3.4e-11	5.9e-05	44.1
Blood Pressure	PP	rs7154431	14	98590709	A	T	-0.200	0.021	1.8e-21	1.2e-04	90.8

Phenotype	Trait	SNP	Chr	Position (GRCh37/hg19)	EA	NEA	BETA	SE	P-value	R2	F
Blood Pressure	PP	rs9324011	14	100136575	A	C	-0.150	0.021	1.8e-12	6.7e-05	49.7
Blood Pressure	PP	rs11160546	14	100233353	T	C	0.192	0.022	6.7e-19	1.1e-04	79.1
Blood Pressure	PP	rs11626434	14	101998443	C	G	-0.134	0.022	7.9e-10	5.2e-05	37.7
Blood Pressure	PP	rs8017780	14	103987305	A	C	0.162	0.025	1.1e-10	5.6e-05	41.7
Blood Pressure	PP	rs11629850	15	40317075	A	G	0.118	0.020	9.7e-09	4.4e-05	33.0
Blood Pressure	PP	rs7178506	15	41096097	T	C	-0.123	0.022	1.3e-08	4.3e-05	32.4
Blood Pressure	PP	rs2015637	15	48716853	T	C	0.501	0.034	8.9e-48	2.8e-04	211.0
Blood Pressure	PP	rs3098186	15	50810621	T	C	-0.174	0.021	4.4e-17	9.4e-05	70.3
Blood Pressure	PP	rs17271730	15	62757722	A	G	0.163	0.021	2.1e-14	7.9e-05	58.6
Blood Pressure	PP	rs55962736	15	63333892	T	G	-0.156	0.020	2.5e-14	7.8e-05	58.1
Blood Pressure	PP	rs832890	15	65166309	T	C	0.129	0.021	3.3e-10	5.3e-05	39.3
Blood Pressure	PP	rs2289261	15	67457485	C	G	-0.148	0.022	5.6e-12	6.3e-05	47.3
Blood Pressure	PP	rs4337253	15	71609306	C	G	0.150	0.022	3.9e-12	6.5e-05	48.2
Blood Pressure	PP	rs8042039	15	74225518	T	C	0.163	0.028	8.2e-09	4.5e-05	33.3
Blood Pressure	PP	rs9210	15	75128501	T	C	0.160	0.023	3.0e-12	6.6e-05	48.9
Blood Pressure	PP	rs61157089	15	79155412	T	C	-0.258	0.029	2.2e-19	1.1e-04	80.9
Blood Pressure	PP	rs11634851	15	81028965	C	G	-0.186	0.021	1.6e-19	1.1e-04	81.3
Blood Pressure	PP	rs17158080	15	83798496	T	C	0.128	0.022	1.4e-08	4.3e-05	32.2
Blood Pressure	PP	rs3743157	15	85680532	A	C	0.167	0.028	1.3e-09	5.0e-05	36.9
Blood Pressure	PP	rs8024518	15	85961551	A	G	-0.191	0.030	2.2e-10	5.4e-05	40.3
Blood Pressure	PP	rs28547445	15	89576961	T	C	0.271	0.040	8.8e-12	6.7e-05	46.5
Blood Pressure	PP	rs28611491	15	90641809	T	C	0.254	0.039	9.5e-11	5.6e-05	41.9
Blood Pressure	PP	rs7497304	15	91429176	T	G	0.282	0.022	1.4e-36	2.2e-04	160.0
Blood Pressure	PP	rs11074093	15	93222897	T	C	-0.132	0.021	3.3e-10	5.3e-05	39.4
Blood Pressure	PP	rs11632112	15	93468276	C	G	-0.164	0.024	8.7e-12	6.3e-05	46.6
Blood Pressure	PP	rs11248862	16	1344291	A	G	0.228	0.032	4.7e-13	7.1e-05	52.5
Blood Pressure	PP	rs17694688	16	4349011	A	G	-0.187	0.029	2.2e-10	5.6e-05	40.2
Blood Pressure	PP	rs6500601	16	4497199	C	G	-0.160	0.024	1.5e-11	6.1e-05	45.4
Blood Pressure	PP	rs10500326	16	4918326	T	G	-0.150	0.024	4.4e-10	5.2e-05	38.9
Blood Pressure	PP	rs30229	16	14404222	T	G	-0.126	0.021	2.8e-09	4.8e-05	35.5
Blood Pressure	PP	rs3915425	16	15912544	T	C	0.191	0.022	4.0e-18	1.0e-04	75.6
Blood Pressure	PP	rs200528	16	24759131	A	G	-0.230	0.026	6.4e-19	1.1e-04	79.1
Blood Pressure	PP	rs55861648	16	30833896	A	G	0.157	0.025	7.3e-10	5.1e-05	38.1
Blood Pressure	PP	rs34941092	16	50550137	A	G	-0.169	0.029	5.8e-09	4.6e-05	34.0
Blood Pressure	PP	rs9937815	16	56330832	A	G	-0.139	0.022	2.9e-10	5.3e-05	39.7
Blood Pressure	PP	rs37060	16	58566304	A	G	0.149	0.024	3.1e-10	5.3e-05	39.5
Blood Pressure	PP	rs28633979	16	65282820	A	C	-0.200	0.021	2.5e-22	1.3e-04	94.3
Blood Pressure	PP	rs12149704	16	69789516	A	G	0.748	0.047	5.5e-58	3.5e-04	257.4
Blood Pressure	PP	rs62048010	16	70803755	T	C	-0.572	0.044	5.3e-38	2.2e-04	165.9
Blood Pressure	PP	rs62053796	16	71966463	A	G	0.550	0.049	2.4e-29	1.7e-04	126.6
Blood Pressure	PP	rs6416749	16	73100308	T	C	0.180	0.023	7.2e-15	8.3e-05	60.7
Blood Pressure	PP	rs4888408	16	75432824	A	G	0.250	0.021	1.0e-32	1.9e-04	142.5
Blood Pressure	PP	rs56844452	16	80864776	T	C	-0.329	0.041	5.4e-16	8.8e-05	65.6
Blood Pressure	PP	rs7500448	16	83045790	A	G	0.359	0.024	3.6e-51	3.1e-04	225.5
Blood Pressure	PP	rs28651151	16	83413352	T	G	0.145	0.023	3.6e-10	5.3e-05	39.3
Blood Pressure	PP	rs3950627	16	86436343	A	C	0.117	0.021	2.6e-08	4.2e-05	30.8
Blood Pressure	PP	rs7499959	16	88110422	C	G	-0.141	0.023	1.5e-09	4.9e-05	36.7
Blood Pressure	PP	rs35613341	16	88535407	C	G	0.127	0.023	2.5e-08	4.3e-05	31.1
Blood Pressure	PP	rs2434865	16	89609572	C	G	-0.177	0.026	5.7e-12	6.4e-05	47.4
Blood Pressure	PP	rs12948875	17	1347649	A	C	-0.182	0.023	1.5e-15	8.5e-05	63.4
Blood Pressure	PP	rs4480845	17	1958609	T	C	0.214	0.022	3.1e-23	1.3e-04	98.2
Blood Pressure	PP	rs4796514	17	6475090	T	C	-0.231	0.021	4.1e-28	1.6e-04	121.3
Blood Pressure	PP	rs2074216	17	7133609	A	G	0.132	0.022	1.1e-09	5.0e-05	36.9
Blood Pressure	PP	rs78378222	17	7571752	T	G	1.049	0.094	1.3e-28	1.7e-04	123.2
Blood Pressure	PP	rs62062618	17	7658587	A	G	-0.275	0.033	1.2e-16	9.2e-05	68.8
Blood Pressure	PP	rs138285687	17	27195674	T	C	-0.330	0.052	1.7e-10	5.5e-05	40.8

Phenotype	Trait	SNP	Chr	Position (GRCh37/hg19)	EA	NEA	BETA	SE	P-value	R2	F
Blood Pressure	PP	rs9900089	17	30772577	T	C	-0.126	0.021	1.7e-09	4.9e-05	36.2
Blood Pressure	PP	rs12150495	17	40388430	A	G	0.358	0.041	2.1e-18	1.1e-04	76.6
Blood Pressure	PP	rs324075	17	41026523	A	G	0.201	0.028	3.5e-13	7.2e-05	53.0
Blood Pressure	PP	rs150055076	17	42009211	A	G	0.178	0.027	5.3e-11	5.8e-05	43.1
Blood Pressure	PP	rs12603813	17	43196584	T	C	-0.268	0.024	8.3e-30	1.7e-04	128.2
Blood Pressure	PP	rs17608766	17	45013271	T	C	-0.527	0.029	2.1e-71	4.3e-04	319.6
Blood Pressure	PP	rs62065796	17	46508156	A	G	0.589	0.055	1.4e-26	1.5e-04	113.9
Blood Pressure	PP	rs9221	17	46675813	T	C	0.282	0.036	1.2e-14	8.0e-05	59.6
Blood Pressure	PP	rs80080721	17	47777540	T	G	-0.179	0.031	9.9e-09	4.4e-05	33.0
Blood Pressure	PP	rs12325702	17	55446364	A	T	0.147	0.021	2.2e-12	6.6e-05	49.5
Blood Pressure	PP	rs2645490	17	57878509	A	G	0.160	0.025	1.5e-10	5.6e-05	41.0
Blood Pressure	PP	rs2109019	17	59475888	A	C	-0.246	0.026	5.2e-22	1.3e-04	92.6
Blood Pressure	PP	rs56288724	17	60767135	A	G	-0.238	0.021	2.0e-29	1.7e-04	126.9
Blood Pressure	PP	rs1991401	17	62502435	A	G	0.179	0.023	2.0e-15	8.6e-05	62.9
Blood Pressure	PP	rs112699970	17	64257958	A	G	-0.341	0.060	1.2e-08	4.4e-05	32.6
Blood Pressure	PP	rs35504735	17	74686809	A	G	0.117	0.020	1.4e-08	4.3e-05	32.4
Blood Pressure	PP	rs34587622	17	75398498	T	C	-0.208	0.035	2.5e-09	4.8e-05	35.5
Blood Pressure	PP	rs9302885	17	76799898	A	G	0.121	0.021	4.1e-09	4.6e-05	34.4
Blood Pressure	PP	rs9901265	17	79415766	A	G	-0.127	0.023	2.1e-08	4.2e-05	31.4
Blood Pressure	PP	rs34413141	18	777282	A	T	-0.162	0.027	1.4e-09	4.9e-05	36.8
Blood Pressure	PP	rs929581	18	20069699	T	C	0.132	0.021	5.7e-10	5.1e-05	38.3
Blood Pressure	PP	rs61735998	18	34289285	T	G	0.471	0.070	2.0e-11	6.1e-05	45.1
Blood Pressure	PP	rs9949727	18	42088422	A	G	-0.237	0.022	1.4e-27	1.6e-04	118.6
Blood Pressure	PP	rs11874246	18	42596789	T	C	0.157	0.022	1.9e-12	6.6e-05	49.4
Blood Pressure	PP	rs7236548	18	43097750	A	C	0.362	0.026	8.5e-43	2.5e-04	188.1
Blood Pressure	PP	rs11872627	18	48287818	T	C	-0.253	0.030	1.8e-17	9.7e-05	72.2
Blood Pressure	PP	rs10048404	18	54578482	T	C	-0.152	0.022	2.0e-12	6.6e-05	49.3
Blood Pressure	PP	rs663640	18	57846077	T	C	-0.155	0.025	5.9e-10	5.1e-05	38.3
Blood Pressure	PP	rs12172847	18	60223017	A	G	-0.128	0.022	4.7e-09	4.6e-05	34.4
Blood Pressure	PP	rs1047922	18	74070562	T	C	-0.212	0.030	9.7e-13	6.9e-05	51.0
Blood Pressure	PP	rs7250835	19	670234	T	C	0.182	0.029	4.4e-10	5.4e-05	39.0
Blood Pressure	PP	rs3760994	19	1435771	A	G	-0.144	0.022	4.3e-11	6.0e-05	43.6
Blood Pressure	PP	rs8102624	19	2161443	A	G	0.557	0.039	1.1e-45	2.7e-04	201.1
Blood Pressure	PP	rs35502362	19	4966041	T	C	-0.151	0.022	3.9e-12	6.5e-05	48.1
Blood Pressure	PP	rs10420008	19	7234575	A	G	-0.186	0.024	1.8e-14	7.9e-05	58.9
Blood Pressure	PP	rs12978472	19	7257990	C	G	0.376	0.033	1.5e-29	1.8e-04	127.2
Blood Pressure	PP	rs10409243	19	10332988	T	C	-0.192	0.021	2.8e-19	1.1e-04	80.2
Blood Pressure	PP	rs167479	19	11526765	T	G	-0.213	0.022	8.0e-22	1.4e-04	92.1
Blood Pressure	PP	rs7408868	19	15285679	C	G	0.291	0.034	1.3e-17	9.8e-05	73.1
Blood Pressure	PP	rs8111708	19	18558876	A	G	0.152	0.022	2.2e-12	6.6e-05	49.2
Blood Pressure	PP	rs28572357	19	31867447	A	C	-0.150	0.021	6.8e-13	7.0e-05	51.8
Blood Pressure	PP	rs7256564	19	33889593	A	G	0.128	0.022	7.2e-09	4.5e-05	33.4
Blood Pressure	PP	rs2303040	19	39138608	T	C	-0.123	0.021	2.8e-09	4.8e-05	35.5
Blood Pressure	PP	rs117557920	19	41078591	A	G	0.236	0.028	9.0e-18	9.9e-05	73.8
Blood Pressure	PP	rs1800470	19	41858921	A	G	-0.150	0.021	1.8e-12	6.7e-05	49.6
Blood Pressure	PP	rs7412	19	45412079	T	C	-0.377	0.039	5.7e-22	1.3e-04	92.9
Blood Pressure	PP	rs17571725	19	46290822	T	C	-0.155	0.021	4.4e-13	7.0e-05	52.3
Blood Pressure	PP	rs73046792	19	49605705	A	G	-0.211	0.029	3.4e-13	7.2e-05	53.0
Blood Pressure	PP	rs2143635	20	2793063	T	C	-0.230	0.035	4.8e-11	5.8e-05	43.3
Blood Pressure	PP	rs11087740	20	6657554	T	C	-0.162	0.021	3.4e-15	8.4e-05	62.1
Blood Pressure	PP	rs6056341	20	9004830	C	G	-0.121	0.021	3.8e-09	4.7e-05	34.7
Blood Pressure	PP	rs2143618	20	10614420	A	G	-0.221	0.028	1.4e-15	8.5e-05	63.7
Blood Pressure	PP	rs2206815	20	10669188	A	C	-0.361	0.021	4.3e-68	4.1e-04	304.0
Blood Pressure	PP	rs6078000	20	10977631	A	G	-0.195	0.023	8.0e-18	9.9e-05	74.1
Blood Pressure	PP	rs6132154	20	19047786	C	G	-0.317	0.050	1.5e-10	5.5e-05	41.1
Blood Pressure	PP	rs6035355	20	19465089	A	T	0.289	0.023	1.2e-36	2.2e-04	160.4

Phenotype	Trait	SNP	Chr	Position (GRCh37/hg19)	EA	NEA	BETA	SE	P-value	R2	F
Blood Pressure	PP	rs6141767	20	31225069	C	G	0.215	0.029	5.6e-14	7.6e-05	56.4
Blood Pressure	PP	rs6060355	20	33890061	A	G	0.142	0.021	2.0e-11	6.1e-05	45.1
Blood Pressure	PP	rs4811601	20	36849007	T	C	0.130	0.021	4.8e-10	5.2e-05	38.6
Blood Pressure	PP	rs4812536	20	40258709	A	G	0.122	0.022	2.8e-08	4.1e-05	30.8
Blood Pressure	PP	rs6031431	20	42795152	A	G	-0.158	0.021	2.7e-14	7.8e-05	57.9
Blood Pressure	PP	rs11906149	20	57744869	C	G	0.228	0.032	7.7e-13	6.9e-05	51.2
Blood Pressure	PP	rs8118848	20	62461572	A	G	-0.188	0.025	4.4e-14	7.8e-05	57.1
Blood Pressure	PP	rs2229742	21	16339172	C	G	0.221	0.034	8.6e-11	5.7e-05	42.1
Blood Pressure	PP	rs11909120	21	30131872	A	T	-0.198	0.029	1.2e-11	6.2e-05	45.9
Blood Pressure	PP	rs9976596	21	35596842	T	C	0.200	0.029	4.0e-12	6.4e-05	48.0
Blood Pressure	PP	rs112005532	21	39957981	T	C	-0.497	0.057	4.7e-18	1.0e-04	74.9
Blood Pressure	PP	rs2277788	21	40817702	C	G	0.185	0.033	3.0e-08	4.1e-05	30.6
Blood Pressure	PP	rs12627651	21	44760603	A	G	0.134	0.023	8.2e-09	4.5e-05	33.4
Blood Pressure	PP	rs35796750	21	47422412	T	C	-0.125	0.021	2.1e-09	4.8e-05	35.9
Blood Pressure	PP	rs11701512	21	47962811	A	G	0.167	0.027	3.7e-10	5.3e-05	39.3
Blood Pressure	PP	rs4819852	22	19988167	A	G	0.249	0.023	8.7e-28	1.6e-04	118.9
Blood Pressure	PP	rs62235149	22	22196532	A	G	0.124	0.021	5.4e-09	4.6e-05	34.0
Blood Pressure	PP	rs134092	22	28068444	A	T	-0.118	0.021	1.2e-08	4.3e-05	32.3
Blood Pressure	PP	rs16986825	22	29300306	T	C	0.175	0.029	8.8e-10	5.1e-05	37.5
Blood Pressure	PP	rs5753103	22	30768777	A	G	0.138	0.021	2.6e-11	6.1e-05	44.7
Blood Pressure	PP	rs5750482	22	38117943	T	C	0.121	0.021	1.1e-08	4.4e-05	32.7
Blood Pressure	PP	rs139919	22	40726183	T	C	-0.248	0.027	8.0e-20	1.1e-04	82.9
Blood Pressure	PP	rs73161324	22	42038786	T	C	0.311	0.048	7.5e-11	5.8e-05	42.4
Blood Pressure	PP	rs6007594	22	45728370	A	G	0.140	0.023	2.0e-09	4.9e-05	36.0
Blood Pressure	PP	rs148140538	22	50228044	T	C	-0.220	0.038	8.5e-09	4.5e-05	33.1

Phenotype	Trait	SNP	Chr	Position (GRCh37/hg19)	EA	NEA	BETA	SE	P-value	R2	F
Lipid Metabolism	HDL	rs2340596	1	913889	A	G	-0.011	0.002	1.4e-12	4.5e-05	50.1
Lipid Metabolism	HDL	rs55775305	1	2975119	G	C	-0.013	0.002	1.3e-11	3.7e-05	45.8
Lipid Metabolism	HDL	rs6691831	1	11220180	G	A	0.010	0.002	4.3e-09	2.9e-05	34.5
Lipid Metabolism	HDL	rs602950	1	20915531	G	A	-0.010	0.001	1.1e-12	4.1e-05	50.6
Lipid Metabolism	HDL	rs4077296	1	22273648	T	G	-0.009	0.001	2.4e-10	3.2e-05	40.1
Lipid Metabolism	HDL	rs1077514	1	23766233	T	C	0.016	0.002	1.0e-16	5.5e-05	69.0
Lipid Metabolism	HDL	rs75460349	1	27180088	C	A	-0.087	0.004	4.2e-84	3.0e-04	377.6
Lipid Metabolism	HDL	rs57215361	1	27314814	G	A	-0.021	0.003	2.2e-12	4.0e-05	49.3
Lipid Metabolism	HDL	rs2815708	1	28347491	T	C	-0.013	0.001	1.6e-17	5.9e-05	72.6
Lipid Metabolism	HDL	rs61780506	1	31531469	C	A	-0.015	0.003	3.1e-08	2.5e-05	30.6
Lipid Metabolism	HDL	rs9787203	1	39288581	A	T	-0.013	0.002	4.5e-09	2.8e-05	34.4
Lipid Metabolism	HDL	rs182329221	1	39716848	C	T	-0.077	0.010	3.0e-14	6.6e-05	57.8
Lipid Metabolism	HDL	rs3768321	1	40035928	T	G	-0.044	0.002	3.8e-138	5.0e-04	625.9
Lipid Metabolism	HDL	rs114874962	1	40119293	A	G	0.027	0.004	1.3e-10	3.3e-05	41.3
Lipid Metabolism	HDL	rs61779839	1	40324111	C	T	-0.013	0.002	2.6e-15	5.0e-05	62.6
Lipid Metabolism	HDL	rs6703709	1	42872725	T	C	-0.008	0.001	5.4e-09	2.7e-05	34.0
Lipid Metabolism	HDL	rs9437006	1	56983656	T	C	-0.015	0.002	2.2e-12	4.0e-05	49.3
Lipid Metabolism	HDL	rs142629567	1	61685540	G	C	0.016	0.003	4.1e-10	3.1e-05	39.0
Lipid Metabolism	HDL	rs11208004	1	63145439	A	G	-0.012	0.001	1.8e-16	5.5e-05	67.9
Lipid Metabolism	HDL	rs185873223	1	63426178	C	T	-0.908	0.156	6.4e-09	8.8e-05	33.7
Lipid Metabolism	HDL	rs4655582	1	66153363	G	C	0.010	0.001	6.0e-13	4.2e-05	51.9
Lipid Metabolism	HDL	rs594454	1	71430468	T	G	0.011	0.001	5.9e-14	4.5e-05	56.4
Lipid Metabolism	HDL	rs2613498	1	72752939	T	C	0.014	0.002	4.4e-15	4.9e-05	61.5
Lipid Metabolism	HDL	rs79389962	1	79375401	G	A	-0.011	0.002	1.3e-08	2.6e-05	32.3
Lipid Metabolism	HDL	rs2207368	1	85847683	G	A	0.008	0.001	1.0e-08	2.6e-05	32.7
Lipid Metabolism	HDL	rs185730318	1	89079489	A	T	-0.035	0.006	2.2e-08	2.5e-05	31.3
Lipid Metabolism	HDL	rs4847412	1	93829248	G	C	-0.026	0.001	2.3e-71	2.6e-04	319.1
Lipid Metabolism	HDL	rs148654281	1	94085147	G	A	0.025	0.005	3.1e-08	2.5e-05	30.7

Phenotype	Trait	SNP	Chr	Position (GRCh37/hg19)	EA	NEA	BETA	SE	P-value	R2	F
Lipid Metabolism	HDL	rs2878349	1	107549245	A	G	-0.009	0.001	7.0e-10	3.1e-05	38.0
Lipid Metabolism	HDL	rs12740374	1	109817590	T	G	0.035	0.002	1.2e-97	3.5e-04	439.8
Lipid Metabolism	HDL	rs36209093	1	110229787	T	C	0.032	0.002	1.4e-39	2.7e-04	173.2
Lipid Metabolism	HDL	rs59688746	1	110445511	A	T	0.019	0.003	2.6e-10	3.2e-05	40.0
Lipid Metabolism	HDL	rs9429767	1	110496087	A	G	-0.022	0.002	1.1e-35	1.3e-04	155.5
Lipid Metabolism	HDL	rs197393	1	112298829	C	T	-0.008	0.001	3.2e-08	2.5e-05	30.6
Lipid Metabolism	HDL	rs186147970	1	120484718	C	T	0.092	0.013	1.8e-12	4.6e-05	49.7
Lipid Metabolism	HDL	rs181473381	1	145472741	A	C	0.141	0.013	8.2e-27	9.6e-05	114.9
Lipid Metabolism	HDL	rs71618972	1	145827097	G	T	0.284	0.047	1.6e-09	8.7e-05	36.4
Lipid Metabolism	HDL	rs587672025	1	145970635	A	T	0.328	0.040	1.5e-16	1.8e-04	68.1
Lipid Metabolism	HDL	rs138030384	1	147345231	C	T	0.117	0.020	6.5e-09	4.0e-05	33.7
Lipid Metabolism	HDL	rs16835135	1	149880483	G	A	0.023	0.003	2.5e-19	6.5e-05	80.8
Lipid Metabolism	HDL	rs72992015	1	150842696	T	C	0.023	0.002	3.8e-39	1.4e-04	171.3
Lipid Metabolism	HDL	rs145904381	1	151017991	C	T	0.051	0.006	2.3e-15	5.1e-05	62.8
Lipid Metabolism	HDL	rs587655553	1	151090532	A	G	0.472	0.085	3.2e-08	7.5e-05	30.6
Lipid Metabolism	HDL	rs59603391	1	151124313	T	G	0.015	0.003	1.7e-09	2.9e-05	36.4
Lipid Metabolism	HDL	rs12028554	1	154223101	A	G	-0.008	0.001	1.2e-08	2.6e-05	32.5
Lipid Metabolism	HDL	rs9426828	1	154591489	A	G	0.009	0.001	1.9e-10	3.3e-05	40.6
Lipid Metabolism	HDL	rs36004974	1	154818403	G	A	-0.009	0.001	3.9e-09	2.8e-05	34.7
Lipid Metabolism	HDL	rs11548200	1	156290656	C	T	-0.016	0.003	1.9e-08	2.6e-05	31.6
Lipid Metabolism	HDL	rs12145743	1	156700651	G	T	0.011	0.001	2.0e-14	4.7e-05	58.6
Lipid Metabolism	HDL	rs4656985	1	161079155	C	T	0.009	0.001	4.6e-10	3.1e-05	38.8
Lipid Metabolism	HDL	rs1771591	1	161610734	A	G	-0.010	0.002	3.1e-09	3.6e-05	35.1
Lipid Metabolism	HDL	rs10911257	1	172166548	T	C	0.008	0.001	1.1e-08	2.6e-05	32.7
Lipid Metabolism	HDL	rs9425591	1	172358697	T	G	0.012	0.001	3.7e-17	5.7e-05	71.0
Lipid Metabolism	HDL	rs10218528	1	174447588	A	T	-0.009	0.001	1.7e-09	2.9e-05	36.3
Lipid Metabolism	HDL	rs478788	1	177887018	C	T	-0.011	0.002	7.7e-11	3.4e-05	42.3
Lipid Metabolism	HDL	rs10913570	1	178523126	C	A	-0.018	0.001	1.5e-39	1.4e-04	173.1
Lipid Metabolism	HDL	rs1689791	1	182151909	G	A	-0.026	0.001	3.7e-70	2.5e-04	313.5
Lipid Metabolism	HDL	rs1952256	1	184035116	G	A	-0.009	0.001	1.6e-09	3.0e-05	36.4
Lipid Metabolism	HDL	rs10801586	1	197021189	T	C	0.012	0.002	1.0e-09	3.0e-05	37.3
Lipid Metabolism	HDL	rs6675366	1	198517308	C	T	-0.022	0.004	8.0e-10	3.0e-05	37.8
Lipid Metabolism	HDL	rs2816916	1	200034969	G	A	-0.009	0.002	3.3e-09	2.8e-05	35.0
Lipid Metabolism	HDL	rs2690034	1	200143844	G	C	0.008	0.002	4.9e-08	2.4e-05	29.8
Lipid Metabolism	HDL	rs7532633	1	202273023	C	T	0.010	0.001	9.0e-12	3.8e-05	46.5
Lipid Metabolism	HDL	rs6677511	1	203510228	G	T	0.011	0.001	4.8e-13	4.5e-05	52.3
Lipid Metabolism	HDL	rs6664360	1	205120640	T	C	-0.012	0.002	5.7e-14	4.5e-05	56.5
Lipid Metabolism	HDL	rs1172122	1	205261174	C	T	0.013	0.002	2.8e-09	2.8e-05	35.3
Lipid Metabolism	HDL	rs951366	1	205685352	C	T	-0.014	0.001	3.2e-24	8.3e-05	103.1
Lipid Metabolism	HDL	rs9242	1	206637395	T	C	0.009	0.001	4.8e-10	3.2e-05	38.7
Lipid Metabolism	HDL	rs12034991	1	210145298	G	A	-0.009	0.001	1.1e-10	3.3e-05	41.6
Lipid Metabolism	HDL	rs12023488	1	212498198	T	C	0.010	0.001	7.2e-12	3.8e-05	47.0
Lipid Metabolism	HDL	rs59770119	1	219664627	A	G	-0.017	0.001	6.4e-33	1.2e-04	142.8
Lipid Metabolism	HDL	rs188273166	1	220101499	A	G	-0.219	0.027	9.7e-16	7.2e-05	64.5
Lipid Metabolism	HDL	rs2807845	1	220996287	T	G	0.014	0.001	1.8e-24	8.4e-05	104.2
Lipid Metabolism	HDL	rs2247213	1	221055463	A	G	-0.008	0.001	1.1e-08	2.6e-05	32.7
Lipid Metabolism	HDL	rs34992407	1	222940523	T	C	-0.010	0.002	4.8e-08	2.4e-05	29.8
Lipid Metabolism	HDL	rs56105022	1	224544746	A	G	-0.030	0.004	2.1e-14	4.7e-05	58.4
Lipid Metabolism	HDL	rs72750850	1	225550831	C	T	-0.021	0.003	4.2e-10	3.1e-05	39.0
Lipid Metabolism	HDL	rs12062317	1	228062661	A	G	0.008	0.001	2.5e-08	2.5e-05	31.1
Lipid Metabolism	HDL	rs116503257	1	230016672	C	A	-0.038	0.005	2.7e-14	4.7e-05	58.0
Lipid Metabolism	HDL	rs12047300	1	230138848	A	G	-0.015	0.003	8.2e-09	2.7e-05	33.2
Lipid Metabolism	HDL	rs116537941	1	230259882	G	A	-0.033	0.005	1.4e-10	3.3e-05	41.1
Lipid Metabolism	HDL	rs116773789	1	230265884	T	C	-0.062	0.009	5.8e-12	4.3e-05	47.4
Lipid Metabolism	HDL	rs10864727	1	230297939	G	A	0.051	0.001	1.0e-200	1.1e-03	1328.4
Lipid Metabolism	HDL	rs78517535	1	230326892	C	T	-0.071	0.008	2.6e-20	6.9e-05	85.3

Phenotype	Trait	SNP	Chr	Position (GRCh37/hg19)	EA	NEA	BETA	SE	P-value	R2	F
Lipid Metabolism	HDL	rs16851339	1	230416832	A	T	0.019	0.002	1.6e-29	1.0e-04	127.3
Lipid Metabolism	HDL	rs78572177	1	230450676	G	C	-0.021	0.003	2.5e-11	3.6e-05	44.6
Lipid Metabolism	HDL	rs558971	1	234853406	G	A	0.017	0.001	2.3e-34	1.2e-04	149.5
Lipid Metabolism	HDL	rs12725590	1	235023889	C	T	-0.014	0.003	1.5e-08	2.6e-05	32.1
Lipid Metabolism	HDL	rs10924177	1	236259417	G	A	-0.010	0.002	4.1e-09	2.8e-05	34.6
Lipid Metabolism	HDL	rs300798	2	105917	G	A	-0.012	0.002	9.3e-09	2.7e-05	33.0
Lipid Metabolism	HDL	rs17713729	2	249092	C	A	0.016	0.001	7.8e-28	9.6e-05	119.6
Lipid Metabolism	HDL	rs6548239	2	635154	A	G	-0.013	0.002	4.7e-13	4.2e-05	52.3
Lipid Metabolism	HDL	rs114703824	2	3435446	G	A	-0.035	0.006	9.2e-09	2.7e-05	33.0
Lipid Metabolism	HDL	rs3820897	2	3642361	C	T	-0.022	0.002	4.2e-37	1.3e-04	162.0
Lipid Metabolism	HDL	rs2884374	2	11731846	C	T	-0.008	0.001	1.2e-08	2.6e-05	32.6
Lipid Metabolism	HDL	rs11096641	2	20374158	A	G	-0.013	0.001	1.8e-20	6.9e-05	86.0
Lipid Metabolism	HDL	rs564696598	2	21087274	A	T	0.082	0.014	4.8e-09	5.0e-05	34.3
Lipid Metabolism	HDL	rs6734506	2	21134656	T	C	0.016	0.001	1.1e-26	9.2e-05	114.3
Lipid Metabolism	HDL	rs1042034	2	21225281	T	C	-0.060	0.002	1.0e-200	1.0e-03	1289.8
Lipid Metabolism	HDL	rs187350477	2	21350277	C	T	0.103	0.013	8.4e-15	5.0e-05	60.2
Lipid Metabolism	HDL	rs11682662	2	21378467	A	G	-0.020	0.002	5.9e-19	6.4e-05	79.1
Lipid Metabolism	HDL	rs543438430	2	21504380	A	G	0.070	0.013	2.8e-08	2.5e-05	30.8
Lipid Metabolism	HDL	rs80341249	2	21658852	C	T	0.055	0.009	1.8e-09	3.0e-05	36.2
Lipid Metabolism	HDL	rs184344489	2	21944355	A	G	0.119	0.018	3.1e-11	5.8e-05	44.1
Lipid Metabolism	HDL	rs13432743	2	25069392	G	A	-0.013	0.002	3.9e-08	2.4e-05	30.2
Lipid Metabolism	HDL	rs6546949	2	26592429	C	T	-0.009	0.001	2.7e-10	3.2e-05	39.9
Lipid Metabolism	HDL	rs1001204	2	30478779	G	A	-0.012	0.002	1.2e-12	4.1e-05	50.5
Lipid Metabolism	HDL	rs12470495	2	32529416	A	G	0.009	0.002	3.9e-09	2.8e-05	34.6
Lipid Metabolism	HDL	rs1988650	2	42689362	T	C	0.010	0.002	1.2e-09	3.0e-05	37.0
Lipid Metabolism	HDL	rs75439527	2	48388666	A	G	-0.012	0.002	5.0e-08	2.4e-05	29.7
Lipid Metabolism	HDL	rs17326656	2	48962291	T	G	-0.016	0.002	4.7e-23	7.9e-05	97.8
Lipid Metabolism	HDL	rs12997031	2	50689260	A	G	-0.008	0.001	8.5e-09	2.7e-05	33.2
Lipid Metabolism	HDL	rs805361	2	54165846	T	G	0.010	0.001	1.1e-13	4.4e-05	55.2
Lipid Metabolism	HDL	rs2902649	2	55248357	G	T	0.008	0.001	2.1e-08	2.5e-05	31.4
Lipid Metabolism	HDL	rs1861410	2	58933591	T	C	0.011	0.001	2.5e-15	5.1e-05	62.7
Lipid Metabolism	HDL	rs10172678	2	59294558	C	T	0.008	0.001	3.7e-09	2.8e-05	34.8
Lipid Metabolism	HDL	rs17432483	2	63023836	C	T	-0.018	0.003	1.0e-09	3.0e-05	37.3
Lipid Metabolism	HDL	rs2723064	2	65279805	C	T	0.016	0.001	5.7e-31	1.1e-04	133.9
Lipid Metabolism	HDL	rs11883967	2	66673862	C	A	0.011	0.001	2.3e-13	4.4e-05	53.7
Lipid Metabolism	HDL	rs1986601	2	70207503	G	A	0.008	0.001	4.2e-08	2.4e-05	30.1
Lipid Metabolism	HDL	rs7586605	2	85561141	T	C	0.012	0.002	6.3e-15	4.9e-05	60.8
Lipid Metabolism	HDL	rs77023845	2	100712346	A	G	0.015	0.002	9.3e-16	5.2e-05	64.6
Lipid Metabolism	HDL	rs62152937	2	101613739	T	C	-0.009	0.002	8.0e-10	3.0e-05	37.8
Lipid Metabolism	HDL	rs4851057	2	105939237	T	C	-0.011	0.002	4.3e-08	2.4e-05	30.0
Lipid Metabolism	HDL	rs3789112	2	111714577	T	C	-0.009	0.002	5.0e-10	3.1e-05	38.7
Lipid Metabolism	HDL	rs3761706	2	111899881	A	G	-0.019	0.003	2.7e-11	3.6e-05	44.4
Lipid Metabolism	HDL	rs11688682	2	121347612	C	G	0.018	0.002	6.4e-23	8.7e-05	97.2
Lipid Metabolism	HDL	rs4953936	2	135581512	T	C	0.011	0.001	3.4e-13	4.3e-05	53.0
Lipid Metabolism	HDL	rs1470457	2	136581848	G	A	0.018	0.002	1.1e-25	9.3e-05	109.7
Lipid Metabolism	HDL	rs7568603	2	146352541	G	T	0.008	0.001	1.8e-09	2.9e-05	36.2
Lipid Metabolism	HDL	rs811869	2	147852042	A	G	0.008	0.001	7.9e-09	2.7e-05	33.3
Lipid Metabolism	HDL	rs4267470	2	153563858	A	G	0.008	0.001	1.2e-08	2.6e-05	32.4
Lipid Metabolism	HDL	rs2509903	2	158514510	C	T	-0.012	0.002	3.5e-09	2.8e-05	34.9
Lipid Metabolism	HDL	rs76840964	2	160754947	G	A	0.013	0.002	1.1e-09	3.0e-05	37.1
Lipid Metabolism	HDL	rs12692596	2	161265910	T	C	-0.009	0.001	2.5e-10	3.2e-05	40.0
Lipid Metabolism	HDL	rs141102226	2	162992004	T	A	0.032	0.006	1.2e-08	2.6e-05	32.6
Lipid Metabolism	HDL	rs1990760	2	163124051	T	C	-0.009	0.001	6.6e-11	3.4e-05	42.6
Lipid Metabolism	HDL	rs13389219	2	165528876	T	C	0.029	0.001	8.0e-95	3.4e-04	426.8
Lipid Metabolism	HDL	rs355799	2	165694898	T	C	0.015	0.002	2.0e-14	4.7e-05	58.5
Lipid Metabolism	HDL	rs17694506	2	171632225	T	C	0.008	0.001	1.7e-08	2.6e-05	31.8

Phenotype	Trait	SNP	Chr	Position (GRCh37/hg19)	EA	NEA	BETA	SE	P-value	R2	F
Lipid Metabolism	HDL	rs3754749	2	173906149	G	A	0.013	0.002	6.2e-13	4.2e-05	51.8
Lipid Metabolism	HDL	rs10497442	2	176436826	C	T	0.009	0.001	5.4e-09	2.7e-05	34.0
Lipid Metabolism	HDL	rs13005431	2	178121112	C	T	-0.008	0.001	1.3e-08	2.6e-05	32.3
Lipid Metabolism	HDL	rs10167485	2	181321102	G	A	-0.009	0.002	5.5e-09	2.7e-05	34.0
Lipid Metabolism	HDL	rs111917980	2	188276584	A	G	0.010	0.001	4.9e-12	3.8e-05	47.7
Lipid Metabolism	HDL	rs7581601	2	203497786	C	A	-0.018	0.001	6.4e-32	1.1e-04	138.3
Lipid Metabolism	HDL	rs2551639	2	208396187	G	A	0.013	0.002	9.8e-13	4.1e-05	50.9
Lipid Metabolism	HDL	rs1047891	2	211540507	A	C	-0.016	0.002	1.2e-27	9.6e-05	118.7
Lipid Metabolism	HDL	rs3791697	2	212272614	A	G	-0.009	0.002	1.2e-08	2.6e-05	32.5
Lipid Metabolism	HDL	rs4273218	2	215370140	G	T	-0.010	0.001	4.5e-12	3.9e-05	47.9
Lipid Metabolism	HDL	rs79399657	2	219400820	C	T	0.027	0.004	9.5e-12	3.8e-05	46.4
Lipid Metabolism	HDL	rs78058190	2	219699999	A	G	-0.081	0.004	2.7e-113	4.2e-04	511.7
Lipid Metabolism	HDL	rs2972145	2	227101309	C	T	-0.037	0.001	2.0e-149	5.4e-04	677.8
Lipid Metabolism	HDL	rs151029783	2	227126806	T	A	-0.040	0.007	1.9e-08	2.6e-05	31.6
Lipid Metabolism	HDL	rs10933140	2	227209828	G	A	0.016	0.002	7.0e-22	7.4e-05	92.4
Lipid Metabolism	HDL	rs11694828	2	228565656	A	G	0.015	0.001	5.3e-24	8.4e-05	102.1
Lipid Metabolism	HDL	rs7586105	2	228571182	C	A	-0.016	0.002	1.7e-13	4.4e-05	54.3
Lipid Metabolism	HDL	rs985891	2	230019348	A	G	-0.009	0.001	7.6e-11	3.4e-05	42.3
Lipid Metabolism	HDL	rs7604426	2	230123353	G	A	0.008	0.002	2.8e-08	2.5e-05	30.8
Lipid Metabolism	HDL	rs59916403	2	242370751	T	G	0.013	0.001	6.7e-19	6.4e-05	78.8
Lipid Metabolism	HDL	rs10191488	2	242404135	T	G	0.009	0.001	1.7e-10	3.3e-05	40.8
Lipid Metabolism	HDL	rs12485478	3	12351223	G	A	-0.054	0.004	3.4e-37	1.3e-04	162.4
Lipid Metabolism	HDL	rs4135250	3	12396845	G	A	0.017	0.002	1.2e-15	5.1e-05	64.0
Lipid Metabolism	HDL	rs147070419	3	12442098	G	T	-0.063	0.010	3.5e-11	3.6e-05	43.9
Lipid Metabolism	HDL	rs148753885	3	12739169	G	A	-0.014	0.003	9.6e-09	2.6e-05	32.9
Lipid Metabolism	HDL	rs1529942	3	14495455	A	G	0.008	0.001	3.3e-08	2.5e-05	30.5
Lipid Metabolism	HDL	rs2654652	3	15782174	T	C	0.013	0.001	1.0e-18	6.3e-05	78.0
Lipid Metabolism	HDL	rs2948080	3	20079489	C	G	-0.009	0.002	2.4e-08	2.5e-05	31.2
Lipid Metabolism	HDL	rs2044754	3	24292940	G	A	-0.017	0.002	1.7e-14	4.7e-05	58.9
Lipid Metabolism	HDL	rs6779665	3	36952932	C	T	0.012	0.001	2.9e-18	6.2e-05	76.0
Lipid Metabolism	HDL	rs4128529	3	43435543	C	T	0.020	0.004	3.5e-08	2.5e-05	30.4
Lipid Metabolism	HDL	rs2305637	3	47045846	T	C	-0.020	0.002	1.6e-27	9.7e-05	118.2
Lipid Metabolism	HDL	rs2100692	3	48193753	A	G	0.019	0.002	2.0e-14	4.7e-05	58.6
Lipid Metabolism	HDL	rs74780677	3	48601774	G	A	-0.068	0.005	1.2e-36	1.3e-04	159.8
Lipid Metabolism	HDL	rs75654367	3	48710739	T	C	-0.020	0.003	2.4e-13	4.3e-05	53.6
Lipid Metabolism	HDL	rs187738872	3	49391714	A	G	0.044	0.008	1.6e-08	3.0e-05	32.0
Lipid Metabolism	HDL	rs116238056	3	49821362	A	G	-0.059	0.006	4.6e-26	9.0e-05	111.5
Lipid Metabolism	HDL	rs11925192	3	50038664	A	G	-0.022	0.001	2.1e-54	1.9e-04	241.2
Lipid Metabolism	HDL	rs73087292	3	51190096	G	A	0.016	0.002	1.0e-13	4.4e-05	55.3
Lipid Metabolism	HDL	rs79558344	3	51754781	T	C	-0.053	0.006	1.2e-19	6.7e-05	82.2
Lipid Metabolism	HDL	rs2240921	3	52830764	T	C	0.026	0.002	4.3e-34	1.2e-04	148.2
Lipid Metabolism	HDL	rs7611386	3	53053402	G	T	0.011	0.002	2.9e-11	5.0e-05	44.2
Lipid Metabolism	HDL	rs6779169	3	57423086	T	A	-0.008	0.001	1.6e-08	2.6e-05	31.9
Lipid Metabolism	HDL	rs76824303	3	62459819	C	A	0.016	0.002	8.0e-12	3.8e-05	46.8
Lipid Metabolism	HDL	rs141148474	3	66291685	C	A	0.015	0.002	1.4e-10	3.3e-05	41.1
Lipid Metabolism	HDL	rs6549403	3	66836922	G	A	-0.011	0.002	6.5e-10	3.1e-05	38.2
Lipid Metabolism	HDL	rs62250977	3	69836592	A	G	0.011	0.002	5.1e-11	3.5e-05	43.1
Lipid Metabolism	HDL	rs7630599	3	70502311	T	A	-0.009	0.001	7.4e-11	3.4e-05	42.4
Lipid Metabolism	HDL	rs35540156	3	71522178	G	A	0.010	0.001	4.9e-12	3.8e-05	47.7
Lipid Metabolism	HDL	rs13066793	3	87037543	G	A	0.018	0.002	1.6e-13	4.4e-05	54.4
Lipid Metabolism	HDL	rs17828045	3	107821546	G	T	0.009	0.001	2.0e-09	2.9e-05	36.0
Lipid Metabolism	HDL	rs9833523	3	114491182	A	G	-0.014	0.002	2.2e-14	4.7e-05	58.3
Lipid Metabolism	HDL	rs12497807	3	115116357	A	C	0.010	0.001	5.7e-11	3.4e-05	42.9
Lipid Metabolism	HDL	rs17810676	3	119780549	C	T	0.013	0.001	2.4e-19	6.6e-05	80.9
Lipid Metabolism	HDL	rs11708067	3	123065778	G	A	-0.013	0.002	2.4e-15	5.0e-05	62.7
Lipid Metabolism	HDL	rs113761591	3	127305355	T	C	-0.011	0.002	7.3e-10	3.0e-05	37.9

Phenotype	Trait	SNP	Chr	Position (GRCh37/hg19)	EA	NEA	BETA	SE	P-value	R2	F
Lipid Metabolism	HDL	rs2811468	3	129296847	G	A	0.014	0.002	1.3e-16	5.5e-05	68.5
Lipid Metabolism	HDL	rs1320903	3	131758077	A	G	-0.009	0.001	1.5e-09	2.9e-05	36.5
Lipid Metabolism	HDL	rs645040	3	135926622	T	G	-0.026	0.002	2.9e-54	1.9e-04	240.6
Lipid Metabolism	HDL	rs17633378	3	142778355	T	C	0.008	0.001	6.1e-09	2.7e-05	33.8
Lipid Metabolism	HDL	rs62271373	3	150066540	A	T	-0.037	0.003	2.8e-32	1.1e-04	139.9
Lipid Metabolism	HDL	rs35598887	3	150146402	A	T	0.012	0.002	6.9e-10	3.6e-05	38.0
Lipid Metabolism	HDL	rs13082076	3	152085277	C	T	0.012	0.002	6.4e-16	5.2e-05	65.3
Lipid Metabolism	HDL	rs7648816	3	153837632	C	T	-0.013	0.002	2.1e-09	2.9e-05	35.8
Lipid Metabolism	HDL	rs62285077	3	156244888	T	C	0.012	0.002	1.6e-10	3.3e-05	40.9
Lipid Metabolism	HDL	rs10049088	3	156797648	T	C	0.021	0.001	3.6e-50	1.8e-04	221.8
Lipid Metabolism	HDL	rs3113	3	156814949	C	T	-0.009	0.001	5.6e-10	3.1e-05	38.5
Lipid Metabolism	HDL	rs2152425	3	160037904	A	G	-0.008	0.001	2.2e-09	2.9e-05	35.8
Lipid Metabolism	HDL	rs9839164	3	168790656	C	T	-0.013	0.002	2.5e-08	2.5e-05	31.0
Lipid Metabolism	HDL	rs79287178	3	172294500	A	G	-0.027	0.004	2.7e-10	3.2e-05	39.9
Lipid Metabolism	HDL	rs7647044	3	173752290	G	A	0.009	0.002	1.2e-08	2.6e-05	32.5
Lipid Metabolism	HDL	rs843371	3	183994536	G	A	0.011	0.002	2.3e-11	3.6e-05	44.7
Lipid Metabolism	HDL	rs10513801	3	185822353	G	T	-0.030	0.002	8.5e-49	1.7e-04	215.5
Lipid Metabolism	HDL	rs4686738	3	185877911	A	G	0.011	0.001	8.2e-16	5.2e-05	64.8
Lipid Metabolism	HDL	rs1491997	3	187447926	T	C	0.008	0.001	1.1e-08	2.7e-05	32.7
Lipid Metabolism	HDL	rs139828053	3	195298892	C	T	-0.030	0.004	2.6e-12	4.0e-05	48.9
Lipid Metabolism	HDL	rs6772271	3	196193763	T	C	0.011	0.002	3.0e-12	3.9e-05	48.7
Lipid Metabolism	HDL	rs34311866	4	951947	C	T	-0.017	0.002	6.6e-21	7.1e-05	88.0
Lipid Metabolism	HDL	rs115880342	4	1365635	G	A	0.023	0.004	5.9e-09	2.7e-05	33.9
Lipid Metabolism	HDL	rs362307	4	3241845	T	C	-0.018	0.003	1.5e-11	3.7e-05	45.5
Lipid Metabolism	HDL	rs4450871	4	4990298	G	A	0.011	0.002	5.1e-14	4.9e-05	56.7
Lipid Metabolism	HDL	rs7694606	4	18028987	A	G	-0.014	0.002	2.3e-13	4.3e-05	53.8
Lipid Metabolism	HDL	rs17576121	4	23893274	C	T	0.009	0.001	2.0e-09	2.9e-05	36.0
Lipid Metabolism	HDL	rs75309341	4	26029869	A	G	0.055	0.010	2.1e-08	2.6e-05	31.4
Lipid Metabolism	HDL	rs73243877	4	26047616	G	A	-0.021	0.002	5.1e-31	1.1e-04	134.1
Lipid Metabolism	HDL	rs12646350	4	37160803	C	A	0.009	0.001	6.2e-10	3.1e-05	38.2
Lipid Metabolism	HDL	rs16994478	4	38398478	T	C	-0.014	0.002	5.6e-09	2.7e-05	34.0
Lipid Metabolism	HDL	rs7658893	4	38782420	A	G	-0.010	0.002	8.1e-10	3.0e-05	37.7
Lipid Metabolism	HDL	rs10517530	4	39788949	C	T	0.010	0.001	2.5e-11	3.6e-05	44.5
Lipid Metabolism	HDL	rs10938398	4	45186139	A	G	-0.010	0.001	2.2e-12	4.0e-05	49.3
Lipid Metabolism	HDL	rs7684939	4	55509189	A	G	0.010	0.001	1.8e-13	4.4e-05	54.2
Lipid Metabolism	HDL	rs1620509	4	57946388	A	T	0.009	0.001	1.0e-09	3.0e-05	37.3
Lipid Metabolism	HDL	rs35662434	4	69339933	G	A	0.015	0.002	2.6e-18	6.4e-05	76.2
Lipid Metabolism	HDL	rs190671241	4	73574142	G	A	0.115	0.018	2.7e-10	5.0e-05	39.9
Lipid Metabolism	HDL	rs187918276	4	74033564	C	G	0.152	0.021	1.1e-12	5.6e-05	50.6
Lipid Metabolism	HDL	rs2175639	4	76638911	T	C	0.009	0.001	7.0e-11	3.4e-05	42.5
Lipid Metabolism	HDL	rs6831339	4	83906408	T	C	-0.012	0.002	5.3e-14	4.6e-05	56.6
Lipid Metabolism	HDL	rs13137142	4	87767219	G	T	-0.009	0.002	2.4e-08	2.5e-05	31.1
Lipid Metabolism	HDL	rs342469	4	88050554	A	G	-0.015	0.001	3.4e-27	9.4e-05	116.7
Lipid Metabolism	HDL	rs114796149	4	88920985	A	G	-0.031	0.006	2.3e-08	2.6e-05	31.2
Lipid Metabolism	HDL	rs2167750	4	89730074	T	C	-0.019	0.001	2.6e-43	1.5e-04	190.4
Lipid Metabolism	HDL	rs10001500	4	89939520	T	A	0.024	0.004	1.1e-08	2.6e-05	32.7
Lipid Metabolism	HDL	rs2571037	4	96138183	A	G	0.009	0.001	5.6e-10	3.1e-05	38.5
Lipid Metabolism	HDL	rs6532773	4	99789010	G	A	0.011	0.001	2.2e-14	4.7e-05	58.3
Lipid Metabolism	HDL	rs547953616	4	100020668	T	C	0.169	0.015	1.4e-29	1.1e-04	127.5
Lipid Metabolism	HDL	rs12639747	4	100493242	T	C	0.029	0.004	2.0e-15	5.1e-05	63.1
Lipid Metabolism	HDL	rs185228188	4	101019003	C	A	0.138	0.024	1.5e-08	4.8e-05	32.1
Lipid Metabolism	HDL	rs115064203	4	102020510	A	T	-0.030	0.005	2.5e-08	2.5e-05	31.1
Lipid Metabolism	HDL	rs13135092	4	103198082	G	A	-0.074	0.003	3.2e-169	6.2e-04	768.8
Lipid Metabolism	HDL	rs112673097	4	103202927	A	G	-0.043	0.007	1.2e-10	3.4e-05	41.4
Lipid Metabolism	HDL	rs150813383	4	103315570	A	C	0.011	0.002	5.9e-09	2.7e-05	33.9
Lipid Metabolism	HDL	rs4699038	4	103879412	A	G	0.015	0.002	5.5e-18	6.0e-05	74.7

Phenotype	Trait	SNP	Chr	Position (GRCh37/hg19)	EA	NEA	BETA	SE	P-value	R2	F
Lipid Metabolism	HDL	rs112477297	4	104246425	C	G	-0.047	0.008	1.4e-08	2.6e-05	32.2
Lipid Metabolism	HDL	rs75877836	4	104313246	C	T	-0.058	0.010	1.5e-08	2.6e-05	32.1
Lipid Metabolism	HDL	rs144317085	4	105806108	T	A	-0.027	0.004	6.5e-14	4.5e-05	56.2
Lipid Metabolism	HDL	rs12645165	4	106087217	C	T	-0.012	0.001	5.4e-16	5.3e-05	65.6
Lipid Metabolism	HDL	rs114816312	4	110638824	T	C	-0.126	0.009	7.4e-43	1.6e-04	188.3
Lipid Metabolism	HDL	rs2271577	4	119147975	G	A	0.010	0.002	1.7e-08	2.6e-05	31.8
Lipid Metabolism	HDL	rs72712556	4	140900251	A	G	0.008	0.001	3.1e-08	2.5e-05	30.7
Lipid Metabolism	HDL	rs10006971	4	143318853	G	A	0.010	0.001	2.2e-13	4.3e-05	53.8
Lipid Metabolism	HDL	rs6813785	4	143777552	C	A	-0.009	0.001	5.3e-10	3.1e-05	38.6
Lipid Metabolism	HDL	rs7658229	4	145709162	T	C	-0.013	0.002	7.9e-10	3.1e-05	37.8
Lipid Metabolism	HDL	rs13134992	4	146393096	T	C	0.009	0.001	2.8e-10	3.2e-05	39.8
Lipid Metabolism	HDL	rs3775327	4	148985720	C	T	-0.010	0.002	2.8e-09	2.9e-05	35.3
Lipid Metabolism	HDL	rs2290846	4	151199080	A	G	-0.011	0.002	6.4e-12	3.8e-05	47.2
Lipid Metabolism	HDL	rs72729623	4	154208278	T	C	-0.016	0.002	5.1e-17	5.6e-05	70.3
Lipid Metabolism	HDL	rs11941933	4	154371229	T	C	0.010	0.002	1.0e-09	3.0e-05	37.3
Lipid Metabolism	HDL	rs1425485	4	157681890	T	C	0.019	0.002	1.1e-29	1.0e-04	128.0
Lipid Metabolism	HDL	rs62340875	4	185782590	A	G	0.010	0.002	3.4e-08	2.5e-05	30.5
Lipid Metabolism	HDL	rs61731455	5	665295	G	A	0.011	0.002	1.2e-10	3.3e-05	41.4
Lipid Metabolism	HDL	rs62358675	5	39094734	G	A	-0.011	0.002	1.3e-08	2.6e-05	32.3
Lipid Metabolism	HDL	rs76086106	5	39491186	A	G	0.027	0.004	7.2e-15	4.9e-05	60.5
Lipid Metabolism	HDL	rs2910943	5	39519027	T	C	0.009	0.001	3.1e-09	2.8e-05	35.2
Lipid Metabolism	HDL	rs6866562	5	52697383	A	G	-0.011	0.001	9.0e-15	4.8e-05	60.1
Lipid Metabolism	HDL	rs7707821	5	52813375	G	A	0.008	0.001	2.9e-09	2.8e-05	35.2
Lipid Metabolism	HDL	rs5013866	5	53300947	G	A	-0.014	0.002	3.7e-18	6.1e-05	75.5
Lipid Metabolism	HDL	rs17266519	5	53388648	G	A	-0.011	0.002	8.1e-09	2.7e-05	33.2
Lipid Metabolism	HDL	rs30000	5	55803533	A	G	-0.025	0.002	5.8e-58	2.1e-04	257.6
Lipid Metabolism	HDL	rs113680536	5	55843687	G	A	0.056	0.010	2.2e-08	2.6e-05	31.3
Lipid Metabolism	HDL	rs28650790	5	55861464	T	C	-0.030	0.002	1.3e-64	2.3e-04	288.2
Lipid Metabolism	HDL	rs37521	5	57577463	T	C	0.009	0.001	1.6e-09	2.9e-05	36.4
Lipid Metabolism	HDL	rs256499	5	67660888	C	T	-0.009	0.001	1.7e-10	3.3e-05	40.7
Lipid Metabolism	HDL	rs2134157	5	72944839	C	T	0.013	0.002	1.2e-16	5.5e-05	68.7
Lipid Metabolism	HDL	rs6874626	5	74999576	A	G	0.016	0.001	1.2e-26	9.4e-05	114.2
Lipid Metabolism	HDL	rs6874833	5	75696662	T	C	0.010	0.001	2.8e-11	3.6e-05	44.3
Lipid Metabolism	HDL	rs10052346	5	78472599	T	G	0.010	0.001	2.6e-12	4.0e-05	49.0
Lipid Metabolism	HDL	rs115912456	5	82815158	G	A	0.029	0.003	4.7e-17	5.7e-05	70.5
Lipid Metabolism	HDL	rs10514370	5	92435481	T	G	0.009	0.001	2.7e-10	3.2e-05	39.9
Lipid Metabolism	HDL	rs25863	5	96105926	A	G	0.009	0.001	1.0e-11	3.7e-05	46.3
Lipid Metabolism	HDL	rs12520308	5	111253269	C	T	0.008	0.001	1.3e-08	2.6e-05	32.3
Lipid Metabolism	HDL	rs11742162	5	112385510	C	T	-0.010	0.001	5.2e-12	3.8e-05	47.6
Lipid Metabolism	HDL	rs12234017	5	118711834	G	A	0.016	0.002	4.3e-25	8.6e-05	107.1
Lipid Metabolism	HDL	rs35369952	5	124547821	A	G	0.011	0.002	9.9e-10	3.0e-05	37.3
Lipid Metabolism	HDL	rs3749748	5	127350549	T	C	0.012	0.002	2.7e-13	4.3e-05	53.4
Lipid Metabolism	HDL	rs2436348	5	129192881	A	G	0.010	0.001	2.3e-11	3.6e-05	44.7
Lipid Metabolism	HDL	rs9327595	5	130492613	A	G	0.023	0.004	2.4e-08	2.5e-05	31.2
Lipid Metabolism	HDL	rs530052742	5	130647274	G	A	0.104	0.019	4.6e-08	3.7e-05	29.9
Lipid Metabolism	HDL	rs680994	5	131316200	C	A	-0.025	0.004	4.9e-12	3.8e-05	47.7
Lipid Metabolism	HDL	rs10479001	5	131607721	T	C	0.024	0.003	9.1e-13	4.1e-05	51.0
Lipid Metabolism	HDL	rs72801474	5	132444128	A	G	0.026	0.002	8.1e-27	9.2e-05	115.0
Lipid Metabolism	HDL	rs254559	5	134444982	A	C	-0.011	0.001	4.4e-15	4.9e-05	61.5
Lipid Metabolism	HDL	rs12520112	5	135450333	G	A	0.009	0.002	1.3e-08	2.6e-05	32.4
Lipid Metabolism	HDL	rs6867299	5	139044217	C	T	-0.008	0.001	6.1e-09	2.8e-05	33.8
Lipid Metabolism	HDL	rs62384243	5	140099762	T	C	0.010	0.002	1.6e-09	3.5e-05	36.5
Lipid Metabolism	HDL	rs13163489	5	141704370	C	A	0.011	0.002	2.4e-14	4.7e-05	58.2
Lipid Metabolism	HDL	rs853175	5	142636790	C	T	-0.008	0.001	1.1e-08	2.6e-05	32.6
Lipid Metabolism	HDL	rs78119163	5	144316672	G	A	-0.013	0.002	1.2e-08	2.6e-05	32.4
Lipid Metabolism	HDL	rs35553545	5	148844793	T	G	-0.010	0.002	2.3e-09	2.9e-05	35.7

Phenotype	Trait	SNP	Chr	Position (GRCh37/hg19)	EA	NEA	BETA	SE	P-value	R2	F
Lipid Metabolism	HDL	rs11167657	5	153443782	A	T	-0.012	0.001	4.1e-16	5.3e-05	66.2
Lipid Metabolism	HDL	rs1650527	5	158022724	T	C	-0.021	0.002	3.3e-36	1.3e-04	157.9
Lipid Metabolism	HDL	rs2901184	5	158652395	A	G	0.011	0.002	7.1e-12	3.8e-05	47.0
Lipid Metabolism	HDL	rs7730676	5	170611224	T	C	-0.010	0.002	2.0e-11	3.7e-05	45.0
Lipid Metabolism	HDL	rs72816122	5	172524035	T	C	0.013	0.002	2.3e-10	3.2e-05	40.2
Lipid Metabolism	HDL	rs72812818	5	173356752	C	G	-0.011	0.001	9.6e-14	4.5e-05	55.4
Lipid Metabolism	HDL	rs6882591	5	176546460	A	G	0.017	0.002	3.5e-17	5.7e-05	71.0
Lipid Metabolism	HDL	rs6879613	5	179159627	C	T	0.012	0.002	1.5e-09	2.9e-05	36.5
Lipid Metabolism	HDL	rs62407768	5	180229950	G	C	-0.013	0.002	4.2e-08	2.8e-05	30.1
Lipid Metabolism	HDL	rs138692142	5	180349778	T	C	-0.072	0.008	1.6e-19	6.8e-05	81.6
Lipid Metabolism	HDL	rs1240811	6	16818805	T	C	0.011	0.001	1.7e-14	4.7e-05	58.8
Lipid Metabolism	HDL	rs6915996	6	20277483	A	G	0.009	0.002	4.3e-08	2.4e-05	30.0
Lipid Metabolism	HDL	rs17491647	6	24713723	C	A	0.010	0.002	2.8e-08	2.5e-05	30.9
Lipid Metabolism	HDL	rs35506517	6	26044864	C	T	-0.015	0.003	7.9e-09	2.7e-05	33.3
Lipid Metabolism	HDL	rs9379829	6	26172219	T	C	0.011	0.002	5.7e-12	3.8e-05	47.4
Lipid Metabolism	HDL	rs9368561	6	28168343	T	C	-0.016	0.002	6.6e-15	4.9e-05	60.7
Lipid Metabolism	HDL	rs2023463	6	29413003	T	C	-0.015	0.003	7.8e-10	3.2e-05	37.8
Lipid Metabolism	HDL	rs1611282	6	29730002	T	G	0.011	0.001	3.7e-13	4.3e-05	52.8
Lipid Metabolism	HDL	rs9263740	6	31111400	C	T	0.015	0.002	1.4e-15	5.1e-05	63.8
Lipid Metabolism	HDL	rs2853940	6	31249732	C	T	0.009	0.001	4.8e-09	2.8e-05	34.3
Lipid Metabolism	HDL	rs9265644	6	31300181	C	T	-0.020	0.002	6.2e-39	1.4e-04	170.3
Lipid Metabolism	HDL	rs146305655	6	32268953	A	G	-0.036	0.004	5.8e-21	7.7e-05	88.3
Lipid Metabolism	HDL	rs112744833	6	32304399	T	G	0.060	0.010	4.4e-10	3.5e-05	38.9
Lipid Metabolism	HDL	rs78546106	6	32305238	T	C	-0.028	0.004	1.6e-15	5.5e-05	63.5
Lipid Metabolism	HDL	rs17209803	6	32443061	A	C	-0.037	0.003	2.3e-26	9.9e-05	112.9
Lipid Metabolism	HDL	rs9273555	6	32628944	G	C	0.012	0.002	8.7e-09	3.8e-05	33.1
Lipid Metabolism	HDL	rs17213554	6	32759694	G	T	0.040	0.007	3.7e-08	2.6e-05	30.3
Lipid Metabolism	HDL	rs55775340	6	33751784	G	C	0.013	0.002	5.3e-15	4.9e-05	61.1
Lipid Metabolism	HDL	rs114760566	6	34192036	A	C	-0.047	0.004	6.2e-38	1.3e-04	165.8
Lipid Metabolism	HDL	rs2744961	6	34655000	T	C	-0.029	0.001	8.1e-88	3.2e-04	394.6
Lipid Metabolism	HDL	rs13205738	6	35101198	A	G	-0.023	0.002	9.4e-31	1.1e-04	132.9
Lipid Metabolism	HDL	rs78498719	6	35437113	G	C	0.017	0.003	1.6e-09	2.9e-05	36.4
Lipid Metabolism	HDL	rs6920166	6	36509376	T	C	-0.010	0.002	2.6e-08	2.5e-05	30.9
Lipid Metabolism	HDL	rs10807201	6	39095603	C	A	-0.008	0.001	4.6e-08	2.4e-05	29.9
Lipid Metabolism	HDL	rs142449754	6	41986427	T	C	-0.019	0.002	6.4e-33	1.1e-04	142.8
Lipid Metabolism	HDL	rs56106855	6	42909931	C	G	0.015	0.002	2.5e-21	7.3e-05	89.9
Lipid Metabolism	HDL	rs9472125	6	43756169	T	C	0.037	0.002	7.8e-56	2.0e-04	247.8
Lipid Metabolism	HDL	rs9394965	6	43794752	C	T	0.013	0.002	3.5e-18	6.1e-05	75.6
Lipid Metabolism	HDL	rs2749014	6	52627322	A	G	0.013	0.001	1.2e-19	6.6e-05	82.2
Lipid Metabolism	HDL	rs73744635	6	55437238	C	T	0.018	0.003	2.6e-08	2.5e-05	31.0
Lipid Metabolism	HDL	rs34724718	6	56557822	C	T	-0.010	0.002	3.8e-09	2.8e-05	34.7
Lipid Metabolism	HDL	rs9352238	6	76403681	A	C	-0.008	0.001	2.9e-09	2.8e-05	35.3
Lipid Metabolism	HDL	rs1173418	6	86402916	A	G	0.009	0.001	5.8e-11	3.5e-05	42.9
Lipid Metabolism	HDL	rs76781147	6	96052987	C	T	-0.022	0.004	6.1e-10	3.1e-05	38.3
Lipid Metabolism	HDL	rs570208294	6	98584715	A	T	0.011	0.002	1.0e-12	5.6e-05	50.8
Lipid Metabolism	HDL	rs180963	6	101127213	C	T	0.009	0.001	1.4e-09	3.0e-05	36.7
Lipid Metabolism	HDL	rs62427983	6	107441254	C	T	0.010	0.002	8.4e-11	3.5e-05	42.2
Lipid Metabolism	HDL	rs2806357	6	109275038	A	G	0.013	0.002	1.1e-14	4.9e-05	59.7
Lipid Metabolism	HDL	rs11759230	6	109640321	C	A	-0.017	0.001	5.9e-33	1.1e-04	143.0
Lipid Metabolism	HDL	rs1338668	6	116394809	A	G	0.015	0.001	2.6e-27	9.4e-05	117.2
Lipid Metabolism	HDL	rs2134088	6	117863688	A	G	-0.012	0.002	3.2e-08	2.5e-05	30.6
Lipid Metabolism	HDL	rs75077623	6	121863603	C	T	-0.014	0.002	4.7e-09	2.8e-05	34.3
Lipid Metabolism	HDL	rs1268074	6	126107791	C	G	-0.008	0.001	1.2e-08	2.6e-05	32.4
Lipid Metabolism	HDL	rs2745358	6	127391470	G	A	0.011	0.001	1.3e-14	4.8e-05	59.3
Lipid Metabolism	HDL	rs72959041	6	127454893	A	G	-0.047	0.003	8.5e-50	1.8e-04	220.1
Lipid Metabolism	HDL	rs9375694	6	130356608	A	G	-0.009	0.002	3.3e-10	3.2e-05	39.5

Phenotype	Trait	SNP	Chr	Position (GRCh37/hg19)	EA	NEA	BETA	SE	P-value	R2	F
Lipid Metabolism	HDL	rs55898152	6	131385009	G	A	-0.008	0.001	1.1e-08	2.6e-05	32.6
Lipid Metabolism	HDL	rs2245133	6	131931092	C	T	-0.014	0.002	1.2e-14	4.8e-05	59.5
Lipid Metabolism	HDL	rs9376090	6	135411228	C	T	-0.012	0.002	5.2e-14	4.6e-05	56.7
Lipid Metabolism	HDL	rs1022690	6	137085296	C	T	0.012	0.001	3.3e-18	6.2e-05	75.7
Lipid Metabolism	HDL	rs12175880	6	139293109	A	C	0.016	0.002	1.3e-17	5.9e-05	73.0
Lipid Metabolism	HDL	rs17585887	6	139835498	C	T	0.021	0.001	6.9e-52	1.8e-04	229.7
Lipid Metabolism	HDL	rs672341	6	153455994	A	G	0.014	0.001	4.0e-23	7.9e-05	98.1
Lipid Metabolism	HDL	rs2504958	6	160750545	T	C	0.014	0.002	9.3e-18	5.9e-05	73.7
Lipid Metabolism	HDL	rs55730499	6	161005610	T	C	0.017	0.003	2.9e-10	3.2e-05	39.7
Lipid Metabolism	HDL	rs11751347	6	161092438	T	C	-0.059	0.002	1.9e-139	5.1e-04	631.9
Lipid Metabolism	HDL	rs783152	6	161227208	T	G	0.027	0.005	1.4e-08	2.6e-05	32.2
Lipid Metabolism	HDL	rs35537826	6	163145867	A	G	0.014	0.002	3.2e-10	3.2e-05	39.6
Lipid Metabolism	HDL	rs4277989	6	163744748	A	G	-0.011	0.001	2.4e-15	5.1e-05	62.7
Lipid Metabolism	HDL	rs2461722	6	166309288	C	T	0.009	0.002	4.9e-09	2.7e-05	34.2
Lipid Metabolism	HDL	rs239933	6	167413230	G	A	-0.008	0.001	2.2e-08	2.5e-05	31.3
Lipid Metabolism	HDL	rs1007766	7	1034903	A	G	-0.012	0.001	3.1e-17	5.8e-05	71.3
Lipid Metabolism	HDL	rs34409228	7	1102674	T	C	0.028	0.002	3.6e-41	1.5e-04	180.6
Lipid Metabolism	HDL	rs77523736	7	2071957	A	G	0.093	0.017	3.1e-08	3.9e-05	30.6
Lipid Metabolism	HDL	rs139753251	7	6449794	G	A	0.050	0.009	5.9e-09	2.8e-05	33.9
Lipid Metabolism	HDL	rs4724804	7	6453845	A	G	0.029	0.002	7.3e-69	2.5e-04	307.6
Lipid Metabolism	HDL	rs71533356	7	7237892	A	G	-0.012	0.002	3.6e-10	3.6e-05	39.3
Lipid Metabolism	HDL	rs17650302	7	12143138	T	C	0.013	0.002	3.3e-10	3.2e-05	39.5
Lipid Metabolism	HDL	rs1990622	7	12283787	G	A	-0.012	0.001	1.1e-18	6.3e-05	77.9
Lipid Metabolism	HDL	rs28273	7	15901834	G	A	0.009	0.001	7.6e-11	3.4e-05	42.4
Lipid Metabolism	HDL	rs4410790	7	17284577	C	T	-0.012	0.001	3.1e-18	6.1e-05	75.8
Lipid Metabolism	HDL	rs6461354	7	17914600	T	C	-0.023	0.001	6.9e-59	2.1e-04	261.8
Lipid Metabolism	HDL	rs10950692	7	18017502	A	G	-0.014	0.002	9.8e-12	3.7e-05	46.4
Lipid Metabolism	HDL	rs56053938	7	22777188	G	A	-0.014	0.002	2.1e-09	2.9e-05	35.8
Lipid Metabolism	HDL	rs11979000	7	26150470	G	A	0.013	0.002	6.9e-14	4.5e-05	56.1
Lipid Metabolism	HDL	rs112078038	7	26422042	T	C	-0.016	0.002	4.3e-16	5.4e-05	66.1
Lipid Metabolism	HDL	rs75458722	7	28655748	A	T	-0.015	0.003	4.3e-08	2.4e-05	30.0
Lipid Metabolism	HDL	rs58609917	7	35666267	A	C	-0.009	0.001	6.0e-10	3.1e-05	38.3
Lipid Metabolism	HDL	rs66763009	7	36193142	G	T	-0.015	0.001	2.6e-28	9.9e-05	121.8
Lipid Metabolism	HDL	rs4723739	7	38270723	G	A	0.010	0.001	4.5e-13	4.2e-05	52.4
Lipid Metabolism	HDL	rs62460516	7	44866362	C	T	-0.016	0.003	1.2e-08	2.6e-05	32.5
Lipid Metabolism	HDL	rs10249036	7	46646735	G	A	0.008	0.001	1.0e-09	3.0e-05	37.3
Lipid Metabolism	HDL	rs876037	7	50308692	A	T	0.015	0.001	4.3e-25	8.7e-05	107.1
Lipid Metabolism	HDL	rs35867424	7	72032358	A	G	0.025	0.004	3.1e-08	2.5e-05	30.6
Lipid Metabolism	HDL	rs42125	7	72823777	G	A	-0.045	0.006	2.2e-16	5.6e-05	67.4
Lipid Metabolism	HDL	rs142001788	7	72833107	T	C	-0.054	0.009	2.3e-10	3.4e-05	40.2
Lipid Metabolism	HDL	rs55747707	7	73037366	A	G	0.038	0.002	7.9e-106	3.9e-04	477.4
Lipid Metabolism	HDL	rs17851629	7	73932560	G	A	-0.010	0.002	2.4e-09	2.9e-05	35.6
Lipid Metabolism	HDL	rs112924314	7	74361240	T	C	-0.024	0.003	3.9e-13	4.3e-05	52.7
Lipid Metabolism	HDL	rs41301433	7	75615439	G	C	0.068	0.011	2.3e-09	2.9e-05	35.7
Lipid Metabolism	HDL	rs10247757	7	79853447	A	C	0.015	0.002	4.8e-12	3.9e-05	47.8
Lipid Metabolism	HDL	rs7807607	7	80226885	T	C	0.011	0.001	1.5e-14	4.8e-05	59.1
Lipid Metabolism	HDL	rs854562	7	94947969	T	C	-0.011	0.001	5.3e-13	4.2e-05	52.1
Lipid Metabolism	HDL	rs183892429	7	95023483	T	A	0.052	0.009	8.6e-09	2.7e-05	33.1
Lipid Metabolism	HDL	rs13226502	7	100506381	T	C	0.011	0.002	6.1e-09	2.7e-05	33.8
Lipid Metabolism	HDL	rs1637252	7	101736163	A	C	-0.009	0.001	4.4e-10	3.2e-05	38.9
Lipid Metabolism	HDL	rs257377	7	106801088	T	G	0.014	0.002	4.0e-16	5.3e-05	66.2
Lipid Metabolism	HDL	rs79597516	7	107619540	A	G	0.013	0.002	2.7e-08	2.5e-05	30.9
Lipid Metabolism	HDL	rs13246825	7	116789048	C	T	-0.011	0.002	1.4e-08	2.6e-05	32.1
Lipid Metabolism	HDL	rs10245477	7	116983083	C	T	-0.012	0.001	5.0e-16	5.4e-05	65.8
Lipid Metabolism	HDL	rs920803	7	121970319	A	C	0.009	0.002	4.3e-09	2.8e-05	34.5
Lipid Metabolism	HDL	rs290804	7	130420872	G	A	-0.012	0.002	4.4e-11	3.5e-05	43.4

Phenotype	Trait	SNP	Chr	Position (GRCh37/hg19)	EA	NEA	BETA	SE	P-value	R2	F
Lipid Metabolism	HDL	rs11762784	7	130424646	A	G	0.030	0.001	8.6e-101	3.7e-04	454.2
Lipid Metabolism	HDL	rs11970875	7	130884601	C	T	-0.008	0.001	1.1e-08	2.6e-05	32.7
Lipid Metabolism	HDL	rs357422	7	138033197	A	G	0.011	0.002	3.7e-12	4.0e-05	48.3
Lipid Metabolism	HDL	rs73154134	7	139039951	A	G	0.012	0.002	1.0e-12	4.1e-05	50.8
Lipid Metabolism	HDL	rs62490267	7	149238823	C	T	-0.008	0.002	3.6e-08	2.5e-05	30.4
Lipid Metabolism	HDL	rs35241484	7	150216171	G	T	-0.017	0.002	1.6e-26	9.2e-05	113.5
Lipid Metabolism	HDL	rs7794796	7	150540196	T	C	-0.017	0.001	2.7e-32	1.1e-04	139.9
Lipid Metabolism	HDL	rs740954	7	150598492	C	T	0.008	0.001	4.3e-09	2.8e-05	34.5
Lipid Metabolism	HDL	rs62489449	8	4824509	C	T	-0.014	0.002	3.5e-08	2.4e-05	30.4
Lipid Metabolism	HDL	rs11783088	8	6620392	G	A	-0.012	0.002	1.1e-13	4.5e-05	55.2
Lipid Metabolism	HDL	rs558115450	8	9162276	T	C	-0.100	0.018	1.2e-08	2.8e-05	32.5
Lipid Metabolism	HDL	rs2169387	8	9181395	G	A	0.089	0.002	1.0e-200	1.2e-03	1467.0
Lipid Metabolism	HDL	rs28396619	8	9197007	G	A	0.019	0.003	6.8e-14	4.5e-05	56.1
Lipid Metabolism	HDL	rs17729883	8	9256631	C	T	-0.016	0.002	7.7e-28	9.7e-05	119.6
Lipid Metabolism	HDL	rs141415741	8	9327336	A	G	-0.097	0.016	2.3e-09	3.0e-05	35.7
Lipid Metabolism	HDL	rs186245508	8	9443064	C	A	-0.061	0.008	4.0e-13	4.3e-05	52.6
Lipid Metabolism	HDL	rs7827623	8	10540931	G	T	0.012	0.002	1.2e-11	3.8e-05	45.9
Lipid Metabolism	HDL	rs28588745	8	10647044	T	A	-0.018	0.002	1.7e-25	8.8e-05	108.9
Lipid Metabolism	HDL	rs2645400	8	11609416	G	T	0.009	0.001	1.8e-09	2.9e-05	36.2
Lipid Metabolism	HDL	rs12680202	8	12617942	A	T	-0.009	0.002	1.4e-08	2.7e-05	32.1
Lipid Metabolism	HDL	rs6984721	8	13510305	T	C	0.008	0.001	4.3e-09	2.8e-05	34.5
Lipid Metabolism	HDL	rs406329	8	17793942	C	T	-0.010	0.001	1.2e-11	3.7e-05	46.0
Lipid Metabolism	HDL	rs144054647	8	19122012	G	A	-0.063	0.011	2.2e-08	2.6e-05	31.3
Lipid Metabolism	HDL	rs532473356	8	19373467	G	A	0.074	0.012	2.3e-09	3.3e-05	35.7
Lipid Metabolism	HDL	rs181056544	8	19379044	G	A	-0.037	0.007	2.3e-08	2.5e-05	31.2
Lipid Metabolism	HDL	rs539412189	8	19458595	G	A	-0.115	0.018	4.8e-11	5.4e-05	43.3
Lipid Metabolism	HDL	rs555362190	8	19532212	A	G	-0.211	0.016	7.5e-39	1.6e-04	170.0
Lipid Metabolism	HDL	rs36082703	8	19597124	G	A	-0.123	0.016	6.4e-14	4.8e-05	56.3
Lipid Metabolism	HDL	rs76962075	8	19623054	T	C	0.153	0.025	1.3e-09	3.5e-05	36.9
Lipid Metabolism	HDL	rs111251122	8	19623795	G	A	-0.144	0.007	5.7e-84	3.1e-04	377.0
Lipid Metabolism	HDL	rs558679944	8	19634363	G	A	0.134	0.012	2.8e-28	1.1e-04	121.6
Lipid Metabolism	HDL	rs186197101	8	19635801	C	A	-0.173	0.010	5.1e-69	2.5e-04	308.3
Lipid Metabolism	HDL	rs144473554	8	19639879	A	T	0.108	0.013	1.0e-15	7.6e-05	64.4
Lipid Metabolism	HDL	rs140014394	8	19673994	T	C	0.116	0.016	1.5e-13	4.7e-05	54.6
Lipid Metabolism	HDL	rs184464807	8	19683122	C	G	-0.120	0.013	1.5e-21	7.5e-05	90.9
Lipid Metabolism	HDL	rs573875923	8	19684438	C	T	0.280	0.044	2.2e-10	1.1e-04	40.3
Lipid Metabolism	HDL	rs190341366	8	19705250	A	G	0.156	0.025	7.6e-10	3.8e-05	37.9
Lipid Metabolism	HDL	rs145964918	8	19717989	C	T	-0.227	0.031	3.1e-13	1.4e-04	53.2
Lipid Metabolism	HDL	rs545516154	8	19729354	C	T	-0.302	0.036	4.1e-17	1.9e-04	70.7
Lipid Metabolism	HDL	rs73607783	8	19742204	A	T	0.067	0.002	1.0e-200	1.2e-03	1486.1
Lipid Metabolism	HDL	rs373010541	8	19771395	T	C	-0.112	0.015	9.5e-14	4.7e-05	55.5
Lipid Metabolism	HDL	rs117521017	8	19776009	T	C	0.112	0.007	2.2e-52	1.9e-04	232.0
Lipid Metabolism	HDL	rs536076610	8	19807477	C	T	-0.482	0.047	6.0e-25	2.8e-04	106.4
Lipid Metabolism	HDL	rs78326602	8	19809220	A	G	-0.062	0.010	2.5e-09	2.9e-05	35.5
Lipid Metabolism	HDL	rs144578061	8	19813878	C	A	0.087	0.008	5.6e-27	9.4e-05	115.7
Lipid Metabolism	HDL	rs3289	8	19823192	C	T	-0.169	0.004	1.0e-200	1.2e-03	1492.9
Lipid Metabolism	HDL	rs1372343	8	19871320	T	C	0.059	0.001	1.0e-200	1.4e-03	1794.8
Lipid Metabolism	HDL	rs551447383	8	19880070	C	T	0.192	0.023	1.0e-16	1.8e-04	68.9
Lipid Metabolism	HDL	rs189848936	8	19883934	C	A	-0.074	0.009	2.5e-15	5.1e-05	62.6
Lipid Metabolism	HDL	rs555211558	8	19893301	A	G	-0.109	0.015	2.3e-13	6.4e-05	53.7
Lipid Metabolism	HDL	rs75846101	8	19960416	A	G	-0.034	0.005	6.5e-10	3.1e-05	38.2
Lipid Metabolism	HDL	rs553001378	8	19961375	C	T	0.140	0.025	1.5e-08	3.7e-05	32.1
Lipid Metabolism	HDL	rs181703090	8	19970086	T	C	-0.053	0.007	4.6e-14	4.6e-05	56.9
Lipid Metabolism	HDL	rs10107837	8	19988676	G	C	-0.020	0.002	2.4e-27	1.0e-04	117.4
Lipid Metabolism	HDL	rs558905601	8	20013731	C	T	-0.333	0.058	1.1e-08	9.1e-05	32.7
Lipid Metabolism	HDL	rs186859575	8	20014706	C	T	0.110	0.016	9.2e-12	4.1e-05	46.5

Phenotype	Trait	SNP	Chr	Position (GRCh37/hg19)	EA	NEA	BETA	SE	P-value	R2	F
Lipid Metabolism	HDL	rs182619883	8	20025108	A	G	0.092	0.010	1.0e-20	7.1e-05	87.1
Lipid Metabolism	HDL	rs147972269	8	20026384	C	T	0.086	0.010	1.9e-18	6.3e-05	76.8
Lipid Metabolism	HDL	rs375092514	8	20061636	T	C	0.079	0.011	9.6e-14	4.5e-05	55.4
Lipid Metabolism	HDL	rs531154338	8	20120775	C	A	0.081	0.014	3.9e-09	3.4e-05	34.7
Lipid Metabolism	HDL	rs549100850	8	20193330	G	A	-0.660	0.114	8.0e-09	5.8e-05	33.3
Lipid Metabolism	HDL	rs528984559	8	20436207	A	T	-0.319	0.058	4.7e-08	4.9e-05	29.8
Lipid Metabolism	HDL	rs6983481	8	23609009	T	G	0.009	0.002	1.3e-08	2.6e-05	32.3
Lipid Metabolism	HDL	rs11992444	8	25464690	T	G	0.012	0.002	2.2e-13	5.1e-05	53.8
Lipid Metabolism	HDL	rs7003404	8	25871350	T	C	-0.012	0.002	7.5e-12	3.8e-05	46.9
Lipid Metabolism	HDL	rs2874670	8	26245658	A	G	-0.008	0.001	2.3e-08	2.5e-05	31.2
Lipid Metabolism	HDL	rs7824992	8	29391794	T	C	-0.008	0.001	5.9e-09	2.7e-05	33.9
Lipid Metabolism	HDL	rs78488552	8	31012237	G	C	0.060	0.011	1.8e-08	2.7e-05	31.7
Lipid Metabolism	HDL	rs7835078	8	34515194	G	A	0.011	0.002	2.7e-12	3.9e-05	48.9
Lipid Metabolism	HDL	rs10087804	8	36858140	G	C	-0.013	0.002	8.7e-13	4.1e-05	51.1
Lipid Metabolism	HDL	rs10958704	8	38328302	G	A	-0.009	0.001	1.8e-11	3.6e-05	45.2
Lipid Metabolism	HDL	rs6999716	8	60651271	A	G	-0.011	0.002	1.2e-11	3.7e-05	46.0
Lipid Metabolism	HDL	rs1217097	8	64623330	G	A	0.010	0.002	3.1e-11	3.6e-05	44.1
Lipid Metabolism	HDL	rs705987	8	64739741	A	C	0.011	0.001	1.4e-15	5.1e-05	63.8
Lipid Metabolism	HDL	rs7003091	8	71194263	G	A	-0.024	0.002	9.1e-26	8.8e-05	110.1
Lipid Metabolism	HDL	rs28471687	8	72459582	G	A	-0.022	0.003	2.7e-16	5.4e-05	67.0
Lipid Metabolism	HDL	rs900053	8	74690315	G	A	-0.013	0.002	2.2e-09	2.9e-05	35.8
Lipid Metabolism	HDL	rs16909263	8	82414690	C	T	0.011	0.002	9.0e-09	2.7e-05	33.0
Lipid Metabolism	HDL	rs4286925	8	93237876	G	A	0.009	0.001	1.7e-09	2.9e-05	36.3
Lipid Metabolism	HDL	rs2247355	8	103876780	T	C	0.013	0.002	1.6e-13	4.4e-05	54.4
Lipid Metabolism	HDL	rs2941657	8	106372180	G	A	-0.011	0.001	3.5e-14	4.6e-05	57.4
Lipid Metabolism	HDL	rs4326410	8	116593582	T	C	0.032	0.001	4.5e-120	4.4e-04	542.8
Lipid Metabolism	HDL	rs12549000	8	118881908	A	T	-0.013	0.002	4.6e-09	2.8e-05	34.3
Lipid Metabolism	HDL	rs10955991	8	121867780	C	T	-0.020	0.001	2.1e-40	1.4e-04	177.1
Lipid Metabolism	HDL	rs114304704	8	121904466	A	G	0.058	0.008	6.8e-13	4.2e-05	51.6
Lipid Metabolism	HDL	rs10097644	8	121926410	C	T	0.010	0.001	7.8e-13	4.1e-05	51.3
Lipid Metabolism	HDL	rs17319046	8	126350794	G	T	0.020	0.003	1.4e-12	4.1e-05	50.2
Lipid Metabolism	HDL	rs72647336	8	126445055	A	G	-0.050	0.003	8.5e-49	1.8e-04	215.5
Lipid Metabolism	HDL	rs76820699	8	126468246	T	C	0.049	0.007	7.7e-11	3.4e-05	42.3
Lipid Metabolism	HDL	rs2980888	8	126507308	C	T	0.043	0.002	6.1e-176	6.4e-04	799.8
Lipid Metabolism	HDL	rs114443260	8	126630967	T	C	-0.019	0.002	2.8e-26	1.2e-04	112.5
Lipid Metabolism	HDL	rs60987469	8	144303753	T	C	0.028	0.002	9.9e-55	2.0e-04	242.8
Lipid Metabolism	HDL	rs28523940	8	144360902	T	C	0.012	0.001	4.6e-18	6.2e-05	75.1
Lipid Metabolism	HDL	rs4741016	9	1036132	C	T	-0.014	0.002	1.5e-18	6.3e-05	77.2
Lipid Metabolism	HDL	rs2219143	9	2622278	A	G	0.008	0.001	3.6e-08	2.5e-05	30.3
Lipid Metabolism	HDL	rs10511582	9	13618117	C	G	-0.010	0.002	5.4e-09	2.7e-05	34.0
Lipid Metabolism	HDL	rs543458	9	14068327	C	A	0.013	0.002	3.0e-13	4.3e-05	53.2
Lipid Metabolism	HDL	rs113261688	9	15255361	T	C	0.015	0.003	3.0e-08	2.5e-05	30.7
Lipid Metabolism	HDL	rs686030	9	15304782	A	C	0.050	0.002	4.6e-140	5.1e-04	634.8
Lipid Metabolism	HDL	rs7868583	9	16906359	A	G	0.010	0.001	4.1e-12	3.9e-05	48.1
Lipid Metabolism	HDL	rs72698383	9	19256362	T	C	0.018	0.003	2.9e-11	3.6e-05	44.3
Lipid Metabolism	HDL	rs2150854	9	28411949	T	G	-0.012	0.001	9.9e-17	5.5e-05	69.0
Lipid Metabolism	HDL	rs11795343	9	32523737	C	T	0.010	0.001	7.1e-13	4.1e-05	51.5
Lipid Metabolism	HDL	rs10217770	9	33146393	A	C	-0.008	0.001	2.1e-08	2.5e-05	31.4
Lipid Metabolism	HDL	rs10971981	9	34186588	G	A	0.009	0.001	2.3e-11	3.6e-05	44.7
Lipid Metabolism	HDL	rs11138313	9	82240968	G	A	0.014	0.002	1.3e-09	3.0e-05	36.9
Lipid Metabolism	HDL	rs1831757	9	86509330	C	T	0.009	0.002	5.4e-09	2.8e-05	34.0
Lipid Metabolism	HDL	rs41310053	9	88940359	T	C	0.064	0.009	6.5e-12	3.9e-05	47.2
Lipid Metabolism	HDL	rs11521719	9	88978487	T	C	0.010	0.002	1.7e-08	2.6e-05	31.8
Lipid Metabolism	HDL	rs76226649	9	93964150	A	G	-0.015	0.002	1.6e-10	3.3e-05	40.9
Lipid Metabolism	HDL	rs7850075	9	95561537	T	C	-0.011	0.002	1.5e-08	2.6e-05	32.0
Lipid Metabolism	HDL	rs10984405	9	100690555	G	A	0.009	0.002	3.0e-08	2.8e-05	30.7

Phenotype	Trait	SNP	Chr	Position (GRCh37/hg19)	EA	NEA	BETA	SE	P-value	R2	F
Lipid Metabolism	HDL	rs185512965	9	102834867	T	C	-0.142	0.025	2.4e-08	4.4e-05	31.1
Lipid Metabolism	HDL	rs186966184	9	104804644	G	T	0.278	0.044	3.5e-10	7.2e-05	39.4
Lipid Metabolism	HDL	rs117235574	9	106495648	G	A	-0.101	0.015	2.6e-11	5.8e-05	44.5
Lipid Metabolism	HDL	rs111848035	9	107004972	T	C	0.060	0.011	2.0e-08	2.6e-05	31.5
Lipid Metabolism	HDL	rs181513740	9	107049208	A	G	-0.542	0.082	3.9e-11	7.8e-05	43.7
Lipid Metabolism	HDL	rs146899053	9	107065930	A	T	-0.089	0.015	1.7e-09	4.2e-05	36.2
Lipid Metabolism	HDL	rs117609250	9	107235403	G	A	0.053	0.007	5.7e-14	4.6e-05	56.5
Lipid Metabolism	HDL	rs142260797	9	107273774	G	A	-0.104	0.011	8.2e-21	9.2e-05	87.6
Lipid Metabolism	HDL	rs145673272	9	107336060	A	G	0.088	0.016	2.7e-08	2.6e-05	30.9
Lipid Metabolism	HDL	rs181721528	9	107394320	G	A	-0.364	0.028	5.5e-40	2.4e-04	175.2
Lipid Metabolism	HDL	rs72730693	9	107435135	G	T	0.026	0.005	7.1e-09	2.7e-05	33.5
Lipid Metabolism	HDL	rs559092527	9	107437623	G	C	-0.268	0.024	6.3e-30	1.7e-04	129.1
Lipid Metabolism	HDL	rs546254959	9	107520742	C	G	-0.169	0.028	1.2e-09	4.3e-05	37.0
Lipid Metabolism	HDL	rs41277761	9	107533232	A	C	-0.080	0.014	1.5e-08	2.7e-05	32.1
Lipid Metabolism	HDL	rs2777800	9	107563269	C	T	0.035	0.002	5.1e-85	3.1e-04	381.8
Lipid Metabolism	HDL	rs529137214	9	107581732	T	C	-0.145	0.020	1.1e-13	1.4e-04	55.1
Lipid Metabolism	HDL	rs56104133	9	107586671	T	C	0.056	0.009	8.5e-10	3.1e-05	37.7
Lipid Metabolism	HDL	rs3824477	9	107588328	A	G	0.103	0.004	1.1e-147	5.4e-04	669.7
Lipid Metabolism	HDL	rs35819696	9	107589246	G	T	-0.074	0.013	8.3e-09	2.8e-05	33.2
Lipid Metabolism	HDL	rs9282543	9	107599376	G	A	-0.067	0.012	4.1e-08	2.8e-05	30.1
Lipid Metabolism	HDL	rs535908385	9	107609329	A	G	-0.217	0.026	9.7e-17	8.6e-05	69.0
Lipid Metabolism	HDL	rs188308962	9	107620797	G	A	-0.482	0.017	8.7e-185	7.1e-04	840.4
Lipid Metabolism	HDL	rs150729477	9	107622941	C	G	-0.228	0.031	1.2e-13	1.4e-04	54.9
Lipid Metabolism	HDL	rs145183203	9	107646756	A	G	-0.204	0.021	2.8e-23	9.6e-05	98.8
Lipid Metabolism	HDL	rs2740488	9	107661742	C	A	-0.069	0.002	1.0e-200	1.5e-03	1925.7
Lipid Metabolism	HDL	rs1800976	9	107690709	G	C	0.021	0.001	2.1e-52	1.9e-04	232.1
Lipid Metabolism	HDL	rs188646957	9	107715216	G	A	-0.157	0.025	1.7e-10	5.6e-05	40.8
Lipid Metabolism	HDL	rs181984180	9	107770247	G	A	0.065	0.011	3.5e-09	2.9e-05	34.9
Lipid Metabolism	HDL	rs192956846	9	107829435	A	G	-0.118	0.016	4.9e-13	4.6e-05	52.3
Lipid Metabolism	HDL	rs535477361	9	107848158	G	A	0.099	0.013	4.3e-15	6.1e-05	61.6
Lipid Metabolism	HDL	rs532347616	9	107905414	A	G	-0.871	0.118	1.5e-13	1.5e-04	54.6
Lipid Metabolism	HDL	rs112341819	9	108672373	C	G	-0.025	0.005	3.9e-08	2.4e-05	30.2
Lipid Metabolism	HDL	rs192266820	9	109468236	T	C	-0.172	0.031	2.9e-08	4.4e-05	30.7
Lipid Metabolism	HDL	rs1880145	9	110469944	T	C	-0.008	0.001	1.1e-08	2.6e-05	32.6
Lipid Metabolism	HDL	rs12344809	9	112533895	A	G	-0.009	0.002	1.2e-08	2.6e-05	32.5
Lipid Metabolism	HDL	rs2150052	9	113945067	T	A	0.008	0.001	1.5e-08	2.6e-05	32.1
Lipid Metabolism	HDL	rs10733608	9	117148430	T	G	0.012	0.001	1.1e-17	5.9e-05	73.4
Lipid Metabolism	HDL	rs4837374	9	119154616	G	C	-0.009	0.002	4.7e-08	2.4e-05	29.8
Lipid Metabolism	HDL	rs10760107	9	123383630	C	T	-0.009	0.002	1.2e-09	3.0e-05	37.0
Lipid Metabolism	HDL	rs4620350	9	126539944	G	A	-0.020	0.003	2.1e-13	4.3e-05	54.0
Lipid Metabolism	HDL	rs3962617	9	128723231	T	A	-0.011	0.002	4.2e-10	4.2e-05	39.0
Lipid Metabolism	HDL	rs6478851	9	131561110	A	G	-0.011	0.002	1.9e-12	4.0e-05	49.6
Lipid Metabolism	HDL	rs1147369	9	132235522	G	C	0.009	0.001	2.1e-10	3.3e-05	40.4
Lipid Metabolism	HDL	rs10901305	9	133789707	G	A	-0.008	0.001	4.5e-09	2.8e-05	34.4
Lipid Metabolism	HDL	rs2519093	9	136141870	T	C	0.017	0.002	1.8e-22	7.6e-05	95.1
Lipid Metabolism	HDL	rs35182096	9	137268682	T	C	-0.009	0.002	3.1e-08	2.5e-05	30.6
Lipid Metabolism	HDL	rs12413228	10	849230	A	C	0.008	0.001	4.0e-08	2.4e-05	30.2
Lipid Metabolism	HDL	rs3750571	10	5247434	A	C	-0.014	0.002	2.3e-13	4.3e-05	53.7
Lipid Metabolism	HDL	rs2152960	10	8447786	C	T	-0.008	0.002	2.3e-08	2.6e-05	31.3
Lipid Metabolism	HDL	rs7903137	10	17259567	T	C	0.014	0.001	1.1e-22	7.9e-05	96.1
Lipid Metabolism	HDL	rs12358424	10	33641149	G	T	-0.026	0.002	1.2e-25	8.8e-05	109.5
Lipid Metabolism	HDL	rs16934748	10	33970319	C	T	-0.014	0.002	2.6e-12	3.9e-05	49.0
Lipid Metabolism	HDL	rs55665473	10	46022005	A	G	0.026	0.002	6.8e-60	2.1e-04	266.4
Lipid Metabolism	HDL	rs11592181	10	51582686	A	G	0.012	0.002	8.3e-10	3.1e-05	37.7
Lipid Metabolism	HDL	rs2242206	10	61414011	T	G	-0.010	0.002	1.0e-10	3.4e-05	41.8
Lipid Metabolism	HDL	rs10821953	10	63828879	A	T	0.010	0.001	1.3e-12	4.0e-05	50.3

Phenotype	Trait	SNP	Chr	Position (GRCh37/hg19)	EA	NEA	BETA	SE	P-value	R2	F
Lipid Metabolism	HDL	rs7916868	10	64988931	T	A	0.011	0.001	2.1e-15	5.1e-05	63.0
Lipid Metabolism	HDL	rs12774848	10	71932436	C	T	-0.009	0.001	2.1e-10	3.2e-05	40.3
Lipid Metabolism	HDL	rs7098444	10	75580042	T	C	-0.009	0.001	5.4e-11	3.5e-05	43.0
Lipid Metabolism	HDL	rs2997754	10	76904137	A	G	-0.009	0.001	1.1e-09	3.0e-05	37.1
Lipid Metabolism	HDL	rs703966	10	80954251	A	G	0.012	0.001	3.2e-17	5.7e-05	71.2
Lipid Metabolism	HDL	rs11201985	10	88097795	A	G	-0.008	0.001	1.9e-09	2.9e-05	36.1
Lipid Metabolism	HDL	rs11186798	10	93773792	A	G	-0.021	0.003	3.5e-13	4.3e-05	52.9
Lipid Metabolism	HDL	rs55843714	10	94822686	T	C	-0.018	0.001	1.4e-38	1.4e-04	168.8
Lipid Metabolism	HDL	rs11187498	10	95290777	C	T	-0.019	0.002	1.4e-23	8.1e-05	100.2
Lipid Metabolism	HDL	rs41290220	10	95348616	G	A	0.016	0.003	1.2e-10	3.3e-05	41.5
Lipid Metabolism	HDL	rs522110	10	99772885	G	A	-0.009	0.001	7.1e-10	3.1e-05	38.0
Lipid Metabolism	HDL	rs2862954	10	101912064	C	T	0.015	0.001	3.0e-26	9.0e-05	112.3
Lipid Metabolism	HDL	rs10787287	10	112647195	T	C	-0.010	0.002	7.0e-09	2.7e-05	33.5
Lipid Metabolism	HDL	rs2803619	10	113934384	C	G	-0.031	0.002	8.6e-90	3.3e-04	403.7
Lipid Metabolism	HDL	rs4573621	10	113987548	G	A	-0.025	0.002	1.4e-45	1.6e-04	200.7
Lipid Metabolism	HDL	rs72823014	10	115786236	A	G	0.031	0.002	1.6e-47	1.7e-04	209.7
Lipid Metabolism	HDL	rs2901114	10	118407702	A	G	-0.012	0.001	1.0e-17	5.9e-05	73.4
Lipid Metabolism	HDL	rs2234962	10	121429633	C	T	0.010	0.002	1.2e-09	3.0e-05	36.9
Lipid Metabolism	HDL	rs12415055	10	122869552	G	A	-0.018	0.002	3.6e-17	5.7e-05	71.0
Lipid Metabolism	HDL	rs10886862	10	122929408	T	C	-0.033	0.005	2.5e-13	4.4e-05	53.5
Lipid Metabolism	HDL	rs10794174	10	126357981	C	T	0.009	0.001	7.5e-11	3.4e-05	42.4
Lipid Metabolism	HDL	rs10901854	10	126733643	C	T	-0.011	0.002	7.0e-13	4.1e-05	51.5
Lipid Metabolism	HDL	rs117739035	11	408174	T	G	-0.028	0.004	2.4e-13	4.5e-05	53.7
Lipid Metabolism	HDL	rs35467761	11	571568	G	A	0.010	0.002	6.3e-09	2.7e-05	33.7
Lipid Metabolism	HDL	rs150004945	11	1051734	T	C	-0.034	0.006	6.1e-09	2.8e-05	33.8
Lipid Metabolism	HDL	rs450244	11	2940492	C	T	0.026	0.002	1.4e-25	8.8e-05	109.4
Lipid Metabolism	HDL	rs72844021	11	3051206	T	A	-0.041	0.006	2.0e-13	4.4e-05	54.0
Lipid Metabolism	HDL	rs2957716	11	10380858	A	G	0.012	0.002	2.6e-16	5.4e-05	67.1
Lipid Metabolism	HDL	rs6486121	11	13355770	T	C	-0.010	0.001	2.1e-11	3.6e-05	44.9
Lipid Metabolism	HDL	rs192849769	11	14451992	C	T	0.184	0.019	1.8e-22	9.1e-05	95.1
Lipid Metabolism	HDL	rs79634051	11	14561945	C	G	0.049	0.005	6.2e-26	9.0e-05	110.9
Lipid Metabolism	HDL	rs12270702	11	14655417	A	G	-0.011	0.002	1.6e-11	3.7e-05	45.4
Lipid Metabolism	HDL	rs61736639	11	14891141	C	G	0.061	0.009	1.0e-11	3.8e-05	46.3
Lipid Metabolism	HDL	rs12286683	11	18348662	T	G	-0.009	0.002	3.7e-09	2.8e-05	34.8
Lipid Metabolism	HDL	rs7944119	11	27722298	T	G	-0.015	0.001	6.7e-25	8.5e-05	106.2
Lipid Metabolism	HDL	rs2035837	11	29200527	C	T	0.012	0.002	9.6e-11	3.4e-05	41.9
Lipid Metabolism	HDL	rs11031793	11	32470828	T	C	-0.012	0.001	3.1e-16	5.4e-05	66.7
Lipid Metabolism	HDL	rs74816520	11	32897280	A	T	0.019	0.003	1.4e-09	2.9e-05	36.7
Lipid Metabolism	HDL	rs11033573	11	36383808	T	C	0.009	0.001	1.1e-09	3.0e-05	37.1
Lipid Metabolism	HDL	rs2863156	11	45188860	A	C	-0.013	0.002	5.0e-10	3.1e-05	38.7
Lipid Metabolism	HDL	rs77403571	11	45913607	A	G	0.036	0.003	5.5e-35	1.2e-04	152.3
Lipid Metabolism	HDL	rs527611410	11	45993577	C	T	0.139	0.020	2.0e-12	6.0e-05	49.4
Lipid Metabolism	HDL	rs188935961	11	46099524	C	A	0.237	0.039	1.0e-09	1.0e-04	37.3
Lipid Metabolism	HDL	rs568132453	11	46129442	G	A	0.163	0.022	3.1e-13	6.8e-05	53.1
Lipid Metabolism	HDL	rs562763773	11	46217988	A	G	0.131	0.021	3.1e-10	4.8e-05	39.6
Lipid Metabolism	HDL	rs567226655	11	46275130	G	A	0.143	0.021	1.2e-11	5.7e-05	46.0
Lipid Metabolism	HDL	rs530063207	11	46660542	T	A	0.267	0.034	3.0e-15	7.1e-05	62.2
Lipid Metabolism	HDL	rs7105052	11	46808600	C	T	-0.021	0.002	1.3e-17	5.9e-05	72.9
Lipid Metabolism	HDL	rs184787588	11	46841661	A	G	0.425	0.038	1.6e-28	1.5e-04	122.7
Lipid Metabolism	HDL	rs11607154	11	46859315	T	G	-0.042	0.008	3.1e-08	2.5e-05	30.6
Lipid Metabolism	HDL	rs566486969	11	47186396	A	G	0.460	0.042	1.6e-27	2.2e-04	118.1
Lipid Metabolism	HDL	rs542644218	11	47210679	T	C	0.398	0.040	1.5e-23	1.6e-04	100.0
Lipid Metabolism	HDL	rs2279239	11	47281780	C	T	0.042	0.002	5.9e-171	6.2e-04	776.8
Lipid Metabolism	HDL	rs145758003	11	47414372	A	G	0.279	0.019	2.0e-49	2.8e-04	218.4
Lipid Metabolism	HDL	rs147216895	11	47431477	T	C	0.246	0.015	6.7e-59	2.3e-04	261.9
Lipid Metabolism	HDL	rs566759374	11	47685822	C	A	0.316	0.039	9.2e-16	9.3e-05	64.6

Phenotype	Trait	SNP	Chr	Position (GRCh37/hg19)	EA	NEA	BETA	SE	P-value	R2	F
Lipid Metabolism	HDL	rs553237375	11	48279069	C	T	0.271	0.041	4.9e-11	7.1e-05	43.2
Lipid Metabolism	HDL	rs148474158	11	48306754	T	C	-0.029	0.004	6.2e-15	5.0e-05	60.8
Lipid Metabolism	HDL	rs553528877	11	48416216	G	A	0.033	0.006	3.9e-08	2.9e-05	30.2
Lipid Metabolism	HDL	rs56236719	11	48427751	A	C	-0.034	0.005	5.6e-10	3.5e-05	38.5
Lipid Metabolism	HDL	rs145276599	11	48444711	C	T	0.045	0.002	3.1e-85	3.1e-04	382.8
Lipid Metabolism	HDL	rs117119710	11	48513033	G	A	-0.044	0.004	5.0e-23	7.9e-05	97.6
Lipid Metabolism	HDL	rs553343968	11	48965035	A	G	0.210	0.016	2.8e-40	1.6e-04	176.5
Lipid Metabolism	HDL	rs117029543	11	49688313	G	A	-0.029	0.005	3.0e-08	2.5e-05	30.7
Lipid Metabolism	HDL	rs3884997	11	49765667	A	G	0.040	0.002	8.9e-64	2.3e-04	284.3
Lipid Metabolism	HDL	rs181964456	11	49913973	A	G	-0.036	0.004	8.7e-17	5.6e-05	69.3
Lipid Metabolism	HDL	rs180914872	11	50164443	T	G	0.273	0.022	1.9e-35	2.1e-04	154.4
Lipid Metabolism	HDL	rs117794669	11	50230789	C	G	0.112	0.013	9.0e-18	8.1e-05	73.7
Lipid Metabolism	HDL	rs12420650	11	51193615	T	G	0.035	0.002	6.3e-51	1.8e-04	225.3
Lipid Metabolism	HDL	rs140827054	11	51471514	T	C	-0.033	0.004	1.4e-14	4.8e-05	59.2
Lipid Metabolism	HDL	rs189355064	11	51511244	A	T	0.197	0.022	7.1e-20	1.0e-04	83.3
Lipid Metabolism	HDL	rs77584654	11	55095202	G	C	-0.036	0.005	1.6e-14	5.8e-05	59.0
Lipid Metabolism	HDL	rs11229252	11	55129640	T	C	0.039	0.002	2.6e-62	2.2e-04	277.5
Lipid Metabolism	HDL	rs575821746	11	55915768	T	C	0.078	0.012	9.5e-11	4.3e-05	41.9
Lipid Metabolism	HDL	rs7124574	11	56137924	A	G	-0.033	0.002	3.4e-51	1.8e-04	226.5
Lipid Metabolism	HDL	rs79209747	11	56290497	T	C	-0.019	0.003	1.1e-08	2.6e-05	32.6
Lipid Metabolism	HDL	rs116875968	11	56334924	C	T	-0.037	0.005	1.5e-12	4.0e-05	50.1
Lipid Metabolism	HDL	rs184399296	11	56457704	A	G	0.171	0.029	5.4e-09	1.2e-04	34.0
Lipid Metabolism	HDL	rs531195033	11	56625398	G	A	0.148	0.024	4.7e-10	4.5e-05	38.8
Lipid Metabolism	HDL	rs77916918	11	56628086	A	G	0.030	0.004	1.1e-13	4.5e-05	55.3
Lipid Metabolism	HDL	rs10792091	11	57119156	C	T	-0.013	0.002	7.3e-11	3.4e-05	42.4
Lipid Metabolism	HDL	rs577363352	11	57437031	T	C	0.099	0.018	2.7e-08	2.7e-05	30.9
Lipid Metabolism	HDL	rs12790710	11	58064160	G	A	0.019	0.003	4.0e-13	4.2e-05	52.6
Lipid Metabolism	HDL	rs187401202	11	59552625	T	G	-0.201	0.037	4.3e-08	5.8e-05	30.0
Lipid Metabolism	HDL	rs572537530	11	61529656	C	G	-0.044	0.008	3.4e-08	2.5e-05	30.5
Lipid Metabolism	HDL	rs174562	11	61585144	G	A	-0.046	0.001	1.0e-200	8.1e-04	1004.0
Lipid Metabolism	HDL	rs79303190	11	61618016	C	T	-0.029	0.003	2.2e-20	6.9e-05	85.6
Lipid Metabolism	HDL	rs174620	11	61629747	G	A	-0.018	0.001	1.0e-39	1.4e-04	174.0
Lipid Metabolism	HDL	rs11231694	11	63862882	C	G	-0.040	0.003	6.1e-41	1.4e-04	179.5
Lipid Metabolism	HDL	rs112868502	11	65074227	A	G	0.027	0.004	7.8e-12	3.8e-05	46.8
Lipid Metabolism	HDL	rs553933843	11	65256987	A	C	-0.098	0.016	8.3e-10	4.8e-05	37.7
Lipid Metabolism	HDL	rs373626664	11	65270924	T	C	-0.104	0.016	1.6e-10	3.6e-05	40.9
Lipid Metabolism	HDL	rs10750766	11	65473798	A	C	-0.018	0.002	6.9e-32	1.1e-04	138.1
Lipid Metabolism	HDL	rs507062	11	65661656	C	T	-0.016	0.002	3.7e-19	6.5e-05	80.0
Lipid Metabolism	HDL	rs12796984	11	66735215	A	G	0.023	0.003	4.2e-12	3.9e-05	48.0
Lipid Metabolism	HDL	rs2003892	11	68593929	A	G	-0.014	0.001	3.0e-22	7.7e-05	94.1
Lipid Metabolism	HDL	rs12277335	11	69176561	T	C	0.011	0.002	2.2e-08	2.5e-05	31.3
Lipid Metabolism	HDL	rs17132126	11	73271443	C	T	0.013	0.002	1.3e-08	2.6e-05	32.3
Lipid Metabolism	HDL	rs1219549	11	75454622	C	T	-0.030	0.002	5.6e-61	2.2e-04	271.4
Lipid Metabolism	HDL	rs150303834	11	75509317	A	C	-0.169	0.029	9.5e-09	3.7e-05	32.9
Lipid Metabolism	HDL	rs2282611	11	76154846	G	T	0.009	0.002	1.4e-08	2.6e-05	32.1
Lipid Metabolism	HDL	rs11237204	11	77198233	C	T	-0.011	0.002	2.4e-11	3.6e-05	44.6
Lipid Metabolism	HDL	rs12575636	11	95311260	G	T	-0.012	0.002	4.9e-12	3.8e-05	47.7
Lipid Metabolism	HDL	rs7127524	11	102353192	G	A	-0.008	0.001	2.9e-08	2.5e-05	30.7
Lipid Metabolism	HDL	rs10791660	11	103871039	A	C	-0.013	0.002	9.7e-13	4.1e-05	50.9
Lipid Metabolism	HDL	rs7110856	11	109968072	C	A	-0.016	0.002	1.1e-27	9.6e-05	118.8
Lipid Metabolism	HDL	rs650461	11	111889635	G	C	0.009	0.001	1.5e-09	3.0e-05	36.6
Lipid Metabolism	HDL	rs570135685	11	116378404	G	A	0.076	0.010	1.2e-14	4.9e-05	59.5
Lipid Metabolism	HDL	rs141986148	11	116388618	T	G	-0.365	0.058	2.3e-10	1.1e-04	40.2
Lipid Metabolism	HDL	rs145742932	11	116440470	A	G	-0.113	0.014	2.3e-16	5.8e-05	67.3
Lipid Metabolism	HDL	rs542629847	11	116459399	C	A	0.154	0.012	5.2e-38	1.6e-04	166.1
Lipid Metabolism	HDL	rs554198415	11	116479698	C	A	0.117	0.020	2.2e-09	3.2e-05	35.8

Phenotype	Trait	SNP	Chr	Position (GRCh37/hg19)	EA	NEA	BETA	SE	P-value	R2	F
Lipid Metabolism	HDL	rs58950690	11	116543098	C	T	-0.072	0.003	2.3e-109	4.0e-04	493.7
Lipid Metabolism	HDL	rs186304413	11	116559059	T	C	0.090	0.010	3.0e-18	6.2e-05	75.9
Lipid Metabolism	HDL	rs964184	11	116648917	C	G	0.108	0.002	1.0e-200	2.4e-03	2924.4
Lipid Metabolism	HDL	rs140390701	11	116655859	T	C	0.081	0.010	5.4e-17	5.7e-05	70.2
Lipid Metabolism	HDL	rs143292359	11	116661001	A	G	-0.224	0.035	1.0e-10	4.0e-05	41.8
Lipid Metabolism	HDL	rs45611741	11	116663128	T	C	-0.267	0.009	3.8e-191	7.1e-04	869.7
Lipid Metabolism	HDL	rs138326449	11	116701354	A	G	0.847	0.017	1.0e-200	2.0e-03	2349.3
Lipid Metabolism	HDL	rs189221782	11	116726290	G	A	0.127	0.023	4.0e-08	2.7e-05	30.1
Lipid Metabolism	HDL	rs555934108	11	116729796	A	C	-0.195	0.032	1.5e-09	5.3e-05	36.5
Lipid Metabolism	HDL	rs187020791	11	116737570	G	C	0.283	0.035	1.3e-15	9.3e-05	63.9
Lipid Metabolism	HDL	rs186714282	11	116742483	G	A	0.078	0.009	2.4e-18	6.2e-05	76.3
Lipid Metabolism	HDL	rs112354884	11	116856464	C	T	0.084	0.006	2.5e-39	1.4e-04	172.2
Lipid Metabolism	HDL	rs548145542	11	116943893	T	C	0.819	0.097	2.3e-17	1.9e-04	71.9
Lipid Metabolism	HDL	rs192737091	11	117018265	G	T	0.184	0.033	3.4e-08	3.8e-05	30.5
Lipid Metabolism	HDL	rs145158897	11	117052799	G	A	0.085	0.012	8.4e-12	4.0e-05	46.7
Lipid Metabolism	HDL	rs59781045	11	117095283	T	C	0.080	0.003	1.0e-189	6.9e-04	863.1
Lipid Metabolism	HDL	rs188307193	11	117206155	A	G	0.109	0.010	1.2e-26	9.3e-05	114.2
Lipid Metabolism	HDL	rs535521501	11	117209054	C	T	-0.116	0.018	2.0e-10	5.0e-05	40.4
Lipid Metabolism	HDL	rs184964322	11	117216224	G	A	0.971	0.170	1.2e-08	8.7e-05	32.6
Lipid Metabolism	HDL	rs3781981	11	117256436	C	T	-0.013	0.001	1.8e-19	6.6e-05	81.4
Lipid Metabolism	HDL	rs529972317	11	117262144	A	G	-0.367	0.037	9.7e-24	1.1e-04	100.9
Lipid Metabolism	HDL	rs553043145	11	117321657	T	C	-0.263	0.038	3.3e-12	1.3e-04	48.5
Lipid Metabolism	HDL	rs151097502	11	117388115	G	C	0.197	0.023	3.6e-17	6.3e-05	71.0
Lipid Metabolism	HDL	rs575628211	11	117525443	C	G	0.447	0.056	1.4e-15	9.7e-05	63.8
Lipid Metabolism	HDL	rs644273	11	118511352	T	C	0.008	0.001	1.1e-08	2.6e-05	32.6
Lipid Metabolism	HDL	rs7925100	11	118941596	A	G	-0.016	0.001	2.4e-29	1.0e-04	126.5
Lipid Metabolism	HDL	rs184407727	11	119095360	C	G	0.085	0.016	4.4e-08	2.5e-05	30.0
Lipid Metabolism	HDL	rs2006623	11	119808535	A	G	0.009	0.002	3.5e-10	3.2e-05	39.4
Lipid Metabolism	HDL	rs1622638	11	121800971	A	G	-0.009	0.001	3.1e-11	3.6e-05	44.1
Lipid Metabolism	HDL	rs10892870	11	122517803	G	A	0.024	0.001	1.8e-64	2.3e-04	287.4
Lipid Metabolism	HDL	rs10892891	11	122608411	A	G	-0.010	0.001	4.0e-12	3.9e-05	48.1
Lipid Metabolism	HDL	rs73632737	11	126226775	C	G	-0.037	0.003	6.7e-44	1.6e-04	193.1
Lipid Metabolism	HDL	rs10750325	11	126331836	A	G	-0.012	0.001	1.2e-18	6.3e-05	77.7
Lipid Metabolism	HDL	rs7960436	12	1166702	A	T	-0.009	0.001	8.0e-11	3.5e-05	42.3
Lipid Metabolism	HDL	rs56196860	12	2908330	A	C	0.034	0.004	3.0e-14	4.7e-05	57.7
Lipid Metabolism	HDL	rs9888313	12	6683011	G	A	-0.017	0.002	4.3e-21	7.2e-05	88.8
Lipid Metabolism	HDL	rs11064373	12	6871709	A	G	-0.012	0.002	7.6e-13	4.1e-05	51.4
Lipid Metabolism	HDL	rs74976859	12	13345397	G	T	0.012	0.002	3.9e-09	2.8e-05	34.6
Lipid Metabolism	HDL	rs11045171	12	20470199	G	A	0.027	0.002	6.2e-53	1.9e-04	234.5
Lipid Metabolism	HDL	rs7134150	12	20591332	A	G	-0.025	0.003	1.5e-19	6.6e-05	81.8
Lipid Metabolism	HDL	rs10842703	12	26456188	T	A	-0.012	0.002	1.1e-14	4.8e-05	59.7
Lipid Metabolism	HDL	rs2892701	12	33547395	C	T	-0.008	0.001	3.1e-09	2.8e-05	35.1
Lipid Metabolism	HDL	rs12367809	12	50256063	T	C	-0.011	0.001	2.5e-15	5.0e-05	62.6
Lipid Metabolism	HDL	rs11169571	12	51213765	C	T	0.011	0.001	6.0e-14	4.6e-05	56.4
Lipid Metabolism	HDL	rs11170418	12	53500948	G	A	0.012	0.002	7.4e-09	2.7e-05	33.4
Lipid Metabolism	HDL	rs10783578	12	53796754	A	G	-0.015	0.002	2.1e-22	9.2e-05	94.8
Lipid Metabolism	HDL	rs11170611	12	54005098	G	C	-0.015	0.002	9.1e-16	5.2e-05	64.6
Lipid Metabolism	HDL	rs7297175	12	56473808	C	T	-0.011	0.001	2.6e-15	5.1e-05	62.5
Lipid Metabolism	HDL	rs35493121	12	57390038	C	T	0.015	0.002	2.3e-09	2.9e-05	35.7
Lipid Metabolism	HDL	rs73119306	12	57826982	G	A	0.027	0.002	8.2e-66	2.4e-04	293.6
Lipid Metabolism	HDL	rs10783828	12	57861484	G	A	0.015	0.001	9.8e-24	8.1e-05	100.9
Lipid Metabolism	HDL	rs10877949	12	63466131	T	C	0.009	0.002	1.8e-08	2.6e-05	31.7
Lipid Metabolism	HDL	rs811820	12	67684702	C	T	0.009	0.001	4.1e-09	2.8e-05	34.6
Lipid Metabolism	HDL	rs800660	12	69944921	T	C	-0.010	0.001	1.3e-11	3.7e-05	45.9
Lipid Metabolism	HDL	rs1913196	12	71418347	G	A	0.009	0.001	4.6e-10	3.1e-05	38.8
Lipid Metabolism	HDL	rs2126606	12	89674799	C	T	-0.010	0.001	1.6e-12	4.1e-05	49.9

Phenotype	Trait	SNP	Chr	Position (GRCh37/hg19)	EA	NEA	BETA	SE	P-value	R2	F
Lipid Metabolism	HDL	rs11107117	12	93979419	C	T	-0.010	0.002	3.3e-09	2.8e-05	35.0
Lipid Metabolism	HDL	rs10777606	12	94979930	A	T	-0.008	0.001	1.8e-08	2.6e-05	31.8
Lipid Metabolism	HDL	rs1012306	12	101888063	T	C	0.010	0.001	7.1e-13	4.2e-05	51.5
Lipid Metabolism	HDL	rs4964188	12	107100717	C	T	-0.010	0.001	1.3e-11	3.7e-05	45.9
Lipid Metabolism	HDL	rs755893	12	109153099	G	A	-0.011	0.002	8.9e-13	4.1e-05	51.1
Lipid Metabolism	HDL	rs149793040	12	109661672	G	A	0.148	0.021	8.1e-13	5.3e-05	51.3
Lipid Metabolism	HDL	rs7309513	12	109712222	A	T	0.011	0.002	5.8e-10	3.1e-05	38.4
Lipid Metabolism	HDL	rs2338104	12	109895168	G	C	0.028	0.001	1.6e-93	3.4e-04	420.9
Lipid Metabolism	HDL	rs561393769	12	111807420	C	A	-0.074	0.008	5.8e-19	6.5e-05	79.1
Lipid Metabolism	HDL	rs534664780	12	111808379	C	T	-0.170	0.022	4.7e-15	8.2e-05	61.4
Lipid Metabolism	HDL	rs145588717	12	111834216	T	G	0.058	0.009	6.9e-10	3.5e-05	38.0
Lipid Metabolism	HDL	rs72650673	12	111885310	A	G	-0.171	0.020	4.7e-18	8.1e-05	75.0
Lipid Metabolism	HDL	rs4766578	12	111904371	A	T	0.021	0.001	5.4e-51	1.8e-04	225.6
Lipid Metabolism	HDL	rs551014092	12	111936993	A	G	-0.197	0.023	2.8e-17	1.0e-04	71.5
Lipid Metabolism	HDL	rs543418213	12	111997902	A	G	-0.168	0.022	5.9e-14	7.7e-05	56.4
Lipid Metabolism	HDL	rs540244401	12	112024096	A	G	-0.196	0.030	5.9e-11	1.2e-04	42.9
Lipid Metabolism	HDL	rs552431601	12	112816664	A	G	-0.075	0.008	1.3e-22	7.8e-05	95.8
Lipid Metabolism	HDL	rs56098743	12	113031957	T	C	0.011	0.001	3.9e-14	4.7e-05	57.2
Lipid Metabolism	HDL	rs6489910	12	114085534	G	T	0.011	0.002	1.0e-09	3.0e-05	37.3
Lipid Metabolism	HDL	rs10507274	12	117160976	C	T	0.018	0.003	6.9e-10	3.1e-05	38.0
Lipid Metabolism	HDL	rs1920792	12	121404584	C	T	-0.015	0.001	9.1e-27	9.3e-05	114.7
Lipid Metabolism	HDL	rs61954254	12	122635613	A	G	0.013	0.002	1.1e-12	4.2e-05	50.6
Lipid Metabolism	HDL	rs573513354	12	122651836	G	A	-0.031	0.004	1.1e-12	4.1e-05	50.7
Lipid Metabolism	HDL	rs182920707	12	122938247	T	C	0.107	0.015	1.8e-12	4.3e-05	49.6
Lipid Metabolism	HDL	rs117243285	12	123036437	A	G	-0.055	0.010	1.6e-08	2.7e-05	31.9
Lipid Metabolism	HDL	rs2454702	12	123209159	T	C	0.030	0.002	1.4e-71	2.6e-04	320.1
Lipid Metabolism	HDL	rs116988067	12	123263421	A	G	-0.054	0.007	1.3e-14	4.9e-05	59.3
Lipid Metabolism	HDL	rs546143564	12	123699738	A	G	0.290	0.043	1.2e-11	6.1e-05	46.0
Lipid Metabolism	HDL	rs28861152	12	123886019	C	A	0.038	0.003	6.0e-39	1.4e-04	170.4
Lipid Metabolism	HDL	rs118170661	12	124056029	A	G	0.043	0.007	1.3e-08	3.3e-05	32.3
Lipid Metabolism	HDL	rs4930731	12	124317416	G	A	-0.017	0.001	4.9e-31	1.1e-04	134.2
Lipid Metabolism	HDL	rs7133378	12	124409502	A	G	0.033	0.001	1.6e-111	4.1e-04	503.6
Lipid Metabolism	HDL	rs76843642	12	124819573	G	C	-0.047	0.008	2.4e-08	2.6e-05	31.1
Lipid Metabolism	HDL	rs61705577	12	125095388	G	C	-0.017	0.002	2.8e-16	5.4e-05	67.0
Lipid Metabolism	HDL	rs191624611	12	125138722	G	A	0.045	0.006	1.9e-12	4.0e-05	49.6
Lipid Metabolism	HDL	rs2694826	12	125282882	G	A	-0.031	0.001	7.6e-114	4.2e-04	514.2
Lipid Metabolism	HDL	rs187831231	12	125298855	C	T	0.455	0.053	1.7e-17	9.4e-05	72.5
Lipid Metabolism	HDL	rs117357660	12	125306572	T	C	0.089	0.008	8.5e-30	1.1e-04	128.6
Lipid Metabolism	HDL	rs139530550	12	125322808	A	G	-0.065	0.011	1.4e-09	3.0e-05	36.7
Lipid Metabolism	HDL	rs61941677	12	125325010	G	C	-0.062	0.002	5.6e-188	6.9e-04	855.1
Lipid Metabolism	HDL	rs148078602	12	132466836	T	C	-0.036	0.006	1.2e-08	2.6e-05	32.5
Lipid Metabolism	HDL	rs12584195	13	23893015	G	A	0.008	0.001	5.2e-09	2.7e-05	34.1
Lipid Metabolism	HDL	rs9507893	13	28002724	T	C	0.009	0.002	1.6e-09	2.9e-05	36.4
Lipid Metabolism	HDL	rs4397962	13	28687084	C	T	-0.012	0.002	7.3e-13	4.1e-05	51.5
Lipid Metabolism	HDL	rs35052580	13	30976240	T	G	-0.018	0.003	2.3e-08	2.5e-05	31.2
Lipid Metabolism	HDL	rs17532371	13	41635301	G	C	-0.025	0.003	6.0e-21	7.1e-05	88.2
Lipid Metabolism	HDL	rs144351842	13	45568590	T	G	0.027	0.004	1.4e-10	3.3e-05	41.1
Lipid Metabolism	HDL	rs1747223	13	47224681	G	A	0.010	0.002	3.0e-10	3.2e-05	39.7
Lipid Metabolism	HDL	rs148899725	13	50196041	C	G	0.032	0.005	1.5e-09	3.0e-05	36.6
Lipid Metabolism	HDL	rs549058	13	51201045	T	G	0.018	0.002	2.5e-18	6.1e-05	76.3
Lipid Metabolism	HDL	rs1537465	13	52093119	C	T	0.008	0.001	7.2e-09	2.7e-05	33.5
Lipid Metabolism	HDL	rs8002963	13	55924014	G	A	-0.010	0.002	3.4e-10	3.2e-05	39.4
Lipid Metabolism	HDL	rs12428841	13	58391491	C	T	-0.011	0.002	5.4e-13	4.2e-05	52.0
Lipid Metabolism	HDL	rs73194763	13	67173665	C	T	-0.010	0.002	2.8e-08	2.5e-05	30.9
Lipid Metabolism	HDL	rs12585801	13	81393264	A	G	0.012	0.002	1.8e-10	3.3e-05	40.7
Lipid Metabolism	HDL	rs6492538	13	91993746	C	A	0.010	0.002	1.2e-08	2.6e-05	32.5

Phenotype	Trait	SNP	Chr	Position (GRCh37/hg19)	EA	NEA	BETA	SE	P-value	R2	F
Lipid Metabolism	HDL	rs4644730	13	94099216	G	T	-0.009	0.002	2.4e-08	2.5e-05	31.1
Lipid Metabolism	HDL	rs9554477	13	99160379	T	C	-0.009	0.001	5.8e-10	3.1e-05	38.4
Lipid Metabolism	HDL	rs374039502	13	108960385	A	T	-0.044	0.006	6.0e-15	5.6e-05	60.9
Lipid Metabolism	HDL	rs75533516	13	111538525	G	T	-0.008	0.001	3.2e-09	2.8e-05	35.0
Lipid Metabolism	HDL	rs9604045	13	113927208	T	G	0.017	0.002	4.2e-20	7.3e-05	84.3
Lipid Metabolism	HDL	rs7401804	14	24755872	T	C	0.009	0.001	3.6e-10	3.2e-05	39.3
Lipid Metabolism	HDL	rs7159513	14	29856951	A	G	-0.011	0.002	6.4e-09	2.7e-05	33.7
Lipid Metabolism	HDL	rs1279143	14	33175100	G	A	-0.008	0.001	4.3e-09	2.8e-05	34.5
Lipid Metabolism	HDL	rs140664623	14	52321987	T	G	0.028	0.005	2.7e-09	2.9e-05	35.4
Lipid Metabolism	HDL	rs146182298	14	52531408	T	C	-0.022	0.004	6.1e-10	3.1e-05	38.3
Lipid Metabolism	HDL	rs2149839	14	65983448	A	G	0.011	0.001	5.7e-13	4.2e-05	51.9
Lipid Metabolism	HDL	rs72729582	14	69149372	G	A	0.023	0.003	1.1e-17	5.9e-05	73.4
Lipid Metabolism	HDL	rs11621104	14	69239751	A	G	-0.010	0.001	4.4e-12	3.9e-05	47.9
Lipid Metabolism	HDL	rs8015046	14	70775792	C	T	-0.010	0.001	1.8e-13	4.4e-05	54.2
Lipid Metabolism	HDL	rs10148631	14	74231727	A	T	-0.010	0.002	1.1e-10	3.4e-05	41.6
Lipid Metabolism	HDL	rs13379042	14	74250100	C	T	0.019	0.002	5.0e-33	1.2e-04	143.3
Lipid Metabolism	HDL	rs2287403	14	75259140	T	C	-0.010	0.001	5.7e-13	4.2e-05	51.9
Lipid Metabolism	HDL	rs10146690	14	79890456	A	G	-0.012	0.002	6.6e-12	3.8e-05	47.1
Lipid Metabolism	HDL	rs2371562	14	81619474	A	C	0.011	0.002	5.4e-13	4.2e-05	52.1
Lipid Metabolism	HDL	rs4904549	14	89799382	T	C	-0.015	0.002	6.4e-17	5.6e-05	69.8
Lipid Metabolism	HDL	rs8014854	14	92550658	A	G	0.008	0.001	1.0e-08	2.6e-05	32.8
Lipid Metabolism	HDL	rs10149866	14	100371963	G	A	-0.014	0.002	3.3e-14	4.6e-05	57.6
Lipid Metabolism	HDL	rs34820917	14	100686816	A	G	0.018	0.003	5.5e-10	3.1e-05	38.5
Lipid Metabolism	HDL	rs8022180	14	103263020	A	G	0.013	0.001	2.7e-18	6.1e-05	76.1
Lipid Metabolism	HDL	rs34251364	14	105180785	T	C	0.016	0.003	2.0e-09	2.9e-05	36.0
Lipid Metabolism	HDL	rs45490496	14	105272678	T	A	-0.025	0.001	1.3e-66	2.5e-04	297.2
Lipid Metabolism	HDL	rs7497973	15	23948571	A	G	0.010	0.001	2.0e-12	4.0e-05	49.5
Lipid Metabolism	HDL	rs28624578	15	31637666	C	T	-0.012	0.002	7.3e-10	3.1e-05	37.9
Lipid Metabolism	HDL	rs17574546	15	38902476	C	A	-0.010	0.002	2.6e-09	2.9e-05	35.5
Lipid Metabolism	HDL	rs275182	15	39446211	G	A	0.015	0.002	4.0e-15	5.0e-05	61.7
Lipid Metabolism	HDL	rs4923869	15	40737490	T	C	0.010	0.001	5.3e-13	4.3e-05	52.1
Lipid Metabolism	HDL	rs62003872	15	41886714	T	C	0.013	0.002	7.6e-17	5.6e-05	69.5
Lipid Metabolism	HDL	rs28364531	15	42701374	T	C	-0.068	0.005	1.3e-40	1.4e-04	178.0
Lipid Metabolism	HDL	rs55707100	15	43820717	T	C	-0.102	0.004	3.3e-123	4.5e-04	557.3
Lipid Metabolism	HDL	rs151291132	15	44842210	G	A	-0.087	0.005	2.1e-72	2.6e-04	323.9
Lipid Metabolism	HDL	rs12443496	15	50766017	T	A	-0.010	0.002	4.1e-09	3.4e-05	34.6
Lipid Metabolism	HDL	rs2414178	15	53001538	C	T	0.011	0.002	3.1e-11	3.6e-05	44.1
Lipid Metabolism	HDL	rs2951900	15	57335354	T	C	0.011	0.002	5.9e-12	3.8e-05	47.4
Lipid Metabolism	HDL	rs552730008	15	58199779	A	G	0.098	0.014	5.3e-12	6.9e-05	47.6
Lipid Metabolism	HDL	rs140155486	15	58330002	G	A	0.089	0.014	5.3e-11	5.0e-05	43.1
Lipid Metabolism	HDL	rs148811668	15	58446025	G	T	0.091	0.014	1.3e-10	3.6e-05	41.3
Lipid Metabolism	HDL	rs530353706	15	58469457	T	C	-0.084	0.015	1.6e-08	2.7e-05	31.9
Lipid Metabolism	HDL	rs571978992	15	58490170	T	C	0.131	0.019	3.0e-12	5.3e-05	48.7
Lipid Metabolism	HDL	rs117739518	15	58515678	T	C	0.085	0.015	2.6e-08	2.6e-05	31.0
Lipid Metabolism	HDL	rs182996383	15	58539524	T	C	0.124	0.021	2.5e-09	4.5e-05	35.6
Lipid Metabolism	HDL	rs144506721	15	58543163	C	T	0.126	0.019	1.1e-10	5.4e-05	41.7
Lipid Metabolism	HDL	rs113629348	15	58545668	T	C	0.052	0.005	9.2e-29	1.0e-04	123.8
Lipid Metabolism	HDL	rs139320792	15	58549825	A	G	0.092	0.009	3.1e-27	9.5e-05	116.8
Lipid Metabolism	HDL	rs76970180	15	58554914	T	G	0.053	0.005	5.7e-24	8.3e-05	102.0
Lipid Metabolism	HDL	rs11071371	15	58576226	T	C	-0.074	0.002	1.0e-200	1.0e-03	1277.1
Lipid Metabolism	HDL	rs76450027	15	58595273	T	C	0.085	0.008	3.3e-28	9.9e-05	121.3
Lipid Metabolism	HDL	rs182906578	15	58622281	C	T	-0.066	0.010	2.4e-11	3.7e-05	44.6
Lipid Metabolism	HDL	rs554814185	15	58626022	G	C	0.092	0.013	2.4e-12	4.1e-05	49.1
Lipid Metabolism	HDL	rs535978974	15	58641302	G	T	0.096	0.009	4.5e-26	1.2e-04	111.5
Lipid Metabolism	HDL	rs575607189	15	58641352	C	T	-0.078	0.014	2.0e-08	2.7e-05	31.5
Lipid Metabolism	HDL	rs575398050	15	58651306	A	C	0.140	0.018	1.8e-14	6.6e-05	58.8

Phenotype	Trait	SNP	Chr	Position (GRCh37/hg19)	EA	NEA	BETA	SE	P-value	R2	F
Lipid Metabolism	HDL	rs1601933	15	58671559	T	C	-0.073	0.001	1.0e-200	2.1e-03	2554.5
Lipid Metabolism	HDL	rs573241250	15	58679688	C	G	0.111	0.008	2.6e-44	1.6e-04	195.0
Lipid Metabolism	HDL	rs189926464	15	58680560	C	A	0.099	0.012	8.4e-17	5.8e-05	69.3
Lipid Metabolism	HDL	rs188104538	15	58690406	C	T	0.137	0.013	3.9e-25	9.2e-05	107.3
Lipid Metabolism	HDL	rs183914442	15	58691130	A	G	0.137	0.014	4.4e-24	1.1e-04	102.4
Lipid Metabolism	HDL	rs529719658	15	58713050	A	G	-0.085	0.013	2.0e-10	3.4e-05	40.5
Lipid Metabolism	HDL	rs575288907	15	58714443	G	T	0.124	0.020	3.9e-10	5.1e-05	39.2
Lipid Metabolism	HDL	rs72740877	15	58728067	A	G	0.121	0.004	1.0e-200	7.7e-04	951.8
Lipid Metabolism	HDL	rs117489790	15	58728372	A	G	-0.107	0.019	2.1e-08	3.3e-05	31.4
Lipid Metabolism	HDL	rs79447951	15	58735638	G	T	0.113	0.006	1.1e-84	3.1e-04	380.2
Lipid Metabolism	HDL	rs41292486	15	58751018	A	G	0.037	0.002	4.3e-51	1.8e-04	226.0
Lipid Metabolism	HDL	rs553316571	15	58766942	T	G	0.127	0.023	3.7e-08	3.8e-05	30.3
Lipid Metabolism	HDL	rs115578058	15	58783364	T	C	0.336	0.049	9.6e-12	5.8e-05	46.4
Lipid Metabolism	HDL	rs143819838	15	58827270	A	G	0.341	0.048	1.1e-12	5.8e-05	50.6
Lipid Metabolism	HDL	rs139878091	15	58854966	G	A	0.057	0.010	6.4e-09	3.1e-05	33.7
Lipid Metabolism	HDL	rs113298164	15	58855748	T	C	0.283	0.012	2.5e-123	5.1e-04	557.8
Lipid Metabolism	HDL	rs534741668	15	58944990	A	G	0.333	0.057	6.2e-09	6.0e-05	33.8
Lipid Metabolism	HDL	rs139233075	15	58956101	T	C	0.094	0.015	7.5e-11	6.2e-05	42.4
Lipid Metabolism	HDL	rs11637189	15	59022251	A	G	0.239	0.028	2.0e-17	1.0e-04	72.1
Lipid Metabolism	HDL	rs12915952	15	59041347	C	A	0.041	0.006	8.1e-13	4.1e-05	51.3
Lipid Metabolism	HDL	rs143599834	15	59058032	T	A	-0.042	0.007	6.9e-10	3.1e-05	38.1
Lipid Metabolism	HDL	rs575158206	15	59238817	G	A	0.427	0.074	6.5e-09	9.1e-05	33.7
Lipid Metabolism	HDL	rs557161294	15	59427594	A	G	0.416	0.059	1.8e-12	1.1e-04	49.7
Lipid Metabolism	HDL	rs554577840	15	59916939	T	C	0.090	0.016	1.3e-08	3.3e-05	32.3
Lipid Metabolism	HDL	rs7178050	15	59923814	G	A	0.009	0.002	2.6e-08	3.0e-05	31.0
Lipid Metabolism	HDL	rs10851698	15	61947694	T	C	-0.013	0.002	7.2e-16	5.2e-05	65.1
Lipid Metabolism	HDL	rs2134263	15	63365394	T	C	0.016	0.001	2.0e-31	1.1e-04	136.0
Lipid Metabolism	HDL	rs75663391	15	63910433	T	G	0.013	0.002	6.0e-16	6.3e-05	65.4
Lipid Metabolism	HDL	rs7178249	15	66560723	T	C	-0.008	0.001	3.9e-08	2.4e-05	30.2
Lipid Metabolism	HDL	rs2469128	15	66877172	A	G	0.010	0.001	6.7e-13	4.2e-05	51.6
Lipid Metabolism	HDL	rs9302245	15	68021137	T	C	-0.009	0.001	4.2e-11	3.5e-05	43.5
Lipid Metabolism	HDL	rs1900901	15	73257725	G	A	-0.009	0.001	4.9e-09	2.8e-05	34.2
Lipid Metabolism	HDL	rs8023580	15	96708291	C	T	0.011	0.002	1.0e-12	4.1e-05	50.8
Lipid Metabolism	HDL	rs3803476	15	99256570	G	A	0.008	0.001	1.4e-08	2.6e-05	32.3
Lipid Metabolism	HDL	rs8025505	15	102067841	T	C	0.011	0.002	3.0e-11	3.6e-05	44.2
Lipid Metabolism	HDL	rs11248955	16	984113	A	C	0.013	0.002	6.3e-18	6.1e-05	74.4
Lipid Metabolism	HDL	rs8045264	16	1251203	G	A	-0.009	0.002	1.2e-08	2.7e-05	32.4
Lipid Metabolism	HDL	rs75510884	16	2161796	A	G	0.015	0.002	1.3e-09	3.0e-05	36.8
Lipid Metabolism	HDL	rs72766660	16	2254626	A	G	-0.023	0.003	2.5e-12	4.0e-05	49.0
Lipid Metabolism	HDL	rs117845654	16	4737950	A	G	-0.028	0.004	3.6e-10	3.2e-05	39.3
Lipid Metabolism	HDL	rs149961585	16	11430375	T	C	0.016	0.002	6.8e-18	6.0e-05	74.3
Lipid Metabolism	HDL	rs393329	16	12070422	C	T	0.008	0.001	5.5e-09	2.8e-05	34.0
Lipid Metabolism	HDL	rs12928099	16	15150505	A	C	0.018	0.002	7.6e-33	1.2e-04	142.5
Lipid Metabolism	HDL	rs58653754	16	19977601	A	C	0.014	0.002	8.8e-13	4.1e-05	51.1
Lipid Metabolism	HDL	rs801754	16	23597878	A	T	0.011	0.002	5.5e-10	3.1e-05	38.5
Lipid Metabolism	HDL	rs34172651	16	24766841	C	T	0.009	0.002	8.4e-10	3.0e-05	37.7
Lipid Metabolism	HDL	rs62037364	16	28868695	A	G	-0.010	0.001	4.4e-12	3.9e-05	47.9
Lipid Metabolism	HDL	rs3814883	16	29994922	T	C	-0.013	0.001	2.4e-19	6.6e-05	80.9
Lipid Metabolism	HDL	rs75500538	16	30782449	A	G	-0.014	0.002	1.5e-10	3.3e-05	41.1
Lipid Metabolism	HDL	rs9673398	16	31347519	G	A	0.010	0.001	2.8e-12	3.9e-05	48.9
Lipid Metabolism	HDL	rs1558902	16	53803574	A	T	-0.019	0.001	2.5e-43	1.5e-04	190.5
Lipid Metabolism	HDL	rs191043807	16	55349464	C	T	0.279	0.048	8.0e-09	5.3e-05	33.3
Lipid Metabolism	HDL	rs373447049	16	55469520	A	G	0.176	0.027	4.4e-11	8.7e-05	43.4
Lipid Metabolism	HDL	rs191123527	16	55775316	A	G	0.121	0.020	1.2e-09	3.7e-05	37.0
Lipid Metabolism	HDL	rs532566544	16	55793904	C	A	0.311	0.038	1.7e-16	9.9e-05	68.0
Lipid Metabolism	HDL	rs187555547	16	55850130	T	C	-0.110	0.018	3.5e-10	4.3e-05	39.4

Phenotype	Trait	SNP	Chr	Position (GRCh37/hg19)	EA	NEA	BETA	SE	P-value	R2	F
Lipid Metabolism	HDL	rs2397965	16	55907811	T	C	-0.013	0.002	2.5e-12	3.9e-05	49.1
Lipid Metabolism	HDL	rs190158139	16	55919424	A	G	0.175	0.031	2.4e-08	4.5e-05	31.1
Lipid Metabolism	HDL	rs148557106	16	55933331	G	C	0.063	0.008	3.0e-15	5.1e-05	62.2
Lipid Metabolism	HDL	rs138443105	16	55946148	T	G	0.076	0.010	8.6e-15	4.9e-05	60.2
Lipid Metabolism	HDL	rs150122297	16	56076200	C	T	0.116	0.012	1.1e-21	9.4e-05	91.5
Lipid Metabolism	HDL	rs571589988	16	56100200	C	T	0.148	0.021	5.8e-12	5.3e-05	47.4
Lipid Metabolism	HDL	rs529223688	16	56200571	A	G	0.144	0.022	1.4e-10	5.3e-05	41.2
Lipid Metabolism	HDL	rs547236680	16	56336270	T	C	-0.164	0.019	3.8e-18	2.0e-04	75.4
Lipid Metabolism	HDL	rs570647289	16	56360351	A	G	0.169	0.030	2.9e-08	8.3e-05	30.8
Lipid Metabolism	HDL	rs9935936	16	56386320	T	G	0.028	0.002	2.2e-52	1.9e-04	232.0
Lipid Metabolism	HDL	rs146232863	16	56471302	T	C	0.085	0.009	6.0e-22	7.6e-05	92.7
Lipid Metabolism	HDL	rs566042676	16	56482025	C	T	-0.113	0.016	6.2e-13	6.2e-05	51.8
Lipid Metabolism	HDL	rs537268934	16	56499391	C	T	0.115	0.016	8.7e-13	5.3e-05	51.1
Lipid Metabolism	HDL	rs9934794	16	56624534	A	T	0.039	0.002	9.4e-64	2.3e-04	284.1
Lipid Metabolism	HDL	rs139872415	16	56648737	T	C	0.187	0.032	3.1e-09	9.3e-05	35.1
Lipid Metabolism	HDL	rs191235553	16	56652702	C	T	-0.139	0.025	3.2e-08	4.3e-05	30.6
Lipid Metabolism	HDL	rs550642522	16	56660107	G	A	-0.227	0.038	1.6e-09	5.2e-05	36.4
Lipid Metabolism	HDL	rs558589053	16	56670548	T	C	0.124	0.021	3.8e-09	3.4e-05	34.7
Lipid Metabolism	HDL	rs533704676	16	56685265	G	A	-0.103	0.014	1.6e-13	5.6e-05	54.4
Lipid Metabolism	HDL	rs186945593	16	56691411	C	G	-0.105	0.007	9.4e-47	1.7e-04	206.2
Lipid Metabolism	HDL	rs561408398	16	56707073	A	C	-0.319	0.043	6.1e-14	9.1e-05	56.3
Lipid Metabolism	HDL	rs186475070	16	56713956	A	G	-0.108	0.018	8.9e-10	3.1e-05	37.6
Lipid Metabolism	HDL	rs551207033	16	56718328	C	T	-0.253	0.043	4.8e-09	5.2e-05	34.3
Lipid Metabolism	HDL	rs185230328	16	56731575	T	C	-0.113	0.015	7.1e-15	5.0e-05	60.6
Lipid Metabolism	HDL	rs138320848	16	56743879	T	C	0.127	0.020	4.5e-10	4.1e-05	38.9
Lipid Metabolism	HDL	rs147804217	16	56759255	T	C	0.661	0.082	1.0e-15	9.3e-05	64.4
Lipid Metabolism	HDL	rs141196069	16	56759652	A	G	-0.112	0.017	1.4e-11	4.0e-05	45.6
Lipid Metabolism	HDL	rs545940065	16	56764966	T	C	0.174	0.019	1.5e-19	9.3e-05	81.8
Lipid Metabolism	HDL	rs34056275	16	56772710	A	G	0.413	0.024	6.4e-69	4.1e-04	307.9
Lipid Metabolism	HDL	rs55951233	16	56783670	G	A	-0.100	0.011	2.4e-19	6.6e-05	80.8
Lipid Metabolism	HDL	rs569764945	16	56839033	A	G	0.112	0.016	2.3e-12	6.8e-05	49.2
Lipid Metabolism	HDL	rs560174974	16	56849828	A	G	0.379	0.054	2.3e-12	4.0e-04	49.2
Lipid Metabolism	HDL	rs574427504	16	56873956	A	G	0.180	0.024	4.5e-14	1.5e-04	57.0
Lipid Metabolism	HDL	rs189385028	16	56881750	A	T	-0.175	0.029	1.4e-09	3.6e-05	36.7
Lipid Metabolism	HDL	rs117254618	16	56892818	G	A	0.084	0.006	1.3e-51	1.9e-04	228.5
Lipid Metabolism	HDL	rs562392336	16	56895768	A	G	0.149	0.019	6.3e-15	1.6e-04	60.8
Lipid Metabolism	HDL	rs200271933	16	56897620	A	G	-0.265	0.028	7.4e-21	8.8e-05	87.8
Lipid Metabolism	HDL	rs534822153	16	56899880	C	T	-0.066	0.008	4.4e-15	5.1e-05	61.5
Lipid Metabolism	HDL	rs141938931	16	56901445	G	A	-0.316	0.013	1.6e-136	5.1e-04	618.4
Lipid Metabolism	HDL	rs547450395	16	56910338	A	G	0.370	0.067	2.8e-08	8.3e-05	30.9
Lipid Metabolism	HDL	rs116383747	16	56910637	A	G	0.166	0.018	2.2e-21	8.2e-05	90.2
Lipid Metabolism	HDL	rs569288154	16	56912293	A	G	-0.178	0.022	3.4e-16	1.8e-04	66.6
Lipid Metabolism	HDL	rs141724276	16	56915407	T	C	0.060	0.005	5.3e-34	1.2e-04	147.8
Lipid Metabolism	HDL	rs12929471	16	56920483	A	G	0.303	0.016	2.5e-76	3.6e-04	341.9
Lipid Metabolism	HDL	rs190351409	16	56922750	A	G	-0.296	0.019	9.6e-56	3.0e-04	247.4
Lipid Metabolism	HDL	rs188174297	16	56923578	C	G	-0.173	0.021	8.9e-17	6.0e-05	69.2
Lipid Metabolism	HDL	rs141890420	16	56931816	A	G	-0.614	0.097	2.1e-10	5.8e-05	40.4
Lipid Metabolism	HDL	rs1138429	16	56942921	T	A	-0.091	0.002	1.0e-200	1.3e-03	1578.0
Lipid Metabolism	HDL	rs188168278	16	56943760	A	G	0.233	0.028	8.0e-17	1.0e-04	69.4
Lipid Metabolism	HDL	rs184498979	16	56952262	A	G	-0.182	0.013	4.6e-45	1.6e-04	198.4
Lipid Metabolism	HDL	rs576324892	16	56956123	G	A	-0.164	0.015	5.7e-28	1.2e-04	120.2
Lipid Metabolism	HDL	rs563586405	16	56962610	A	G	-0.126	0.013	6.2e-21	7.5e-05	88.1
Lipid Metabolism	HDL	rs557774831	16	56971678	T	C	0.231	0.040	7.5e-09	8.9e-05	33.4
Lipid Metabolism	HDL	rs550183772	16	56973744	C	T	-0.296	0.015	2.0e-89	3.4e-04	402.0
Lipid Metabolism	HDL	rs577442496	16	56974340	A	T	0.422	0.073	8.8e-09	8.9e-05	33.1
Lipid Metabolism	HDL	rs573155644	16	56976064	A	C	0.192	0.033	3.3e-09	9.3e-05	35.0

Phenotype	Trait	SNP	Chr	Position (GRCh37/hg19)	EA	NEA	BETA	SE	P-value	R2	F
Lipid Metabolism	HDL	rs184418117	16	56976191	G	A	0.225	0.035	1.8e-10	1.1e-04	40.6
Lipid Metabolism	HDL	rs377449652	16	56976743	T	C	-0.199	0.024	1.2e-16	7.8e-05	68.5
Lipid Metabolism	HDL	rs554121714	16	56977748	C	T	0.207	0.024	8.6e-18	9.6e-05	73.8
Lipid Metabolism	HDL	rs150266133	16	56980193	T	C	-0.115	0.011	1.1e-23	8.3e-05	100.6
Lipid Metabolism	HDL	rs72786786	16	56985514	A	G	0.212	0.002	1.0e-200	1.5e-02	18865.9
Lipid Metabolism	HDL	rs139884539	16	56989246	T	C	0.215	0.038	1.1e-08	4.3e-05	32.7
Lipid Metabolism	HDL	rs140806876	16	56992473	T	C	-0.409	0.038	3.2e-27	1.2e-04	116.8
Lipid Metabolism	HDL	rs541129597	16	56993748	A	G	-0.259	0.044	5.2e-09	4.8e-05	34.1
Lipid Metabolism	HDL	rs142464301	16	56996985	T	C	-0.555	0.051	5.5e-28	1.7e-04	120.3
Lipid Metabolism	HDL	rs573857010	16	57000986	A	G	0.207	0.033	4.3e-10	4.0e-05	39.0
Lipid Metabolism	HDL	rs12708972	16	57004892	T	C	0.143	0.009	2.1e-52	2.0e-04	232.1
Lipid Metabolism	HDL	rs573864685	16	57007053	G	A	-0.542	0.099	4.9e-08	4.5e-05	29.8
Lipid Metabolism	HDL	rs545858676	16	57009137	C	A	-0.093	0.005	4.0e-65	3.2e-04	290.4
Lipid Metabolism	HDL	rs72771489	16	57010117	A	G	-0.139	0.010	2.5e-44	1.6e-04	195.1
Lipid Metabolism	HDL	rs551343692	16	57011265	T	C	0.277	0.040	6.2e-12	1.3e-04	47.3
Lipid Metabolism	HDL	rs567823047	16	57011350	G	A	0.569	0.096	2.6e-09	5.2e-05	35.5
Lipid Metabolism	HDL	rs534053619	16	57011643	G	T	0.182	0.026	4.0e-12	5.6e-05	48.1
Lipid Metabolism	HDL	rs138298866	16	57012038	T	C	0.225	0.024	3.3e-20	1.3e-04	84.8
Lipid Metabolism	HDL	rs182237338	16	57012174	T	C	-0.204	0.023	2.0e-19	7.1e-05	81.3
Lipid Metabolism	HDL	rs184617400	16	57019498	T	G	-0.258	0.024	4.4e-26	1.1e-04	111.6
Lipid Metabolism	HDL	rs562763805	16	57041609	T	C	0.119	0.022	4.0e-08	3.8e-05	30.2
Lipid Metabolism	HDL	rs182457758	16	57043340	T	C	0.105	0.008	1.4e-44	1.6e-04	196.2
Lipid Metabolism	HDL	rs191020702	16	57047121	T	C	-0.128	0.015	2.3e-17	8.5e-05	71.8
Lipid Metabolism	HDL	rs538640172	16	57050024	A	G	0.171	0.024	1.3e-12	1.3e-04	50.3
Lipid Metabolism	HDL	rs140440847	16	57054648	T	C	-0.147	0.024	1.3e-09	5.8e-05	36.9
Lipid Metabolism	HDL	rs185197208	16	57055635	A	G	-0.261	0.008	1.0e-200	9.1e-04	1117.3
Lipid Metabolism	HDL	rs291040	16	57061189	C	T	-0.033	0.001	9.9e-113	4.1e-04	509.1
Lipid Metabolism	HDL	rs118063294	16	57069661	A	G	0.296	0.044	1.4e-11	7.4e-05	45.7
Lipid Metabolism	HDL	rs568271305	16	57071647	G	C	-0.139	0.022	2.9e-10	3.9e-05	39.8
Lipid Metabolism	HDL	rs116932832	16	57074739	A	C	0.094	0.008	4.1e-33	1.2e-04	143.7
Lipid Metabolism	HDL	rs200883407	16	57080525	T	C	0.196	0.032	5.7e-10	8.6e-05	38.4
Lipid Metabolism	HDL	rs146644349	16	57093472	A	G	-0.077	0.012	1.8e-11	3.7e-05	45.2
Lipid Metabolism	HDL	rs190152429	16	57098236	C	T	0.096	0.008	3.1e-30	1.1e-04	130.5
Lipid Metabolism	HDL	rs181828544	16	57100168	A	G	0.075	0.012	8.9e-10	3.1e-05	37.5
Lipid Metabolism	HDL	rs564055199	16	57106823	T	C	0.130	0.022	2.1e-09	4.7e-05	35.9
Lipid Metabolism	HDL	rs149946718	16	57160796	A	G	-0.092	0.016	2.1e-08	3.4e-05	31.4
Lipid Metabolism	HDL	rs72777124	16	57170403	A	C	0.074	0.009	1.0e-15	5.3e-05	64.4
Lipid Metabolism	HDL	rs140778950	16	57207815	A	T	0.071	0.012	6.7e-10	3.2e-05	38.1
Lipid Metabolism	HDL	rs117574240	16	57224797	G	C	0.073	0.008	8.3e-22	7.5e-05	92.1
Lipid Metabolism	HDL	rs141702855	16	57274272	C	A	-0.124	0.012	1.3e-25	1.3e-04	109.4
Lipid Metabolism	HDL	rs555380585	16	57280321	A	C	0.102	0.017	7.1e-10	4.5e-05	38.0
Lipid Metabolism	HDL	rs145338123	16	57285539	T	C	0.062	0.007	1.0e-20	7.1e-05	87.1
Lipid Metabolism	HDL	rs534302442	16	57286573	G	T	0.193	0.034	1.0e-08	6.0e-05	32.8
Lipid Metabolism	HDL	rs535345929	16	57320410	G	A	-0.201	0.026	6.3e-15	6.0e-05	60.8
Lipid Metabolism	HDL	rs571201725	16	57334448	T	C	0.051	0.009	2.0e-08	2.6e-05	31.5
Lipid Metabolism	HDL	rs185303872	16	57358964	G	A	-0.080	0.013	8.7e-10	3.1e-05	37.6
Lipid Metabolism	HDL	rs190909246	16	57435730	T	C	0.262	0.045	5.9e-09	4.9e-05	33.9
Lipid Metabolism	HDL	rs545967095	16	57470145	C	T	0.104	0.016	2.3e-10	5.1e-05	40.2
Lipid Metabolism	HDL	rs568583826	16	57511575	T	C	0.252	0.038	2.0e-11	1.3e-04	45.0
Lipid Metabolism	HDL	rs141363574	16	57560075	A	G	-0.080	0.014	1.3e-08	2.7e-05	32.3
Lipid Metabolism	HDL	rs186329738	16	58329622	G	C	-0.172	0.031	1.8e-08	3.1e-05	31.7
Lipid Metabolism	HDL	rs62042608	16	58436831	G	T	-0.147	0.026	1.8e-08	3.9e-05	31.7
Lipid Metabolism	HDL	rs111804055	16	66481845	G	A	0.187	0.033	2.1e-08	3.3e-05	31.4
Lipid Metabolism	HDL	rs150034947	16	66540996	C	T	-0.307	0.030	5.2e-25	1.3e-04	106.7
Lipid Metabolism	HDL	rs140824838	16	66618446	A	G	0.081	0.010	3.3e-16	5.5e-05	66.6
Lipid Metabolism	HDL	rs187605327	16	66679390	A	G	0.070	0.012	9.1e-09	2.8e-05	33.0

Phenotype	Trait	SNP	Chr	Position (GRCh37/hg19)	EA	NEA	BETA	SE	P-value	R2	F
Lipid Metabolism	HDL	rs75895207	16	66705632	T	G	0.060	0.011	3.4e-08	2.5e-05	30.5
Lipid Metabolism	HDL	rs189151056	16	66753820	T	C	0.126	0.023	3.7e-08	2.7e-05	30.3
Lipid Metabolism	HDL	rs574765101	16	66817138	T	C	-0.574	0.082	2.2e-12	1.2e-04	49.3
Lipid Metabolism	HDL	rs111741113	16	66878058	T	C	0.051	0.008	7.7e-10	3.1e-05	37.8
Lipid Metabolism	HDL	rs190019902	16	66900280	T	G	0.043	0.008	4.6e-08	2.4e-05	29.9
Lipid Metabolism	HDL	rs186208576	16	66910303	A	G	0.117	0.012	2.6e-24	9.2e-05	103.5
Lipid Metabolism	HDL	rs77234291	16	66911898	G	A	0.042	0.005	1.1e-14	4.8e-05	59.7
Lipid Metabolism	HDL	rs11645675	16	67010749	T	C	-0.043	0.003	5.9e-45	1.6e-04	197.9
Lipid Metabolism	HDL	rs112819029	16	67028572	G	A	0.101	0.012	4.9e-17	8.3e-05	70.4
Lipid Metabolism	HDL	rs574144420	16	67028940	T	C	0.070	0.007	1.3e-21	8.3e-05	91.2
Lipid Metabolism	HDL	rs535142017	16	67737082	A	G	0.092	0.010	1.8e-18	7.2e-05	76.9
Lipid Metabolism	HDL	rs553482792	16	67770610	T	C	0.117	0.019	1.1e-09	4.9e-05	37.1
Lipid Metabolism	HDL	rs7206896	16	67806525	C	T	-0.102	0.010	1.7e-23	8.3e-05	99.8
Lipid Metabolism	HDL	rs8054091	16	67931826	C	T	0.070	0.002	1.0e-200	8.9e-04	1113.0
Lipid Metabolism	HDL	rs560841049	16	67955953	G	C	-0.361	0.029	1.2e-35	2.6e-04	155.3
Lipid Metabolism	HDL	rs144922262	16	67977348	T	C	-0.987	0.048	2.4e-94	8.0e-04	424.7
Lipid Metabolism	HDL	rs548291389	16	68004792	G	T	-1.162	0.057	1.8e-93	8.4e-04	420.7
Lipid Metabolism	HDL	rs191285534	16	68112322	C	T	-0.331	0.054	7.8e-10	7.7e-05	37.8
Lipid Metabolism	HDL	rs187702522	16	68143204	T	C	0.202	0.030	1.0e-11	5.1e-05	46.3
Lipid Metabolism	HDL	rs186429262	16	68239482	G	A	-0.825	0.055	1.3e-51	3.8e-04	228.4
Lipid Metabolism	HDL	rs538690834	16	68284105	T	C	-0.865	0.053	9.4e-60	7.3e-04	265.8
Lipid Metabolism	HDL	rs116977332	16	68411826	C	T	-0.035	0.006	4.5e-08	2.4e-05	29.9
Lipid Metabolism	HDL	rs148323613	16	68945000	A	C	-0.774	0.102	3.9e-14	9.9e-05	57.2
Lipid Metabolism	HDL	rs568737248	16	68968555	A	G	-0.271	0.031	2.7e-18	1.1e-04	76.1
Lipid Metabolism	HDL	rs77631377	16	69049293	T	C	-0.043	0.004	2.5e-32	1.1e-04	140.1
Lipid Metabolism	HDL	rs150587891	16	69050241	T	C	-0.402	0.036	1.7e-29	1.2e-04	127.1
Lipid Metabolism	HDL	rs189972470	16	69103034	A	G	0.095	0.012	9.0e-16	6.2e-05	64.6
Lipid Metabolism	HDL	rs183415860	16	69104946	C	A	-0.291	0.034	9.2e-18	1.2e-04	73.7
Lipid Metabolism	HDL	rs534785954	16	69295084	A	G	-0.339	0.044	1.0e-14	4.1e-04	59.9
Lipid Metabolism	HDL	rs16958751	16	69357406	A	G	0.027	0.004	2.7e-14	4.7e-05	58.0
Lipid Metabolism	HDL	rs142680585	16	69381489	A	C	0.046	0.007	2.6e-10	3.3e-05	39.9
Lipid Metabolism	HDL	rs11075780	16	70723925	G	A	-0.009	0.001	1.1e-09	3.0e-05	37.2
Lipid Metabolism	HDL	rs544354531	16	70738447	T	C	-0.130	0.023	2.0e-08	3.1e-05	31.5
Lipid Metabolism	HDL	rs2334877	16	71833332	T	C	0.014	0.002	1.0e-13	4.4e-05	55.3
Lipid Metabolism	HDL	rs4788681	16	72974453	A	G	-0.011	0.002	2.4e-11	3.6e-05	44.6
Lipid Metabolism	HDL	rs117107922	16	73067378	T	C	-0.024	0.004	2.0e-08	2.5e-05	31.5
Lipid Metabolism	HDL	rs35754964	16	75184661	T	C	0.011	0.001	2.9e-13	4.3e-05	53.3
Lipid Metabolism	HDL	rs17197883	16	81523013	C	T	0.017	0.002	1.2e-26	9.3e-05	114.2
Lipid Metabolism	HDL	rs2966094	16	81538637	C	A	0.025	0.001	9.9e-66	2.4e-04	293.2
Lipid Metabolism	HDL	rs191022	16	85932132	G	C	0.012	0.002	3.7e-13	4.3e-05	52.8
Lipid Metabolism	HDL	rs9938885	16	86001212	G	A	0.008	0.001	4.7e-09	2.8e-05	34.3
Lipid Metabolism	HDL	rs11117329	16	88008109	A	G	-0.012	0.001	1.7e-16	5.5e-05	67.9
Lipid Metabolism	HDL	rs8060375	16	88507538	T	C	-0.009	0.002	2.7e-09	2.9e-05	35.4
Lipid Metabolism	HDL	rs34345377	17	489296	G	C	-0.015	0.002	8.2e-17	5.6e-05	69.4
Lipid Metabolism	HDL	rs2280460	17	1424012	A	C	0.012	0.002	1.9e-09	2.9e-05	36.1
Lipid Metabolism	HDL	rs216195	17	2203167	G	T	0.009	0.001	1.4e-09	2.9e-05	36.6
Lipid Metabolism	HDL	rs12449758	17	3884026	G	A	-0.011	0.002	8.3e-14	4.5e-05	55.7
Lipid Metabolism	HDL	rs12602989	17	7456489	T	C	-0.018	0.002	7.3e-24	8.2e-05	101.5
Lipid Metabolism	HDL	rs141442459	17	7515256	T	G	0.054	0.009	7.3e-09	2.7e-05	33.5
Lipid Metabolism	HDL	rs6503091	17	8109598	A	G	-0.011	0.001	1.1e-13	4.4e-05	55.1
Lipid Metabolism	HDL	rs9905191	17	17469918	T	C	-0.025	0.003	2.0e-18	6.2e-05	76.7
Lipid Metabolism	HDL	rs854796	17	18070761	A	G	0.010	0.002	9.2e-11	3.4e-05	42.0
Lipid Metabolism	HDL	rs7212510	17	26703682	A	T	-0.009	0.001	3.7e-11	3.5e-05	43.8
Lipid Metabolism	HDL	rs538728506	17	27886635	T	C	-0.101	0.018	1.2e-08	3.1e-05	32.4
Lipid Metabolism	HDL	rs141385558	17	27893545	T	C	-0.098	0.015	2.0e-10	4.0e-05	40.5
Lipid Metabolism	HDL	rs185533580	17	28606498	A	G	-0.105	0.018	3.3e-09	4.3e-05	35.0

Phenotype	Trait	SNP	Chr	Position (GRCh37/hg19)	EA	NEA	BETA	SE	P-value	R2	F
Lipid Metabolism	HDL	rs8071110	17	28672885	G	A	-0.008	0.001	3.1e-08	2.5e-05	30.7
Lipid Metabolism	HDL	rs9906189	17	34905408	G	A	-0.008	0.001	3.1e-08	2.5e-05	30.6
Lipid Metabolism	HDL	rs1877031	17	37814080	A	G	0.030	0.001	1.2e-93	3.4e-04	421.4
Lipid Metabolism	HDL	rs8065443	17	38208940	G	A	0.012	0.001	2.2e-16	5.5e-05	67.4
Lipid Metabolism	HDL	rs12600570	17	40261545	T	C	-0.013	0.002	6.3e-09	2.7e-05	33.7
Lipid Metabolism	HDL	rs180705493	17	40359483	A	G	-0.112	0.014	1.8e-16	5.8e-05	67.8
Lipid Metabolism	HDL	rs34855406	17	40731411	C	G	-0.014	0.002	8.9e-19	6.3e-05	78.3
Lipid Metabolism	HDL	rs627345	17	41714118	G	A	0.024	0.004	1.4e-10	3.3e-05	41.1
Lipid Metabolism	HDL	rs566430451	17	41774038	T	C	-0.165	0.021	1.6e-15	7.9e-05	63.6
Lipid Metabolism	HDL	rs112259268	17	41874745	A	C	-0.190	0.004	1.0e-200	1.6e-03	1979.7
Lipid Metabolism	HDL	rs147596228	17	41926254	T	C	-0.313	0.018	1.0e-66	2.5e-04	297.8
Lipid Metabolism	HDL	rs231541	17	41941788	G	C	0.026	0.002	2.3e-42	1.5e-04	186.1
Lipid Metabolism	HDL	rs560640688	17	42001961	A	G	-0.133	0.019	3.3e-12	6.1e-05	48.5
Lipid Metabolism	HDL	rs571868032	17	42031175	T	A	-0.184	0.025	8.5e-14	6.2e-05	55.7
Lipid Metabolism	HDL	rs191399298	17	42071538	T	A	-0.422	0.059	6.2e-13	6.4e-05	51.8
Lipid Metabolism	HDL	rs113368813	17	42075598	C	T	0.025	0.004	1.9e-11	3.7e-05	45.1
Lipid Metabolism	HDL	rs183676321	17	42191873	A	G	0.053	0.010	2.2e-08	2.6e-05	31.3
Lipid Metabolism	HDL	rs72828813	17	42984085	T	C	-0.062	0.006	9.2e-24	8.2e-05	101.0
Lipid Metabolism	HDL	rs9907503	17	43421115	C	T	-0.017	0.003	9.3e-12	3.7e-05	46.5
Lipid Metabolism	HDL	rs111639757	17	43556661	A	G	-0.031	0.006	3.7e-08	2.5e-05	30.3
Lipid Metabolism	HDL	rs3890958	17	45046865	A	G	0.009	0.001	7.1e-10	3.1e-05	38.0
Lipid Metabolism	HDL	rs9912450	17	45402208	T	C	0.011	0.002	4.4e-12	4.4e-05	47.9
Lipid Metabolism	HDL	rs115271198	17	46319476	T	C	0.028	0.003	1.4e-24	8.4e-05	104.8
Lipid Metabolism	HDL	rs4794008	17	47048961	T	C	0.012	0.002	1.4e-13	4.5e-05	54.7
Lipid Metabolism	HDL	rs28412876	17	47454515	T	G	-0.009	0.001	3.6e-10	3.2e-05	39.3
Lipid Metabolism	HDL	rs11657291	17	53407592	G	A	0.011	0.001	3.4e-14	4.6e-05	57.5
Lipid Metabolism	HDL	rs17834140	17	55465771	T	C	0.019	0.003	6.6e-09	2.7e-05	33.7
Lipid Metabolism	HDL	rs1292066	17	57932314	A	T	-0.012	0.002	1.1e-12	4.1e-05	50.6
Lipid Metabolism	HDL	rs12941370	17	61604736	G	A	-0.008	0.001	3.9e-09	2.8e-05	34.7
Lipid Metabolism	HDL	rs4791048	17	65848994	A	G	-0.014	0.002	2.6e-16	5.4e-05	67.1
Lipid Metabolism	HDL	rs34931250	17	66879927	T	C	-0.050	0.003	3.6e-64	2.3e-04	286.1
Lipid Metabolism	HDL	rs1605750	17	68471073	A	G	0.009	0.001	7.0e-10	3.1e-05	38.0
Lipid Metabolism	HDL	rs7217475	17	70296142	G	A	-0.009	0.001	6.0e-10	3.1e-05	38.3
Lipid Metabolism	HDL	rs9891685	17	73033378	A	G	-0.009	0.002	1.2e-08	2.6e-05	32.4
Lipid Metabolism	HDL	rs113645343	17	74229146	C	T	-0.019	0.003	1.0e-12	4.1e-05	50.8
Lipid Metabolism	HDL	rs16971107	17	76394042	A	G	0.030	0.001	2.7e-101	3.7e-04	456.6
Lipid Metabolism	HDL	rs8075420	17	76431417	A	G	0.010	0.002	7.2e-11	3.5e-05	42.4
Lipid Metabolism	HDL	rs9903134	17	76738386	G	A	0.008	0.001	3.6e-09	2.8e-05	34.9
Lipid Metabolism	HDL	rs17842114	18	100977	G	A	0.010	0.002	3.7e-09	2.8e-05	34.8
Lipid Metabolism	HDL	rs6506032	18	287534	A	G	0.022	0.003	9.6e-17	5.6e-05	69.1
Lipid Metabolism	HDL	rs3934427	18	2979828	C	T	0.015	0.002	1.7e-10	3.3e-05	40.7
Lipid Metabolism	HDL	rs11083157	18	19018225	G	C	-0.011	0.002	3.2e-08	2.5e-05	30.6
Lipid Metabolism	HDL	rs11081879	18	19909866	G	A	0.009	0.001	5.0e-10	3.1e-05	38.7
Lipid Metabolism	HDL	rs178008	18	20093905	A	T	-0.009	0.002	2.4e-08	2.5e-05	31.1
Lipid Metabolism	HDL	rs1623060	18	21143183	T	C	0.013	0.001	3.9e-21	7.2e-05	89.0
Lipid Metabolism	HDL	rs9956721	18	22621869	A	T	-0.008	0.001	6.5e-09	2.7e-05	33.7
Lipid Metabolism	HDL	rs190691924	18	29501708	T	C	-0.164	0.024	1.5e-11	6.4e-05	45.5
Lipid Metabolism	HDL	rs144242131	18	29769910	A	G	-0.188	0.024	2.9e-15	7.8e-05	62.3
Lipid Metabolism	HDL	rs673408	18	29800119	G	A	0.009	0.001	5.7e-10	3.1e-05	38.4
Lipid Metabolism	HDL	rs567424502	18	29991345	T	C	-0.207	0.028	1.4e-13	9.9e-05	54.8
Lipid Metabolism	HDL	rs8086460	18	31270819	T	C	-0.008	0.001	1.9e-09	2.9e-05	36.1
Lipid Metabolism	HDL	rs7242026	18	40702833	C	T	0.011	0.001	1.4e-15	5.1e-05	63.7
Lipid Metabolism	HDL	rs4890501	18	42599987	G	A	-0.008	0.001	2.5e-08	2.5e-05	31.0
Lipid Metabolism	HDL	rs543062797	18	46307606	C	T	0.131	0.019	1.2e-11	5.9e-05	46.0
Lipid Metabolism	HDL	rs183119479	18	46342691	A	G	0.139	0.018	3.9e-14	7.2e-05	57.2
Lipid Metabolism	HDL	rs9963479	18	46532088	G	A	0.013	0.001	5.3e-19	6.5e-05	79.3

Phenotype	Trait	SNP	Chr	Position (GRCh37/hg19)	EA	NEA	BETA	SE	P-value	R2	F
Lipid Metabolism	HDL	rs112138271	18	46613435	G	A	0.102	0.017	1.5e-09	3.1e-05	36.6
Lipid Metabolism	HDL	rs7229507	18	46620107	T	C	0.023	0.003	4.1e-13	4.2e-05	52.6
Lipid Metabolism	HDL	rs143015228	18	46638218	C	G	0.056	0.009	1.2e-10	3.4e-05	41.5
Lipid Metabolism	HDL	rs188574990	18	46659191	G	A	0.124	0.010	1.1e-33	1.2e-04	146.3
Lipid Metabolism	HDL	rs377524871	18	46678789	A	G	0.533	0.067	1.6e-15	1.2e-04	63.5
Lipid Metabolism	HDL	rs546175659	18	47039412	T	C	0.123	0.021	6.9e-09	3.8e-05	33.6
Lipid Metabolism	HDL	rs74913936	18	47085818	A	T	-0.117	0.020	9.7e-09	4.6e-05	32.9
Lipid Metabolism	HDL	rs147418632	18	47089986	A	G	0.092	0.016	6.2e-09	2.9e-05	33.8
Lipid Metabolism	HDL	rs149615216	18	47106028	T	C	0.262	0.007	1.0e-200	1.2e-03	1530.7
Lipid Metabolism	HDL	rs117316234	18	47117616	T	G	0.056	0.008	2.9e-12	4.0e-05	48.8
Lipid Metabolism	HDL	rs570076017	18	47153746	C	A	-0.200	0.036	3.3e-08	3.6e-05	30.5
Lipid Metabolism	HDL	rs7240405	18	47159090	G	A	0.074	0.002	1.0e-200	1.3e-03	1675.1
Lipid Metabolism	HDL	rs569057272	18	47165270	T	A	0.408	0.044	4.3e-20	2.3e-04	84.3
Lipid Metabolism	HDL	rs117451311	18	47180845	A	G	-0.051	0.007	1.4e-13	4.4e-05	54.6
Lipid Metabolism	HDL	rs532078033	18	47193944	A	G	0.254	0.030	1.4e-17	1.1e-04	72.8
Lipid Metabolism	HDL	rs11874381	18	47203408	A	G	0.035	0.001	2.8e-133	4.9e-04	603.6
Lipid Metabolism	HDL	rs185432346	18	47283792	T	G	-0.040	0.007	7.7e-09	2.7e-05	33.3
Lipid Metabolism	HDL	rs190932527	18	47557160	A	G	0.313	0.035	4.5e-19	2.2e-04	79.6
Lipid Metabolism	HDL	rs17660048	18	47631526	A	G	-0.033	0.005	1.2e-09	3.0e-05	37.0
Lipid Metabolism	HDL	rs185232623	18	47638315	G	A	-0.063	0.007	1.2e-17	5.9e-05	73.2
Lipid Metabolism	HDL	rs150075950	18	47692905	C	A	0.275	0.028	3.6e-23	2.6e-04	98.3
Lipid Metabolism	HDL	rs143518209	18	48067072	A	T	0.102	0.018	2.9e-08	2.8e-05	30.8
Lipid Metabolism	HDL	rs768048	18	50285398	T	C	-0.013	0.002	4.7e-10	3.1e-05	38.8
Lipid Metabolism	HDL	rs67290736	18	56082558	T	C	-0.018	0.003	1.4e-08	2.6e-05	32.2
Lipid Metabolism	HDL	rs41292412	18	56118358	T	C	-0.061	0.007	2.3e-17	5.8e-05	71.9
Lipid Metabolism	HDL	rs8089364	18	57858829	C	T	-0.018	0.002	5.2e-29	1.0e-04	125.0
Lipid Metabolism	HDL	rs113265974	18	58062940	A	G	0.041	0.005	2.9e-18	6.1e-05	75.9
Lipid Metabolism	HDL	rs17070809	18	60846738	A	G	0.012	0.002	8.4e-13	4.1e-05	51.2
Lipid Metabolism	HDL	rs878893	19	2781325	G	A	0.010	0.002	4.3e-09	2.8e-05	34.5
Lipid Metabolism	HDL	rs12975319	19	3414088	A	G	-0.015	0.002	9.0e-24	8.3e-05	101.0
Lipid Metabolism	HDL	rs58519763	19	4058395	A	G	-0.021	0.002	5.8e-18	6.2e-05	74.6
Lipid Metabolism	HDL	rs2890215	19	4116081	T	A	0.020	0.003	5.1e-10	3.1e-05	38.6
Lipid Metabolism	HDL	rs10413045	19	5024099	T	G	-0.010	0.001	2.4e-12	4.0e-05	49.1
Lipid Metabolism	HDL	rs6510959	19	7184238	A	G	0.011	0.002	3.9e-10	3.1e-05	39.2
Lipid Metabolism	HDL	rs8100562	19	7241501	T	C	-0.017	0.002	4.7e-22	7.5e-05	93.2
Lipid Metabolism	HDL	rs2287909	19	7506668	G	T	0.008	0.001	9.9e-09	2.6e-05	32.9
Lipid Metabolism	HDL	rs2115108	19	7963976	C	T	0.014	0.002	1.5e-19	6.7e-05	81.7
Lipid Metabolism	HDL	rs139473010	19	8416806	A	C	0.039	0.004	5.4e-18	6.0e-05	74.7
Lipid Metabolism	HDL	rs79293855	19	8420583	A	G	0.047	0.004	3.5e-33	1.2e-04	144.0
Lipid Metabolism	HDL	rs116843064	19	8429323	A	G	0.215	0.005	1.0e-200	1.5e-03	1884.4
Lipid Metabolism	HDL	rs4488592	19	8640452	G	A	-0.009	0.002	6.8e-09	2.8e-05	33.6
Lipid Metabolism	HDL	rs10469364	19	11274679	A	G	0.014	0.001	7.9e-24	8.2e-05	101.3
Lipid Metabolism	HDL	rs56322906	19	11346155	A	G	-0.079	0.003	2.8e-113	4.1e-04	511.6
Lipid Metabolism	HDL	rs8106047	19	18505741	A	T	-0.013	0.002	2.5e-11	3.6e-05	44.5
Lipid Metabolism	HDL	rs56195015	19	33785141	T	C	0.018	0.002	3.1e-13	4.4e-05	53.2
Lipid Metabolism	HDL	rs4805881	19	33896432	C	A	0.020	0.001	4.9e-41	1.5e-04	180.0
Lipid Metabolism	HDL	rs12610663	19	35492129	T	C	0.011	0.002	9.6e-14	4.5e-05	55.4
Lipid Metabolism	HDL	rs2258314	19	41338835	T	C	0.016	0.003	2.0e-09	3.0e-05	35.9
Lipid Metabolism	HDL	rs73045269	19	41825191	T	C	0.016	0.002	1.9e-17	5.9e-05	72.2
Lipid Metabolism	HDL	rs4760	19	44153100	G	A	-0.017	0.002	1.3e-17	5.9e-05	73.0
Lipid Metabolism	HDL	rs140105611	19	45318745	A	G	-0.131	0.022	3.2e-09	4.3e-05	35.1
Lipid Metabolism	HDL	rs118147862	19	45319631	A	G	0.074	0.004	2.5e-91	3.3e-04	410.8
Lipid Metabolism	HDL	rs190387002	19	45331542	C	G	-0.087	0.015	4.5e-09	3.3e-05	34.4
Lipid Metabolism	HDL	rs111371860	19	45345787	T	A	0.032	0.003	2.9e-30	1.1e-04	130.7
Lipid Metabolism	HDL	rs117310449	19	45393516	T	C	-0.077	0.006	2.8e-33	1.2e-04	144.5
Lipid Metabolism	HDL	rs769449	19	45410002	A	G	-0.085	0.002	1.0e-200	1.3e-03	1621.4

Phenotype	Trait	SNP	Chr	Position (GRCh37/hg19)	EA	NEA	BETA	SE	P-value	R2	F
Lipid Metabolism	HDL	rs182067414	19	45425954	C	T	0.098	0.013	5.7e-14	4.7e-05	56.5
Lipid Metabolism	HDL	rs541817716	19	45433531	A	T	-0.172	0.028	5.1e-10	4.1e-05	38.6
Lipid Metabolism	HDL	rs5157	19	45447161	C	T	0.037	0.001	2.3e-149	5.5e-04	677.5
Lipid Metabolism	HDL	rs185625998	19	45505348	C	T	0.033	0.005	1.5e-11	4.3e-05	45.6
Lipid Metabolism	HDL	rs35070962	19	45653040	A	G	0.043	0.006	2.7e-12	4.0e-05	48.9
Lipid Metabolism	HDL	rs202193462	19	46048053	T	A	-0.175	0.028	3.0e-10	5.3e-05	39.7
Lipid Metabolism	HDL	rs8107376	19	46163769	C	T	-0.014	0.002	3.1e-19	6.5e-05	80.4
Lipid Metabolism	HDL	rs34255979	19	46384830	T	C	-0.030	0.002	2.2e-44	1.6e-04	195.3
Lipid Metabolism	HDL	rs12609461	19	47581464	C	A	-0.016	0.001	5.4e-26	9.1e-05	111.2
Lipid Metabolism	HDL	rs10413478	19	48106345	C	T	0.018	0.002	1.9e-18	6.2e-05	76.8
Lipid Metabolism	HDL	rs572347870	19	49496539	G	C	-0.465	0.085	3.8e-08	8.0e-05	30.2
Lipid Metabolism	HDL	rs10469298	19	50024391	G	A	0.011	0.002	5.8e-10	3.1e-05	38.4
Lipid Metabolism	HDL	rs7247412	19	52313868	C	T	-0.021	0.002	9.0e-40	1.4e-04	174.2
Lipid Metabolism	HDL	rs11670364	19	53601172	T	C	0.010	0.002	5.6e-11	3.5e-05	43.0
Lipid Metabolism	HDL	rs11669763	19	54750334	A	C	0.021	0.003	1.3e-13	4.5e-05	54.9
Lipid Metabolism	HDL	rs103294	19	54797848	T	C	0.055	0.002	1.0e-200	8.7e-04	1083.4
Lipid Metabolism	HDL	rs12981222	19	56154339	T	C	-0.014	0.002	9.8e-12	3.7e-05	46.4
Lipid Metabolism	HDL	rs12978189	19	57487813	C	T	-0.009	0.001	4.1e-11	3.5e-05	43.6
Lipid Metabolism	HDL	rs73602128	20	566768	C	T	0.017	0.003	1.6e-10	3.3e-05	41.0
Lipid Metabolism	HDL	rs151235402	20	569164	T	C	-0.068	0.006	2.9e-34	1.2e-04	149.0
Lipid Metabolism	HDL	rs6051445	20	2836657	A	T	0.008	0.001	1.5e-08	2.6e-05	32.1
Lipid Metabolism	HDL	rs62194921	20	6712689	T	C	0.017	0.003	1.3e-09	3.0e-05	36.8
Lipid Metabolism	HDL	rs1321940	20	12959885	G	A	0.008	0.001	1.9e-08	2.5e-05	31.6
Lipid Metabolism	HDL	rs1054787	20	17600114	C	G	-0.018	0.002	1.4e-21	7.3e-05	91.0
Lipid Metabolism	HDL	rs6047730	20	21897493	C	G	-0.009	0.001	7.1e-10	3.1e-05	38.0
Lipid Metabolism	HDL	rs7266471	20	30220599	A	T	0.015	0.002	1.7e-16	5.5e-05	67.9
Lipid Metabolism	HDL	rs12624640	20	32952125	A	G	-0.018	0.001	1.3e-36	1.3e-04	159.7
Lipid Metabolism	HDL	rs2425023	20	33832494	C	T	-0.016	0.001	1.3e-27	9.6e-05	118.6
Lipid Metabolism	HDL	rs2345386	20	36835420	T	C	0.008	0.001	2.7e-08	2.5e-05	30.9
Lipid Metabolism	HDL	rs2224539	20	38552107	T	A	0.009	0.001	1.3e-09	3.0e-05	36.8
Lipid Metabolism	HDL	rs6016378	20	39175267	T	G	0.008	0.002	3.0e-08	2.5e-05	30.7
Lipid Metabolism	HDL	rs2093139	20	42255864	A	G	-0.009	0.002	4.8e-09	2.8e-05	34.3
Lipid Metabolism	HDL	rs1800961	20	43042364	T	C	-0.134	0.004	1.0e-200	9.9e-04	1223.1
Lipid Metabolism	HDL	rs73910253	20	44184056	T	G	-0.037	0.006	1.2e-10	3.3e-05	41.5
Lipid Metabolism	HDL	rs74174967	20	44386234	T	C	0.024	0.004	7.2e-09	2.8e-05	33.5
Lipid Metabolism	HDL	rs6065908	20	44569930	T	C	-0.060	0.002	1.0e-200	9.1e-04	1137.1
Lipid Metabolism	HDL	rs148342289	20	44757526	T	C	-0.082	0.015	3.7e-08	2.9e-05	30.3
Lipid Metabolism	HDL	rs2143698	20	44759161	A	G	-0.018	0.002	3.5e-16	5.3e-05	66.5
Lipid Metabolism	HDL	rs73304093	20	45953341	T	C	0.018	0.003	1.6e-12	4.0e-05	49.9
Lipid Metabolism	HDL	rs11086219	20	46274848	A	C	0.014	0.002	3.8e-19	6.5e-05	80.0
Lipid Metabolism	HDL	rs6125085	20	46344574	C	T	-0.016	0.001	1.4e-28	1.0e-04	123.0
Lipid Metabolism	HDL	rs150290140	20	46454972	C	T	0.035	0.004	7.8e-18	6.0e-05	74.0
Lipid Metabolism	HDL	rs6013456	20	51033123	C	A	0.011	0.001	4.6e-14	4.6e-05	56.9
Lipid Metabolism	HDL	rs6123685	20	55836040	A	G	0.013	0.002	1.5e-14	4.9e-05	59.2
Lipid Metabolism	HDL	rs1062600	20	56137798	A	G	-0.009	0.001	2.6e-11	3.6e-05	44.4
Lipid Metabolism	HDL	rs76602912	20	57459868	C	T	-0.031	0.005	6.0e-10	3.1e-05	38.3
Lipid Metabolism	HDL	rs140896965	20	57648366	T	C	0.040	0.007	1.0e-08	2.7e-05	32.8
Lipid Metabolism	HDL	rs4809369	20	62470785	A	G	0.009	0.001	1.3e-10	3.4e-05	41.4
Lipid Metabolism	HDL	rs8126001	20	62711459	T	C	0.014	0.001	4.4e-20	8.1e-05	84.2
Lipid Metabolism	HDL	rs8133958	21	16844896	A	G	-0.010	0.002	5.0e-09	2.8e-05	34.2
Lipid Metabolism	HDL	rs2832299	21	30749237	C	G	-0.010	0.001	3.6e-12	3.9e-05	48.4
Lipid Metabolism	HDL	rs2834707	21	36343552	T	C	-0.008	0.001	1.7e-08	2.6e-05	31.8
Lipid Metabolism	HDL	rs2282108	21	37707768	T	C	-0.009	0.001	3.2e-11	3.6e-05	44.1
Lipid Metabolism	HDL	rs62222270	21	38750935	A	G	0.012	0.002	9.6e-09	2.6e-05	32.9
Lipid Metabolism	HDL	rs9975388	21	42626362	A	C	-0.010	0.001	3.8e-13	4.2e-05	52.7
Lipid Metabolism	HDL	rs11910395	21	43722586	T	C	-0.022	0.003	5.3e-11	3.5e-05	43.1

Phenotype	Trait	SNP	Chr	Position (GRCh37/hg19)	EA	NEA	BETA	SE	P-value	R2	F
Lipid Metabolism	HDL	rs235314	21	46271452	T	C	-0.018	0.001	7.5e-37	1.3e-04	160.8
Lipid Metabolism	HDL	rs2838788	21	46542374	C	T	0.008	0.001	3.7e-08	2.5e-05	30.3
Lipid Metabolism	HDL	rs9976784	21	46907479	A	G	-0.019	0.002	4.7e-27	9.4e-05	116.0
Lipid Metabolism	HDL	rs181362	22	21932068	T	C	-0.036	0.002	2.2e-100	3.6e-04	452.4
Lipid Metabolism	HDL	rs2142561	22	28374216	C	T	0.018	0.003	6.0e-09	2.7e-05	33.8
Lipid Metabolism	HDL	rs28613006	22	29087142	C	G	-0.011	0.002	3.0e-08	2.9e-05	30.7
Lipid Metabolism	HDL	rs186430430	22	29103598	C	T	-0.091	0.015	1.4e-09	3.1e-05	36.6
Lipid Metabolism	HDL	rs2179129	22	29450923	G	A	0.009	0.001	2.9e-10	3.2e-05	39.7
Lipid Metabolism	HDL	rs41156	22	30404653	C	T	0.010	0.001	7.1e-12	3.8e-05	47.0
Lipid Metabolism	HDL	rs9608956	22	30901592	C	T	-0.017	0.002	1.7e-25	8.8e-05	108.9
Lipid Metabolism	HDL	rs12167681	22	32334686	A	G	-0.010	0.002	9.9e-09	2.6e-05	32.9
Lipid Metabolism	HDL	rs3819663	22	33965639	G	A	-0.008	0.001	5.0e-08	2.5e-05	29.7
Lipid Metabolism	HDL	rs5750141	22	36034983	C	T	-0.009	0.001	5.5e-10	3.1e-05	38.5
Lipid Metabolism	HDL	rs2267375	22	38601231	T	G	-0.020	0.001	6.9e-44	1.6e-04	193.0
Lipid Metabolism	HDL	rs1946990	22	38912771	G	A	-0.012	0.001	3.2e-16	5.4e-05	66.7
Lipid Metabolism	HDL	rs7287294	22	41775541	G	A	0.008	0.001	1.6e-08	2.6e-05	32.0
Lipid Metabolism	HDL	rs2294922	22	44379565	C	G	-0.012	0.002	1.0e-13	4.4e-05	55.3
Lipid Metabolism	HDL	rs13056230	22	48874310	T	C	-0.008	0.001	1.4e-09	3.0e-05	36.7
Lipid Metabolism	LDL	rs1123571	1	2326009	A	G	-0.011	0.001	1.3e-14	5.0e-05	59.3
Lipid Metabolism	LDL	rs35365738	1	16068364	A	G	-0.009	0.002	1.6e-08	2.6e-05	31.9
Lipid Metabolism	LDL	rs924204	1	16513926	G	A	0.014	0.001	7.3e-22	7.6e-05	92.3
Lipid Metabolism	LDL	rs1360916	1	18801697	A	G	-0.010	0.001	2.6e-12	4.0e-05	49.0
Lipid Metabolism	LDL	rs80295797	1	23341690	T	C	0.011	0.002	7.2e-14	4.6e-05	56.0
Lipid Metabolism	LDL	rs612177	1	23792009	T	G	0.018	0.002	3.1e-14	4.8e-05	57.7
Lipid Metabolism	LDL	rs10903129	1	25768937	G	A	0.026	0.001	2.8e-77	2.8e-04	346.3
Lipid Metabolism	LDL	rs56214942	1	25891959	A	G	0.011	0.001	6.1e-14	4.7e-05	56.3
Lipid Metabolism	LDL	rs76713804	1	26392437	A	G	-0.013	0.002	5.9e-13	4.2e-05	51.9
Lipid Metabolism	LDL	rs114165349	1	27021913	C	G	0.084	0.005	5.5e-73	2.7e-04	326.5
Lipid Metabolism	LDL	rs2483719	1	29468198	T	C	-0.009	0.002	2.6e-08	2.5e-05	31.0
Lipid Metabolism	LDL	rs4329504	1	39451488	G	A	-0.008	0.001	9.6e-09	2.7e-05	32.9
Lipid Metabolism	LDL	rs638769	1	42647030	G	C	-0.008	0.002	3.5e-08	2.5e-05	30.4
Lipid Metabolism	LDL	rs531458869	1	45283153	T	C	-0.515	0.085	1.8e-09	5.7e-05	36.2
Lipid Metabolism	LDL	rs188254602	1	45462248	C	T	0.087	0.016	3.0e-08	2.6e-05	30.7
Lipid Metabolism	LDL	rs17853159	1	45810865	A	G	-0.022	0.003	4.2e-18	6.1e-05	75.2
Lipid Metabolism	LDL	rs72677557	1	46602345	C	T	0.010	0.002	1.8e-08	2.6e-05	31.7
Lipid Metabolism	LDL	rs547856250	1	49263479	A	G	-0.137	0.019	1.1e-12	7.6e-05	50.6
Lipid Metabolism	LDL	rs185716614	1	51068058	C	A	-0.149	0.018	1.1e-16	9.7e-05	68.8
Lipid Metabolism	LDL	rs570553136	1	52262405	G	A	-0.167	0.019	2.6e-19	1.3e-04	80.7
Lipid Metabolism	LDL	rs191875604	1	54246772	C	T	-0.193	0.017	6.2e-30	1.4e-04	129.2
Lipid Metabolism	LDL	rs563391624	1	54748004	T	C	-1.658	0.160	4.1e-25	2.6e-04	107.2
Lipid Metabolism	LDL	rs192958267	1	55218101	T	C	-0.652	0.056	2.3e-31	3.4e-04	135.7
Lipid Metabolism	LDL	rs576264565	1	55272126	A	C	0.065	0.011	8.1e-09	2.8e-05	33.3
Lipid Metabolism	LDL	rs182928365	1	55316058	G	T	-0.615	0.099	4.9e-10	8.0e-05	38.7
Lipid Metabolism	LDL	rs561554246	1	55453057	G	A	-0.429	0.052	1.7e-16	1.7e-04	67.9
Lipid Metabolism	LDL	rs34232196	1	55489542	T	C	-0.066	0.002	1.0e-200	1.3e-03	1632.3
Lipid Metabolism	LDL	rs28385700	1	55504401	T	C	0.051	0.009	5.7e-09	2.8e-05	33.9
Lipid Metabolism	LDL	rs11800243	1	55518093	A	G	-0.040	0.004	8.0e-30	1.1e-04	128.7
Lipid Metabolism	LDL	rs148195424	1	55518374	T	C	-0.555	0.043	8.5e-38	2.4e-04	165.1
Lipid Metabolism	LDL	rs45508296	1	55520547	G	A	-0.065	0.005	2.0e-33	1.2e-04	145.1
Lipid Metabolism	LDL	rs539259897	1	55529944	C	G	-0.701	0.101	4.0e-12	1.2e-04	48.1
Lipid Metabolism	LDL	rs192276219	1	55532511	G	A	-0.041	0.007	3.2e-08	2.5e-05	30.6
Lipid Metabolism	LDL	rs77333170	1	55553210	T	A	-0.038	0.004	1.4e-23	8.1e-05	100.2
Lipid Metabolism	LDL	rs181149007	1	55562928	T	G	-0.592	0.082	3.8e-13	1.3e-04	52.7
Lipid Metabolism	LDL	rs115460230	1	55566699	T	C	-0.052	0.009	9.5e-10	3.1e-05	37.4
Lipid Metabolism	LDL	rs139471498	1	55584033	C	T	0.085	0.012	1.8e-13	4.5e-05	54.3
Lipid Metabolism	LDL	rs575331971	1	55596509	G	A	-0.358	0.055	1.1e-10	1.0e-04	41.6

Phenotype	Trait	SNP	Chr	Position (GRCh37/hg19)	EA	NEA	BETA	SE	P-value	R2	F
Lipid Metabolism	LDL	rs574121437	1	55600247	A	G	0.129	0.022	3.4e-09	8.6e-05	35.0
Lipid Metabolism	LDL	rs553629879	1	55602330	C	T	0.136	0.015	2.9e-19	6.8e-05	80.5
Lipid Metabolism	LDL	rs564951008	1	55624448	C	T	-0.431	0.061	1.1e-12	1.3e-04	50.7
Lipid Metabolism	LDL	rs1475701	1	55638546	C	T	0.087	0.004	3.3e-123	4.5e-04	557.3
Lipid Metabolism	LDL	rs148742777	1	55647559	C	T	0.062	0.007	5.7e-17	6.3e-05	70.1
Lipid Metabolism	LDL	rs544896149	1	55663881	G	C	0.081	0.012	2.8e-12	4.0e-05	48.8
Lipid Metabolism	LDL	rs12750160	1	55674000	T	C	-0.305	0.008	1.0e-200	1.2e-03	1503.8
Lipid Metabolism	LDL	rs116378305	1	55699161	T	G	0.072	0.007	9.0e-25	8.6e-05	105.6
Lipid Metabolism	LDL	rs79685888	1	55734545	G	A	0.034	0.003	4.0e-25	8.8e-05	107.2
Lipid Metabolism	LDL	rs115980985	1	55746471	C	T	-0.083	0.014	2.7e-09	3.0e-05	35.4
Lipid Metabolism	LDL	rs150088310	1	55757529	A	G	0.043	0.006	1.9e-13	4.4e-05	54.1
Lipid Metabolism	LDL	rs115449167	1	55780468	C	A	0.042	0.005	5.0e-18	6.1e-05	74.9
Lipid Metabolism	LDL	rs183378272	1	55881468	C	T	-0.448	0.078	8.8e-09	8.2e-05	33.1
Lipid Metabolism	LDL	rs142027040	1	55938051	G	A	-1.092	0.071	5.1e-53	6.0e-04	234.9
Lipid Metabolism	LDL	rs188026950	1	55939497	A	G	-0.352	0.010	1.0e-200	1.0e-03	1223.1
Lipid Metabolism	LDL	rs570729995	1	56196706	G	A	-0.288	0.040	3.0e-13	6.4e-05	53.2
Lipid Metabolism	LDL	rs189870580	1	56454599	T	G	-0.196	0.026	5.4e-14	5.9e-05	56.6
Lipid Metabolism	LDL	rs558153898	1	56574672	A	G	-0.405	0.058	4.2e-12	8.8e-05	48.0
Lipid Metabolism	LDL	rs565120608	1	56675202	A	G	-0.173	0.029	1.5e-09	4.3e-05	36.5
Lipid Metabolism	LDL	rs17113546	1	56713183	G	A	-0.144	0.025	5.5e-09	4.4e-05	34.0
Lipid Metabolism	LDL	rs186907611	1	56995693	G	A	-0.324	0.021	2.8e-54	3.6e-04	240.6
Lipid Metabolism	LDL	rs78519632	1	57028940	T	C	-0.046	0.007	6.4e-12	3.8e-05	47.2
Lipid Metabolism	LDL	rs184826255	1	57156173	C	T	-0.152	0.016	5.1e-21	8.0e-05	88.5
Lipid Metabolism	LDL	rs17121798	1	61675857	G	C	-0.018	0.003	1.1e-11	3.7e-05	46.1
Lipid Metabolism	LDL	rs75954052	1	62769144	T	C	0.014	0.003	4.5e-08	2.4e-05	29.9
Lipid Metabolism	LDL	rs548994316	1	62887772	T	C	-0.282	0.049	9.3e-09	5.3e-05	33.0
Lipid Metabolism	LDL	rs598253	1	62911341	C	T	-0.042	0.001	6.5e-174	6.5e-04	790.4
Lipid Metabolism	LDL	rs55763644	1	62961001	A	G	-0.431	0.077	1.9e-08	6.8e-05	31.6
Lipid Metabolism	LDL	rs559647195	1	63352650	T	C	-0.177	0.032	4.9e-08	7.5e-05	29.7
Lipid Metabolism	LDL	rs549777175	1	63413521	A	G	-0.208	0.030	2.4e-12	6.5e-05	49.1
Lipid Metabolism	LDL	rs185873223	1	63426178	C	T	-0.937	0.163	9.4e-09	8.3e-05	33.0
Lipid Metabolism	LDL	rs6684364	1	78574129	A	G	-0.010	0.002	3.3e-09	2.8e-05	35.0
Lipid Metabolism	LDL	rs568052947	1	79741522	T	A	-0.383	0.070	3.6e-08	6.8e-05	30.3
Lipid Metabolism	LDL	rs165316	1	91533297	G	A	-0.010	0.002	1.4e-08	2.6e-05	32.2
Lipid Metabolism	LDL	rs962222	1	92970953	T	C	0.023	0.002	4.0e-42	1.5e-04	185.0
Lipid Metabolism	LDL	rs143968008	1	93181359	A	G	0.058	0.008	7.1e-13	4.2e-05	51.5
Lipid Metabolism	LDL	rs56111229	1	107566689	A	T	0.012	0.001	9.0e-17	5.6e-05	69.2
Lipid Metabolism	LDL	rs114254196	1	108635400	T	C	-0.045	0.008	4.4e-08	2.5e-05	30.0
Lipid Metabolism	LDL	rs188443112	1	109389899	A	G	-0.086	0.010	6.0e-17	5.7e-05	70.0
Lipid Metabolism	LDL	rs146765950	1	109682276	G	A	-0.064	0.005	2.6e-38	1.4e-04	167.5
Lipid Metabolism	LDL	rs34364548	1	109714635	T	C	0.029	0.004	7.9e-13	4.2e-05	51.3
Lipid Metabolism	LDL	rs114713488	1	109751285	C	A	0.038	0.004	1.8e-19	6.6e-05	81.5
Lipid Metabolism	LDL	rs4970829	1	109757295	A	G	-0.104	0.003	1.0e-200	1.2e-03	1494.4
Lipid Metabolism	LDL	rs561691858	1	109762187	G	T	-0.121	0.022	4.4e-08	4.0e-05	30.0
Lipid Metabolism	LDL	rs35271870	1	109776285	C	T	-0.117	0.003	1.0e-200	1.8e-03	2164.6
Lipid Metabolism	LDL	rs575507604	1	109785599	T	C	-0.190	0.022	1.6e-18	8.3e-05	77.1
Lipid Metabolism	LDL	rs143238579	1	109797954	A	G	0.046	0.005	1.4e-18	6.3e-05	77.5
Lipid Metabolism	LDL	rs6680227	1	109798914	A	G	-0.087	0.004	1.0e-116	4.3e-04	527.5
Lipid Metabolism	LDL	rs78852738	1	109800086	C	A	0.042	0.005	6.6e-18	6.1e-05	74.3
Lipid Metabolism	LDL	rs659782	1	109817947	T	C	0.034	0.003	1.5e-33	1.2e-04	145.7
Lipid Metabolism	LDL	rs543600517	1	109823291	T	C	-0.136	0.023	3.1e-09	3.8e-05	35.1
Lipid Metabolism	LDL	rs138361368	1	109825558	A	C	-0.094	0.005	8.2e-74	2.7e-04	330.3
Lipid Metabolism	LDL	rs148631275	1	109927624	C	T	0.034	0.005	6.8e-10	3.1e-05	38.1
Lipid Metabolism	LDL	rs72703257	1	109942924	T	C	0.037	0.005	3.9e-15	5.0e-05	61.7
Lipid Metabolism	LDL	rs61797139	1	110006665	T	C	0.030	0.003	4.4e-24	8.5e-05	102.5
Lipid Metabolism	LDL	rs145291952	1	110013402	C	G	-0.082	0.012	3.5e-11	3.7e-05	43.9

Phenotype	Trait	SNP	Chr	Position (GRCh37/hg19)	EA	NEA	BETA	SE	P-value	R2	F
Lipid Metabolism	LDL	rs77956617	1	110029023	T	C	-0.128	0.021	1.6e-09	6.0e-05	36.4
Lipid Metabolism	LDL	rs41279740	1	110085511	T	C	0.050	0.008	1.3e-10	3.4e-05	41.4
Lipid Metabolism	LDL	rs186952258	1	110124850	T	C	-0.087	0.016	2.7e-08	2.9e-05	30.9
Lipid Metabolism	LDL	rs56116536	1	110131439	T	C	0.028	0.004	4.5e-11	3.5e-05	43.4
Lipid Metabolism	LDL	rs2243393	1	111726026	A	G	-0.008	0.001	3.7e-09	2.8e-05	34.8
Lipid Metabolism	LDL	rs11102709	1	114512083	C	G	-0.009	0.002	2.0e-08	2.6e-05	31.5
Lipid Metabolism	LDL	rs267733	1	150958836	G	A	-0.019	0.002	8.3e-23	7.9e-05	96.7
Lipid Metabolism	LDL	rs184980867	1	151658543	A	G	-0.113	0.013	5.3e-17	5.9e-05	70.2
Lipid Metabolism	LDL	rs113462784	1	154044453	C	T	0.015	0.003	2.2e-08	3.0e-05	31.3
Lipid Metabolism	LDL	rs4390169	1	155106054	G	A	-0.012	0.001	5.5e-18	6.1e-05	74.7
Lipid Metabolism	LDL	rs2022003	1	158586966	T	A	0.010	0.002	3.5e-11	3.6e-05	43.9
Lipid Metabolism	LDL	rs3754459	1	159013044	C	T	-0.018	0.003	4.2e-08	2.4e-05	30.1
Lipid Metabolism	LDL	rs115383270	1	161531340	A	G	0.019	0.003	2.0e-12	4.1e-05	49.4
Lipid Metabolism	LDL	rs10912842	1	171334827	G	C	0.009	0.002	4.1e-08	2.8e-05	30.1
Lipid Metabolism	LDL	rs2933547	1	172625154	G	A	0.009	0.001	2.3e-09	2.9e-05	35.7
Lipid Metabolism	LDL	rs76900682	1	174064560	A	G	0.013	0.002	5.5e-11	3.5e-05	43.0
Lipid Metabolism	LDL	rs6682862	1	177938437	A	G	-0.014	0.002	3.5e-13	4.3e-05	52.9
Lipid Metabolism	LDL	rs1689801	1	182165484	A	G	0.014	0.001	1.8e-22	7.7e-05	95.2
Lipid Metabolism	LDL	rs12086466	1	183075778	C	T	0.011	0.001	1.1e-15	5.2e-05	64.2
Lipid Metabolism	LDL	rs1434282	1	199010721	T	C	0.011	0.002	3.8e-12	4.0e-05	48.2
Lipid Metabolism	LDL	rs2491258	1	221033754	G	A	0.019	0.001	4.6e-39	1.4e-04	170.9
Lipid Metabolism	LDL	rs1414520	1	221121399	A	G	0.012	0.001	1.4e-16	5.6e-05	68.3
Lipid Metabolism	LDL	rs11803981	1	224545410	C	T	-0.011	0.002	2.8e-11	3.6e-05	44.3
Lipid Metabolism	LDL	rs12144137	1	224654854	T	C	-0.024	0.004	2.5e-10	3.3e-05	40.0
Lipid Metabolism	LDL	rs7543281	1	234743237	A	G	0.023	0.002	1.4e-28	1.0e-04	122.9
Lipid Metabolism	LDL	rs2587534	1	234849339	A	G	0.041	0.001	2.1e-180	6.7e-04	820.3
Lipid Metabolism	LDL	rs10797566	1	235011530	G	T	-0.016	0.001	6.9e-30	1.1e-04	129.0
Lipid Metabolism	LDL	rs16844292	1	235099547	C	T	-0.015	0.002	9.5e-12	3.8e-05	46.4
Lipid Metabolism	LDL	rs12085823	1	246920514	C	T	0.008	0.001	1.9e-09	2.9e-05	36.0
Lipid Metabolism	LDL	rs3820897	2	3642361	C	T	-0.016	0.002	2.3e-19	6.7e-05	81.0
Lipid Metabolism	LDL	rs67269656	2	8720038	T	C	-0.010	0.002	4.2e-11	3.5e-05	43.5
Lipid Metabolism	LDL	rs7556983	2	17955255	A	G	-0.015	0.002	1.3e-11	3.7e-05	45.8
Lipid Metabolism	LDL	rs870527	2	20369701	A	T	-0.016	0.001	2.8e-29	1.0e-04	126.2
Lipid Metabolism	LDL	rs559345870	2	20864070	G	C	-1.247	0.150	7.9e-17	1.7e-04	69.4
Lipid Metabolism	LDL	rs139095428	2	20899937	A	C	-0.171	0.023	2.2e-13	5.4e-05	53.8
Lipid Metabolism	LDL	rs537919294	2	20904624	A	C	-0.134	0.022	1.8e-09	3.3e-05	36.2
Lipid Metabolism	LDL	rs573290684	2	20915168	C	A	-0.156	0.028	2.3e-08	4.6e-05	31.2
Lipid Metabolism	LDL	rs191742577	2	20974074	A	G	-0.154	0.023	1.9e-11	1.1e-04	45.1
Lipid Metabolism	LDL	rs185930747	2	20997604	T	A	-0.090	0.009	1.2e-21	7.5e-05	91.4
Lipid Metabolism	LDL	rs181651026	2	21055686	T	C	-0.098	0.014	1.0e-12	4.3e-05	50.8
Lipid Metabolism	LDL	rs182926316	2	21067006	A	G	-0.088	0.013	1.5e-11	4.7e-05	45.5
Lipid Metabolism	LDL	rs181472012	2	21096406	T	C	-0.118	0.021	3.6e-08	2.9e-05	30.4
Lipid Metabolism	LDL	rs547264415	2	21108212	G	A	0.117	0.015	2.7e-14	5.6e-05	58.0
Lipid Metabolism	LDL	rs74828123	2	21113461	T	A	-0.211	0.022	2.4e-21	1.2e-04	90.0
Lipid Metabolism	LDL	rs536643142	2	21181524	A	G	-0.179	0.013	5.2e-43	1.6e-04	189.0
Lipid Metabolism	LDL	rs141870095	2	21204244	A	G	-0.130	0.012	3.2e-28	1.0e-04	121.4
Lipid Metabolism	LDL	rs62122481	2	21216815	A	C	0.071	0.001	1.0e-200	1.9e-03	2347.9
Lipid Metabolism	LDL	rs72654427	2	21224614	G	A	-0.140	0.021	2.4e-11	4.4e-05	44.6
Lipid Metabolism	LDL	rs72654423	2	21225354	C	T	0.139	0.008	8.5e-74	2.7e-04	330.2
Lipid Metabolism	LDL	rs531608823	2	21226413	G	A	0.127	0.018	3.7e-13	5.8e-05	52.8
Lipid Metabolism	LDL	rs1801698	2	21227503	C	T	-0.099	0.012	4.5e-16	6.1e-05	66.0
Lipid Metabolism	LDL	rs12713559	2	21229068	A	G	0.315	0.029	8.5e-27	1.3e-04	114.8
Lipid Metabolism	LDL	rs1042023	2	21229446	C	G	0.136	0.007	3.6e-90	3.3e-04	405.5
Lipid Metabolism	LDL	rs1801696	2	21232044	T	C	0.071	0.011	3.2e-10	3.3e-05	39.5
Lipid Metabolism	LDL	rs553103896	2	21242330	A	G	0.210	0.037	1.5e-08	8.1e-05	32.0
Lipid Metabolism	LDL	rs533794587	2	21247364	A	G	0.106	0.016	5.8e-11	4.1e-05	42.9

Phenotype	Trait	SNP	Chr	Position (GRCh37/hg19)	EA	NEA	BETA	SE	P-value	R2	F
Lipid Metabolism	LDL	rs72653053	2	21266223	C	T	-0.080	0.007	6.4e-32	1.1e-04	138.2
Lipid Metabolism	LDL	rs186030351	2	21271840	T	C	-0.106	0.013	8.5e-17	5.8e-05	69.3
Lipid Metabolism	LDL	rs57667647	2	21292394	A	G	-0.094	0.004	1.7e-150	5.7e-04	682.8
Lipid Metabolism	LDL	rs183281507	2	21298013	A	G	-0.154	0.010	7.4e-59	2.1e-04	261.7
Lipid Metabolism	LDL	rs188462600	2	21307980	T	C	0.127	0.023	2.1e-08	4.1e-05	31.4
Lipid Metabolism	LDL	rs549173888	2	21308173	G	A	0.140	0.019	1.2e-13	1.4e-04	54.9
Lipid Metabolism	LDL	rs563927682	2	21308261	T	C	0.082	0.013	7.8e-11	3.9e-05	42.3
Lipid Metabolism	LDL	rs189339404	2	21326834	C	A	-0.191	0.025	4.0e-14	7.7e-05	57.2
Lipid Metabolism	LDL	rs141437972	2	21336831	T	C	-0.131	0.012	6.2e-27	1.4e-04	115.5
Lipid Metabolism	LDL	rs567270299	2	21346488	G	A	0.153	0.023	1.4e-11	4.0e-05	45.7
Lipid Metabolism	LDL	rs72782175	2	21366682	C	T	-0.096	0.006	2.8e-56	2.0e-04	249.8
Lipid Metabolism	LDL	rs143497571	2	21380700	T	C	-0.089	0.013	1.4e-11	3.8e-05	45.7
Lipid Metabolism	LDL	rs537990504	2	21383374	C	G	0.131	0.020	1.8e-11	5.7e-05	45.2
Lipid Metabolism	LDL	rs549421994	2	21391233	C	G	-0.303	0.042	3.6e-13	9.0e-05	52.9
Lipid Metabolism	LDL	rs553806006	2	21400604	G	A	-0.107	0.018	2.5e-09	3.1e-05	35.6
Lipid Metabolism	LDL	rs191341651	2	21404976	A	C	0.137	0.013	8.3e-27	9.5e-05	114.9
Lipid Metabolism	LDL	rs184675979	2	21405099	A	G	0.105	0.011	2.2e-22	1.1e-04	94.7
Lipid Metabolism	LDL	rs143013272	2	21408185	T	C	-0.111	0.012	2.0e-21	7.4e-05	90.3
Lipid Metabolism	LDL	rs144552532	2	21460714	T	C	-0.098	0.012	2.8e-17	6.0e-05	71.5
Lipid Metabolism	LDL	rs149535160	2	21470474	A	G	-0.146	0.015	1.7e-21	7.7e-05	90.6
Lipid Metabolism	LDL	rs72789623	2	21472913	C	A	0.049	0.004	2.2e-38	1.4e-04	167.8
Lipid Metabolism	LDL	rs559672768	2	21513823	T	C	-0.146	0.025	6.6e-09	4.4e-05	33.7
Lipid Metabolism	LDL	rs13014768	2	21514796	G	C	-0.090	0.003	1.0e-200	1.0e-03	1280.7
Lipid Metabolism	LDL	rs191450029	2	21942500	C	G	0.799	0.074	6.5e-27	2.2e-04	115.4
Lipid Metabolism	LDL	rs115295106	2	22063652	A	C	-0.041	0.007	1.8e-08	2.6e-05	31.7
Lipid Metabolism	LDL	rs552748859	2	22097445	C	A	-0.180	0.025	5.0e-13	6.2e-05	52.2
Lipid Metabolism	LDL	rs181370243	2	22245786	A	G	0.402	0.054	1.4e-13	7.0e-05	54.7
Lipid Metabolism	LDL	rs139099166	2	22250397	T	C	0.270	0.048	2.1e-08	7.9e-05	31.4
Lipid Metabolism	LDL	rs541138574	2	22812173	T	C	0.260	0.033	3.0e-15	8.1e-05	62.3
Lipid Metabolism	LDL	rs565710874	2	22850972	A	G	0.298	0.042	2.0e-12	7.6e-05	49.4
Lipid Metabolism	LDL	rs150520162	2	23541654	C	T	-0.860	0.123	3.2e-12	7.6e-05	48.6
Lipid Metabolism	LDL	rs2543662	2	24443271	A	C	-0.009	0.002	1.8e-08	2.6e-05	31.7
Lipid Metabolism	LDL	rs146556681	2	25830370	T	C	-0.046	0.008	2.9e-08	2.5e-05	30.7
Lipid Metabolism	LDL	rs142787485	2	26358156	G	A	-0.029	0.004	7.0e-13	4.2e-05	51.5
Lipid Metabolism	LDL	rs13394970	2	26929282	G	T	0.010	0.001	2.5e-11	3.7e-05	44.6
Lipid Metabolism	LDL	rs4665972	2	27598097	C	T	-0.030	0.001	2.7e-94	3.5e-04	424.4
Lipid Metabolism	LDL	rs191419430	2	27656038	A	G	-0.058	0.010	4.5e-09	2.8e-05	34.4
Lipid Metabolism	LDL	rs74429612	2	27993843	T	C	-0.016	0.002	2.7e-20	6.9e-05	85.2
Lipid Metabolism	LDL	rs71441185	2	42404073	G	A	0.025	0.004	6.5e-10	3.1e-05	38.2
Lipid Metabolism	LDL	rs2009563	2	43242358	C	T	0.012	0.002	3.0e-09	2.9e-05	35.2
Lipid Metabolism	LDL	rs113191497	2	43439585	A	G	-0.029	0.005	1.7e-09	3.0e-05	36.3
Lipid Metabolism	LDL	rs137932185	2	43545301	T	C	0.025	0.004	1.4e-11	3.7e-05	45.6
Lipid Metabolism	LDL	rs76922218	2	43786585	T	C	0.029	0.004	2.9e-16	5.4e-05	66.9
Lipid Metabolism	LDL	rs77293106	2	43843950	C	G	0.020	0.003	1.1e-15	5.2e-05	64.2
Lipid Metabolism	LDL	rs181664929	2	43919041	A	G	-0.101	0.012	4.2e-17	5.8e-05	70.7
Lipid Metabolism	LDL	rs192169402	2	43933171	G	C	0.078	0.009	6.9e-19	6.5e-05	78.8
Lipid Metabolism	LDL	rs13414390	2	43942171	T	C	0.042	0.008	2.6e-08	2.5e-05	31.0
Lipid Metabolism	LDL	rs149484430	2	44031257	G	A	0.051	0.009	4.0e-09	2.8e-05	34.6
Lipid Metabolism	LDL	rs150401285	2	44040341	G	A	0.200	0.024	2.1e-17	6.7e-05	72.1
Lipid Metabolism	LDL	rs72796720	2	44053599	A	G	0.164	0.022	6.6e-14	5.5e-05	56.2
Lipid Metabolism	LDL	rs111617668	2	44055922	T	C	-0.114	0.003	1.0e-200	1.2e-03	1537.9
Lipid Metabolism	LDL	rs531730588	2	44058373	A	G	0.105	0.019	4.4e-08	2.9e-05	30.0
Lipid Metabolism	LDL	rs145164937	2	44059195	C	G	0.134	0.023	9.2e-09	3.2e-05	33.0
Lipid Metabolism	LDL	rs4148218	2	44099582	A	G	-0.039	0.002	6.3e-106	3.9e-04	477.8
Lipid Metabolism	LDL	rs141953582	2	44165037	C	T	0.069	0.012	1.7e-08	2.6e-05	31.8
Lipid Metabolism	LDL	rs79351940	2	44179642	C	T	-0.067	0.011	1.5e-09	3.1e-05	36.6

Phenotype	Trait	SNP	Chr	Position (GRCh37/hg19)	EA	NEA	BETA	SE	P-value	R2	F
Lipid Metabolism	LDL	rs75120545	2	44271496	T	C	-0.024	0.004	2.3e-08	2.5e-05	31.2
Lipid Metabolism	LDL	rs13023530	2	44322668	A	G	0.019	0.003	1.2e-13	4.5e-05	55.0
Lipid Metabolism	LDL	rs786416	2	44693146	G	T	-0.020	0.002	9.3e-16	5.2e-05	64.6
Lipid Metabolism	LDL	rs74262166	2	44902771	G	A	-0.045	0.007	2.0e-09	3.0e-05	35.9
Lipid Metabolism	LDL	rs12712955	2	46166321	G	A	-0.011	0.001	2.0e-16	5.5e-05	67.6
Lipid Metabolism	LDL	rs34606253	2	61582738	C	A	-0.011	0.002	9.9e-09	2.7e-05	32.9
Lipid Metabolism	LDL	rs4671050	2	62988169	T	G	-0.022	0.002	3.5e-50	1.8e-04	221.9
Lipid Metabolism	LDL	rs13036222	2	64046736	G	A	-0.022	0.004	3.2e-09	2.8e-05	35.0
Lipid Metabolism	LDL	rs72808249	2	64172502	T	C	-0.022	0.003	1.6e-12	4.1e-05	50.0
Lipid Metabolism	LDL	rs4671611	2	64967175	A	G	0.011	0.001	1.9e-15	5.2e-05	63.2
Lipid Metabolism	LDL	rs10186240	2	65577651	G	T	0.008	0.001	8.3e-09	2.7e-05	33.2
Lipid Metabolism	LDL	rs2860450	2	70545888	T	G	0.011	0.001	9.9e-14	4.5e-05	55.4
Lipid Metabolism	LDL	rs11887442	2	85879037	A	G	-0.012	0.002	1.9e-12	4.6e-05	49.6
Lipid Metabolism	LDL	rs2970901	2	88428834	T	G	0.010	0.001	6.2e-12	3.8e-05	47.3
Lipid Metabolism	LDL	rs10185855	2	101642260	G	A	-0.013	0.001	1.5e-19	6.6e-05	81.9
Lipid Metabolism	LDL	rs1992172	2	109093836	G	A	0.020	0.002	1.4e-28	1.0e-04	122.9
Lipid Metabolism	LDL	rs6734238	2	113841030	G	A	-0.009	0.001	3.6e-11	3.6e-05	43.8
Lipid Metabolism	LDL	rs116704418	2	117806119	A	G	-0.021	0.003	2.3e-11	3.6e-05	44.7
Lipid Metabolism	LDL	rs140198034	2	118630649	T	G	0.033	0.003	2.4e-28	9.9e-05	121.9
Lipid Metabolism	LDL	rs141213470	2	118788583	T	A	0.045	0.006	2.1e-14	4.7e-05	58.4
Lipid Metabolism	LDL	rs55938402	2	118843158	A	G	-0.042	0.002	1.5e-70	2.6e-04	315.3
Lipid Metabolism	LDL	rs17050272	2	121306440	A	G	-0.023	0.001	4.2e-56	2.1e-04	249.0
Lipid Metabolism	LDL	rs2460382	2	135014116	A	G	0.011	0.002	9.4e-11	3.4e-05	41.9
Lipid Metabolism	LDL	rs4954195	2	135656503	G	C	0.019	0.003	2.9e-08	2.6e-05	30.8
Lipid Metabolism	LDL	rs1375131	2	135954797	C	T	0.020	0.002	8.0e-30	1.2e-04	128.7
Lipid Metabolism	LDL	rs7602937	2	144145842	C	A	0.010	0.002	2.7e-09	2.9e-05	35.4
Lipid Metabolism	LDL	rs12614487	2	158434569	T	C	-0.027	0.003	5.7e-24	8.3e-05	102.0
Lipid Metabolism	LDL	rs10184004	2	165508389	T	C	-0.010	0.001	1.0e-13	4.5e-05	55.3
Lipid Metabolism	LDL	rs10184673	2	169827796	A	G	-0.022	0.001	6.6e-54	1.9e-04	239.0
Lipid Metabolism	LDL	rs12693968	2	203302627	A	G	0.021	0.002	2.0e-40	1.4e-04	177.1
Lipid Metabolism	LDL	rs934287	2	203708307	G	A	0.022	0.002	6.5e-34	1.2e-04	147.4
Lipid Metabolism	LDL	rs1250259	2	216300482	A	T	0.018	0.002	1.7e-28	1.0e-04	122.6
Lipid Metabolism	LDL	rs34353396	2	219153982	C	T	0.009	0.002	2.2e-09	3.4e-05	35.8
Lipid Metabolism	LDL	rs78058190	2	219699999	A	G	0.024	0.004	3.2e-11	3.7e-05	44.1
Lipid Metabolism	LDL	rs116001967	2	228451050	A	G	-0.037	0.006	1.0e-08	2.7e-05	32.7
Lipid Metabolism	LDL	rs11675168	2	234664679	A	G	-0.023	0.004	2.7e-08	2.5e-05	30.9
Lipid Metabolism	LDL	rs6431630	2	234677386	A	G	0.024	0.002	1.2e-26	9.3e-05	114.2
Lipid Metabolism	LDL	rs9873301	3	12065264	T	G	-0.033	0.004	2.5e-16	5.5e-05	67.2
Lipid Metabolism	LDL	rs564271093	3	12344726	T	C	-0.145	0.021	1.8e-12	5.6e-05	49.7
Lipid Metabolism	LDL	rs709147	3	12448940	G	A	-0.021	0.001	2.2e-49	1.8e-04	218.3
Lipid Metabolism	LDL	rs9852128	3	12635623	G	A	-0.021	0.002	1.7e-36	1.3e-04	159.2
Lipid Metabolism	LDL	rs6792725	3	24520283	G	A	-0.015	0.002	1.2e-22	7.9e-05	95.9
Lipid Metabolism	LDL	rs9837622	3	32514647	A	T	-0.037	0.003	9.0e-43	1.5e-04	187.9
Lipid Metabolism	LDL	rs11711421	3	52561779	T	C	0.011	0.001	4.8e-15	5.0e-05	61.3
Lipid Metabolism	LDL	rs144767829	3	52828293	A	G	0.055	0.010	9.0e-09	2.7e-05	33.1
Lipid Metabolism	LDL	rs71311871	3	58420613	G	A	-0.038	0.003	3.0e-51	1.8e-04	226.8
Lipid Metabolism	LDL	rs9852465	3	58465183	G	A	-0.010	0.001	6.2e-12	3.9e-05	47.3
Lipid Metabolism	LDL	rs541035252	3	66608509	A	T	0.202	0.036	2.1e-08	7.7e-05	31.4
Lipid Metabolism	LDL	rs55921103	3	69810294	T	G	0.013	0.001	1.4e-17	6.0e-05	72.8
Lipid Metabolism	LDL	rs7637250	3	100445155	C	G	-0.009	0.002	2.7e-09	2.9e-05	35.4
Lipid Metabolism	LDL	rs3732359	3	119536429	A	G	-0.015	0.002	1.6e-19	6.6e-05	81.6
Lipid Metabolism	LDL	rs3817040	3	122254769	A	G	0.019	0.002	8.8e-23	8.0e-05	96.5
Lipid Metabolism	LDL	rs9828317	3	122563600	G	T	0.008	0.001	3.8e-08	2.5e-05	30.2
Lipid Metabolism	LDL	rs2011442	3	124959834	T	C	0.012	0.001	3.8e-16	5.4e-05	66.3
Lipid Metabolism	LDL	rs7641302	3	126060315	C	T	0.013	0.002	2.1e-15	5.1e-05	63.0
Lipid Metabolism	LDL	rs12489937	3	127281914	A	G	0.014	0.002	4.0e-10	3.2e-05	39.1

Phenotype	Trait	SNP	Chr	Position (GRCh37/hg19)	EA	NEA	BETA	SE	P-value	R2	F
Lipid Metabolism	LDL	rs56299595	3	129278182	G	A	0.018	0.002	6.9e-18	6.1e-05	74.2
Lipid Metabolism	LDL	rs74341202	3	132183991	A	G	-0.043	0.003	1.0e-39	1.4e-04	174.0
Lipid Metabolism	LDL	rs74735511	3	132269004	G	A	-0.031	0.002	2.4e-36	1.3e-04	158.5
Lipid Metabolism	LDL	rs576771	3	135989292	T	C	0.013	0.002	1.1e-15	5.3e-05	64.3
Lipid Metabolism	LDL	rs9653945	3	142660706	A	G	-0.013	0.001	4.8e-19	6.5e-05	79.5
Lipid Metabolism	LDL	rs407258	3	155560978	C	T	0.010	0.002	1.2e-10	3.4e-05	41.5
Lipid Metabolism	LDL	rs6441313	3	160036400	G	A	-0.013	0.001	7.4e-19	6.4e-05	78.6
Lipid Metabolism	LDL	rs56118251	3	171534525	G	A	0.014	0.002	4.4e-13	4.3e-05	52.5
Lipid Metabolism	LDL	rs78248633	3	178867338	C	T	-0.042	0.008	4.0e-08	2.5e-05	30.2
Lipid Metabolism	LDL	rs16861497	3	186731327	T	G	-0.009	0.001	2.4e-09	2.9e-05	35.6
Lipid Metabolism	LDL	rs59950280	4	3452345	A	G	0.018	0.002	9.8e-34	1.2e-04	146.5
Lipid Metabolism	LDL	rs731450	4	3490328	T	C	0.010	0.001	4.8e-12	3.9e-05	47.8
Lipid Metabolism	LDL	rs4689653	4	7223319	G	T	0.011	0.001	5.8e-15	5.0e-05	61.0
Lipid Metabolism	LDL	rs9291634	4	15021185	C	T	0.008	0.001	4.5e-09	2.8e-05	34.4
Lipid Metabolism	LDL	rs874040	4	26108197	C	G	0.009	0.002	1.3e-08	2.6e-05	32.4
Lipid Metabolism	LDL	rs79260345	4	26298288	A	C	0.017	0.003	3.2e-08	2.5e-05	30.6
Lipid Metabolism	LDL	rs10025454	4	39486969	A	T	-0.009	0.001	5.0e-10	3.1e-05	38.7
Lipid Metabolism	LDL	rs79623641	4	39999404	A	G	-0.018	0.003	2.2e-10	3.4e-05	40.3
Lipid Metabolism	LDL	rs278940	4	40420752	G	A	0.009	0.001	3.3e-10	3.2e-05	39.5
Lipid Metabolism	LDL	rs11736427	4	48039902	T	A	-0.008	0.001	2.9e-08	2.5e-05	30.8
Lipid Metabolism	LDL	rs4864471	4	54426184	T	G	-0.014	0.002	1.4e-10	3.3e-05	41.2
Lipid Metabolism	LDL	rs1543179	4	57941700	T	C	-0.009	0.002	3.1e-08	2.5e-05	30.7
Lipid Metabolism	LDL	rs2060992	4	69328485	A	G	-0.018	0.002	2.5e-14	5.0e-05	58.1
Lipid Metabolism	LDL	rs34707604	4	69491456	C	T	0.032	0.002	8.8e-65	2.6e-04	288.9
Lipid Metabolism	LDL	rs293433	4	69590353	A	G	0.011	0.002	1.9e-10	3.3e-05	40.6
Lipid Metabolism	LDL	rs61361928	4	69962375	C	T	-0.088	0.011	9.8e-17	6.3e-05	69.0
Lipid Metabolism	LDL	rs531876114	4	71341209	A	G	0.119	0.019	2.8e-10	5.0e-05	39.8
Lipid Metabolism	LDL	rs143736900	4	72871285	C	T	0.067	0.011	7.3e-10	3.5e-05	37.9
Lipid Metabolism	LDL	rs190671241	4	73574142	G	A	0.299	0.019	9.1e-58	3.2e-04	256.7
Lipid Metabolism	LDL	rs187918276	4	74033564	C	G	0.467	0.022	8.6e-104	5.1e-04	468.0
Lipid Metabolism	LDL	rs72663045	4	74177397	G	T	0.040	0.005	1.2e-15	5.2e-05	64.0
Lipid Metabolism	LDL	rs148885848	4	74853287	A	G	0.324	0.021	2.9e-56	3.1e-04	249.8
Lipid Metabolism	LDL	rs4493564	4	77420407	G	A	-0.009	0.001	2.7e-10	3.2e-05	39.9
Lipid Metabolism	LDL	rs342467	4	88052219	C	T	0.010	0.001	8.3e-13	4.2e-05	51.2
Lipid Metabolism	LDL	rs55695526	4	100290620	G	C	0.017	0.002	3.2e-14	4.7e-05	57.6
Lipid Metabolism	LDL	rs1503778	4	100490688	A	G	-0.018	0.002	4.9e-28	9.8e-05	120.5
Lipid Metabolism	LDL	rs13135092	4	103198082	G	A	-0.021	0.003	1.6e-15	5.2e-05	63.5
Lipid Metabolism	LDL	rs17617028	4	106064683	A	G	0.013	0.002	2.5e-15	5.1e-05	62.6
Lipid Metabolism	LDL	rs2298992	4	110892163	C	T	-0.009	0.001	2.4e-10	3.3e-05	40.1
Lipid Metabolism	LDL	rs148046400	4	119487471	A	G	-0.016	0.003	1.7e-08	2.6e-05	31.8
Lipid Metabolism	LDL	rs138204164	4	120123417	G	C	-0.015	0.002	2.0e-12	4.0e-05	49.5
Lipid Metabolism	LDL	rs7692092	4	126257065	T	C	-0.010	0.002	9.2e-09	2.7e-05	33.0
Lipid Metabolism	LDL	rs2085724	4	151206200	C	A	0.010	0.001	2.1e-11	3.6e-05	44.9
Lipid Metabolism	LDL	rs116355217	4	151829580	C	T	0.067	0.011	4.8e-09	2.9e-05	34.3
Lipid Metabolism	LDL	rs72729610	4	154190965	G	A	-0.015	0.002	3.7e-16	5.4e-05	66.4
Lipid Metabolism	LDL	rs534659996	4	155308673	G	T	0.104	0.016	5.5e-11	3.8e-05	43.0
Lipid Metabolism	LDL	rs551659708	4	155424667	T	C	0.167	0.024	6.4e-12	6.2e-05	47.2
Lipid Metabolism	LDL	rs6054	4	155489608	T	C	0.125	0.011	1.9e-30	1.1e-04	131.5
Lipid Metabolism	LDL	rs793900	4	185253019	T	C	0.009	0.001	3.8e-10	3.2e-05	39.2
Lipid Metabolism	LDL	rs546347707	5	21261739	A	G	0.240	0.042	1.5e-08	4.9e-05	32.0
Lipid Metabolism	LDL	rs569181343	5	21600697	T	C	0.164	0.027	2.4e-09	5.6e-05	35.6
Lipid Metabolism	LDL	rs116734477	5	52095024	T	C	-0.051	0.004	2.5e-44	1.6e-04	195.1
Lipid Metabolism	LDL	rs4074793	5	52193125	G	A	0.021	0.003	3.1e-16	5.4e-05	66.7
Lipid Metabolism	LDL	rs79760705	5	53298716	T	G	0.014	0.002	1.0e-09	3.0e-05	37.3
Lipid Metabolism	LDL	rs11743303	5	55859952	G	A	0.016	0.002	3.3e-19	6.5e-05	80.2
Lipid Metabolism	LDL	rs3010277	5	72014400	G	A	-0.018	0.002	5.5e-27	9.4e-05	115.7

Phenotype	Trait	SNP	Chr	Position (GRCh37/hg19)	EA	NEA	BETA	SE	P-value	R2	F
Lipid Metabolism	LDL	rs266433	5	72171715	T	C	-0.017	0.002	1.7e-18	6.3e-05	77.0
Lipid Metabolism	LDL	rs145275181	5	74201340	G	T	0.041	0.007	1.7e-08	2.6e-05	31.8
Lipid Metabolism	LDL	rs73116844	5	74521249	T	C	0.038	0.006	3.1e-11	3.6e-05	44.1
Lipid Metabolism	LDL	rs11741997	5	74560542	G	A	-0.039	0.006	8.0e-12	4.3e-05	46.8
Lipid Metabolism	LDL	rs72633961	5	74564190	G	A	0.068	0.002	1.0e-200	1.3e-03	1633.7
Lipid Metabolism	LDL	rs536947347	5	74633133	A	G	-0.049	0.008	8.1e-09	2.7e-05	33.3
Lipid Metabolism	LDL	rs6892146	5	74971990	G	C	0.036	0.002	4.7e-79	2.9e-04	354.4
Lipid Metabolism	LDL	rs142710807	5	75035402	T	C	-0.034	0.005	8.9e-11	3.4e-05	42.0
Lipid Metabolism	LDL	rs148352135	5	75171112	G	A	-0.046	0.008	8.8e-10	3.1e-05	37.6
Lipid Metabolism	LDL	rs113691753	5	75276840	A	T	0.017	0.002	6.0e-13	4.2e-05	51.8
Lipid Metabolism	LDL	rs77075344	5	75313733	C	G	0.038	0.006	4.1e-09	2.8e-05	34.6
Lipid Metabolism	LDL	rs2617447	5	96267655	G	A	0.008	0.001	1.9e-09	2.9e-05	36.1
Lipid Metabolism	LDL	rs6896005	5	122834112	C	T	0.017	0.001	1.0e-32	1.2e-04	141.9
Lipid Metabolism	LDL	rs2057655	5	131807624	A	G	-0.019	0.002	1.2e-28	1.0e-04	123.3
Lipid Metabolism	LDL	rs6596284	5	135470544	C	T	0.008	0.002	4.6e-08	2.4e-05	29.9
Lipid Metabolism	LDL	rs13179861	5	139527721	A	G	-0.012	0.002	1.6e-11	3.7e-05	45.5
Lipid Metabolism	LDL	rs11167778	5	141888881	T	C	0.022	0.002	3.3e-22	7.6e-05	93.9
Lipid Metabolism	LDL	rs6886573	5	156265523	G	T	0.035	0.004	2.0e-18	6.2e-05	76.7
Lipid Metabolism	LDL	rs139294635	5	156360413	C	T	-0.062	0.010	1.3e-09	3.0e-05	36.8
Lipid Metabolism	LDL	rs11134475	5	156399950	G	A	0.035	0.001	4.2e-132	4.9e-04	598.1
Lipid Metabolism	LDL	rs193010652	5	156410046	A	C	0.156	0.019	7.4e-16	5.6e-05	65.0
Lipid Metabolism	LDL	rs186756395	5	156449311	C	T	0.155	0.021	4.1e-13	6.2e-05	52.6
Lipid Metabolism	LDL	rs12520950	5	156474469	A	G	-0.080	0.007	1.4e-33	1.2e-04	145.9
Lipid Metabolism	LDL	rs11134822	5	157001302	T	C	0.011	0.002	3.0e-08	2.5e-05	30.7
Lipid Metabolism	LDL	rs2112535	5	176531075	A	G	-0.010	0.002	1.9e-10	3.3e-05	40.5
Lipid Metabolism	LDL	rs272441	5	179301260	G	A	-0.014	0.002	2.2e-08	2.6e-05	31.3
Lipid Metabolism	LDL	rs730551	6	6971753	T	C	0.008	0.001	4.9e-09	2.8e-05	34.2
Lipid Metabolism	LDL	rs59408219	6	11850754	C	T	-0.022	0.003	2.1e-17	5.9e-05	72.1
Lipid Metabolism	LDL	rs6459440	6	15995565	G	A	-0.013	0.001	3.6e-20	6.9e-05	84.6
Lipid Metabolism	LDL	rs2235215	6	16131156	C	T	-0.032	0.001	1.5e-101	3.7e-04	457.7
Lipid Metabolism	LDL	rs74451139	6	16176210	G	C	-0.020	0.003	2.1e-10	3.3e-05	40.3
Lipid Metabolism	LDL	rs9477876	6	18769236	G	A	-0.009	0.001	2.8e-09	3.0e-05	35.3
Lipid Metabolism	LDL	rs6927936	6	21379611	C	T	0.009	0.001	2.2e-10	3.3e-05	40.3
Lipid Metabolism	LDL	rs113977268	6	22359515	A	G	-0.017	0.003	8.8e-11	3.4e-05	42.1
Lipid Metabolism	LDL	rs111663960	6	26082220	G	A	-0.014	0.002	1.1e-11	3.8e-05	46.2
Lipid Metabolism	LDL	rs1800562	6	26093141	A	G	-0.057	0.003	3.0e-95	3.5e-04	428.7
Lipid Metabolism	LDL	rs62396175	6	26648517	T	G	0.032	0.006	3.6e-08	2.5e-05	30.4
Lipid Metabolism	LDL	rs71559014	6	27122444	G	A	-0.032	0.003	2.1e-32	1.1e-04	140.5
Lipid Metabolism	LDL	rs62401424	6	27966203	C	T	0.047	0.007	4.5e-11	3.8e-05	43.4
Lipid Metabolism	LDL	rs35814746	6	28299088	C	T	-0.027	0.003	8.5e-25	8.6e-05	105.7
Lipid Metabolism	LDL	rs2517671	6	29937977	G	A	0.015	0.001	4.3e-24	9.0e-05	102.5
Lipid Metabolism	LDL	rs9394034	6	31041958	T	C	0.018	0.002	1.6e-21	7.4e-05	90.8
Lipid Metabolism	LDL	rs9469004	6	31407896	C	T	0.023	0.002	4.2e-32	1.2e-04	139.1
Lipid Metabolism	LDL	rs2230365	6	31525448	T	C	0.016	0.002	1.8e-16	5.8e-05	67.8
Lipid Metabolism	LDL	rs113176001	6	32571483	C	G	0.023	0.002	8.9e-26	1.3e-04	110.2
Lipid Metabolism	LDL	rs6689	6	32627700	G	A	0.039	0.002	6.0e-94	3.5e-04	422.8
Lipid Metabolism	LDL	rs116315708	6	32683744	A	T	0.048	0.007	1.4e-11	4.0e-05	45.7
Lipid Metabolism	LDL	rs3800461	6	34616322	C	G	-0.024	0.002	3.0e-30	1.1e-04	130.6
Lipid Metabolism	LDL	rs7739991	6	34867606	A	G	0.012	0.002	1.7e-09	3.0e-05	36.3
Lipid Metabolism	LDL	rs143018638	6	35679692	T	C	-0.041	0.007	1.4e-09	3.0e-05	36.7
Lipid Metabolism	LDL	rs9394401	6	37038033	C	T	-0.011	0.001	1.8e-15	5.1e-05	63.3
Lipid Metabolism	LDL	rs11961538	6	39231013	C	T	0.010	0.002	9.3e-11	3.4e-05	42.0
Lipid Metabolism	LDL	rs2296805	6	42928758	G	T	-0.013	0.001	1.1e-21	7.4e-05	91.6
Lipid Metabolism	LDL	rs68137036	6	43820215	G	A	-0.009	0.002	7.4e-10	3.1e-05	37.9
Lipid Metabolism	LDL	rs73429745	6	44101422	T	C	0.011	0.002	9.5e-10	3.0e-05	37.4
Lipid Metabolism	LDL	rs55959984	6	52433894	G	A	-0.016	0.002	5.0e-24	8.3e-05	102.2

Phenotype	Trait	SNP	Chr	Position (GRCh37/hg19)	EA	NEA	BETA	SE	P-value	R2	F
Lipid Metabolism	LDL	rs17665563	6	52458872	T	C	0.012	0.001	6.0e-17	5.7e-05	70.0
Lipid Metabolism	LDL	rs12662589	6	53509035	C	G	0.015	0.002	4.5e-22	7.6e-05	93.3
Lipid Metabolism	LDL	rs9496567	6	100602753	A	G	-0.021	0.002	2.7e-37	1.3e-04	162.8
Lipid Metabolism	LDL	rs62419249	6	101447338	A	G	0.012	0.001	6.6e-18	6.1e-05	74.3
Lipid Metabolism	LDL	rs11968490	6	106381668	T	C	0.010	0.001	9.1e-13	4.2e-05	51.0
Lipid Metabolism	LDL	rs11153143	6	109300661	T	C	-0.014	0.002	1.2e-11	3.8e-05	45.9
Lipid Metabolism	LDL	rs3798227	6	116324898	G	A	-0.017	0.001	4.2e-33	1.2e-04	143.6
Lipid Metabolism	LDL	rs9388498	6	126873423	T	G	-0.015	0.002	1.6e-15	5.2e-05	63.5
Lipid Metabolism	LDL	rs577721086	6	127440047	C	T	0.023	0.003	3.0e-13	4.3e-05	53.2
Lipid Metabolism	LDL	rs3890746	6	130371055	T	C	0.010	0.001	2.1e-12	4.0e-05	49.4
Lipid Metabolism	LDL	rs9399137	6	135419018	C	T	-0.026	0.002	1.3e-58	2.1e-04	260.5
Lipid Metabolism	LDL	rs111613573	6	139231576	T	C	-0.036	0.005	9.0e-13	4.1e-05	51.1
Lipid Metabolism	LDL	rs62441843	6	139331253	G	A	0.021	0.002	5.1e-30	1.1e-04	129.5
Lipid Metabolism	LDL	rs9480534	6	151015562	G	A	-0.017	0.002	2.8e-12	4.0e-05	48.8
Lipid Metabolism	LDL	rs4870040	6	151872090	C	T	0.017	0.002	2.2e-14	4.7e-05	58.3
Lipid Metabolism	LDL	rs17710008	6	153043035	A	G	0.011	0.002	8.2e-09	2.7e-05	33.2
Lipid Metabolism	LDL	rs140990847	6	160339987	A	G	-0.093	0.017	3.3e-08	3.0e-05	30.5
Lipid Metabolism	LDL	rs3798205	6	160398063	A	G	0.032	0.004	4.3e-19	6.5e-05	79.7
Lipid Metabolism	LDL	rs73021693	6	160439725	A	G	0.036	0.004	7.8e-21	7.1e-05	87.6
Lipid Metabolism	LDL	rs78423775	6	160519228	A	G	-0.033	0.003	1.4e-31	1.1e-04	136.7
Lipid Metabolism	LDL	rs12208357	6	160543148	T	C	0.064	0.003	3.3e-126	4.6e-04	571.0
Lipid Metabolism	LDL	rs146785976	6	160545953	G	A	-0.032	0.005	2.5e-10	3.3e-05	40.0
Lipid Metabolism	LDL	rs117301306	6	160561308	T	C	-0.039	0.007	2.8e-09	2.9e-05	35.3
Lipid Metabolism	LDL	rs118053827	6	160580496	T	C	-0.038	0.005	1.9e-15	5.2e-05	63.2
Lipid Metabolism	LDL	rs562632887	6	160654044	T	C	0.297	0.039	1.1e-14	1.5e-04	59.7
Lipid Metabolism	LDL	rs144833821	6	160813782	C	T	-0.050	0.008	5.7e-11	3.5e-05	42.9
Lipid Metabolism	LDL	rs189821701	6	160862809	T	C	0.081	0.011	1.7e-14	5.0e-05	58.9
Lipid Metabolism	LDL	rs549975194	6	160863985	T	C	0.163	0.010	5.5e-63	2.3e-04	280.6
Lipid Metabolism	LDL	rs12214416	6	160910517	A	T	-0.027	0.003	1.1e-15	5.2e-05	64.2
Lipid Metabolism	LDL	rs147555597	6	160911596	A	G	0.140	0.008	6.9e-73	2.7e-04	326.1
Lipid Metabolism	LDL	rs529471813	6	160959137	A	G	0.247	0.041	1.5e-09	6.5e-05	36.6
Lipid Metabolism	LDL	rs558059457	6	160960190	G	A	0.198	0.032	3.5e-10	9.5e-05	39.4
Lipid Metabolism	LDL	rs41265924	6	160965618	G	A	-0.039	0.004	3.8e-28	9.8e-05	121.0
Lipid Metabolism	LDL	rs76000021	6	160985501	C	T	-0.040	0.006	1.1e-10	3.4e-05	41.7
Lipid Metabolism	LDL	rs118039278	6	160985526	A	G	0.114	0.003	1.0e-200	1.4e-03	1680.2
Lipid Metabolism	LDL	rs140570886	6	161013013	C	T	0.208	0.006	1.0e-200	9.6e-04	1174.8
Lipid Metabolism	LDL	rs142231215	6	161015985	A	G	0.070	0.012	2.8e-09	2.9e-05	35.3
Lipid Metabolism	LDL	rs59948046	6	161119211	T	C	0.734	0.066	6.5e-29	3.2e-04	124.5
Lipid Metabolism	LDL	rs557242113	6	161175245	A	G	0.165	0.026	2.8e-10	5.2e-05	39.8
Lipid Metabolism	LDL	rs185990325	6	161200366	G	A	0.097	0.012	1.5e-16	8.2e-05	68.2
Lipid Metabolism	LDL	rs532754706	6	161329915	G	A	0.065	0.008	3.8e-16	7.3e-05	66.3
Lipid Metabolism	LDL	rs138797047	6	161334303	T	A	0.320	0.042	4.0e-14	1.5e-04	57.2
Lipid Metabolism	LDL	rs150908701	6	161421886	C	T	0.175	0.017	1.1e-23	9.8e-05	100.6
Lipid Metabolism	LDL	rs551938102	6	161430830	G	A	0.167	0.022	9.1e-15	6.8e-05	60.1
Lipid Metabolism	LDL	rs55660955	6	161658128	A	G	0.035	0.004	2.8e-20	6.9e-05	85.1
Lipid Metabolism	LDL	rs73672461	7	943950	A	G	0.019	0.003	2.2e-09	2.9e-05	35.8
Lipid Metabolism	LDL	rs10272002	7	1047615	G	A	-0.022	0.002	1.0e-38	1.4e-04	169.4
Lipid Metabolism	LDL	rs77523736	7	2071957	A	G	0.132	0.017	3.0e-15	7.9e-05	62.3
Lipid Metabolism	LDL	rs532639357	7	2393346	C	A	0.147	0.020	1.1e-13	6.2e-05	55.2
Lipid Metabolism	LDL	rs7811417	7	21534152	C	T	0.020	0.001	3.4e-44	1.6e-04	194.4
Lipid Metabolism	LDL	rs55649657	7	21607283	G	C	0.038	0.002	1.2e-110	4.1e-04	499.6
Lipid Metabolism	LDL	rs78741986	7	25991232	C	T	0.023	0.002	1.2e-50	1.8e-04	223.9
Lipid Metabolism	LDL	rs12700705	7	26160458	G	A	-0.012	0.002	4.5e-13	4.3e-05	52.4
Lipid Metabolism	LDL	rs4719925	7	28188995	G	A	0.013	0.002	1.7e-08	2.6e-05	31.8
Lipid Metabolism	LDL	rs12533280	7	36171953	T	C	0.018	0.002	1.2e-24	8.5e-05	105.0
Lipid Metabolism	LDL	rs73102255	7	36410738	T	A	-0.015	0.002	1.5e-12	4.1e-05	50.1

Phenotype	Trait	SNP	Chr	Position (GRCh37/hg19)	EA	NEA	BETA	SE	P-value	R2	F
Lipid Metabolism	LDL	rs7776825	7	41753331	G	A	0.011	0.002	1.2e-11	3.8e-05	46.0
Lipid Metabolism	LDL	rs7792961	7	44569399	C	G	0.034	0.002	2.6e-75	2.8e-04	337.2
Lipid Metabolism	LDL	rs139659653	7	44578747	A	G	-0.200	0.027	9.6e-14	7.4e-05	55.4
Lipid Metabolism	LDL	rs217385	7	44602187	T	G	-0.029	0.001	1.0e-93	3.4e-04	421.8
Lipid Metabolism	LDL	rs112866709	7	44780274	T	C	0.023	0.004	5.0e-10	3.2e-05	38.7
Lipid Metabolism	LDL	rs3873477	7	72715451	A	G	-0.012	0.002	3.4e-08	2.8e-05	30.5
Lipid Metabolism	LDL	rs113562761	7	73081694	G	C	0.026	0.004	4.4e-11	3.5e-05	43.4
Lipid Metabolism	LDL	rs2302434	7	75630183	T	C	0.015	0.002	3.3e-15	5.0e-05	62.1
Lipid Metabolism	LDL	rs56223611	7	87083157	A	C	-0.015	0.002	6.3e-14	4.6e-05	56.3
Lipid Metabolism	LDL	rs3779196	7	97990106	C	T	0.018	0.002	1.0e-21	7.5e-05	91.7
Lipid Metabolism	LDL	rs221786	7	100266081	C	T	-0.027	0.002	5.9e-33	1.2e-04	143.0
Lipid Metabolism	LDL	rs4729624	7	100529261	A	G	0.008	0.001	1.6e-08	2.6e-05	32.0
Lipid Metabolism	LDL	rs803073	7	101928279	A	G	-0.008	0.001	1.7e-09	3.0e-05	36.3
Lipid Metabolism	LDL	rs10264718	7	104638501	C	A	-0.012	0.002	2.8e-08	2.6e-05	30.9
Lipid Metabolism	LDL	rs10248717	7	107056795	G	A	0.013	0.002	2.3e-16	5.5e-05	67.4
Lipid Metabolism	LDL	rs62621812	7	127015083	A	G	0.030	0.005	4.7e-11	3.5e-05	43.3
Lipid Metabolism	LDL	rs1838931	7	130455588	T	C	0.011	0.002	1.1e-13	4.5e-05	55.2
Lipid Metabolism	LDL	rs12707169	7	134384117	C	T	-0.008	0.001	3.2e-08	2.5e-05	30.6
Lipid Metabolism	LDL	rs34372369	7	143092269	A	G	0.019	0.003	5.4e-10	3.1e-05	38.5
Lipid Metabolism	LDL	rs4374942	7	155026807	C	T	0.020	0.003	5.5e-15	5.0e-05	61.1
Lipid Metabolism	LDL	rs11761517	7	158532706	T	C	-0.008	0.001	1.2e-08	2.7e-05	32.5
Lipid Metabolism	LDL	rs2928630	8	6587067	C	T	0.011	0.001	2.7e-14	4.7e-05	58.0
Lipid Metabolism	LDL	rs2169387	8	9181395	G	A	0.058	0.002	2.5e-134	5.0e-04	608.4
Lipid Metabolism	LDL	rs28396619	8	9197007	G	A	0.018	0.003	1.1e-12	4.1e-05	50.7
Lipid Metabolism	LDL	rs17729883	8	9256631	C	T	-0.015	0.002	1.4e-23	8.1e-05	100.1
Lipid Metabolism	LDL	rs7005902	8	9543169	A	G	0.023	0.004	2.6e-09	2.9e-05	35.4
Lipid Metabolism	LDL	rs149715208	8	9845336	G	C	-0.065	0.012	4.9e-08	2.5e-05	29.8
Lipid Metabolism	LDL	rs4321967	8	10641407	T	C	-0.010	0.001	3.9e-12	4.0e-05	48.2
Lipid Metabolism	LDL	rs7459983	8	11807221	C	T	0.010	0.001	1.1e-12	4.1e-05	50.7
Lipid Metabolism	LDL	rs7844866	8	18196091	G	A	0.018	0.003	2.3e-10	3.3e-05	40.2
Lipid Metabolism	LDL	rs1495741	8	18272881	A	G	-0.022	0.002	4.1e-40	1.4e-04	175.7
Lipid Metabolism	LDL	rs900776	8	21918089	C	A	-0.020	0.002	1.8e-25	8.9e-05	108.8
Lipid Metabolism	LDL	rs543488205	8	22911067	A	G	-0.424	0.078	4.9e-08	5.7e-05	29.8
Lipid Metabolism	LDL	rs151150389	8	28897171	C	T	0.030	0.004	7.3e-15	4.9e-05	60.5
Lipid Metabolism	LDL	rs117139027	8	29024943	A	G	-0.078	0.006	3.6e-34	1.4e-04	148.5
Lipid Metabolism	LDL	rs576038680	8	29097172	G	T	-0.151	0.027	1.9e-08	4.3e-05	31.6
Lipid Metabolism	LDL	rs34265667	8	41542093	A	G	-0.024	0.004	1.1e-09	3.0e-05	37.2
Lipid Metabolism	LDL	rs9298506	8	55437524	G	A	0.025	0.002	6.2e-47	1.7e-04	207.0
Lipid Metabolism	LDL	rs9297994	8	59392324	A	G	-0.036	0.001	8.7e-130	4.8e-04	587.5
Lipid Metabolism	LDL	rs113847133	8	59542732	A	G	-0.035	0.003	2.5e-26	9.2e-05	112.7
Lipid Metabolism	LDL	rs144157766	8	59543665	C	T	0.162	0.028	1.2e-08	3.2e-05	32.4
Lipid Metabolism	LDL	rs6992869	8	61395832	C	T	0.010	0.001	9.7e-13	4.1e-05	50.9
Lipid Metabolism	LDL	rs62509311	8	74907295	T	A	-0.015	0.002	1.5e-22	7.8e-05	95.5
Lipid Metabolism	LDL	rs396760	8	81377803	A	G	0.010	0.002	1.4e-10	3.3e-05	41.1
Lipid Metabolism	LDL	rs800888	8	116464988	C	T	0.008	0.001	2.5e-08	2.6e-05	31.0
Lipid Metabolism	LDL	rs2737245	8	116658583	T	G	-0.025	0.002	4.0e-60	2.2e-04	267.5
Lipid Metabolism	LDL	rs577874819	8	125978106	T	C	-1.016	0.185	3.7e-08	7.6e-05	30.3
Lipid Metabolism	LDL	rs34447699	8	126461739	G	A	0.032	0.002	1.7e-79	2.9e-04	356.4
Lipid Metabolism	LDL	rs6982502	8	126479362	T	C	-0.055	0.001	1.0e-200	1.3e-03	1575.1
Lipid Metabolism	LDL	rs59310016	8	126514997	C	T	0.022	0.002	5.3e-29	1.2e-04	124.9
Lipid Metabolism	LDL	rs12542138	8	141833938	A	G	-0.008	0.001	3.4e-08	2.5e-05	30.5
Lipid Metabolism	LDL	rs11787335	8	145044104	T	C	0.023	0.001	5.3e-56	2.0e-04	248.6
Lipid Metabolism	LDL	rs3780181	9	2640759	G	A	-0.036	0.003	1.4e-38	1.4e-04	168.7
Lipid Metabolism	LDL	rs7043836	9	2651714	G	T	-0.025	0.004	5.3e-12	3.9e-05	47.6
Lipid Metabolism	LDL	rs4644350	9	16882916	G	C	-0.013	0.001	2.5e-20	7.0e-05	85.4
Lipid Metabolism	LDL	rs10964035	9	19218048	C	T	-0.015	0.002	3.8e-18	6.1e-05	75.4

Phenotype	Trait	SNP	Chr	Position (GRCh37/hg19)	EA	NEA	BETA	SE	P-value	R2	F
Lipid Metabolism	LDL	rs12554969	9	19261842	A	C	0.035	0.003	1.5e-39	1.4e-04	173.1
Lipid Metabolism	LDL	rs10757273	9	22090301	A	C	-0.014	0.002	1.3e-19	7.6e-05	82.2
Lipid Metabolism	LDL	rs79394247	9	25221128	C	A	-0.361	0.065	3.2e-08	7.1e-05	30.6
Lipid Metabolism	LDL	rs60988380	9	33117954	T	C	0.013	0.002	1.4e-08	2.6e-05	32.2
Lipid Metabolism	LDL	rs10869598	9	78214452	C	T	0.015	0.002	8.7e-23	8.2e-05	96.6
Lipid Metabolism	LDL	rs1571791	9	78729213	C	T	-0.014	0.001	4.7e-22	7.6e-05	93.2
Lipid Metabolism	LDL	rs9410207	9	91404799	C	T	-0.020	0.003	1.4e-12	4.1e-05	50.2
Lipid Metabolism	LDL	rs2401637	9	100541473	C	T	0.008	0.001	1.1e-08	2.7e-05	32.6
Lipid Metabolism	LDL	rs2777799	9	107559059	A	G	0.014	0.002	1.2e-09	3.0e-05	37.0
Lipid Metabolism	LDL	rs4149311	9	107588777	T	C	0.021	0.002	6.2e-22	7.6e-05	92.7
Lipid Metabolism	LDL	rs2740488	9	107661742	C	A	-0.025	0.002	6.7e-56	2.0e-04	248.1
Lipid Metabolism	LDL	rs56294298	9	117133524	A	G	-0.018	0.003	2.3e-12	4.0e-05	49.2
Lipid Metabolism	LDL	rs7025486	9	124422403	A	G	0.009	0.002	2.0e-09	2.9e-05	36.0
Lipid Metabolism	LDL	rs13289095	9	131466489	T	G	-0.029	0.002	1.5e-44	1.6e-04	196.0
Lipid Metabolism	LDL	rs139884023	9	135867131	A	G	0.068	0.011	1.4e-09	3.1e-05	36.6
Lipid Metabolism	LDL	rs2519093	9	136141870	T	C	0.072	0.002	1.0e-200	1.4e-03	1704.7
Lipid Metabolism	LDL	rs10901256	9	136163447	C	T	-0.024	0.002	9.6e-52	1.9e-04	229.0
Lipid Metabolism	LDL	rs3780190	9	139099073	G	A	-0.011	0.001	1.7e-15	5.2e-05	63.3
Lipid Metabolism	LDL	rs13301660	9	139340802	T	C	-0.016	0.002	2.2e-23	8.2e-05	99.3
Lipid Metabolism	LDL	rs7903259	10	17259642	G	C	0.016	0.001	1.9e-30	1.1e-04	131.6
Lipid Metabolism	LDL	rs1045873	10	25137772	C	A	0.008	0.001	3.0e-08	2.5e-05	30.7
Lipid Metabolism	LDL	rs12354765	10	43918823	A	T	-0.011	0.002	1.3e-09	3.5e-05	36.8
Lipid Metabolism	LDL	rs7908745	10	45953767	G	A	0.009	0.001	1.7e-09	2.9e-05	36.3
Lipid Metabolism	LDL	rs150770378	10	52616492	A	G	0.066	0.008	6.0e-16	5.9e-05	65.4
Lipid Metabolism	LDL	rs1800450	10	54531235	T	C	0.012	0.002	1.6e-09	3.0e-05	36.5
Lipid Metabolism	LDL	rs10761750	10	65128619	A	G	0.014	0.001	4.4e-24	8.3e-05	102.5
Lipid Metabolism	LDL	rs17476364	10	71094504	C	T	-0.027	0.002	1.1e-30	1.1e-04	132.5
Lipid Metabolism	LDL	rs79124660	10	74683921	A	G	-0.021	0.003	3.2e-10	3.2e-05	39.5
Lipid Metabolism	LDL	rs1782651	10	81076477	T	C	0.009	0.002	1.3e-08	2.8e-05	32.4
Lipid Metabolism	LDL	rs6586028	10	82253984	T	C	0.010	0.002	1.4e-09	3.0e-05	36.6
Lipid Metabolism	LDL	rs638846	10	89804585	G	C	-0.025	0.004	3.8e-11	3.6e-05	43.7
Lipid Metabolism	LDL	rs2250781	10	91007470	A	C	-0.009	0.001	6.0e-10	3.1e-05	38.3
Lipid Metabolism	LDL	rs11187263	10	94816817	C	T	-0.014	0.003	1.9e-08	2.9e-05	31.6
Lipid Metabolism	LDL	rs6583855	10	94843032	C	A	-0.015	0.001	1.2e-28	1.0e-04	123.3
Lipid Metabolism	LDL	rs77688653	10	96105877	T	C	-0.019	0.003	4.9e-11	3.5e-05	43.2
Lipid Metabolism	LDL	rs12354860	10	101902958	C	T	0.010	0.001	1.0e-12	4.2e-05	50.8
Lipid Metabolism	LDL	rs603424	10	102075479	A	G	0.016	0.002	3.7e-17	5.8e-05	70.9
Lipid Metabolism	LDL	rs78531123	10	104082953	A	G	0.018	0.003	3.5e-10	3.3e-05	39.4
Lipid Metabolism	LDL	rs2803621	10	113939584	A	G	-0.024	0.002	4.0e-54	1.9e-04	239.9
Lipid Metabolism	LDL	rs60191209	10	113988995	C	T	-0.019	0.002	1.1e-26	9.3e-05	114.3
Lipid Metabolism	LDL	rs7079542	10	115794898	G	A	-0.016	0.002	3.8e-13	4.4e-05	52.8
Lipid Metabolism	LDL	rs7910135	10	118398046	A	C	0.016	0.001	1.2e-29	1.0e-04	127.8
Lipid Metabolism	LDL	rs7904973	10	124693587	T	G	0.021	0.001	2.8e-50	1.8e-04	222.4
Lipid Metabolism	LDL	rs11246273	11	704864	A	G	0.008	0.001	3.4e-08	2.5e-05	30.4
Lipid Metabolism	LDL	rs35338528	11	1074635	G	T	-0.031	0.004	2.4e-15	5.1e-05	62.7
Lipid Metabolism	LDL	rs7124487	11	2988323	T	C	-0.012	0.002	2.0e-10	3.3e-05	40.4
Lipid Metabolism	LDL	rs188513096	11	4993424	A	T	-0.195	0.026	4.9e-14	6.4e-05	56.8
Lipid Metabolism	LDL	rs11038488	11	5663048	A	T	-0.012	0.002	6.0e-15	5.0e-05	60.9
Lipid Metabolism	LDL	rs3824949	11	5701409	C	G	0.014	0.001	6.2e-24	8.3e-05	101.8
Lipid Metabolism	LDL	rs7944706	11	10331311	A	G	0.008	0.001	7.1e-09	2.8e-05	33.5
Lipid Metabolism	LDL	rs11022130	11	12071547	G	A	0.010	0.002	6.1e-10	3.2e-05	38.3
Lipid Metabolism	LDL	rs11023881	11	16246700	A	T	-0.009	0.001	1.6e-09	3.0e-05	36.4
Lipid Metabolism	LDL	rs214093	11	17294788	G	C	0.008	0.001	3.2e-08	2.5e-05	30.6
Lipid Metabolism	LDL	rs2084220	11	18656932	T	C	0.021	0.002	4.0e-39	1.4e-04	171.2
Lipid Metabolism	LDL	rs10835849	11	32167943	C	T	0.010	0.002	4.0e-08	2.4e-05	30.2
Lipid Metabolism	LDL	rs61882680	11	46370636	T	C	-0.028	0.004	1.0e-11	3.8e-05	46.3

Phenotype	Trait	SNP	Chr	Position (GRCh37/hg19)	EA	NEA	BETA	SE	P-value	R2	F
Lipid Metabolism	LDL	rs1227995	11	47960210	T	C	-0.009	0.001	4.0e-10	3.2e-05	39.1
Lipid Metabolism	LDL	rs11230285	11	60123817	G	A	-0.009	0.002	2.9e-09	2.9e-05	35.3
Lipid Metabolism	LDL	rs174547	11	61570783	C	T	-0.043	0.001	2.9e-191	7.1e-04	870.3
Lipid Metabolism	LDL	rs79303190	11	61618016	C	T	-0.022	0.003	1.4e-12	4.1e-05	50.2
Lipid Metabolism	LDL	rs174618	11	61629322	C	T	-0.020	0.001	2.9e-44	1.6e-04	194.8
Lipid Metabolism	LDL	rs569620418	11	62407641	A	G	0.484	0.083	6.4e-09	8.5e-05	33.7
Lipid Metabolism	LDL	rs7950543	11	63599440	C	T	0.011	0.001	6.7e-15	5.0e-05	60.7
Lipid Metabolism	LDL	rs144256002	11	64039589	A	G	-0.022	0.004	1.9e-09	2.9e-05	36.1
Lipid Metabolism	LDL	rs4930295	11	65390554	G	C	0.012	0.002	8.6e-13	4.2e-05	51.1
Lipid Metabolism	LDL	rs79177013	11	65733465	A	G	0.017	0.003	3.3e-08	2.5e-05	30.5
Lipid Metabolism	LDL	rs117777720	11	66196384	T	C	-0.015	0.002	1.3e-19	6.7e-05	82.1
Lipid Metabolism	LDL	rs12803635	11	67224391	A	G	-0.024	0.003	1.3e-14	4.8e-05	59.4
Lipid Metabolism	LDL	rs11237488	11	78128775	T	C	-0.013	0.002	2.6e-10	3.2e-05	40.0
Lipid Metabolism	LDL	rs12295353	11	95470147	C	T	-0.008	0.001	1.9e-08	2.6e-05	31.6
Lipid Metabolism	LDL	rs10791660	11	103871039	A	C	-0.014	0.002	2.6e-15	5.1e-05	62.5
Lipid Metabolism	LDL	rs61906105	11	116550528	G	A	0.038	0.003	2.2e-32	1.1e-04	140.4
Lipid Metabolism	LDL	rs148945243	11	116601062	T	C	-0.050	0.007	9.9e-15	4.9e-05	59.9
Lipid Metabolism	LDL	rs964184	11	116648917	C	G	-0.055	0.002	9.1e-165	6.1e-04	748.4
Lipid Metabolism	LDL	rs45611741	11	116663128	T	C	0.094	0.009	3.7e-25	8.8e-05	107.4
Lipid Metabolism	LDL	rs138326449	11	116701354	A	G	-0.164	0.018	2.2e-20	7.3e-05	85.6
Lipid Metabolism	LDL	rs4639966	11	118573519	C	T	0.015	0.002	9.8e-20	6.7e-05	82.7
Lipid Metabolism	LDL	rs12795657	11	121372406	T	C	0.009	0.001	1.6e-10	3.4e-05	40.9
Lipid Metabolism	LDL	rs10750217	11	122531574	G	A	0.016	0.001	1.5e-27	9.6e-05	118.3
Lipid Metabolism	LDL	rs12418326	11	125978913	A	G	0.011	0.002	1.3e-08	2.6e-05	32.3
Lipid Metabolism	LDL	rs3862627	11	126181592	A	G	-0.013	0.002	2.9e-16	5.4e-05	66.9
Lipid Metabolism	LDL	rs181897168	11	126227095	A	G	-0.072	0.008	8.8e-18	6.0e-05	73.8
Lipid Metabolism	LDL	rs7928577	11	126227723	T	G	0.061	0.003	1.9e-116	4.3e-04	526.2
Lipid Metabolism	LDL	rs55943924	11	134294813	A	G	-0.012	0.002	2.5e-08	2.5e-05	31.1
Lipid Metabolism	LDL	rs35882350	12	623129	G	A	0.018	0.002	5.5e-29	1.0e-04	124.8
Lipid Metabolism	LDL	rs143750586	12	4358078	G	A	-0.059	0.010	8.1e-10	3.1e-05	37.7
Lipid Metabolism	LDL	rs147654565	12	21029965	A	G	-0.023	0.004	2.4e-09	2.9e-05	35.6
Lipid Metabolism	LDL	rs75667995	12	25409070	C	T	-0.029	0.003	1.4e-26	9.2e-05	113.9
Lipid Metabolism	LDL	rs11048645	12	26829961	A	T	-0.008	0.001	4.6e-09	2.8e-05	34.4
Lipid Metabolism	LDL	rs10843390	12	29496991	T	C	-0.010	0.002	2.8e-10	3.2e-05	39.8
Lipid Metabolism	LDL	rs11175540	12	40586295	A	T	0.026	0.003	5.1e-20	7.0e-05	83.9
Lipid Metabolism	LDL	rs2250751	12	51106178	A	G	-0.017	0.001	4.6e-29	1.0e-04	125.2
Lipid Metabolism	LDL	rs10876171	12	51793821	A	G	-0.013	0.001	6.7e-20	7.0e-05	83.4
Lipid Metabolism	LDL	rs7132175	12	53266339	A	G	0.009	0.001	4.3e-10	3.2e-05	39.0
Lipid Metabolism	LDL	rs7300593	12	53790450	C	T	0.014	0.002	3.3e-15	5.1e-05	62.1
Lipid Metabolism	LDL	rs1078604	12	57107154	T	G	-0.015	0.003	2.8e-08	2.5e-05	30.9
Lipid Metabolism	LDL	rs2122982	12	57781893	A	G	-0.014	0.002	2.4e-17	5.8e-05	71.8
Lipid Metabolism	LDL	rs61754230	12	72179446	T	C	0.049	0.006	1.2e-17	6.7e-05	73.2
Lipid Metabolism	LDL	rs12306780	12	89921860	T	A	0.012	0.001	3.0e-15	5.1e-05	62.3
Lipid Metabolism	LDL	rs7966546	12	92807320	T	C	0.009	0.001	8.0e-11	3.5e-05	42.3
Lipid Metabolism	LDL	rs11837065	12	100859983	T	C	-0.011	0.001	4.3e-14	4.7e-05	57.0
Lipid Metabolism	LDL	rs11610264	12	100932375	C	T	0.010	0.002	3.1e-10	3.3e-05	39.6
Lipid Metabolism	LDL	rs978458	12	102802239	C	T	-0.012	0.002	4.6e-14	4.6e-05	56.9
Lipid Metabolism	LDL	rs1850956	12	103453387	C	T	0.008	0.001	5.3e-09	2.8e-05	34.1
Lipid Metabolism	LDL	rs11112451	12	105688430	G	A	0.011	0.002	6.4e-09	2.8e-05	33.7
Lipid Metabolism	LDL	rs11114053	12	109168515	T	C	0.013	0.002	1.8e-18	6.2e-05	76.9
Lipid Metabolism	LDL	rs118014788	12	109611786	T	G	0.028	0.004	7.9e-11	3.4e-05	42.3
Lipid Metabolism	LDL	rs149793040	12	109661672	G	A	-0.113	0.021	4.0e-08	3.1e-05	30.1
Lipid Metabolism	LDL	rs11065979	12	112059557	T	C	-0.022	0.001	1.1e-55	2.0e-04	247.1
Lipid Metabolism	LDL	rs232923	12	113104241	G	C	-0.010	0.001	8.2e-13	4.2e-05	51.2
Lipid Metabolism	LDL	rs11066391	12	113163771	T	G	0.015	0.002	2.0e-21	7.4e-05	90.3
Lipid Metabolism	LDL	rs1634536	12	120856874	A	T	-0.008	0.001	1.8e-09	3.0e-05	36.2

Phenotype	Trait	SNP	Chr	Position (GRCh37/hg19)	EA	NEA	BETA	SE	P-value	R2	F
Lipid Metabolism	LDL	rs541619	12	121096987	G	A	0.012	0.002	8.8e-14	4.6e-05	55.6
Lipid Metabolism	LDL	rs2244608	12	121416988	G	A	0.035	0.001	2.5e-124	4.6e-04	562.4
Lipid Metabolism	LDL	rs1182946	12	121601893	C	A	0.023	0.004	4.8e-08	2.4e-05	29.8
Lipid Metabolism	LDL	rs895959	12	122320352	T	C	-0.013	0.002	2.2e-10	3.3e-05	40.3
Lipid Metabolism	LDL	rs28532037	12	123883406	A	G	-0.020	0.002	3.7e-17	5.8e-05	70.9
Lipid Metabolism	LDL	rs146326139	12	124157175	C	G	-0.045	0.006	1.4e-12	4.1e-05	50.2
Lipid Metabolism	LDL	rs825456	12	124537575	A	G	0.010	0.001	7.5e-13	4.2e-05	51.4
Lipid Metabolism	LDL	rs73223595	12	124853700	T	A	0.012	0.002	3.6e-10	3.2e-05	39.3
Lipid Metabolism	LDL	rs11057830	12	125307053	A	G	0.022	0.002	2.2e-27	9.6e-05	117.6
Lipid Metabolism	LDL	rs75588192	12	133048600	A	G	0.016	0.002	8.5e-15	5.5e-05	60.2
Lipid Metabolism	LDL	rs34287227	12	133458175	T	C	0.015	0.003	1.3e-08	2.9e-05	32.3
Lipid Metabolism	LDL	rs11619246	13	31007977	T	C	-0.008	0.001	1.6e-08	2.7e-05	31.9
Lipid Metabolism	LDL	rs9315148	13	32481926	T	C	-0.011	0.002	5.7e-11	3.5e-05	42.9
Lipid Metabolism	LDL	rs2238162	13	32959199	T	C	-0.020	0.001	1.8e-45	1.6e-04	200.3
Lipid Metabolism	LDL	rs208426	13	33191371	C	T	-0.011	0.002	3.3e-14	4.7e-05	57.6
Lipid Metabolism	LDL	rs17532371	13	41635301	G	C	-0.019	0.003	9.5e-13	4.1e-05	50.9
Lipid Metabolism	LDL	rs7317982	13	50108252	C	T	-0.009	0.001	4.2e-10	3.2e-05	39.0
Lipid Metabolism	LDL	rs9535746	13	52317358	G	A	0.012	0.002	4.8e-10	3.1e-05	38.8
Lipid Metabolism	LDL	rs9592980	13	74855425	A	G	0.010	0.001	2.3e-11	3.7e-05	44.7
Lipid Metabolism	LDL	rs35049975	13	76034297	T	A	0.012	0.002	1.3e-08	2.7e-05	32.4
Lipid Metabolism	LDL	rs7330899	13	95223400	A	G	0.014	0.002	6.3e-19	6.4e-05	79.0
Lipid Metabolism	LDL	rs4771674	13	111039070	G	A	0.014	0.001	3.0e-22	7.7e-05	94.1
Lipid Metabolism	LDL	rs7140110	13	114544024	C	T	0.021	0.002	5.8e-42	1.5e-04	184.2
Lipid Metabolism	LDL	rs12016920	13	114639025	C	T	-0.017	0.002	1.6e-21	7.5e-05	90.8
Lipid Metabolism	LDL	rs147773754	14	21492374	T	C	0.071	0.013	1.6e-08	2.6e-05	32.0
Lipid Metabolism	LDL	rs11621792	14	24871926	T	C	0.022	0.001	8.1e-54	2.0e-04	238.5
Lipid Metabolism	LDL	rs139262716	14	31725111	A	G	-0.039	0.005	4.1e-13	4.3e-05	52.6
Lipid Metabolism	LDL	rs72677070	14	35165238	A	G	-0.013	0.002	2.0e-08	2.6e-05	31.5
Lipid Metabolism	LDL	rs8005490	14	35184541	C	T	0.011	0.001	3.4e-14	4.7e-05	57.5
Lipid Metabolism	LDL	rs114491880	14	51373247	T	A	0.033	0.005	3.4e-10	3.2e-05	39.4
Lipid Metabolism	LDL	rs12897637	14	64239351	C	T	0.016	0.002	3.4e-16	5.4e-05	66.6
Lipid Metabolism	LDL	rs10139233	14	70859431	T	C	0.025	0.002	2.9e-36	1.3e-04	158.1
Lipid Metabolism	LDL	rs13379042	14	74250100	C	T	-0.016	0.002	1.5e-25	8.9e-05	109.1
Lipid Metabolism	LDL	rs34956813	14	75254167	A	G	-0.009	0.001	1.6e-11	3.7e-05	45.4
Lipid Metabolism	LDL	rs35802157	14	90066634	C	T	-0.010	0.001	1.9e-11	3.7e-05	45.1
Lipid Metabolism	LDL	rs112458284	14	94672731	C	T	0.026	0.004	2.0e-10	3.3e-05	40.5
Lipid Metabolism	LDL	rs17580	14	94847262	A	T	0.050	0.004	3.3e-43	1.6e-04	189.9
Lipid Metabolism	LDL	rs1004572	14	101178330	A	G	0.012	0.002	6.3e-09	2.8e-05	33.8
Lipid Metabolism	LDL	rs2413926	15	49336477	A	T	-0.013	0.001	1.1e-20	7.1e-05	87.0
Lipid Metabolism	LDL	rs79101008	15	53076370	A	C	-0.014	0.002	1.4e-10	3.3e-05	41.2
Lipid Metabolism	LDL	rs79391862	15	53739426	C	A	-0.064	0.005	3.3e-33	1.2e-04	144.2
Lipid Metabolism	LDL	rs72749499	15	57295579	G	A	0.029	0.003	5.3e-25	8.7e-05	106.7
Lipid Metabolism	LDL	rs10468017	15	58678512	T	C	0.011	0.002	1.1e-13	4.5e-05	55.1
Lipid Metabolism	LDL	rs261332	15	58727325	G	A	-0.014	0.002	3.0e-15	5.1e-05	62.3
Lipid Metabolism	LDL	rs2414759	15	62366721	T	C	0.008	0.001	4.6e-08	2.4e-05	29.9
Lipid Metabolism	LDL	rs11636087	15	63788267	C	T	0.014	0.002	1.3e-20	7.1e-05	86.7
Lipid Metabolism	LDL	rs28658260	15	66916052	G	A	0.009	0.002	1.4e-08	2.6e-05	32.2
Lipid Metabolism	LDL	rs4776342	15	67418391	G	A	0.009	0.002	4.8e-08	2.4e-05	29.8
Lipid Metabolism	LDL	rs12917376	15	75116167	T	C	0.013	0.001	8.8e-19	6.5e-05	78.3
Lipid Metabolism	LDL	rs2458250	15	77270131	T	A	-0.008	0.001	2.6e-08	2.5e-05	31.0
Lipid Metabolism	LDL	rs8038512	15	89106233	G	T	0.009	0.001	4.3e-10	3.2e-05	39.0
Lipid Metabolism	LDL	rs576644950	15	101744507	T	C	-0.070	0.011	4.6e-10	3.2e-05	38.8
Lipid Metabolism	LDL	rs8029775	15	101906129	C	T	-0.009	0.001	2.1e-10	3.3e-05	40.3
Lipid Metabolism	LDL	rs17703146	16	4860105	C	T	0.011	0.002	8.8e-09	2.7e-05	33.1
Lipid Metabolism	LDL	rs4280262	16	11647492	C	T	0.010	0.002	1.5e-08	2.7e-05	32.1
Lipid Metabolism	LDL	rs57900082	16	11679101	G	A	0.018	0.002	7.5e-22	7.7e-05	92.3

Phenotype	Trait	SNP	Chr	Position (GRCh37/hg19)	EA	NEA	BETA	SE	P-value	R2	F
Lipid Metabolism	LDL	rs12928099	16	15150505	A	C	0.009	0.002	2.8e-08	2.5e-05	30.9
Lipid Metabolism	LDL	rs72772042	16	15863759	A	C	-0.012	0.002	3.1e-10	3.2e-05	39.6
Lipid Metabolism	LDL	rs17839567	16	31057945	G	A	0.012	0.001	1.2e-16	5.6e-05	68.6
Lipid Metabolism	LDL	rs74019283	16	55870239	T	G	-0.021	0.004	3.0e-08	2.5e-05	30.7
Lipid Metabolism	LDL	rs140770781	16	56925500	T	C	0.063	0.011	2.1e-08	2.6e-05	31.4
Lipid Metabolism	LDL	rs247617	16	56990716	A	C	-0.037	0.001	3.1e-135	5.0e-04	612.5
Lipid Metabolism	LDL	rs1075349	16	65199587	T	C	-0.008	0.001	2.2e-08	2.6e-05	31.3
Lipid Metabolism	LDL	rs12149704	16	69789516	A	G	0.023	0.003	5.1e-14	4.6e-05	56.7
Lipid Metabolism	LDL	rs62048010	16	70803755	C	T	0.024	0.003	9.0e-15	4.9e-05	60.1
Lipid Metabolism	LDL	rs74340438	16	70807284	T	C	0.027	0.004	1.5e-13	4.4e-05	54.5
Lipid Metabolism	LDL	rs11646091	16	70825649	C	T	-0.020	0.004	4.0e-08	2.4e-05	30.1
Lipid Metabolism	LDL	rs56212732	16	70930370	T	C	-0.027	0.003	9.7e-23	7.9e-05	96.3
Lipid Metabolism	LDL	rs71403812	16	71014962	A	G	0.025	0.004	4.5e-10	3.3e-05	38.9
Lipid Metabolism	LDL	rs191625687	16	71785389	C	A	-0.058	0.009	3.1e-11	4.2e-05	44.1
Lipid Metabolism	LDL	rs12930656	16	71863880	A	G	-0.033	0.005	5.5e-10	3.1e-05	38.5
Lipid Metabolism	LDL	rs193210477	16	71992460	A	C	-0.064	0.009	1.4e-13	5.0e-05	54.7
Lipid Metabolism	LDL	rs141154498	16	72030455	A	G	-0.072	0.011	1.4e-10	3.4e-05	41.1
Lipid Metabolism	LDL	rs12924886	16	72075593	T	A	0.057	0.002	1.0e-200	8.5e-04	1044.8
Lipid Metabolism	LDL	rs563195118	16	72087343	T	C	-0.098	0.016	1.2e-09	4.5e-05	36.9
Lipid Metabolism	LDL	rs117660239	16	72109892	A	T	-0.038	0.007	1.1e-08	2.7e-05	32.6
Lipid Metabolism	LDL	rs141181263	16	72140548	G	A	-0.043	0.007	5.5e-09	2.8e-05	34.0
Lipid Metabolism	LDL	rs11647069	16	72155237	C	T	0.023	0.001	3.5e-53	1.9e-04	235.7
Lipid Metabolism	LDL	rs142985659	16	72171853	C	T	-0.048	0.008	2.2e-10	3.7e-05	40.3
Lipid Metabolism	LDL	rs188598167	16	72207445	C	T	-0.061	0.009	3.5e-11	3.6e-05	43.9
Lipid Metabolism	LDL	rs113368886	16	72303317	C	T	-0.033	0.002	2.7e-40	1.4e-04	176.6
Lipid Metabolism	LDL	rs1396703	16	79358608	T	C	0.009	0.001	1.9e-09	3.0e-05	36.1
Lipid Metabolism	LDL	rs1862719	16	79504057	G	A	-0.011	0.002	3.5e-11	3.6e-05	43.9
Lipid Metabolism	LDL	rs11149612	16	83980965	T	C	-0.016	0.001	6.9e-30	1.1e-04	129.0
Lipid Metabolism	LDL	rs904789	16	88528722	G	A	-0.010	0.002	1.3e-10	3.4e-05	41.3
Lipid Metabolism	LDL	rs74035509	16	88567333	T	C	0.023	0.003	3.0e-17	5.9e-05	71.3
Lipid Metabolism	LDL	rs216217	17	2145090	A	G	0.009	0.001	1.2e-09	3.0e-05	37.0
Lipid Metabolism	LDL	rs11656732	17	4670778	T	C	0.014	0.001	8.7e-22	7.6e-05	92.0
Lipid Metabolism	LDL	rs140779352	17	4672983	A	C	-0.086	0.009	2.6e-22	7.8e-05	94.4
Lipid Metabolism	LDL	rs188002707	17	6839871	T	C	-0.284	0.035	2.9e-16	7.5e-05	66.9
Lipid Metabolism	LDL	rs577492325	17	6918254	C	A	-0.247	0.033	6.2e-14	6.8e-05	56.3
Lipid Metabolism	LDL	rs186021206	17	7069412	A	G	-0.324	0.011	1.0e-200	7.6e-04	917.9
Lipid Metabolism	LDL	rs72837687	17	7073747	A	G	-0.033	0.002	1.1e-72	2.8e-04	325.1
Lipid Metabolism	LDL	rs577996183	17	7105556	A	T	-0.154	0.027	1.9e-08	3.3e-05	31.6
Lipid Metabolism	LDL	rs72842814	17	7314137	A	G	-0.026	0.004	1.1e-09	3.0e-05	37.1
Lipid Metabolism	LDL	rs150438405	17	7443059	C	G	0.037	0.007	3.3e-08	2.5e-05	30.5
Lipid Metabolism	LDL	rs12949853	17	7570878	A	G	-0.013	0.002	5.4e-13	4.3e-05	52.1
Lipid Metabolism	LDL	rs9908765	17	8214427	A	G	0.014	0.001	8.5e-24	8.3e-05	101.2
Lipid Metabolism	LDL	rs542092519	17	8431576	A	C	-0.379	0.064	3.2e-09	8.1e-05	35.1
Lipid Metabolism	LDL	rs17681708	17	9792872	C	T	0.009	0.001	5.1e-10	3.1e-05	38.6
Lipid Metabolism	LDL	rs527671460	17	11428501	A	G	-0.698	0.126	2.9e-08	7.7e-05	30.8
Lipid Metabolism	LDL	rs71367414	17	17634550	T	C	-0.013	0.002	1.7e-12	4.3e-05	49.8
Lipid Metabolism	LDL	rs62072467	17	18116455	T	C	0.013	0.002	7.7e-13	4.2e-05	51.4
Lipid Metabolism	LDL	rs704	17	26694861	A	G	0.019	0.001	5.1e-42	1.5e-04	184.5
Lipid Metabolism	LDL	rs11080072	17	27081160	T	G	-0.013	0.002	9.3e-15	4.9e-05	60.0
Lipid Metabolism	LDL	rs56336338	17	27645258	A	G	-0.019	0.002	1.0e-20	7.1e-05	87.2
Lipid Metabolism	LDL	rs56881390	17	28603828	C	T	-0.017	0.002	1.0e-11	3.8e-05	46.3
Lipid Metabolism	LDL	rs4435317	17	29436512	C	G	0.017	0.002	8.3e-29	1.0e-04	124.0
Lipid Metabolism	LDL	rs118073397	17	33390766	C	T	-0.068	0.011	6.4e-10	3.2e-05	38.2
Lipid Metabolism	LDL	rs12943633	17	37970365	T	C	-0.018	0.003	3.5e-12	3.9e-05	48.4
Lipid Metabolism	LDL	rs12150495	17	40388430	A	G	-0.016	0.003	1.1e-09	3.1e-05	37.2
Lipid Metabolism	LDL	rs72826970	17	40883541	G	A	0.020	0.003	1.4e-08	2.6e-05	32.2

Phenotype	Trait	SNP	Chr	Position (GRCh37/hg19)	EA	NEA	BETA	SE	P-value	R2	F
Lipid Metabolism	LDL	rs72836561	17	41926126	T	C	-0.030	0.004	1.8e-13	4.4e-05	54.2
Lipid Metabolism	LDL	rs185129555	17	45399047	T	A	-0.035	0.005	1.9e-10	3.4e-05	40.5
Lipid Metabolism	LDL	rs12603290	17	45650196	C	T	-0.027	0.001	2.0e-77	2.9e-04	346.9
Lipid Metabolism	LDL	rs62076102	17	45887132	A	G	-0.020	0.002	6.9e-16	5.3e-05	65.2
Lipid Metabolism	LDL	rs4793944	17	46667304	T	C	0.013	0.001	1.5e-20	7.1e-05	86.3
Lipid Metabolism	LDL	rs1292061	17	57911230	G	A	0.010	0.001	1.2e-12	4.1e-05	50.5
Lipid Metabolism	LDL	rs3020619	17	61993137	G	A	-0.009	0.002	1.4e-08	2.6e-05	32.2
Lipid Metabolism	LDL	rs8178824	17	64224775	T	C	0.091	0.004	5.6e-98	3.6e-04	441.3
Lipid Metabolism	LDL	rs34310593	17	65252129	A	G	-0.011	0.002	4.3e-10	3.2e-05	39.0
Lipid Metabolism	LDL	rs78186330	17	66004715	A	G	0.016	0.002	2.2e-20	7.2e-05	85.6
Lipid Metabolism	LDL	rs34931250	17	66879927	T	C	0.034	0.003	3.2e-30	1.1e-04	130.5
Lipid Metabolism	LDL	rs140522356	17	67012841	T	C	-0.064	0.008	2.7e-14	4.8e-05	57.9
Lipid Metabolism	LDL	rs77542162	17	67081278	G	A	0.183	0.005	1.0e-200	1.1e-03	1297.3
Lipid Metabolism	LDL	rs143856581	17	67084059	T	C	0.381	0.070	4.8e-08	3.6e-05	29.8
Lipid Metabolism	LDL	rs138479597	17	67111065	A	G	-0.063	0.011	2.1e-08	2.6e-05	31.4
Lipid Metabolism	LDL	rs550836771	17	67135617	T	G	-0.059	0.008	3.2e-12	4.6e-05	48.6
Lipid Metabolism	LDL	rs145958325	17	67165906	C	T	-0.060	0.008	7.4e-15	5.0e-05	60.5
Lipid Metabolism	LDL	rs77049332	17	67226150	G	A	-0.037	0.004	2.0e-25	8.9e-05	108.5
Lipid Metabolism	LDL	rs34366352	17	68161384	C	T	0.016	0.002	5.4e-13	4.2e-05	52.1
Lipid Metabolism	LDL	rs1979215	17	73761966	T	C	0.016	0.002	3.4e-25	8.8e-05	107.5
Lipid Metabolism	LDL	rs11657987	17	76387363	T	G	0.018	0.001	1.0e-36	1.3e-04	160.2
Lipid Metabolism	LDL	rs7501528	17	76658346	G	C	-0.011	0.002	1.6e-09	3.1e-05	36.4
Lipid Metabolism	LDL	rs11656152	17	80994674	T	C	0.012	0.002	1.3e-10	3.4e-05	41.2
Lipid Metabolism	LDL	rs62088911	18	9615667	G	T	0.015	0.002	8.9e-11	3.4e-05	42.1
Lipid Metabolism	LDL	rs1010810	18	19677934	A	G	-0.013	0.002	1.6e-09	3.0e-05	36.5
Lipid Metabolism	LDL	rs76537328	18	42072173	G	C	-0.012	0.002	4.6e-09	2.8e-05	34.3
Lipid Metabolism	LDL	rs149615216	18	47106028	T	C	0.075	0.007	1.3e-28	1.0e-04	123.1
Lipid Metabolism	LDL	rs3744843	18	47117174	C	T	0.020	0.003	1.4e-09	3.0e-05	36.7
Lipid Metabolism	LDL	rs7240405	18	47159090	G	A	0.018	0.002	1.2e-22	7.8e-05	95.8
Lipid Metabolism	LDL	rs12968116	18	55322502	T	C	0.014	0.002	1.2e-11	3.7e-05	46.0
Lipid Metabolism	LDL	rs185081762	18	65608586	T	C	0.501	0.088	1.0e-08	8.2e-05	32.8
Lipid Metabolism	LDL	rs8096658	18	77156537	G	C	-0.008	0.001	1.4e-08	2.7e-05	32.2
Lipid Metabolism	LDL	rs11081591	18	77880802	A	G	-0.012	0.002	1.2e-08	2.9e-05	32.5
Lipid Metabolism	LDL	rs4807570	19	1123652	A	G	-0.012	0.002	1.5e-11	3.7e-05	45.5
Lipid Metabolism	LDL	rs4807332	19	2793289	C	T	0.015	0.002	2.8e-24	8.5e-05	103.3
Lipid Metabolism	LDL	rs892161	19	4493708	G	A	-0.011	0.001	7.6e-14	4.6e-05	55.9
Lipid Metabolism	LDL	rs151006362	19	4753266	A	G	0.013	0.002	1.0e-09	3.0e-05	37.3
Lipid Metabolism	LDL	rs708686	19	5840619	T	C	0.011	0.002	1.4e-12	4.1e-05	50.1
Lipid Metabolism	LDL	rs571497	19	7827830	A	G	-0.015	0.002	8.2e-14	4.5e-05	55.8
Lipid Metabolism	LDL	rs60006507	19	8394937	A	G	-0.010	0.002	1.4e-09	3.0e-05	36.7
Lipid Metabolism	LDL	rs559470475	19	9366002	T	C	-0.061	0.011	1.9e-08	2.6e-05	31.6
Lipid Metabolism	LDL	rs2287802	19	10112688	G	A	0.010	0.002	1.0e-10	3.5e-05	41.8
Lipid Metabolism	LDL	rs557704368	19	10234409	G	A	-0.106	0.011	2.0e-20	7.3e-05	85.8
Lipid Metabolism	LDL	rs576769308	19	10237384	G	A	-0.133	0.023	5.3e-09	4.5e-05	34.1
Lipid Metabolism	LDL	rs564800172	19	10261309	T	C	-0.237	0.022	9.8e-28	1.7e-04	119.1
Lipid Metabolism	LDL	rs570748267	19	10461755	T	C	0.263	0.043	1.2e-09	6.8e-05	37.0
Lipid Metabolism	LDL	rs12975360	19	10537485	G	A	0.073	0.011	1.2e-11	3.9e-05	45.9
Lipid Metabolism	LDL	rs35518329	19	10636818	C	T	0.025	0.002	2.4e-40	1.8e-04	176.9
Lipid Metabolism	LDL	rs546689040	19	10639478	T	C	-0.192	0.019	1.4e-23	2.5e-04	100.2
Lipid Metabolism	LDL	rs184823980	19	10655605	A	G	-0.102	0.014	5.0e-14	5.2e-05	56.7
Lipid Metabolism	LDL	rs552836936	19	10663402	C	T	-0.230	0.038	1.0e-09	9.1e-05	37.3
Lipid Metabolism	LDL	rs563756708	19	10687621	T	C	0.889	0.141	2.6e-10	1.0e-04	39.9
Lipid Metabolism	LDL	rs150005806	19	10693149	A	G	-0.362	0.063	7.1e-09	5.6e-05	33.5
Lipid Metabolism	LDL	rs542777597	19	10730126	G	T	-0.207	0.028	8.7e-14	6.2e-05	55.6
Lipid Metabolism	LDL	rs191488621	19	10738826	T	C	-0.319	0.056	1.1e-08	5.5e-05	32.7
Lipid Metabolism	LDL	rs538971370	19	10752814	A	C	0.083	0.014	4.9e-09	5.0e-05	34.2

Phenotype	Trait	SNP	Chr	Position (GRCh37/hg19)	EA	NEA	BETA	SE	P-value	R2	F
Lipid Metabolism	LDL	rs191563172	19	10762999	A	G	0.151	0.028	4.9e-08	7.0e-05	29.8
Lipid Metabolism	LDL	rs572009601	19	10767520	T	C	0.071	0.010	4.8e-13	5.6e-05	52.3
Lipid Metabolism	LDL	rs181305866	19	10773834	A	G	-0.119	0.020	1.7e-09	4.5e-05	36.3
Lipid Metabolism	LDL	rs148477953	19	10847987	T	C	-0.067	0.012	2.8e-08	2.6e-05	30.9
Lipid Metabolism	LDL	rs148790687	19	10883157	T	C	-0.118	0.016	4.4e-13	4.6e-05	52.4
Lipid Metabolism	LDL	rs572821566	19	10897010	T	C	-0.119	0.020	4.2e-09	4.3e-05	34.5
Lipid Metabolism	LDL	rs1109375	19	10914719	G	C	-0.072	0.002	1.0e-200	1.3e-03	1553.4
Lipid Metabolism	LDL	rs547704470	19	10918373	G	T	0.149	0.018	4.7e-16	8.7e-05	65.9
Lipid Metabolism	LDL	rs185728685	19	10952199	C	T	0.470	0.060	4.1e-15	1.5e-04	61.7
Lipid Metabolism	LDL	rs200169512	19	11032164	A	G	-0.321	0.039	1.6e-16	1.0e-04	68.0
Lipid Metabolism	LDL	rs182491671	19	11036679	T	C	0.298	0.042	1.1e-12	5.7e-05	50.6
Lipid Metabolism	LDL	rs189389232	19	11049371	A	G	0.122	0.018	8.8e-12	5.6e-05	46.6
Lipid Metabolism	LDL	rs574982898	19	11071839	T	C	-0.326	0.040	2.7e-16	1.3e-04	67.0
Lipid Metabolism	LDL	rs145648099	19	11073440	C	A	0.086	0.014	3.5e-09	3.2e-05	34.9
Lipid Metabolism	LDL	rs140701596	19	11090248	A	G	0.110	0.016	1.1e-11	4.1e-05	46.2
Lipid Metabolism	LDL	rs548746023	19	11104727	C	T	-0.406	0.047	2.6e-18	1.9e-04	76.2
Lipid Metabolism	LDL	rs557024333	19	11125383	T	C	-0.250	0.044	1.6e-08	4.8e-05	31.9
Lipid Metabolism	LDL	rs192566697	19	11131652	T	G	-0.209	0.033	2.2e-10	5.7e-05	40.2
Lipid Metabolism	LDL	rs144108046	19	11140707	T	C	-0.506	0.091	2.5e-08	5.1e-05	31.0
Lipid Metabolism	LDL	rs562146065	19	11148274	A	G	-0.181	0.031	8.6e-09	4.6e-05	33.1
Lipid Metabolism	LDL	rs112634605	19	11150013	C	G	-0.109	0.004	1.0e-170	6.3e-04	775.7
Lipid Metabolism	LDL	rs189076912	19	11155283	A	C	0.133	0.020	1.2e-11	6.3e-05	46.0
Lipid Metabolism	LDL	rs192255738	19	11158575	T	C	0.128	0.018	9.9e-13	4.3e-05	50.9
Lipid Metabolism	LDL	rs143580067	19	11161477	A	G	0.078	0.009	4.6e-18	6.1e-05	75.0
Lipid Metabolism	LDL	rs534203832	19	11166422	A	C	0.160	0.027	4.9e-09	4.5e-05	34.2
Lipid Metabolism	LDL	rs371899114	19	11177496	A	G	0.085	0.012	8.8e-13	4.3e-05	51.1
Lipid Metabolism	LDL	rs188188102	19	11178961	G	A	0.058	0.009	1.3e-10	3.5e-05	41.3
Lipid Metabolism	LDL	rs10423733	19	11185919	C	T	-0.131	0.002	1.0e-200	4.0e-03	4977.0
Lipid Metabolism	LDL	rs551719483	19	11189265	C	T	0.078	0.013	2.7e-09	3.0e-05	35.4
Lipid Metabolism	LDL	rs537829044	19	11204927	C	T	-0.469	0.080	4.2e-09	5.4e-05	34.5
Lipid Metabolism	LDL	rs553539173	19	11205304	A	G	0.156	0.025	2.0e-10	5.4e-05	40.4
Lipid Metabolism	LDL	rs180760728	19	11219281	C	T	-0.345	0.008	1.0e-200	1.5e-03	1739.5
Lipid Metabolism	LDL	rs568336016	19	11219624	T	C	0.231	0.036	1.2e-10	1.1e-04	41.6
Lipid Metabolism	LDL	rs55792959	19	11221289	T	C	-0.098	0.011	3.5e-20	7.0e-05	84.7
Lipid Metabolism	LDL	rs192390193	19	11221487	A	G	0.114	0.013	5.1e-18	6.2e-05	74.8
Lipid Metabolism	LDL	rs180876220	19	11223083	G	C	0.107	0.010	1.5e-28	1.0e-04	122.9
Lipid Metabolism	LDL	rs527357541	19	11227855	A	C	0.216	0.034	2.1e-10	1.0e-04	40.4
Lipid Metabolism	LDL	rs12611153	19	11230332	T	C	-0.040	0.002	1.6e-126	4.7e-04	572.5
Lipid Metabolism	LDL	rs527424716	19	11231402	A	G	-0.179	0.028	1.6e-10	4.1e-05	40.8
Lipid Metabolism	LDL	rs563930198	19	11232812	T	C	0.067	0.008	1.3e-18	7.5e-05	77.6
Lipid Metabolism	LDL	rs45508991	19	11233886	T	C	0.105	0.007	1.5e-47	1.7e-04	209.9
Lipid Metabolism	LDL	rs181994650	19	11235610	T	C	0.074	0.011	4.8e-11	5.1e-05	43.2
Lipid Metabolism	LDL	rs531359724	19	11250445	A	G	-0.323	0.045	9.7e-13	6.3e-05	50.9
Lipid Metabolism	LDL	rs553556107	19	11259571	T	C	0.085	0.009	2.3e-20	7.0e-05	85.5
Lipid Metabolism	LDL	rs189143685	19	11274168	T	C	0.049	0.007	7.3e-13	4.2e-05	51.4
Lipid Metabolism	LDL	rs148079581	19	11274668	C	A	0.092	0.009	5.0e-27	9.5e-05	115.9
Lipid Metabolism	LDL	rs570974999	19	11280249	T	C	0.090	0.014	1.4e-10	3.5e-05	41.2
Lipid Metabolism	LDL	rs193296317	19	11284560	T	C	-0.149	0.022	6.4e-12	4.5e-05	47.2
Lipid Metabolism	LDL	rs151111259	19	11294226	A	G	-0.199	0.036	4.2e-08	4.4e-05	30.1
Lipid Metabolism	LDL	rs12980863	19	11309871	T	C	0.022	0.001	4.5e-55	2.0e-04	244.3
Lipid Metabolism	LDL	rs200393834	19	11311386	A	G	0.108	0.016	4.9e-12	4.2e-05	47.7
Lipid Metabolism	LDL	rs564214041	19	11330355	G	A	0.083	0.015	1.5e-08	3.4e-05	32.1
Lipid Metabolism	LDL	rs139347491	19	11334329	T	A	-0.137	0.011	6.6e-36	1.3e-04	156.5
Lipid Metabolism	LDL	rs190268411	19	11334682	C	T	-0.070	0.012	1.8e-09	3.0e-05	36.2
Lipid Metabolism	LDL	rs563468321	19	11416795	G	T	-0.262	0.042	2.7e-10	9.8e-05	39.8
Lipid Metabolism	LDL	rs536959248	19	11446539	T	A	-0.223	0.034	7.7e-11	6.3e-05	42.3

Phenotype	Trait	SNP	Chr	Position (GRCh37/hg19)	EA	NEA	BETA	SE	P-value	R2	F
Lipid Metabolism	LDL	rs183085199	19	11507217	A	G	-0.120	0.011	4.1e-28	1.4e-04	120.9
Lipid Metabolism	LDL	rs181006367	19	11549886	T	C	-0.115	0.020	6.2e-09	4.3e-05	33.8
Lipid Metabolism	LDL	rs183540342	19	11685354	G	A	0.097	0.017	1.3e-08	2.9e-05	32.4
Lipid Metabolism	LDL	rs558811253	19	11737127	A	G	-0.132	0.020	9.4e-11	6.0e-05	41.9
Lipid Metabolism	LDL	rs534770939	19	11777205	C	T	-0.085	0.010	4.6e-16	6.3e-05	66.0
Lipid Metabolism	LDL	rs543464461	19	12174123	T	C	-0.102	0.017	2.5e-09	4.0e-05	35.5
Lipid Metabolism	LDL	rs553952570	19	12749320	G	A	-0.493	0.060	1.6e-16	1.4e-04	68.1
Lipid Metabolism	LDL	rs62109911	19	13234145	A	G	-0.010	0.002	3.0e-09	2.9e-05	35.2
Lipid Metabolism	LDL	rs560788989	19	13250023	C	T	1.297	0.146	6.3e-19	1.2e-04	79.0
Lipid Metabolism	LDL	rs527932938	19	13295968	T	C	-0.253	0.032	3.6e-15	9.8e-05	61.9
Lipid Metabolism	LDL	rs574000814	19	13766888	A	G	-0.343	0.061	2.2e-08	6.6e-05	31.4
Lipid Metabolism	LDL	rs36014207	19	14172728	T	C	-0.011	0.002	6.8e-13	4.5e-05	51.6
Lipid Metabolism	LDL	rs633553	19	15796363	G	A	0.010	0.001	3.3e-13	4.3e-05	53.0
Lipid Metabolism	LDL	rs59997441	19	17381463	C	T	0.009	0.001	1.0e-10	3.4e-05	41.8
Lipid Metabolism	LDL	rs12972417	19	17525314	C	T	-0.013	0.002	4.4e-13	4.3e-05	52.4
Lipid Metabolism	LDL	rs4808765	19	18334443	T	C	0.014	0.002	2.0e-18	6.3e-05	76.7
Lipid Metabolism	LDL	rs10423802	19	18567901	T	C	0.012	0.002	4.1e-12	3.9e-05	48.1
Lipid Metabolism	LDL	rs547734426	19	19254381	T	C	-0.161	0.019	8.8e-17	8.5e-05	69.2
Lipid Metabolism	LDL	rs542068965	19	19354844	G	A	-0.214	0.030	2.0e-12	7.0e-05	49.5
Lipid Metabolism	LDL	rs55989964	19	19361152	G	C	0.027	0.004	2.1e-14	4.8e-05	58.5
Lipid Metabolism	LDL	rs72999033	19	19366632	T	C	-0.111	0.003	1.0e-200	1.2e-03	1511.2
Lipid Metabolism	LDL	rs187429064	19	19380513	G	A	-0.160	0.007	1.4e-118	4.5e-04	536.0
Lipid Metabolism	LDL	rs144821371	19	19381973	A	G	-0.118	0.013	1.1e-19	7.3e-05	82.3
Lipid Metabolism	LDL	rs541344524	19	19427886	C	G	-0.175	0.023	6.9e-14	1.4e-04	56.1
Lipid Metabolism	LDL	rs117970794	19	19468655	T	C	-0.138	0.010	2.0e-39	1.5e-04	172.7
Lipid Metabolism	LDL	rs535451845	19	19504211	G	A	-0.262	0.042	6.2e-10	5.6e-05	38.3
Lipid Metabolism	LDL	rs144984216	19	20479901	T	C	-0.051	0.005	1.6e-21	1.1e-04	90.8
Lipid Metabolism	LDL	rs141904453	19	21635117	C	T	-0.031	0.005	2.3e-08	2.6e-05	31.2
Lipid Metabolism	LDL	rs185319288	19	23923635	A	G	-0.112	0.019	2.4e-09	4.8e-05	35.6
Lipid Metabolism	LDL	rs147791730	19	33864260	A	G	-0.029	0.004	3.3e-12	3.9e-05	48.5
Lipid Metabolism	LDL	rs8102391	19	35674420	A	G	-0.010	0.002	3.3e-10	3.6e-05	39.5
Lipid Metabolism	LDL	rs4805129	19	36019522	C	T	-0.009	0.001	7.2e-10	3.1e-05	38.0
Lipid Metabolism	LDL	rs7257190	19	38392049	C	T	-0.009	0.001	2.2e-10	3.3e-05	40.3
Lipid Metabolism	LDL	rs10405633	19	41291703	A	G	-0.012	0.002	5.9e-11	3.5e-05	42.8
Lipid Metabolism	LDL	rs56113850	19	41353107	C	T	0.014	0.001	1.7e-21	7.9e-05	90.7
Lipid Metabolism	LDL	rs35538872	19	41754430	A	G	-0.065	0.009	6.9e-12	3.8e-05	47.1
Lipid Metabolism	LDL	rs565114573	19	42232879	A	C	-0.319	0.049	8.7e-11	8.9e-05	42.1
Lipid Metabolism	LDL	rs575109902	19	42357175	G	A	-0.083	0.014	6.2e-09	2.8e-05	33.8
Lipid Metabolism	LDL	rs567930445	19	43286858	C	T	-0.323	0.048	2.1e-11	9.7e-05	44.9
Lipid Metabolism	LDL	rs1064919	19	43579627	A	G	-0.314	0.047	1.8e-11	9.9e-05	45.2
Lipid Metabolism	LDL	rs547268356	19	43757939	C	T	-0.494	0.079	3.5e-10	4.5e-04	39.4
Lipid Metabolism	LDL	rs184626726	19	43823028	C	A	-0.198	0.033	3.1e-09	3.8e-05	35.1
Lipid Metabolism	LDL	rs186752767	19	44001983	A	G	-0.206	0.036	1.4e-08	4.2e-05	32.2
Lipid Metabolism	LDL	rs571370258	19	44042739	G	A	-0.247	0.033	3.7e-14	5.6e-05	57.3
Lipid Metabolism	LDL	rs531814632	19	44083959	T	C	-0.339	0.061	2.4e-08	3.9e-04	31.2
Lipid Metabolism	LDL	rs145149776	19	44179442	G	C	-0.073	0.010	3.0e-13	4.5e-05	53.2
Lipid Metabolism	LDL	rs186659901	19	44234411	A	G	-0.230	0.030	1.3e-14	1.5e-04	59.4
Lipid Metabolism	LDL	rs369665838	19	44241404	A	G	0.050	0.009	4.5e-08	2.5e-05	29.9
Lipid Metabolism	LDL	rs180939415	19	44326213	T	C	-0.399	0.068	5.2e-09	6.3e-05	34.1
Lipid Metabolism	LDL	rs73050566	19	44330642	A	T	-0.082	0.010	3.2e-17	7.2e-05	71.2
Lipid Metabolism	LDL	rs142669812	19	44440096	G	A	-0.233	0.032	4.0e-13	6.4e-05	52.7
Lipid Metabolism	LDL	rs558290612	19	44548377	A	G	-0.132	0.017	3.9e-14	6.9e-05	57.2
Lipid Metabolism	LDL	rs373550392	19	44592355	T	C	-0.463	0.080	7.8e-09	5.3e-05	33.3
Lipid Metabolism	LDL	rs530734271	19	44760914	C	T	-0.451	0.078	6.3e-09	8.4e-05	33.7
Lipid Metabolism	LDL	rs74796695	19	44805948	G	T	-0.092	0.006	1.3e-51	1.9e-04	228.4
Lipid Metabolism	LDL	rs139777621	19	44832662	T	C	-0.582	0.101	8.4e-09	5.3e-05	33.2

Phenotype	Trait	SNP	Chr	Position (GRCh37/hg19)	EA	NEA	BETA	SE	P-value	R2	F
Lipid Metabolism	LDL	rs138181035	19	44863163	T	C	-0.187	0.012	1.9e-56	2.1e-04	250.7
Lipid Metabolism	LDL	rs189484322	19	44877060	A	G	-0.103	0.014	4.3e-14	5.0e-05	57.0
Lipid Metabolism	LDL	rs182015576	19	44931746	T	C	-0.273	0.046	4.3e-09	5.5e-05	34.5
Lipid Metabolism	LDL	rs562852949	19	45047467	A	G	-0.275	0.024	1.2e-29	3.2e-04	127.8
Lipid Metabolism	LDL	rs568756641	19	45059854	T	C	0.119	0.019	2.8e-10	4.9e-05	39.8
Lipid Metabolism	LDL	rs186513223	19	45081310	A	T	-0.408	0.035	1.4e-31	2.0e-04	136.7
Lipid Metabolism	LDL	rs576293107	19	45099023	T	A	-0.448	0.080	2.1e-08	6.8e-05	31.4
Lipid Metabolism	LDL	rs142246818	19	45123354	A	G	0.283	0.033	4.5e-18	1.1e-04	75.1
Lipid Metabolism	LDL	rs55791016	19	45124455	T	C	-0.175	0.012	1.0e-49	2.6e-04	219.8
Lipid Metabolism	LDL	rs150611785	19	45131439	T	C	-0.600	0.089	1.4e-11	1.1e-04	45.7
Lipid Metabolism	LDL	rs62119267	19	45134682	C	A	-0.225	0.005	1.0e-200	1.7e-03	2082.2
Lipid Metabolism	LDL	rs550835657	19	45148926	T	C	-0.336	0.033	1.7e-24	1.5e-04	104.4
Lipid Metabolism	LDL	rs201098299	19	45176115	T	C	-0.483	0.070	6.5e-12	1.0e-04	47.2
Lipid Metabolism	LDL	rs550480522	19	45180107	T	C	-0.443	0.046	9.5e-22	1.8e-04	91.8
Lipid Metabolism	LDL	rs190579316	19	45180335	A	G	0.216	0.037	5.3e-09	3.8e-05	34.1
Lipid Metabolism	LDL	rs151330717	19	45196964	A	G	-0.301	0.006	1.0e-200	2.1e-03	2624.0
Lipid Metabolism	LDL	rs567541985	19	45201347	A	G	-0.149	0.023	5.1e-11	5.7e-05	43.2
Lipid Metabolism	LDL	rs550621052	19	45207835	A	T	-0.347	0.025	1.3e-44	1.9e-04	196.4
Lipid Metabolism	LDL	rs139163794	19	45221641	C	T	0.586	0.107	4.4e-08	7.6e-05	29.9
Lipid Metabolism	LDL	rs551091979	19	45232725	A	G	0.176	0.027	3.2e-11	4.1e-05	44.0
Lipid Metabolism	LDL	rs574608353	19	45248568	T	C	0.154	0.021	4.1e-13	5.2e-05	52.6
Lipid Metabolism	LDL	rs2965169	19	45251156	C	A	-0.075	0.001	1.0e-200	2.1e-03	2577.4
Lipid Metabolism	LDL	rs573007449	19	45263188	T	C	-0.438	0.021	5.1e-100	5.5e-04	450.7
Lipid Metabolism	LDL	rs568828389	19	45265764	T	C	0.253	0.038	1.7e-11	1.1e-04	45.3
Lipid Metabolism	LDL	rs548150833	19	45274462	A	G	0.207	0.024	1.6e-17	8.9e-05	72.6
Lipid Metabolism	LDL	rs529813323	19	45300470	A	G	0.184	0.019	4.2e-22	2.3e-04	93.4
Lipid Metabolism	LDL	rs547570291	19	45308756	A	G	-0.552	0.021	6.5e-147	8.2e-04	666.3
Lipid Metabolism	LDL	rs148501359	19	45315452	T	C	-2.434	0.425	9.9e-09	5.3e-05	32.9
Lipid Metabolism	LDL	rs372019933	19	45316423	A	C	0.160	0.019	8.3e-18	9.5e-05	73.9
Lipid Metabolism	LDL	rs180887453	19	45318495	G	A	0.206	0.014	2.2e-50	2.3e-04	222.8
Lipid Metabolism	LDL	rs529877444	19	45328216	G	A	-0.724	0.058	1.4e-35	3.9e-04	155.0
Lipid Metabolism	LDL	rs143433171	19	45329418	A	G	0.506	0.081	4.1e-10	7.8e-05	39.1
Lipid Metabolism	LDL	rs547509922	19	45331261	T	C	0.245	0.013	9.5e-77	2.9e-04	343.8
Lipid Metabolism	LDL	rs3021439	19	45336443	A	G	0.083	0.002	1.0e-200	2.5e-03	3033.1
Lipid Metabolism	LDL	rs189409600	19	45341066	T	C	0.195	0.020	9.9e-23	1.1e-04	96.3
Lipid Metabolism	LDL	rs547934469	19	45364230	T	C	0.370	0.047	4.1e-15	1.1e-04	61.6
Lipid Metabolism	LDL	rs182824418	19	45370451	T	G	0.144	0.013	2.0e-27	1.1e-04	117.7
Lipid Metabolism	LDL	rs112484236	19	45376893	C	A	0.207	0.012	1.6e-66	3.3e-04	296.9
Lipid Metabolism	LDL	rs535134554	19	45388444	T	G	-1.020	0.163	3.5e-10	6.4e-05	39.4
Lipid Metabolism	LDL	rs2075650	19	45395619	G	A	0.161	0.002	1.0e-200	5.4e-03	6646.4
Lipid Metabolism	LDL	rs545127526	19	45396772	C	G	-0.917	0.105	2.3e-18	1.9e-04	76.4
Lipid Metabolism	LDL	rs542944951	19	45402486	T	C	0.355	0.052	8.1e-12	8.7e-05	46.7
Lipid Metabolism	LDL	rs373985746	19	45409136	A	G	0.099	0.015	9.9e-11	1.1e-04	41.8
Lipid Metabolism	LDL	rs2007708	19	45410420	A	G	-1.248	0.113	2.2e-28	3.0e-04	122.1
Lipid Metabolism	LDL	rs146404925	19	45416270	C	T	-0.583	0.086	8.9e-12	6.9e-05	46.5
Lipid Metabolism	LDL	rs187408586	19	45422921	G	A	-0.577	0.017	1.0e-200	2.8e-03	1165.7
Lipid Metabolism	LDL	rs545076529	19	45424988	T	C	0.821	0.121	1.0e-11	1.1e-04	46.2
Lipid Metabolism	LDL	rs182067414	19	45425954	C	T	-0.547	0.013	1.0e-200	1.4e-03	1688.7
Lipid Metabolism	LDL	rs550063874	19	45426283	G	C	0.897	0.164	4.8e-08	7.6e-05	29.8
Lipid Metabolism	LDL	rs565334527	19	45428674	C	T	0.251	0.039	8.1e-11	6.3e-05	42.2
Lipid Metabolism	LDL	rs529060034	19	45430476	G	C	0.124	0.019	1.8e-10	1.0e-04	40.7
Lipid Metabolism	LDL	rs541817716	19	45433531	A	T	0.337	0.029	3.4e-32	1.5e-04	139.5
Lipid Metabolism	LDL	rs371390156	19	45444802	A	G	-0.486	0.030	9.8e-61	4.6e-04	270.3
Lipid Metabolism	LDL	rs532259779	19	45445838	T	C	-0.884	0.148	2.6e-09	8.9e-05	35.5
Lipid Metabolism	LDL	rs547358144	19	45446430	A	G	-0.551	0.080	7.6e-12	9.3e-05	46.9
Lipid Metabolism	LDL	rs182695610	19	45450733	C	G	-0.826	0.120	6.3e-12	1.2e-04	47.2

Phenotype	Trait	SNP	Chr	Position (GRCh37/hg19)	EA	NEA	BETA	SE	P-value	R2	F
Lipid Metabolism	LDL	rs190651665	19	45456103	G	T	-0.057	0.004	7.5e-42	1.5e-04	183.7
Lipid Metabolism	LDL	rs117198034	19	45469902	T	C	-0.041	0.005	5.2e-15	5.1e-05	61.2
Lipid Metabolism	LDL	rs148947391	19	45483674	A	G	0.075	0.008	4.4e-21	7.3e-05	88.8
Lipid Metabolism	LDL	rs574070471	19	45487040	G	A	-0.340	0.008	1.0e-200	1.6e-03	1955.4
Lipid Metabolism	LDL	rs150484293	19	45496095	T	C	0.323	0.020	1.6e-59	2.9e-04	264.8
Lipid Metabolism	LDL	rs537956257	19	45497376	T	C	0.300	0.035	5.5e-18	3.8e-04	74.7
Lipid Metabolism	LDL	rs188820899	19	45499042	A	G	0.072	0.013	2.6e-08	3.0e-05	31.0
Lipid Metabolism	LDL	rs560250454	19	45508045	C	A	-0.510	0.036	2.0e-45	3.1e-04	200.0
Lipid Metabolism	LDL	rs117413065	19	45511784	A	G	0.183	0.011	2.4e-67	2.5e-04	300.6
Lipid Metabolism	LDL	rs74359223	19	45522289	A	G	0.107	0.007	2.9e-52	1.9e-04	231.4
Lipid Metabolism	LDL	rs188161988	19	45523453	G	A	0.156	0.020	1.2e-14	7.3e-05	59.6
Lipid Metabolism	LDL	rs114627037	19	45524364	A	G	0.078	0.008	5.2e-21	7.3e-05	88.4
Lipid Metabolism	LDL	rs553887842	19	45529797	A	G	0.158	0.018	9.0e-19	9.8e-05	78.3
Lipid Metabolism	LDL	rs529650868	19	45541168	G	A	-0.640	0.077	6.8e-17	1.2e-04	69.7
Lipid Metabolism	LDL	rs183321458	19	45581076	A	G	0.068	0.012	1.6e-08	2.9e-05	31.9
Lipid Metabolism	LDL	rs575180058	19	45599213	T	C	-0.616	0.078	2.6e-15	9.7e-05	62.5
Lipid Metabolism	LDL	rs145208519	19	45612729	T	C	0.060	0.005	1.6e-39	1.4e-04	173.0
Lipid Metabolism	LDL	rs551901850	19	45620234	T	C	-0.364	0.018	9.1e-89	3.5e-04	399.0
Lipid Metabolism	LDL	rs565386331	19	45645328	T	C	-0.479	0.075	1.7e-10	7.6e-05	40.8
Lipid Metabolism	LDL	rs550905135	19	45660421	T	C	-0.436	0.051	1.1e-17	1.8e-04	73.3
Lipid Metabolism	LDL	rs552359704	19	45694254	A	G	-0.038	0.006	2.2e-09	2.9e-05	35.8
Lipid Metabolism	LDL	rs552314144	19	45706447	G	A	-0.292	0.048	1.3e-09	5.8e-05	36.8
Lipid Metabolism	LDL	rs535470529	19	45732956	G	A	-0.449	0.029	5.2e-53	3.2e-04	234.9
Lipid Metabolism	LDL	rs539517471	19	45741168	A	G	0.086	0.014	7.9e-10	4.5e-05	37.8
Lipid Metabolism	LDL	rs112202258	19	45766237	A	G	0.139	0.023	1.3e-09	3.7e-05	36.8
Lipid Metabolism	LDL	rs577988419	19	45790310	A	G	-0.323	0.049	2.9e-11	5.8e-05	44.2
Lipid Metabolism	LDL	rs191846322	19	45821856	T	C	-0.371	0.055	2.0e-11	1.0e-04	44.9
Lipid Metabolism	LDL	rs561239872	19	45822724	A	G	-0.407	0.070	6.7e-09	7.3e-05	33.6
Lipid Metabolism	LDL	rs527685951	19	45835305	A	G	-0.274	0.028	2.3e-22	2.4e-04	94.6
Lipid Metabolism	LDL	rs113733462	19	45840488	C	T	0.157	0.025	2.2e-10	5.5e-05	40.3
Lipid Metabolism	LDL	rs3916805	19	45870452	A	G	-0.605	0.089	9.8e-12	6.1e-05	46.4
Lipid Metabolism	LDL	rs181765708	19	45880690	T	C	-0.174	0.005	1.0e-200	8.4e-04	1032.9
Lipid Metabolism	LDL	rs560577742	19	45893023	G	T	-1.231	0.144	1.5e-17	1.2e-04	72.8
Lipid Metabolism	LDL	rs148956143	19	45934658	T	C	-0.380	0.060	1.7e-10	8.5e-05	40.8
Lipid Metabolism	LDL	rs566147398	19	46123098	T	C	0.219	0.040	4.0e-08	3.2e-05	30.2
Lipid Metabolism	LDL	rs538473976	19	46173575	T	C	-0.052	0.006	4.4e-18	6.1e-05	75.2
Lipid Metabolism	LDL	rs543235981	19	46197099	A	G	0.493	0.075	5.5e-11	1.1e-04	43.0
Lipid Metabolism	LDL	rs578060652	19	46366299	T	C	-0.356	0.051	2.5e-12	1.2e-04	49.1
Lipid Metabolism	LDL	rs8112972	19	46390605	A	G	-0.028	0.002	1.4e-39	1.4e-04	173.4
Lipid Metabolism	LDL	rs145051273	19	46423738	T	C	0.198	0.035	2.2e-08	3.7e-05	31.3
Lipid Metabolism	LDL	rs547104645	19	46440323	G	C	-0.412	0.050	1.2e-16	1.2e-04	68.6
Lipid Metabolism	LDL	rs151126738	19	46480373	G	A	-0.574	0.094	1.0e-09	4.8e-05	37.3
Lipid Metabolism	LDL	rs182662731	19	46483340	C	T	0.092	0.017	3.5e-08	3.8e-05	30.4
Lipid Metabolism	LDL	rs560342930	19	46493356	T	C	-0.596	0.099	1.6e-09	5.2e-04	36.5
Lipid Metabolism	LDL	rs576210968	19	46511827	T	C	-0.156	0.019	1.2e-15	6.2e-05	64.1
Lipid Metabolism	LDL	rs554272516	19	46587878	G	A	-0.781	0.128	1.1e-09	1.6e-04	37.1
Lipid Metabolism	LDL	rs180885509	19	46612398	G	C	-0.107	0.016	7.7e-12	4.1e-05	46.8
Lipid Metabolism	LDL	rs546424021	19	46707108	T	G	-0.128	0.023	1.5e-08	3.1e-05	32.1
Lipid Metabolism	LDL	rs759290	19	46892111	T	C	-0.010	0.002	1.9e-09	2.9e-05	36.0
Lipid Metabolism	LDL	rs141229555	19	46940767	G	A	-0.298	0.047	1.7e-10	5.0e-05	40.8
Lipid Metabolism	LDL	rs574347155	19	47210829	A	G	-0.252	0.044	7.5e-09	5.2e-05	33.4
Lipid Metabolism	LDL	rs443081	19	47212343	T	C	0.014	0.002	3.3e-12	3.9e-05	48.5
Lipid Metabolism	LDL	rs56011104	19	48092978	T	C	-0.301	0.054	2.0e-08	4.1e-05	31.5
Lipid Metabolism	LDL	rs4801770	19	49129791	G	A	-0.021	0.002	4.1e-21	7.3e-05	88.9
Lipid Metabolism	LDL	rs601338	19	49206674	A	G	0.029	0.001	1.2e-97	3.6e-04	439.7
Lipid Metabolism	LDL	rs184817754	19	50144571	G	A	-0.052	0.007	2.5e-12	4.0e-05	49.0

Phenotype	Trait	SNP	Chr	Position (GRCh37/hg19)	EA	NEA	BETA	SE	P-value	R2	F
Lipid Metabolism	LDL	rs4806498	19	54674742	T	C	0.008	0.001	6.4e-09	2.8e-05	33.7
Lipid Metabolism	LDL	rs2189699	19	57031408	A	G	-0.012	0.002	5.7e-15	5.0e-05	61.0
Lipid Metabolism	LDL	rs145467679	19	58308789	C	T	0.061	0.008	8.3e-15	5.0e-05	60.3
Lipid Metabolism	LDL	rs144794875	19	58391476	G	A	-0.018	0.002	1.5e-14	5.6e-05	59.1
Lipid Metabolism	LDL	rs35081008	19	58662235	T	C	-0.031	0.002	1.6e-57	2.1e-04	255.5
Lipid Metabolism	LDL	rs1867727	19	59084181	C	G	-0.012	0.002	4.5e-15	5.0e-05	61.5
Lipid Metabolism	LDL	rs6051908	20	403653	G	T	0.009	0.002	8.1e-10	3.1e-05	37.7
Lipid Metabolism	LDL	rs66879753	20	3312625	A	C	0.011	0.002	2.2e-08	2.5e-05	31.3
Lipid Metabolism	LDL	rs11699903	20	4019036	A	G	0.012	0.002	1.9e-08	2.6e-05	31.6
Lipid Metabolism	LDL	rs55649827	20	5508885	A	T	-0.013	0.002	9.5e-12	3.8e-05	46.4
Lipid Metabolism	LDL	rs73075609	20	5580789	T	C	0.048	0.005	6.5e-24	8.3e-05	101.7
Lipid Metabolism	LDL	rs438568	20	12958687	G	A	0.014	0.001	1.7e-21	7.4e-05	90.7
Lipid Metabolism	LDL	rs137981542	20	17617260	A	G	-0.175	0.030	6.2e-09	3.7e-05	33.8
Lipid Metabolism	LDL	rs6131987	20	17670847	T	C	0.012	0.002	1.0e-11	3.8e-05	46.3
Lipid Metabolism	LDL	rs117077869	20	17731736	C	T	0.056	0.008	2.5e-12	4.0e-05	49.0
Lipid Metabolism	LDL	rs6111702	20	17830903	T	C	0.031	0.004	5.1e-14	4.6e-05	56.7
Lipid Metabolism	LDL	rs2618568	20	17843968	A	C	-0.038	0.001	1.2e-141	5.5e-04	642.0
Lipid Metabolism	LDL	rs3746337	20	25208272	T	C	0.010	0.001	4.2e-13	4.3e-05	52.6
Lipid Metabolism	LDL	rs7261820	20	34160840	A	G	-0.030	0.002	1.2e-51	1.9e-04	228.7
Lipid Metabolism	LDL	rs115989692	20	34739488	G	T	-0.027	0.004	1.0e-12	4.1e-05	50.8
Lipid Metabolism	LDL	rs56202412	20	39119602	A	T	-0.025	0.003	6.3e-20	6.8e-05	83.5
Lipid Metabolism	LDL	rs1883711	20	39179822	C	G	0.131	0.004	1.0e-200	8.0e-04	981.5
Lipid Metabolism	LDL	rs2865509	20	39239797	C	T	-0.019	0.001	3.0e-37	1.3e-04	162.6
Lipid Metabolism	LDL	rs6072195	20	39560474	T	C	0.010	0.002	8.1e-09	2.7e-05	33.3
Lipid Metabolism	LDL	rs6029549	20	39754695	G	C	0.030	0.001	1.1e-104	3.8e-04	472.2
Lipid Metabolism	LDL	rs56275254	20	40253910	G	A	0.025	0.003	9.8e-16	5.2e-05	64.5
Lipid Metabolism	LDL	rs12480046	20	40305932	T	C	0.011	0.002	2.3e-11	3.6e-05	44.7
Lipid Metabolism	LDL	rs75564175	20	40317984	T	G	0.034	0.006	4.8e-08	2.4e-05	29.8
Lipid Metabolism	LDL	rs1800961	20	43042364	T	C	-0.055	0.004	3.3e-46	1.7e-04	203.7
Lipid Metabolism	LDL	rs6104410	20	44607601	G	A	-0.012	0.002	1.2e-13	4.5e-05	55.0
Lipid Metabolism	LDL	rs1569723	20	44742064	A	C	0.011	0.002	4.2e-12	3.9e-05	48.0
Lipid Metabolism	LDL	rs2295027	20	47582884	A	G	0.012	0.002	3.5e-14	4.7e-05	57.5
Lipid Metabolism	LDL	rs6063965	20	52191200	A	G	0.013	0.002	7.4e-10	3.1e-05	37.9
Lipid Metabolism	LDL	rs6022849	20	52532637	C	G	0.011	0.001	5.8e-14	4.7e-05	56.4
Lipid Metabolism	LDL	rs3746778	20	61341472	A	G	-0.013	0.002	2.4e-16	6.0e-05	67.2
Lipid Metabolism	LDL	rs932826	20	62380516	C	A	0.009	0.001	2.2e-09	3.0e-05	35.8
Lipid Metabolism	LDL	rs8126001	20	62711459	T	C	-0.014	0.001	1.8e-21	8.7e-05	90.5
Lipid Metabolism	LDL	rs116843256	20	62917667	T	C	0.016	0.002	5.1e-14	5.1e-05	56.7
Lipid Metabolism	LDL	rs12106385	21	16586682	A	T	-0.036	0.006	3.9e-11	3.5e-05	43.6
Lipid Metabolism	LDL	rs2833487	21	33087863	G	A	0.031	0.003	1.1e-22	7.8e-05	96.1
Lipid Metabolism	LDL	rs11911615	21	37553132	G	T	-0.008	0.001	5.0e-08	2.4e-05	29.7
Lipid Metabolism	LDL	rs1963676	21	40709960	T	C	0.014	0.001	8.9e-24	8.3e-05	101.1
Lipid Metabolism	LDL	rs11703382	22	18481857	T	C	0.012	0.002	4.2e-14	4.7e-05	57.1
Lipid Metabolism	LDL	rs165722	22	19949013	T	C	0.009	0.001	8.2e-11	3.5e-05	42.2
Lipid Metabolism	LDL	rs5754102	22	21916272	A	C	-0.015	0.002	4.8e-14	5.3e-05	56.8
Lipid Metabolism	LDL	rs5752963	22	30203833	A	G	0.027	0.004	2.5e-13	4.4e-05	53.6
Lipid Metabolism	LDL	rs117147052	22	32411817	C	T	0.020	0.003	1.1e-09	3.0e-05	37.2
Lipid Metabolism	LDL	rs2235145	22	35654133	G	T	-0.014	0.001	6.9e-21	7.2e-05	87.9
Lipid Metabolism	LDL	rs138352	22	41268925	G	T	-0.014	0.001	9.5e-21	7.1e-05	87.3
Lipid Metabolism	LDL	rs4823173	22	44328730	A	G	-0.014	0.002	2.1e-13	4.4e-05	53.9
Lipid Metabolism	LDL	rs5764726	22	45826033	G	A	-0.010	0.002	2.4e-10	3.3e-05	40.1
Lipid Metabolism	LDL	rs13268	22	45996298	G	A	-0.038	0.005	4.8e-17	5.8e-05	70.4
Lipid Metabolism	LDL	rs4253766	22	46623905	T	C	0.014	0.002	8.5e-10	3.1e-05	37.6
Lipid Metabolism	LDL	rs1042311	22	46627780	T	C	0.072	0.010	8.0e-13	4.4e-05	51.3
Lipid Metabolism	LDL	rs7410608	22	50858813	C	G	0.011	0.001	4.9e-15	5.0e-05	61.3
Lipid Metabolism	TC	rs262679	1	2169792	T	C	-0.008	0.001	4.8e-09	2.6e-05	34.3

Phenotype	Trait	SNP	Chr	Position (GRCh37/hg19)	EA	NEA	BETA	SE	P-value	R2	F
Lipid Metabolism	TC	rs622623	1	10555257	T	C	0.009	0.001	4.5e-10	3.0e-05	38.9
Lipid Metabolism	TC	rs12046278	1	10799577	C	T	-0.011	0.001	1.0e-13	4.3e-05	55.3
Lipid Metabolism	TC	rs35365738	1	16068364	A	G	-0.009	0.001	1.7e-10	3.1e-05	40.8
Lipid Metabolism	TC	rs924204	1	16513926	G	A	0.014	0.001	4.3e-23	7.5e-05	98.0
Lipid Metabolism	TC	rs6704301	1	16688635	G	A	0.009	0.002	3.6e-08	2.7e-05	30.3
Lipid Metabolism	TC	rs1360916	1	18801697	A	G	-0.009	0.001	9.7e-11	3.2e-05	41.9
Lipid Metabolism	TC	rs1200993	1	23472048	T	C	0.014	0.002	1.4e-14	4.5e-05	59.2
Lipid Metabolism	TC	rs2297841	1	23764149	G	C	0.024	0.002	3.1e-36	1.2e-04	158.0
Lipid Metabolism	TC	rs35345536	1	25766488	G	A	0.026	0.001	5.2e-83	2.8e-04	372.5
Lipid Metabolism	TC	rs56214942	1	25891959	A	G	0.010	0.001	2.2e-14	4.5e-05	58.4
Lipid Metabolism	TC	rs55820521	1	26374286	T	C	-0.010	0.002	4.3e-10	3.0e-05	39.0
Lipid Metabolism	TC	rs12096570	1	26852707	C	T	0.011	0.002	1.2e-11	3.5e-05	46.0
Lipid Metabolism	TC	rs114165349	1	27021913	C	G	0.060	0.004	1.2e-41	1.4e-04	182.8
Lipid Metabolism	TC	rs2815708	1	28347491	T	C	-0.011	0.001	1.9e-14	4.5e-05	58.6
Lipid Metabolism	TC	rs2483719	1	29468198	T	C	-0.009	0.002	2.2e-08	2.4e-05	31.4
Lipid Metabolism	TC	rs12040626	1	33449184	A	C	-0.008	0.001	2.4e-08	2.4e-05	31.1
Lipid Metabolism	TC	rs4329504	1	39451488	G	A	-0.010	0.001	3.1e-12	3.7e-05	48.6
Lipid Metabolism	TC	rs140723719	1	45306595	A	G	-0.020	0.004	2.8e-08	2.3e-05	30.9
Lipid Metabolism	TC	rs17853159	1	45810865	A	G	-0.018	0.002	2.2e-13	4.1e-05	53.8
Lipid Metabolism	TC	rs12407052	1	46512588	C	T	-0.025	0.004	1.1e-10	3.2e-05	41.7
Lipid Metabolism	TC	rs72677557	1	46602345	C	T	0.010	0.002	1.4e-08	2.4e-05	32.2
Lipid Metabolism	TC	rs547856250	1	49263479	A	G	-0.114	0.019	1.4e-09	5.3e-05	36.7
Lipid Metabolism	TC	rs185716614	1	51068058	C	A	-0.135	0.018	2.0e-14	7.9e-05	58.6
Lipid Metabolism	TC	rs570553136	1	52262405	G	A	-0.157	0.018	1.3e-17	1.1e-04	73.0
Lipid Metabolism	TC	rs191875604	1	54246772	C	T	-0.168	0.017	1.5e-23	1.0e-04	100.0
Lipid Metabolism	TC	rs563391624	1	54748004	T	C	-1.073	0.115	1.2e-20	1.9e-04	86.8
Lipid Metabolism	TC	rs188400795	1	55175165	G	A	-0.180	0.026	6.4e-12	4.5e-05	47.2
Lipid Metabolism	TC	rs192958267	1	55218101	T	C	-0.550	0.055	2.2e-23	2.5e-04	99.3
Lipid Metabolism	TC	rs555953510	1	55241563	A	G	-0.129	0.016	6.8e-16	5.2e-05	65.2
Lipid Metabolism	TC	rs564277757	1	55387294	A	G	-0.472	0.079	2.5e-09	6.2e-05	35.6
Lipid Metabolism	TC	rs561554246	1	55453057	G	A	-0.381	0.051	1.2e-13	1.3e-04	55.0
Lipid Metabolism	TC	rs183549858	1	55465741	A	C	0.056	0.009	3.5e-09	2.7e-05	34.9
Lipid Metabolism	TC	rs79204901	1	55487489	T	C	0.040	0.004	1.9e-24	8.0e-05	104.1
Lipid Metabolism	TC	rs2479409	1	55504650	A	G	-0.038	0.001	1.1e-158	5.6e-04	720.3
Lipid Metabolism	TC	rs148195424	1	55518374	T	C	-0.466	0.041	2.0e-30	1.8e-04	131.4
Lipid Metabolism	TC	rs539259897	1	55529944	C	G	-0.546	0.100	4.3e-08	7.5e-05	30.0
Lipid Metabolism	TC	rs28385715	1	55532142	G	T	0.037	0.005	4.2e-15	4.8e-05	61.6
Lipid Metabolism	TC	rs145372536	1	55532710	A	G	0.109	0.014	1.7e-14	4.6e-05	58.9
Lipid Metabolism	TC	rs138856381	1	55542694	T	C	-0.055	0.008	9.5e-13	3.9e-05	50.9
Lipid Metabolism	TC	rs77333170	1	55553210	T	A	-0.032	0.004	2.5e-18	5.8e-05	76.2
Lipid Metabolism	TC	rs181149007	1	55562928	T	G	-0.475	0.080	3.3e-09	8.6e-05	35.0
Lipid Metabolism	TC	rs115465289	1	55580914	A	G	-0.032	0.004	1.6e-15	4.8e-05	63.5
Lipid Metabolism	TC	rs191448950	1	55584844	A	G	-0.361	0.008	1.0e-200	1.6e-03	2111.6
Lipid Metabolism	TC	rs553629879	1	55602330	C	T	0.116	0.015	2.8e-15	4.9e-05	62.4
Lipid Metabolism	TC	rs1475701	1	55638546	C	T	0.076	0.004	4.2e-99	3.4e-04	446.5
Lipid Metabolism	TC	rs12750160	1	55674000	T	C	-0.260	0.008	1.0e-200	9.1e-04	1186.7
Lipid Metabolism	TC	rs114412656	1	55718442	T	C	-0.034	0.005	1.4e-10	3.1e-05	41.2
Lipid Metabolism	TC	rs143583677	1	55724341	A	G	0.055	0.008	1.6e-11	3.5e-05	45.4
Lipid Metabolism	TC	rs115449167	1	55780468	C	A	0.036	0.005	8.8e-15	4.6e-05	60.1
Lipid Metabolism	TC	rs183378272	1	55881468	C	T	-0.449	0.077	5.2e-09	8.4e-05	34.1
Lipid Metabolism	TC	rs142027040	1	55938051	G	A	-0.940	0.070	1.2e-40	4.5e-04	178.2
Lipid Metabolism	TC	rs570729995	1	56196706	G	A	-0.255	0.037	8.6e-12	5.3e-05	46.6
Lipid Metabolism	TC	rs558153898	1	56574672	A	G	-0.364	0.056	5.3e-11	7.2e-05	43.1
Lipid Metabolism	TC	rs547260049	1	56599040	G	A	-0.219	0.013	2.4e-65	2.3e-04	291.5
Lipid Metabolism	TC	rs72675550	1	56603817	A	G	0.029	0.005	2.5e-08	2.4e-05	31.1
Lipid Metabolism	TC	rs565120608	1	56675202	A	G	-0.172	0.027	1.9e-10	4.4e-05	40.5

Phenotype	Trait	SNP	Chr	Position (GRCh37/hg19)	EA	NEA	BETA	SE	P-value	R2	F
Lipid Metabolism	TC	rs550894946	1	56733065	T	A	-0.117	0.019	1.1e-09	3.4e-05	37.1
Lipid Metabolism	TC	rs186538116	1	56840574	C	A	-0.308	0.019	7.4e-60	3.5e-04	266.3
Lipid Metabolism	TC	rs186907611	1	56995693	G	A	-0.273	0.021	4.0e-40	2.5e-04	175.8
Lipid Metabolism	TC	rs78519632	1	57028940	T	C	-0.041	0.006	1.9e-10	3.1e-05	40.6
Lipid Metabolism	TC	rs185172024	1	57872099	C	T	-0.119	0.018	5.6e-11	4.1e-05	43.0
Lipid Metabolism	TC	rs11806791	1	61684868	C	A	-0.018	0.003	7.5e-13	3.9e-05	51.4
Lipid Metabolism	TC	rs187240865	1	62859760	G	A	-0.085	0.012	5.1e-13	4.1e-05	52.2
Lipid Metabolism	TC	rs626787	1	62901243	G	C	-0.066	0.001	1.0e-200	1.6e-03	2070.0
Lipid Metabolism	TC	rs55763644	1	62961001	A	G	-0.569	0.073	5.6e-15	1.2e-04	61.0
Lipid Metabolism	TC	rs554472916	1	63011663	A	G	-0.053	0.008	9.9e-13	3.9e-05	50.9
Lipid Metabolism	TC	rs115210273	1	63205553	T	G	-0.015	0.002	3.3e-14	4.4e-05	57.6
Lipid Metabolism	TC	rs559647195	1	63352650	T	C	-0.226	0.032	1.9e-12	1.2e-04	49.6
Lipid Metabolism	TC	rs185691644	1	63359330	G	A	-0.067	0.012	4.1e-08	2.4e-05	30.1
Lipid Metabolism	TC	rs186955977	1	63381339	G	A	-0.179	0.025	3.4e-13	5.6e-05	53.0
Lipid Metabolism	TC	rs549777175	1	63413521	A	G	-0.315	0.028	7.0e-30	1.5e-04	128.9
Lipid Metabolism	TC	rs565739460	1	63415321	C	T	-0.598	0.089	1.8e-11	1.1e-04	45.2
Lipid Metabolism	TC	rs185873223	1	63426178	C	T	-1.176	0.150	5.2e-15	1.5e-04	61.2
Lipid Metabolism	TC	rs541438139	1	63824464	C	T	-0.647	0.106	1.1e-09	8.3e-05	37.2
Lipid Metabolism	TC	rs6697515	1	66111766	G	A	0.009	0.002	1.1e-09	3.2e-05	37.1
Lipid Metabolism	TC	rs165316	1	91533297	G	A	-0.016	0.002	1.1e-21	6.9e-05	91.4
Lipid Metabolism	TC	rs962222	1	92970953	T	C	0.024	0.002	1.0e-45	1.5e-04	201.4
Lipid Metabolism	TC	rs151257922	1	93067218	G	C	0.059	0.008	8.7e-15	4.6e-05	60.2
Lipid Metabolism	TC	rs149607479	1	94087611	G	T	0.057	0.009	1.7e-09	2.8e-05	36.3
Lipid Metabolism	TC	rs71654413	1	95607088	T	A	-0.014	0.002	9.0e-09	2.5e-05	33.1
Lipid Metabolism	TC	rs34364548	1	109714635	T	C	0.026	0.004	2.1e-11	3.4e-05	44.8
Lipid Metabolism	TC	rs576654087	1	109719983	A	G	-0.069	0.009	1.1e-15	8.0e-05	64.2
Lipid Metabolism	TC	rs1815307	1	109724824	T	C	-0.083	0.008	2.4e-26	8.7e-05	112.8
Lipid Metabolism	TC	rs35271870	1	109776285	C	T	-0.097	0.002	1.0e-200	1.2e-03	1612.2
Lipid Metabolism	TC	rs575507604	1	109785599	T	C	-0.152	0.021	1.7e-13	5.4e-05	54.3
Lipid Metabolism	TC	rs149841512	1	109796865	A	G	0.036	0.005	4.6e-14	4.3e-05	56.9
Lipid Metabolism	TC	rs6680227	1	109798914	A	G	-0.071	0.004	1.0e-83	2.9e-04	375.8
Lipid Metabolism	TC	rs78852738	1	109800086	C	A	0.042	0.005	8.3e-20	6.3e-05	83.0
Lipid Metabolism	TC	rs35358959	1	109824250	A	G	-0.090	0.002	1.0e-200	1.0e-03	1348.3
Lipid Metabolism	TC	rs17036094	1	109834940	C	A	-0.090	0.006	4.0e-48	1.6e-04	212.4
Lipid Metabolism	TC	rs72647819	1	109851793	T	C	0.028	0.005	4.7e-09	2.6e-05	34.3
Lipid Metabolism	TC	rs17585355	1	109857815	C	A	0.024	0.003	1.1e-17	5.6e-05	73.3
Lipid Metabolism	TC	rs137872213	1	109916336	G	C	0.033	0.005	1.9e-11	3.5e-05	45.0
Lipid Metabolism	TC	rs116363925	1	109926599	T	G	0.023	0.003	2.8e-14	4.4e-05	57.9
Lipid Metabolism	TC	rs72703257	1	109942924	T	C	0.032	0.005	1.6e-12	3.8e-05	50.0
Lipid Metabolism	TC	rs61797139	1	110006665	T	C	0.030	0.003	1.4e-24	8.2e-05	104.8
Lipid Metabolism	TC	rs146887897	1	110073203	C	G	0.036	0.006	1.2e-08	2.5e-05	32.6
Lipid Metabolism	TC	rs1553450	1	114490780	G	A	-0.010	0.002	1.1e-10	3.1e-05	41.6
Lipid Metabolism	TC	rs6696923	1	118172538	G	A	0.009	0.001	3.7e-10	3.0e-05	39.3
Lipid Metabolism	TC	rs483180	1	120267505	G	C	-0.008	0.001	3.7e-08	2.3e-05	30.3
Lipid Metabolism	TC	rs181473381	1	145472741	A	C	0.109	0.013	1.4e-17	5.8e-05	72.8
Lipid Metabolism	TC	rs185073199	1	145536716	A	T	0.058	0.009	2.7e-10	4.1e-05	39.9
Lipid Metabolism	TC	rs587699996	1	149036350	G	A	-0.434	0.078	2.2e-08	7.9e-05	31.3
Lipid Metabolism	TC	rs267733	1	150958836	G	A	-0.013	0.002	5.0e-13	4.0e-05	52.2
Lipid Metabolism	TC	rs1673161	1	150986662	G	A	0.014	0.003	1.6e-08	2.4e-05	31.9
Lipid Metabolism	TC	rs184980867	1	151658543	A	G	-0.076	0.013	2.6e-09	2.8e-05	35.5
Lipid Metabolism	TC	rs113462784	1	154044453	C	T	0.016	0.003	8.5e-10	3.4e-05	37.6
Lipid Metabolism	TC	rs4390169	1	155106054	G	A	-0.011	0.001	3.4e-15	4.7e-05	62.0
Lipid Metabolism	TC	rs2022003	1	158586966	T	A	0.012	0.001	5.7e-17	5.3e-05	70.1
Lipid Metabolism	TC	rs115383270	1	161531340	A	G	0.025	0.003	7.1e-21	6.9e-05	87.8
Lipid Metabolism	TC	rs12136407	1	161678377	C	T	-0.007	0.001	2.5e-08	2.4e-05	31.0
Lipid Metabolism	TC	rs4656539	1	167357561	G	A	-0.013	0.002	1.6e-09	2.9e-05	36.5

Phenotype	Trait	SNP	Chr	Position (GRCh37/hg19)	EA	NEA	BETA	SE	P-value	R2	F
Lipid Metabolism	TC	rs76900682	1	174064560	A	G	0.011	0.002	1.0e-08	2.5e-05	32.8
Lipid Metabolism	TC	rs6682862	1	177938437	A	G	-0.011	0.002	6.0e-10	2.9e-05	38.3
Lipid Metabolism	TC	rs7531837	1	177980220	C	T	-0.008	0.001	4.8e-08	2.3e-05	29.8
Lipid Metabolism	TC	rs10797843	1	183081995	G	A	0.012	0.001	1.6e-19	6.2e-05	81.6
Lipid Metabolism	TC	rs4639796	1	197126649	A	G	0.011	0.002	9.8e-10	2.8e-05	37.4
Lipid Metabolism	TC	rs1434282	1	199010721	T	C	0.010	0.002	6.7e-12	3.6e-05	47.1
Lipid Metabolism	TC	rs4246550	1	203263044	G	C	-0.008	0.001	9.4e-10	2.9e-05	37.5
Lipid Metabolism	TC	rs7516171	1	210466932	T	C	0.010	0.002	8.4e-09	2.5e-05	33.2
Lipid Metabolism	TC	rs11119887	1	212407562	T	C	0.008	0.001	3.0e-08	2.3e-05	30.7
Lipid Metabolism	TC	rs17712208	1	214150445	A	T	0.027	0.004	9.1e-11	3.2e-05	42.0
Lipid Metabolism	TC	rs12046069	1	220725990	G	A	-0.025	0.004	2.1e-10	3.1e-05	40.4
Lipid Metabolism	TC	rs34404525	1	220975776	T	C	-0.024	0.002	2.8e-37	1.2e-04	162.8
Lipid Metabolism	TC	rs11803981	1	224545410	C	T	-0.014	0.002	2.1e-18	5.8e-05	76.6
Lipid Metabolism	TC	rs12144137	1	224654854	T	C	-0.029	0.004	1.5e-15	4.8e-05	63.6
Lipid Metabolism	TC	rs73083551	1	226276065	T	G	0.015	0.003	1.1e-08	2.5e-05	32.6
Lipid Metabolism	TC	rs7543281	1	234743237	A	G	0.024	0.002	4.2e-35	1.2e-04	152.8
Lipid Metabolism	TC	rs2587534	1	234849339	A	G	0.043	0.001	1.0e-200	7.7e-04	1003.0
Lipid Metabolism	TC	rs10797566	1	235011530	G	T	-0.016	0.001	6.6e-31	1.0e-04	133.6
Lipid Metabolism	TC	rs16844292	1	235099547	C	T	-0.018	0.002	2.9e-18	5.8e-05	75.9
Lipid Metabolism	TC	rs7543562	1	236306825	G	A	-0.008	0.001	4.4e-08	2.3e-05	30.0
Lipid Metabolism	TC	rs3820897	2	3642361	C	T	-0.017	0.002	2.5e-23	7.6e-05	99.0
Lipid Metabolism	TC	rs67269656	2	8720038	T	C	-0.011	0.002	8.4e-13	3.9e-05	51.2
Lipid Metabolism	TC	rs7556983	2	17955255	A	G	-0.016	0.002	1.5e-12	3.8e-05	50.1
Lipid Metabolism	TC	rs870527	2	20369701	A	T	-0.024	0.001	4.5e-73	2.5e-04	326.9
Lipid Metabolism	TC	rs185384697	2	20777778	T	C	-0.042	0.007	5.5e-09	2.6e-05	34.0
Lipid Metabolism	TC	rs559345870	2	20864070	G	C	-1.068	0.148	5.7e-13	1.3e-04	51.9
Lipid Metabolism	TC	rs139095428	2	20899937	A	C	-0.143	0.022	1.3e-10	3.8e-05	41.3
Lipid Metabolism	TC	rs537919294	2	20904624	A	C	-0.153	0.022	1.4e-12	4.2e-05	50.2
Lipid Metabolism	TC	rs185930747	2	20997604	T	A	-0.087	0.009	1.0e-21	7.1e-05	91.7
Lipid Metabolism	TC	rs181651026	2	21055686	T	C	-0.087	0.013	4.8e-11	3.4e-05	43.3
Lipid Metabolism	TC	rs182926316	2	21067006	A	G	-0.083	0.012	3.7e-11	4.2e-05	43.8
Lipid Metabolism	TC	rs547264415	2	21108212	G	A	0.096	0.015	1.2e-10	3.8e-05	41.4
Lipid Metabolism	TC	rs74828123	2	21113461	T	A	-0.162	0.021	9.6e-15	7.4e-05	60.0
Lipid Metabolism	TC	rs536643142	2	21181524	A	G	-0.169	0.012	5.9e-42	1.4e-04	184.2
Lipid Metabolism	TC	rs141870095	2	21204244	A	G	-0.107	0.011	1.1e-20	6.7e-05	87.0
Lipid Metabolism	TC	rs62122481	2	21216815	A	C	0.062	0.001	1.0e-200	1.5e-03	1969.4
Lipid Metabolism	TC	rs72654427	2	21224614	G	A	-0.123	0.020	1.4e-09	3.4e-05	36.6
Lipid Metabolism	TC	rs72654423	2	21225354	C	T	0.120	0.007	5.4e-59	2.0e-04	262.3
Lipid Metabolism	TC	rs1801698	2	21227503	C	T	-0.093	0.012	3.3e-15	5.4e-05	62.1
Lipid Metabolism	TC	rs12713559	2	21229068	A	G	0.268	0.029	1.6e-20	9.5e-05	86.3
Lipid Metabolism	TC	rs1042023	2	21229446	C	G	0.114	0.007	4.5e-69	2.4e-04	308.6
Lipid Metabolism	TC	rs72653053	2	21266223	C	T	-0.073	0.007	5.7e-29	9.5e-05	124.8
Lipid Metabolism	TC	rs186030351	2	21271840	T	C	-0.090	0.012	2.0e-13	4.2e-05	54.1
Lipid Metabolism	TC	rs186651179	2	21278854	C	T	0.093	0.014	5.2e-11	3.5e-05	43.1
Lipid Metabolism	TC	rs57667647	2	21292394	A	G	-0.081	0.003	1.9e-120	4.2e-04	544.6
Lipid Metabolism	TC	rs183281507	2	21298013	A	G	-0.132	0.009	8.8e-47	1.6e-04	206.3
Lipid Metabolism	TC	rs549173888	2	21308173	G	A	0.106	0.019	1.3e-08	8.2e-05	32.3
Lipid Metabolism	TC	rs563927682	2	21308261	T	C	0.068	0.012	3.3e-08	2.7e-05	30.5
Lipid Metabolism	TC	rs189339404	2	21326834	C	A	-0.161	0.024	1.9e-11	5.6e-05	45.1
Lipid Metabolism	TC	rs141437972	2	21336831	T	C	-0.122	0.012	7.4e-25	1.2e-04	106.0
Lipid Metabolism	TC	rs567270299	2	21346488	G	A	0.136	0.022	2.9e-10	3.2e-05	39.8
Lipid Metabolism	TC	rs72782175	2	21366682	C	T	-0.087	0.006	1.3e-49	1.7e-04	219.3
Lipid Metabolism	TC	rs143497571	2	21380700	T	C	-0.082	0.013	1.1e-10	3.2e-05	41.6
Lipid Metabolism	TC	rs537990504	2	21383374	C	G	0.100	0.018	5.0e-08	3.5e-05	29.7
Lipid Metabolism	TC	rs549421994	2	21391233	C	G	-0.227	0.039	6.0e-09	5.4e-05	33.8
Lipid Metabolism	TC	rs553806006	2	21400604	G	A	-0.096	0.017	3.1e-08	2.5e-05	30.6

Phenotype	Trait	SNP	Chr	Position (GRCh37/hg19)	EA	NEA	BETA	SE	P-value	R2	F
Lipid Metabolism	TC	rs191341651	2	21404976	A	C	0.115	0.012	3.5e-21	6.9e-05	89.3
Lipid Metabolism	TC	rs184675979	2	21405099	A	G	0.095	0.010	9.0e-20	8.9e-05	82.8
Lipid Metabolism	TC	rs143013272	2	21408185	T	C	-0.101	0.011	1.6e-19	6.3e-05	81.7
Lipid Metabolism	TC	rs144552532	2	21460714	T	C	-0.092	0.011	1.1e-16	5.3e-05	68.7
Lipid Metabolism	TC	rs149535160	2	21470474	A	G	-0.124	0.015	6.5e-17	5.5e-05	69.8
Lipid Metabolism	TC	rs72789623	2	21472913	C	A	0.045	0.004	1.9e-35	1.2e-04	154.4
Lipid Metabolism	TC	rs13014768	2	21514796	G	C	-0.080	0.002	1.0e-200	8.3e-04	1091.8
Lipid Metabolism	TC	rs191450029	2	21942500	C	G	0.628	0.073	6.5e-18	1.3e-04	74.4
Lipid Metabolism	TC	rs115295106	2	22063652	A	C	-0.039	0.007	2.2e-08	2.4e-05	31.3
Lipid Metabolism	TC	rs552748859	2	22097445	C	A	-0.167	0.024	4.2e-12	5.5e-05	48.0
Lipid Metabolism	TC	rs181370243	2	22245786	A	G	0.303	0.051	2.8e-09	4.3e-05	35.3
Lipid Metabolism	TC	rs144885846	2	22447166	T	G	0.021	0.004	2.8e-09	2.7e-05	35.3
Lipid Metabolism	TC	rs541138574	2	22812173	T	C	0.209	0.032	3.9e-11	5.3e-05	43.7
Lipid Metabolism	TC	rs565710874	2	22850972	A	G	0.253	0.039	1.2e-10	6.0e-05	41.5
Lipid Metabolism	TC	rs150520162	2	23541654	C	T	-0.653	0.113	6.7e-09	5.1e-05	33.6
Lipid Metabolism	TC	rs2113423	2	23942287	T	C	-0.009	0.001	3.4e-11	3.3e-05	43.9
Lipid Metabolism	TC	rs141240885	2	24449850	G	T	-0.016	0.003	4.5e-08	2.3e-05	29.9
Lipid Metabolism	TC	rs146556681	2	25830370	T	C	-0.046	0.008	8.3e-09	2.5e-05	33.2
Lipid Metabolism	TC	rs142787485	2	26358156	G	A	-0.027	0.004	8.1e-13	3.9e-05	51.3
Lipid Metabolism	TC	rs62129477	2	26832419	C	T	0.014	0.002	1.2e-08	2.5e-05	32.4
Lipid Metabolism	TC	rs13394970	2	26929282	G	T	0.012	0.001	1.8e-17	5.5e-05	72.4
Lipid Metabolism	TC	rs77507687	2	26939229	G	A	-0.027	0.004	4.1e-12	3.7e-05	48.1
Lipid Metabolism	TC	rs562421855	2	27434983	C	G	-0.044	0.008	4.7e-08	2.3e-05	29.8
Lipid Metabolism	TC	rs4665972	2	27598097	C	T	-0.058	0.001	1.0e-200	1.3e-03	1723.8
Lipid Metabolism	TC	rs191419430	2	27656038	A	G	-0.059	0.010	6.8e-10	2.9e-05	38.1
Lipid Metabolism	TC	rs183573614	2	27734458	T	C	0.069	0.012	6.4e-09	2.7e-05	33.7
Lipid Metabolism	TC	rs8179239	2	27742747	G	C	-0.051	0.009	7.7e-09	2.6e-05	33.4
Lipid Metabolism	TC	rs75429814	2	27961614	C	T	-0.027	0.002	4.8e-56	1.9e-04	248.8
Lipid Metabolism	TC	rs116827840	2	28010444	C	G	-0.027	0.005	4.0e-08	2.3e-05	30.1
Lipid Metabolism	TC	rs77667092	2	28426708	G	A	-0.048	0.007	1.3e-10	3.2e-05	41.4
Lipid Metabolism	TC	rs4666138	2	29148507	C	G	-0.009	0.001	1.7e-10	3.1e-05	40.7
Lipid Metabolism	TC	rs62142080	2	32182528	C	T	-0.010	0.001	9.1e-12	3.6e-05	46.5
Lipid Metabolism	TC	rs13021948	2	42382775	A	G	0.031	0.005	4.7e-09	2.6e-05	34.3
Lipid Metabolism	TC	rs150139742	2	43492370	A	G	0.044	0.008	2.5e-08	2.4e-05	31.1
Lipid Metabolism	TC	rs186284394	2	43620574	A	G	0.060	0.010	6.5e-10	2.9e-05	38.2
Lipid Metabolism	TC	rs568697539	2	43643014	G	A	-0.118	0.018	3.0e-11	4.9e-05	44.1
Lipid Metabolism	TC	rs116585342	2	43745689	A	T	0.047	0.007	1.4e-11	3.5e-05	45.6
Lipid Metabolism	TC	rs181664929	2	43919041	A	G	-0.098	0.012	2.1e-17	5.6e-05	72.0
Lipid Metabolism	TC	rs113857915	2	43938274	T	C	-0.047	0.005	9.3e-19	6.0e-05	78.2
Lipid Metabolism	TC	rs28473566	2	44012206	A	G	0.028	0.002	1.7e-55	1.9e-04	246.3
Lipid Metabolism	TC	rs141828689	2	44055163	T	C	0.159	0.018	1.3e-19	6.5e-05	82.1
Lipid Metabolism	TC	rs531730588	2	44058373	A	G	0.105	0.018	7.1e-09	3.1e-05	33.5
Lipid Metabolism	TC	rs116487096	2	44067220	A	T	-0.026	0.004	1.7e-13	4.1e-05	54.3
Lipid Metabolism	TC	rs75331444	2	44069772	A	G	-0.104	0.003	1.0e-200	1.1e-03	1512.6
Lipid Metabolism	TC	rs72796745	2	44078192	C	T	0.048	0.003	3.6e-51	1.7e-04	226.4
Lipid Metabolism	TC	rs553497006	2	44085889	G	A	0.047	0.008	6.6e-09	3.0e-05	33.7
Lipid Metabolism	TC	rs574243139	2	44089790	C	T	0.058	0.011	3.5e-08	3.2e-05	30.4
Lipid Metabolism	TC	rs76814535	2	44101743	T	C	-0.026	0.004	5.5e-11	3.3e-05	43.0
Lipid Metabolism	TC	rs111859423	2	44188846	C	T	0.031	0.004	5.0e-14	4.3e-05	56.7
Lipid Metabolism	TC	rs185066471	2	44192439	T	C	-0.078	0.014	2.3e-08	2.4e-05	31.2
Lipid Metabolism	TC	rs11674459	2	44247513	T	C	0.056	0.008	1.2e-11	3.6e-05	46.0
Lipid Metabolism	TC	rs698798	2	44690514	C	A	-0.015	0.003	4.7e-09	2.6e-05	34.3
Lipid Metabolism	TC	rs12712955	2	46166321	G	A	-0.011	0.001	9.4e-16	4.9e-05	64.5
Lipid Metabolism	TC	rs34606253	2	61582738	C	A	-0.013	0.002	2.5e-12	3.8e-05	49.1
Lipid Metabolism	TC	rs4671050	2	62988169	T	G	-0.018	0.001	3.0e-37	1.2e-04	162.6
Lipid Metabolism	TC	rs13036222	2	64046736	G	A	-0.021	0.004	6.8e-09	2.6e-05	33.6

Phenotype	Trait	SNP	Chr	Position (GRCh37/hg19)	EA	NEA	BETA	SE	P-value	R2	F
Lipid Metabolism	TC	rs72808249	2	64172502	T	C	-0.017	0.003	3.5e-08	2.3e-05	30.4
Lipid Metabolism	TC	rs4671103	2	64916181	C	A	0.015	0.001	8.0e-24	7.7e-05	101.3
Lipid Metabolism	TC	rs906578	2	65601600	A	C	-0.011	0.001	1.5e-15	4.8e-05	63.6
Lipid Metabolism	TC	rs2441355	2	70459227	G	A	0.013	0.001	1.2e-20	6.6e-05	86.8
Lipid Metabolism	TC	rs11126331	2	71671116	C	T	-0.009	0.001	2.8e-10	3.0e-05	39.8
Lipid Metabolism	TC	rs62166774	2	85853463	C	G	-0.010	0.001	4.7e-13	4.0e-05	52.3
Lipid Metabolism	TC	rs308914	2	86857824	A	G	-0.009	0.001	1.3e-10	3.2e-05	41.3
Lipid Metabolism	TC	rs2970901	2	88428834	T	G	0.009	0.001	8.0e-12	3.5e-05	46.8
Lipid Metabolism	TC	rs10171847	2	101631888	G	C	-0.011	0.001	9.7e-16	4.9e-05	64.5
Lipid Metabolism	TC	rs1992172	2	109093836	G	A	0.018	0.002	4.4e-25	8.1e-05	107.0
Lipid Metabolism	TC	rs3789129	2	111698040	C	A	0.012	0.002	9.1e-13	3.9e-05	51.0
Lipid Metabolism	TC	rs6734238	2	113841030	G	A	-0.014	0.001	1.1e-23	7.6e-05	100.7
Lipid Metabolism	TC	rs116704418	2	117806119	A	G	-0.021	0.003	7.0e-12	3.6e-05	47.0
Lipid Metabolism	TC	rs140198034	2	118630649	T	G	0.029	0.003	1.7e-24	7.9e-05	104.3
Lipid Metabolism	TC	rs141213470	2	118788583	T	A	0.040	0.006	1.0e-12	3.9e-05	50.8
Lipid Metabolism	TC	rs55938402	2	118843158	A	G	-0.038	0.002	5.3e-62	2.1e-04	276.1
Lipid Metabolism	TC	rs17050272	2	121306440	A	G	-0.020	0.001	6.4e-46	1.6e-04	202.4
Lipid Metabolism	TC	rs2069906	2	128176677	A	G	-0.019	0.003	4.0e-08	2.3e-05	30.1
Lipid Metabolism	TC	rs13032850	2	128615330	G	C	-0.008	0.001	2.0e-08	2.4e-05	31.5
Lipid Metabolism	TC	rs1996591	2	134865046	C	T	0.010	0.002	1.2e-08	2.5e-05	32.4
Lipid Metabolism	TC	rs2460382	2	135014116	A	G	0.011	0.002	1.8e-12	3.8e-05	49.7
Lipid Metabolism	TC	rs4954195	2	135656503	G	C	0.019	0.003	8.2e-09	2.6e-05	33.2
Lipid Metabolism	TC	rs1375131	2	135954797	C	T	0.022	0.002	6.1e-38	1.5e-04	165.8
Lipid Metabolism	TC	rs11688302	2	136960483	T	C	0.012	0.002	1.2e-13	4.2e-05	55.0
Lipid Metabolism	TC	rs11690735	2	144240994	G	A	0.010	0.001	5.7e-11	3.3e-05	42.9
Lipid Metabolism	TC	rs4233566	2	144272229	A	G	-0.010	0.001	2.0e-12	3.8e-05	49.5
Lipid Metabolism	TC	rs10164853	2	158481992	G	A	-0.027	0.003	2.7e-25	8.2e-05	108.0
Lipid Metabolism	TC	rs2111485	2	163110536	G	A	-0.009	0.001	2.3e-11	3.4e-05	44.7
Lipid Metabolism	TC	rs10184004	2	165508389	T	C	-0.009	0.001	1.7e-10	3.1e-05	40.8
Lipid Metabolism	TC	rs10184673	2	169827796	A	G	-0.024	0.001	1.7e-68	2.3e-04	305.9
Lipid Metabolism	TC	rs11553741	2	174232978	C	G	-0.016	0.003	2.0e-08	2.4e-05	31.5
Lipid Metabolism	TC	rs1155061	2	191566381	A	G	0.009	0.001	5.0e-11	3.3e-05	43.2
Lipid Metabolism	TC	rs17860428	2	202151400	A	G	-0.010	0.002	4.9e-08	2.3e-05	29.7
Lipid Metabolism	TC	rs77319662	2	202991651	C	T	0.020	0.002	3.7e-22	7.1e-05	93.7
Lipid Metabolism	TC	rs113867238	2	203495058	A	G	-0.027	0.001	2.2e-74	2.5e-04	333.0
Lipid Metabolism	TC	rs2250522	2	204301296	A	G	0.012	0.001	1.1e-18	5.9e-05	77.9
Lipid Metabolism	TC	rs192276988	2	204472324	A	G	0.046	0.008	4.0e-09	2.7e-05	34.6
Lipid Metabolism	TC	rs2360970	2	208409339	C	G	-0.009	0.001	2.8e-11	3.4e-05	44.3
Lipid Metabolism	TC	rs1250259	2	216300482	A	T	0.017	0.002	1.1e-27	9.0e-05	118.8
Lipid Metabolism	TC	rs10193189	2	219187603	A	C	-0.008	0.001	9.4e-09	2.5e-05	33.0
Lipid Metabolism	TC	rs58650092	2	219304851	A	C	-0.010	0.001	5.1e-13	4.0e-05	52.2
Lipid Metabolism	TC	rs3754629	2	225367669	T	C	0.011	0.002	7.6e-09	2.5e-05	33.4
Lipid Metabolism	TC	rs116001967	2	228451050	A	G	-0.039	0.006	2.5e-10	3.1e-05	40.1
Lipid Metabolism	TC	rs7586105	2	228571182	C	A	-0.012	0.002	1.2e-09	2.8e-05	36.9
Lipid Metabolism	TC	rs6714486	2	234580305	A	T	0.029	0.003	4.0e-26	8.5e-05	111.8
Lipid Metabolism	TC	rs141411255	2	234643591	A	G	-0.029	0.004	1.0e-13	4.2e-05	55.3
Lipid Metabolism	TC	rs887829	2	234668570	T	C	-0.018	0.001	3.1e-38	1.3e-04	167.2
Lipid Metabolism	TC	rs9873301	3	12065264	T	G	-0.033	0.004	1.5e-17	5.5e-05	72.7
Lipid Metabolism	TC	rs564271093	3	12344726	T	C	-0.139	0.020	5.2e-12	5.2e-05	47.6
Lipid Metabolism	TC	rs709147	3	12448940	G	A	-0.024	0.001	1.1e-66	2.3e-04	297.7
Lipid Metabolism	TC	rs186678632	3	12512243	G	A	0.063	0.011	1.2e-08	3.0e-05	32.5
Lipid Metabolism	TC	rs9852128	3	12635623	G	A	-0.025	0.002	1.0e-56	1.9e-04	251.9
Lipid Metabolism	TC	rs6792725	3	24520283	G	A	-0.018	0.002	9.3e-32	1.1e-04	137.5
Lipid Metabolism	TC	rs9837622	3	32514647	A	T	-0.035	0.003	1.2e-40	1.3e-04	178.2
Lipid Metabolism	TC	rs149667965	3	38369902	C	A	0.056	0.010	1.0e-08	2.5e-05	32.8
Lipid Metabolism	TC	rs78595810	3	46909812	T	G	-0.036	0.006	8.1e-11	3.2e-05	42.2

Phenotype	Trait	SNP	Chr	Position (GRCh37/hg19)	EA	NEA	BETA	SE	P-value	R2	F
Lipid Metabolism	TC	rs74780677	3	48601774	G	A	-0.042	0.005	2.2e-15	4.8e-05	62.8
Lipid Metabolism	TC	rs4688756	3	50040194	A	T	-0.008	0.001	3.0e-10	3.0e-05	39.7
Lipid Metabolism	TC	rs2073499	3	50374293	G	A	-0.015	0.002	1.3e-14	4.5e-05	59.4
Lipid Metabolism	TC	rs115578531	3	51435781	G	A	0.038	0.007	1.0e-08	2.5e-05	32.8
Lipid Metabolism	TC	rs79558344	3	51754781	T	C	-0.037	0.006	9.2e-11	3.2e-05	42.0
Lipid Metabolism	TC	rs143799471	3	52559042	T	C	0.053	0.009	5.7e-09	2.6e-05	33.9
Lipid Metabolism	TC	rs11711421	3	52561779	T	C	0.009	0.001	5.1e-12	3.6e-05	47.7
Lipid Metabolism	TC	rs9844432	3	58391293	G	C	-0.037	0.002	3.1e-53	1.8e-04	235.9
Lipid Metabolism	TC	rs7616659	3	58467587	A	G	-0.011	0.001	1.2e-14	4.6e-05	59.6
Lipid Metabolism	TC	rs541035252	3	66608509	A	T	0.196	0.036	3.8e-08	7.4e-05	30.2
Lipid Metabolism	TC	rs55921103	3	69810294	T	G	0.014	0.001	4.1e-23	7.5e-05	98.0
Lipid Metabolism	TC	rs3732359	3	119536429	A	G	-0.020	0.002	1.6e-36	1.2e-04	159.4
Lipid Metabolism	TC	rs112769274	3	122064916	T	G	0.014	0.002	1.1e-20	6.6e-05	86.9
Lipid Metabolism	TC	rs4678218	3	122570898	T	C	0.009	0.001	1.0e-11	3.5e-05	46.3
Lipid Metabolism	TC	rs11708067	3	123065778	G	A	-0.013	0.002	2.5e-17	5.4e-05	71.7
Lipid Metabolism	TC	rs2011442	3	124959834	T	C	0.012	0.001	5.0e-18	5.7e-05	74.9
Lipid Metabolism	TC	rs7641302	3	126060315	C	T	0.012	0.002	8.8e-15	4.6e-05	60.2
Lipid Metabolism	TC	rs12489937	3	127281914	A	G	0.013	0.002	1.9e-09	2.7e-05	36.1
Lipid Metabolism	TC	rs2811477	3	127898783	T	C	0.014	0.002	3.0e-09	2.7e-05	35.2
Lipid Metabolism	TC	rs11920311	3	129275206	T	C	0.019	0.002	8.3e-20	6.3e-05	83.0
Lipid Metabolism	TC	rs74341202	3	132183991	A	G	-0.045	0.003	5.0e-48	1.6e-04	212.0
Lipid Metabolism	TC	rs11915472	3	132250941	G	A	-0.029	0.002	3.5e-33	1.1e-04	144.0
Lipid Metabolism	TC	rs13326361	3	133500294	G	A	0.010	0.002	3.0e-10	3.0e-05	39.6
Lipid Metabolism	TC	rs6765239	3	136389317	C	T	0.009	0.001	1.6e-10	3.1e-05	40.8
Lipid Metabolism	TC	rs6440047	3	141616059	T	C	0.007	0.001	4.9e-08	2.3e-05	29.8
Lipid Metabolism	TC	rs73238180	3	142123392	A	G	-0.015	0.002	1.5e-11	3.5e-05	45.6
Lipid Metabolism	TC	rs9653945	3	142660706	A	G	-0.015	0.001	1.5e-27	9.0e-05	118.3
Lipid Metabolism	TC	rs145146100	3	145947690	T	C	-0.060	0.010	3.9e-10	3.7e-05	39.2
Lipid Metabolism	TC	rs143011636	3	145990185	C	T	-0.062	0.010	6.0e-10	4.2e-05	38.3
Lipid Metabolism	TC	rs62275345	3	147052282	T	C	0.010	0.002	1.7e-10	3.1e-05	40.8
Lipid Metabolism	TC	rs1025567	3	152105979	A	T	0.009	0.002	1.9e-09	2.7e-05	36.1
Lipid Metabolism	TC	rs407258	3	155560978	C	T	0.012	0.002	5.5e-15	4.6e-05	61.1
Lipid Metabolism	TC	rs4679881	3	160028014	C	T	-0.016	0.001	1.9e-31	1.0e-04	136.1
Lipid Metabolism	TC	rs11711206	3	170732945	G	A	0.018	0.002	9.0e-16	4.9e-05	64.6
Lipid Metabolism	TC	rs79287178	3	172294500	A	G	0.023	0.004	2.3e-08	2.4e-05	31.2
Lipid Metabolism	TC	rs10513801	3	185822353	G	T	-0.018	0.002	3.8e-19	6.1e-05	80.0
Lipid Metabolism	TC	rs2518134	3	186332182	T	C	-0.008	0.001	1.9e-08	2.4e-05	31.6
Lipid Metabolism	TC	rs16861497	3	186731327	T	G	-0.008	0.001	3.2e-08	2.3e-05	30.6
Lipid Metabolism	TC	rs139828053	3	195298892	C	T	-0.023	0.004	3.7e-08	2.3e-05	30.3
Lipid Metabolism	TC	rs78139882	4	3287361	T	C	0.010	0.002	1.7e-09	2.8e-05	36.3
Lipid Metabolism	TC	rs59950280	4	3452345	A	G	0.025	0.001	2.1e-63	2.2e-04	282.5
Lipid Metabolism	TC	rs731450	4	3490328	T	C	0.014	0.001	1.3e-21	7.0e-05	91.2
Lipid Metabolism	TC	rs13141777	4	6913571	A	G	-0.011	0.002	1.7e-08	2.4e-05	31.8
Lipid Metabolism	TC	rs4689653	4	7223319	G	T	0.011	0.001	4.3e-15	4.7e-05	61.6
Lipid Metabolism	TC	rs2002574	4	8224276	C	T	-0.009	0.002	1.2e-08	2.5e-05	32.5
Lipid Metabolism	TC	rs734122	4	10089865	A	G	0.009	0.001	6.1e-10	2.9e-05	38.3
Lipid Metabolism	TC	rs12504134	4	39485834	T	C	-0.009	0.001	1.6e-09	2.8e-05	36.4
Lipid Metabolism	TC	rs78711227	4	40002526	T	C	-0.015	0.003	4.1e-08	2.3e-05	30.1
Lipid Metabolism	TC	rs278940	4	40420752	G	A	0.008	0.001	2.5e-08	2.4e-05	31.0
Lipid Metabolism	TC	rs4864471	4	54426184	T	G	-0.011	0.002	2.7e-08	2.3e-05	30.9
Lipid Metabolism	TC	rs142565287	4	69290405	G	A	-0.051	0.008	1.8e-10	3.1e-05	40.6
Lipid Metabolism	TC	rs142107676	4	69319842	A	G	-0.049	0.008	9.6e-11	3.2e-05	41.9
Lipid Metabolism	TC	rs185528097	4	69339236	T	A	0.057	0.008	1.2e-11	3.6e-05	46.0
Lipid Metabolism	TC	rs138894878	4	69365984	T	C	-0.051	0.008	8.2e-10	3.0e-05	37.7
Lipid Metabolism	TC	rs34707604	4	69491456	C	T	0.039	0.002	5.9e-103	3.8e-04	464.2
Lipid Metabolism	TC	rs4148258	4	69534943	A	T	-0.017	0.002	3.1e-24	8.5e-05	103.2

Phenotype	Trait	SNP	Chr	Position (GRCh37/hg19)	EA	NEA	BETA	SE	P-value	R2	F
Lipid Metabolism	TC	rs61361928	4	69962375	C	T	-0.094	0.010	5.9e-20	7.3e-05	83.6
Lipid Metabolism	TC	rs559866136	4	70239794	C	T	-0.090	0.015	5.1e-09	4.0e-05	34.2
Lipid Metabolism	TC	rs569629844	4	71166236	A	G	0.158	0.021	5.6e-14	6.9e-05	56.5
Lipid Metabolism	TC	rs531876114	4	71341209	A	G	0.153	0.018	7.6e-17	8.3e-05	69.5
Lipid Metabolism	TC	rs143736900	4	72871285	C	T	0.082	0.011	5.2e-15	5.3e-05	61.2
Lipid Metabolism	TC	rs190671241	4	73574142	G	A	0.332	0.018	2.3e-76	4.0e-04	342.0
Lipid Metabolism	TC	rs187918276	4	74033564	C	G	0.523	0.021	6.7e-134	6.3e-04	606.4
Lipid Metabolism	TC	rs72663045	4	74177397	G	T	0.043	0.005	4.5e-19	6.1e-05	79.6
Lipid Metabolism	TC	rs148885848	4	74853287	A	G	0.361	0.020	1.7e-72	3.7e-04	324.3
Lipid Metabolism	TC	rs6531778	4	76541303	A	G	0.017	0.002	1.1e-16	5.2e-05	68.7
Lipid Metabolism	TC	rs17002033	4	77432950	T	C	-0.015	0.002	1.3e-10	3.1e-05	41.3
Lipid Metabolism	TC	rs17605615	4	87996745	A	G	0.013	0.001	1.4e-20	6.6e-05	86.5
Lipid Metabolism	TC	rs114112910	4	100313350	G	A	0.032	0.006	2.6e-08	2.4e-05	31.0
Lipid Metabolism	TC	rs72906779	4	100445063	C	T	-0.031	0.004	7.4e-13	3.9e-05	51.4
Lipid Metabolism	TC	rs10516444	4	100462137	G	A	-0.017	0.002	2.0e-27	8.9e-05	117.7
Lipid Metabolism	TC	rs10022442	4	102470700	T	G	0.007	0.001	4.2e-08	2.3e-05	30.1
Lipid Metabolism	TC	rs13135092	4	103198082	G	A	-0.035	0.003	4.0e-42	1.4e-04	185.0
Lipid Metabolism	TC	rs230515	4	103471434	A	G	-0.009	0.001	1.7e-09	2.8e-05	36.3
Lipid Metabolism	TC	rs1860129	4	110886343	C	G	-0.008	0.001	5.1e-09	2.6e-05	34.2
Lipid Metabolism	TC	rs148046400	4	119487471	A	G	-0.015	0.003	3.7e-08	2.3e-05	30.3
Lipid Metabolism	TC	rs138204164	4	120123417	G	C	-0.014	0.002	1.3e-12	3.8e-05	50.3
Lipid Metabolism	TC	rs62335637	4	128987524	A	G	0.009	0.001	1.0e-10	3.2e-05	41.8
Lipid Metabolism	TC	rs2085724	4	151206200	C	A	0.010	0.001	9.1e-12	3.5e-05	46.5
Lipid Metabolism	TC	rs72729610	4	154190965	G	A	-0.020	0.002	5.8e-28	9.1e-05	120.2
Lipid Metabolism	TC	rs17029886	4	154277323	A	G	0.009	0.002	4.5e-10	2.9e-05	38.9
Lipid Metabolism	TC	rs534659996	4	155308673	G	T	0.120	0.015	1.9e-15	5.2e-05	63.2
Lipid Metabolism	TC	rs551659708	4	155424667	T	C	0.186	0.023	1.2e-15	7.8e-05	64.0
Lipid Metabolism	TC	rs6054	4	155489608	T	C	0.143	0.010	6.9e-43	1.5e-04	188.5
Lipid Metabolism	TC	rs793900	4	185253019	T	C	0.010	0.001	8.9e-14	4.2e-05	55.6
Lipid Metabolism	TC	rs548247798	5	12967662	A	T	0.748	0.132	1.4e-08	8.2e-05	32.2
Lipid Metabolism	TC	rs10060055	5	13989882	G	A	-0.008	0.001	5.0e-08	2.3e-05	29.7
Lipid Metabolism	TC	rs10044883	5	35885710	C	T	0.008	0.001	3.7e-08	2.3e-05	30.3
Lipid Metabolism	TC	rs1605145	5	39559905	T	A	0.010	0.001	1.4e-13	4.1e-05	54.7
Lipid Metabolism	TC	rs116734477	5	52095024	T	C	-0.035	0.004	6.9e-23	7.4e-05	97.0
Lipid Metabolism	TC	rs4074793	5	52193125	G	A	0.017	0.002	2.3e-11	3.4e-05	44.7
Lipid Metabolism	TC	rs79760705	5	53298716	T	G	0.015	0.002	7.2e-12	3.6e-05	47.0
Lipid Metabolism	TC	rs11743303	5	55859952	G	A	0.015	0.002	4.1e-19	6.0e-05	79.8
Lipid Metabolism	TC	rs43217	5	57578037	G	A	-0.009	0.001	3.6e-09	2.6e-05	34.8
Lipid Metabolism	TC	rs3010278	5	72013573	G	T	-0.017	0.002	2.2e-26	8.6e-05	113.0
Lipid Metabolism	TC	rs10462337	5	72386784	T	C	-0.014	0.002	1.3e-14	4.5e-05	59.3
Lipid Metabolism	TC	rs145275181	5	74201340	G	T	0.039	0.007	4.6e-08	2.3e-05	29.9
Lipid Metabolism	TC	rs114634813	5	74264761	A	G	0.042	0.007	3.9e-10	3.0e-05	39.2
Lipid Metabolism	TC	rs4554186	5	74295766	C	G	-0.024	0.003	5.7e-15	4.6e-05	61.0
Lipid Metabolism	TC	rs6879223	5	74370768	C	T	0.035	0.006	1.9e-10	3.1e-05	40.5
Lipid Metabolism	TC	rs139159707	5	74389034	A	G	0.094	0.016	1.4e-08	2.5e-05	32.2
Lipid Metabolism	TC	rs192436363	5	74451087	T	G	0.047	0.008	6.0e-09	3.0e-05	33.8
Lipid Metabolism	TC	rs369330343	5	74566173	C	T	0.059	0.002	1.0e-200	1.3e-03	1483.1
Lipid Metabolism	TC	rs180978629	5	74688599	T	C	0.062	0.011	4.8e-08	2.3e-05	29.8
Lipid Metabolism	TC	rs145888305	5	74697794	G	A	0.063	0.011	4.6e-09	2.7e-05	34.3
Lipid Metabolism	TC	rs186466152	5	74894653	G	A	0.084	0.011	1.9e-15	4.9e-05	63.2
Lipid Metabolism	TC	rs544623152	5	75020576	C	T	0.122	0.022	1.2e-08	3.4e-05	32.4
Lipid Metabolism	TC	rs55740595	5	75162866	T	C	-0.019	0.002	1.9e-21	6.9e-05	90.5
Lipid Metabolism	TC	rs192990170	5	75195986	A	G	0.048	0.008	4.0e-09	2.7e-05	34.6
Lipid Metabolism	TC	rs184829739	5	75296844	C	A	0.038	0.007	7.1e-09	2.6e-05	33.5
Lipid Metabolism	TC	rs6874435	5	75574855	G	A	0.017	0.002	2.4e-17	5.4e-05	71.8
Lipid Metabolism	TC	rs6896005	5	122834112	C	T	0.015	0.001	4.1e-28	9.2e-05	120.8

Phenotype	Trait	SNP	Chr	Position (GRCh37/hg19)	EA	NEA	BETA	SE	P-value	R2	F
Lipid Metabolism	TC	rs2057655	5	131807624	A	G	-0.015	0.002	1.3e-18	5.9e-05	77.6
Lipid Metabolism	TC	rs269781	5	139545447	A	G	-0.013	0.002	1.1e-15	4.9e-05	64.2
Lipid Metabolism	TC	rs11167778	5	141888881	T	C	0.016	0.002	1.3e-13	4.2e-05	54.8
Lipid Metabolism	TC	rs185653065	5	155581796	G	A	0.156	0.027	8.9e-09	4.3e-05	33.1
Lipid Metabolism	TC	rs555957167	5	155706629	G	T	-0.080	0.013	1.1e-09	4.2e-05	37.1
Lipid Metabolism	TC	rs115859006	5	155935836	G	A	0.019	0.003	3.8e-08	2.3e-05	30.2
Lipid Metabolism	TC	rs6886573	5	156265523	G	T	0.038	0.004	6.5e-24	7.7e-05	101.7
Lipid Metabolism	TC	rs139294635	5	156360413	C	T	-0.061	0.010	5.3e-10	3.0e-05	38.5
Lipid Metabolism	TC	rs11134475	5	156399950	G	A	0.043	0.001	1.0e-200	7.2e-04	952.1
Lipid Metabolism	TC	rs193010652	5	156410046	A	C	0.170	0.019	5.8e-20	6.7e-05	83.7
Lipid Metabolism	TC	rs186756395	5	156449311	C	T	0.158	0.020	3.6e-15	6.6e-05	61.9
Lipid Metabolism	TC	rs12520950	5	156474469	A	G	-0.094	0.006	3.2e-48	1.6e-04	212.9
Lipid Metabolism	TC	rs189819253	5	156596113	C	T	0.052	0.009	1.7e-09	2.8e-05	36.2
Lipid Metabolism	TC	rs188238483	5	180308441	C	T	-0.043	0.008	1.5e-08	2.5e-05	32.1
Lipid Metabolism	TC	rs4713979	6	11868682	C	T	-0.013	0.002	3.0e-16	5.2e-05	66.8
Lipid Metabolism	TC	rs6459440	6	15995565	G	A	-0.011	0.001	3.4e-15	4.7e-05	62.0
Lipid Metabolism	TC	rs2235215	6	16131156	C	T	-0.028	0.001	3.5e-82	2.8e-04	368.7
Lipid Metabolism	TC	rs74451139	6	16176210	G	C	-0.019	0.003	3.9e-10	3.0e-05	39.1
Lipid Metabolism	TC	rs6920873	6	18771285	T	C	-0.008	0.001	1.3e-08	2.5e-05	32.3
Lipid Metabolism	TC	rs72828564	6	19146030	G	T	0.019	0.003	4.9e-12	3.6e-05	47.7
Lipid Metabolism	TC	rs9465733	6	20409775	C	A	0.011	0.001	9.9e-16	5.0e-05	64.5
Lipid Metabolism	TC	rs113977268	6	22359515	A	G	-0.018	0.002	6.1e-13	4.0e-05	51.8
Lipid Metabolism	TC	rs144523305	6	25534304	T	C	0.032	0.006	1.2e-08	2.5e-05	32.5
Lipid Metabolism	TC	rs80215559	6	25918225	C	T	-0.051	0.003	6.5e-78	2.7e-04	349.1
Lipid Metabolism	TC	rs141146569	6	25927030	T	C	0.038	0.007	1.6e-08	2.5e-05	31.9
Lipid Metabolism	TC	rs2032451	6	26092170	T	G	-0.012	0.002	6.8e-10	2.9e-05	38.1
Lipid Metabolism	TC	rs62396175	6	26648517	T	G	0.038	0.006	5.3e-12	3.7e-05	47.6
Lipid Metabolism	TC	rs35657082	6	27067657	T	A	-0.029	0.003	6.5e-30	9.8e-05	129.1
Lipid Metabolism	TC	rs62401424	6	27966203	C	T	0.049	0.007	8.0e-13	4.2e-05	51.3
Lipid Metabolism	TC	rs71559077	6	28328065	A	G	-0.027	0.003	5.2e-26	8.5e-05	111.3
Lipid Metabolism	TC	rs9257187	6	28756902	T	C	-0.016	0.002	1.1e-10	3.2e-05	41.6
Lipid Metabolism	TC	rs113360274	6	29850784	G	A	0.020	0.002	4.3e-28	9.9e-05	120.8
Lipid Metabolism	TC	rs1264706	6	30063652	C	G	-0.019	0.002	4.0e-16	5.4e-05	66.2
Lipid Metabolism	TC	rs2524143	6	31263510	C	T	0.025	0.002	2.3e-58	2.0e-04	259.4
Lipid Metabolism	TC	rs114751021	6	31504194	G	A	0.048	0.004	5.1e-28	9.7e-05	120.4
Lipid Metabolism	TC	rs34744948	6	31562767	C	G	0.025	0.003	3.0e-18	6.1e-05	75.9
Lipid Metabolism	TC	rs138719004	6	31576508	G	A	0.036	0.004	1.1e-15	5.3e-05	64.3
Lipid Metabolism	TC	rs28752516	6	32583611	G	A	0.022	0.002	3.1e-45	1.8e-04	199.2
Lipid Metabolism	TC	rs188694433	6	32617104	T	C	-0.017	0.003	1.1e-10	6.6e-05	41.6
Lipid Metabolism	TC	rs545361737	6	32618493	A	G	-0.033	0.006	9.5e-09	1.4e-04	32.9
Lipid Metabolism	TC	rs6689	6	32627700	G	A	0.043	0.002	5.3e-125	4.4e-04	565.5
Lipid Metabolism	TC	rs3208181	6	32713030	C	T	0.017	0.001	8.7e-32	1.1e-04	137.7
Lipid Metabolism	TC	rs2071350	6	33043526	T	C	0.020	0.004	2.2e-08	2.5e-05	31.3
Lipid Metabolism	TC	rs3800461	6	34616322	C	G	-0.035	0.002	3.4e-64	2.2e-04	286.2
Lipid Metabolism	TC	rs77262732	6	34734915	A	G	0.035	0.005	8.6e-11	3.2e-05	42.1
Lipid Metabolism	TC	rs763155	6	35193773	C	A	-0.017	0.002	1.7e-15	4.8e-05	63.3
Lipid Metabolism	TC	rs143018638	6	35679692	T	C	-0.052	0.007	3.6e-15	4.7e-05	61.9
Lipid Metabolism	TC	rs4714035	6	37048827	A	G	-0.015	0.001	5.1e-29	9.5e-05	125.0
Lipid Metabolism	TC	rs11961538	6	39231013	C	T	0.010	0.001	1.7e-11	3.5e-05	45.3
Lipid Metabolism	TC	rs9472125	6	43756169	T	C	-0.013	0.002	4.9e-09	2.7e-05	34.2
Lipid Metabolism	TC	rs68137036	6	43820215	G	A	-0.010	0.001	7.0e-12	3.6e-05	47.0
Lipid Metabolism	TC	rs59842359	6	44105929	T	C	0.009	0.002	4.9e-08	2.3e-05	29.8
Lipid Metabolism	TC	rs9463799	6	52445000	G	A	-0.013	0.001	8.9e-19	5.9e-05	78.3
Lipid Metabolism	TC	rs9474328	6	52687768	A	G	-0.010	0.001	5.7e-13	4.0e-05	51.9
Lipid Metabolism	TC	rs12662589	6	53509035	C	G	0.015	0.002	4.1e-22	7.1e-05	93.5
Lipid Metabolism	TC	rs78485726	6	53742184	A	G	-0.019	0.004	3.4e-08	2.3e-05	30.4

Phenotype	Trait	SNP	Chr	Position (GRCh37/hg19)	EA	NEA	BETA	SE	P-value	R2	F
Lipid Metabolism	TC	rs146824639	6	88166740	C	T	0.010	0.002	1.8e-10	3.6e-05	40.6
Lipid Metabolism	TC	rs9496567	6	100602753	A	G	-0.021	0.002	7.2e-41	1.4e-04	179.2
Lipid Metabolism	TC	rs1358756	6	101432314	G	A	0.012	0.001	3.5e-18	5.7e-05	75.6
Lipid Metabolism	TC	rs11968490	6	106381668	T	C	0.010	0.001	1.6e-12	3.8e-05	49.9
Lipid Metabolism	TC	rs11153143	6	109300661	T	C	-0.016	0.002	4.7e-15	4.8e-05	61.4
Lipid Metabolism	TC	rs3798227	6	116324898	G	A	-0.021	0.001	7.9e-55	1.8e-04	243.2
Lipid Metabolism	TC	rs12195627	6	121803109	C	G	-0.009	0.002	4.0e-08	2.3e-05	30.1
Lipid Metabolism	TC	rs9388498	6	126873423	T	G	-0.016	0.002	4.2e-18	5.8e-05	75.2
Lipid Metabolism	TC	rs577721086	6	127440047	C	T	0.017	0.003	2.0e-08	2.4e-05	31.5
Lipid Metabolism	TC	rs3890746	6	130371055	T	C	0.010	0.001	3.6e-13	4.0e-05	52.9
Lipid Metabolism	TC	rs6929820	6	131882080	C	T	-0.009	0.002	5.5e-09	2.6e-05	34.0
Lipid Metabolism	TC	rs6900310	6	135200656	T	C	0.017	0.003	5.9e-11	3.7e-05	42.8
Lipid Metabolism	TC	rs9402685	6	135419688	C	T	-0.027	0.002	1.3e-72	2.5e-04	324.8
Lipid Metabolism	TC	rs62441843	6	139331253	G	A	0.020	0.002	1.2e-29	9.7e-05	127.8
Lipid Metabolism	TC	rs6570299	6	139340944	T	A	0.012	0.001	7.8e-15	5.3e-05	60.4
Lipid Metabolism	TC	rs2095369	6	143270216	G	A	-0.009	0.002	8.0e-10	2.9e-05	37.8
Lipid Metabolism	TC	rs73006267	6	144322823	T	G	0.014	0.002	7.9e-09	2.5e-05	33.3
Lipid Metabolism	TC	rs9397427	6	151875034	G	A	0.018	0.002	5.4e-18	5.7e-05	74.7
Lipid Metabolism	TC	rs3853252	6	152170247	A	G	-0.008	0.001	7.2e-09	2.6e-05	33.5
Lipid Metabolism	TC	rs140990847	6	160339987	A	G	-0.088	0.016	3.0e-08	2.9e-05	30.7
Lipid Metabolism	TC	rs73021693	6	160439725	A	G	0.035	0.004	2.2e-21	6.9e-05	90.2
Lipid Metabolism	TC	rs78423775	6	160519228	A	G	-0.026	0.003	3.6e-22	7.1e-05	93.8
Lipid Metabolism	TC	rs185887328	6	160534053	G	C	-0.050	0.007	1.0e-11	3.6e-05	46.3
Lipid Metabolism	TC	rs12208357	6	160543148	T	C	0.060	0.003	3.0e-119	4.1e-04	539.0
Lipid Metabolism	TC	rs146785976	6	160545953	G	A	-0.035	0.005	6.2e-13	4.0e-05	51.8
Lipid Metabolism	TC	rs117301306	6	160561308	T	C	-0.036	0.006	1.3e-08	2.5e-05	32.3
Lipid Metabolism	TC	rs118053827	6	160580496	T	C	-0.038	0.005	5.6e-16	5.0e-05	65.6
Lipid Metabolism	TC	rs565797680	6	160628813	T	C	0.121	0.022	3.6e-08	3.6e-05	30.4
Lipid Metabolism	TC	rs562632887	6	160654044	T	C	0.258	0.038	1.1e-11	1.1e-04	46.2
Lipid Metabolism	TC	rs148163986	6	160692311	A	G	0.137	0.023	3.8e-09	4.3e-05	34.7
Lipid Metabolism	TC	rs189821701	6	160862809	T	C	0.081	0.010	1.9e-15	5.0e-05	63.2
Lipid Metabolism	TC	rs549975194	6	160863985	T	C	0.146	0.009	2.0e-54	1.9e-04	241.3
Lipid Metabolism	TC	rs117275341	6	160898907	T	G	-0.050	0.007	5.8e-12	3.6e-05	47.4
Lipid Metabolism	TC	rs147555597	6	160911596	A	G	0.115	0.007	1.5e-53	1.8e-04	237.4
Lipid Metabolism	TC	rs558059457	6	160960190	G	A	0.180	0.031	7.3e-09	8.0e-05	33.5
Lipid Metabolism	TC	rs41265924	6	160965618	G	A	-0.044	0.003	1.9e-38	1.3e-04	168.1
Lipid Metabolism	TC	rs41264844	6	160969075	T	C	-0.036	0.004	6.5e-18	5.7e-05	74.4
Lipid Metabolism	TC	rs10455872	6	161010118	G	A	0.101	0.003	1.0e-200	1.1e-03	1447.9
Lipid Metabolism	TC	rs140570886	6	161013013	C	T	0.172	0.006	1.9e-190	6.6e-04	866.5
Lipid Metabolism	TC	rs59948046	6	161119211	T	C	0.619	0.065	2.0e-21	2.3e-04	90.4
Lipid Metabolism	TC	rs557242113	6	161175245	A	G	0.139	0.025	2.2e-08	3.8e-05	31.3
Lipid Metabolism	TC	rs185990325	6	161200366	G	A	0.082	0.011	3.4e-13	6.0e-05	53.0
Lipid Metabolism	TC	rs532754706	6	161329915	G	A	0.048	0.008	1.4e-09	3.9e-05	36.6
Lipid Metabolism	TC	rs138797047	6	161334303	T	A	0.282	0.042	1.4e-11	1.2e-04	45.7
Lipid Metabolism	TC	rs150908701	6	161421886	C	T	0.162	0.017	5.9e-22	8.5e-05	92.8
Lipid Metabolism	TC	rs551938102	6	161430830	G	A	0.154	0.021	7.1e-14	5.8e-05	56.0
Lipid Metabolism	TC	rs55660955	6	161658128	A	G	0.030	0.004	5.3e-17	5.3e-05	70.2
Lipid Metabolism	TC	rs73672461	7	943950	A	G	0.022	0.003	2.8e-13	4.1e-05	53.3
Lipid Metabolism	TC	rs10272002	7	1047615	G	A	-0.023	0.002	8.7e-45	1.5e-04	197.2
Lipid Metabolism	TC	rs566502095	7	1095714	A	G	-0.092	0.015	7.8e-10	3.3e-05	37.8
Lipid Metabolism	TC	rs188948393	7	1120654	A	G	-0.110	0.020	2.5e-08	3.3e-05	31.1
Lipid Metabolism	TC	rs77523736	7	2071957	A	G	0.132	0.016	2.3e-16	7.9e-05	67.4
Lipid Metabolism	TC	rs532639357	7	2393346	C	A	0.157	0.019	3.2e-16	7.2e-05	66.7
Lipid Metabolism	TC	rs836506	7	6455246	G	A	0.013	0.001	1.4e-17	6.3e-05	72.8
Lipid Metabolism	TC	rs12702510	7	6485196	T	C	-0.015	0.001	8.3e-24	7.7e-05	101.2
Lipid Metabolism	TC	rs10282707	7	17911038	T	C	-0.010	0.001	3.7e-14	4.3e-05	57.3

Phenotype	Trait	SNP	Chr	Position (GRCh37/hg19)	EA	NEA	BETA	SE	P-value	R2	F
Lipid Metabolism	TC	rs11763703	7	21243071	C	A	-0.008	0.001	5.0e-09	2.6e-05	34.2
Lipid Metabolism	TC	rs7811417	7	21534152	C	T	0.020	0.001	9.6e-46	1.5e-04	201.6
Lipid Metabolism	TC	rs55649657	7	21607283	G	C	0.037	0.002	5.3e-113	3.9e-04	510.4
Lipid Metabolism	TC	rs4000713	7	25990597	A	G	0.016	0.001	6.1e-27	8.8e-05	115.5
Lipid Metabolism	TC	rs4719925	7	28188995	G	A	0.013	0.002	3.9e-08	2.3e-05	30.2
Lipid Metabolism	TC	rs57854543	7	36184158	G	C	0.014	0.002	5.8e-16	5.0e-05	65.5
Lipid Metabolism	TC	rs73102255	7	36410738	T	A	-0.017	0.002	2.8e-16	5.1e-05	66.9
Lipid Metabolism	TC	rs7776825	7	41753331	G	A	0.012	0.002	1.2e-13	4.2e-05	55.1
Lipid Metabolism	TC	rs145402019	7	43584065	C	A	0.026	0.004	1.1e-09	2.8e-05	37.1
Lipid Metabolism	TC	rs141530948	7	44511663	A	G	-0.050	0.009	4.2e-08	2.3e-05	30.0
Lipid Metabolism	TC	rs139659653	7	44578747	A	G	-0.162	0.025	2.4e-10	5.0e-05	40.1
Lipid Metabolism	TC	rs2073547	7	44582331	G	A	0.041	0.002	2.5e-90	4.5e-04	406.2
Lipid Metabolism	TC	rs886652	7	44671016	T	C	0.020	0.001	2.9e-48	1.6e-04	213.1
Lipid Metabolism	TC	rs113548930	7	44847350	A	T	0.023	0.004	7.0e-11	3.3e-05	42.5
Lipid Metabolism	TC	rs35489648	7	72870211	A	G	-0.016	0.003	2.9e-09	2.7e-05	35.2
Lipid Metabolism	TC	rs113562761	7	73081694	G	C	0.034	0.004	3.2e-20	6.5e-05	84.8
Lipid Metabolism	TC	rs1057868	7	75615006	T	C	0.012	0.001	1.3e-15	4.8e-05	63.8
Lipid Metabolism	TC	rs73138424	7	77488316	G	C	0.008	0.002	2.6e-08	2.4e-05	31.0
Lipid Metabolism	TC	rs79196540	7	81471627	C	T	0.016	0.002	1.1e-10	3.1e-05	41.6
Lipid Metabolism	TC	rs45479991	7	87083339	T	A	-0.014	0.002	1.9e-15	4.8e-05	63.1
Lipid Metabolism	TC	rs28364270	7	87133932	C	T	0.040	0.007	3.2e-08	2.4e-05	30.6
Lipid Metabolism	TC	rs42032	7	92237426	A	G	-0.008	0.002	4.1e-08	2.3e-05	30.1
Lipid Metabolism	TC	rs854562	7	94947969	T	C	-0.008	0.001	1.7e-08	2.4e-05	31.8
Lipid Metabolism	TC	rs3779196	7	97990106	C	T	0.017	0.002	3.8e-21	6.8e-05	89.1
Lipid Metabolism	TC	rs7803454	7	99991548	T	C	-0.010	0.002	1.6e-09	2.8e-05	36.4
Lipid Metabolism	TC	rs221786	7	100266081	C	T	-0.025	0.002	4.5e-32	1.1e-04	139.0
Lipid Metabolism	TC	rs3757459	7	100881429	T	C	-0.009	0.002	5.2e-09	2.6e-05	34.1
Lipid Metabolism	TC	rs803073	7	101928279	A	G	-0.008	0.001	1.2e-08	2.5e-05	32.5
Lipid Metabolism	TC	rs10248717	7	107056795	G	A	0.009	0.001	3.9e-09	2.7e-05	34.7
Lipid Metabolism	TC	rs38835	7	116304538	A	C	-0.010	0.002	2.9e-08	2.4e-05	30.8
Lipid Metabolism	TC	rs6960373	7	123339177	T	C	-0.007	0.001	4.3e-08	2.3e-05	30.0
Lipid Metabolism	TC	rs62621812	7	127015083	A	G	0.026	0.004	5.7e-09	2.6e-05	33.9
Lipid Metabolism	TC	rs78129283	7	134842929	A	G	-0.016	0.002	8.9e-11	3.2e-05	42.1
Lipid Metabolism	TC	rs12530805	7	135278791	T	C	-0.010	0.002	1.5e-08	2.4e-05	32.1
Lipid Metabolism	TC	rs80206917	7	140159389	C	T	-0.009	0.002	1.5e-08	2.5e-05	32.0
Lipid Metabolism	TC	rs4374942	7	155026807	C	T	0.021	0.002	4.9e-17	5.3e-05	70.4
Lipid Metabolism	TC	rs6975911	7	155046182	A	G	0.028	0.004	6.8e-11	3.3e-05	42.6
Lipid Metabolism	TC	rs11761517	7	158532706	T	C	-0.008	0.001	1.1e-09	2.9e-05	37.1
Lipid Metabolism	TC	rs1971312	8	6582981	T	C	0.012	0.001	3.3e-16	5.1e-05	66.6
Lipid Metabolism	TC	rs2169387	8	9181395	G	A	0.075	0.002	1.0e-200	8.5e-04	1114.1
Lipid Metabolism	TC	rs28396619	8	9197007	G	A	0.019	0.002	7.3e-15	4.6e-05	60.5
Lipid Metabolism	TC	rs17729883	8	9256631	C	T	-0.015	0.001	1.2e-24	8.0e-05	105.1
Lipid Metabolism	TC	rs186245508	8	9443064	C	A	-0.059	0.008	4.3e-13	4.0e-05	52.5
Lipid Metabolism	TC	rs77193855	8	9505888	G	A	0.024	0.004	2.0e-11	3.4e-05	44.9
Lipid Metabolism	TC	rs187373241	8	9938285	A	G	-0.063	0.010	4.4e-10	3.0e-05	38.9
Lipid Metabolism	TC	rs1736082	8	11703420	T	C	0.013	0.002	1.0e-16	5.2e-05	68.9
Lipid Metabolism	TC	rs7844866	8	18196091	G	A	0.025	0.003	8.5e-19	5.9e-05	78.4
Lipid Metabolism	TC	rs35570672	8	18272635	C	T	-0.033	0.002	1.5e-91	3.1e-04	411.8
Lipid Metabolism	TC	rs900776	8	21918089	C	A	-0.018	0.002	4.9e-23	7.4e-05	97.7
Lipid Metabolism	TC	rs11781222	8	23389571	C	T	-0.012	0.002	6.4e-10	2.9e-05	38.2
Lipid Metabolism	TC	rs11779927	8	27947787	G	A	0.008	0.001	2.8e-08	2.3e-05	30.8
Lipid Metabolism	TC	rs76324938	8	28843833	A	G	0.029	0.004	4.9e-15	4.7e-05	61.3
Lipid Metabolism	TC	rs117139027	8	29024943	A	G	-0.067	0.006	1.0e-26	1.0e-04	114.5
Lipid Metabolism	TC	rs34265667	8	41542093	A	G	-0.027	0.004	1.9e-12	3.8e-05	49.6
Lipid Metabolism	TC	rs9298506	8	55437524	G	A	0.025	0.002	9.1e-51	1.7e-04	224.6
Lipid Metabolism	TC	rs533849833	8	55848528	G	A	-0.069	0.012	1.4e-08	2.8e-05	32.2

Phenotype	Trait	SNP	Chr	Position (GRCh37/hg19)	EA	NEA	BETA	SE	P-value	R2	F
Lipid Metabolism	TC	rs111787389	8	59197756	A	G	0.042	0.007	7.1e-10	2.9e-05	38.0
Lipid Metabolism	TC	rs75716564	8	59310828	A	G	-0.056	0.008	4.8e-11	3.3e-05	43.2
Lipid Metabolism	TC	rs9297994	8	59392324	A	G	-0.039	0.001	1.5e-167	5.8e-04	761.1
Lipid Metabolism	TC	rs113847133	8	59542732	A	G	-0.040	0.003	1.5e-36	1.2e-04	159.4
Lipid Metabolism	TC	rs6992869	8	61395832	C	T	0.014	0.001	1.9e-25	8.3e-05	108.7
Lipid Metabolism	TC	rs2383922	8	74892520	T	C	-0.013	0.001	7.1e-21	6.7e-05	87.8
Lipid Metabolism	TC	rs396760	8	81377803	A	G	0.009	0.001	5.4e-10	2.9e-05	38.5
Lipid Metabolism	TC	rs1474412	8	102587259	A	G	-0.010	0.002	3.0e-10	3.0e-05	39.7
Lipid Metabolism	TC	rs2737245	8	116658583	T	G	-0.029	0.001	2.8e-82	2.8e-04	369.2
Lipid Metabolism	TC	rs10955991	8	121867780	C	T	-0.009	0.001	5.3e-11	3.3e-05	43.1
Lipid Metabolism	TC	rs2385094	8	126356449	C	T	-0.018	0.002	9.4e-19	5.9e-05	78.2
Lipid Metabolism	TC	rs11989091	8	126437886	A	G	0.016	0.002	1.2e-18	5.9e-05	77.7
Lipid Metabolism	TC	rs6999569	8	126475770	G	A	-0.062	0.001	1.0e-200	1.6e-03	2074.1
Lipid Metabolism	TC	rs67346026	8	126489818	G	A	0.051	0.005	9.8e-29	1.8e-04	123.7
Lipid Metabolism	TC	rs78350031	8	126518697	C	T	0.047	0.008	3.7e-09	2.7e-05	34.8
Lipid Metabolism	TC	rs149516560	8	126542126	T	C	0.048	0.007	1.3e-12	3.8e-05	50.3
Lipid Metabolism	TC	rs11787335	8	145044104	T	C	0.020	0.001	2.2e-46	1.6e-04	204.5
Lipid Metabolism	TC	rs3780181	9	2640759	G	A	-0.032	0.003	7.0e-34	1.1e-04	147.2
Lipid Metabolism	TC	rs7043836	9	2651714	G	T	-0.021	0.004	3.1e-09	2.7e-05	35.1
Lipid Metabolism	TC	rs820503	9	6667928	A	C	0.011	0.002	5.0e-09	2.6e-05	34.2
Lipid Metabolism	TC	rs113261688	9	15255361	T	C	0.016	0.003	3.9e-09	2.6e-05	34.7
Lipid Metabolism	TC	rs686030	9	15304782	A	C	0.026	0.002	6.9e-42	1.4e-04	183.9
Lipid Metabolism	TC	rs3927680	9	16887366	A	T	-0.012	0.001	5.3e-19	6.1e-05	79.3
Lipid Metabolism	TC	rs10964035	9	19218048	C	T	-0.017	0.002	7.2e-24	7.7e-05	101.5
Lipid Metabolism	TC	rs12554969	9	19261842	A	C	0.035	0.003	3.5e-44	1.5e-04	194.4
Lipid Metabolism	TC	rs10757273	9	22090301	A	C	-0.013	0.002	6.0e-19	6.9e-05	79.1
Lipid Metabolism	TC	rs62555904	9	33828873	G	A	-0.010	0.002	3.8e-09	2.6e-05	34.7
Lipid Metabolism	TC	rs60277997	9	78191921	T	C	-0.012	0.001	9.6e-18	5.7e-05	73.6
Lipid Metabolism	TC	rs1571791	9	78729213	C	T	-0.013	0.001	2.7e-21	6.8e-05	89.8
Lipid Metabolism	TC	rs9410206	9	91404728	C	T	-0.018	0.003	3.6e-11	3.3e-05	43.8
Lipid Metabolism	TC	rs2401637	9	100541473	C	T	0.008	0.001	7.1e-09	2.5e-05	33.5
Lipid Metabolism	TC	rs142260797	9	107273774	G	A	-0.066	0.011	8.3e-10	3.7e-05	37.7
Lipid Metabolism	TC	rs78431564	9	107331727	T	C	0.059	0.008	8.9e-13	3.9e-05	51.1
Lipid Metabolism	TC	rs145673272	9	107336060	A	G	0.084	0.015	4.0e-08	2.4e-05	30.1
Lipid Metabolism	TC	rs181721528	9	107394320	G	A	-0.200	0.027	1.6e-13	6.8e-05	54.4
Lipid Metabolism	TC	rs559092527	9	107437623	G	C	-0.138	0.023	1.5e-09	4.4e-05	36.5
Lipid Metabolism	TC	rs2777799	9	107559059	A	G	0.032	0.002	3.9e-48	1.6e-04	212.5
Lipid Metabolism	TC	rs55858182	9	107578675	T	C	-0.056	0.009	8.4e-11	3.3e-05	42.2
Lipid Metabolism	TC	rs4149311	9	107588777	T	C	0.039	0.002	1.8e-79	2.7e-04	356.3
Lipid Metabolism	TC	rs535908385	9	107609329	A	G	-0.144	0.025	1.2e-08	3.8e-05	32.4
Lipid Metabolism	TC	rs188308962	9	107620797	G	A	-0.264	0.016	3.4e-59	2.1e-04	263.2
Lipid Metabolism	TC	rs145771066	9	107630894	A	G	0.063	0.010	4.0e-10	3.4e-05	39.1
Lipid Metabolism	TC	rs534236518	9	107641324	T	C	0.215	0.039	3.5e-08	3.8e-05	30.4
Lipid Metabolism	TC	rs145183203	9	107646756	A	G	-0.135	0.020	1.4e-11	4.2e-05	45.7
Lipid Metabolism	TC	rs2740488	9	107661742	C	A	-0.053	0.002	1.0e-200	9.1e-04	1206.8
Lipid Metabolism	TC	rs545623893	9	107668375	G	C	-0.163	0.025	1.0e-10	4.6e-05	41.7
Lipid Metabolism	TC	rs76958885	9	107734726	A	G	0.033	0.005	1.7e-11	3.4e-05	45.3
Lipid Metabolism	TC	rs78773632	9	107910640	C	T	-0.018	0.003	2.1e-08	2.4e-05	31.4
Lipid Metabolism	TC	rs1831382	9	110535285	C	T	-0.009	0.001	2.7e-10	3.0e-05	39.9
Lipid Metabolism	TC	rs12000215	9	112223059	G	A	-0.009	0.001	3.2e-10	3.0e-05	39.6
Lipid Metabolism	TC	rs10733608	9	117148430	T	G	0.013	0.001	6.0e-21	6.7e-05	88.2
Lipid Metabolism	TC	rs10818578	9	124414751	G	A	0.010	0.002	4.3e-10	3.0e-05	38.9
Lipid Metabolism	TC	rs13289095	9	131466489	T	G	-0.028	0.002	3.5e-44	1.5e-04	194.4
Lipid Metabolism	TC	rs139884023	9	135867131	A	G	0.066	0.011	1.1e-09	2.9e-05	37.1
Lipid Metabolism	TC	rs33923294	9	136031817	C	G	-0.012	0.002	2.5e-09	2.7e-05	35.6
Lipid Metabolism	TC	rs2519093	9	136141870	T	C	0.068	0.002	1.0e-200	1.2e-03	1628.3

Phenotype	Trait	SNP	Chr	Position (GRCh37/hg19)	EA	NEA	BETA	SE	P-value	R2	F
Lipid Metabolism	TC	rs10901256	9	136163447	C	T	-0.021	0.002	9.5e-42	1.4e-04	183.2
Lipid Metabolism	TC	rs3780190	9	139099073	G	A	-0.013	0.001	1.3e-20	6.6e-05	86.7
Lipid Metabolism	TC	rs3812591	9	139341612	C	T	-0.010	0.002	7.1e-11	3.3e-05	42.5
Lipid Metabolism	TC	rs7921838	10	5247701	A	G	-0.018	0.002	1.3e-21	6.9e-05	91.3
Lipid Metabolism	TC	rs1976684	10	8090380	A	G	0.010	0.002	2.0e-10	3.1e-05	40.4
Lipid Metabolism	TC	rs7903259	10	17259642	G	C	0.021	0.001	2.2e-51	1.8e-04	227.4
Lipid Metabolism	TC	rs2992333	10	26727454	A	G	0.008	0.001	1.5e-08	2.4e-05	32.1
Lipid Metabolism	TC	rs2291428	10	45958856	C	G	0.020	0.002	6.3e-37	1.2e-04	161.2
Lipid Metabolism	TC	rs17011755	10	50287572	G	A	-0.009	0.002	2.9e-08	2.3e-05	30.8
Lipid Metabolism	TC	rs150770378	10	52616492	A	G	0.073	0.008	8.6e-21	7.5e-05	87.5
Lipid Metabolism	TC	rs1800450	10	54531235	T	C	0.011	0.002	1.3e-08	2.5e-05	32.4
Lipid Metabolism	TC	rs10761750	10	65128619	A	G	0.008	0.001	1.3e-08	2.5e-05	32.4
Lipid Metabolism	TC	rs71478838	10	70420998	A	G	-0.014	0.002	8.9e-10	3.3e-05	37.6
Lipid Metabolism	TC	rs17476364	10	71094504	C	T	-0.030	0.002	1.2e-41	1.4e-04	182.7
Lipid Metabolism	TC	rs1782651	10	81076477	T	C	0.012	0.002	6.8e-15	4.9e-05	60.6
Lipid Metabolism	TC	rs7080009	10	82269461	C	T	0.011	0.002	6.8e-11	3.3e-05	42.6
Lipid Metabolism	TC	rs11599018	10	88538345	A	C	-0.008	0.001	1.2e-08	2.5e-05	32.5
Lipid Metabolism	TC	rs638846	10	89804585	G	C	-0.028	0.004	4.3e-14	4.3e-05	57.0
Lipid Metabolism	TC	rs2250781	10	91007470	A	C	-0.011	0.001	9.9e-16	4.9e-05	64.4
Lipid Metabolism	TC	rs118052097	10	94663148	G	A	-0.021	0.004	4.7e-08	2.3e-05	29.8
Lipid Metabolism	TC	rs6583855	10	94843032	C	A	-0.016	0.001	5.2e-33	1.1e-04	143.3
Lipid Metabolism	TC	rs10786156	10	96014622	G	C	-0.012	0.001	2.5e-18	5.8e-05	76.3
Lipid Metabolism	TC	rs11597321	10	97511949	G	A	-0.008	0.001	4.1e-08	2.3e-05	30.1
Lipid Metabolism	TC	rs17668357	10	102003906	C	G	0.016	0.001	1.9e-32	1.1e-04	140.7
Lipid Metabolism	TC	rs2803621	10	113939584	A	G	-0.026	0.001	1.0e-67	2.3e-04	302.3
Lipid Metabolism	TC	rs12413488	10	113986840	A	G	-0.021	0.002	1.2e-32	1.1e-04	141.5
Lipid Metabolism	TC	rs75931981	10	114104053	T	C	-0.016	0.003	5.2e-10	2.9e-05	38.6
Lipid Metabolism	TC	rs113999985	10	114711677	T	C	0.011	0.002	2.6e-12	4.6e-05	48.9
Lipid Metabolism	TC	rs2286779	10	118394551	C	G	0.012	0.001	1.5e-20	6.6e-05	86.3
Lipid Metabolism	TC	rs2475313	10	121646618	G	C	0.009	0.001	1.2e-09	2.8e-05	36.9
Lipid Metabolism	TC	rs2130779	10	122879732	T	G	-0.017	0.003	2.9e-10	3.0e-05	39.7
Lipid Metabolism	TC	rs7904973	10	124693587	T	G	0.020	0.001	1.4e-50	1.7e-04	223.8
Lipid Metabolism	TC	rs35338528	11	1074635	G	T	-0.022	0.004	1.7e-09	2.8e-05	36.2
Lipid Metabolism	TC	rs450244	11	2940492	C	T	0.024	0.002	2.7e-24	7.9e-05	103.4
Lipid Metabolism	TC	rs188947737	11	2947009	T	C	-0.039	0.005	1.3e-13	4.2e-05	54.9
Lipid Metabolism	TC	rs112830587	11	3127829	G	A	-0.060	0.010	3.2e-09	2.7e-05	35.0
Lipid Metabolism	TC	rs188513096	11	4993424	A	T	-0.209	0.025	5.3e-17	7.5e-05	70.2
Lipid Metabolism	TC	rs17305868	11	5701883	G	A	-0.010	0.001	4.5e-14	4.3e-05	56.9
Lipid Metabolism	TC	rs11022130	11	12071547	G	A	0.010	0.002	1.1e-10	3.2e-05	41.6
Lipid Metabolism	TC	rs214093	11	17294788	G	C	0.009	0.001	8.4e-12	3.5e-05	46.7
Lipid Metabolism	TC	rs2084220	11	18656932	T	C	0.021	0.002	1.4e-44	1.5e-04	196.2
Lipid Metabolism	TC	rs141181296	11	46537370	T	C	-0.040	0.004	6.1e-25	8.1e-05	106.4
Lipid Metabolism	TC	rs78506520	11	47102183	A	G	0.019	0.003	4.4e-11	3.3e-05	43.4
Lipid Metabolism	TC	rs1228044	11	48011180	G	A	0.018	0.002	5.2e-30	1.0e-04	129.5
Lipid Metabolism	TC	rs116314949	11	49247978	T	C	-0.032	0.004	1.7e-16	5.2e-05	67.9
Lipid Metabolism	TC	rs78608491	11	49782913	T	C	0.030	0.004	7.1e-14	4.3e-05	56.0
Lipid Metabolism	TC	rs118146000	11	50364638	A	G	-0.034	0.004	1.3e-16	5.3e-05	68.4
Lipid Metabolism	TC	rs79661854	11	51263367	A	T	0.033	0.005	2.9e-11	6.8e-05	44.3
Lipid Metabolism	TC	rs140827054	11	51471514	T	C	-0.033	0.004	2.6e-15	4.8e-05	62.6
Lipid Metabolism	TC	rs117628555	11	55135876	T	C	0.030	0.004	2.0e-12	5.4e-05	49.4
Lipid Metabolism	TC	rs74369367	11	55329613	G	A	-0.032	0.004	1.2e-14	4.5e-05	59.5
Lipid Metabolism	TC	rs75230704	11	56225592	A	G	0.029	0.004	4.0e-15	4.7e-05	61.7
Lipid Metabolism	TC	rs116875968	11	56334924	C	T	-0.037	0.005	2.5e-13	4.1e-05	53.6
Lipid Metabolism	TC	rs950802	11	60152584	A	G	-0.010	0.001	2.1e-12	3.8e-05	49.4
Lipid Metabolism	TC	rs572537530	11	61529656	C	G	-0.043	0.008	2.6e-08	2.4e-05	31.0
Lipid Metabolism	TC	rs102275	11	61557803	C	T	-0.041	0.001	6.7e-193	6.7e-04	877.8

Phenotype	Trait	SNP	Chr	Position (GRCh37/hg19)	EA	NEA	BETA	SE	P-value	R2	F
Lipid Metabolism	TC	rs17156442	11	61614023	T	C	-0.023	0.003	4.4e-14	4.3e-05	57.0
Lipid Metabolism	TC	rs2727260	11	61712242	T	C	0.021	0.002	1.8e-21	6.9e-05	90.6
Lipid Metabolism	TC	rs77631946	11	63688374	A	C	-0.019	0.002	2.0e-15	4.8e-05	63.1
Lipid Metabolism	TC	rs4601790	11	65353906	G	A	0.011	0.002	2.1e-13	4.1e-05	53.9
Lipid Metabolism	TC	rs117777720	11	66196384	T	C	-0.014	0.002	5.5e-18	5.7e-05	74.7
Lipid Metabolism	TC	rs12803635	11	67224391	A	G	-0.019	0.003	7.3e-10	2.9e-05	37.9
Lipid Metabolism	TC	rs72997616	11	75474195	A	C	-0.024	0.002	4.0e-26	8.5e-05	111.8
Lipid Metabolism	TC	rs4945274	11	78109260	A	G	-0.015	0.002	6.5e-16	4.9e-05	65.3
Lipid Metabolism	TC	rs12575636	11	95311260	G	T	-0.012	0.002	6.1e-13	3.9e-05	51.8
Lipid Metabolism	TC	rs12295353	11	95470147	C	T	-0.009	0.001	7.3e-12	3.6e-05	47.0
Lipid Metabolism	TC	rs10791660	11	103871039	A	C	-0.014	0.002	1.8e-16	5.1e-05	67.9
Lipid Metabolism	TC	rs61906105	11	116550528	G	A	0.069	0.003	3.8e-110	3.8e-04	497.2
Lipid Metabolism	TC	rs76814754	11	116557959	T	C	-0.027	0.005	2.2e-08	2.4e-05	31.3
Lipid Metabolism	TC	rs148945243	11	116601062	T	C	-0.082	0.006	6.3e-39	1.3e-04	170.3
Lipid Metabolism	TC	rs964184	11	116648917	C	G	-0.100	0.002	1.0e-200	2.0e-03	2660.8
Lipid Metabolism	TC	rs3135507	11	116661488	T	C	0.039	0.004	8.0e-21	6.7e-05	87.6
Lipid Metabolism	TC	rs45611741	11	116663128	T	C	0.144	0.009	9.4e-61	2.1e-04	270.4
Lipid Metabolism	TC	rs12721030	11	116705278	T	C	0.035	0.002	5.7e-92	3.1e-04	413.7
Lipid Metabolism	TC	rs140301504	11	116708750	C	T	-0.066	0.010	6.3e-12	3.7e-05	47.2
Lipid Metabolism	TC	rs183737298	11	116712329	A	G	0.104	0.018	2.6e-09	3.9e-05	35.4
Lipid Metabolism	TC	rs144578147	11	116830092	C	G	-0.049	0.007	1.5e-13	4.2e-05	54.6
Lipid Metabolism	TC	rs185000149	11	116865588	C	A	-0.075	0.011	2.8e-11	3.4e-05	44.3
Lipid Metabolism	TC	rs143595798	11	116876446	G	A	0.095	0.012	1.6e-15	5.0e-05	63.5
Lipid Metabolism	TC	rs566419460	11	116989101	A	G	0.117	0.020	7.5e-09	3.7e-05	33.4
Lipid Metabolism	TC	rs146226340	11	117113666	G	A	0.119	0.021	1.5e-08	5.2e-05	32.1
Lipid Metabolism	TC	rs117619191	11	117248283	C	T	0.031	0.006	3.8e-08	2.3e-05	30.3
Lipid Metabolism	TC	rs4639966	11	118573519	C	T	0.014	0.002	2.0e-20	6.5e-05	85.8
Lipid Metabolism	TC	rs116993355	11	119012962	C	T	-0.017	0.003	7.2e-11	3.2e-05	42.5
Lipid Metabolism	TC	rs10750217	11	122531574	G	A	0.022	0.001	2.8e-59	2.0e-04	263.6
Lipid Metabolism	TC	rs4055121	11	126232337	T	C	0.036	0.002	7.7e-74	2.5e-04	330.4
Lipid Metabolism	TC	rs76054236	11	126232615	A	G	0.053	0.008	8.8e-11	3.2e-05	42.1
Lipid Metabolism	TC	rs11820880	11	134268453	C	T	-0.010	0.002	3.8e-08	2.3e-05	30.2
Lipid Metabolism	TC	rs35882350	12	623129	G	A	0.016	0.002	1.6e-25	8.4e-05	109.0
Lipid Metabolism	TC	rs142394917	12	4401472	A	G	-0.083	0.011	3.5e-15	4.8e-05	61.9
Lipid Metabolism	TC	rs35398641	12	6514969	T	C	-0.009	0.002	4.2e-10	3.1e-05	39.0
Lipid Metabolism	TC	rs2302368	12	6859797	C	T	-0.008	0.001	2.2e-08	2.4e-05	31.3
Lipid Metabolism	TC	rs12824533	12	11791685	G	A	0.008	0.001	3.6e-08	2.4e-05	30.3
Lipid Metabolism	TC	rs35756741	12	12868701	T	C	0.014	0.002	3.5e-10	3.0e-05	39.4
Lipid Metabolism	TC	rs75667995	12	25409070	C	T	-0.033	0.003	2.4e-36	1.2e-04	158.5
Lipid Metabolism	TC	rs11048645	12	26829961	A	T	-0.009	0.001	5.2e-11	3.3e-05	43.1
Lipid Metabolism	TC	rs10843390	12	29496991	T	C	-0.014	0.001	4.8e-22	7.1e-05	93.2
Lipid Metabolism	TC	rs11175540	12	40586295	A	T	0.023	0.003	2.3e-17	5.6e-05	71.8
Lipid Metabolism	TC	rs12828693	12	46385848	T	C	0.013	0.002	3.5e-14	4.3e-05	57.4
Lipid Metabolism	TC	rs77180961	12	50060818	C	G	-0.037	0.006	2.2e-11	3.4e-05	44.8
Lipid Metabolism	TC	rs2250751	12	51106178	A	G	-0.019	0.001	2.4e-39	1.3e-04	172.2
Lipid Metabolism	TC	rs10876171	12	51793821	A	G	-0.015	0.001	4.0e-27	9.1e-05	116.3
Lipid Metabolism	TC	rs703848	12	57428353	G	A	0.010	0.001	1.2e-10	3.2e-05	41.4
Lipid Metabolism	TC	rs74093304	12	59923805	A	G	0.013	0.002	5.3e-10	2.9e-05	38.6
Lipid Metabolism	TC	rs61754230	12	72179446	T	C	0.047	0.006	1.8e-17	6.2e-05	72.3
Lipid Metabolism	TC	rs12306780	12	89921860	T	A	0.009	0.001	5.2e-11	3.3e-05	43.1
Lipid Metabolism	TC	rs4760390	12	92815669	T	C	0.008	0.001	3.7e-10	3.0e-05	39.3
Lipid Metabolism	TC	rs11837065	12	100859983	T	C	-0.012	0.001	6.2e-17	5.4e-05	69.9
Lipid Metabolism	TC	rs11610264	12	100932375	C	T	0.011	0.002	1.8e-12	3.9e-05	49.7
Lipid Metabolism	TC	rs5742629	12	102857263	C	T	0.009	0.002	4.2e-09	2.6e-05	34.5
Lipid Metabolism	TC	rs113131186	12	104921693	G	C	0.011	0.002	1.3e-08	2.5e-05	32.3
Lipid Metabolism	TC	rs192645005	12	109452628	A	G	0.045	0.007	8.8e-11	3.2e-05	42.1

Phenotype	Trait	SNP	Chr	Position (GRCh37/hg19)	EA	NEA	BETA	SE	P-value	R2	F
Lipid Metabolism	TC	rs1075467	12	109986935	A	G	0.018	0.001	5.5e-41	1.4e-04	179.7
Lipid Metabolism	TC	rs12819646	12	111319847	T	C	0.010	0.002	4.2e-09	2.6e-05	34.5
Lipid Metabolism	TC	rs569106137	12	111597874	A	G	-0.140	0.024	5.0e-09	4.7e-05	34.2
Lipid Metabolism	TC	rs534664780	12	111808379	C	T	-0.134	0.021	1.2e-10	5.1e-05	41.4
Lipid Metabolism	TC	rs546459162	12	111865264	G	C	0.049	0.007	1.5e-12	4.7e-05	50.1
Lipid Metabolism	TC	rs4766578	12	111904371	A	T	0.025	0.001	2.4e-75	2.6e-04	337.3
Lipid Metabolism	TC	rs570074821	12	111926961	G	A	-0.143	0.022	1.4e-10	5.4e-05	41.1
Lipid Metabolism	TC	rs543418213	12	111997902	A	G	-0.130	0.021	1.1e-09	4.7e-05	37.2
Lipid Metabolism	TC	rs535267270	12	112852124	T	C	-0.151	0.024	3.0e-10	5.2e-05	39.7
Lipid Metabolism	TC	rs58116261	12	112935344	T	C	0.020	0.002	2.3e-16	5.1e-05	67.3
Lipid Metabolism	TC	rs233722	12	113031474	A	G	0.014	0.001	7.5e-24	7.7e-05	101.4
Lipid Metabolism	TC	rs7298194	12	115902571	A	T	0.008	0.001	2.0e-08	2.4e-05	31.5
Lipid Metabolism	TC	rs1634536	12	120856874	A	T	-0.010	0.001	1.6e-14	4.5e-05	58.9
Lipid Metabolism	TC	rs541619	12	121096987	G	A	0.014	0.002	4.4e-19	6.1e-05	79.7
Lipid Metabolism	TC	rs2244608	12	121416988	G	A	0.035	0.001	6.7e-132	4.5e-04	597.2
Lipid Metabolism	TC	rs895959	12	122320352	T	C	-0.012	0.002	2.3e-10	3.0e-05	40.2
Lipid Metabolism	TC	rs7972811	12	123820780	T	G	-0.034	0.002	2.0e-49	1.7e-04	218.5
Lipid Metabolism	TC	rs12367493	12	125071709	T	A	-0.014	0.001	6.9e-21	6.7e-05	87.9
Lipid Metabolism	TC	rs61933425	12	125237978	A	G	-0.026	0.002	5.7e-27	8.8e-05	115.7
Lipid Metabolism	TC	rs921919	12	125265201	A	G	-0.019	0.001	5.6e-40	1.4e-04	175.1
Lipid Metabolism	TC	rs530697899	12	125410268	G	A	0.040	0.007	1.8e-08	2.8e-05	31.7
Lipid Metabolism	TC	rs75588192	12	133048600	A	G	0.018	0.002	2.2e-19	7.0e-05	81.0
Lipid Metabolism	TC	rs145096717	13	28761592	A	G	-0.045	0.007	2.8e-10	3.1e-05	39.8
Lipid Metabolism	TC	rs9508721	13	30964283	T	C	0.020	0.003	1.2e-11	3.5e-05	46.0
Lipid Metabolism	TC	rs9315148	13	32481926	T	C	-0.010	0.002	1.1e-11	3.5e-05	46.1
Lipid Metabolism	TC	rs2238162	13	32959199	T	C	-0.017	0.001	4.1e-35	1.2e-04	152.9
Lipid Metabolism	TC	rs208426	13	33191371	C	T	-0.010	0.001	1.0e-12	3.9e-05	50.8
Lipid Metabolism	TC	rs17532371	13	41635301	G	C	-0.027	0.003	1.6e-24	7.9e-05	104.4
Lipid Metabolism	TC	rs9568208	13	50027333	C	T	0.014	0.002	5.1e-10	2.9e-05	38.6
Lipid Metabolism	TC	rs201798	13	50954721	A	G	-0.010	0.001	1.8e-12	3.8e-05	49.6
Lipid Metabolism	TC	rs9592980	13	74855425	A	G	0.011	0.001	1.8e-16	5.2e-05	67.8
Lipid Metabolism	TC	rs4643195	13	76103840	A	G	0.009	0.002	1.2e-09	2.8e-05	37.0
Lipid Metabolism	TC	rs9544591	13	78315135	A	G	0.015	0.003	2.9e-08	2.3e-05	30.8
Lipid Metabolism	TC	rs7336371	13	95221268	A	G	0.009	0.002	2.5e-09	2.7e-05	35.6
Lipid Metabolism	TC	rs4150336	13	103519251	T	C	0.017	0.003	9.0e-09	2.5e-05	33.0
Lipid Metabolism	TC	rs150861794	13	109003805	T	C	-0.028	0.005	3.0e-08	2.3e-05	30.7
Lipid Metabolism	TC	rs4771674	13	111039070	G	A	0.014	0.001	1.5e-23	7.7e-05	100.1
Lipid Metabolism	TC	rs7140110	13	114544024	C	T	0.026	0.001	3.3e-66	2.3e-04	295.4
Lipid Metabolism	TC	rs9604570	13	114631940	G	A	-0.021	0.002	3.7e-31	1.0e-04	134.8
Lipid Metabolism	TC	rs74036552	14	21486590	T	C	-0.033	0.006	2.8e-08	2.4e-05	30.9
Lipid Metabolism	TC	rs111988830	14	24666856	T	C	-0.010	0.002	3.5e-08	2.3e-05	30.4
Lipid Metabolism	TC	rs11621792	14	24871926	T	C	0.021	0.001	2.1e-53	1.8e-04	236.6
Lipid Metabolism	TC	rs139262716	14	31725111	A	G	-0.039	0.005	1.4e-14	4.5e-05	59.2
Lipid Metabolism	TC	rs10133108	14	35185617	G	A	0.009	0.001	1.0e-11	3.5e-05	46.3
Lipid Metabolism	TC	rs11626768	14	39686917	A	T	0.011	0.002	5.2e-14	4.3e-05	56.7
Lipid Metabolism	TC	rs17123116	14	51371081	C	A	0.029	0.005	9.3e-09	2.5e-05	33.0
Lipid Metabolism	TC	rs12897637	14	64239351	C	T	0.018	0.002	1.6e-23	7.6e-05	99.9
Lipid Metabolism	TC	rs945309	14	70178981	A	G	-0.012	0.002	4.7e-08	2.3e-05	29.8
Lipid Metabolism	TC	rs8008400	14	70862888	T	A	0.019	0.002	4.1e-23	7.4e-05	98.1
Lipid Metabolism	TC	rs10047892	14	75111346	T	C	-0.009	0.001	1.1e-10	3.2e-05	41.6
Lipid Metabolism	TC	rs11159104	14	75293374	G	A	-0.012	0.001	1.2e-18	5.9e-05	77.7
Lipid Metabolism	TC	rs35802157	14	90066634	C	T	-0.009	0.001	1.5e-10	3.1e-05	41.0
Lipid Metabolism	TC	rs188439677	14	94771094	G	C	0.064	0.011	1.5e-08	2.5e-05	32.0
Lipid Metabolism	TC	rs17580	14	94847262	A	T	0.047	0.003	4.5e-42	1.4e-04	184.7
Lipid Metabolism	TC	rs1004572	14	101178330	A	G	0.011	0.002	3.9e-09	2.6e-05	34.7
Lipid Metabolism	TC	rs67004399	14	102263973	T	C	0.011	0.002	1.6e-08	2.4e-05	31.9

Phenotype	Trait	SNP	Chr	Position (GRCh37/hg19)	EA	NEA	BETA	SE	P-value	R2	F
Lipid Metabolism	TC	rs2210315	14	102764820	T	C	-0.011	0.002	2.3e-10	3.0e-05	40.2
Lipid Metabolism	TC	rs8015761	14	104353308	C	T	-0.008	0.001	8.5e-10	2.9e-05	37.6
Lipid Metabolism	TC	rs45490496	14	105272678	T	A	-0.008	0.001	1.9e-08	2.5e-05	31.6
Lipid Metabolism	TC	rs2413926	15	49336477	A	T	-0.012	0.001	2.9e-18	5.8e-05	75.9
Lipid Metabolism	TC	rs72740245	15	53022588	T	C	0.020	0.003	5.0e-11	3.3e-05	43.2
Lipid Metabolism	TC	rs79101008	15	53076370	A	C	-0.014	0.002	4.8e-10	2.9e-05	38.7
Lipid Metabolism	TC	rs79391862	15	53739426	C	A	-0.059	0.005	5.8e-31	1.0e-04	133.9
Lipid Metabolism	TC	rs72749502	15	57297078	T	A	0.032	0.003	3.8e-33	1.1e-04	143.9
Lipid Metabolism	TC	rs552730008	15	58199779	A	G	0.086	0.014	9.0e-10	5.1e-05	37.5
Lipid Metabolism	TC	rs4775024	15	58502135	G	A	-0.015	0.002	3.2e-16	5.1e-05	66.7
Lipid Metabolism	TC	rs2899623	15	58579644	C	T	-0.039	0.002	8.6e-90	3.1e-04	403.7
Lipid Metabolism	TC	rs7350789	15	58679668	A	G	0.053	0.001	1.0e-200	1.1e-03	1450.7
Lipid Metabolism	TC	rs573241250	15	58679688	C	G	0.062	0.008	7.5e-16	5.0e-05	65.0
Lipid Metabolism	TC	rs1077835	15	58723426	G	A	0.065	0.002	1.0e-200	1.2e-03	1600.5
Lipid Metabolism	TC	rs17190566	15	58759940	T	A	0.017	0.002	5.3e-14	4.3e-05	56.6
Lipid Metabolism	TC	rs113298164	15	58855748	T	C	0.140	0.012	3.3e-32	1.2e-04	139.6
Lipid Metabolism	TC	rs62011285	15	63791063	C	T	0.017	0.001	5.9e-33	1.1e-04	143.0
Lipid Metabolism	TC	rs4776342	15	67418391	G	A	0.010	0.002	4.2e-10	3.0e-05	39.0
Lipid Metabolism	TC	rs4243086	15	74697084	A	G	-0.011	0.002	1.1e-09	2.8e-05	37.1
Lipid Metabolism	TC	rs6495122	15	75125645	C	A	0.012	0.001	1.1e-17	5.6e-05	73.4
Lipid Metabolism	TC	rs2458250	15	77270131	T	A	-0.008	0.001	3.1e-09	2.7e-05	35.1
Lipid Metabolism	TC	rs11632720	15	79169499	T	C	0.008	0.001	2.8e-08	2.4e-05	30.8
Lipid Metabolism	TC	rs8043389	15	89069457	T	G	0.010	0.001	1.1e-11	3.5e-05	46.2
Lipid Metabolism	TC	rs6416554	15	91138290	C	T	-0.010	0.002	4.5e-08	2.3e-05	29.9
Lipid Metabolism	TC	rs12898856	15	96227920	T	C	-0.007	0.001	3.3e-08	2.3e-05	30.5
Lipid Metabolism	TC	rs8023580	15	96708291	C	T	0.009	0.001	2.6e-09	2.7e-05	35.5
Lipid Metabolism	TC	rs9672549	15	99180105	C	T	0.008	0.002	3.8e-08	2.6e-05	30.3
Lipid Metabolism	TC	rs576644950	15	101744507	T	C	-0.059	0.011	3.2e-08	2.4e-05	30.6
Lipid Metabolism	TC	rs74002781	16	2146288	T	C	0.015	0.002	4.1e-11	3.4e-05	43.6
Lipid Metabolism	TC	rs2113260	16	11455993	C	T	-0.011	0.001	5.5e-14	4.3e-05	56.6
Lipid Metabolism	TC	rs57900082	16	11679101	G	A	0.018	0.002	3.1e-22	7.3e-05	94.0
Lipid Metabolism	TC	rs1704531	16	14381078	T	C	-0.010	0.002	6.6e-10	3.5e-05	38.1
Lipid Metabolism	TC	rs55734390	16	15781092	T	G	0.010	0.002	4.2e-08	2.3e-05	30.0
Lipid Metabolism	TC	rs11640942	16	19919946	T	C	0.014	0.002	2.3e-12	3.7e-05	49.2
Lipid Metabolism	TC	rs7186298	16	21088031	T	C	0.008	0.001	3.6e-10	3.0e-05	39.3
Lipid Metabolism	TC	rs28888764	16	28829228	G	A	-0.009	0.001	1.5e-09	2.8e-05	36.5
Lipid Metabolism	TC	rs8062719	16	31002664	G	A	-0.012	0.001	5.4e-18	5.7e-05	74.7
Lipid Metabolism	TC	rs9923357	16	31286014	A	C	-0.012	0.002	8.5e-09	2.5e-05	33.2
Lipid Metabolism	TC	rs113710278	16	56295380	T	C	0.033	0.006	5.3e-09	2.6e-05	34.1
Lipid Metabolism	TC	rs147643198	16	56874133	T	C	-0.076	0.013	1.7e-09	2.9e-05	36.3
Lipid Metabolism	TC	rs141938931	16	56901445	G	A	-0.077	0.012	5.6e-10	3.0e-05	38.5
Lipid Metabolism	TC	rs247615	16	56984763	G	A	-0.017	0.002	2.3e-23	7.5e-05	99.2
Lipid Metabolism	TC	rs247616	16	56989590	T	C	0.041	0.001	1.3e-176	6.1e-04	802.8
Lipid Metabolism	TC	rs185197208	16	57055635	A	G	-0.050	0.008	7.3e-11	3.3e-05	42.4
Lipid Metabolism	TC	rs75257814	16	57118381	C	T	-0.024	0.003	1.4e-15	4.9e-05	63.8
Lipid Metabolism	TC	rs28382814	16	66971780	T	C	-0.027	0.004	1.2e-13	4.2e-05	55.1
Lipid Metabolism	TC	rs143264468	16	67253519	C	T	-0.372	0.050	9.3e-14	9.7e-05	55.5
Lipid Metabolism	TC	rs1109166	16	67977382	C	T	0.017	0.002	3.8e-21	6.7e-05	89.1
Lipid Metabolism	TC	rs186429262	16	68239482	G	A	-0.321	0.053	9.4e-10	5.7e-05	37.4
Lipid Metabolism	TC	rs562097375	16	68246413	A	G	-0.388	0.049	1.2e-15	1.1e-04	64.1
Lipid Metabolism	TC	rs538690834	16	68284105	T	C	-0.377	0.050	7.6e-14	1.4e-04	55.9
Lipid Metabolism	TC	rs11540992	16	69376269	A	G	-0.027	0.003	2.3e-15	4.8e-05	62.8
Lipid Metabolism	TC	rs181501802	16	70067440	A	G	0.024	0.003	9.3e-16	4.9e-05	64.6
Lipid Metabolism	TC	rs374464308	16	70080280	T	C	0.052	0.009	1.6e-08	3.0e-05	31.9
Lipid Metabolism	TC	rs74340438	16	70807284	T	C	0.027	0.004	8.9e-14	4.2e-05	55.6
Lipid Metabolism	TC	rs11646091	16	70825649	C	T	-0.023	0.003	2.3e-11	3.4e-05	44.7

Phenotype	Trait	SNP	Chr	Position (GRCh37/hg19)	EA	NEA	BETA	SE	P-value	R2	F
Lipid Metabolism	TC	rs56212732	16	70930370	T	C	-0.029	0.003	6.1e-28	9.2e-05	120.1
Lipid Metabolism	TC	rs71403812	16	71014962	A	G	0.028	0.004	3.4e-13	4.1e-05	52.9
Lipid Metabolism	TC	rs12921168	16	71920250	T	C	-0.032	0.005	9.7e-09	2.5e-05	32.9
Lipid Metabolism	TC	rs193210477	16	71992460	A	C	-0.066	0.008	5.1e-15	5.3e-05	61.2
Lipid Metabolism	TC	rs141154498	16	72030455	A	G	-0.069	0.011	3.0e-10	3.1e-05	39.7
Lipid Metabolism	TC	rs12924886	16	72075593	T	A	0.055	0.002	1.0e-200	8.0e-04	1053.3
Lipid Metabolism	TC	rs563195118	16	72087343	T	C	-0.105	0.015	1.4e-11	5.1e-05	45.7
Lipid Metabolism	TC	rs144130656	16	72136549	A	G	-0.047	0.008	3.9e-09	2.7e-05	34.7
Lipid Metabolism	TC	rs141181263	16	72140548	G	A	-0.042	0.007	4.1e-09	2.6e-05	34.6
Lipid Metabolism	TC	rs11647069	16	72155237	C	T	0.024	0.001	2.2e-61	2.1e-04	273.3
Lipid Metabolism	TC	rs142985659	16	72171853	C	T	-0.052	0.007	7.1e-13	4.4e-05	51.5
Lipid Metabolism	TC	rs188598167	16	72207445	C	T	-0.066	0.009	1.4e-13	4.2e-05	54.7
Lipid Metabolism	TC	rs113368886	16	72303317	C	T	-0.034	0.002	1.2e-44	1.5e-04	196.5
Lipid Metabolism	TC	rs75225488	16	72705938	A	G	-0.088	0.016	2.0e-08	2.6e-05	31.5
Lipid Metabolism	TC	rs191079161	16	76104525	G	A	0.383	0.067	8.8e-09	7.4e-05	33.1
Lipid Metabolism	TC	rs1396703	16	79358608	T	C	0.009	0.001	1.2e-10	3.2e-05	41.5
Lipid Metabolism	TC	rs1862719	16	79504057	G	A	-0.012	0.002	2.9e-13	4.1e-05	53.2
Lipid Metabolism	TC	rs73575083	16	79747855	G	A	0.009	0.001	9.7e-10	2.8e-05	37.4
Lipid Metabolism	TC	rs874834	16	81486276	A	G	0.008	0.001	1.3e-09	2.8e-05	36.9
Lipid Metabolism	TC	rs11149612	16	83980965	T	C	-0.016	0.001	1.1e-28	9.8e-05	123.4
Lipid Metabolism	TC	rs16975110	16	85028352	C	T	-0.010	0.002	1.2e-09	2.8e-05	37.0
Lipid Metabolism	TC	rs4843770	16	88027725	C	T	-0.009	0.001	9.6e-10	2.8e-05	37.4
Lipid Metabolism	TC	rs904789	16	88528722	G	A	-0.012	0.001	8.1e-16	5.0e-05	64.8
Lipid Metabolism	TC	rs74035509	16	88567333	T	C	0.025	0.003	1.9e-20	6.6e-05	85.9
Lipid Metabolism	TC	rs11078597	17	1618363	C	T	0.013	0.002	3.6e-14	4.4e-05	57.4
Lipid Metabolism	TC	rs4480845	17	1958609	C	T	-0.012	0.001	1.9e-18	5.9e-05	76.8
Lipid Metabolism	TC	rs537438344	17	4436098	C	G	-0.104	0.016	3.1e-10	4.7e-05	39.6
Lipid Metabolism	TC	rs4533344	17	4627181	T	C	0.012	0.002	4.2e-08	2.3e-05	30.1
Lipid Metabolism	TC	rs11656732	17	4670778	T	C	0.016	0.001	1.1e-30	1.0e-04	132.6
Lipid Metabolism	TC	rs140779352	17	4672983	A	C	-0.115	0.009	8.6e-41	1.4e-04	178.9
Lipid Metabolism	TC	rs141001600	17	4691309	C	T	0.054	0.010	4.9e-08	2.3e-05	29.8
Lipid Metabolism	TC	rs188002707	17	6839871	T	C	-0.265	0.033	2.4e-15	6.8e-05	62.7
Lipid Metabolism	TC	rs577492325	17	6918254	C	A	-0.216	0.032	1.6e-11	5.3e-05	45.4
Lipid Metabolism	TC	rs186021206	17	7069412	A	G	-0.270	0.010	8.0e-153	5.4e-04	693.4
Lipid Metabolism	TC	rs72837687	17	7073747	A	G	-0.030	0.002	1.4e-62	2.3e-04	278.8
Lipid Metabolism	TC	rs62059170	17	7155056	C	T	-0.009	0.001	7.6e-10	2.9e-05	37.9
Lipid Metabolism	TC	rs150438405	17	7443059	C	G	0.042	0.007	9.9e-11	3.2e-05	41.8
Lipid Metabolism	TC	rs12944954	17	7485131	G	A	0.033	0.005	1.9e-13	4.1e-05	54.1
Lipid Metabolism	TC	rs62059826	17	7485583	T	C	-0.015	0.002	4.0e-17	5.4e-05	70.8
Lipid Metabolism	TC	rs9908765	17	8214427	A	G	0.015	0.001	2.1e-28	9.3e-05	122.2
Lipid Metabolism	TC	rs2654722	17	9539316	T	C	-0.010	0.002	4.5e-08	2.3e-05	29.9
Lipid Metabolism	TC	rs62067561	17	9785912	T	C	0.011	0.001	4.5e-15	4.7e-05	61.5
Lipid Metabolism	TC	rs12941301	17	17549259	G	A	-0.008	0.001	7.8e-10	2.9e-05	37.8
Lipid Metabolism	TC	rs111589301	17	18116773	A	G	0.014	0.002	6.0e-17	5.3e-05	70.0
Lipid Metabolism	TC	rs4795434	17	26716917	T	G	0.008	0.001	1.4e-09	2.8e-05	36.6
Lipid Metabolism	TC	rs7216105	17	27048296	T	C	-0.026	0.002	8.2e-28	9.1e-05	119.5
Lipid Metabolism	TC	rs66777321	17	27692362	A	G	-0.015	0.002	5.6e-15	4.6e-05	61.0
Lipid Metabolism	TC	rs8067252	17	29263700	T	C	0.013	0.002	5.2e-16	5.0e-05	65.7
Lipid Metabolism	TC	rs2952996	17	29416083	C	T	0.019	0.002	3.4e-34	1.1e-04	148.6
Lipid Metabolism	TC	rs6505242	17	29750139	G	A	0.010	0.002	2.3e-08	2.4e-05	31.2
Lipid Metabolism	TC	rs118073397	17	33390766	C	T	-0.058	0.011	4.7e-08	2.3e-05	29.8
Lipid Metabolism	TC	rs4795393	17	37893484	T	C	0.014	0.001	1.4e-23	7.7e-05	100.2
Lipid Metabolism	TC	rs2062214	17	40593005	C	G	-0.014	0.002	9.2e-14	4.3e-05	55.5
Lipid Metabolism	TC	rs56017239	17	40989002	G	A	0.025	0.004	1.1e-10	3.2e-05	41.7
Lipid Metabolism	TC	rs72836561	17	41926126	T	C	-0.036	0.004	1.5e-20	6.6e-05	86.4
Lipid Metabolism	TC	rs197912	17	44989888	A	T	0.009	0.001	1.7e-09	2.8e-05	36.3

Phenotype	Trait	SNP	Chr	Position (GRCh37/hg19)	EA	NEA	BETA	SE	P-value	R2	F
Lipid Metabolism	TC	rs12603290	17	45650196	C	T	-0.025	0.001	3.7e-75	2.6e-04	336.5
Lipid Metabolism	TC	rs2428608	17	46040795	T	C	0.012	0.001	2.7e-19	6.1e-05	80.6
Lipid Metabolism	TC	rs2326016	17	46694415	G	C	-0.010	0.001	2.1e-12	3.9e-05	49.4
Lipid Metabolism	TC	rs41524846	17	49252238	C	T	-0.010	0.002	2.8e-10	3.0e-05	39.8
Lipid Metabolism	TC	rs894623	17	55419694	T	C	0.008	0.001	3.7e-08	2.3e-05	30.3
Lipid Metabolism	TC	rs116985917	17	56409956	C	T	-0.015	0.002	1.4e-09	2.8e-05	36.6
Lipid Metabolism	TC	rs1292061	17	57911230	G	A	0.008	0.001	5.7e-09	2.6e-05	33.9
Lipid Metabolism	TC	rs3020619	17	61993137	G	A	-0.009	0.002	3.3e-09	2.7e-05	35.0
Lipid Metabolism	TC	rs8178824	17	64224775	T	C	0.062	0.004	3.6e-50	1.7e-04	221.8
Lipid Metabolism	TC	rs34310593	17	65252129	A	G	-0.011	0.002	1.4e-10	3.1e-05	41.1
Lipid Metabolism	TC	rs62084208	17	65827443	T	C	0.016	0.002	1.5e-22	7.2e-05	95.5
Lipid Metabolism	TC	rs139077622	17	67005758	T	C	-0.051	0.008	7.6e-10	2.9e-05	37.8
Lipid Metabolism	TC	rs72850341	17	67071579	A	G	-0.041	0.005	1.1e-16	5.2e-05	68.7
Lipid Metabolism	TC	rs77542162	17	67081278	G	A	0.143	0.005	1.1e-186	6.5e-04	849.2
Lipid Metabolism	TC	rs117406864	17	67107911	C	T	-0.052	0.008	4.7e-11	3.4e-05	43.3
Lipid Metabolism	TC	rs145958325	17	67165906	C	T	-0.052	0.007	2.5e-12	3.8e-05	49.0
Lipid Metabolism	TC	rs140245225	17	67218414	A	G	-0.043	0.006	1.9e-11	3.4e-05	45.0
Lipid Metabolism	TC	rs55812797	17	67292725	C	T	-0.033	0.003	2.0e-21	6.9e-05	90.4
Lipid Metabolism	TC	rs12452852	17	68161876	C	A	0.016	0.002	1.0e-14	4.5e-05	59.9
Lipid Metabolism	TC	rs11871352	17	68613765	T	G	0.011	0.002	4.7e-08	2.3e-05	29.9
Lipid Metabolism	TC	rs12603194	17	72754924	A	G	0.008	0.001	2.9e-08	2.4e-05	30.7
Lipid Metabolism	TC	rs1979215	17	73761966	T	C	0.013	0.001	7.6e-20	6.4e-05	83.2
Lipid Metabolism	TC	rs11657987	17	76387363	T	G	0.021	0.001	5.4e-53	1.8e-04	234.8
Lipid Metabolism	TC	rs7501528	17	76658346	G	C	-0.011	0.002	3.6e-10	3.1e-05	39.3
Lipid Metabolism	TC	rs11656152	17	80994674	T	C	0.013	0.002	4.8e-13	4.0e-05	52.3
Lipid Metabolism	TC	rs623325	18	2968275	T	C	-0.008	0.001	2.1e-09	2.7e-05	35.9
Lipid Metabolism	TC	rs62088911	18	9615667	G	T	0.016	0.002	3.5e-12	3.7e-05	48.4
Lipid Metabolism	TC	rs8097782	18	19673769	C	G	-0.008	0.001	3.8e-08	2.3e-05	30.3
Lipid Metabolism	TC	rs76537328	18	42072173	G	C	-0.013	0.002	3.3e-11	3.3e-05	44.0
Lipid Metabolism	TC	rs183119479	18	46342691	A	G	0.105	0.018	2.2e-09	4.2e-05	35.8
Lipid Metabolism	TC	rs188574990	18	46659191	G	A	0.093	0.010	6.2e-21	6.7e-05	88.1
Lipid Metabolism	TC	rs116844653	18	47060728	T	G	0.043	0.007	1.2e-10	3.2e-05	41.4
Lipid Metabolism	TC	rs149615216	18	47106028	T	C	0.182	0.006	1.0e-172	6.0e-04	785.0
Lipid Metabolism	TC	rs117316234	18	47117616	T	G	0.043	0.008	2.5e-08	2.4e-05	31.0
Lipid Metabolism	TC	rs186723634	18	47146860	T	C	-0.089	0.016	2.8e-08	3.2e-05	30.8
Lipid Metabolism	TC	rs7240405	18	47159090	G	A	0.046	0.002	1.7e-151	5.2e-04	687.3
Lipid Metabolism	TC	rs569057272	18	47165270	T	A	0.304	0.042	5.2e-13	1.3e-04	52.1
Lipid Metabolism	TC	rs11660468	18	47209143	T	C	0.022	0.001	1.3e-56	1.9e-04	251.3
Lipid Metabolism	TC	rs190932527	18	47557160	A	G	0.217	0.033	7.9e-11	1.0e-04	42.3
Lipid Metabolism	TC	rs150075950	18	47692905	C	A	0.155	0.026	5.1e-09	8.2e-05	34.2
Lipid Metabolism	TC	rs12968116	18	55322502	T	C	0.018	0.002	1.2e-18	5.9e-05	77.7
Lipid Metabolism	TC	rs2615198	18	61009966	G	A	-0.008	0.001	2.9e-08	2.4e-05	30.8
Lipid Metabolism	TC	rs185081762	18	65608586	T	C	0.490	0.087	1.5e-08	8.0e-05	32.0
Lipid Metabolism	TC	rs11081591	18	77880802	A	G	-0.012	0.002	1.4e-08	2.8e-05	32.2
Lipid Metabolism	TC	rs4807570	19	1123652	A	G	-0.010	0.002	1.8e-09	2.8e-05	36.2
Lipid Metabolism	TC	rs4807332	19	2793289	C	T	0.017	0.001	7.8e-31	1.0e-04	133.3
Lipid Metabolism	TC	rs7260465	19	4139440	T	C	-0.012	0.002	5.8e-14	4.3e-05	56.4
Lipid Metabolism	TC	rs892161	19	4493708	G	A	-0.012	0.001	3.9e-17	5.5e-05	70.8
Lipid Metabolism	TC	rs708686	19	5840619	T	C	0.010	0.001	9.7e-12	3.5e-05	46.4
Lipid Metabolism	TC	rs571497	19	7827830	A	G	-0.017	0.002	3.2e-19	6.1e-05	80.3
Lipid Metabolism	TC	rs1044250	19	8436164	T	C	0.010	0.001	2.6e-11	3.4e-05	44.4
Lipid Metabolism	TC	rs557704368	19	10234409	G	A	-0.100	0.011	6.4e-20	6.6e-05	83.5
Lipid Metabolism	TC	rs564800172	19	10261309	T	C	-0.181	0.021	7.8e-18	9.8e-05	74.0
Lipid Metabolism	TC	rs570748267	19	10461755	T	C	0.245	0.041	3.7e-09	6.3e-05	34.8
Lipid Metabolism	TC	rs118170044	19	10610968	T	C	-0.177	0.031	1.4e-08	4.3e-05	32.2
Lipid Metabolism	TC	rs144714727	19	10633354	C	G	-0.141	0.018	3.1e-15	6.0e-05	62.2

Phenotype	Trait	SNP	Chr	Position (GRCh37/hg19)	EA	NEA	BETA	SE	P-value	R2	F
Lipid Metabolism	TC	rs546689040	19	10639478	T	C	-0.182	0.019	8.8e-22	2.2e-04	92.0
Lipid Metabolism	TC	rs184823980	19	10655605	A	G	-0.092	0.013	1.3e-12	4.3e-05	50.3
Lipid Metabolism	TC	rs555159550	19	10675873	A	G	-0.212	0.038	2.8e-08	3.5e-05	30.8
Lipid Metabolism	TC	rs187996885	19	10682771	T	A	-0.063	0.010	3.6e-10	3.1e-05	39.3
Lipid Metabolism	TC	rs144295214	19	10693253	A	G	0.042	0.003	1.1e-35	1.2e-04	155.5
Lipid Metabolism	TC	rs191488621	19	10738826	T	C	-0.290	0.053	3.5e-08	4.7e-05	30.4
Lipid Metabolism	TC	rs572009601	19	10767520	T	C	0.066	0.009	1.5e-12	4.9e-05	50.1
Lipid Metabolism	TC	rs181305866	19	10773834	A	G	-0.114	0.019	1.2e-09	4.2e-05	37.0
Lipid Metabolism	TC	rs533519096	19	10862920	T	G	-0.110	0.014	1.7e-15	5.0e-05	63.4
Lipid Metabolism	TC	rs572821566	19	10897010	T	C	-0.121	0.019	2.5e-10	4.6e-05	40.0
Lipid Metabolism	TC	rs528872074	19	10914536	C	A	-0.209	0.026	6.1e-16	1.1e-04	65.4
Lipid Metabolism	TC	rs547704470	19	10918373	G	T	0.137	0.018	1.1e-14	7.3e-05	59.6
Lipid Metabolism	TC	rs532812516	19	10950497	T	C	-0.228	0.035	8.6e-11	6.1e-05	42.1
Lipid Metabolism	TC	rs185728685	19	10952199	C	T	0.444	0.060	8.2e-14	1.3e-04	55.8
Lipid Metabolism	TC	rs12977837	19	10953603	T	C	0.070	0.011	3.3e-10	3.8e-05	39.5
Lipid Metabolism	TC	rs200169512	19	11032164	A	G	-0.258	0.038	7.2e-12	6.6e-05	47.0
Lipid Metabolism	TC	rs182491671	19	11036679	T	C	0.295	0.040	1.2e-13	5.8e-05	55.0
Lipid Metabolism	TC	rs189389232	19	11049371	A	G	0.109	0.017	1.3e-10	4.6e-05	41.3
Lipid Metabolism	TC	rs548746023	19	11104727	C	T	-0.331	0.046	7.0e-13	1.3e-04	51.6
Lipid Metabolism	TC	rs536693949	19	11116576	G	A	-0.256	0.035	4.7e-13	7.1e-05	52.3
Lipid Metabolism	TC	rs563243095	19	11117282	A	G	0.142	0.021	4.1e-12	5.6e-05	48.1
Lipid Metabolism	TC	rs557024333	19	11125383	T	C	-0.236	0.042	2.3e-08	4.3e-05	31.2
Lipid Metabolism	TC	rs564402674	19	11132291	A	G	0.092	0.012	4.7e-14	4.6e-05	56.8
Lipid Metabolism	TC	rs112634605	19	11150013	C	G	-0.093	0.004	2.3e-134	4.6e-04	608.5
Lipid Metabolism	TC	rs189076912	19	11155283	A	C	0.131	0.018	6.4e-13	6.6e-05	51.7
Lipid Metabolism	TC	rs143580067	19	11161477	A	G	0.062	0.009	1.4e-12	3.9e-05	50.2
Lipid Metabolism	TC	rs3786722	19	11161537	A	C	-0.060	0.002	1.0e-200	1.1e-03	1495.9
Lipid Metabolism	TC	rs189085623	19	11165896	T	C	0.089	0.016	4.4e-08	2.4e-05	30.0
Lipid Metabolism	TC	rs10423733	19	11185919	C	T	-0.112	0.002	1.0e-200	2.9e-03	3889.1
Lipid Metabolism	TC	rs537582537	19	11217096	T	C	-0.240	0.025	1.1e-22	9.2e-05	96.1
Lipid Metabolism	TC	rs568336016	19	11219624	T	C	0.204	0.036	8.9e-09	8.4e-05	33.1
Lipid Metabolism	TC	rs55792959	19	11221289	T	C	-0.079	0.010	8.1e-15	4.6e-05	60.3
Lipid Metabolism	TC	rs527357541	19	11227855	A	C	0.192	0.034	1.2e-08	8.3e-05	32.5
Lipid Metabolism	TC	rs72658867	19	11231203	A	G	-0.269	0.006	1.0e-200	1.4e-03	1782.1
Lipid Metabolism	TC	rs527424716	19	11231402	A	G	-0.173	0.026	1.9e-11	4.3e-05	45.0
Lipid Metabolism	TC	rs563930198	19	11232812	T	C	0.057	0.007	7.1e-15	5.6e-05	60.6
Lipid Metabolism	TC	rs45508991	19	11233886	T	C	0.088	0.007	3.1e-36	1.2e-04	158.0
Lipid Metabolism	TC	rs181994650	19	11235610	T	C	0.064	0.011	2.2e-09	3.9e-05	35.8
Lipid Metabolism	TC	rs184231419	19	11245928	G	A	0.299	0.047	2.0e-10	6.0e-05	40.4
Lipid Metabolism	TC	rs531359724	19	11250445	A	G	-0.281	0.044	1.8e-10	4.9e-05	40.6
Lipid Metabolism	TC	rs143026258	19	11251500	C	T	-0.303	0.047	7.4e-11	1.1e-04	42.4
Lipid Metabolism	TC	rs184755907	19	11256675	T	C	-0.140	0.024	2.8e-09	3.8e-05	35.3
Lipid Metabolism	TC	rs142846953	19	11260682	A	G	0.107	0.013	1.4e-15	5.0e-05	63.8
Lipid Metabolism	TC	rs189143685	19	11274168	T	C	0.042	0.007	3.6e-10	3.0e-05	39.3
Lipid Metabolism	TC	rs148079581	19	11274668	C	A	0.083	0.008	7.2e-24	7.8e-05	101.5
Lipid Metabolism	TC	rs570974999	19	11280249	T	C	0.082	0.013	1.2e-09	3.0e-05	36.9
Lipid Metabolism	TC	rs562896829	19	11284299	G	A	-0.254	0.041	6.6e-10	4.1e-05	38.2
Lipid Metabolism	TC	rs12980863	19	11309871	T	C	0.021	0.001	3.9e-53	1.8e-04	235.4
Lipid Metabolism	TC	rs200393834	19	11311386	A	G	0.088	0.015	3.2e-09	2.9e-05	35.1
Lipid Metabolism	TC	rs139347491	19	11334329	T	A	-0.108	0.011	7.4e-25	8.2e-05	106.0
Lipid Metabolism	TC	rs190268411	19	11334682	C	T	-0.098	0.011	3.3e-18	5.8e-05	75.7
Lipid Metabolism	TC	rs117189398	19	11379317	T	C	-0.134	0.023	8.7e-09	3.5e-05	33.1
Lipid Metabolism	TC	rs563468321	19	11416795	G	T	-0.233	0.041	1.5e-08	7.8e-05	32.1
Lipid Metabolism	TC	rs183085199	19	11507217	A	G	-0.100	0.011	2.4e-21	1.0e-04	90.0
Lipid Metabolism	TC	rs534770939	19	11777205	C	T	-0.070	0.010	4.6e-12	4.3e-05	47.8
Lipid Metabolism	TC	rs543464461	19	12174123	T	C	-0.094	0.017	1.2e-08	3.5e-05	32.5

Phenotype	Trait	SNP	Chr	Position (GRCh37/hg19)	EA	NEA	BETA	SE	P-value	R2	F
Lipid Metabolism	TC	rs553952570	19	12749320	G	A	-0.447	0.057	4.2e-15	1.2e-04	61.6
Lipid Metabolism	TC	rs560788989	19	13250023	C	T	1.098	0.141	6.9e-15	8.9e-05	60.6
Lipid Metabolism	TC	rs527932938	19	13295968	T	C	-0.220	0.030	4.6e-13	7.6e-05	52.4
Lipid Metabolism	TC	rs574000814	19	13766888	A	G	-0.338	0.059	1.3e-08	6.8e-05	32.3
Lipid Metabolism	TC	rs10421262	19	14172951	G	T	-0.009	0.001	4.1e-09	2.8e-05	34.6
Lipid Metabolism	TC	rs633553	19	15796363	G	A	0.011	0.001	1.7e-15	4.8e-05	63.4
Lipid Metabolism	TC	rs8107753	19	17148165	T	C	0.012	0.002	9.6e-09	2.5e-05	32.9
Lipid Metabolism	TC	rs35365035	19	17252151	C	T	0.010	0.001	5.6e-12	3.8e-05	47.5
Lipid Metabolism	TC	rs59997441	19	17381463	C	T	0.009	0.001	4.3e-12	3.7e-05	48.0
Lipid Metabolism	TC	rs4808765	19	18334443	T	C	0.012	0.001	3.7e-16	5.0e-05	66.4
Lipid Metabolism	TC	rs10423802	19	18567901	T	C	0.014	0.002	1.6e-16	5.2e-05	68.1
Lipid Metabolism	TC	rs547734426	19	19254381	T	C	-0.175	0.019	4.9e-21	1.0e-04	88.6
Lipid Metabolism	TC	rs542068965	19	19354844	G	A	-0.237	0.029	4.0e-16	8.7e-05	66.3
Lipid Metabolism	TC	rs55989964	19	19361152	G	C	0.025	0.003	2.0e-13	4.1e-05	54.0
Lipid Metabolism	TC	rs72999033	19	19366632	T	C	-0.130	0.003	1.0e-200	1.7e-03	2226.3
Lipid Metabolism	TC	rs187429064	19	19380513	G	A	-0.187	0.007	1.3e-170	6.1e-04	775.2
Lipid Metabolism	TC	rs144821371	19	19381973	A	G	-0.128	0.012	6.0e-25	8.7e-05	106.4
Lipid Metabolism	TC	rs575932654	19	19425156	T	A	-0.304	0.047	7.7e-11	5.9e-05	42.3
Lipid Metabolism	TC	rs541344524	19	19427886	C	G	-0.176	0.023	2.1e-14	1.4e-04	58.4
Lipid Metabolism	TC	rs117970794	19	19468655	T	C	-0.155	0.010	1.0e-53	1.9e-04	238.1
Lipid Metabolism	TC	rs535451845	19	19504211	G	A	-0.259	0.041	3.5e-10	5.4e-05	39.4
Lipid Metabolism	TC	rs34498230	19	19544403	G	T	0.016	0.003	1.0e-09	2.9e-05	37.3
Lipid Metabolism	TC	rs34123065	19	19660577	A	G	0.019	0.003	2.3e-08	2.4e-05	31.2
Lipid Metabolism	TC	rs117316432	19	19689191	T	C	0.025	0.004	3.9e-11	3.4e-05	43.7
Lipid Metabolism	TC	rs185741822	19	19705018	T	C	-0.176	0.030	2.9e-09	5.7e-05	35.2
Lipid Metabolism	TC	rs566182996	19	19726518	T	C	-0.179	0.027	6.5e-11	6.1e-05	42.7
Lipid Metabolism	TC	rs79892166	19	19804335	T	G	0.018	0.003	5.9e-09	2.6e-05	33.9
Lipid Metabolism	TC	rs528779347	19	19868095	C	G	-0.168	0.026	5.5e-11	6.8e-05	43.0
Lipid Metabolism	TC	rs192564555	19	20402896	C	T	-0.184	0.033	1.9e-08	2.0e-04	31.6
Lipid Metabolism	TC	rs139549297	19	20435035	G	A	-0.117	0.021	2.2e-08	3.8e-05	31.3
Lipid Metabolism	TC	rs144984216	19	20479901	T	C	-0.059	0.005	1.3e-30	1.4e-04	132.3
Lipid Metabolism	TC	rs184681882	19	21326928	G	T	-0.171	0.029	3.9e-09	5.3e-05	34.7
Lipid Metabolism	TC	rs141904453	19	21635117	C	T	-0.032	0.005	1.1e-09	2.8e-05	37.2
Lipid Metabolism	TC	rs12461296	19	33774591	A	C	0.009	0.002	3.4e-08	2.3e-05	30.5
Lipid Metabolism	TC	rs117047468	19	33825670	G	T	-0.023	0.003	5.1e-15	4.6e-05	61.2
Lipid Metabolism	TC	rs1688042	19	35553588	T	A	0.017	0.003	1.2e-10	3.2e-05	41.5
Lipid Metabolism	TC	rs739495	19	35662809	C	A	-0.009	0.002	3.0e-09	2.9e-05	35.2
Lipid Metabolism	TC	rs56268127	19	38294448	T	C	-0.010	0.001	1.5e-13	4.2e-05	54.5
Lipid Metabolism	TC	rs56113850	19	41353107	C	T	0.010	0.001	2.2e-11	3.7e-05	44.8
Lipid Metabolism	TC	rs35538872	19	41754430	A	G	-0.073	0.009	5.4e-16	5.0e-05	65.7
Lipid Metabolism	TC	rs547268356	19	43757939	C	T	-0.457	0.076	1.6e-09	3.9e-04	36.4
Lipid Metabolism	TC	rs571370258	19	44042739	G	A	-0.194	0.032	8.5e-10	3.5e-05	37.6
Lipid Metabolism	TC	rs145149776	19	44179442	G	C	-0.052	0.009	3.6e-08	2.4e-05	30.4
Lipid Metabolism	TC	rs13344768	19	44189441	A	G	-0.017	0.003	1.8e-09	2.7e-05	36.2
Lipid Metabolism	TC	rs142669812	19	44440096	G	A	-0.197	0.030	5.4e-11	4.7e-05	43.0
Lipid Metabolism	TC	rs189047355	19	44480751	T	C	-0.108	0.014	6.0e-15	4.8e-05	60.9
Lipid Metabolism	TC	rs139777621	19	44832662	T	C	-0.608	0.101	1.7e-09	5.7e-05	36.3
Lipid Metabolism	TC	rs138181035	19	44863163	T	C	-0.128	0.011	2.6e-29	9.7e-05	126.4
Lipid Metabolism	TC	rs143777867	19	44978625	A	G	-0.098	0.006	4.4e-53	1.8e-04	235.2
Lipid Metabolism	TC	rs78458671	19	45022612	T	A	-0.034	0.003	6.0e-30	1.0e-04	129.2
Lipid Metabolism	TC	rs55791016	19	45124455	T	C	-0.119	0.011	1.3e-25	1.2e-04	109.5
Lipid Metabolism	TC	rs550835657	19	45148926	T	C	-0.255	0.031	1.4e-16	8.9e-05	68.2
Lipid Metabolism	TC	rs554166514	19	45194343	T	C	-0.122	0.017	5.0e-13	4.3e-05	52.2
Lipid Metabolism	TC	rs550621052	19	45207835	A	T	-0.237	0.024	4.1e-23	9.1e-05	98.0
Lipid Metabolism	TC	rs1551891	19	45231821	A	G	-0.112	0.002	1.0e-200	1.6e-03	2090.2
Lipid Metabolism	TC	rs573007449	19	45263188	T	C	-0.317	0.020	1.1e-57	2.9e-04	256.4

Phenotype	Trait	SNP	Chr	Position (GRCh37/hg19)	EA	NEA	BETA	SE	P-value	R2	F
Lipid Metabolism	TC	rs548150833	19	45274462	A	G	0.176	0.023	2.6e-14	6.5e-05	58.0
Lipid Metabolism	TC	rs112450640	19	45296364	A	G	-0.272	0.005	1.0e-200	2.3e-03	3079.7
Lipid Metabolism	TC	rs547570291	19	45308756	A	G	-0.398	0.021	9.7e-84	4.3e-04	375.9
Lipid Metabolism	TC	rs528070791	19	45316221	G	A	0.135	0.007	4.3e-80	3.3e-04	359.1
Lipid Metabolism	TC	rs372019933	19	45316423	A	C	0.126	0.018	1.2e-12	6.0e-05	50.5
Lipid Metabolism	TC	rs180887453	19	45318495	G	A	0.161	0.013	3.7e-34	1.4e-04	148.5
Lipid Metabolism	TC	rs140105611	19	45318745	A	G	0.184	0.021	8.0e-18	8.5e-05	74.0
Lipid Metabolism	TC	rs117012738	19	45322671	T	C	0.161	0.017	1.3e-20	9.8e-05	86.6
Lipid Metabolism	TC	rs28399637	19	45324138	A	G	0.066	0.002	1.0e-200	1.5e-03	1769.4
Lipid Metabolism	TC	rs529877444	19	45328216	G	A	-0.565	0.058	1.1e-22	2.4e-04	96.1
Lipid Metabolism	TC	rs569365275	19	45331133	A	G	0.215	0.018	4.9e-34	1.6e-04	147.9
Lipid Metabolism	TC	rs577543168	19	45338928	T	C	0.227	0.036	4.9e-10	9.7e-05	38.7
Lipid Metabolism	TC	rs147695675	19	45339947	A	G	0.109	0.018	3.1e-09	8.6e-05	35.2
Lipid Metabolism	TC	rs189409600	19	45341066	T	C	0.151	0.019	6.8e-15	6.5e-05	60.6
Lipid Metabolism	TC	rs528951928	19	45351519	T	C	-0.460	0.038	9.3e-34	2.4e-04	146.7
Lipid Metabolism	TC	rs554115297	19	45364216	G	A	0.274	0.035	4.8e-15	4.0e-04	61.3
Lipid Metabolism	TC	rs547934469	19	45364230	T	C	0.336	0.046	2.4e-13	9.3e-05	53.6
Lipid Metabolism	TC	rs112484236	19	45376893	C	A	0.160	0.012	2.0e-42	2.0e-04	186.3
Lipid Metabolism	TC	rs539910115	19	45388402	T	C	0.292	0.025	3.0e-32	1.6e-04	139.7
Lipid Metabolism	TC	rs527428691	19	45389633	T	C	0.205	0.014	1.3e-51	2.5e-04	228.5
Lipid Metabolism	TC	rs148665049	19	45391132	T	C	0.266	0.045	4.3e-09	4.3e-05	34.5
Lipid Metabolism	TC	rs77301115	19	45396973	A	G	0.202	0.004	1.0e-200	1.7e-03	2234.1
Lipid Metabolism	TC	rs549854089	19	45407113	A	G	0.152	0.020	4.5e-14	5.4e-05	56.9
Lipid Metabolism	TC	rs182067414	19	45425954	C	T	-0.357	0.013	3.2e-170	6.0e-04	773.5
Lipid Metabolism	TC	rs5112	19	45430280	G	C	0.051	0.002	1.0e-200	8.6e-04	1000.0
Lipid Metabolism	TC	rs541817716	19	45433531	A	T	0.262	0.027	6.5e-23	9.8e-05	97.1
Lipid Metabolism	TC	rs116949436	19	45463386	A	G	0.142	0.007	4.1e-89	3.1e-04	400.6
Lipid Metabolism	TC	rs137928795	19	45495742	T	C	0.081	0.012	3.8e-11	3.8e-05	43.7
Lipid Metabolism	TC	rs184856492	19	45507148	G	T	0.204	0.037	4.5e-08	4.5e-05	29.9
Lipid Metabolism	TC	rs188161988	19	45523453	G	A	0.111	0.019	8.0e-09	3.8e-05	33.3
Lipid Metabolism	TC	rs553887842	19	45529797	A	G	0.122	0.017	9.0e-13	5.9e-05	51.0
Lipid Metabolism	TC	rs117701768	19	45559557	G	A	0.058	0.010	5.4e-09	2.6e-05	34.0
Lipid Metabolism	TC	rs181826797	19	45619516	A	G	0.037	0.007	4.8e-08	2.3e-05	29.8
Lipid Metabolism	TC	rs537785075	19	45641786	A	G	-0.264	0.034	4.9e-15	9.0e-05	61.3
Lipid Metabolism	TC	rs550905135	19	45660421	T	C	-0.344	0.050	9.3e-12	1.1e-04	46.5
Lipid Metabolism	TC	rs200262696	19	45667538	A	G	0.199	0.033	1.7e-09	5.0e-05	36.3
Lipid Metabolism	TC	rs182286598	19	45682698	A	C	0.036	0.006	8.1e-11	3.2e-05	42.2
Lipid Metabolism	TC	rs62118471	19	45691049	C	T	-0.097	0.005	3.2e-103	3.6e-04	465.4
Lipid Metabolism	TC	rs528302268	19	45744160	C	T	-0.141	0.022	2.6e-10	4.9e-05	40.0
Lipid Metabolism	TC	rs151174524	19	45793916	C	G	0.092	0.013	3.8e-13	7.2e-05	52.7
Lipid Metabolism	TC	rs191846322	19	45821856	T	C	-0.336	0.054	6.8e-10	8.5e-05	38.1
Lipid Metabolism	TC	rs560577742	19	45893023	G	T	-0.963	0.143	1.7e-11	7.4e-05	45.3
Lipid Metabolism	TC	rs182598991	19	45953594	G	C	0.072	0.009	7.8e-16	5.0e-05	64.9
Lipid Metabolism	TC	rs11666505	19	46004312	G	A	0.061	0.009	4.2e-11	3.4e-05	43.5
Lipid Metabolism	TC	rs141651018	19	46042665	T	C	-0.066	0.004	9.0e-62	2.1e-04	275.1
Lipid Metabolism	TC	rs4802269	19	46167469	G	A	0.010	0.001	7.0e-11	3.3e-05	42.5
Lipid Metabolism	TC	rs578060652	19	46366299	T	C	-0.284	0.050	1.4e-08	7.8e-05	32.2
Lipid Metabolism	TC	rs34934360	19	46398989	G	A	-0.031	0.002	1.5e-47	1.6e-04	209.8
Lipid Metabolism	TC	rs180885509	19	46612398	G	C	-0.087	0.015	4.1e-09	2.8e-05	34.6
Lipid Metabolism	TC	rs1644350	19	47214996	A	T	0.016	0.002	1.8e-15	4.8e-05	63.3
Lipid Metabolism	TC	rs679574	19	49206108	G	C	0.029	0.001	9.0e-102	3.5e-04	458.7
Lipid Metabolism	TC	rs838148	19	49246378	A	G	-0.017	0.002	3.4e-11	3.3e-05	43.9
Lipid Metabolism	TC	rs2009984	19	50001877	A	T	-0.050	0.008	4.6e-10	3.0e-05	38.8
Lipid Metabolism	TC	rs141409212	19	50006402	T	C	-0.060	0.008	3.1e-15	4.8e-05	62.2
Lipid Metabolism	TC	rs111981233	19	50016479	G	T	0.027	0.003	1.2e-25	8.3e-05	109.6
Lipid Metabolism	TC	rs138578094	19	50055899	A	G	0.050	0.007	2.4e-11	3.4e-05	44.6

Phenotype	Trait	SNP	Chr	Position (GRCh37/hg19)	EA	NEA	BETA	SE	P-value	R2	F
Lipid Metabolism	TC	rs103294	19	54797848	T	C	0.018	0.002	1.5e-28	9.3e-05	122.9
Lipid Metabolism	TC	rs12610709	19	56102362	A	G	0.010	0.002	3.7e-08	2.3e-05	30.3
Lipid Metabolism	TC	rs2189699	19	57031408	A	G	-0.011	0.001	4.4e-14	4.3e-05	57.0
Lipid Metabolism	TC	rs145467679	19	58308789	C	T	0.061	0.008	1.3e-15	4.9e-05	63.9
Lipid Metabolism	TC	rs12983990	19	58341843	G	A	-0.015	0.002	3.2e-17	5.4e-05	71.2
Lipid Metabolism	TC	rs35081008	19	58662235	T	C	-0.028	0.002	2.7e-52	1.8e-04	231.6
Lipid Metabolism	TC	rs56390031	19	58998327	G	A	-0.009	0.002	2.1e-09	2.7e-05	35.9
Lipid Metabolism	TC	rs6051908	20	403653	G	T	0.010	0.001	2.1e-11	3.4e-05	44.9
Lipid Metabolism	TC	rs615355	20	3682761	C	T	0.009	0.001	1.5e-10	3.1e-05	41.0
Lipid Metabolism	TC	rs62204030	20	5532306	G	T	-0.017	0.003	3.6e-08	2.3e-05	30.4
Lipid Metabolism	TC	rs73075609	20	5580789	T	C	0.045	0.005	2.7e-23	7.5e-05	98.9
Lipid Metabolism	TC	rs440090	20	5785194	T	C	0.010	0.002	6.1e-11	3.4e-05	42.8
Lipid Metabolism	TC	rs438568	20	12958687	G	A	0.013	0.001	1.9e-20	6.5e-05	85.9
Lipid Metabolism	TC	rs6105819	20	17811820	A	C	0.022	0.004	1.0e-09	2.8e-05	37.3
Lipid Metabolism	TC	rs2618568	20	17843968	A	C	-0.030	0.001	3.0e-96	3.4e-04	433.3
Lipid Metabolism	TC	rs3746337	20	25208272	T	C	0.010	0.001	9.2e-13	3.9e-05	51.0
Lipid Metabolism	TC	rs6058897	20	31398105	C	A	0.010	0.001	1.2e-12	3.9e-05	50.5
Lipid Metabolism	TC	rs7261820	20	34160840	A	G	-0.035	0.002	3.4e-72	2.4e-04	322.9
Lipid Metabolism	TC	rs2590947	20	34755927	T	C	-0.029	0.003	2.9e-17	5.4e-05	71.4
Lipid Metabolism	TC	rs146472249	20	38624741	G	A	0.054	0.009	8.8e-09	2.5e-05	33.1
Lipid Metabolism	TC	rs56202412	20	39119602	A	T	-0.030	0.003	1.7e-29	9.6e-05	127.2
Lipid Metabolism	TC	rs1883711	20	39179822	C	G	0.125	0.004	1.0e-200	7.4e-04	962.9
Lipid Metabolism	TC	rs2865509	20	39239797	C	T	-0.018	0.001	5.1e-38	1.3e-04	166.2
Lipid Metabolism	TC	rs6072195	20	39560474	T	C	0.010	0.002	3.7e-09	2.6e-05	34.7
Lipid Metabolism	TC	rs6029549	20	39754695	G	C	0.031	0.001	9.7e-118	4.0e-04	532.1
Lipid Metabolism	TC	rs56275254	20	40253910	G	A	0.023	0.003	5.7e-14	4.3e-05	56.5
Lipid Metabolism	TC	rs12480046	20	40305932	T	C	0.011	0.002	1.2e-12	3.8e-05	50.5
Lipid Metabolism	TC	rs2425633	20	43021214	A	G	0.009	0.001	7.0e-10	2.9e-05	38.0
Lipid Metabolism	TC	rs1800961	20	43042364	T	C	-0.087	0.004	8.0e-121	4.2e-04	546.3
Lipid Metabolism	TC	rs1569723	20	44742064	A	C	0.011	0.002	1.5e-13	4.1e-05	54.5
Lipid Metabolism	TC	rs2295027	20	47582884	A	G	0.012	0.001	1.3e-15	4.9e-05	64.0
Lipid Metabolism	TC	rs6063965	20	52191200	A	G	0.014	0.002	2.4e-11	3.4e-05	44.6
Lipid Metabolism	TC	rs6022849	20	52532637	C	G	0.013	0.001	8.4e-20	6.5e-05	82.9
Lipid Metabolism	TC	rs3746778	20	61341472	A	G	-0.009	0.002	6.2e-09	2.9e-05	33.8
Lipid Metabolism	TC	rs4337532	20	62715089	A	G	-0.011	0.001	1.7e-13	4.8e-05	54.4
Lipid Metabolism	TC	rs11906201	20	62903535	C	T	0.016	0.002	7.7e-20	7.2e-05	83.1
Lipid Metabolism	TC	rs1475884	21	17591962	G	A	0.009	0.001	2.4e-10	3.1e-05	40.1
Lipid Metabolism	TC	rs112646542	21	33104440	C	T	0.029	0.003	1.0e-20	6.6e-05	87.2
Lipid Metabolism	TC	rs2094871	21	40460859	A	G	0.008	0.001	1.9e-08	2.4e-05	31.5
Lipid Metabolism	TC	rs1963676	21	40709960	T	C	0.014	0.001	7.6e-25	8.1e-05	105.9
Lipid Metabolism	TC	rs9977637	21	46914642	G	A	-0.012	0.002	1.9e-13	4.1e-05	54.1
Lipid Metabolism	TC	rs35665085	22	17625915	A	G	0.019	0.003	2.2e-10	3.1e-05	40.3
Lipid Metabolism	TC	rs11703382	22	18481857	T	C	0.012	0.002	1.1e-13	4.2e-05	55.2
Lipid Metabolism	TC	rs11089620	22	21922456	G	C	-0.025	0.002	1.6e-51	1.7e-04	228.1
Lipid Metabolism	TC	rs975704	22	30572736	A	G	0.034	0.005	7.7e-14	4.3e-05	55.9
Lipid Metabolism	TC	rs9606738	22	30891250	C	T	-0.013	0.002	9.4e-16	4.9e-05	64.5
Lipid Metabolism	TC	rs117147052	22	32411817	C	T	0.020	0.003	2.6e-10	3.0e-05	40.0
Lipid Metabolism	TC	rs138767	22	35703366	C	T	-0.014	0.001	1.5e-22	7.3e-05	95.5
Lipid Metabolism	TC	rs138347	22	41248031	G	A	-0.013	0.001	2.7e-19	6.2e-05	80.6
Lipid Metabolism	TC	rs4823173	22	44328730	A	G	-0.025	0.002	8.7e-43	1.4e-04	188.0
Lipid Metabolism	TC	rs12165526	22	44361713	A	T	-0.017	0.002	8.3e-14	4.2e-05	55.7
Lipid Metabolism	TC	rs5764726	22	45826033	G	A	-0.009	0.001	4.4e-09	2.6e-05	34.5
Lipid Metabolism	TC	rs13268	22	45996298	G	A	-0.031	0.004	6.7e-13	3.9e-05	51.6
Lipid Metabolism	TC	rs4253766	22	46623905	T	C	0.019	0.002	5.6e-19	6.0e-05	79.2
Lipid Metabolism	TC	rs1042311	22	46627780	T	C	0.077	0.010	1.7e-15	5.1e-05	63.4
Lipid Metabolism	TC	rs7410608	22	50858813	C	G	0.012	0.001	8.7e-17	5.2e-05	69.2

Phenotype	Trait	SNP	Chr	Position (GRCh37/hg19)	EA	NEA	BETA	SE	P-value	R2	F
Lipid Metabolism	TG	rs12060249	1	2151579	A	C	0.012	0.002	6.0e-13	4.2e-05	51.8
Lipid Metabolism	TG	rs34464114	1	11224916	G	T	0.067	0.012	3.9e-08	4.6e-05	30.2
Lipid Metabolism	TG	rs12563724	1	15259476	T	C	-0.013	0.002	3.6e-08	2.4e-05	30.4
Lipid Metabolism	TG	rs4661718	1	16505908	T	C	0.011	0.001	8.2e-15	4.8e-05	60.3
Lipid Metabolism	TG	rs1986133	1	23804917	T	C	0.012	0.001	2.7e-15	5.0e-05	62.5
Lipid Metabolism	TG	rs572818211	1	24244867	C	G	0.196	0.035	3.1e-08	7.9e-05	30.6
Lipid Metabolism	TG	rs3120159	1	26238289	G	A	0.010	0.001	1.6e-11	3.7e-05	45.4
Lipid Metabolism	TG	rs2219320	1	26803430	C	T	0.011	0.002	2.3e-11	3.6e-05	44.7
Lipid Metabolism	TG	rs114165349	1	27021913	C	G	0.068	0.005	7.9e-51	1.8e-04	224.9
Lipid Metabolism	TG	rs3766823	1	32197257	A	G	0.010	0.002	3.5e-08	2.4e-05	30.4
Lipid Metabolism	TG	rs184666487	1	32983895	T	C	0.108	0.019	7.1e-09	3.0e-05	33.5
Lipid Metabolism	TG	rs4660690	1	39819503	A	G	0.021	0.002	4.4e-35	1.2e-04	152.7
Lipid Metabolism	TG	rs7529794	1	40403370	T	G	0.013	0.002	2.5e-16	5.4e-05	67.2
Lipid Metabolism	TG	rs12078980	1	51508526	C	T	-0.022	0.002	9.4e-21	7.0e-05	87.3
Lipid Metabolism	TG	rs213484	1	54858872	C	G	-0.012	0.001	2.8e-17	5.7e-05	71.5
Lipid Metabolism	TG	rs138823858	1	61256030	T	C	-0.101	0.017	1.2e-09	3.2e-05	37.0
Lipid Metabolism	TG	rs9970140	1	61684288	G	A	-0.021	0.003	1.6e-16	5.4e-05	68.1
Lipid Metabolism	TG	rs12138529	1	62639035	G	T	-0.042	0.007	1.7e-10	3.8e-05	40.8
Lipid Metabolism	TG	rs10889325	1	62856695	G	A	-0.021	0.002	4.5e-26	9.0e-05	111.5
Lipid Metabolism	TG	rs624698	1	62900811	G	A	-0.064	0.002	1.0e-200	1.5e-03	1584.5
Lipid Metabolism	TG	rs183115103	1	62974243	A	T	-0.084	0.015	1.7e-08	2.7e-05	31.8
Lipid Metabolism	TG	rs554472916	1	63011663	A	G	-0.057	0.008	1.7e-13	4.4e-05	54.4
Lipid Metabolism	TG	rs182008693	1	63027257	G	A	-0.095	0.015	5.7e-10	3.2e-05	38.4
Lipid Metabolism	TG	rs181465052	1	63044818	A	G	-0.064	0.011	2.2e-09	2.9e-05	35.8
Lipid Metabolism	TG	rs184187742	1	63131310	A	G	-0.076	0.012	7.2e-11	3.5e-05	42.5
Lipid Metabolism	TG	rs186242996	1	63132165	T	G	-0.055	0.008	5.2e-12	5.1e-05	47.6
Lipid Metabolism	TG	rs559647195	1	63352650	T	C	-0.232	0.032	7.2e-13	1.3e-04	51.5
Lipid Metabolism	TG	rs186955977	1	63381339	G	A	-0.184	0.025	1.2e-13	6.0e-05	54.9
Lipid Metabolism	TG	rs143999074	1	63383255	A	G	-0.028	0.005	4.3e-09	2.8e-05	34.5
Lipid Metabolism	TG	rs549777175	1	63413521	A	G	-0.367	0.029	1.9e-36	2.1e-04	158.9
Lipid Metabolism	TG	rs583691	1	71397835	G	A	-0.009	0.001	3.2e-11	3.6e-05	44.0
Lipid Metabolism	TG	rs12740789	1	72752073	A	G	-0.014	0.002	1.9e-14	4.7e-05	58.7
Lipid Metabolism	TG	rs165316	1	91533297	G	A	-0.013	0.002	8.0e-15	4.8e-05	60.3
Lipid Metabolism	TG	rs12133576	1	93816400	G	A	0.011	0.001	5.2e-14	4.5e-05	56.6
Lipid Metabolism	TG	rs11165937	1	98433535	A	T	0.015	0.002	9.2e-16	5.2e-05	64.6
Lipid Metabolism	TG	rs7545402	1	101321076	A	G	-0.010	0.002	3.0e-08	2.5e-05	30.7
Lipid Metabolism	TG	rs7528419	1	109817192	G	A	-0.011	0.002	4.8e-12	3.8e-05	47.7
Lipid Metabolism	TG	rs579035	1	110346885	A	C	-0.009	0.001	2.6e-09	3.0e-05	35.5
Lipid Metabolism	TG	rs61785483	1	110504663	A	G	-0.008	0.001	4.4e-08	2.4e-05	30.0
Lipid Metabolism	TG	rs197439	1	112280990	G	A	0.008	0.001	3.9e-09	2.8e-05	34.7
Lipid Metabolism	TG	rs10494363	1	149909495	A	G	-0.027	0.003	4.9e-25	8.5e-05	106.8
Lipid Metabolism	TG	rs141281255	1	149927630	T	C	0.056	0.010	2.2e-08	3.4e-05	31.3
Lipid Metabolism	TG	rs11580946	1	150551327	A	G	-0.037	0.006	7.9e-11	3.4e-05	42.3
Lipid Metabolism	TG	rs72704620	1	150738596	A	G	0.017	0.003	5.1e-09	2.7e-05	34.2
Lipid Metabolism	TG	rs12750321	1	154251626	G	A	-0.015	0.002	1.9e-22	7.6e-05	95.0
Lipid Metabolism	TG	rs12743272	1	155033334	A	G	0.014	0.002	2.2e-11	3.6e-05	44.8
Lipid Metabolism	TG	rs2502810	1	161223292	T	C	-0.009	0.002	2.7e-08	2.5e-05	30.9
Lipid Metabolism	TG	rs9425591	1	172358697	T	G	-0.013	0.001	3.2e-21	7.2e-05	89.4
Lipid Metabolism	TG	rs77772149	1	174046339	G	A	-0.017	0.003	8.8e-09	2.6e-05	33.1
Lipid Metabolism	TG	rs115276619	1	184865132	A	T	-0.050	0.006	1.5e-18	6.2e-05	77.2
Lipid Metabolism	TG	rs12069549	1	200036139	T	C	0.013	0.002	1.5e-09	2.9e-05	36.6
Lipid Metabolism	TG	rs12129763	1	202184031	C	T	-0.009	0.001	1.4e-10	3.3e-05	41.2
Lipid Metabolism	TG	rs6677511	1	203510228	G	T	-0.012	0.001	2.3e-15	5.3e-05	62.8
Lipid Metabolism	TG	rs1572993	1	205045087	A	G	0.013	0.001	4.2e-20	6.7e-05	84.3
Lipid Metabolism	TG	rs17014323	1	209513852	A	G	0.012	0.002	9.9e-10	3.0e-05	37.3
Lipid Metabolism	TG	rs910109	1	210423049	T	A	-0.009	0.002	7.1e-10	3.0e-05	38.0

Phenotype	Trait	SNP	Chr	Position (GRCh37/hg19)	EA	NEA	BETA	SE	P-value	R2	F
Lipid Metabolism	TG	rs72753599	1	214180519	T	C	-0.010	0.002	4.2e-09	2.8e-05	34.5
Lipid Metabolism	TG	rs2791560	1	219589065	T	G	0.013	0.002	1.7e-13	4.3e-05	54.4
Lipid Metabolism	TG	rs1415293	1	219730006	T	A	-0.020	0.001	3.4e-40	1.4e-04	176.1
Lipid Metabolism	TG	rs2807845	1	220996287	T	G	-0.013	0.001	9.3e-22	7.3e-05	91.9
Lipid Metabolism	TG	rs2247213	1	221055463	A	G	0.009	0.001	9.7e-10	3.0e-05	37.4
Lipid Metabolism	TG	rs2291834	1	222832295	C	T	0.009	0.002	1.5e-08	2.6e-05	32.0
Lipid Metabolism	TG	rs4653443	1	226506053	T	C	-0.011	0.002	7.0e-09	4.1e-05	33.5
Lipid Metabolism	TG	rs12145479	1	226659924	T	C	0.019	0.003	4.0e-10	3.1e-05	39.1
Lipid Metabolism	TG	rs12062317	1	228062661	A	G	-0.009	0.001	1.7e-09	2.9e-05	36.3
Lipid Metabolism	TG	rs67093013	1	230242395	G	A	0.015	0.002	4.8e-17	5.6e-05	70.4
Lipid Metabolism	TG	rs116537941	1	230259882	G	A	0.032	0.005	3.3e-10	3.2e-05	39.5
Lipid Metabolism	TG	rs2281721	1	230297136	T	C	-0.044	0.001	1.0e-200	8.1e-04	1011.5
Lipid Metabolism	TG	rs78517535	1	230326892	C	T	0.063	0.008	3.0e-16	5.4e-05	66.8
Lipid Metabolism	TG	rs146783593	1	230360471	T	C	0.048	0.008	1.2e-08	2.6e-05	32.5
Lipid Metabolism	TG	rs16851339	1	230416832	A	T	-0.020	0.002	9.3e-32	1.1e-04	137.5
Lipid Metabolism	TG	rs271772	1	234679850	G	A	0.008	0.001	1.2e-08	2.6e-05	32.4
Lipid Metabolism	TG	rs556107	1	234853059	T	C	0.008	0.001	1.4e-08	2.6e-05	32.2
Lipid Metabolism	TG	rs4660149	1	236199787	A	T	0.009	0.001	4.2e-09	2.8e-05	34.5
Lipid Metabolism	TG	rs300744	2	165003	T	A	0.010	0.002	2.6e-08	2.5e-05	31.0
Lipid Metabolism	TG	rs17713879	2	254215	A	G	-0.011	0.001	4.8e-15	4.9e-05	61.3
Lipid Metabolism	TG	rs114703824	2	3435446	G	A	0.034	0.006	3.1e-08	2.5e-05	30.7
Lipid Metabolism	TG	rs3820897	2	3642361	C	T	0.016	0.002	2.2e-20	6.9e-05	85.6
Lipid Metabolism	TG	rs11096641	2	20374158	A	G	-0.017	0.001	1.1e-35	1.2e-04	155.6
Lipid Metabolism	TG	rs185930747	2	20997604	T	A	-0.075	0.009	7.5e-16	5.3e-05	65.0
Lipid Metabolism	TG	rs6734506	2	21134656	T	C	-0.016	0.001	3.2e-29	1.0e-04	125.9
Lipid Metabolism	TG	rs540847230	2	21197077	A	G	-0.073	0.013	2.4e-08	2.8e-05	31.1
Lipid Metabolism	TG	rs10184054	2	21203877	G	C	-0.064	0.002	1.0e-200	1.3e-03	1576.9
Lipid Metabolism	TG	rs563525668	2	21258045	T	C	-0.096	0.015	3.7e-11	5.1e-05	43.8
Lipid Metabolism	TG	rs571518593	2	21293728	G	A	-0.122	0.022	4.5e-08	3.9e-05	29.9
Lipid Metabolism	TG	rs187350477	2	21350277	C	T	-0.085	0.013	1.8e-10	3.4e-05	40.7
Lipid Metabolism	TG	rs12997825	2	21406831	T	C	0.026	0.002	1.1e-31	1.1e-04	137.3
Lipid Metabolism	TG	rs543438430	2	21504380	A	G	-0.089	0.013	2.2e-12	4.0e-05	49.3
Lipid Metabolism	TG	rs80341249	2	21658852	C	T	-0.062	0.009	1.4e-11	3.7e-05	45.7
Lipid Metabolism	TG	rs184344489	2	21944355	A	G	-0.108	0.018	1.8e-09	4.7e-05	36.2
Lipid Metabolism	TG	rs11895890	2	25557045	T	C	0.008	0.001	1.2e-08	2.6e-05	32.4
Lipid Metabolism	TG	rs6721446	2	25943974	G	A	-0.009	0.001	2.8e-11	3.6e-05	44.3
Lipid Metabolism	TG	rs62130490	2	26492367	T	C	0.027	0.003	3.2e-16	7.7e-05	66.7
Lipid Metabolism	TG	rs116408176	2	26921027	T	C	-0.035	0.005	2.3e-12	3.9e-05	49.2
Lipid Metabolism	TG	rs113595686	2	26928992	G	A	-0.043	0.005	8.4e-16	5.2e-05	64.8
Lipid Metabolism	TG	rs71441048	2	27059947	T	C	0.077	0.012	7.6e-11	3.5e-05	42.4
Lipid Metabolism	TG	rs150190141	2	27104418	A	G	0.058	0.008	1.4e-12	4.1e-05	50.2
Lipid Metabolism	TG	rs546704946	2	27115540	C	A	0.089	0.013	8.5e-12	5.6e-05	46.7
Lipid Metabolism	TG	rs116231110	2	27121650	A	G	0.063	0.011	1.1e-08	2.9e-05	32.7
Lipid Metabolism	TG	rs541195380	2	27122508	T	C	-0.114	0.019	3.0e-09	3.8e-05	35.2
Lipid Metabolism	TG	rs540806374	2	27191706	A	G	-0.071	0.011	9.9e-11	3.5e-05	41.8
Lipid Metabolism	TG	rs540367586	2	27229081	C	A	-0.075	0.011	7.1e-11	4.0e-05	42.5
Lipid Metabolism	TG	rs139623340	2	27287740	C	G	-0.042	0.005	2.2e-14	4.7e-05	58.3
Lipid Metabolism	TG	rs72808965	2	27302658	T	C	0.090	0.012	4.6e-13	6.0e-05	52.4
Lipid Metabolism	TG	rs561757894	2	27320225	G	C	-0.100	0.018	2.3e-08	2.7e-05	31.2
Lipid Metabolism	TG	rs193028604	2	27337534	T	G	0.141	0.023	7.4e-10	3.7e-05	37.9
Lipid Metabolism	TG	rs41288801	2	27355972	C	T	0.061	0.010	1.0e-09	3.4e-05	37.3
Lipid Metabolism	TG	rs934987	2	27372522	T	G	0.092	0.011	4.6e-16	5.3e-05	65.9
Lipid Metabolism	TG	rs6728736	2	27385318	T	C	-0.084	0.015	3.8e-08	2.5e-05	30.2
Lipid Metabolism	TG	rs566781109	2	27414950	T	G	0.076	0.013	1.3e-08	3.0e-05	32.3
Lipid Metabolism	TG	rs140069117	2	27493631	A	G	-0.068	0.011	2.8e-10	3.2e-05	39.8
Lipid Metabolism	TG	rs192516059	2	27520175	A	G	0.120	0.015	6.0e-15	5.2e-05	60.9

Phenotype	Trait	SNP	Chr	Position (GRCh37/hg19)	EA	NEA	BETA	SE	P-value	R2	F
Lipid Metabolism	TG	rs185010846	2	27525434	T	C	0.063	0.010	4.6e-11	3.9e-05	43.3
Lipid Metabolism	TG	rs138393441	2	27537060	A	G	-0.113	0.020	1.2e-08	3.1e-05	32.5
Lipid Metabolism	TG	rs569884373	2	27555227	C	T	0.170	0.031	4.6e-08	7.3e-05	29.9
Lipid Metabolism	TG	rs3739095	2	27556721	A	G	-0.066	0.001	1.0e-200	1.8e-03	2261.6
Lipid Metabolism	TG	rs559530894	2	27561346	T	C	0.128	0.021	1.8e-09	3.6e-05	36.2
Lipid Metabolism	TG	rs186953711	2	27571211	A	G	0.102	0.018	6.6e-09	4.0e-05	33.6
Lipid Metabolism	TG	rs192462587	2	27652700	A	G	-0.089	0.013	2.1e-11	3.7e-05	44.9
Lipid Metabolism	TG	rs186952957	2	27655894	A	G	-0.062	0.011	3.2e-09	3.0e-05	35.1
Lipid Metabolism	TG	rs145761128	2	27695484	A	G	-0.126	0.020	7.8e-10	4.5e-05	37.8
Lipid Metabolism	TG	rs554639259	2	27727719	C	T	0.169	0.026	7.6e-11	4.1e-05	42.4
Lipid Metabolism	TG	rs183573614	2	27734458	T	C	0.119	0.012	1.1e-22	8.2e-05	96.2
Lipid Metabolism	TG	rs144176364	2	27842159	T	A	0.095	0.013	2.0e-12	5.2e-05	49.5
Lipid Metabolism	TG	rs546365681	2	27879470	C	T	0.091	0.016	1.2e-08	2.7e-05	32.5
Lipid Metabolism	TG	rs554500575	2	27900936	G	A	0.071	0.010	8.6e-12	3.8e-05	46.6
Lipid Metabolism	TG	rs577232373	2	27937346	G	A	-0.129	0.017	1.1e-14	6.5e-05	59.7
Lipid Metabolism	TG	rs13013484	2	27988821	A	G	0.045	0.002	5.7e-190	6.9e-04	864.3
Lipid Metabolism	TG	rs188149359	2	28068999	C	T	0.109	0.019	1.7e-08	3.7e-05	31.8
Lipid Metabolism	TG	rs560102432	2	28118614	G	T	-0.092	0.011	2.2e-16	6.1e-05	67.4
Lipid Metabolism	TG	rs184444512	2	28147230	G	A	-0.089	0.015	8.7e-10	3.1e-05	37.6
Lipid Metabolism	TG	rs34722407	2	28336244	A	G	0.049	0.002	4.5e-102	3.7e-04	460.1
Lipid Metabolism	TG	rs561615284	2	28388504	C	G	-0.056	0.009	1.5e-09	3.0e-05	36.5
Lipid Metabolism	TG	rs186272193	2	28433175	T	C	0.084	0.013	1.4e-10	3.4e-05	41.1
Lipid Metabolism	TG	rs114248375	2	28446850	T	G	-0.037	0.003	3.5e-29	1.0e-04	125.7
Lipid Metabolism	TG	rs139783901	2	28448948	A	G	0.092	0.016	2.0e-08	2.6e-05	31.5
Lipid Metabolism	TG	rs148697092	2	28550879	C	T	0.086	0.014	4.3e-10	3.3e-05	39.0
Lipid Metabolism	TG	rs184499635	2	28578354	A	G	0.078	0.012	1.3e-10	3.4e-05	41.4
Lipid Metabolism	TG	rs35126720	2	28589360	A	G	0.064	0.009	1.4e-12	4.1e-05	50.2
Lipid Metabolism	TG	rs7590263	2	28645802	T	C	0.011	0.001	4.2e-14	4.7e-05	57.1
Lipid Metabolism	TG	rs555063792	2	28720690	G	A	0.047	0.008	9.2e-09	2.7e-05	33.0
Lipid Metabolism	TG	rs2716712	2	39858533	T	C	-0.008	0.001	5.2e-09	2.8e-05	34.1
Lipid Metabolism	TG	rs6737575	2	42682581	G	C	-0.009	0.002	1.2e-08	2.6e-05	32.5
Lipid Metabolism	TG	rs72616903	2	43743012	C	T	0.015	0.002	4.1e-15	4.9e-05	61.7
Lipid Metabolism	TG	rs17326656	2	48962291	T	G	0.013	0.002	1.0e-14	4.8e-05	59.8
Lipid Metabolism	TG	rs2111068	2	50717885	G	C	0.009	0.001	2.2e-09	2.9e-05	35.8
Lipid Metabolism	TG	rs1861410	2	58933591	T	C	-0.011	0.001	6.0e-15	4.9e-05	60.9
Lipid Metabolism	TG	rs10172678	2	59294558	C	T	-0.010	0.001	9.4e-12	3.7e-05	46.5
Lipid Metabolism	TG	rs7564220	2	60823556	A	C	0.010	0.002	2.4e-08	2.5e-05	31.1
Lipid Metabolism	TG	rs7570707	2	61451744	T	C	0.010	0.001	2.6e-12	3.9e-05	49.0
Lipid Metabolism	TG	rs6744311	2	64961036	G	A	0.009	0.001	2.7e-09	2.8e-05	35.4
Lipid Metabolism	TG	rs2723064	2	65279805	C	T	-0.020	0.001	7.0e-45	1.6e-04	197.6
Lipid Metabolism	TG	rs840974	2	65705581	A	G	-0.009	0.001	1.2e-10	3.3e-05	41.5
Lipid Metabolism	TG	rs11126079	2	66676367	C	G	-0.010	0.001	9.8e-12	3.7e-05	46.4
Lipid Metabolism	TG	rs66584237	2	71229195	A	G	0.010	0.002	2.3e-09	2.9e-05	35.7
Lipid Metabolism	TG	rs55772482	2	105954140	A	G	0.012	0.002	4.5e-09	2.7e-05	34.4
Lipid Metabolism	TG	rs3789065	2	111915022	G	C	-0.013	0.001	1.7e-20	6.9e-05	86.1
Lipid Metabolism	TG	rs4353657	2	112760944	A	C	-0.009	0.001	2.2e-10	3.2e-05	40.3
Lipid Metabolism	TG	rs13409360	2	113838102	A	G	-0.010	0.001	2.6e-13	4.3e-05	53.5
Lipid Metabolism	TG	rs138033166	2	119755849	G	A	0.043	0.006	1.1e-13	4.4e-05	55.2
Lipid Metabolism	TG	rs7578604	2	121308660	T	G	0.016	0.002	4.8e-24	8.2e-05	102.3
Lipid Metabolism	TG	rs56664075	2	121438750	T	C	-0.019	0.003	4.9e-11	3.5e-05	43.2
Lipid Metabolism	TG	rs12995180	2	145814198	G	A	-0.009	0.001	6.2e-09	2.7e-05	33.8
Lipid Metabolism	TG	rs7568603	2	146352541	G	T	-0.010	0.001	1.7e-13	4.4e-05	54.4
Lipid Metabolism	TG	rs12694933	2	158525226	T	C	0.012	0.002	1.2e-08	2.6e-05	32.4
Lipid Metabolism	TG	rs10181181	2	161087411	C	T	0.011	0.002	1.7e-12	4.0e-05	49.8
Lipid Metabolism	TG	rs671689	2	165352405	A	G	-0.011	0.001	4.2e-13	4.2e-05	52.6
Lipid Metabolism	TG	rs114544257	2	165419705	C	T	-0.048	0.008	2.7e-09	2.8e-05	35.4

Phenotype	Trait	SNP	Chr	Position (GRCh37/hg19)	EA	NEA	BETA	SE	P-value	R2	F
Lipid Metabolism	TG	rs13389219	2	165528876	T	C	-0.034	0.001	2.3e-127	4.6e-04	576.4
Lipid Metabolism	TG	rs355799	2	165694898	T	C	-0.017	0.002	6.9e-19	6.3e-05	78.8
Lipid Metabolism	TG	rs554494	2	169729563	A	G	-0.009	0.001	4.3e-10	3.1e-05	39.0
Lipid Metabolism	TG	rs6433249	2	171639622	A	T	-0.010	0.001	8.1e-12	3.8e-05	46.7
Lipid Metabolism	TG	rs77625597	2	175243224	C	G	-0.010	0.002	3.7e-08	2.4e-05	30.3
Lipid Metabolism	TG	rs16867014	2	180674147	C	T	-0.010	0.001	5.2e-11	3.4e-05	43.1
Lipid Metabolism	TG	rs17576323	2	188003118	C	T	-0.010	0.002	4.1e-09	2.8e-05	34.6
Lipid Metabolism	TG	rs72913721	2	193859247	G	A	-0.009	0.002	4.6e-08	2.4e-05	29.9
Lipid Metabolism	TG	rs1918881	2	202190668	A	G	0.009	0.001	8.4e-11	3.4e-05	42.2
Lipid Metabolism	TG	rs3731696	2	203431804	G	A	0.017	0.002	1.6e-16	5.4e-05	68.1
Lipid Metabolism	TG	rs7581601	2	203497786	C	A	-0.008	0.001	1.0e-08	2.6e-05	32.8
Lipid Metabolism	TG	rs10207710	2	206543852	A	T	-0.008	0.001	3.8e-09	2.8e-05	34.7
Lipid Metabolism	TG	rs57314858	2	208031471	T	C	0.008	0.001	4.3e-09	2.8e-05	34.5
Lipid Metabolism	TG	rs78584857	2	213114091	A	G	-0.009	0.002	4.1e-08	2.4e-05	30.1
Lipid Metabolism	TG	rs6760280	2	215349689	G	A	0.008	0.001	6.0e-09	2.7e-05	33.8
Lipid Metabolism	TG	rs2024487	2	217663271	C	T	0.008	0.001	3.4e-08	2.4e-05	30.5
Lipid Metabolism	TG	rs10165153	2	219159629	C	T	-0.008	0.001	6.2e-09	2.7e-05	33.8
Lipid Metabolism	TG	rs186838921	2	219474183	G	A	0.095	0.017	2.6e-08	2.7e-05	30.9
Lipid Metabolism	TG	rs6436066	2	219578361	G	T	-0.016	0.001	2.5e-31	1.1e-04	135.5
Lipid Metabolism	TG	rs78058190	2	219699999	A	G	0.077	0.004	9.1e-104	3.8e-04	467.9
Lipid Metabolism	TG	rs2972146	2	227100698	T	G	0.034	0.001	1.6e-128	4.6e-04	581.7
Lipid Metabolism	TG	rs10933140	2	227209828	G	A	-0.014	0.002	4.2e-17	5.7e-05	70.7
Lipid Metabolism	TG	rs7604426	2	230123353	G	A	-0.009	0.002	1.4e-08	2.6e-05	32.3
Lipid Metabolism	TG	rs13395911	2	233520254	T	A	-0.008	0.001	2.1e-08	2.5e-05	31.4
Lipid Metabolism	TG	rs35203651	2	234679549	C	T	0.013	0.002	4.4e-09	2.8e-05	34.5
Lipid Metabolism	TG	rs2305076	2	242186391	T	C	0.013	0.002	1.0e-08	2.6e-05	32.8
Lipid Metabolism	TG	rs7645905	3	4758244	G	T	-0.012	0.001	5.8e-17	5.6e-05	70.0
Lipid Metabolism	TG	rs62246311	3	9498143	A	G	0.016	0.002	1.0e-12	4.2e-05	50.8
Lipid Metabolism	TG	rs9848170	3	11495983	C	G	0.008	0.001	3.9e-08	2.4e-05	30.2
Lipid Metabolism	TG	rs1584063	3	12266855	A	G	-0.011	0.001	1.1e-15	5.3e-05	64.3
Lipid Metabolism	TG	rs4684847	3	12386337	T	C	-0.027	0.002	2.7e-39	1.4e-04	172.0
Lipid Metabolism	TG	rs147070419	3	12442098	G	T	0.083	0.009	1.9e-18	6.2e-05	76.8
Lipid Metabolism	TG	rs73029206	3	12494894	G	A	-0.016	0.002	1.4e-16	5.5e-05	68.3
Lipid Metabolism	TG	rs148153324	3	12629892	T	C	0.048	0.008	9.3e-10	3.0e-05	37.5
Lipid Metabolism	TG	rs7640807	3	15685137	C	T	0.009	0.001	5.4e-11	3.4e-05	43.0
Lipid Metabolism	TG	rs11717397	3	23368583	A	G	0.009	0.001	4.7e-10	3.1e-05	38.8
Lipid Metabolism	TG	rs2044755	3	24292926	A	T	0.014	0.002	1.0e-11	3.7e-05	46.2
Lipid Metabolism	TG	rs6792725	3	24520283	G	A	-0.016	0.002	5.9e-25	8.7e-05	106.4
Lipid Metabolism	TG	rs4678556	3	37007396	G	C	-0.010	0.001	8.5e-14	4.5e-05	55.7
Lipid Metabolism	TG	rs114584454	3	39222510	G	T	0.010	0.002	4.7e-09	2.7e-05	34.3
Lipid Metabolism	TG	rs60488096	3	46050355	A	C	0.009	0.002	2.9e-08	2.5e-05	30.8
Lipid Metabolism	TG	rs111970477	3	47105581	G	A	0.031	0.006	4.2e-08	2.4e-05	30.1
Lipid Metabolism	TG	rs2267846	3	48556339	A	G	-0.012	0.002	1.8e-14	4.7e-05	58.7
Lipid Metabolism	TG	rs56049603	3	49585243	G	C	-0.014	0.002	4.6e-16	5.3e-05	66.0
Lipid Metabolism	TG	rs115506546	3	49808007	G	A	0.023	0.003	4.4e-12	3.9e-05	48.0
Lipid Metabolism	TG	rs73084757	3	50589611	G	A	-0.017	0.003	5.1e-10	3.1e-05	38.6
Lipid Metabolism	TG	rs73080631	3	51708144	T	G	-0.017	0.003	4.0e-09	2.8e-05	34.6
Lipid Metabolism	TG	rs17052061	3	52346240	G	T	-0.020	0.002	5.6e-28	9.6e-05	120.2
Lipid Metabolism	TG	rs6445923	3	57655009	T	C	0.011	0.002	6.0e-09	2.7e-05	33.8
Lipid Metabolism	TG	rs76824303	3	62459819	C	A	-0.015	0.002	9.5e-10	3.1e-05	37.4
Lipid Metabolism	TG	rs7639854	3	66868310	T	C	0.012	0.002	2.4e-14	4.6e-05	58.1
Lipid Metabolism	TG	rs62251038	3	69868660	G	A	0.009	0.001	2.1e-10	3.2e-05	40.3
Lipid Metabolism	TG	rs13066793	3	87037543	G	A	-0.018	0.002	5.0e-13	4.2e-05	52.2
Lipid Metabolism	TG	rs10934286	3	115090105	G	A	0.012	0.002	2.8e-08	2.5e-05	30.8
Lipid Metabolism	TG	rs56724294	3	129341193	T	C	-0.011	0.002	1.5e-10	3.3e-05	41.0
Lipid Metabolism	TG	rs2685183	3	130603680	T	C	0.008	0.001	3.0e-08	2.5e-05	30.7

Phenotype	Trait	SNP	Chr	Position (GRCh37/hg19)	EA	NEA	BETA	SE	P-value	R2	F
Lipid Metabolism	TG	rs548288	3	135969755	C	T	0.024	0.002	7.1e-45	1.6e-04	197.6
Lipid Metabolism	TG	rs34773643	3	141685947	T	C	-0.011	0.002	1.1e-08	2.6e-05	32.6
Lipid Metabolism	TG	rs9653945	3	142660706	A	G	-0.013	0.001	6.0e-20	6.7e-05	83.6
Lipid Metabolism	TG	rs62271373	3	150066540	A	T	0.035	0.003	4.1e-29	1.0e-04	125.4
Lipid Metabolism	TG	rs358702	3	155542475	A	G	0.012	0.002	3.8e-15	4.9e-05	61.8
Lipid Metabolism	TG	rs9817452	3	156795414	T	G	-0.018	0.001	5.5e-36	1.3e-04	156.9
Lipid Metabolism	TG	rs3113	3	156814949	C	T	0.010	0.001	1.3e-11	3.7e-05	45.9
Lipid Metabolism	TG	rs11924648	3	170717996	G	A	0.020	0.002	3.3e-23	7.9e-05	98.5
Lipid Metabolism	TG	rs79287178	3	172294500	A	G	0.060	0.004	3.2e-46	1.6e-04	203.7
Lipid Metabolism	TG	rs16859708	3	184886137	C	T	0.013	0.002	1.8e-10	3.3e-05	40.7
Lipid Metabolism	TG	rs3772109	3	196005229	C	T	0.009	0.001	7.2e-10	3.1e-05	38.0
Lipid Metabolism	TG	rs34311866	4	951947	C	T	0.017	0.002	2.6e-22	7.6e-05	94.4
Lipid Metabolism	TG	rs78139882	4	3287361	T	C	0.012	0.002	7.5e-12	3.7e-05	46.9
Lipid Metabolism	TG	rs59950280	4	3452345	A	G	0.029	0.002	6.0e-82	3.0e-04	367.7
Lipid Metabolism	TG	rs731450	4	3490328	T	C	0.015	0.001	2.9e-25	8.7e-05	107.9
Lipid Metabolism	TG	rs4450871	4	4990298	G	A	-0.015	0.002	7.5e-22	8.0e-05	92.3
Lipid Metabolism	TG	rs182629106	4	15070632	C	T	0.070	0.013	4.0e-08	2.5e-05	30.2
Lipid Metabolism	TG	rs71603400	4	18032493	A	G	0.021	0.002	1.7e-24	8.3e-05	104.3
Lipid Metabolism	TG	rs73243877	4	26047616	G	A	0.021	0.002	2.6e-31	1.1e-04	135.5
Lipid Metabolism	TG	rs10939110	4	26418638	T	C	-0.008	0.001	1.2e-08	2.6e-05	32.4
Lipid Metabolism	TG	rs114447556	4	53207093	T	C	0.015	0.003	4.3e-08	2.4e-05	30.0
Lipid Metabolism	TG	rs7684939	4	55509189	A	G	-0.009	0.001	1.5e-11	3.7e-05	45.6
Lipid Metabolism	TG	rs35662434	4	69339933	G	A	0.017	0.002	9.7e-23	8.0e-05	96.3
Lipid Metabolism	TG	rs12650728	4	69542100	T	C	0.015	0.002	3.8e-13	4.3e-05	52.8
Lipid Metabolism	TG	rs185620092	4	69595297	A	G	-0.060	0.011	4.0e-08	2.7e-05	30.2
Lipid Metabolism	TG	rs4694362	4	71893864	T	C	0.009	0.001	9.2e-10	3.0e-05	37.5
Lipid Metabolism	TG	rs187918276	4	74033564	C	G	0.158	0.021	1.5e-13	5.9e-05	54.6
Lipid Metabolism	TG	rs13146355	4	77412140	A	G	0.010	0.001	6.4e-14	4.5e-05	56.3
Lipid Metabolism	TG	rs7691492	4	84411233	T	C	-0.008	0.001	3.4e-08	2.5e-05	30.5
Lipid Metabolism	TG	rs7439032	4	86931091	C	T	-0.014	0.002	3.7e-14	4.7e-05	57.3
Lipid Metabolism	TG	rs17605615	4	87996745	A	G	0.027	0.001	1.9e-83	3.0e-04	374.6
Lipid Metabolism	TG	rs411365	4	88067124	A	G	0.011	0.001	1.7e-15	5.2e-05	63.4
Lipid Metabolism	TG	rs1031794	4	88451182	T	C	0.012	0.001	6.5e-19	6.3e-05	78.9
Lipid Metabolism	TG	rs2167750	4	89730074	T	C	0.012	0.001	1.0e-18	6.2e-05	78.1
Lipid Metabolism	TG	rs12644723	4	95942505	A	G	-0.008	0.001	1.5e-08	2.6e-05	32.0
Lipid Metabolism	TG	rs1126673	4	100045616	T	C	0.014	0.001	1.2e-21	7.3e-05	91.4
Lipid Metabolism	TG	rs146635149	4	100441465	A	G	-0.015	0.002	7.8e-10	3.0e-05	37.8
Lipid Metabolism	TG	rs35225200	4	103146888	C	A	0.034	0.003	2.3e-36	1.3e-04	158.6
Lipid Metabolism	TG	rs4699038	4	103879412	A	G	-0.012	0.002	1.6e-12	4.0e-05	49.9
Lipid Metabolism	TG	rs12645144	4	106152111	G	T	0.008	0.001	1.6e-08	2.6e-05	32.0
Lipid Metabolism	TG	rs149800	4	110515471	A	G	-0.009	0.002	8.4e-10	3.0e-05	37.7
Lipid Metabolism	TG	rs114816312	4	110638824	T	C	0.134	0.009	9.5e-50	1.8e-04	219.9
Lipid Metabolism	TG	rs113824614	4	110888661	A	G	0.100	0.016	1.1e-09	4.6e-05	37.2
Lipid Metabolism	TG	rs3813829	4	111120166	G	A	0.012	0.002	2.5e-14	4.7e-05	58.1
Lipid Metabolism	TG	rs192929239	4	124641827	G	A	0.046	0.006	1.2e-13	4.4e-05	55.1
Lipid Metabolism	TG	rs74647354	4	124793787	T	G	0.013	0.002	9.6e-10	3.0e-05	37.4
Lipid Metabolism	TG	rs2874600	4	141818185	C	G	-0.008	0.001	1.0e-08	2.6e-05	32.7
Lipid Metabolism	TG	rs10006971	4	143318853	G	A	-0.011	0.001	5.3e-16	5.3e-05	65.7
Lipid Metabolism	TG	rs1509333	4	147850007	C	T	-0.008	0.001	4.7e-09	2.7e-05	34.3
Lipid Metabolism	TG	rs3775327	4	148985720	C	T	0.010	0.002	6.8e-11	3.4e-05	42.6
Lipid Metabolism	TG	rs534659996	4	155308673	G	T	0.114	0.016	3.5e-13	4.6e-05	52.9
Lipid Metabolism	TG	rs6054	4	155489608	T	C	0.118	0.011	3.9e-28	1.0e-04	121.0
Lipid Metabolism	TG	rs4691378	4	157704459	G	A	-0.014	0.001	5.2e-22	7.4e-05	93.0
Lipid Metabolism	TG	rs6866562	5	52697383	A	G	0.008	0.001	2.5e-08	2.5e-05	31.0
Lipid Metabolism	TG	rs79760705	5	53298716	T	G	0.026	0.002	2.3e-32	1.1e-04	140.3
Lipid Metabolism	TG	rs255761	5	53321956	T	G	0.014	0.002	4.2e-17	5.6e-05	70.7

Phenotype	Trait	SNP	Chr	Position (GRCh37/hg19)	EA	NEA	BETA	SE	P-value	R2	F
Lipid Metabolism	TG	rs40270	5	55804552	C	A	0.029	0.002	1.1e-73	2.7e-04	329.7
Lipid Metabolism	TG	rs188010585	5	55858239	C	T	-0.040	0.007	5.8e-09	2.7e-05	33.9
Lipid Metabolism	TG	rs28650790	5	55861464	T	C	0.038	0.002	2.6e-103	3.7e-04	465.8
Lipid Metabolism	TG	rs34196215	5	55965964	C	T	-0.015	0.002	6.4e-14	4.5e-05	56.2
Lipid Metabolism	TG	rs6450398	5	55998242	T	C	-0.009	0.001	5.9e-10	3.1e-05	38.3
Lipid Metabolism	TG	rs152012	5	57620409	C	T	-0.011	0.001	7.9e-16	5.2e-05	64.9
Lipid Metabolism	TG	rs62366190	5	60483935	G	C	-0.009	0.001	2.2e-10	3.2e-05	40.3
Lipid Metabolism	TG	rs10038774	5	67696345	C	T	0.013	0.002	2.7e-19	6.4e-05	80.7
Lipid Metabolism	TG	rs246562	5	71810246	A	G	0.010	0.002	1.2e-08	2.6e-05	32.4
Lipid Metabolism	TG	rs10052346	5	78472599	T	G	-0.011	0.001	3.5e-15	5.1e-05	62.0
Lipid Metabolism	TG	rs115912456	5	82815158	G	A	-0.027	0.003	9.9e-16	5.1e-05	64.5
Lipid Metabolism	TG	rs12653396	5	87847273	A	T	0.008	0.001	1.8e-09	2.9e-05	36.2
Lipid Metabolism	TG	rs34088804	5	90210432	G	A	-0.010	0.002	5.2e-11	3.4e-05	43.1
Lipid Metabolism	TG	rs6881840	5	97441259	G	A	-0.008	0.001	4.8e-08	2.4e-05	29.8
Lipid Metabolism	TG	rs288178	5	107351517	C	T	-0.008	0.001	8.6e-09	2.6e-05	33.1
Lipid Metabolism	TG	rs7732198	5	112379659	C	T	0.009	0.001	3.8e-10	3.1e-05	39.2
Lipid Metabolism	TG	rs6595187	5	118723609	G	A	-0.019	0.002	1.2e-36	1.3e-04	159.8
Lipid Metabolism	TG	rs17764730	5	127357526	T	C	-0.011	0.002	7.3e-12	3.8e-05	47.0
Lipid Metabolism	TG	rs253942	5	131320462	T	C	0.025	0.003	2.0e-18	6.1e-05	76.7
Lipid Metabolism	TG	rs141197607	5	131338023	A	C	-0.083	0.012	1.3e-11	3.8e-05	45.9
Lipid Metabolism	TG	rs3900685	5	131431746	A	C	-0.056	0.009	6.9e-10	3.1e-05	38.1
Lipid Metabolism	TG	rs270612	5	131637338	A	G	-0.010	0.002	1.8e-10	3.3e-05	40.7
Lipid Metabolism	TG	rs72801474	5	132444128	A	G	-0.030	0.002	1.5e-36	1.3e-04	159.5
Lipid Metabolism	TG	rs11242227	5	134135076	G	A	0.014	0.002	1.4e-10	3.3e-05	41.2
Lipid Metabolism	TG	rs143322232	5	139766996	G	A	-0.011	0.002	8.3e-12	3.7e-05	46.7
Lipid Metabolism	TG	rs13163489	5	141704370	C	A	-0.010	0.001	1.6e-11	3.7e-05	45.4
Lipid Metabolism	TG	rs6865870	5	144498018	G	A	0.009	0.002	4.0e-09	2.8e-05	34.6
Lipid Metabolism	TG	rs62388970	5	147908385	A	G	-0.008	0.001	4.9e-08	2.4e-05	29.8
Lipid Metabolism	TG	rs245078	5	149353598	G	A	-0.010	0.001	3.8e-12	3.9e-05	48.2
Lipid Metabolism	TG	rs576105320	5	155928536	G	A	-0.098	0.018	4.0e-08	3.8e-05	30.1
Lipid Metabolism	TG	rs72803088	5	156275390	G	C	0.025	0.004	1.3e-10	3.3e-05	41.3
Lipid Metabolism	TG	rs113519918	5	156339114	G	T	-0.071	0.006	3.2e-29	1.0e-04	125.9
Lipid Metabolism	TG	rs11134475	5	156399950	G	A	0.034	0.001	2.1e-128	4.7e-04	581.2
Lipid Metabolism	TG	rs193010652	5	156410046	A	C	0.138	0.019	2.3e-13	4.6e-05	53.7
Lipid Metabolism	TG	rs2963468	5	158003020	G	A	0.022	0.002	3.2e-41	1.4e-04	180.8
Lipid Metabolism	TG	rs72812818	5	173356752	C	G	0.011	0.001	2.6e-13	4.3e-05	53.5
Lipid Metabolism	TG	rs12652509	5	176626441	G	A	-0.014	0.002	7.0e-19	6.4e-05	78.8
Lipid Metabolism	TG	rs75479205	6	7255610	G	A	-0.011	0.002	1.3e-10	3.4e-05	41.3
Lipid Metabolism	TG	rs58640099	6	12835141	A	G	-0.009	0.002	7.6e-10	3.5e-05	37.9
Lipid Metabolism	TG	rs9477720	6	18358686	G	T	-0.008	0.001	9.7e-10	3.0e-05	37.4
Lipid Metabolism	TG	rs1012441	6	18682998	G	A	-0.010	0.002	6.9e-11	3.4e-05	42.5
Lipid Metabolism	TG	rs6456352	6	20455280	A	G	-0.016	0.002	1.1e-10	3.3e-05	41.7
Lipid Metabolism	TG	rs9348441	6	20680678	A	T	0.012	0.002	2.4e-14	4.7e-05	58.2
Lipid Metabolism	TG	rs806973	6	26148326	G	A	-0.009	0.001	5.4e-11	3.5e-05	43.0
Lipid Metabolism	TG	rs58825580	6	26365679	G	T	-0.012	0.002	2.8e-08	2.5e-05	30.9
Lipid Metabolism	TG	rs34864796	6	27459923	A	G	-0.016	0.002	2.1e-12	3.9e-05	49.4
Lipid Metabolism	TG	rs144130050	6	28264188	C	T	0.038	0.007	1.2e-08	2.8e-05	32.5
Lipid Metabolism	TG	rs3131345	6	28825571	A	G	-0.020	0.003	9.5e-15	4.8e-05	60.0
Lipid Metabolism	TG	rs61730327	6	29640951	C	T	0.039	0.006	3.7e-11	3.8e-05	43.7
Lipid Metabolism	TG	rs2428506	6	30229768	C	A	-0.012	0.001	3.1e-16	5.8e-05	66.8
Lipid Metabolism	TG	rs3132631	6	30344645	T	C	-0.024	0.002	1.2e-25	9.5e-05	109.5
Lipid Metabolism	TG	rs11968046	6	31302642	T	C	0.032	0.002	8.4e-80	2.9e-04	357.8
Lipid Metabolism	TG	rs7773105	6	31329372	G	A	0.022	0.002	4.6e-39	1.4e-04	170.9
Lipid Metabolism	TG	rs2844546	6	31344657	A	G	-0.009	0.001	9.0e-10	3.0e-05	37.5
Lipid Metabolism	TG	rs2523675	6	31436032	A	G	0.023	0.002	5.4e-52	2.0e-04	230.2
Lipid Metabolism	TG	rs71563353	6	31562924	T	C	0.032	0.003	1.7e-27	1.0e-04	118.1

Phenotype	Trait	SNP	Chr	Position (GRCh37/hg19)	EA	NEA	BETA	SE	P-value	R2	F
Lipid Metabolism	TG	rs9461755	6	32441046	A	G	0.076	0.003	2.3e-105	4.1e-04	475.2
Lipid Metabolism	TG	rs9271573	6	32590501	C	A	0.029	0.001	7.0e-87	3.3e-04	390.3
Lipid Metabolism	TG	rs9275638	6	32684760	T	C	0.023	0.002	1.0e-26	9.7e-05	114.4
Lipid Metabolism	TG	rs531054135	6	32876079	G	A	0.073	0.013	2.0e-08	4.2e-05	31.5
Lipid Metabolism	TG	rs12206946	6	33677649	A	G	0.051	0.006	1.1e-18	6.3e-05	77.9
Lipid Metabolism	TG	rs10947433	6	33689629	A	G	-0.018	0.003	4.8e-10	3.1e-05	38.8
Lipid Metabolism	TG	rs116839142	6	34258571	G	T	0.029	0.004	3.0e-11	3.6e-05	44.2
Lipid Metabolism	TG	rs6934083	6	35237153	T	C	0.026	0.004	1.8e-12	4.0e-05	49.7
Lipid Metabolism	TG	rs4135240	6	36647680	C	T	-0.010	0.001	5.8e-12	3.8e-05	47.4
Lipid Metabolism	TG	rs742493	6	40998167	C	T	-0.017	0.002	3.9e-15	4.9e-05	61.7
Lipid Metabolism	TG	rs3025047	6	43746410	T	C	0.047	0.006	2.4e-13	4.3e-05	53.6
Lipid Metabolism	TG	rs9472125	6	43756169	T	C	-0.042	0.002	1.1e-71	2.6e-04	320.6
Lipid Metabolism	TG	rs68137036	6	43820215	G	A	-0.018	0.002	1.3e-31	1.1e-04	136.8
Lipid Metabolism	TG	rs2749024	6	52630587	A	T	-0.018	0.001	1.0e-36	1.3e-04	160.2
Lipid Metabolism	TG	rs6458951	6	53508627	C	T	0.009	0.002	3.1e-08	2.4e-05	30.7
Lipid Metabolism	TG	rs73744635	6	55437238	C	T	-0.018	0.003	4.3e-08	2.4e-05	30.0
Lipid Metabolism	TG	rs6454486	6	86433587	T	C	-0.011	0.001	1.0e-13	4.5e-05	55.3
Lipid Metabolism	TG	rs2092094	6	96844123	C	G	-0.011	0.002	1.2e-08	2.6e-05	32.5
Lipid Metabolism	TG	rs12194350	6	100058725	T	C	0.011	0.002	1.2e-08	2.6e-05	32.5
Lipid Metabolism	TG	rs9496567	6	100602753	A	G	-0.012	0.002	1.0e-13	4.4e-05	55.3
Lipid Metabolism	TG	rs10872003	6	106378449	A	T	-0.010	0.002	5.8e-11	4.0e-05	42.9
Lipid Metabolism	TG	rs35045014	6	107432157	A	C	0.014	0.001	4.1e-21	7.5e-05	88.9
Lipid Metabolism	TG	rs75538668	6	109115827	C	T	0.013	0.002	1.9e-11	3.6e-05	45.1
Lipid Metabolism	TG	rs2768539	6	109195215	A	G	0.014	0.002	9.0e-17	5.6e-05	69.2
Lipid Metabolism	TG	rs781495	6	111245074	C	T	-0.010	0.002	6.0e-10	3.1e-05	38.3
Lipid Metabolism	TG	rs193282	6	111543067	A	G	0.008	0.001	3.2e-09	2.8e-05	35.0
Lipid Metabolism	TG	rs9401908	6	127105492	G	A	-0.016	0.001	7.0e-32	1.1e-04	138.1
Lipid Metabolism	TG	rs577721086	6	127440047	C	T	0.053	0.003	2.9e-63	2.3e-04	281.9
Lipid Metabolism	TG	rs1415700	6	130345791	A	G	0.014	0.002	2.3e-21	7.2e-05	90.1
Lipid Metabolism	TG	rs71574648	6	133571382	G	A	-0.012	0.002	4.7e-08	2.4e-05	29.8
Lipid Metabolism	TG	rs212775	6	133797863	G	A	0.008	0.001	2.2e-09	2.9e-05	35.8
Lipid Metabolism	TG	rs672457	6	139836562	A	G	-0.026	0.001	4.1e-80	2.9e-04	359.2
Lipid Metabolism	TG	rs9321765	6	140595744	C	T	-0.013	0.002	1.4e-13	4.4e-05	54.7
Lipid Metabolism	TG	rs11759804	6	151875122	T	C	0.012	0.002	4.0e-08	2.4e-05	30.2
Lipid Metabolism	TG	rs1281953	6	153462029	T	A	-0.010	0.001	2.8e-12	3.9e-05	48.8
Lipid Metabolism	TG	rs73025562	6	160562481	A	G	0.014	0.002	4.7e-19	6.4e-05	79.6
Lipid Metabolism	TG	rs2504958	6	160750545	T	C	-0.020	0.002	4.5e-33	1.1e-04	143.5
Lipid Metabolism	TG	rs10455872	6	161010118	G	A	-0.021	0.003	2.4e-15	5.0e-05	62.7
Lipid Metabolism	TG	rs11751347	6	161092438	T	C	0.039	0.002	3.8e-62	2.2e-04	276.7
Lipid Metabolism	TG	rs186696265	6	161111700	T	C	-0.046	0.006	2.2e-14	4.7e-05	58.4
Lipid Metabolism	TG	rs34709632	6	163048380	G	A	-0.012	0.002	3.3e-08	2.4e-05	30.5
Lipid Metabolism	TG	rs73029263	6	164113762	G	A	-0.019	0.002	1.8e-20	6.9e-05	86.0
Lipid Metabolism	TG	rs1472266	6	166184881	G	T	0.009	0.001	8.0e-10	3.1e-05	37.8
Lipid Metabolism	TG	rs2461725	6	166317884	A	G	-0.009	0.002	4.9e-08	2.4e-05	29.8
Lipid Metabolism	TG	rs56208327	7	1128011	C	G	-0.023	0.004	1.1e-08	2.7e-05	32.6
Lipid Metabolism	TG	rs2966450	7	5569715	A	G	0.009	0.001	1.3e-10	3.3e-05	41.2
Lipid Metabolism	TG	rs5011431	7	12268551	A	G	0.009	0.001	1.3e-11	3.7e-05	45.9
Lipid Metabolism	TG	rs38190	7	15903720	T	C	-0.012	0.001	9.7e-18	5.9e-05	73.6
Lipid Metabolism	TG	rs6461213	7	16027998	G	A	-0.012	0.002	3.6e-13	4.2e-05	52.8
Lipid Metabolism	TG	rs4410790	7	17284577	C	T	0.013	0.001	1.6e-20	6.9e-05	86.2
Lipid Metabolism	TG	rs6461354	7	17914600	T	C	0.013	0.001	3.7e-20	6.8e-05	84.6
Lipid Metabolism	TG	rs16872619	7	20831800	G	A	0.012	0.002	1.4e-09	2.9e-05	36.7
Lipid Metabolism	TG	rs55649657	7	21607283	G	C	0.012	0.002	2.5e-12	3.9e-05	49.0
Lipid Metabolism	TG	rs7786102	7	25965759	A	G	-0.022	0.002	3.0e-45	1.6e-04	199.3
Lipid Metabolism	TG	rs1406754	7	26396198	T	G	-0.015	0.001	6.6e-27	9.3e-05	115.4
Lipid Metabolism	TG	rs849336	7	28224053	G	A	-0.010	0.001	9.0e-13	4.1e-05	51.0

Phenotype	Trait	SNP	Chr	Position (GRCh37/hg19)	EA	NEA	BETA	SE	P-value	R2	F
Lipid Metabolism	TG	rs2070971	7	44197583	T	G	0.029	0.002	7.4e-49	1.7e-04	215.8
Lipid Metabolism	TG	rs78785345	7	44222360	A	G	-0.039	0.007	2.5e-08	2.5e-05	31.1
Lipid Metabolism	TG	rs306999	7	44538713	G	A	0.012	0.002	6.4e-13	4.1e-05	51.7
Lipid Metabolism	TG	rs13237404	7	46331207	A	G	0.014	0.003	4.3e-08	2.4e-05	30.0
Lipid Metabolism	TG	rs71551223	7	71256489	C	A	-0.037	0.004	1.1e-18	6.2e-05	77.8
Lipid Metabolism	TG	rs77891554	7	71444550	C	G	0.015	0.003	1.4e-08	2.6e-05	32.2
Lipid Metabolism	TG	rs118167704	7	71650012	G	A	0.020	0.003	2.2e-11	3.6e-05	44.8
Lipid Metabolism	TG	rs73129519	7	71721627	C	T	0.035	0.005	1.2e-10	3.3e-05	41.5
Lipid Metabolism	TG	rs73129534	7	71728551	C	T	-0.034	0.006	6.4e-09	2.7e-05	33.7
Lipid Metabolism	TG	rs112299859	7	71865494	C	T	-0.084	0.009	4.6e-21	7.2e-05	88.7
Lipid Metabolism	TG	rs576720725	7	72121608	T	G	-0.112	0.020	2.5e-08	3.9e-05	31.1
Lipid Metabolism	TG	rs528469919	7	72156022	T	C	-0.102	0.015	6.3e-12	4.0e-05	47.2
Lipid Metabolism	TG	rs62466873	7	72220596	A	G	0.028	0.005	2.6e-09	2.9e-05	35.5
Lipid Metabolism	TG	rs528735287	7	72395981	A	T	-0.148	0.026	6.9e-09	5.7e-05	33.6
Lipid Metabolism	TG	rs190366589	7	72404882	T	C	-0.099	0.017	5.8e-09	3.4e-05	33.9
Lipid Metabolism	TG	rs560006979	7	72725733	C	T	-0.092	0.014	4.5e-11	4.0e-05	43.4
Lipid Metabolism	TG	rs548074364	7	72736308	G	A	-0.106	0.016	1.2e-10	4.9e-05	41.5
Lipid Metabolism	TG	rs182956504	7	72740075	G	T	-0.107	0.012	1.7e-20	7.0e-05	86.1
Lipid Metabolism	TG	rs531271095	7	72764800	A	G	0.097	0.016	2.7e-09	3.1e-05	35.4
Lipid Metabolism	TG	rs117103006	7	72794921	A	G	0.035	0.006	1.0e-09	3.0e-05	37.3
Lipid Metabolism	TG	rs542125752	7	72826667	C	T	-0.208	0.031	3.4e-11	5.1e-05	43.9
Lipid Metabolism	TG	rs538489936	7	72839396	G	T	-0.159	0.024	3.5e-11	6.4e-05	43.9
Lipid Metabolism	TG	rs42122	7	72841823	A	G	-0.074	0.002	1.0e-200	1.3e-03	1571.0
Lipid Metabolism	TG	rs533842724	7	72866802	T	C	-0.162	0.024	1.0e-11	5.6e-05	46.3
Lipid Metabolism	TG	rs73134935	7	72887285	A	T	-0.039	0.003	1.9e-46	1.7e-04	204.8
Lipid Metabolism	TG	rs545698747	7	72898670	C	T	-0.165	0.029	9.5e-09	4.9e-05	32.9
Lipid Metabolism	TG	rs535140552	7	72899939	A	C	-0.245	0.031	4.1e-15	8.2e-05	61.6
Lipid Metabolism	TG	rs188632825	7	72950554	T	C	-0.125	0.022	1.6e-08	3.8e-05	31.9
Lipid Metabolism	TG	rs541412327	7	73045213	T	C	0.304	0.038	9.9e-16	1.6e-04	64.4
Lipid Metabolism	TG	rs7807870	7	73067723	T	C	0.023	0.001	1.3e-53	2.0e-04	237.5
Lipid Metabolism	TG	rs190346330	7	73086782	A	G	-0.151	0.024	3.2e-10	4.2e-05	39.6
Lipid Metabolism	TG	rs34200032	7	73663898	T	A	-0.020	0.002	1.1e-17	6.0e-05	73.3
Lipid Metabolism	TG	rs201452362	7	74014141	T	C	-0.143	0.026	4.4e-08	3.4e-05	30.0
Lipid Metabolism	TG	rs139100778	7	75170955	A	G	-0.145	0.025	6.5e-09	4.9e-05	33.7
Lipid Metabolism	TG	rs1057868	7	75615006	T	C	0.012	0.002	3.6e-15	4.9e-05	61.9
Lipid Metabolism	TG	rs5745687	7	81359051	T	C	0.020	0.003	8.4e-13	4.1e-05	51.2
Lipid Metabolism	TG	rs2906184	7	97939987	A	T	-0.011	0.002	9.0e-09	2.7e-05	33.0
Lipid Metabolism	TG	rs66905828	7	104576670	C	A	-0.009	0.002	5.4e-10	3.1e-05	38.5
Lipid Metabolism	TG	rs3801964	7	106836242	C	T	-0.011	0.002	1.9e-11	3.6e-05	45.1
Lipid Metabolism	TG	rs4727695	7	107614003	G	A	-0.016	0.002	3.0e-11	3.5e-05	44.2
Lipid Metabolism	TG	rs7800249	7	116525501	T	C	-0.010	0.001	1.2e-12	4.0e-05	50.4
Lipid Metabolism	TG	rs6977665	7	116976830	G	A	0.012	0.001	5.5e-17	5.6e-05	70.2
Lipid Metabolism	TG	rs11765411	7	128448311	A	G	0.026	0.005	3.0e-08	2.5e-05	30.7
Lipid Metabolism	TG	rs34282904	7	130434529	A	G	-0.025	0.001	4.6e-74	2.6e-04	331.4
Lipid Metabolism	TG	rs11765347	7	139120072	A	C	-0.010	0.002	1.5e-08	2.6e-05	32.1
Lipid Metabolism	TG	rs855918	7	149196533	A	G	0.009	0.001	6.5e-10	3.1e-05	38.2
Lipid Metabolism	TG	rs10254829	7	150213151	A	C	-0.016	0.002	1.9e-23	8.0e-05	99.5
Lipid Metabolism	TG	rs6977416	7	150542711	A	G	0.015	0.001	2.6e-23	7.9e-05	98.9
Lipid Metabolism	TG	rs3802124	7	156970609	A	G	-0.008	0.001	4.2e-08	2.4e-05	30.1
Lipid Metabolism	TG	rs9657371	8	4782658	A	C	-0.009	0.001	1.3e-10	3.3e-05	41.4
Lipid Metabolism	TG	rs2980766	8	8318095	C	T	-0.016	0.001	6.7e-29	9.9e-05	124.5
Lipid Metabolism	TG	rs35922567	8	9218929	G	T	0.009	0.002	3.2e-08	2.4e-05	30.6
Lipid Metabolism	TG	rs10100760	8	9370559	C	T	-0.015	0.001	1.7e-24	8.4e-05	104.3
Lipid Metabolism	TG	rs57819090	8	10469340	A	G	-0.013	0.002	9.9e-10	3.0e-05	37.4
Lipid Metabolism	TG	rs183513149	8	10557504	T	C	-0.027	0.005	5.3e-09	2.7e-05	34.1
Lipid Metabolism	TG	rs28588745	8	10647044	T	A	0.028	0.002	5.3e-61	2.2e-04	271.5

Phenotype	Trait	SNP	Chr	Position (GRCh37/hg19)	EA	NEA	BETA	SE	P-value	R2	F
Lipid Metabolism	TG	rs11250154	8	11496686	C	T	-0.013	0.002	3.9e-17	5.7e-05	70.8
Lipid Metabolism	TG	rs1497042	8	11660614	T	C	-0.020	0.001	2.0e-46	1.6e-04	204.7
Lipid Metabolism	TG	rs13264888	8	12639212	A	T	0.008	0.001	4.6e-08	2.4e-05	29.9
Lipid Metabolism	TG	rs13250575	8	13508920	C	G	-0.010	0.001	3.2e-12	3.9e-05	48.6
Lipid Metabolism	TG	rs961220	8	16212923	C	A	0.009	0.002	4.4e-08	2.4e-05	30.0
Lipid Metabolism	TG	rs381826	8	17619304	G	A	0.009	0.001	2.5e-10	3.2e-05	40.0
Lipid Metabolism	TG	rs7844866	8	18196091	G	A	0.025	0.003	1.3e-18	6.2e-05	77.5
Lipid Metabolism	TG	rs4921913	8	18272377	T	C	-0.034	0.002	1.0e-96	3.5e-04	435.5
Lipid Metabolism	TG	rs555362190	8	19532212	A	G	0.188	0.016	2.5e-31	1.2e-04	135.5
Lipid Metabolism	TG	rs36082703	8	19597124	G	A	0.122	0.016	6.8e-14	4.7e-05	56.1
Lipid Metabolism	TG	rs111251122	8	19623795	G	A	0.143	0.007	2.7e-83	3.0e-04	373.9
Lipid Metabolism	TG	rs558679944	8	19634363	G	A	-0.149	0.012	8.9e-35	1.4e-04	151.3
Lipid Metabolism	TG	rs144473554	8	19639879	A	T	-0.091	0.013	1.3e-11	5.4e-05	45.8
Lipid Metabolism	TG	rs549365374	8	19642533	T	C	-0.221	0.026	7.7e-17	7.6e-05	69.5
Lipid Metabolism	TG	rs140014394	8	19673994	T	C	-0.134	0.016	8.5e-18	6.2e-05	73.8
Lipid Metabolism	TG	rs539118782	8	19676524	A	G	-0.168	0.025	1.8e-11	4.2e-05	45.2
Lipid Metabolism	TG	rs190341366	8	19705250	A	G	-0.156	0.025	5.0e-10	3.8e-05	38.7
Lipid Metabolism	TG	rs145964918	8	19717989	C	T	0.225	0.030	5.7e-14	1.4e-04	56.5
Lipid Metabolism	TG	rs117860853	8	19722204	A	G	0.145	0.007	1.1e-106	3.9e-04	481.4
Lipid Metabolism	TG	rs545516154	8	19729354	C	T	0.268	0.034	3.6e-15	1.6e-04	61.9
Lipid Metabolism	TG	rs73607783	8	19742204	A	T	-0.068	0.002	1.0e-200	1.2e-03	1550.0
Lipid Metabolism	TG	rs578043912	8	19745762	C	T	-0.220	0.033	3.9e-11	5.8e-05	43.7
Lipid Metabolism	TG	rs182184672	8	19776058	A	G	0.072	0.012	8.0e-09	2.7e-05	33.3
Lipid Metabolism	TG	rs536076610	8	19807477	C	T	0.378	0.045	3.8e-17	1.7e-04	70.9
Lipid Metabolism	TG	rs78326602	8	19809220	A	G	0.076	0.010	2.6e-13	4.4e-05	53.5
Lipid Metabolism	TG	rs144578061	8	19813878	C	A	-0.072	0.008	2.6e-19	6.5e-05	80.7
Lipid Metabolism	TG	rs554540115	8	19818084	C	T	0.228	0.039	4.6e-09	5.2e-05	34.4
Lipid Metabolism	TG	rs548617076	8	19828740	C	T	0.134	0.015	2.1e-18	6.6e-05	76.6
Lipid Metabolism	TG	rs1372343	8	19871320	T	C	-0.063	0.001	1.0e-200	1.7e-03	2083.7
Lipid Metabolism	TG	rs551447383	8	19880070	C	T	-0.200	0.022	1.9e-19	2.0e-04	81.3
Lipid Metabolism	TG	rs186879193	8	19881560	C	A	0.204	0.036	2.1e-08	8.1e-05	31.4
Lipid Metabolism	TG	rs189848936	8	19883934	C	A	0.066	0.009	7.3e-13	4.2e-05	51.5
Lipid Metabolism	TG	rs530229037	8	19896484	C	T	0.101	0.013	5.5e-15	5.0e-05	61.1
Lipid Metabolism	TG	rs577319938	8	19914917	A	T	0.122	0.017	9.6e-13	6.4e-05	50.9
Lipid Metabolism	TG	rs117416256	8	19935660	T	C	0.048	0.008	8.8e-09	2.7e-05	33.1
Lipid Metabolism	TG	rs7001307	8	19938946	C	T	-0.084	0.002	1.0e-200	1.7e-03	1961.3
Lipid Metabolism	TG	rs552492876	8	19939808	T	A	0.130	0.022	1.4e-09	3.5e-05	36.6
Lipid Metabolism	TG	rs77675968	8	19959983	C	T	0.041	0.004	1.4e-25	8.8e-05	109.4
Lipid Metabolism	TG	rs553001378	8	19961375	C	T	-0.145	0.024	2.1e-09	3.9e-05	35.9
Lipid Metabolism	TG	rs181703090	8	19970086	T	C	0.062	0.007	7.2e-19	6.3e-05	78.7
Lipid Metabolism	TG	rs192981862	8	19977354	T	C	-0.185	0.027	3.4e-12	6.3e-05	48.5
Lipid Metabolism	TG	rs558905601	8	20013731	C	T	0.357	0.056	1.6e-10	1.0e-04	40.9
Lipid Metabolism	TG	rs182619883	8	20025108	A	G	-0.094	0.010	7.3e-22	7.5e-05	92.3
Lipid Metabolism	TG	rs375092514	8	20061636	T	C	-0.088	0.011	9.2e-17	5.6e-05	69.1
Lipid Metabolism	TG	rs181843961	8	20116779	T	C	-0.165	0.021	9.5e-15	6.4e-05	60.0
Lipid Metabolism	TG	rs876435	8	22873533	A	G	0.008	0.001	2.0e-09	2.9e-05	35.9
Lipid Metabolism	TG	rs11781886	8	23540417	T	C	-0.011	0.002	6.7e-12	3.8e-05	47.1
Lipid Metabolism	TG	rs11992444	8	25464690	T	G	-0.012	0.002	1.1e-13	5.1e-05	55.3
Lipid Metabolism	TG	rs7003404	8	25871350	T	C	0.010	0.002	1.6e-09	2.9e-05	36.4
Lipid Metabolism	TG	rs13264882	8	26224240	T	G	0.010	0.002	1.0e-10	3.3e-05	41.7
Lipid Metabolism	TG	rs10095380	8	36853217	C	G	0.014	0.002	9.1e-15	4.8e-05	60.1
Lipid Metabolism	TG	rs12681487	8	37384868	G	C	-0.020	0.003	1.1e-08	2.6e-05	32.6
Lipid Metabolism	TG	rs4647906	8	38325411	A	G	0.013	0.001	4.4e-19	6.4e-05	79.7
Lipid Metabolism	TG	rs72648770	8	55454564	G	C	0.010	0.002	2.1e-08	2.5e-05	31.4
Lipid Metabolism	TG	rs2081687	8	59388565	C	T	-0.023	0.001	1.8e-58	2.1e-04	259.9
Lipid Metabolism	TG	rs113211945	8	59558531	C	T	-0.025	0.003	1.5e-14	4.7e-05	59.1

Phenotype	Trait	SNP	Chr	Position (GRCh37/hg19)	EA	NEA	BETA	SE	P-value	R2	F
Lipid Metabolism	TG	rs6992869	8	61395832	C	T	0.008	0.001	4.5e-09	2.8e-05	34.4
Lipid Metabolism	TG	rs28542151	8	64658276	G	T	0.010	0.002	1.8e-09	2.9e-05	36.2
Lipid Metabolism	TG	rs62530460	8	71114795	A	G	-0.009	0.002	2.6e-08	2.5e-05	31.0
Lipid Metabolism	TG	rs13269725	8	72459889	G	A	0.028	0.003	6.4e-26	8.9e-05	110.8
Lipid Metabolism	TG	rs16909233	8	82401680	A	G	-0.012	0.002	9.4e-11	3.4e-05	41.9
Lipid Metabolism	TG	rs768878	8	103801557	T	C	0.010	0.002	7.4e-09	2.7e-05	33.4
Lipid Metabolism	TG	rs7830852	8	106415088	G	A	0.012	0.002	1.6e-12	4.0e-05	50.0
Lipid Metabolism	TG	rs2737246	8	116659578	C	G	-0.012	0.002	3.4e-16	5.3e-05	66.5
Lipid Metabolism	TG	rs10100863	8	117128746	A	G	0.010	0.001	1.7e-11	3.6e-05	45.3
Lipid Metabolism	TG	rs9650069	8	118204020	T	C	-0.009	0.001	5.8e-10	3.1e-05	38.4
Lipid Metabolism	TG	rs140376255	8	126268729	T	C	0.052	0.009	1.7e-09	3.0e-05	36.3
Lipid Metabolism	TG	rs17319046	8	126350794	G	T	-0.017	0.003	1.0e-09	3.0e-05	37.3
Lipid Metabolism	TG	rs2385110	8	126439329	C	A	0.019	0.001	7.0e-38	1.3e-04	165.5
Lipid Metabolism	TG	rs6999569	8	126475770	G	A	-0.081	0.001	1.0e-200	2.7e-03	3390.1
Lipid Metabolism	TG	rs67346026	8	126489818	G	A	0.045	0.005	4.0e-21	1.4e-04	89.0
Lipid Metabolism	TG	rs76599771	8	126513718	A	G	0.045	0.005	2.0e-17	5.8e-05	72.2
Lipid Metabolism	TG	rs78309295	8	126528722	G	A	0.066	0.005	3.2e-40	1.4e-04	176.2
Lipid Metabolism	TG	rs189481193	8	126531375	C	T	-0.099	0.015	2.2e-11	5.4e-05	44.8
Lipid Metabolism	TG	rs13255329	8	126608267	G	T	0.020	0.002	4.5e-27	9.3e-05	116.1
Lipid Metabolism	TG	rs139446067	8	126683833	C	A	0.027	0.004	9.3e-11	3.4e-05	42.0
Lipid Metabolism	TG	rs1372993	8	129565662	A	G	-0.015	0.002	9.8e-13	4.1e-05	50.9
Lipid Metabolism	TG	rs4909360	8	135758340	G	A	0.008	0.001	1.2e-08	2.6e-05	32.5
Lipid Metabolism	TG	rs56925758	8	144299352	A	G	-0.014	0.002	1.1e-15	5.1e-05	64.1
Lipid Metabolism	TG	rs755097	8	144377141	C	T	0.008	0.001	2.6e-09	2.9e-05	35.4
Lipid Metabolism	TG	rs7856817	9	1054587	A	G	0.014	0.002	2.6e-15	5.0e-05	62.6
Lipid Metabolism	TG	rs35043989	9	2633295	A	C	-0.009	0.002	1.1e-09	3.0e-05	37.1
Lipid Metabolism	TG	rs1658970	9	6664853	A	C	0.014	0.002	1.1e-12	4.0e-05	50.6
Lipid Metabolism	TG	rs1590404	9	13697152	C	A	-0.011	0.001	3.0e-14	4.6e-05	57.7
Lipid Metabolism	TG	rs686030	9	15304782	A	C	0.017	0.002	1.3e-17	5.8e-05	72.9
Lipid Metabolism	TG	rs4644350	9	16882916	G	C	-0.015	0.001	5.3e-27	9.3e-05	115.8
Lipid Metabolism	TG	rs10757445	9	23669278	C	G	0.009	0.001	2.0e-09	3.0e-05	35.9
Lipid Metabolism	TG	rs2383766	9	28412500	T	G	0.010	0.001	7.5e-12	3.7e-05	46.9
Lipid Metabolism	TG	rs2889318	9	32565895	A	G	-0.010	0.002	4.1e-09	2.8e-05	34.6
Lipid Metabolism	TG	rs10971977	9	34180023	T	A	-0.011	0.001	8.5e-15	4.8e-05	60.2
Lipid Metabolism	TG	rs7852201	9	35117717	A	T	-0.011	0.002	1.1e-10	3.3e-05	41.7
Lipid Metabolism	TG	rs1831757	9	86509330	C	T	-0.011	0.002	1.7e-12	4.1e-05	49.9
Lipid Metabolism	TG	rs7869923	9	92202486	G	A	0.012	0.001	1.2e-14	4.8e-05	59.5
Lipid Metabolism	TG	rs7855203	9	95557334	T	C	0.012	0.002	1.6e-10	3.3e-05	41.0
Lipid Metabolism	TG	rs10821317	9	96975668	T	C	0.009	0.001	3.5e-10	3.1e-05	39.4
Lipid Metabolism	TG	rs6477489	9	99239841	C	A	-0.009	0.002	3.5e-08	2.4e-05	30.4
Lipid Metabolism	TG	rs62565259	9	102162570	T	C	-0.012	0.002	4.5e-10	3.1e-05	38.9
Lipid Metabolism	TG	rs2777798	9	107557865	G	A	0.013	0.002	7.9e-09	2.7e-05	33.3
Lipid Metabolism	TG	rs2740488	9	107661742	C	A	-0.018	0.002	2.2e-31	1.1e-04	135.8
Lipid Metabolism	TG	rs111936426	9	107724707	A	G	0.018	0.002	3.0e-13	4.3e-05	53.2
Lipid Metabolism	TG	rs11789974	9	110488875	A	C	-0.029	0.004	7.2e-14	4.5e-05	56.0
Lipid Metabolism	TG	rs6477694	9	111932342	T	C	-0.008	0.001	1.3e-08	2.6e-05	32.3
Lipid Metabolism	TG	rs77824033	9	112241136	C	T	-0.031	0.004	1.4e-16	5.5e-05	68.3
Lipid Metabolism	TG	rs10982156	9	117088064	A	T	-0.019	0.003	2.9e-11	3.5e-05	44.2
Lipid Metabolism	TG	rs4837374	9	119154616	G	C	0.009	0.002	4.8e-08	2.4e-05	29.8
Lipid Metabolism	TG	rs7849566	9	123460769	C	A	0.011	0.002	4.7e-14	4.5e-05	56.8
Lipid Metabolism	TG	rs7863124	9	125757627	G	A	-0.012	0.002	7.2e-09	2.7e-05	33.5
Lipid Metabolism	TG	rs10760297	9	126458789	G	A	0.011	0.002	2.5e-10	3.2e-05	40.1
Lipid Metabolism	TG	rs2519093	9	136141870	T	C	-0.010	0.002	2.4e-09	2.8e-05	35.7
Lipid Metabolism	TG	rs3780190	9	139099073	G	A	-0.009	0.001	9.3e-10	3.0e-05	37.5
Lipid Metabolism	TG	rs13295248	9	139386640	C	T	0.009	0.002	3.3e-09	2.8e-05	35.0
Lipid Metabolism	TG	rs142122182	10	5263341	G	A	-0.021	0.002	1.2e-28	1.0e-04	123.3

Phenotype	Trait	SNP	Chr	Position (GRCh37/hg19)	EA	NEA	BETA	SE	P-value	R2	F
Lipid Metabolism	TG	rs10795465	10	17260525	T	C	0.008	0.001	1.7e-08	2.6e-05	31.8
Lipid Metabolism	TG	rs148628052	10	31019231	C	T	-0.030	0.005	2.1e-09	2.9e-05	35.9
Lipid Metabolism	TG	rs10763928	10	33632954	T	C	0.018	0.002	2.1e-13	4.3e-05	54.0
Lipid Metabolism	TG	rs7477945	10	33962855	G	A	0.010	0.002	5.7e-10	3.1e-05	38.4
Lipid Metabolism	TG	rs1413611	10	36466891	C	T	0.009	0.001	5.5e-10	3.1e-05	38.5
Lipid Metabolism	TG	rs3011802	10	52327054	T	C	-0.008	0.001	1.9e-08	2.5e-05	31.6
Lipid Metabolism	TG	rs150770378	10	52616492	A	G	0.084	0.008	2.0e-25	9.7e-05	108.5
Lipid Metabolism	TG	rs12356193	10	61413353	G	A	-0.017	0.002	1.1e-20	6.9e-05	87.1
Lipid Metabolism	TG	rs17287962	10	63770969	G	A	0.025	0.004	1.3e-09	3.0e-05	36.9
Lipid Metabolism	TG	rs35706909	10	63922233	C	T	-0.018	0.002	9.8e-15	4.8e-05	59.9
Lipid Metabolism	TG	rs192354769	10	65019597	C	T	0.314	0.053	3.9e-09	5.8e-05	34.7
Lipid Metabolism	TG	rs7912893	10	65162000	A	T	-0.029	0.001	2.6e-97	3.5e-04	438.3
Lipid Metabolism	TG	rs7911148	10	70334293	G	A	-0.008	0.001	2.1e-08	2.5e-05	31.4
Lipid Metabolism	TG	rs7898735	10	74712546	C	T	0.024	0.002	4.3e-22	7.5e-05	93.4
Lipid Metabolism	TG	rs71473777	10	77217080	G	A	0.014	0.002	2.8e-11	3.5e-05	44.3
Lipid Metabolism	TG	rs12414178	10	81095519	T	C	0.009	0.002	4.4e-09	2.8e-05	34.4
Lipid Metabolism	TG	rs811036	10	89777068	G	A	-0.015	0.003	4.3e-08	2.4e-05	30.0
Lipid Metabolism	TG	rs10788578	10	89791195	C	T	0.010	0.002	1.5e-09	3.0e-05	36.6
Lipid Metabolism	TG	rs11186798	10	93773792	A	G	0.027	0.003	8.9e-21	7.0e-05	87.4
Lipid Metabolism	TG	rs11187258	10	94804778	T	C	-0.016	0.002	8.8e-11	3.8e-05	42.1
Lipid Metabolism	TG	rs913423	10	94845036	A	G	-0.024	0.001	9.3e-69	2.5e-04	307.1
Lipid Metabolism	TG	rs11187498	10	95290777	C	T	0.014	0.002	2.3e-13	4.3e-05	53.8
Lipid Metabolism	TG	rs10786156	10	96014622	G	C	-0.010	0.001	4.3e-13	4.2e-05	52.5
Lipid Metabolism	TG	rs10883026	10	99793865	T	C	-0.008	0.001	3.0e-08	2.5e-05	30.7
Lipid Metabolism	TG	rs1408579	10	101912194	T	C	0.010	0.001	3.0e-13	4.2e-05	53.2
Lipid Metabolism	TG	rs75398587	10	103946480	G	C	-0.027	0.003	7.3e-21	7.0e-05	87.8
Lipid Metabolism	TG	rs2803619	10	113934384	C	G	0.018	0.002	2.0e-32	1.1e-04	140.5
Lipid Metabolism	TG	rs4573621	10	113987548	G	A	0.017	0.002	3.0e-22	7.5e-05	94.1
Lipid Metabolism	TG	rs4918784	10	114643471	G	A	0.009	0.002	3.9e-08	2.4e-05	30.2
Lipid Metabolism	TG	rs740746	10	115792787	A	G	-0.011	0.002	1.1e-11	3.7e-05	46.1
Lipid Metabolism	TG	rs12772930	10	120493295	A	G	0.010	0.001	6.6e-13	4.2e-05	51.6
Lipid Metabolism	TG	rs1873449	10	122931652	T	G	-0.037	0.005	6.5e-15	4.9e-05	60.7
Lipid Metabolism	TG	rs1133400	10	134459388	G	A	0.013	0.002	2.0e-15	5.1e-05	63.1
Lipid Metabolism	TG	rs72868350	10	135308877	C	T	0.033	0.005	6.9e-12	3.8e-05	47.1
Lipid Metabolism	TG	rs117739035	11	408174	T	G	0.025	0.004	1.1e-11	3.8e-05	46.1
Lipid Metabolism	TG	rs12797496	11	583767	T	C	-0.010	0.002	6.2e-09	2.7e-05	33.8
Lipid Metabolism	TG	rs10840453	11	10664901	T	C	-0.014	0.002	1.0e-19	6.6e-05	82.6
Lipid Metabolism	TG	rs10765918	11	12071855	G	A	0.009	0.002	3.4e-08	2.5e-05	30.5
Lipid Metabolism	TG	rs10832027	11	13357183	A	G	0.017	0.001	5.6e-33	1.1e-04	143.1
Lipid Metabolism	TG	rs146920633	11	14371176	A	G	-0.067	0.009	6.9e-13	4.2e-05	51.6
Lipid Metabolism	TG	rs192849769	11	14451992	C	T	-0.165	0.019	3.4e-18	7.1e-05	75.7
Lipid Metabolism	TG	rs79634051	11	14561945	C	G	-0.042	0.005	1.5e-20	6.9e-05	86.3
Lipid Metabolism	TG	rs211108	11	18058662	T	C	-0.008	0.001	2.7e-09	2.8e-05	35.4
Lipid Metabolism	TG	rs72872737	11	26247314	G	A	0.021	0.003	1.2e-13	4.4e-05	55.0
Lipid Metabolism	TG	rs7119628	11	27302716	A	G	0.008	0.001	2.6e-09	2.8e-05	35.5
Lipid Metabolism	TG	rs7127507	11	27714884	C	T	0.014	0.001	1.3e-22	7.7e-05	95.8
Lipid Metabolism	TG	rs12576396	11	28555952	T	C	-0.010	0.002	1.3e-09	3.0e-05	36.8
Lipid Metabolism	TG	rs75160368	11	30402714	A	G	-0.017	0.002	3.1e-12	3.9e-05	48.6
Lipid Metabolism	TG	rs62621409	11	36458997	G	A	0.019	0.003	4.6e-09	2.7e-05	34.3
Lipid Metabolism	TG	rs2625387	11	43613426	A	G	-0.008	0.001	1.9e-08	2.5e-05	31.6
Lipid Metabolism	TG	rs149361023	11	45348323	C	T	0.024	0.004	1.0e-08	2.6e-05	32.8
Lipid Metabolism	TG	rs117847116	11	46048240	C	T	-0.027	0.004	3.1e-14	4.6e-05	57.7
Lipid Metabolism	TG	rs75817747	11	46231010	T	C	-0.024	0.004	5.0e-09	2.7e-05	34.2
Lipid Metabolism	TG	rs112990625	11	46327806	T	C	-0.053	0.008	1.2e-10	3.3e-05	41.4
Lipid Metabolism	TG	rs187725533	11	46332586	T	A	-0.107	0.011	1.3e-21	1.0e-04	91.2
Lipid Metabolism	TG	rs2279239	11	47281780	C	T	-0.023	0.001	1.7e-53	1.9e-04	237.1

Phenotype	Trait	SNP	Chr	Position (GRCh37/hg19)	EA	NEA	BETA	SE	P-value	R2	F
Lipid Metabolism	TG	rs1228023	11	47951771	G	C	0.016	0.002	2.7e-15	5.0e-05	62.5
Lipid Metabolism	TG	rs7943570	11	48085968	T	C	0.021	0.002	2.7e-17	5.7e-05	71.6
Lipid Metabolism	TG	rs3974807	11	49161967	T	C	0.015	0.002	1.7e-16	5.4e-05	68.0
Lipid Metabolism	TG	rs113774743	11	50301750	A	G	-0.023	0.004	3.5e-08	2.9e-05	30.4
Lipid Metabolism	TG	rs118174209	11	50344013	A	T	0.019	0.003	7.9e-13	4.1e-05	51.3
Lipid Metabolism	TG	rs117334884	11	51438835	C	T	0.022	0.004	1.4e-08	3.2e-05	32.2
Lipid Metabolism	TG	rs117350740	11	55108959	T	G	-0.022	0.003	1.6e-10	3.3e-05	41.0
Lipid Metabolism	TG	rs77945636	11	55508858	T	C	0.022	0.003	2.5e-11	3.6e-05	44.5
Lipid Metabolism	TG	rs80284347	11	56436290	A	G	-0.023	0.003	7.4e-12	3.7e-05	46.9
Lipid Metabolism	TG	rs188804108	11	56442809	G	C	-0.086	0.014	7.1e-10	3.9e-05	38.0
Lipid Metabolism	TG	rs536995174	11	56870907	A	T	-0.077	0.013	5.1e-09	4.0e-05	34.2
Lipid Metabolism	TG	rs118188894	11	57142624	C	T	0.015	0.002	4.9e-11	3.4e-05	43.2
Lipid Metabolism	TG	rs7232	11	59940599	A	T	-0.008	0.001	4.6e-09	2.8e-05	34.4
Lipid Metabolism	TG	rs539690492	11	61063662	G	A	0.091	0.014	1.6e-11	5.3e-05	45.5
Lipid Metabolism	TG	rs174562	11	61585144	G	A	0.052	0.001	1.0e-200	1.1e-03	1319.3
Lipid Metabolism	TG	rs79303190	11	61618016	C	T	0.031	0.003	1.3e-23	8.0e-05	100.3
Lipid Metabolism	TG	rs174618	11	61629322	C	T	0.022	0.001	1.1e-54	1.9e-04	242.5
Lipid Metabolism	TG	rs187651672	11	61750516	A	G	0.066	0.011	1.5e-09	3.0e-05	36.5
Lipid Metabolism	TG	rs35927325	11	63882495	T	C	0.041	0.003	8.5e-47	1.6e-04	206.4
Lipid Metabolism	TG	rs10750766	11	65473798	A	C	0.017	0.002	1.3e-29	1.0e-04	127.7
Lipid Metabolism	TG	rs1055615	11	65846922	G	C	0.012	0.002	8.1e-12	3.7e-05	46.7
Lipid Metabolism	TG	rs10896373	11	68614810	T	A	-0.014	0.001	2.0e-24	8.4e-05	104.0
Lipid Metabolism	TG	rs881337	11	78128335	C	G	-0.014	0.002	4.7e-15	4.9e-05	61.4
Lipid Metabolism	TG	rs11020124	11	92690661	C	T	0.009	0.002	1.1e-09	3.0e-05	37.2
Lipid Metabolism	TG	rs12787911	11	95492148	A	T	-0.008	0.001	9.5e-09	2.6e-05	32.9
Lipid Metabolism	TG	rs647080	11	111609174	A	G	0.009	0.001	3.4e-10	3.1e-05	39.4
Lipid Metabolism	TG	rs550941381	11	114913567	G	T	-0.524	0.088	2.1e-09	7.9e-05	35.9
Lipid Metabolism	TG	rs531278380	11	116202870	G	A	-0.310	0.054	1.0e-08	5.0e-05	32.8
Lipid Metabolism	TG	rs569552644	11	116370922	T	C	0.104	0.014	6.7e-13	5.1e-05	51.6
Lipid Metabolism	TG	rs145742932	11	116440470	A	G	0.227	0.014	4.9e-62	2.4e-04	276.2
Lipid Metabolism	TG	rs570487657	11	116448417	G	A	1.225	0.194	2.9e-10	9.7e-05	39.7
Lipid Metabolism	TG	rs542629847	11	116459399	C	A	-0.177	0.012	1.1e-49	2.0e-04	219.5
Lipid Metabolism	TG	rs542930795	11	116469231	G	A	0.202	0.037	4.0e-08	4.3e-05	30.1
Lipid Metabolism	TG	rs528868277	11	116472039	T	C	0.227	0.022	2.3e-24	1.3e-04	103.7
Lipid Metabolism	TG	rs567638473	11	116475210	T	C	0.162	0.028	1.0e-08	3.2e-05	32.8
Lipid Metabolism	TG	rs117993491	11	116508705	A	G	0.426	0.054	1.8e-15	9.8e-05	63.3
Lipid Metabolism	TG	rs513533	11	116519358	G	A	0.113	0.002	1.0e-200	2.0e-03	2535.8
Lipid Metabolism	TG	rs559220724	11	116523568	C	A	0.407	0.009	1.0e-200	1.7e-03	2151.7
Lipid Metabolism	TG	rs2186670	11	116531950	T	C	0.178	0.005	1.0e-200	1.2e-03	1516.1
Lipid Metabolism	TG	rs575671591	11	116543940	A	G	0.227	0.024	2.9e-21	1.1e-04	89.6
Lipid Metabolism	TG	rs530640504	11	116544986	C	T	-0.046	0.006	1.4e-15	5.3e-05	63.8
Lipid Metabolism	TG	rs149473698	11	116550869	T	C	0.226	0.019	6.3e-34	1.8e-04	147.4
Lipid Metabolism	TG	rs141695016	11	116554296	G	C	1.862	0.298	4.2e-10	6.3e-05	39.0
Lipid Metabolism	TG	rs545133050	11	116559645	T	C	0.238	0.040	2.2e-09	4.9e-05	35.8
Lipid Metabolism	TG	rs192379604	11	116562754	G	A	0.247	0.029	7.7e-18	7.6e-05	74.0
Lipid Metabolism	TG	rs146474639	11	116568024	A	G	-0.385	0.059	5.9e-11	5.6e-05	42.9
Lipid Metabolism	TG	rs71480317	11	116572169	C	T	-0.047	0.005	1.3e-19	6.6e-05	82.1
Lipid Metabolism	TG	rs138742363	11	116613578	A	G	-0.046	0.005	5.4e-24	8.2e-05	102.1
Lipid Metabolism	TG	rs61905108	11	116624601	A	C	0.219	0.003	1.0e-200	3.4e-03	4278.5
Lipid Metabolism	TG	rs186911592	11	116629483	G	T	-0.069	0.010	7.8e-13	4.1e-05	51.3
Lipid Metabolism	TG	rs187117860	11	116631789	A	T	-0.075	0.010	1.2e-13	4.6e-05	55.0
Lipid Metabolism	TG	rs117106280	11	116634837	C	G	-0.039	0.007	6.3e-09	2.7e-05	33.7
Lipid Metabolism	TG	rs547886066	11	116637571	T	G	-0.053	0.007	2.4e-13	4.3e-05	53.6
Lipid Metabolism	TG	rs545950926	11	116641187	G	C	0.123	0.017	2.2e-12	4.3e-05	49.3
Lipid Metabolism	TG	rs138452085	11	116660001	A	G	0.338	0.048	2.0e-12	5.4e-05	49.5
Lipid Metabolism	TG	rs143292359	11	116661001	A	G	0.329	0.034	5.2e-22	8.9e-05	93.0

Phenotype	Trait	SNP	Chr	Position (GRCh37/hg19)	EA	NEA	BETA	SE	P-value	R2	F
Lipid Metabolism	TG	rs191179333	11	116664914	T	C	-0.098	0.017	1.1e-08	3.9e-05	32.7
Lipid Metabolism	TG	rs148134535	11	116674897	G	C	-0.075	0.005	4.4e-53	1.9e-04	235.2
Lipid Metabolism	TG	rs549729213	11	116677825	T	C	0.464	0.083	2.0e-08	4.4e-05	31.5
Lipid Metabolism	TG	rs147448028	11	116693647	A	G	0.241	0.015	1.6e-58	2.2e-04	260.1
Lipid Metabolism	TG	rs138326449	11	116701354	A	G	-1.125	0.018	1.0e-200	3.5e-03	4119.6
Lipid Metabolism	TG	rs12721098	11	116702229	T	C	-0.052	0.008	4.4e-11	3.5e-05	43.4
Lipid Metabolism	TG	rs187537072	11	116731872	C	T	-0.114	0.018	5.1e-10	4.5e-05	38.6
Lipid Metabolism	TG	rs189340738	11	116749656	C	T	0.506	0.057	1.1e-18	8.7e-05	77.9
Lipid Metabolism	TG	rs182483424	11	116753830	G	A	-0.035	0.005	4.4e-12	3.8e-05	47.9
Lipid Metabolism	TG	rs541798108	11	116754415	T	C	1.534	0.252	1.2e-09	5.9e-05	37.0
Lipid Metabolism	TG	rs556278194	11	116757869	G	C	0.280	0.039	8.8e-13	5.5e-05	51.1
Lipid Metabolism	TG	rs17120177	11	116827369	C	T	-0.043	0.003	5.7e-49	1.7e-04	216.3
Lipid Metabolism	TG	rs117046382	11	116830423	T	C	-0.040	0.006	2.5e-12	4.0e-05	49.1
Lipid Metabolism	TG	rs185000149	11	116865588	C	A	-0.095	0.012	4.3e-16	5.4e-05	66.1
Lipid Metabolism	TG	rs181349066	11	116874579	C	T	0.264	0.031	9.5e-18	1.0e-04	73.6
Lipid Metabolism	TG	rs182738832	11	116885682	G	A	0.198	0.012	2.4e-64	2.4e-04	286.9
Lipid Metabolism	TG	rs117239858	11	116915241	T	C	0.124	0.019	5.8e-11	3.8e-05	42.9
Lipid Metabolism	TG	rs181940958	11	116961320	G	A	0.845	0.072	8.9e-32	2.0e-04	137.6
Lipid Metabolism	TG	rs184004596	11	116967284	A	G	0.138	0.015	1.3e-19	8.3e-05	82.1
Lipid Metabolism	TG	rs60639658	11	116968850	A	C	0.308	0.038	4.4e-16	8.2e-05	66.1
Lipid Metabolism	TG	rs566419460	11	116989101	A	G	0.236	0.021	1.3e-29	1.5e-04	127.7
Lipid Metabolism	TG	rs554042065	11	117002795	G	A	0.371	0.064	5.9e-09	5.9e-05	33.9
Lipid Metabolism	TG	rs562810477	11	117004213	C	A	-0.052	0.007	2.3e-15	1.0e-04	62.8
Lipid Metabolism	TG	rs547074253	11	117010980	C	T	0.380	0.055	6.5e-12	6.9e-05	47.2
Lipid Metabolism	TG	rs529684599	11	117017799	G	A	-0.338	0.060	1.4e-08	7.9e-05	32.2
Lipid Metabolism	TG	rs534938579	11	117030494	G	A	0.136	0.011	2.9e-37	2.6e-04	162.7
Lipid Metabolism	TG	rs554381783	11	117034421	C	T	0.305	0.033	9.0e-21	1.2e-04	87.4
Lipid Metabolism	TG	rs528110141	11	117041200	G	T	0.260	0.025	6.0e-26	1.8e-04	111.0
Lipid Metabolism	TG	rs529725602	11	117051546	C	G	0.316	0.040	2.5e-15	1.0e-04	62.6
Lipid Metabolism	TG	rs567689836	11	117056766	A	G	0.291	0.032	3.6e-20	1.0e-04	84.6
Lipid Metabolism	TG	rs4938370	11	117193350	C	A	0.019	0.002	1.9e-33	1.2e-04	145.2
Lipid Metabolism	TG	rs181103771	11	117202680	T	C	-1.400	0.166	3.0e-17	1.8e-04	71.3
Lipid Metabolism	TG	rs138933461	11	117212481	T	C	0.265	0.034	3.7e-15	8.4e-05	61.8
Lipid Metabolism	TG	rs186564865	11	117253270	G	A	0.284	0.028	1.2e-23	1.3e-04	100.4
Lipid Metabolism	TG	rs147554220	11	117352857	A	G	0.185	0.021	1.9e-18	1.1e-04	76.8
Lipid Metabolism	TG	rs528432516	11	117383779	T	G	0.118	0.015	5.9e-15	7.0e-05	60.9
Lipid Metabolism	TG	rs151097502	11	117388115	G	C	-0.222	0.023	1.8e-21	7.9e-05	90.6
Lipid Metabolism	TG	rs535157666	11	117555331	C	G	0.475	0.076	3.5e-10	8.2e-05	39.4
Lipid Metabolism	TG	rs186974307	11	117576049	T	A	0.183	0.034	4.6e-08	4.4e-05	29.9
Lipid Metabolism	TG	rs138698876	11	117696311	A	G	0.564	0.102	3.3e-08	6.7e-05	30.5
Lipid Metabolism	TG	rs117923501	11	117721398	T	C	0.183	0.029	2.4e-10	6.9e-05	40.1
Lipid Metabolism	TG	rs78534587	11	117791632	C	T	0.131	0.022	2.3e-09	5.7e-05	35.7
Lipid Metabolism	TG	rs187303914	11	117846882	C	G	-0.455	0.067	9.2e-12	1.1e-04	46.5
Lipid Metabolism	TG	rs187547940	11	117990110	G	A	-0.295	0.054	4.7e-08	4.6e-05	29.8
Lipid Metabolism	TG	rs148118632	11	118399100	G	A	-0.033	0.005	4.0e-11	3.5e-05	43.6
Lipid Metabolism	TG	rs202215150	11	118881946	T	C	-0.583	0.085	8.3e-12	1.2e-04	46.7
Lipid Metabolism	TG	rs73632737	11	126226775	C	G	0.014	0.003	3.5e-08	2.4e-05	30.4
Lipid Metabolism	TG	rs2007537	12	504797	T	C	-0.010	0.002	6.3e-10	3.1e-05	38.2
Lipid Metabolism	TG	rs564579011	12	4343343	G	A	-0.153	0.020	1.2e-14	6.7e-05	59.6
Lipid Metabolism	TG	rs190243882	12	4347455	C	A	-0.079	0.012	3.0e-11	3.7e-05	44.2
Lipid Metabolism	TG	rs142394917	12	4401472	A	G	-0.110	0.011	7.9e-24	8.2e-05	101.3
Lipid Metabolism	TG	rs142409867	12	4518681	G	A	-0.058	0.011	3.4e-08	2.5e-05	30.4
Lipid Metabolism	TG	rs73047887	12	6736843	C	T	0.011	0.002	6.2e-12	3.8e-05	47.3
Lipid Metabolism	TG	rs12372459	12	6866355	T	C	0.013	0.002	4.1e-09	2.8e-05	34.6
Lipid Metabolism	TG	rs10772493	12	11787922	T	C	0.009	0.001	3.0e-10	3.2e-05	39.6
Lipid Metabolism	TG	rs184257876	12	20413592	T	C	-0.016	0.003	5.4e-09	2.8e-05	34.0

Phenotype	Trait	SNP	Chr	Position (GRCh37/hg19)	EA	NEA	BETA	SE	P-value	R2	F
Lipid Metabolism	TG	rs11045247	12	20592624	A	G	0.021	0.003	4.1e-15	4.9e-05	61.7
Lipid Metabolism	TG	rs4149056	12	21331549	C	T	0.027	0.002	1.3e-47	1.7e-04	210.1
Lipid Metabolism	TG	rs3809195	12	22776605	T	A	-0.015	0.002	2.4e-20	6.8e-05	85.5
Lipid Metabolism	TG	rs17389465	12	25414408	A	T	-0.020	0.003	1.5e-13	4.4e-05	54.6
Lipid Metabolism	TG	rs12809441	12	26302161	G	A	0.008	0.001	4.9e-08	2.4e-05	29.7
Lipid Metabolism	TG	rs7132434	12	26472562	G	A	-0.015	0.002	3.7e-21	7.1e-05	89.1
Lipid Metabolism	TG	rs10128781	12	29520706	C	T	-0.013	0.002	5.6e-17	5.6e-05	70.1
Lipid Metabolism	TG	rs2731006	12	43160825	T	C	-0.010	0.002	4.4e-08	2.4e-05	30.0
Lipid Metabolism	TG	rs11183212	12	46213867	G	A	0.016	0.002	7.8e-20	6.6e-05	83.1
Lipid Metabolism	TG	rs145878042	12	48143315	G	A	0.046	0.006	8.5e-13	4.1e-05	51.2
Lipid Metabolism	TG	rs1126930	12	49399132	C	G	0.028	0.004	1.7e-13	4.4e-05	54.4
Lipid Metabolism	TG	rs10783578	12	53796754	A	G	0.010	0.002	5.4e-11	4.0e-05	43.0
Lipid Metabolism	TG	rs67036743	12	56948786	A	C	-0.011	0.001	3.3e-14	4.6e-05	57.6
Lipid Metabolism	TG	rs79395356	12	57738600	G	T	-0.025	0.002	2.6e-53	1.9e-04	236.2
Lipid Metabolism	TG	rs10783828	12	57861484	G	A	-0.011	0.001	7.7e-15	4.8e-05	60.4
Lipid Metabolism	TG	rs113439801	12	62838230	T	C	-0.012	0.002	3.2e-11	3.5e-05	44.1
Lipid Metabolism	TG	rs7308703	12	63494993	T	C	-0.009	0.001	7.2e-10	3.0e-05	38.0
Lipid Metabolism	TG	rs145775785	12	65902265	T	C	-0.041	0.006	3.0e-12	3.9e-05	48.7
Lipid Metabolism	TG	rs1993669	12	66190153	T	C	0.015	0.002	5.1e-11	3.4e-05	43.1
Lipid Metabolism	TG	rs10784771	12	69621264	G	A	-0.010	0.001	1.6e-11	3.7e-05	45.4
Lipid Metabolism	TG	rs6220	12	102794515	A	G	0.009	0.002	8.3e-09	2.7e-05	33.2
Lipid Metabolism	TG	rs11113118	12	107199142	A	G	0.017	0.002	7.2e-25	8.5e-05	106.0
Lipid Metabolism	TG	rs7300215	12	109089394	A	G	0.010	0.001	1.5e-12	4.0e-05	50.1
Lipid Metabolism	TG	rs56358165	12	109610802	T	C	-0.033	0.005	5.2e-10	3.1e-05	38.6
Lipid Metabolism	TG	rs149793040	12	109661672	G	A	-0.215	0.020	7.7e-26	1.1e-04	110.5
Lipid Metabolism	TG	rs2075260	12	109696838	A	G	0.017	0.002	6.3e-22	7.4e-05	92.6
Lipid Metabolism	TG	rs3858704	12	111705893	G	A	-0.012	0.002	2.5e-15	5.0e-05	62.7
Lipid Metabolism	TG	rs1966342	12	112881342	A	G	-0.017	0.002	6.1e-13	4.1e-05	51.8
Lipid Metabolism	TG	rs61954254	12	122635613	A	G	-0.010	0.002	2.3e-08	2.6e-05	31.2
Lipid Metabolism	TG	rs580063	12	123206340	C	T	-0.017	0.002	2.3e-24	8.4e-05	103.7
Lipid Metabolism	TG	rs4930731	12	124317416	G	A	0.015	0.001	1.9e-25	8.7e-05	108.7
Lipid Metabolism	TG	rs12303671	12	124492610	G	T	-0.026	0.001	3.5e-70	2.5e-04	313.6
Lipid Metabolism	TG	rs61941660	12	125319181	C	T	0.015	0.003	2.9e-09	2.8e-05	35.2
Lipid Metabolism	TG	rs11057878	12	125367223	A	G	0.025	0.004	8.8e-10	3.0e-05	37.6
Lipid Metabolism	TG	rs12812512	12	133134522	A	G	0.009	0.001	4.4e-09	3.1e-05	34.4
Lipid Metabolism	TG	rs4770433	13	23903791	G	A	-0.011	0.001	1.4e-14	4.7e-05	59.3
Lipid Metabolism	TG	rs1340819	13	29145323	C	A	-0.010	0.001	7.5e-12	3.8e-05	46.9
Lipid Metabolism	TG	rs7987358	13	31003264	T	C	-0.012	0.002	6.6e-16	6.1e-05	65.2
Lipid Metabolism	TG	rs78844280	13	50349898	A	G	-0.044	0.005	2.1e-21	7.2e-05	90.2
Lipid Metabolism	TG	rs9562973	13	51019018	T	C	0.015	0.002	3.6e-12	3.9e-05	48.3
Lipid Metabolism	TG	rs2160251	13	51172269	G	A	0.009	0.001	6.9e-11	3.4e-05	42.6
Lipid Metabolism	TG	rs3013318	13	56009364	G	C	0.010	0.002	3.9e-09	2.8e-05	34.7
Lipid Metabolism	TG	rs17263198	13	58319127	A	G	0.009	0.002	1.7e-08	2.6e-05	31.8
Lipid Metabolism	TG	rs6492721	13	95258944	C	T	-0.018	0.001	3.9e-34	1.2e-04	148.4
Lipid Metabolism	TG	rs3825430	13	99129653	T	C	0.008	0.001	1.2e-08	2.6e-05	32.4
Lipid Metabolism	TG	rs4773157	13	110992623	A	C	-0.010	0.002	3.1e-10	3.2e-05	39.6
Lipid Metabolism	TG	rs7140110	13	114544024	C	T	0.027	0.002	1.7e-69	2.6e-04	310.6
Lipid Metabolism	TG	rs9604570	13	114631940	G	A	-0.023	0.002	1.8e-35	1.2e-04	154.5
Lipid Metabolism	TG	rs71413977	14	23733062	C	T	-0.011	0.002	1.2e-09	3.0e-05	37.0
Lipid Metabolism	TG	rs79873985	14	24560159	G	A	-0.037	0.006	2.1e-10	3.3e-05	40.4
Lipid Metabolism	TG	rs7159513	14	29856951	A	G	0.011	0.002	4.8e-09	2.7e-05	34.3
Lipid Metabolism	TG	rs1958466	14	38830251	T	C	-0.009	0.002	1.1e-08	2.6e-05	32.6
Lipid Metabolism	TG	rs62017272	14	40014604	C	T	0.011	0.002	9.0e-11	3.4e-05	42.0
Lipid Metabolism	TG	rs72683923	14	50735947	C	T	-0.037	0.005	7.5e-12	3.8e-05	46.9
Lipid Metabolism	TG	rs140664623	14	52321987	T	G	-0.029	0.005	4.0e-10	3.1e-05	39.1
Lipid Metabolism	TG	rs146182298	14	52531408	T	C	0.023	0.004	1.7e-10	3.3e-05	40.8

Phenotype	Trait	SNP	Chr	Position (GRCh37/hg19)	EA	NEA	BETA	SE	P-value	R2	F
Lipid Metabolism	TG	rs1035663	14	53832870	A	G	-0.008	0.001	2.6e-09	2.8e-05	35.5
Lipid Metabolism	TG	rs3813402	14	55257590	G	A	0.008	0.001	2.1e-08	2.5e-05	31.4
Lipid Metabolism	TG	rs61974517	14	58856652	C	T	-0.009	0.001	6.4e-09	2.7e-05	33.7
Lipid Metabolism	TG	rs12878001	14	64239629	G	T	0.021	0.002	1.4e-28	9.9e-05	123.0
Lipid Metabolism	TG	rs36028293	14	68976059	A	G	0.009	0.002	4.5e-08	2.4e-05	29.9
Lipid Metabolism	TG	rs4899263	14	69285264	A	G	0.008	0.001	9.8e-10	3.0e-05	37.4
Lipid Metabolism	TG	rs11158800	14	69747046	A	G	0.008	0.001	3.5e-08	2.4e-05	30.4
Lipid Metabolism	TG	rs2810071	14	71387648	G	A	0.008	0.001	3.4e-08	2.5e-05	30.5
Lipid Metabolism	TG	rs10136360	14	79894046	A	G	0.010	0.002	9.6e-09	2.6e-05	32.9
Lipid Metabolism	TG	rs8020112	14	89798125	C	T	-0.012	0.002	4.7e-12	3.8e-05	47.8
Lipid Metabolism	TG	rs7142571	14	90035606	C	A	-0.008	0.001	8.4e-09	2.7e-05	33.2
Lipid Metabolism	TG	rs10484041	14	94852691	T	C	-0.011	0.002	6.3e-09	2.7e-05	33.7
Lipid Metabolism	TG	rs61993685	14	100765823	C	T	-0.019	0.003	4.8e-13	4.2e-05	52.3
Lipid Metabolism	TG	rs12890430	14	104293534	A	G	0.011	0.001	1.3e-14	4.8e-05	59.4
Lipid Metabolism	TG	rs28624578	15	31637666	C	T	0.013	0.002	7.1e-13	4.2e-05	51.5
Lipid Metabolism	TG	rs8026348	15	35943377	G	A	-0.011	0.002	1.8e-09	2.9e-05	36.1
Lipid Metabolism	TG	rs34245505	15	40397191	G	C	0.015	0.002	4.1e-17	5.9e-05	70.7
Lipid Metabolism	TG	rs4923869	15	40737490	T	C	-0.011	0.001	9.7e-14	4.6e-05	55.4
Lipid Metabolism	TG	rs4420508	15	40956681	G	C	0.011	0.002	4.0e-08	2.4e-05	30.2
Lipid Metabolism	TG	rs7167078	15	41972392	G	C	-0.011	0.001	8.4e-14	4.5e-05	55.7
Lipid Metabolism	TG	rs184334219	15	42721845	A	G	0.087	0.005	3.9e-68	2.4e-04	304.3
Lipid Metabolism	TG	rs35975365	15	43671545	G	A	-0.011	0.002	1.1e-09	3.0e-05	37.1
Lipid Metabolism	TG	rs139974673	15	44027885	C	T	0.134	0.004	1.0e-200	7.8e-04	979.9
Lipid Metabolism	TG	rs2414109	15	51901015	C	G	-0.009	0.001	6.0e-10	3.1e-05	38.3
Lipid Metabolism	TG	rs72749502	15	57297078	T	A	0.030	0.003	2.9e-27	9.3e-05	117.0
Lipid Metabolism	TG	rs58038553	15	58574324	G	A	-0.020	0.002	2.8e-23	7.9e-05	98.8
Lipid Metabolism	TG	rs7350789	15	58679668	A	G	0.029	0.001	6.3e-94	3.4e-04	422.7
Lipid Metabolism	TG	rs573241250	15	58679688	C	G	0.051	0.008	1.4e-10	3.3e-05	41.1
Lipid Metabolism	TG	rs1077835	15	58723426	G	A	0.045	0.002	2.6e-159	5.8e-04	723.3
Lipid Metabolism	TG	rs78819330	15	58828908	A	G	0.028	0.005	5.4e-09	2.8e-05	34.0
Lipid Metabolism	TG	rs121912502	15	58840586	T	C	0.135	0.022	8.1e-10	3.8e-05	37.7
Lipid Metabolism	TG	rs113298164	15	58855748	T	C	0.145	0.012	2.9e-33	1.3e-04	144.4
Lipid Metabolism	TG	rs2245787	15	61951529	A	G	0.010	0.001	7.0e-13	4.1e-05	51.5
Lipid Metabolism	TG	rs4774478	15	63434763	G	A	-0.013	0.001	2.4e-19	6.5e-05	80.9
Lipid Metabolism	TG	rs62011285	15	63791063	C	T	0.024	0.001	2.0e-60	2.2e-04	268.9
Lipid Metabolism	TG	rs539924034	15	64124734	A	C	0.095	0.017	2.8e-08	3.7e-05	30.8
Lipid Metabolism	TG	rs6494481	15	64849904	T	G	-0.015	0.002	5.2e-12	3.8e-05	47.6
Lipid Metabolism	TG	rs2218181	15	66872325	C	T	0.014	0.001	1.2e-22	7.7e-05	96.0
Lipid Metabolism	TG	rs3850986	15	73091283	T	G	0.011	0.001	1.0e-13	4.5e-05	55.4
Lipid Metabolism	TG	rs7176647	15	99177595	G	C	0.009	0.001	7.2e-11	3.4e-05	42.5
Lipid Metabolism	TG	rs11632987	15	101891362	G	A	-0.011	0.001	3.6e-15	5.0e-05	61.9
Lipid Metabolism	TG	rs8025505	15	102067841	T	C	0.014	0.002	7.7e-18	6.0e-05	74.0
Lipid Metabolism	TG	rs35168378	16	920870	T	C	0.036	0.005	1.3e-14	5.6e-05	59.4
Lipid Metabolism	TG	rs12600110	16	962154	C	T	-0.015	0.001	1.2e-25	8.8e-05	109.6
Lipid Metabolism	TG	rs72766660	16	2254626	A	G	0.021	0.003	2.8e-10	3.2e-05	39.8
Lipid Metabolism	TG	rs1684608	16	4676852	A	C	0.012	0.002	4.6e-12	3.8e-05	47.8
Lipid Metabolism	TG	rs7193637	16	11764920	T	C	0.009	0.001	7.1e-11	3.4e-05	42.5
Lipid Metabolism	TG	rs12928099	16	15150505	A	C	-0.026	0.002	1.4e-64	2.3e-04	288.0
Lipid Metabolism	TG	rs75512281	16	15473683	A	T	0.022	0.004	1.5e-09	3.3e-05	36.5
Lipid Metabolism	TG	rs3814883	16	29994922	T	C	0.012	0.001	7.4e-18	6.0e-05	74.1
Lipid Metabolism	TG	rs1978487	16	31129942	T	C	0.015	0.001	1.4e-25	8.8e-05	109.3
Lipid Metabolism	TG	rs1861649	16	49886366	C	T	-0.013	0.002	1.1e-10	3.3e-05	41.7
Lipid Metabolism	TG	rs62033400	16	53811788	G	A	0.016	0.001	3.8e-29	1.0e-04	125.6
Lipid Metabolism	TG	rs146571693	16	56962029	C	T	-0.063	0.011	2.7e-08	2.5e-05	30.9
Lipid Metabolism	TG	rs247615	16	56984763	G	A	0.013	0.002	5.4e-14	4.5e-05	56.6
Lipid Metabolism	TG	rs247617	16	56990716	A	C	-0.033	0.001	5.7e-112	4.1e-04	505.6

Phenotype	Trait	SNP	Chr	Position (GRCh37/hg19)	EA	NEA	BETA	SE	P-value	R2	F
Lipid Metabolism	TG	rs34213076	16	67564059	T	C	-0.022	0.004	2.1e-08	2.5e-05	31.4
Lipid Metabolism	TG	rs73597575	16	67677001	T	C	-0.017	0.002	8.1e-13	4.1e-05	51.3
Lipid Metabolism	TG	rs56285339	16	69160925	G	A	0.009	0.002	2.6e-08	2.5e-05	31.0
Lipid Metabolism	TG	rs862320	16	69651866	T	C	-0.016	0.001	2.8e-30	1.1e-04	130.7
Lipid Metabolism	TG	rs145973320	16	70983413	C	T	0.019	0.003	1.4e-09	2.9e-05	36.7
Lipid Metabolism	TG	rs12924886	16	72075593	T	A	0.022	0.002	2.9e-36	1.3e-04	158.1
Lipid Metabolism	TG	rs11647069	16	72155237	C	T	0.010	0.001	2.2e-11	3.6e-05	44.8
Lipid Metabolism	TG	rs4788495	16	73196659	T	C	0.008	0.001	1.8e-08	2.5e-05	31.7
Lipid Metabolism	TG	rs11865979	16	79722913	C	T	0.012	0.002	9.2e-15	4.8e-05	60.1
Lipid Metabolism	TG	rs17197883	16	81523013	C	T	-0.011	0.002	4.4e-11	3.5e-05	43.4
Lipid Metabolism	TG	rs2966094	16	81538637	C	A	-0.020	0.001	1.4e-43	1.5e-04	191.7
Lipid Metabolism	TG	rs11641586	16	85270707	T	G	-0.014	0.002	1.7e-15	5.1e-05	63.4
Lipid Metabolism	TG	rs8054124	16	86434553	C	T	0.012	0.002	1.2e-13	4.4e-05	55.0
Lipid Metabolism	TG	rs12926107	16	88004092	G	A	0.009	0.001	1.5e-10	3.3e-05	41.0
Lipid Metabolism	TG	rs1109334	16	89938144	A	G	-0.009	0.001	3.7e-09	2.8e-05	34.8
Lipid Metabolism	TG	rs12948505	17	464962	T	C	0.012	0.002	1.6e-12	4.0e-05	50.0
Lipid Metabolism	TG	rs11078597	17	1618363	C	T	0.017	0.002	8.3e-23	7.8e-05	96.6
Lipid Metabolism	TG	rs7214177	17	2153776	A	G	-0.015	0.002	2.9e-10	3.2e-05	39.7
Lipid Metabolism	TG	rs544142176	17	4431836	A	C	-0.145	0.023	5.9e-10	6.0e-05	38.4
Lipid Metabolism	TG	rs537438344	17	4436098	C	G	-0.130	0.017	6.7e-15	7.7e-05	60.7
Lipid Metabolism	TG	rs140779352	17	4672983	A	C	-0.137	0.009	1.5e-54	2.0e-04	241.9
Lipid Metabolism	TG	rs9890797	17	4673609	C	T	0.013	0.001	7.8e-18	6.0e-05	74.0
Lipid Metabolism	TG	rs149485603	17	5686310	C	T	-0.082	0.011	3.9e-13	4.3e-05	52.7
Lipid Metabolism	TG	rs200489612	17	7106378	A	G	-0.119	0.012	3.8e-22	8.0e-05	93.6
Lipid Metabolism	TG	rs4796407	17	7245371	G	A	-0.010	0.001	1.4e-10	3.5e-05	41.2
Lipid Metabolism	TG	rs11651783	17	7447910	T	C	0.015	0.002	1.8e-19	6.5e-05	81.4
Lipid Metabolism	TG	rs11870307	17	7617787	G	A	-0.010	0.002	9.3e-10	3.0e-05	37.5
Lipid Metabolism	TG	rs1263507	17	17373571	G	A	-0.015	0.002	2.0e-20	6.9e-05	85.8
Lipid Metabolism	TG	rs9905032	17	17457768	A	G	0.029	0.003	3.0e-24	8.3e-05	103.2
Lipid Metabolism	TG	rs704	17	26694861	A	G	-0.011	0.001	5.6e-16	5.3e-05	65.6
Lipid Metabolism	TG	rs141385558	17	27893545	T	C	0.085	0.015	3.0e-08	3.0e-05	30.7
Lipid Metabolism	TG	rs12939390	17	28635688	C	T	-0.011	0.001	2.4e-13	4.3e-05	53.6
Lipid Metabolism	TG	rs62070652	17	29221277	T	C	0.009	0.002	2.0e-08	2.5e-05	31.5
Lipid Metabolism	TG	rs9906189	17	34905408	G	A	0.009	0.001	3.0e-11	3.5e-05	44.1
Lipid Metabolism	TG	rs180705493	17	40359483	A	G	0.086	0.013	1.5e-10	3.5e-05	41.0
Lipid Metabolism	TG	rs117770911	17	40394367	T	C	0.044	0.007	2.4e-09	2.9e-05	35.6
Lipid Metabolism	TG	rs650558	17	40721042	T	C	0.015	0.002	1.1e-21	7.3e-05	91.5
Lipid Metabolism	TG	rs149951966	17	41137136	C	T	-0.011	0.002	9.6e-09	2.8e-05	32.9
Lipid Metabolism	TG	rs550900797	17	41379936	C	A	0.099	0.016	6.5e-10	4.2e-05	38.2
Lipid Metabolism	TG	rs566430451	17	41774038	T	C	0.121	0.021	5.0e-09	4.2e-05	34.2
Lipid Metabolism	TG	rs140859835	17	41821630	G	A	-0.022	0.004	1.3e-09	3.0e-05	36.9
Lipid Metabolism	TG	rs147712113	17	41873380	C	T	0.022	0.004	3.1e-08	2.7e-05	30.7
Lipid Metabolism	TG	rs72836561	17	41926126	T	C	0.150	0.004	1.0e-200	1.1e-03	1403.3
Lipid Metabolism	TG	rs552316287	17	41977548	C	T	0.218	0.017	1.8e-37	1.4e-04	163.7
Lipid Metabolism	TG	rs11657450	17	42002464	T	C	0.020	0.002	7.5e-23	7.7e-05	96.8
Lipid Metabolism	TG	rs572318283	17	42216708	T	C	-0.055	0.010	1.1e-08	2.7e-05	32.7
Lipid Metabolism	TG	rs12150561	17	42246196	C	T	-0.025	0.003	9.3e-14	4.4e-05	55.5
Lipid Metabolism	TG	rs72828813	17	42984085	T	C	0.039	0.006	1.1e-10	3.3e-05	41.7
Lipid Metabolism	TG	rs11653796	17	43218259	G	A	0.010	0.002	5.6e-10	3.1e-05	38.5
Lipid Metabolism	TG	rs8065669	17	45724996	A	G	-0.010	0.001	9.5e-13	4.1e-05	51.0
Lipid Metabolism	TG	rs2051821	17	46143515	C	T	-0.015	0.002	1.5e-19	6.6e-05	81.7
Lipid Metabolism	TG	rs61632152	17	47025654	T	C	-0.008	0.002	3.1e-08	2.5e-05	30.6
Lipid Metabolism	TG	rs28412876	17	47454515	T	G	0.016	0.001	7.9e-30	1.0e-04	128.7
Lipid Metabolism	TG	rs8076632	17	48625928	G	C	-0.009	0.001	1.3e-10	3.3e-05	41.2
Lipid Metabolism	TG	rs71381337	17	49274511	A	G	-0.011	0.002	1.5e-11	3.7e-05	45.5
Lipid Metabolism	TG	rs15944	17	55198248	T	A	0.025	0.004	2.3e-11	3.6e-05	44.7

Phenotype	Trait	SNP	Chr	Position (GRCh37/hg19)	EA	NEA	BETA	SE	P-value	R2	F
Lipid Metabolism	TG	rs1292037	17	57918908	C	T	0.016	0.002	2.4e-22	7.5e-05	94.5
Lipid Metabolism	TG	rs1801690	17	64208285	G	C	-0.023	0.003	3.9e-15	4.9e-05	61.7
Lipid Metabolism	TG	rs8178824	17	64224775	T	C	-0.057	0.004	3.9e-40	1.4e-04	175.8
Lipid Metabolism	TG	rs4790985	17	65272255	A	G	0.010	0.002	9.3e-11	3.4e-05	42.0
Lipid Metabolism	TG	rs11656743	17	65980289	A	G	-0.024	0.002	1.9e-47	1.7e-04	209.3
Lipid Metabolism	TG	rs5821683	17	66845642	C	A	0.010	0.002	6.1e-09	2.7e-05	33.8
Lipid Metabolism	TG	rs77542162	17	67081278	G	A	-0.049	0.005	3.2e-22	7.5e-05	94.0
Lipid Metabolism	TG	rs717118	17	68464817	A	T	-0.010	0.001	1.1e-13	4.5e-05	55.2
Lipid Metabolism	TG	rs11653006	17	70296543	G	A	0.008	0.001	4.2e-09	2.8e-05	34.5
Lipid Metabolism	TG	rs2279162	17	73259083	T	C	0.010	0.002	4.8e-11	3.5e-05	43.2
Lipid Metabolism	TG	rs7370	17	73894804	A	G	-0.009	0.002	4.1e-08	2.4e-05	30.1
Lipid Metabolism	TG	rs193220	17	74268619	T	C	-0.012	0.001	2.4e-15	5.0e-05	62.7
Lipid Metabolism	TG	rs16971107	17	76394042	A	G	-0.020	0.001	2.8e-44	1.6e-04	194.8
Lipid Metabolism	TG	rs11650000	17	76828154	G	A	-0.009	0.001	3.9e-10	3.1e-05	39.2
Lipid Metabolism	TG	rs62078746	17	80053590	A	G	-0.011	0.001	4.1e-14	4.6e-05	57.1
Lipid Metabolism	TG	rs8083730	18	293519	A	G	-0.019	0.002	6.9e-14	4.5e-05	56.1
Lipid Metabolism	TG	rs3810068	18	2846499	C	T	-0.009	0.002	1.6e-08	2.7e-05	31.9
Lipid Metabolism	TG	rs62086665	18	13770318	A	C	-0.010	0.002	2.0e-11	3.6e-05	45.0
Lipid Metabolism	TG	rs55827748	18	19656596	C	A	-0.013	0.002	3.1e-09	2.8e-05	35.1
Lipid Metabolism	TG	rs35321365	18	19905141	T	C	-0.011	0.001	1.8e-16	5.5e-05	67.8
Lipid Metabolism	TG	rs1623060	18	21143183	T	C	-0.015	0.001	3.0e-26	9.0e-05	112.4
Lipid Metabolism	TG	rs190691924	18	29501708	T	C	0.179	0.024	1.6e-13	7.5e-05	54.4
Lipid Metabolism	TG	rs150261265	18	29772887	C	T	0.191	0.024	1.4e-15	7.7e-05	63.8
Lipid Metabolism	TG	rs567424502	18	29991345	T	C	0.196	0.028	2.7e-12	8.4e-05	48.9
Lipid Metabolism	TG	rs4890501	18	42599987	G	A	0.010	0.001	2.5e-11	3.6e-05	44.5
Lipid Metabolism	TG	rs149615216	18	47106028	T	C	0.036	0.007	4.8e-08	2.4e-05	29.8
Lipid Metabolism	TG	rs143393480	18	56089858	T	G	0.056	0.007	1.3e-14	4.8e-05	59.4
Lipid Metabolism	TG	rs12954782	18	57864092	G	C	0.013	0.002	1.6e-17	5.8e-05	72.5
Lipid Metabolism	TG	rs77842638	18	58061423	A	G	-0.033	0.005	1.0e-12	4.1e-05	50.8
Lipid Metabolism	TG	rs74625348	18	60846430	C	G	-0.013	0.002	1.4e-14	4.8e-05	59.2
Lipid Metabolism	TG	rs8110974	19	798643	A	G	0.013	0.002	2.5e-11	3.7e-05	44.5
Lipid Metabolism	TG	rs140448673	19	4029056	A	G	-0.037	0.006	1.4e-09	3.1e-05	36.6
Lipid Metabolism	TG	rs7260465	19	4139440	T	C	-0.017	0.002	2.9e-25	8.7e-05	107.9
Lipid Metabolism	TG	rs142674900	19	4157371	C	G	-0.072	0.009	3.5e-16	6.2e-05	66.5
Lipid Metabolism	TG	rs35502362	19	4966041	T	C	-0.015	0.001	2.4e-25	8.7e-05	108.2
Lipid Metabolism	TG	rs1799816	19	7125518	T	C	-0.055	0.008	1.2e-11	3.7e-05	46.1
Lipid Metabolism	TG	rs113216019	19	7212836	A	G	0.015	0.002	7.5e-22	7.4e-05	92.3
Lipid Metabolism	TG	rs4804414	19	7223785	T	C	0.020	0.001	4.3e-49	1.7e-04	216.9
Lipid Metabolism	TG	rs2115108	19	7963976	C	T	-0.015	0.001	1.4e-22	7.8e-05	95.6
Lipid Metabolism	TG	rs139473010	19	8416806	A	C	-0.033	0.004	2.3e-13	4.3e-05	53.8
Lipid Metabolism	TG	rs79293855	19	8420583	A	G	-0.047	0.004	2.6e-32	1.1e-04	140.1
Lipid Metabolism	TG	rs116843064	19	8429323	A	G	-0.236	0.005	1.0e-200	1.9e-03	2315.2
Lipid Metabolism	TG	rs140744493	19	8436373	T	C	-0.107	0.015	4.8e-13	5.2e-05	52.3
Lipid Metabolism	TG	rs557556361	19	8996805	A	G	-0.324	0.059	3.4e-08	5.4e-05	30.4
Lipid Metabolism	TG	rs5004102	19	9982334	C	A	-0.008	0.001	2.1e-08	2.5e-05	31.4
Lipid Metabolism	TG	rs149788261	19	10884515	A	C	-0.424	0.074	1.1e-08	8.3e-05	32.6
Lipid Metabolism	TG	rs11671872	19	11265020	A	G	-0.011	0.001	7.3e-14	4.5e-05	56.0
Lipid Metabolism	TG	rs74179972	19	11296830	G	A	0.022	0.003	1.2e-16	5.5e-05	68.6
Lipid Metabolism	TG	rs564310366	19	11297176	T	C	-0.339	0.062	4.8e-08	6.6e-05	29.8
Lipid Metabolism	TG	rs7249669	19	11302904	A	G	0.034	0.004	1.1e-20	7.0e-05	87.0
Lipid Metabolism	TG	rs11666808	19	18383506	C	T	-0.009	0.001	1.3e-10	3.3e-05	41.4
Lipid Metabolism	TG	rs1862644	19	18724315	A	C	-0.012	0.001	1.2e-16	5.5e-05	68.7
Lipid Metabolism	TG	rs11671696	19	19108375	T	C	-0.014	0.002	3.6e-18	6.1e-05	75.6
Lipid Metabolism	TG	rs547734426	19	19254381	T	C	-0.122	0.019	2.4e-10	4.9e-05	40.1
Lipid Metabolism	TG	rs187429064	19	19380513	G	A	-0.156	0.007	3.1e-114	4.2e-04	516.0
Lipid Metabolism	TG	rs10401969	19	19407718	C	T	-0.103	0.003	1.0e-200	1.3e-03	1618.8

Phenotype	Trait	SNP	Chr	Position (GRCh37/hg19)	EA	NEA	BETA	SE	P-value	R2	F
Lipid Metabolism	TG	rs10426801	19	19849201	T	A	-0.041	0.003	1.6e-39	1.4e-04	173.0
Lipid Metabolism	TG	rs541919209	19	20204158	G	C	-0.162	0.028	7.5e-09	5.6e-05	33.4
Lipid Metabolism	TG	rs144581040	19	20645763	G	A	-0.034	0.005	1.1e-12	4.1e-05	50.6
Lipid Metabolism	TG	rs187888358	19	21391607	A	G	-0.156	0.028	2.2e-08	3.7e-05	31.4
Lipid Metabolism	TG	rs34635674	19	33741951	G	A	-0.009	0.001	2.6e-10	3.3e-05	40.0
Lipid Metabolism	TG	rs7256564	19	33889593	G	A	-0.015	0.001	2.9e-23	7.9e-05	98.7
Lipid Metabolism	TG	rs140105410	19	35544552	A	G	-0.033	0.005	7.7e-12	3.7e-05	46.8
Lipid Metabolism	TG	rs45512696	19	35550878	T	C	0.024	0.002	3.0e-38	1.3e-04	167.2
Lipid Metabolism	TG	rs35462777	19	38241216	G	A	-0.008	0.001	6.8e-09	2.7e-05	33.6
Lipid Metabolism	TG	rs114806364	19	41752739	C	T	-0.011	0.002	2.2e-11	4.3e-05	44.8
Lipid Metabolism	TG	rs35538872	19	41754430	A	G	-0.089	0.009	1.2e-21	7.3e-05	91.3
Lipid Metabolism	TG	rs551434785	19	44009811	T	C	-0.396	0.071	2.3e-08	6.3e-05	31.3
Lipid Metabolism	TG	rs558152372	19	44356388	A	T	-0.303	0.047	1.1e-10	9.9e-05	41.7
Lipid Metabolism	TG	rs367913375	19	44976393	G	C	-0.382	0.055	3.3e-12	9.0e-05	48.5
Lipid Metabolism	TG	rs144908353	19	45078453	C	A	0.058	0.009	9.4e-11	3.4e-05	42.0
Lipid Metabolism	TG	rs62119328	19	45187163	G	A	0.046	0.005	9.5e-20	9.6e-05	82.7
Lipid Metabolism	TG	rs111278137	19	45215081	A	G	0.031	0.005	1.8e-09	2.9e-05	36.2
Lipid Metabolism	TG	rs144099906	19	45243898	C	T	-0.034	0.004	1.2e-14	4.9e-05	59.6
Lipid Metabolism	TG	rs573007449	19	45263188	T	C	0.128	0.020	3.3e-10	4.7e-05	39.5
Lipid Metabolism	TG	rs191815867	19	45271121	A	G	-0.079	0.012	1.1e-10	3.4e-05	41.7
Lipid Metabolism	TG	rs113330691	19	45295886	A	G	0.054	0.004	1.0e-45	1.6e-04	201.5
Lipid Metabolism	TG	rs547570291	19	45308756	A	G	0.134	0.021	1.7e-10	4.9e-05	40.7
Lipid Metabolism	TG	rs569365275	19	45331133	A	G	0.137	0.018	2.5e-14	6.4e-05	58.1
Lipid Metabolism	TG	rs76205446	19	45355267	A	T	0.069	0.007	5.2e-23	7.9e-05	97.6
Lipid Metabolism	TG	rs283815	19	45390333	G	A	0.072	0.002	1.0e-200	1.5e-03	1823.8
Lipid Metabolism	TG	rs552796536	19	45401987	A	G	0.162	0.017	1.5e-21	9.7e-05	90.9
Lipid Metabolism	TG	rs200337138	19	45406450	T	C	-0.107	0.018	9.1e-10	3.2e-05	37.5
Lipid Metabolism	TG	rs539667984	19	45421949	C	G	-0.348	0.021	2.8e-63	2.7e-04	282.0
Lipid Metabolism	TG	rs182067414	19	45425954	C	T	0.149	0.013	6.3e-30	1.1e-04	129.2
Lipid Metabolism	TG	rs5112	19	45430280	G	C	0.068	0.002	1.0e-200	1.5e-03	1730.6
Lipid Metabolism	TG	rs190291260	19	45438573	A	T	-0.633	0.098	1.3e-10	8.7e-05	41.3
Lipid Metabolism	TG	rs77659021	19	45460254	A	G	-0.040	0.007	3.3e-09	4.1e-05	35.0
Lipid Metabolism	TG	rs75178253	19	45461007	G	A	-0.019	0.003	2.6e-13	4.3e-05	53.5
Lipid Metabolism	TG	rs565517951	19	45487708	A	G	-0.397	0.071	2.6e-08	6.0e-05	31.0
Lipid Metabolism	TG	rs150484293	19	45496095	T	C	0.133	0.020	9.4e-12	4.9e-05	46.4
Lipid Metabolism	TG	rs74359223	19	45522289	A	G	0.094	0.007	5.5e-42	1.5e-04	184.3
Lipid Metabolism	TG	rs34034621	19	45542471	T	C	0.044	0.008	2.9e-08	2.5e-05	30.8
Lipid Metabolism	TG	rs188624748	19	45761361	T	A	0.052	0.009	1.9e-09	2.9e-05	36.0
Lipid Metabolism	TG	rs6509245	19	46468703	G	A	0.010	0.002	4.0e-08	2.4e-05	30.1
Lipid Metabolism	TG	rs62136106	19	46868385	G	A	-0.013	0.002	7.1e-18	6.0e-05	74.2
Lipid Metabolism	TG	rs184290	19	47627065	A	G	0.010	0.002	1.3e-10	3.3e-05	41.3
Lipid Metabolism	TG	rs2287922	19	49232226	A	G	0.017	0.001	3.2e-35	1.2e-04	153.3
Lipid Metabolism	TG	rs62132804	19	49272461	A	C	-0.018	0.003	2.3e-13	4.3e-05	53.7
Lipid Metabolism	TG	rs7256116	19	50038220	C	A	0.024	0.003	8.7e-20	6.7e-05	82.9
Lipid Metabolism	TG	rs798889	19	54793250	T	G	-0.013	0.002	6.3e-15	4.9e-05	60.8
Lipid Metabolism	TG	rs12610709	19	56102362	A	G	0.021	0.002	4.2e-29	1.0e-04	125.4
Lipid Metabolism	TG	rs7260649	19	57487497	C	T	0.010	0.001	1.2e-12	4.1e-05	50.5
Lipid Metabolism	TG	rs35073025	20	400872	C	A	0.008	0.001	3.7e-08	2.6e-05	30.3
Lipid Metabolism	TG	rs151235402	20	569164	T	C	0.048	0.006	5.2e-18	6.0e-05	74.8
Lipid Metabolism	TG	rs2317652	20	1414652	T	C	-0.008	0.001	7.8e-09	2.7e-05	33.3
Lipid Metabolism	TG	rs73075609	20	5580789	T	C	0.028	0.005	1.7e-09	2.9e-05	36.3
Lipid Metabolism	TG	rs440090	20	5785194	T	C	0.009	0.002	3.8e-08	2.5e-05	30.3
Lipid Metabolism	TG	rs78896696	20	10616382	T	G	-0.016	0.003	2.1e-09	2.9e-05	35.9
Lipid Metabolism	TG	rs686548	20	12973521	T	A	-0.008	0.001	7.2e-09	2.7e-05	33.5
Lipid Metabolism	TG	rs746748	20	25282967	T	C	-0.016	0.003	1.5e-08	2.6e-05	32.1
Lipid Metabolism	TG	rs117495226	20	25565515	C	G	-0.024	0.004	2.1e-08	2.5e-05	31.4

Phenotype	Trait	SNP	Chr	Position (GRCh37/hg19)	EA	NEA	BETA	SE	P-value	R2	F
Lipid Metabolism	TG	rs6120879	20	30202961	C	T	-0.013	0.002	1.0e-11	3.7e-05	46.3
Lipid Metabolism	TG	rs17305657	20	31806588	C	T	-0.015	0.002	1.8e-10	3.2e-05	40.7
Lipid Metabolism	TG	rs11696338	20	32687611	C	T	0.012	0.001	3.8e-16	5.3e-05	66.3
Lipid Metabolism	TG	rs62212343	20	33196937	A	C	-0.014	0.002	1.3e-08	2.6e-05	32.3
Lipid Metabolism	TG	rs6120973	20	34125311	C	T	-0.018	0.002	8.6e-19	6.3e-05	78.4
Lipid Metabolism	TG	rs12481388	20	38561681	T	C	-0.011	0.002	1.2e-11	3.7e-05	46.0
Lipid Metabolism	TG	rs56202412	20	39119602	A	T	-0.026	0.003	1.4e-21	7.3e-05	91.1
Lipid Metabolism	TG	rs1883711	20	39179822	C	G	0.059	0.004	7.9e-47	1.7e-04	206.5
Lipid Metabolism	TG	rs2865509	20	39239797	C	T	-0.008	0.001	3.5e-08	2.5e-05	30.4
Lipid Metabolism	TG	rs6102309	20	39856080	C	A	0.016	0.002	1.1e-25	8.8e-05	109.9
Lipid Metabolism	TG	rs6030982	20	42238857	A	G	0.011	0.002	8.4e-10	3.1e-05	37.7
Lipid Metabolism	TG	rs3212180	20	43034513	G	C	-0.011	0.002	3.5e-08	2.4e-05	30.4
Lipid Metabolism	TG	rs6032132	20	43981237	G	A	-0.010	0.002	5.2e-10	3.1e-05	38.6
Lipid Metabolism	TG	rs508681	20	44385064	C	G	-0.008	0.001	2.6e-08	2.5e-05	31.0
Lipid Metabolism	TG	rs6073958	20	44551855	C	T	0.049	0.002	1.1e-179	6.5e-04	817.0
Lipid Metabolism	TG	rs536134631	20	44740278	A	G	0.024	0.004	1.5e-09	3.8e-05	36.5
Lipid Metabolism	TG	rs35893705	20	45543616	A	G	0.009	0.001	4.7e-11	3.5e-05	43.3
Lipid Metabolism	TG	rs6066141	20	45597546	C	T	-0.017	0.002	1.6e-28	9.8e-05	122.7
Lipid Metabolism	TG	rs2092954	20	51016925	C	T	-0.011	0.001	7.7e-15	4.9e-05	60.4
Lipid Metabolism	TG	rs6123685	20	55836040	A	G	-0.012	0.002	1.1e-12	4.1e-05	50.7
Lipid Metabolism	TG	rs2039098	20	56112882	T	C	0.015	0.001	7.5e-25	8.7e-05	106.0
Lipid Metabolism	TG	rs41302559	20	56140439	A	G	-0.116	0.014	2.1e-16	5.8e-05	67.5
Lipid Metabolism	TG	rs7274432	20	56143533	T	C	0.015	0.002	2.0e-11	3.6e-05	45.0
Lipid Metabolism	TG	rs144468328	20	57780137	A	G	-0.041	0.006	3.3e-11	3.5e-05	44.0
Lipid Metabolism	TG	rs1151625	20	62369997	T	C	-0.017	0.003	1.3e-11	3.7e-05	45.8
Lipid Metabolism	TG	rs8126001	20	62711459	T	C	-0.016	0.001	6.9e-27	1.1e-04	115.3
Lipid Metabolism	TG	rs13048321	21	40459092	T	C	0.008	0.002	2.2e-08	2.5e-05	31.3
Lipid Metabolism	TG	rs8134638	21	40644170	C	T	0.012	0.001	1.2e-17	5.9e-05	73.1
Lipid Metabolism	TG	rs960230	21	42622479	G	A	0.012	0.002	2.7e-12	3.9e-05	48.9
Lipid Metabolism	TG	rs373687	21	46558780	C	T	0.009	0.001	6.4e-10	3.1e-05	38.2
Lipid Metabolism	TG	rs35665085	22	17625915	A	G	0.023	0.003	2.3e-14	4.7e-05	58.2
Lipid Metabolism	TG	rs73166619	22	21930093	T	C	-0.010	0.002	1.6e-09	2.9e-05	36.4
Lipid Metabolism	TG	rs9612520	22	24269459	T	G	-0.011	0.001	7.8e-15	4.9e-05	60.4
Lipid Metabolism	TG	rs4352308	22	29131788	T	C	0.012	0.002	2.7e-14	4.7e-05	58.0
Lipid Metabolism	TG	rs134596	22	29470115	G	A	0.009	0.002	1.6e-09	2.9e-05	36.5
Lipid Metabolism	TG	rs456300	22	29960335	G	A	-0.011	0.001	2.5e-14	4.6e-05	58.1
Lipid Metabolism	TG	rs5755804	22	36030683	T	C	0.011	0.001	1.4e-13	4.4e-05	54.8
Lipid Metabolism	TG	rs2267375	22	38601231	T	G	0.021	0.001	3.3e-50	1.8e-04	222.0
Lipid Metabolism	TG	rs7290979	22	39044427	C	A	0.015	0.002	3.4e-24	8.3e-05	102.9
Lipid Metabolism	TG	rs12484801	22	44325565	T	C	-0.012	0.002	4.3e-10	3.1e-05	39.0
Lipid Metabolism	TG	rs4253766	22	46623905	T	C	0.023	0.002	2.7e-24	8.3e-05	103.5
Lipid Metabolism	TG	rs79589523	22	50124139	A	C	-0.030	0.004	6.9e-13	4.1e-05	51.6
Lipid Metabolism	TG	rs73188911	22	50746706	T	C	-0.018	0.003	8.9e-13	4.1e-05	51.1

Phenotype	Trait	SNP	Chr	Position (GRCh37/hg19)	EA	NEA	BETA	SE	P-value	R2	F
Glucose Metabolism	HbA1c	rs1175549	1	3691727	C	A	-0.010	0.002	7.1e-13	3.0e-04	42.7
Glucose Metabolism	HbA1c	rs2375278	1	25529038	G	A	-0.011	0.002	1.0e-11	3.1e-04	43.4
Glucose Metabolism	HbA1c	rs267734	1	150951477	C	T	-0.010	0.002	5.0e-10	2.8e-04	40.6
Glucose Metabolism	HbA1c	rs7534795	1	155275553	T	C	0.010	0.002	2.1e-09	3.0e-04	39.1
Glucose Metabolism	HbA1c	rs857725	1	158607935	G	T	0.021	0.001	5.4e-55	1.5e-03	220.7
Glucose Metabolism	HbA1c	rs7547793	1	203653544	C	A	0.012	0.002	6.6e-09	2.5e-04	31.6
Glucose Metabolism	HbA1c	rs340882	1	214145731	G	C	0.008	0.001	1.5e-10	2.8e-04	41.8
Glucose Metabolism	HbA1c	rs10169706	2	5791194	T	C	0.026	0.005	1.5e-08	3.7e-04	31.9
Glucose Metabolism	HbA1c	rs12612492	2	24093756	T	C	0.019	0.002	1.9e-26	6.8e-04	97.9
Glucose Metabolism	HbA1c	rs1367173	2	43449385	T	C	-0.015	0.002	1.7e-14	4.1e-04	57.8
Glucose Metabolism	HbA1c	rs17037289	2	48587198	G	A	0.009	0.002	2.4e-09	2.7e-04	35.2

Phenotype	Trait	SNP	Chr	Position (GRCh37/hg19)	EA	NEA	BETA	SE	P-value	R2	F
Glucose Metabolism	HbA1c	rs7585751	2	169594581	C	T	0.018	0.003	3.4e-12	3.4e-04	44.0
Glucose Metabolism	HbA1c	rs2461387	2	169743887	G	A	-0.013	0.002	9.8e-20	6.0e-04	79.8
Glucose Metabolism	HbA1c	rs560887	2	169763148	C	T	0.031	0.001	5.5e-122	3.3e-03	480.9
Glucose Metabolism	HbA1c	rs17256082	2	175292364	C	T	0.007	0.001	3.2e-08	1.9e-04	28.2
Glucose Metabolism	HbA1c	rs13419763	2	219134950	T	C	0.008	0.001	5.5e-09	2.5e-04	32.7
Glucose Metabolism	HbA1c	rs12491937	3	12268244	G	A	-0.009	0.001	1.4e-13	3.3e-04	47.9
Glucose Metabolism	HbA1c	rs9818758	3	49382925	A	G	0.013	0.002	1.5e-13	4.1e-04	59.4
Glucose Metabolism	HbA1c	rs6445541	3	52880128	T	G	0.008	0.001	1.1e-08	2.3e-04	30.2
Glucose Metabolism	HbA1c	rs11719201	3	123068744	T	C	-0.013	0.002	2.4e-18	5.0e-04	74.0
Glucose Metabolism	HbA1c	rs6804915	3	170627909	A	C	-0.011	0.001	2.8e-16	4.1e-04	59.5
Glucose Metabolism	HbA1c	rs13092034	3	171501137	A	G	-0.009	0.002	2.7e-08	1.8e-04	24.5
Glucose Metabolism	HbA1c	rs13089972	3	171798694	A	T	0.011	0.001	1.9e-15	4.9e-04	62.9
Glucose Metabolism	HbA1c	rs188223066	4	6992446	G	A	0.649	0.134	1.8e-08	1.0e-03	23.4
Glucose Metabolism	HbA1c	rs13134327	4	144659795	A	G	0.014	0.001	2.8e-26	7.4e-04	105.8
Glucose Metabolism	HbA1c	rs6877043	5	154048367	C	T	-0.009	0.001	2.0e-10	2.9e-04	36.9
Glucose Metabolism	HbA1c	rs1948759	5	156442657	G	A	0.010	0.002	2.4e-08	2.2e-04	32.6
Glucose Metabolism	HbA1c	rs3778321	6	7250270	A	G	-0.011	0.002	4.2e-11	3.1e-04	43.9
Glucose Metabolism	HbA1c	rs6931514	6	20703952	G	A	0.010	0.001	1.2e-13	3.6e-04	53.1
Glucose Metabolism	HbA1c	rs12193223	6	24978511	G	C	-0.020	0.003	5.9e-09	2.7e-04	34.9
Glucose Metabolism	HbA1c	rs1800562	6	26093141	A	G	-0.038	0.003	2.3e-50	1.4e-03	201.2
Glucose Metabolism	HbA1c	rs129128	6	26125342	T	C	0.024	0.002	8.4e-40	1.1e-03	162.2
Glucose Metabolism	HbA1c	rs71559014	6	27122444	G	A	-0.024	0.003	1.1e-16	5.6e-04	60.4
Glucose Metabolism	HbA1c	rs34124863	6	28420249	T	C	-0.018	0.003	3.4e-13	3.5e-04	45.3
Glucose Metabolism	HbA1c	rs3134947	6	32145205	T	C	0.010	0.002	2.5e-10	2.1e-04	28.4
Glucose Metabolism	HbA1c	rs5012874	6	135267925	T	C	-0.012	0.002	1.7e-12	4.6e-04	51.5
Glucose Metabolism	HbA1c	rs9376090	6	135411228	C	T	-0.025	0.001	1.9e-62	2.2e-03	311.3
Glucose Metabolism	HbA1c	rs10231021	7	15060429	A	T	0.009	0.001	8.7e-14	3.2e-04	46.9
Glucose Metabolism	HbA1c	rs2971670	7	44226101	T	C	0.032	0.002	5.1e-88	2.3e-03	345.5
Glucose Metabolism	HbA1c	rs138917529	7	44235694	T	A	-0.038	0.006	9.5e-11	3.2e-04	43.7
Glucose Metabolism	HbA1c	rs13234131	7	73025975	G	A	0.011	0.002	2.1e-09	2.2e-04	31.9
Glucose Metabolism	HbA1c	rs4727979	7	123429697	C	A	-0.012	0.002	4.6e-08	2.0e-04	25.4
Glucose Metabolism	HbA1c	rs188556327	8	41500324	A	G	-0.086	0.015	6.2e-10	4.6e-04	33.8
Glucose Metabolism	HbA1c	rs114181382	8	41548886	C	G	-0.085	0.015	2.8e-10	4.7e-04	34.5
Glucose Metabolism	HbA1c	rs10101635	8	41587428	C	T	-0.038	0.004	2.0e-24	6.3e-04	90.2
Glucose Metabolism	HbA1c	rs141024463	8	41590143	T	C	-0.088	0.015	1.6e-10	4.5e-04	35.0
Glucose Metabolism	HbA1c	rs4737009	8	41630405	A	G	0.023	0.002	8.3e-56	1.6e-03	231.0
Glucose Metabolism	HbA1c	rs187305792	8	41655947	T	G	-0.060	0.011	8.5e-09	3.1e-04	32.0
Glucose Metabolism	HbA1c	rs2923444	8	42397004	T	C	-0.012	0.001	1.4e-19	5.3e-04	68.7
Glucose Metabolism	HbA1c	rs11558471	8	118185733	G	A	-0.015	0.001	3.4e-25	8.3e-04	116.3
Glucose Metabolism	HbA1c	rs2954021	8	126482077	G	A	0.007	0.001	1.9e-10	2.3e-04	34.0
Glucose Metabolism	HbA1c	rs10811661	9	22134094	C	T	-0.013	0.002	1.7e-14	3.9e-04	56.7
Glucose Metabolism	HbA1c	rs7861647	9	79977386	T	C	0.013	0.002	4.5e-14	5.0e-04	64.0
Glucose Metabolism	HbA1c	rs61750929	9	91495135	T	C	-0.028	0.003	9.5e-24	7.5e-04	95.9
Glucose Metabolism	HbA1c	rs7042939	9	110511408	G	A	-0.010	0.001	1.5e-15	4.2e-04	61.6
Glucose Metabolism	HbA1c	rs651007	9	136153875	T	C	0.011	0.002	3.3e-15	3.5e-04	51.8
Glucose Metabolism	HbA1c	rs3829109	9	139256766	A	G	-0.009	0.002	2.7e-08	2.4e-04	32.9
Glucose Metabolism	HbA1c	rs11257655	10	12307894	T	C	0.011	0.002	1.9e-13	3.2e-04	47.3
Glucose Metabolism	HbA1c	rs7918272	10	71001110	G	C	0.015	0.002	8.2e-23	7.2e-04	96.0
Glucose Metabolism	HbA1c	rs4746840	10	71064319	T	C	0.022	0.002	5.4e-24	6.3e-04	83.3
Glucose Metabolism	HbA1c	rs17476364	10	71094504	C	T	-0.086	0.002	1.0e-200	1.0e-02	1391.6
Glucose Metabolism	HbA1c	rs144844291	10	71115421	A	G	-0.125	0.015	6.6e-21	9.3e-04	73.8
Glucose Metabolism	HbA1c	rs189798995	10	106318002	G	A	1.006	0.177	8.3e-09	3.5e-03	32.2
Glucose Metabolism	HbA1c	rs7903146	10	114758349	T	C	0.013	0.001	1.0e-22	6.2e-04	90.2
Glucose Metabolism	HbA1c	rs4980325	11	234451	T	G	0.011	0.001	4.7e-14	4.6e-04	59.5
Glucose Metabolism	HbA1c	rs3842753	11	2181060	G	T	-0.007	0.002	3.9e-08	1.7e-04	22.0
Glucose Metabolism	HbA1c	rs147943288	11	5168482	C	G	0.218	0.033	2.2e-08	1.4e-03	42.9
Glucose Metabolism	HbA1c	rs360140	11	9776567	A	C	-0.008	0.001	9.6e-13	2.9e-04	41.8
Glucose Metabolism	HbA1c	rs11039154	11	47278502	T	C	-0.009	0.001	3.1e-09	2.6e-04	38.6
Glucose Metabolism	HbA1c	rs174559	11	61581656	A	G	-0.011	0.001	3.3e-13	3.9e-04	57.3
Glucose Metabolism	HbA1c	rs10830963	11	92708710	G	C	0.020	0.002	1.5e-36	1.2e-03	172.5
Glucose Metabolism	HbA1c	rs11224302	11	100456604	T	C	-0.016	0.002	4.6e-14	3.8e-04	50.9
Glucose Metabolism	HbA1c	rs608793	11	118986659	T	C	0.006	0.001	4.5e-08	1.9e-04	25.0

Phenotype	Trait	SNP	Chr	Position (GRCh37/hg19)	EA	NEA	BETA	SE	P-value	R2	F
Glucose Metabolism	HbA1c	rs117233107	12	4328521	A	G	-0.047	0.007	8.5e-11	3.9e-04	42.6
Glucose Metabolism	HbA1c	rs4760682	12	48512285	A	C	0.016	0.002	3.2e-20	6.3e-04	83.0
Glucose Metabolism	HbA1c	rs76815645	12	48709949	A	G	-0.013	0.002	3.4e-09	2.7e-04	35.5
Glucose Metabolism	HbA1c	rs10774624	12	111833788	A	G	0.009	0.001	4.2e-14	3.5e-04	51.2
Glucose Metabolism	HbA1c	rs11066301	12	112871372	G	A	-0.008	0.001	1.1e-09	2.4e-04	35.1
Glucose Metabolism	HbA1c	rs76533333	13	113352916	G	A	0.026	0.002	2.8e-29	8.7e-04	112.4
Glucose Metabolism	HbA1c	rs1278769	13	113536627	G	A	0.009	0.002	5.5e-12	2.6e-04	36.8
Glucose Metabolism	HbA1c	rs7994900	13	114553134	T	C	0.011	0.002	6.7e-14	4.2e-04	45.0
Glucose Metabolism	HbA1c	rs2273475	14	65268605	G	A	0.013	0.002	2.0e-09	2.4e-04	31.0
Glucose Metabolism	HbA1c	rs10151436	14	73616095	T	A	-0.013	0.002	3.9e-11	3.0e-04	38.3
Glucose Metabolism	HbA1c	rs1535464	14	100793431	A	G	-0.009	0.002	1.1e-08	2.0e-04	25.6
Glucose Metabolism	HbA1c	rs452306	15	65822777	T	C	-0.010	0.001	5.5e-13	3.8e-04	49.0
Glucose Metabolism	HbA1c	rs11248914	16	293562	C	T	-0.011	0.001	1.4e-14	5.2e-04	66.3
Glucose Metabolism	HbA1c	rs11643024	16	11443183	G	A	-0.008	0.002	8.0e-10	2.4e-04	31.4
Glucose Metabolism	HbA1c	rs7190771	16	28590030	A	G	0.009	0.001	6.0e-11	2.9e-04	42.8
Glucose Metabolism	HbA1c	rs7198799	16	68818390	T	C	0.008	0.001	4.8e-09	2.4e-04	35.1
Glucose Metabolism	HbA1c	rs247833	16	84581684	A	G	0.009	0.002	2.8e-08	1.9e-04	25.0
Glucose Metabolism	HbA1c	rs837763	16	88853729	T	C	0.018	0.001	5.2e-38	1.3e-03	183.3
Glucose Metabolism	HbA1c	rs11656775	17	17654319	G	A	-0.007	0.001	2.5e-08	1.8e-04	25.0
Glucose Metabolism	HbA1c	rs9914988	17	27183104	A	G	0.013	0.002	4.7e-17	4.2e-04	61.0
Glucose Metabolism	HbA1c	rs150926587	17	76059718	G	T	-0.082	0.015	5.3e-10	4.1e-04	30.6
Glucose Metabolism	HbA1c	rs111787532	17	76109516	T	C	-0.077	0.014	8.1e-09	3.2e-04	29.3
Glucose Metabolism	HbA1c	rs2748427	17	76121864	G	A	0.031	0.002	9.8e-49	1.9e-03	194.7
Glucose Metabolism	HbA1c	rs117595050	17	80666925	G	A	0.011	0.002	1.7e-12	3.5e-04	49.9
Glucose Metabolism	HbA1c	rs9909940	17	80689036	T	C	0.032	0.001	1.4e-116	3.6e-03	529.0
Glucose Metabolism	HbA1c	rs78357131	17	81026043	A	G	0.014	0.002	1.2e-09	3.2e-04	29.6
Glucose Metabolism	HbA1c	rs7220522	17	81046644	A	G	-0.009	0.002	2.5e-09	2.7e-04	30.6
Glucose Metabolism	HbA1c	rs28671200	18	43774444	T	G	0.009	0.002	1.6e-08	2.0e-04	25.6
Glucose Metabolism	HbA1c	rs17533945	19	17257802	C	T	0.013	0.001	1.6e-23	6.0e-04	83.6
Glucose Metabolism	HbA1c	rs12978547	19	33037212	G	C	-0.028	0.004	4.0e-12	3.8e-04	48.6
Glucose Metabolism	HbA1c	rs10405535	19	33072085	G	A	-0.012	0.002	6.5e-14	4.5e-04	58.1
Glucose Metabolism	HbA1c	rs737092	20	55990405	C	T	0.007	0.001	7.6e-09	2.2e-04	31.5
Glucose Metabolism	HbA1c	rs4820268	22	37469591	A	G	-0.018	0.001	1.2e-51	1.3e-03	189.6
Glucose Metabolism	HbA1c	rs8138197	22	43114551	A	G	-0.007	0.001	3.5e-08	2.2e-04	27.2
Glucose Metabolism	T2D	rs11583755	1	6672729	A	C	0.039	0.005	4.3e-16	6.2e-05	65.0
Glucose Metabolism	T2D	rs4845858	1	11304720	C	T	0.028	0.005	2.9e-08	2.9e-05	30.6
Glucose Metabolism	T2D	rs10916785	1	20731117	T	C	-0.027	0.005	4.3e-09	3.3e-05	34.7
Glucose Metabolism	T2D	rs11580180	1	26867453	A	G	-0.029	0.005	1.5e-08	3.0e-05	31.7
Glucose Metabolism	T2D	rs10915188	1	29024956	A	G	-0.029	0.005	3.7e-10	3.8e-05	39.7
Glucose Metabolism	T2D	rs61779275	1	39820310	C	T	0.075	0.005	3.4e-43	1.8e-04	187.4
Glucose Metabolism	T2D	rs3176466	1	51438365	C	T	-0.064	0.008	9.4e-16	6.2e-05	64.4
Glucose Metabolism	T2D	rs11208660	1	65983626	C	T	0.048	0.008	5.2e-09	3.2e-05	33.8
Glucose Metabolism	T2D	rs12740789	1	72752073	G	A	-0.039	0.006	9.2e-11	4.0e-05	41.5
Glucose Metabolism	T2D	rs197374	1	112289983	C	T	0.027	0.005	9.8e-09	3.1e-05	32.8
Glucose Metabolism	T2D	rs10801922	1	117529458	G	A	-0.038	0.005	1.2e-16	6.5e-05	68.2
Glucose Metabolism	T2D	rs41276588	1	118148384	G	A	0.038	0.005	4.1e-13	5.0e-05	52.0
Glucose Metabolism	T2D	rs2793829	1	120461253	C	T	0.070	0.007	8.1e-22	8.9e-05	92.5
Glucose Metabolism	T2D	rs79489938	1	147121000	G	A	-0.113	0.021	4.8e-08	2.9e-05	29.9
Glucose Metabolism	T2D	rs72692807	1	149894673	C	A	-0.054	0.009	6.0e-10	3.7e-05	38.4
Glucose Metabolism	T2D	rs145904381	1	151017991	T	C	-0.174	0.023	4.3e-14	5.5e-05	56.9
Glucose Metabolism	T2D	rs1194606	1	154294260	T	C	0.030	0.005	3.4e-08	2.9e-05	30.0
Glucose Metabolism	T2D	rs8847	1	155259323	C	T	0.033	0.005	2.7e-10	3.8e-05	39.5
Glucose Metabolism	T2D	rs9425591	1	172358697	G	T	-0.027	0.005	3.9e-09	3.4e-05	35.0
Glucose Metabolism	T2D	rs543874	1	177889480	A	G	0.035	0.006	8.7e-10	3.7e-05	38.1
Glucose Metabolism	T2D	rs10920254	1	201767474	C	G	0.036	0.005	2.8e-14	5.5e-05	57.8
Glucose Metabolism	T2D	rs1572993	1	205045087	G	A	0.031	0.005	2.0e-11	4.4e-05	45.4
Glucose Metabolism	T2D	rs963894	1	206625429	G	A	-0.029	0.005	1.3e-10	3.9e-05	40.6
Glucose Metabolism	T2D	rs340874	1	214159256	T	C	0.067	0.005	5.4e-48	2.0e-04	210.9
Glucose Metabolism	T2D	rs2820444	1	219741820	G	A	-0.047	0.005	8.7e-21	8.2e-05	85.7
Glucose Metabolism	T2D	rs348330	1	229672955	G	A	-0.052	0.006	6.5e-16	7.7e-05	65.2
Glucose Metabolism	T2D	rs867363	1	235579727	A	G	0.026	0.005	4.5e-08	2.9e-05	29.9
Glucose Metabolism	T2D	rs62107261	2	422144	T	C	-0.075	0.011	3.6e-11	4.2e-05	43.9

Phenotype	Trait	SNP	Chr	Position (GRCh37/hg19)	EA	NEA	BETA	SE	P-value	R2	F
Glucose Metabolism	T2D	rs35913461	2	653575	C	T	-0.050	0.006	9.9e-16	6.3e-05	65.3
Glucose Metabolism	T2D	rs34845373	2	25635771	A	G	-0.037	0.005	1.2e-12	4.9e-05	51.2
Glucose Metabolism	T2D	rs72803684	2	26192802	C	T	0.068	0.012	6.0e-09	3.3e-05	33.7
Glucose Metabolism	T2D	rs780094	2	27741237	T	C	0.062	0.005	1.4e-40	1.7e-04	176.8
Glucose Metabolism	T2D	rs7605475	2	43213411	T	C	0.029	0.005	2.6e-10	3.9e-05	40.3
Glucose Metabolism	T2D	rs13414140	2	43671176	C	T	-0.118	0.007	4.3e-60	2.6e-04	270.0
Glucose Metabolism	T2D	rs1861410	2	58933591	C	T	-0.035	0.005	2.9e-14	5.6e-05	58.9
Glucose Metabolism	T2D	rs10172678	2	59294558	T	C	-0.035	0.005	5.8e-14	5.3e-05	55.5
Glucose Metabolism	T2D	rs1912980	2	60581855	C	T	-0.051	0.005	4.4e-28	1.2e-04	121.0
Glucose Metabolism	T2D	rs2723064	2	65279805	T	C	-0.050	0.005	1.2e-25	1.1e-04	111.4
Glucose Metabolism	T2D	rs840976	2	65707760	C	T	-0.042	0.006	1.9e-11	5.3e-05	44.9
Glucose Metabolism	T2D	rs13004493	2	100598635	C	T	-0.068	0.012	1.8e-08	3.1e-05	31.8
Glucose Metabolism	T2D	rs17624303	2	105148418	C	T	-0.028	0.005	4.9e-08	2.9e-05	30.0
Glucose Metabolism	T2D	rs72836348	2	111888043	G	A	-0.056	0.008	3.2e-13	5.1e-05	53.3
Glucose Metabolism	T2D	rs34589210	2	112795492	G	A	0.039	0.007	6.9e-09	3.2e-05	33.9
Glucose Metabolism	T2D	rs9784137	2	121325908	G	A	-0.061	0.006	5.3e-21	8.4e-05	87.5
Glucose Metabolism	T2D	rs2033159	2	145261174	A	C	0.035	0.006	4.9e-10	3.7e-05	38.1
Glucose Metabolism	T2D	rs7609422	2	146348037	G	A	-0.029	0.005	2.7e-10	3.8e-05	39.4
Glucose Metabolism	T2D	rs7559658	2	147920213	T	C	0.034	0.006	3.2e-09	3.4e-05	35.2
Glucose Metabolism	T2D	rs3845843	2	152198598	C	T	0.031	0.005	2.8e-11	4.3e-05	44.8
Glucose Metabolism	T2D	rs13426680	2	158339550	A	G	-0.064	0.009	7.0e-12	4.5e-05	47.1
Glucose Metabolism	T2D	rs6710938	2	161333872	A	C	-0.040	0.005	1.4e-13	5.3e-05	55.1
Glucose Metabolism	T2D	rs76209259	2	161536620	T	C	-0.034	0.005	1.2e-09	3.6e-05	37.5
Glucose Metabolism	T2D	rs13389219	2	165528876	C	T	-0.065	0.005	7.3e-44	1.8e-04	190.1
Glucose Metabolism	T2D	rs355799	2	165694898	C	T	-0.044	0.007	3.4e-11	4.2e-05	44.2
Glucose Metabolism	T2D	rs1863196	2	166557510	A	G	-0.028	0.005	1.2e-09	3.5e-05	36.8
Glucose Metabolism	T2D	rs72917531	2	175238176	C	A	-0.035	0.006	1.0e-08	3.2e-05	33.3
Glucose Metabolism	T2D	rs6715901	2	179650954	G	A	-0.027	0.005	3.1e-09	3.4e-05	35.2
Glucose Metabolism	T2D	rs6741676	2	181618654	A	G	-0.032	0.005	3.6e-11	4.2e-05	43.7
Glucose Metabolism	T2D	rs13386084	2	191552157	C	T	-2.200	0.390	1.7e-08	3.9e-05	31.8
Glucose Metabolism	T2D	rs74766638	2	203309891	G	A	0.044	0.007	9.5e-10	3.5e-05	37.0
Glucose Metabolism	T2D	rs10932227	2	208918917	C	T	-0.028	0.005	3.9e-09	3.3e-05	34.7
Glucose Metabolism	T2D	rs13005841	2	212302573	A	T	-0.028	0.005	1.9e-08	3.0e-05	31.2
Glucose Metabolism	T2D	rs17354348	2	213835977	A	G	-0.029	0.005	3.0e-08	3.0e-05	30.8
Glucose Metabolism	T2D	rs113414093	2	219859171	G	A	0.072	0.013	4.3e-08	2.9e-05	30.0
Glucose Metabolism	T2D	rs2972145	2	227101309	T	C	0.090	0.005	7.5e-80	3.4e-04	351.6
Glucose Metabolism	T2D	rs7561798	2	228973660	A	G	0.028	0.005	1.2e-09	3.6e-05	37.3
Glucose Metabolism	T2D	rs838733	2	234323261	A	G	0.029	0.005	7.3e-10	3.6e-05	37.8
Glucose Metabolism	T2D	rs9842137	3	3649850	C	T	0.425	0.074	9.3e-09	3.2e-05	33.0
Glucose Metabolism	T2D	rs3872707	3	9514016	G	A	0.045	0.007	3.5e-11	4.3e-05	44.3
Glucose Metabolism	T2D	rs1584063	3	12266855	G	A	-0.030	0.005	8.7e-11	4.0e-05	42.0
Glucose Metabolism	T2D	rs11709077	3	12336507	G	A	-0.102	0.007	6.8e-47	2.0e-04	204.4
Glucose Metabolism	T2D	rs2470540	3	15718652	T	A	-0.030	0.005	6.2e-11	4.0e-05	42.1
Glucose Metabolism	T2D	rs35352848	3	23455582	T	C	-0.062	0.006	7.4e-28	1.1e-04	119.5
Glucose Metabolism	T2D	rs10490871	3	35667761	A	G	0.027	0.005	6.6e-09	3.2e-05	33.5
Glucose Metabolism	T2D	rs4327336	3	36896270	C	T	-0.026	0.005	4.9e-08	2.8e-05	29.3
Glucose Metabolism	T2D	rs62262091	3	47693664	C	T	0.056	0.009	1.1e-10	4.0e-05	41.9
Glucose Metabolism	T2D	rs4688760	3	49980596	C	T	0.034	0.005	1.1e-11	4.4e-05	46.2
Glucose Metabolism	T2D	rs6785040	3	63897915	T	C	-0.055	0.006	1.4e-17	6.9e-05	72.1
Glucose Metabolism	T2D	rs4132228	3	64708114	C	T	-0.047	0.005	6.2e-21	8.5e-05	88.7
Glucose Metabolism	T2D	rs853866	3	71648868	T	A	-0.026	0.005	2.7e-08	3.0e-05	31.3
Glucose Metabolism	T2D	rs11927126	3	72806622	A	G	0.029	0.005	2.5e-08	3.0e-05	30.8
Glucose Metabolism	T2D	rs1437055	3	86831077	A	C	-0.027	0.005	1.2e-08	3.1e-05	32.3
Glucose Metabolism	T2D	rs11716527	3	89986280	T	C	0.049	0.009	6.7e-09	3.2e-05	33.5
Glucose Metabolism	T2D	rs6438247	3	115084080	C	T	-0.044	0.007	4.0e-11	4.1e-05	43.1
Glucose Metabolism	T2D	rs11708067	3	123065778	A	G	-0.078	0.005	1.5e-46	1.9e-04	202.2
Glucose Metabolism	T2D	rs6801517	3	124918879	C	T	0.035	0.005	9.8e-13	4.8e-05	50.1
Glucose Metabolism	T2D	rs181752889	3	128579324	C	T	0.282	0.047	1.9e-09	3.5e-05	36.1
Glucose Metabolism	T2D	rs9828772	3	129333182	C	G	-0.052	0.008	2.7e-11	4.2e-05	43.9
Glucose Metabolism	T2D	rs1225052	3	131644937	A	G	0.027	0.005	1.0e-08	3.2e-05	33.2
Glucose Metabolism	T2D	rs9852406	3	135625498	C	T	0.038	0.005	9.0e-13	5.0e-05	52.0
Glucose Metabolism	T2D	rs9879531	3	136638975	A	G	0.036	0.005	1.8e-11	4.4e-05	45.6

Phenotype	Trait	SNP	Chr	Position (GRCh37/hg19)	EA	NEA	BETA	SE	P-value	R2	F
Glucose Metabolism	T2D	rs2054468	3	138057903	C	T	-0.032	0.005	2.7e-11	4.2e-05	44.2
Glucose Metabolism	T2D	rs73872717	3	141134569	C	T	-0.086	0.011	4.8e-15	5.9e-05	61.7
Glucose Metabolism	T2D	rs34573045	3	149196752	C	G	0.031	0.005	2.3e-11	4.4e-05	45.7
Glucose Metabolism	T2D	rs3796273	3	152160855	T	A	-0.031	0.005	1.8e-10	3.9e-05	40.6
Glucose Metabolism	T2D	rs74672008	3	152451616	G	A	-0.080	0.012	1.4e-11	4.4e-05	45.6
Glucose Metabolism	T2D	rs6808083	3	160171453	C	T	-0.031	0.005	6.9e-12	4.4e-05	46.3
Glucose Metabolism	T2D	rs1449348	3	168225055	C	T	-0.045	0.006	6.6e-12	4.6e-05	47.5
Glucose Metabolism	T2D	rs9873618	3	170733076	G	A	-0.058	0.005	2.4e-30	1.2e-04	129.8
Glucose Metabolism	T2D	rs488029	3	173119378	G	A	0.026	0.005	1.7e-08	3.1e-05	31.9
Glucose Metabolism	T2D	rs6776802	3	183740672	A	G	-0.052	0.008	4.1e-10	3.8e-05	39.3
Glucose Metabolism	T2D	rs9854769	3	185520948	A	G	0.108	0.005	1.1e-107	4.6e-04	481.3
Glucose Metabolism	T2D	rs12631579	3	185579678	T	C	0.042	0.007	2.8e-10	3.8e-05	39.5
Glucose Metabolism	T2D	rs3887925	3	186665645	C	T	0.042	0.005	6.5e-20	8.1e-05	85.0
Glucose Metabolism	T2D	rs11929598	3	187753264	T	C	0.046	0.006	9.3e-15	5.7e-05	59.5
Glucose Metabolism	T2D	rs7619708	3	195810187	T	C	-0.033	0.005	3.5e-10	3.8e-05	39.7
Glucose Metabolism	T2D	rs1531583	4	744972	G	T	0.099	0.011	7.1e-19	7.6e-05	78.8
Glucose Metabolism	T2D	rs73069940	4	1236502	C	G	-0.082	0.013	4.1e-10	3.7e-05	39.0
Glucose Metabolism	T2D	rs10011821	4	1777512	G	A	0.036	0.006	3.0e-08	3.0e-05	30.9
Glucose Metabolism	T2D	rs362307	4	3241845	C	T	0.050	0.009	3.8e-08	2.9e-05	30.1
Glucose Metabolism	T2D	rs4234730	4	6296343	A	G	0.082	0.005	8.2e-69	2.9e-04	303.6
Glucose Metabolism	T2D	rs2011603	4	18025484	G	A	0.038	0.005	1.6e-13	5.2e-05	54.2
Glucose Metabolism	T2D	rs11940813	4	20210953	A	G	0.037	0.007	2.6e-08	2.9e-05	30.7
Glucose Metabolism	T2D	rs10938398	4	45186139	G	A	0.043	0.005	3.9e-20	8.2e-05	85.4
Glucose Metabolism	T2D	rs2768969	4	49043925	T	C	-0.032	0.005	3.4e-10	3.8e-05	39.4
Glucose Metabolism	T2D	rs114447556	4	53207093	C	T	0.058	0.009	7.8e-11	4.0e-05	42.2
Glucose Metabolism	T2D	rs11729174	4	76551767	C	T	-0.034	0.006	2.5e-09	3.4e-05	35.2
Glucose Metabolism	T2D	rs11723275	4	77528821	A	C	0.027	0.005	4.3e-09	3.3e-05	34.2
Glucose Metabolism	T2D	rs3733227	4	83582211	C	G	-0.033	0.005	1.2e-11	4.4e-05	46.1
Glucose Metabolism	T2D	rs1444954	4	85372711	C	T	-0.151	0.026	8.6e-09	3.2e-05	33.2
Glucose Metabolism	T2D	rs7660000	4	89751858	C	T	-0.031	0.005	1.3e-09	3.5e-05	36.7
Glucose Metabolism	T2D	rs17227797	4	91249838	T	C	0.026	0.005	2.4e-08	3.0e-05	31.2
Glucose Metabolism	T2D	rs6821638	4	95091943	C	T	-0.029	0.005	3.7e-10	3.8e-05	39.5
Glucose Metabolism	T2D	rs2289043	4	96106322	A	G	-0.031	0.005	1.5e-10	3.9e-05	41.1
Glucose Metabolism	T2D	rs7695096	4	103932556	C	T	-0.038	0.005	1.3e-16	6.6e-05	68.6
Glucose Metabolism	T2D	rs17035289	4	106048291	T	C	0.043	0.006	5.7e-12	4.6e-05	47.7
Glucose Metabolism	T2D	rs4975198	4	129173123	C	T	0.031	0.006	3.1e-08	3.0e-05	30.8
Glucose Metabolism	T2D	rs1724557	4	137094048	C	A	-0.025	0.005	4.7e-08	2.8e-05	29.2
Glucose Metabolism	T2D	rs56218782	4	140893287	T	C	-0.031	0.005	2.8e-10	3.8e-05	39.4
Glucose Metabolism	T2D	rs75686861	4	145621328	G	A	0.047	0.008	4.2e-09	3.3e-05	34.4
Glucose Metabolism	T2D	rs10014477	4	153517741	T	A	-0.041	0.005	3.4e-16	6.3e-05	65.6
Glucose Metabolism	T2D	rs4691378	4	157704459	A	G	-0.037	0.005	4.6e-14	5.6e-05	57.9
Glucose Metabolism	T2D	rs55691245	4	185716100	G	A	-0.061	0.007	1.6e-19	7.9e-05	82.6
Glucose Metabolism	T2D	rs146886108	5	14751305	C	T	-0.390	0.033	1.9e-31	1.3e-04	136.3
Glucose Metabolism	T2D	rs17250977	5	14753745	A	G	0.122	0.014	8.2e-18	7.1e-05	73.9
Glucose Metabolism	T2D	rs55791375	5	14771845	C	T	0.033	0.005	6.8e-10	3.7e-05	38.5
Glucose Metabolism	T2D	rs114136102	5	36084426	T	C	0.072	0.012	2.1e-09	3.5e-05	36.0
Glucose Metabolism	T2D	rs4412123	5	44876288	T	C	-0.031	0.005	2.4e-11	4.3e-05	45.1
Glucose Metabolism	T2D	rs152839	5	50145266	C	T	-0.026	0.005	1.2e-08	3.1e-05	32.7
Glucose Metabolism	T2D	rs12187734	5	51763665	C	T	-0.029	0.005	2.2e-10	3.9e-05	40.6
Glucose Metabolism	T2D	rs62370480	5	52774510	G	A	0.034	0.006	2.0e-09	3.4e-05	35.8
Glucose Metabolism	T2D	rs6876198	5	53303595	T	C	0.047	0.005	1.4e-19	7.9e-05	82.0
Glucose Metabolism	T2D	rs459193	5	55806751	A	G	0.069	0.005	2.8e-39	1.7e-04	174.5
Glucose Metabolism	T2D	rs13179413	5	55868097	C	T	0.054	0.007	3.0e-15	7.3e-05	61.9
Glucose Metabolism	T2D	rs138596070	5	56261335	G	A	0.063	0.011	5.5e-09	3.3e-05	34.0
Glucose Metabolism	T2D	rs253412	5	74955841	G	A	0.046	0.005	2.8e-21	8.8e-05	91.4
Glucose Metabolism	T2D	rs6453282	5	76435688	A	T	-0.042	0.006	1.3e-11	4.5e-05	46.3
Glucose Metabolism	T2D	rs6898760	5	76449180	T	C	-0.026	0.005	3.5e-08	2.9e-05	30.0
Glucose Metabolism	T2D	rs12519500	5	78436905	C	A	-0.038	0.005	1.8e-15	6.0e-05	62.7
Glucose Metabolism	T2D	rs2410767	5	87705268	C	G	-0.032	0.006	6.8e-09	3.2e-05	33.7
Glucose Metabolism	T2D	rs77372998	5	101250990	A	G	0.236	0.030	1.9e-15	6.2e-05	63.0
Glucose Metabolism	T2D	rs1116046	5	101578769	A	T	0.080	0.010	1.3e-16	6.6e-05	68.8
Glucose Metabolism	T2D	rs75432112	5	102586407	G	A	0.134	0.011	5.3e-36	1.5e-04	157.8

Phenotype	Trait	SNP	Chr	Position (GRCh37/hg19)	EA	NEA	BETA	SE	P-value	R2	F
Glucose Metabolism	T2D	rs329122	5	133864599	G	A	0.026	0.005	1.3e-08	3.2e-05	32.9
Glucose Metabolism	T2D	rs890940	5	158026744	C	T	0.048	0.006	9.8e-18	7.2e-05	74.7
Glucose Metabolism	T2D	rs9379084	6	7231843	G	A	-0.075	0.008	3.7e-22	9.1e-05	94.1
Glucose Metabolism	T2D	rs2252857	6	7255015	G	A	0.043	0.005	2.4e-18	7.2e-05	75.6
Glucose Metabolism	T2D	rs577063987	6	20531791	G	A	0.218	0.036	1.9e-09	3.5e-05	36.1
Glucose Metabolism	T2D	rs35261542	6	20675792	C	A	0.122	0.005	1.6e-125	5.5e-04	573.2
Glucose Metabolism	T2D	rs10806924	6	20790509	A	G	-0.031	0.005	4.3e-11	4.1e-05	42.7
Glucose Metabolism	T2D	rs6919908	6	31244960	T	C	0.055	0.006	1.8e-21	8.7e-05	90.9
Glucose Metabolism	T2D	rs9265727	6	31302211	A	G	0.029	0.005	1.9e-09	3.5e-05	36.3
Glucose Metabolism	T2D	rs115470510	6	31618511	G	A	0.090	0.015	2.8e-09	3.4e-05	35.1
Glucose Metabolism	T2D	rs550127074	6	32499466	G	A	0.065	0.011	3.4e-09	4.2e-05	34.9
Glucose Metabolism	T2D	rs529082000	6	32500749	A	T	0.064	0.011	5.9e-09	4.1e-05	33.9
Glucose Metabolism	T2D	rs557673768	6	32544901	T	C	0.069	0.011	3.5e-10	4.8e-05	39.3
Glucose Metabolism	T2D	rs555746221	6	32616993	T	A	0.069	0.011	3.5e-10	4.8e-05	39.3
Glucose Metabolism	T2D	rs547454879	6	32625324	A	G	0.075	0.010	4.9e-14	6.8e-05	56.2
Glucose Metabolism	T2D	rs9275184	6	32654714	T	C	0.098	0.010	5.6e-24	1.2e-04	102.3
Glucose Metabolism	T2D	rs3763364	6	32807520	A	T	0.026	0.005	1.3e-08	3.1e-05	32.2
Glucose Metabolism	T2D	rs5745587	6	33545125	A	G	-0.034	0.006	5.3e-09	3.3e-05	34.4
Glucose Metabolism	T2D	rs10305420	6	39016636	C	T	-0.032	0.005	2.7e-11	4.3e-05	45.2
Glucose Metabolism	T2D	rs41273118	6	39072334	T	A	-0.032	0.005	6.1e-09	3.2e-05	33.9
Glucose Metabolism	T2D	rs34298980	6	40409243	T	C	-0.038	0.006	4.2e-09	4.1e-05	34.4
Glucose Metabolism	T2D	rs4714422	6	41012405	A	G	0.029	0.005	4.8e-08	2.8e-05	29.4
Glucose Metabolism	T2D	rs62401198	6	43801654	C	T	0.038	0.006	2.0e-09	3.4e-05	35.8
Glucose Metabolism	T2D	rs68137036	6	43820215	A	G	0.038	0.005	1.2e-13	5.2e-05	54.6
Glucose Metabolism	T2D	rs987237	6	50803050	A	G	0.048	0.006	2.6e-16	6.4e-05	67.0
Glucose Metabolism	T2D	rs16881572	6	51413013	G	A	0.075	0.014	4.5e-08	2.9e-05	30.0
Glucose Metabolism	T2D	rs60519666	6	107427166	G	A	-0.035	0.005	3.2e-12	4.6e-05	48.2
Glucose Metabolism	T2D	rs72951506	6	118011723	C	T	-0.042	0.006	9.2e-11	4.1e-05	42.3
Glucose Metabolism	T2D	rs9388501	6	126903011	T	C	0.040	0.005	5.0e-18	7.2e-05	74.9
Glucose Metabolism	T2D	rs9482769	6	127437576	G	A	0.029	0.005	2.9e-08	2.9e-05	30.7
Glucose Metabolism	T2D	rs3756784	6	131950233	T	G	0.032	0.006	3.0e-08	2.9e-05	30.3
Glucose Metabolism	T2D	rs1573090	6	137302159	T	G	-0.045	0.005	3.9e-22	9.0e-05	94.0
Glucose Metabolism	T2D	rs11155073	6	139837128	T	C	-0.030	0.005	4.4e-11	4.2e-05	43.7
Glucose Metabolism	T2D	rs62429515	6	140289389	C	T	-0.037	0.006	9.8e-11	3.9e-05	41.1
Glucose Metabolism	T2D	rs197482	6	143069315	T	C	0.030	0.005	2.6e-10	3.9e-05	40.2
Glucose Metabolism	T2D	rs6557267	6	153433701	C	T	0.033	0.005	2.0e-12	4.8e-05	50.2
Glucose Metabolism	T2D	rs543159	6	160776017	C	A	-0.032	0.005	1.3e-12	4.8e-05	49.6
Glucose Metabolism	T2D	rs73029263	6	164113762	A	G	-0.057	0.007	1.9e-16	6.6e-05	68.5
Glucose Metabolism	T2D	rs4721089	7	1872921	C	T	0.034	0.005	7.9e-10	3.7e-05	38.2
Glucose Metabolism	T2D	rs62452060	7	4683572	G	A	0.038	0.007	2.6e-08	3.0e-05	30.8
Glucose Metabolism	T2D	rs1990621	7	12283873	C	G	0.028	0.005	1.1e-09	3.5e-05	36.8
Glucose Metabolism	T2D	rs35559105	7	13897920	G	T	0.026	0.005	1.7e-08	3.0e-05	31.5
Glucose Metabolism	T2D	rs17168486	7	14898282	C	T	0.069	0.006	8.2e-31	1.3e-04	132.2
Glucose Metabolism	T2D	rs10228456	7	15063926	C	T	0.066	0.004	8.7e-48	2.1e-04	215.1
Glucose Metabolism	T2D	rs38250	7	15945102	C	G	-0.030	0.005	2.0e-08	3.1e-05	31.8
Glucose Metabolism	T2D	rs583769	7	18331915	G	A	0.031	0.005	2.7e-09	3.4e-05	35.8
Glucose Metabolism	T2D	rs75693095	7	23440057	G	C	0.112	0.017	1.4e-11	4.4e-05	45.5
Glucose Metabolism	T2D	rs12666580	7	23832169	G	A	-0.039	0.007	1.6e-08	3.1e-05	32.3
Glucose Metabolism	T2D	rs1513272	7	28200097	C	T	-0.081	0.004	4.0e-71	3.1e-04	324.8
Glucose Metabolism	T2D	rs917195	7	30728452	C	T	-0.047	0.005	2.7e-17	6.9e-05	72.1
Glucose Metabolism	T2D	rs17439448	7	40816653	C	T	0.040	0.007	3.0e-08	2.9e-05	30.6
Glucose Metabolism	T2D	rs118040648	7	43780096	A	G	0.118	0.021	3.3e-08	2.9e-05	30.5
Glucose Metabolism	T2D	rs2908286	7	44234737	C	T	0.068	0.006	2.6e-29	1.2e-04	125.4
Glucose Metabolism	T2D	rs12539264	7	48839003	A	G	0.029	0.005	6.6e-09	3.3e-05	34.3
Glucose Metabolism	T2D	rs2876826	7	50581972	A	G	0.031	0.005	3.3e-08	3.0e-05	31.0
Glucose Metabolism	T2D	rs35303761	7	69810892	A	G	0.032	0.005	2.2e-09	3.4e-05	35.8
Glucose Metabolism	T2D	rs35275911	7	74108249	G	C	0.033	0.006	2.5e-08	2.9e-05	30.6
Glucose Metabolism	T2D	rs10240790	7	89880949	A	G	0.028	0.005	4.7e-08	2.9e-05	29.9
Glucose Metabolism	T2D	rs221786	7	100266081	T	C	0.046	0.008	2.8e-09	3.4e-05	35.2
Glucose Metabolism	T2D	rs7800548	7	102481842	T	C	-0.031	0.005	1.3e-10	3.9e-05	40.5
Glucose Metabolism	T2D	rs4360229	7	102798857	G	C	-0.057	0.008	1.1e-12	4.8e-05	50.1
Glucose Metabolism	T2D	rs39328	7	103444978	C	T	0.028	0.005	3.3e-09	3.4e-05	35.7

Phenotype	Trait	SNP	Chr	Position (GRCh37/hg19)	EA	NEA	BETA	SE	P-value	R2	F
Glucose Metabolism	T2D	rs13239186	7	117510621	C	T	0.029	0.005	3.4e-09	3.3e-05	34.6
Glucose Metabolism	T2D	rs10238574	7	130452227	T	C	0.040	0.005	3.9e-18	7.1e-05	74.3
Glucose Metabolism	T2D	rs62492368	7	150537635	G	A	0.034	0.005	6.4e-12	4.5e-05	46.8
Glucose Metabolism	T2D	rs3808317	7	156944460	A	G	-0.049	0.005	3.3e-24	9.7e-05	101.6
Glucose Metabolism	T2D	rs112391427	8	4193125	A	G	0.088	0.016	4.4e-08	2.9e-05	29.9
Glucose Metabolism	T2D	rs2979138	8	8268437	C	T	0.032	0.005	2.6e-12	4.7e-05	49.0
Glucose Metabolism	T2D	rs34990153	8	9996389	A	G	-0.038	0.005	2.0e-16	6.4e-05	66.8
Glucose Metabolism	T2D	rs2409742	8	11069960	C	T	-0.036	0.005	7.3e-15	5.9e-05	61.6
Glucose Metabolism	T2D	rs73206315	8	12651322	T	C	0.029	0.005	5.3e-10	3.7e-05	38.7
Glucose Metabolism	T2D	rs17228833	8	14125121	T	C	0.027	0.005	4.8e-09	3.3e-05	34.0
Glucose Metabolism	T2D	rs17410962	8	19848080	G	A	-0.042	0.007	3.6e-09	3.3e-05	34.7
Glucose Metabolism	T2D	rs7003404	8	25871350	C	T	0.034	0.006	1.0e-09	3.5e-05	36.9
Glucose Metabolism	T2D	rs2725371	8	30854033	A	G	-0.037	0.005	2.1e-13	5.1e-05	52.9
Glucose Metabolism	T2D	rs35273426	8	41524688	C	T	-0.084	0.005	8.1e-55	2.3e-04	244.3
Glucose Metabolism	T2D	rs2241897	8	41555077	C	T	0.029	0.005	2.0e-09	3.4e-05	35.8
Glucose Metabolism	T2D	rs62515938	8	57483013	C	T	0.029	0.005	3.0e-08	2.9e-05	30.2
Glucose Metabolism	T2D	rs10808671	8	95967372	A	G	-0.037	0.004	2.4e-16	6.6e-05	68.7
Glucose Metabolism	T2D	rs149364428	8	97737741	G	A	0.224	0.031	5.7e-13	5.1e-05	51.8
Glucose Metabolism	T2D	rs34340810	8	105661926	G	C	-0.054	0.009	4.1e-10	3.8e-05	39.1
Glucose Metabolism	T2D	rs2142331	8	116636719	C	T	-0.038	0.005	5.1e-16	6.2e-05	64.7
Glucose Metabolism	T2D	rs13262027	8	118116861	G	A	0.044	0.007	1.2e-10	3.9e-05	41.1
Glucose Metabolism	T2D	rs11558471	8	118185733	A	G	-0.103	0.005	4.0e-98	4.2e-04	442.7
Glucose Metabolism	T2D	rs17772814	8	128711742	G	A	-0.075	0.013	3.4e-09	4.2e-05	35.1
Glucose Metabolism	T2D	rs4733612	8	129569999	G	A	-0.035	0.005	1.8e-11	4.3e-05	44.8
Glucose Metabolism	T2D	rs3757971	8	145545949	T	C	0.046	0.005	9.5e-22	8.8e-05	91.8
Glucose Metabolism	T2D	rs7015203	8	145976021	T	C	-0.027	0.005	4.5e-09	3.3e-05	34.0
Glucose Metabolism	T2D	rs2175055	9	1033354	C	T	0.029	0.005	5.6e-09	3.2e-05	33.4
Glucose Metabolism	T2D	rs10974438	9	4291928	A	C	0.047	0.005	9.0e-23	9.2e-05	95.9
Glucose Metabolism	T2D	rs4439216	9	19066115	A	G	0.036	0.005	1.3e-14	5.7e-05	59.7
Glucose Metabolism	T2D	rs7867635	9	20241069	T	C	0.037	0.006	4.2e-09	4.1e-05	34.5
Glucose Metabolism	T2D	rs10965246	9	22132698	T	C	-0.136	0.006	1.9e-111	4.8e-04	500.7
Glucose Metabolism	T2D	rs12555274	9	22136440	G	C	0.110	0.007	1.3e-55	2.9e-04	245.1
Glucose Metabolism	T2D	rs7029718	9	23358495	G	A	0.027	0.005	6.0e-09	3.3e-05	34.5
Glucose Metabolism	T2D	rs1412235	9	28410996	G	C	0.044	0.005	8.6e-19	7.6e-05	79.2
Glucose Metabolism	T2D	rs62558859	9	34076984	G	A	0.033	0.006	8.9e-09	3.2e-05	33.2
Glucose Metabolism	T2D	rs2225510	9	81372944	C	G	-0.035	0.006	1.6e-08	3.8e-05	31.9
Glucose Metabolism	T2D	rs17791513	9	81905590	A	G	-0.069	0.010	2.2e-12	4.7e-05	49.0
Glucose Metabolism	T2D	rs9410573	9	84311800	T	C	-0.059	0.005	1.9e-36	1.5e-04	158.1
Glucose Metabolism	T2D	rs7856840	9	85288745	T	G	0.030	0.005	2.5e-10	3.9e-05	40.7
Glucose Metabolism	T2D	rs10761324	9	96961247	T	A	0.036	0.005	4.2e-13	5.0e-05	52.1
Glucose Metabolism	T2D	rs7046845	9	97804641	A	C	-0.046	0.008	2.3e-08	3.0e-05	31.4
Glucose Metabolism	T2D	rs6415807	9	111958345	G	A	-0.031	0.005	3.0e-08	3.0e-05	31.2
Glucose Metabolism	T2D	rs7026688	9	125975397	G	A	-0.044	0.007	3.4e-11	4.2e-05	43.9
Glucose Metabolism	T2D	rs8176663	9	136144427	T	C	0.049	0.005	1.1e-24	1.0e-04	105.5
Glucose Metabolism	T2D	rs55923934	9	136888233	C	G	0.032	0.005	1.3e-09	3.5e-05	36.5
Glucose Metabolism	T2D	rs28463558	9	139242656	C	T	0.051	0.005	2.1e-27	1.1e-04	118.7
Glucose Metabolism	T2D	rs12779790	10	12328010	A	G	0.087	0.008	7.6e-28	1.4e-04	118.3
Glucose Metabolism	T2D	rs878017	10	13566204	A	G	-0.035	0.006	1.1e-08	3.9e-05	32.5
Glucose Metabolism	T2D	rs36051838	10	34018730	T	C	0.044	0.008	4.5e-08	2.9e-05	30.2
Glucose Metabolism	T2D	rs12263283	10	65305197	C	T	0.028	0.005	7.8e-09	3.2e-05	33.1
Glucose Metabolism	T2D	rs10998304	10	70342775	C	T	-0.031	0.004	1.8e-11	4.4e-05	46.2
Glucose Metabolism	T2D	rs187201965	10	71314539	C	G	-0.179	0.029	7.2e-10	3.7e-05	37.9
Glucose Metabolism	T2D	rs10823407	10	71325721	C	T	0.042	0.007	1.2e-08	3.1e-05	32.7
Glucose Metabolism	T2D	rs2642588	10	71466578	T	G	0.036	0.005	3.7e-13	5.1e-05	52.7
Glucose Metabolism	T2D	rs964706	10	72594665	G	A	0.035	0.006	1.1e-09	3.6e-05	37.3
Glucose Metabolism	T2D	rs2221169	10	77558657	C	T	0.028	0.005	1.6e-09	3.5e-05	36.5
Glucose Metabolism	T2D	rs12571751	10	80942631	A	G	-0.061	0.005	3.9e-40	1.7e-04	174.7
Glucose Metabolism	T2D	rs1782646	10	81040031	G	A	0.029	0.005	8.0e-10	3.6e-05	37.8
Glucose Metabolism	T2D	rs10788575	10	89768584	G	A	0.035	0.006	3.7e-08	2.9e-05	30.2
Glucose Metabolism	T2D	rs12778642	10	94464307	G	T	-0.092	0.005	1.2e-88	3.8e-04	399.1
Glucose Metabolism	T2D	rs10882884	10	99025611	T	A	-0.031	0.005	1.9e-11	4.2e-05	44.1
Glucose Metabolism	T2D	rs2862954	10	101912064	T	C	-0.029	0.004	1.6e-10	4.0e-05	41.8

Phenotype	Trait	SNP	Chr	Position (GRCh37/hg19)	EA	NEA	BETA	SE	P-value	R2	F
Glucose Metabolism	T2D	rs2250301	10	104548393	G	A	-0.032	0.005	1.3e-09	3.5e-05	36.9
Glucose Metabolism	T2D	rs10787287	10	112647195	C	T	0.036	0.005	6.6e-11	4.1e-05	42.8
Glucose Metabolism	T2D	rs141117847	10	114604287	A	G	0.175	0.019	1.2e-20	8.3e-05	86.7
Glucose Metabolism	T2D	rs140242150	10	114702962	G	A	0.279	0.049	1.6e-08	3.1e-05	31.9
Glucose Metabolism	T2D	rs149911199	10	114703335	G	A	-0.128	0.021	2.3e-09	3.4e-05	35.6
Glucose Metabolism	T2D	rs117569363	10	114704334	C	G	-0.097	0.013	5.1e-13	5.0e-05	52.4
Glucose Metabolism	T2D	rs181344886	10	114705589	G	T	-0.307	0.029	2.1e-26	1.1e-04	112.8
Glucose Metabolism	T2D	rs117736037	10	114735110	G	A	0.253	0.024	5.9e-26	1.1e-04	111.0
Glucose Metabolism	T2D	rs77653074	10	114742141	C	G	0.133	0.023	1.2e-08	3.2e-05	32.4
Glucose Metabolism	T2D	rs35011184	10	114749734	G	A	0.261	0.005	1.0e-200	2.2e-03	2332.5
Glucose Metabolism	T2D	rs138425717	10	114750211	A	G	-0.162	0.026	4.2e-10	3.8e-05	38.9
Glucose Metabolism	T2D	rs10885414	10	114861304	A	G	-0.079	0.005	5.8e-54	2.3e-04	238.1
Glucose Metabolism	T2D	rs11200629	10	124198585	A	G	-0.045	0.006	2.3e-13	6.3e-05	53.6
Glucose Metabolism	T2D	rs73398088	11	2096919	G	A	0.035	0.006	6.6e-09	3.2e-05	33.6
Glucose Metabolism	T2D	rs11043003	11	2203154	T	C	0.054	0.005	4.1e-23	9.3e-05	96.8
Glucose Metabolism	T2D	rs11023461	11	2632430	C	G	0.068	0.010	8.1e-13	4.9e-05	51.4
Glucose Metabolism	T2D	rs11023938	11	2756965	C	T	0.045	0.006	7.5e-13	5.0e-05	51.7
Glucose Metabolism	T2D	rs181923855	11	2774910	T	A	-0.163	0.028	3.1e-09	3.4e-05	35.2
Glucose Metabolism	T2D	rs234864	11	2857297	G	A	-0.070	0.005	4.4e-50	2.1e-04	221.8
Glucose Metabolism	T2D	rs10769936	11	8654528	T	C	0.035	0.005	6.3e-12	4.5e-05	46.8
Glucose Metabolism	T2D	rs7937716	11	9838235	A	G	0.032	0.005	6.3e-11	4.0e-05	42.1
Glucose Metabolism	T2D	rs78959242	11	14522000	T	C	0.129	0.018	6.7e-13	4.9e-05	51.4
Glucose Metabolism	T2D	rs5215	11	17408630	C	T	-0.068	0.005	3.4e-48	2.0e-04	211.8
Glucose Metabolism	T2D	rs180732108	11	32664422	A	T	-0.067	0.010	3.2e-12	4.6e-05	48.1
Glucose Metabolism	T2D	rs11555762	11	43876698	C	T	0.041	0.005	8.7e-17	6.6e-05	69.0
Glucose Metabolism	T2D	rs6485644	11	45855998	C	T	-0.029	0.005	1.1e-10	3.9e-05	40.8
Glucose Metabolism	T2D	rs1056387	11	47681370	T	G	-0.035	0.005	1.9e-14	5.6e-05	58.6
Glucose Metabolism	T2D	rs137930259	11	49590473	G	T	0.080	0.012	1.3e-11	4.4e-05	45.8
Glucose Metabolism	T2D	rs116861182	11	55588216	A	C	0.064	0.011	5.9e-09	3.3e-05	34.2
Glucose Metabolism	T2D	rs174541	11	61565908	T	C	-0.029	0.005	8.6e-10	3.5e-05	37.0
Glucose Metabolism	T2D	rs1783541	11	65294799	C	T	0.048	0.006	3.9e-17	6.9e-05	72.1
Glucose Metabolism	T2D	rs61881118	11	68999817	A	G	-0.037	0.006	1.2e-08	3.1e-05	32.1
Glucose Metabolism	T2D	rs3212880	11	69463679	A	G	-0.128	0.015	1.6e-17	7.0e-05	72.8
Glucose Metabolism	T2D	rs11602873	11	72460762	A	T	-0.098	0.006	8.9e-52	2.2e-04	232.6
Glucose Metabolism	T2D	rs480840	11	74625997	T	C	0.025	0.005	4.5e-08	2.9e-05	30.5
Glucose Metabolism	T2D	rs10899282	11	76504698	G	A	-0.031	0.006	2.0e-08	3.0e-05	31.4
Glucose Metabolism	T2D	rs11020124	11	92690661	T	C	0.076	0.005	9.6e-52	2.2e-04	232.3
Glucose Metabolism	T2D	rs3019212	11	93054457	C	T	0.036	0.005	1.9e-13	5.2e-05	54.3
Glucose Metabolism	T2D	rs11820614	11	93217394	C	T	0.027	0.005	3.0e-08	2.9e-05	30.1
Glucose Metabolism	T2D	rs10893827	11	128040810	A	G	-0.043	0.006	2.1e-11	4.3e-05	45.4
Glucose Metabolism	T2D	rs10750397	11	128234144	A	G	-0.048	0.005	1.0e-20	8.5e-05	88.6
Glucose Metabolism	T2D	rs67232546	11	128398938	C	T	0.055	0.008	1.4e-12	6.0e-05	50.8
Glucose Metabolism	T2D	rs118172320	12	4296534	A	G	-0.162	0.022	6.3e-14	5.5e-05	56.5
Glucose Metabolism	T2D	rs11063028	12	4300172	T	C	0.037	0.006	7.3e-10	3.7e-05	38.5
Glucose Metabolism	T2D	rs190243882	12	4347455	A	C	-0.428	0.042	7.0e-24	9.9e-05	101.7
Glucose Metabolism	T2D	rs143750586	12	4358078	A	G	-0.464	0.036	5.2e-38	1.6e-04	166.1
Glucose Metabolism	T2D	rs3217795	12	4386064	A	G	-0.102	0.008	1.3e-34	1.5e-04	151.6
Glucose Metabolism	T2D	rs3217862	12	4399087	T	G	0.042	0.006	1.8e-11	4.3e-05	45.2
Glucose Metabolism	T2D	rs527807612	12	4419392	C	G	-0.262	0.047	3.0e-08	3.0e-05	30.7
Glucose Metabolism	T2D	rs78470967	12	4521511	T	A	-0.083	0.013	9.1e-11	4.0e-05	41.8
Glucose Metabolism	T2D	rs11064266	12	6694655	A	G	0.035	0.006	4.7e-08	2.9e-05	30.2
Glucose Metabolism	T2D	rs10841860	12	21778904	G	A	-0.032	0.005	1.7e-09	3.4e-05	35.5
Glucose Metabolism	T2D	rs11048458	12	26465585	C	T	0.046	0.005	4.7e-18	7.2e-05	74.7
Glucose Metabolism	T2D	rs10771372	12	27962260	C	T	-0.072	0.006	2.2e-35	1.5e-04	154.5
Glucose Metabolism	T2D	rs10771813	12	31367856	A	C	0.026	0.005	2.6e-08	2.9e-05	30.4
Glucose Metabolism	T2D	rs10844518	12	33410780	A	G	0.033	0.005	8.8e-11	4.1e-05	42.5
Glucose Metabolism	T2D	rs2733289	12	41838235	T	C	0.030	0.005	4.0e-11	4.1e-05	42.8
Glucose Metabolism	T2D	rs11181613	12	43046449	C	A	-0.043	0.007	1.4e-10	4.0e-05	41.2
Glucose Metabolism	T2D	rs2732480	12	48736303	C	A	-0.034	0.005	3.1e-13	5.1e-05	53.7
Glucose Metabolism	T2D	rs12367809	12	50256063	C	T	0.033	0.005	3.1e-12	4.7e-05	48.7
Glucose Metabolism	T2D	rs1872635	12	54541750	A	G	-0.028	0.005	1.3e-08	3.2e-05	33.1
Glucose Metabolism	T2D	rs2583921	12	66170481	A	C	0.095	0.008	9.0e-33	1.4e-04	142.2

Phenotype	Trait	SNP	Chr	Position (GRCh37/hg19)	EA	NEA	BETA	SE	P-value	R2	F
Glucose Metabolism	T2D	rs7959830	12	66347368	G	T	0.030	0.005	1.9e-10	3.9e-05	40.5
Glucose Metabolism	T2D	rs4760895	12	71439127	A	G	-0.035	0.005	1.6e-14	5.7e-05	59.6
Glucose Metabolism	T2D	rs1580713	12	71500166	C	T	-0.039	0.005	7.2e-17	6.5e-05	68.2
Glucose Metabolism	T2D	rs11108094	12	95928113	C	A	0.060	0.009	1.2e-10	4.0e-05	41.8
Glucose Metabolism	T2D	rs113036477	12	97848227	C	T	-0.072	0.010	4.1e-13	5.0e-05	52.2
Glucose Metabolism	T2D	rs3764002	12	108618630	C	T	-0.040	0.005	2.7e-14	5.6e-05	58.0
Glucose Metabolism	T2D	rs4766891	12	118410302	G	T	0.050	0.007	1.1e-13	5.3e-05	54.9
Glucose Metabolism	T2D	rs77056961	12	121065826	A	G	0.111	0.017	8.3e-11	4.0e-05	42.1
Glucose Metabolism	T2D	rs75152767	12	121099805	G	A	0.210	0.030	1.5e-12	4.9e-05	49.9
Glucose Metabolism	T2D	rs56158042	12	121455589	G	A	-0.057	0.005	2.2e-29	1.2e-04	128.1
Glucose Metabolism	T2D	rs10773000	12	123736084	G	T	-0.038	0.005	1.4e-14	5.6e-05	58.6
Glucose Metabolism	T2D	rs3789967	12	124447339	T	G	-0.041	0.005	3.0e-17	6.9e-05	71.5
Glucose Metabolism	T2D	rs11830243	12	132544694	C	T	0.044	0.007	2.1e-09	3.4e-05	35.7
Glucose Metabolism	T2D	rs11614914	12	133070294	C	T	0.039	0.005	4.7e-15	5.8e-05	60.5
Glucose Metabolism	T2D	rs12828318	12	133766122	A	G	-0.035	0.006	9.4e-09	3.2e-05	33.5
Glucose Metabolism	T2D	rs12867151	13	23322525	A	G	0.037	0.007	1.8e-08	3.1e-05	31.9
Glucose Metabolism	T2D	rs34584161	13	26776999	A	G	-0.052	0.005	3.5e-22	8.9e-05	93.1
Glucose Metabolism	T2D	rs9319382	13	28245127	C	T	-0.028	0.005	2.9e-08	2.9e-05	30.5
Glucose Metabolism	T2D	rs576674	13	33554302	G	A	-0.061	0.006	1.3e-23	9.7e-05	100.7
Glucose Metabolism	T2D	rs7334971	13	41683827	A	C	-0.028	0.005	5.4e-09	3.3e-05	34.3
Glucose Metabolism	T2D	rs10459327	13	51080224	C	T	-0.058	0.007	4.5e-15	3.0e-04	62.3
Glucose Metabolism	T2D	rs9563574	13	58656599	T	C	-0.041	0.006	1.6e-11	4.4e-05	45.8
Glucose Metabolism	T2D	rs3861150	13	80611140	A	C	-0.034	0.005	1.1e-12	4.9e-05	51.1
Glucose Metabolism	T2D	rs1215451	13	80715893	G	A	-0.080	0.005	3.9e-54	2.3e-04	243.0
Glucose Metabolism	T2D	rs1475655	13	91963080	A	T	-0.044	0.005	2.5e-16	6.5e-05	67.7
Glucose Metabolism	T2D	rs7987740	13	109947213	T	C	-0.029	0.005	4.7e-10	3.7e-05	38.9
Glucose Metabolism	T2D	rs9521510	13	110426871	T	C	-0.029	0.005	3.3e-09	3.3e-05	34.8
Glucose Metabolism	T2D	rs8005994	14	29744532	A	G	-0.026	0.005	4.5e-08	2.9e-05	30.5
Glucose Metabolism	T2D	rs17522122	14	33302882	G	T	0.034	0.005	1.3e-13	5.3e-05	55.6
Glucose Metabolism	T2D	rs712316	14	35411537	T	G	0.042	0.007	1.3e-08	3.1e-05	32.7
Glucose Metabolism	T2D	rs980010	14	38822190	A	G	-0.037	0.005	1.3e-12	4.8e-05	50.1
Glucose Metabolism	T2D	rs12885033	14	47311988	C	A	-0.026	0.005	8.7e-09	3.1e-05	32.7
Glucose Metabolism	T2D	rs10137475	14	58797953	G	A	-0.025	0.005	4.4e-08	2.8e-05	29.4
Glucose Metabolism	T2D	rs4899280	14	69526307	C	T	0.028	0.005	1.0e-08	3.2e-05	33.1
Glucose Metabolism	T2D	rs8008540	14	74948180	C	T	-0.030	0.005	8.0e-11	4.1e-05	42.8
Glucose Metabolism	T2D	rs2056857	14	77300863	C	T	-0.026	0.005	2.6e-08	3.0e-05	30.8
Glucose Metabolism	T2D	rs10145154	14	79939525	C	T	0.055	0.006	1.1e-22	9.1e-05	95.1
Glucose Metabolism	T2D	rs8010382	14	91963722	A	G	0.032	0.005	5.7e-12	4.5e-05	46.9
Glucose Metabolism	T2D	rs73347525	14	101255172	A	G	-0.048	0.008	7.4e-09	4.0e-05	33.3
Glucose Metabolism	T2D	rs10133111	14	103377321	G	A	0.034	0.006	2.4e-08	3.0e-05	31.2
Glucose Metabolism	T2D	rs7152202	14	103886757	A	G	-0.028	0.005	2.9e-09	3.5e-05	36.0
Glucose Metabolism	T2D	rs12912777	15	38852386	C	T	0.059	0.007	1.8e-15	6.0e-05	62.7
Glucose Metabolism	T2D	rs34245505	15	40397191	C	G	0.038	0.006	1.8e-10	3.9e-05	41.0
Glucose Metabolism	T2D	rs1473781	15	41818917	G	A	0.032	0.005	1.5e-11	4.4e-05	45.8
Glucose Metabolism	T2D	rs2007854	15	52528507	G	A	-0.077	0.012	6.9e-11	4.1e-05	42.6
Glucose Metabolism	T2D	rs75332279	15	53099306	T	C	0.056	0.008	4.9e-12	4.6e-05	47.6
Glucose Metabolism	T2D	rs12441888	15	57340491	G	A	0.078	0.013	1.8e-09	3.5e-05	36.2
Glucose Metabolism	T2D	rs6494307	15	62394690	C	G	-0.040	0.005	2.6e-18	7.3e-05	76.4
Glucose Metabolism	T2D	rs34143602	15	63940058	G	A	-0.035	0.005	1.0e-13	5.2e-05	54.5
Glucose Metabolism	T2D	rs12900340	15	67264385	T	C	0.038	0.006	4.5e-09	3.3e-05	34.9
Glucose Metabolism	T2D	rs12917449	15	74331659	A	C	0.036	0.006	4.7e-10	3.8e-05	39.4
Glucose Metabolism	T2D	rs8029112	15	75808972	T	C	-0.040	0.005	5.2e-14	5.5e-05	57.5
Glucose Metabolism	T2D	rs952472	15	77776562	A	C	0.071	0.005	1.1e-43	1.9e-04	194.4
Glucose Metabolism	T2D	rs36111056	15	83461873	G	A	-0.034	0.006	2.0e-09	3.4e-05	35.8
Glucose Metabolism	T2D	rs7174644	15	90385868	C	T	-0.057	0.005	2.5e-29	1.2e-04	124.5
Glucose Metabolism	T2D	rs2290203	15	91512067	G	A	0.056	0.006	1.8e-22	9.2e-05	95.5
Glucose Metabolism	T2D	rs6600204	16	300177	A	G	-0.052	0.006	1.6e-17	6.9e-05	72.1
Glucose Metabolism	T2D	rs9933578	16	401385	A	G	-0.034	0.005	1.5e-10	4.0e-05	41.4
Glucose Metabolism	T2D	rs4984980	16	968292	G	A	0.035	0.006	3.8e-09	3.3e-05	34.2
Glucose Metabolism	T2D	rs12933120	16	3634746	C	A	0.042	0.007	3.2e-10	3.8e-05	40.0
Glucose Metabolism	T2D	rs7404466	16	15129092	C	T	-0.036	0.006	1.3e-08	3.1e-05	32.0
Glucose Metabolism	T2D	rs4408552	16	20383531	A	G	0.030	0.005	1.2e-09	3.5e-05	36.5

Phenotype	Trait	SNP	Chr	Position (GRCh37/hg19)	EA	NEA	BETA	SE	P-value	R2	F
Glucose Metabolism	T2D	rs8056890	16	28897452	G	A	0.029	0.005	1.9e-09	3.5e-05	36.3
Glucose Metabolism	T2D	rs35467921	16	30048553	C	T	0.037	0.005	5.6e-15	5.8e-05	60.1
Glucose Metabolism	T2D	rs7203132	16	53429775	G	A	-0.036	0.005	9.5e-15	5.7e-05	59.0
Glucose Metabolism	T2D	rs1421085	16	53800954	T	C	0.118	0.005	5.6e-144	6.3e-04	654.7
Glucose Metabolism	T2D	rs12929503	16	69565461	T	C	-0.042	0.005	2.2e-19	7.7e-05	79.9
Glucose Metabolism	T2D	rs55993634	16	75236763	C	G	-0.111	0.009	1.9e-37	1.6e-04	163.4
Glucose Metabolism	T2D	rs2966094	16	81538637	A	C	-0.039	0.005	1.8e-15	6.0e-05	63.0
Glucose Metabolism	T2D	rs11646052	16	85716463	A	G	0.026	0.005	2.6e-08	3.0e-05	31.3
Glucose Metabolism	T2D	rs9889005	16	88133115	C	T	-0.030	0.005	8.7e-09	3.2e-05	33.1
Glucose Metabolism	T2D	rs12920022	16	89564055	T	A	0.039	0.006	2.3e-09	3.5e-05	36.2
Glucose Metabolism	T2D	rs12453032	17	3833563	G	A	0.056	0.008	1.3e-12	4.9e-05	51.0
Glucose Metabolism	T2D	rs8071043	17	3988451	T	C	0.054	0.005	5.2e-28	1.2e-04	121.9
Glucose Metabolism	T2D	rs2243102	17	4839149	C	T	-0.025	0.005	4.9e-08	2.8e-05	29.4
Glucose Metabolism	T2D	rs858519	17	7531965	T	C	-0.026	0.005	1.9e-08	3.0e-05	31.1
Glucose Metabolism	T2D	rs7219033	17	9787958	G	A	0.029	0.005	4.9e-09	3.3e-05	34.5
Glucose Metabolism	T2D	rs11654081	17	17708129	T	C	-0.033	0.005	3.2e-12	4.6e-05	47.8
Glucose Metabolism	T2D	rs9913225	17	27570622	G	A	-0.027	0.005	5.4e-09	3.3e-05	34.5
Glucose Metabolism	T2D	rs35109242	17	29649223	A	G	-0.029	0.005	7.5e-10	3.6e-05	37.8
Glucose Metabolism	T2D	rs4795215	17	36055614	C	G	0.036	0.006	2.4e-08	3.0e-05	31.6
Glucose Metabolism	T2D	rs11651755	17	36099840	C	T	-0.058	0.005	4.1e-36	1.5e-04	159.0
Glucose Metabolism	T2D	rs7221875	17	37543328	G	A	0.037	0.005	2.4e-12	4.8e-05	49.5
Glucose Metabolism	T2D	rs684214	17	40696915	C	T	0.042	0.005	2.6e-16	6.4e-05	66.5
Glucose Metabolism	T2D	rs9900074	17	46124326	G	C	-0.055	0.009	3.2e-10	3.8e-05	40.0
Glucose Metabolism	T2D	rs35895680	17	47060322	C	A	-0.056	0.005	1.4e-28	1.2e-04	125.0
Glucose Metabolism	T2D	rs142228490	17	47202944	G	A	0.094	0.016	1.2e-08	3.1e-05	32.6
Glucose Metabolism	T2D	rs302864	17	56757584	G	A	0.058	0.011	4.8e-08	3.5e-05	29.8
Glucose Metabolism	T2D	rs4332	17	61564281	T	C	-0.034	0.004	8.7e-14	5.4e-05	56.8
Glucose Metabolism	T2D	rs56383601	17	61929968	A	C	-0.031	0.005	1.3e-09	3.5e-05	36.7
Glucose Metabolism	T2D	rs2080090	17	65828371	T	A	0.052	0.006	7.8e-19	7.6e-05	79.2
Glucose Metabolism	T2D	rs3924958	17	70645446	A	G	-0.048	0.008	2.8e-09	3.4e-05	35.0
Glucose Metabolism	T2D	rs94056	17	75384397	T	C	0.028	0.005	1.9e-08	3.0e-05	31.4
Glucose Metabolism	T2D	rs62075585	17	76762039	G	A	-0.029	0.005	1.3e-10	3.9e-05	41.1
Glucose Metabolism	T2D	rs2034126	17	77895915	A	G	-0.031	0.005	1.8e-08	3.0e-05	31.6
Glucose Metabolism	T2D	rs10083952	18	7073590	A	G	0.036	0.006	2.1e-08	3.7e-05	31.3
Glucose Metabolism	T2D	rs11662800	18	13271367	A	G	-0.028	0.005	2.4e-09	3.4e-05	35.7
Glucose Metabolism	T2D	rs113780182	18	13566624	C	T	0.031	0.005	1.9e-08	3.0e-05	31.6
Glucose Metabolism	T2D	rs303760	18	21083738	C	T	0.034	0.005	1.5e-12	4.9e-05	50.8
Glucose Metabolism	T2D	rs346240	18	40063830	G	A	-0.031	0.006	3.6e-08	2.9e-05	30.6
Glucose Metabolism	T2D	rs72926932	18	53050646	A	C	0.075	0.008	7.6e-20	8.0e-05	83.4
Glucose Metabolism	T2D	rs12963868	18	54679366	G	A	-0.029	0.005	2.8e-08	3.0e-05	31.0
Glucose Metabolism	T2D	rs9957145	18	56876228	G	A	-0.039	0.006	3.9e-10	3.7e-05	39.0
Glucose Metabolism	T2D	rs663640	18	57846077	C	T	0.050	0.005	1.1e-19	8.0e-05	83.6
Glucose Metabolism	T2D	rs76227980	18	58036384	C	T	-0.116	0.016	1.1e-12	4.9e-05	50.6
Glucose Metabolism	T2D	rs2658746	18	74582340	T	C	0.030	0.005	9.9e-11	4.0e-05	41.6
Glucose Metabolism	T2D	rs35004890	19	1224286	G	T	0.036	0.006	8.6e-10	3.6e-05	37.9
Glucose Metabolism	T2D	rs12977104	19	4949921	G	A	0.041	0.006	1.5e-12	4.8e-05	50.5
Glucose Metabolism	T2D	rs75253922	19	7240848	T	C	0.044	0.006	2.7e-14	5.6e-05	58.6
Glucose Metabolism	T2D	rs8107967	19	7972615	A	G	-0.035	0.005	1.6e-13	5.2e-05	54.5
Glucose Metabolism	T2D	rs2900660	19	13022859	C	A	0.044	0.005	4.3e-21	8.4e-05	88.0
Glucose Metabolism	T2D	rs739846	19	19419071	G	A	0.086	0.009	9.4e-24	9.6e-05	100.5
Glucose Metabolism	T2D	rs10418558	19	33889215	C	T	-0.038	0.005	1.9e-13	5.3e-05	54.8
Glucose Metabolism	T2D	rs429358	19	45411941	T	C	-0.073	0.007	4.0e-28	1.2e-04	122.3
Glucose Metabolism	T2D	rs10407429	19	46157237	G	A	-0.054	0.005	4.8e-31	1.3e-04	131.5
Glucose Metabolism	T2D	rs111713189	19	46204587	G	A	0.043	0.007	1.6e-09	3.5e-05	36.3
Glucose Metabolism	T2D	rs11667244	19	47580185	A	G	0.035	0.005	2.2e-12	4.8e-05	50.1
Glucose Metabolism	T2D	rs34587839	20	32300671	G	A	0.044	0.007	3.7e-11	4.2e-05	43.8
Glucose Metabolism	T2D	rs2056990	20	32623464	C	T	0.037	0.005	1.4e-14	5.6e-05	58.3
Glucose Metabolism	T2D	rs17265513	20	39832628	T	C	0.032	0.006	1.1e-08	3.1e-05	32.5
Glucose Metabolism	T2D	rs826951	20	42306260	A	G	0.043	0.007	1.5e-09	3.5e-05	36.9
Glucose Metabolism	T2D	rs12625671	20	42994812	T	C	0.065	0.007	6.7e-19	7.5e-05	77.9
Glucose Metabolism	T2D	rs191830490	20	43023355	G	A	-0.226	0.034	5.8e-11	4.2e-05	43.0
Glucose Metabolism	T2D	rs55966194	20	45599090	C	G	-0.045	0.005	1.7e-18	7.4e-05	77.5

Phenotype	Trait	SNP	Chr	Position (GRCh37/hg19)	EA	NEA	BETA	SE	P-value	R2	F
Glucose Metabolism	T2D	rs11699802	20	48832135	C	T	-0.030	0.005	3.6e-11	4.2e-05	44.0
Glucose Metabolism	T2D	rs2426439	20	50999627	T	C	0.036	0.005	2.1e-14	5.5e-05	57.8
Glucose Metabolism	T2D	rs3746709	20	57393941	C	T	0.034	0.005	8.3e-14	5.2e-05	54.6
Glucose Metabolism	T2D	rs34885433	20	61273960	G	A	-0.033	0.005	1.4e-11	4.5e-05	46.4
Glucose Metabolism	T2D	rs3787103	20	62295701	A	G	0.029	0.005	8.4e-10	3.6e-05	37.0
Glucose Metabolism	T2D	rs4809369	20	62470785	G	A	-0.034	0.005	4.3e-13	5.2e-05	53.7
Glucose Metabolism	T2D	rs75756987	21	47767295	G	C	-0.044	0.007	1.6e-09	3.5e-05	36.2
Glucose Metabolism	T2D	rs17518058	22	29378610	T	C	0.026	0.005	4.9e-08	2.9e-05	29.8
Glucose Metabolism	T2D	rs5763878	22	30591824	C	T	0.061	0.008	6.2e-14	5.4e-05	56.2
Glucose Metabolism	T2D	rs117001013	22	32348841	C	T	-0.047	0.008	1.4e-08	3.1e-05	31.8
Glucose Metabolism	T2D	rs4820324	22	38599857	G	C	0.028	0.005	1.1e-09	3.5e-05	36.3
Glucose Metabolism	T2D	rs55947928	22	41516608	A	G	-0.028	0.005	4.4e-08	2.9e-05	29.9
Glucose Metabolism	T2D	rs2294922	22	44379565	G	C	0.040	0.006	7.3e-13	5.0e-05	51.8
Glucose Metabolism	T2D	rs5771069	22	50435480	A	G	0.032	0.005	1.8e-12	4.8e-05	49.9
Glucose Metabolism	T2D	rs112915006	22	50604696	A	G	0.063	0.011	1.8e-08	3.0e-05	31.7
Glucose Metabolism	TG:HDL	rs114165349	1	27021913	C	G	0.138	0.009	1.6e-39	6.5e-04	262.8
Glucose Metabolism	TG:HDL	rs3768321	1	40035928	T	G	0.048	0.003	4.4e-34	5.6e-04	225.0
Glucose Metabolism	TG:HDL	rs7530647	1	40345611	A	G	0.020	0.003	2.6e-08	1.2e-04	47.1
Glucose Metabolism	TG:HDL	rs12097606	1	51227064	T	G	-0.034	0.004	9.1e-10	1.4e-04	57.0
Glucose Metabolism	TG:HDL	rs10889333	1	62957030	A	G	-0.066	0.003	3.4e-89	1.5e-03	609.0
Glucose Metabolism	TG:HDL	rs549777175	1	63413521	A	G	-0.369	0.040	6.0e-14	2.1e-04	85.6
Glucose Metabolism	TG:HDL	rs2613498	1	72752939	T	C	-0.022	0.003	2.7e-08	1.2e-04	47.0
Glucose Metabolism	TG:HDL	rs771458	1	93822349	C	G	-0.034	0.003	2.5e-17	2.7e-04	108.9
Glucose Metabolism	TG:HDL	rs1782815	1	98498898	A	C	-0.025	0.003	4.3e-09	1.3e-04	52.4
Glucose Metabolism	TG:HDL	rs12740374	1	109817590	T	G	-0.030	0.003	7.9e-16	2.4e-04	98.6
Glucose Metabolism	TG:HDL	rs390923	1	110499669	G	A	0.022	0.003	3.8e-12	1.8e-04	73.3
Glucose Metabolism	TG:HDL	rs141142383	1	149864493	T	C	-0.043	0.005	5.8e-14	2.1e-04	85.7
Glucose Metabolism	TG:HDL	rs6681742	1	150940358	T	C	0.021	0.003	3.5e-10	1.5e-04	59.8
Glucose Metabolism	TG:HDL	rs1760801	1	154259650	A	G	-0.025	0.003	4.7e-13	2.0e-04	79.5
Glucose Metabolism	TG:HDL	rs9425591	1	172358697	T	G	-0.020	0.003	5.3e-10	1.5e-04	58.6
Glucose Metabolism	TG:HDL	rs6676507	1	178508743	G	C	-0.021	0.003	2.9e-10	1.5e-04	60.4
Glucose Metabolism	TG:HDL	rs6677511	1	203510228	G	T	-0.018	0.003	1.2e-08	1.2e-04	49.3
Glucose Metabolism	TG:HDL	rs3903399	1	205041542	C	T	0.024	0.003	4.9e-10	1.5e-04	58.8
Glucose Metabolism	TG:HDL	rs2791544	1	219664400	G	A	0.028	0.003	2.4e-18	2.9e-04	115.9
Glucose Metabolism	TG:HDL	rs4445477	1	219759481	A	G	-0.023	0.003	1.8e-10	1.5e-04	61.8
Glucose Metabolism	TG:HDL	rs2807845	1	220996287	T	G	-0.024	0.003	5.4e-14	2.1e-04	85.9
Glucose Metabolism	TG:HDL	rs12062317	1	228062661	A	G	-0.020	0.003	1.4e-09	1.4e-04	55.6
Glucose Metabolism	TG:HDL	rs10779835	1	230299949	C	T	-0.084	0.003	2.5e-150	2.6e-03	1035.8
Glucose Metabolism	TG:HDL	rs16851339	1	230416832	A	T	-0.029	0.003	1.7e-14	2.2e-04	89.5
Glucose Metabolism	TG:HDL	rs115369740	1	230708632	A	G	0.062	0.009	1.1e-08	1.2e-04	49.6
Glucose Metabolism	TG:HDL	rs3820897	2	3642361	C	T	0.035	0.003	4.9e-17	2.7e-04	106.9
Glucose Metabolism	TG:HDL	rs601325	2	20982391	C	T	-0.039	0.004	1.6e-15	2.4e-04	96.5
Glucose Metabolism	TG:HDL	rs6734506	2	21134656	T	C	-0.023	0.003	3.5e-12	1.8e-04	73.5
Glucose Metabolism	TG:HDL	rs1042034	2	21225281	T	C	0.105	0.003	9.7e-163	2.8e-03	1122.4
Glucose Metabolism	TG:HDL	rs10168718	2	26053166	A	G	0.020	0.003	1.8e-09	1.4e-04	54.9
Glucose Metabolism	TG:HDL	rs13394970	2	26929282	G	T	0.018	0.003	1.0e-08	1.2e-04	49.8
Glucose Metabolism	TG:HDL	rs780094	2	27741237	C	T	-0.105	0.003	1.0e-200	4.0e-03	1624.1
Glucose Metabolism	TG:HDL	rs114940462	2	27861618	T	G	-0.067	0.006	3.1e-18	2.9e-04	115.2
Glucose Metabolism	TG:HDL	rs551204754	2	28065602	T	C	-0.183	0.026	1.2e-08	1.2e-04	49.4
Glucose Metabolism	TG:HDL	rs72807305	2	28447718	A	C	0.048	0.005	3.2e-17	2.7e-04	108.2
Glucose Metabolism	TG:HDL	rs7600413	2	37067137	G	A	-0.018	0.003	2.8e-08	1.2e-04	46.8
Glucose Metabolism	TG:HDL	rs17326656	2	48962291	T	G	0.028	0.003	8.5e-14	2.1e-04	84.6
Glucose Metabolism	TG:HDL	rs2723064	2	65279805	C	T	-0.027	0.003	3.6e-17	2.7e-04	107.8
Glucose Metabolism	TG:HDL	rs10211562	2	111930796	T	G	-0.017	0.003	3.1e-08	1.2e-04	46.5
Glucose Metabolism	TG:HDL	rs7578604	2	121308660	T	G	0.023	0.003	2.5e-10	1.5e-04	60.8
Glucose Metabolism	TG:HDL	rs72853953	2	135257744	G	A	-0.025	0.003	3.7e-09	1.3e-04	52.8
Glucose Metabolism	TG:HDL	rs12694933	2	158525226	T	C	0.027	0.004	2.1e-08	1.2e-04	47.8
Glucose Metabolism	TG:HDL	rs13389219	2	165528876	T	C	-0.054	0.003	1.8e-64	1.1e-03	436.6
Glucose Metabolism	TG:HDL	rs355799	2	165694898	T	C	-0.029	0.004	7.0e-11	1.6e-04	64.6
Glucose Metabolism	TG:HDL	rs13014069	2	171658382	C	T	0.019	0.003	6.4e-09	1.3e-04	51.2
Glucose Metabolism	TG:HDL	rs148358468	2	219590348	A	G	0.057	0.006	4.0e-15	2.3e-04	93.7
Glucose Metabolism	TG:HDL	rs148224341	2	219878668	C	T	0.158	0.019	7.2e-12	1.8e-04	71.3

Phenotype	Trait	SNP	Chr	Position (GRCh37/hg19)	EA	NEA	BETA	SE	P-value	R2	F
Glucose Metabolism	TG:HDL	rs2972146	2	227100698	T	G	0.056	0.003	4.2e-66	1.1e-03	447.9
Glucose Metabolism	TG:HDL	rs10933140	2	227209828	G	A	-0.024	0.003	1.9e-10	1.5e-04	61.6
Glucose Metabolism	TG:HDL	rs7596814	2	230128204	T	G	-0.019	0.003	3.2e-08	1.2e-04	46.4
Glucose Metabolism	TG:HDL	rs59916403	2	242370751	T	G	-0.020	0.003	1.4e-09	1.4e-04	55.7
Glucose Metabolism	TG:HDL	rs2067819	3	12359049	A	G	-0.035	0.003	1.1e-19	3.1e-04	125.3
Glucose Metabolism	TG:HDL	rs2470526	3	15681646	A	T	0.022	0.003	2.4e-10	1.5e-04	60.9
Glucose Metabolism	TG:HDL	rs9862795	3	49915506	T	A	0.019	0.003	3.2e-09	1.3e-04	53.2
Glucose Metabolism	TG:HDL	rs17052058	3	52344680	G	A	-0.044	0.003	3.1e-27	4.4e-04	177.4
Glucose Metabolism	TG:HDL	rs830620	3	71679148	T	C	-0.019	0.003	3.7e-09	1.3e-04	52.8
Glucose Metabolism	TG:HDL	rs79983121	3	127306462	T	C	0.023	0.003	2.2e-09	1.4e-04	54.4
Glucose Metabolism	TG:HDL	rs61789601	3	135954979	T	C	-0.046	0.003	1.7e-31	5.1e-04	207.1
Glucose Metabolism	TG:HDL	rs4683708	3	142653145	T	C	-0.017	0.003	3.2e-08	1.2e-04	46.5
Glucose Metabolism	TG:HDL	rs62271373	3	150066540	A	T	0.067	0.005	2.5e-23	3.7e-04	150.3
Glucose Metabolism	TG:HDL	rs9817452	3	156795414	T	G	-0.027	0.003	8.2e-17	2.6e-04	105.3
Glucose Metabolism	TG:HDL	rs5402	3	170727739	A	T	0.027	0.004	2.3e-08	1.2e-04	47.4
Glucose Metabolism	TG:HDL	rs79287178	3	172294500	A	G	0.065	0.008	8.4e-12	1.8e-04	70.9
Glucose Metabolism	TG:HDL	rs7631606	3	187628006	G	T	-0.021	0.003	4.3e-09	1.3e-04	52.4
Glucose Metabolism	TG:HDL	rs13101828	4	965720	G	A	-0.018	0.003	8.8e-09	1.2e-04	50.3
Glucose Metabolism	TG:HDL	rs59950280	4	3452345	A	G	0.026	0.003	1.2e-14	2.2e-04	90.5
Glucose Metabolism	TG:HDL	rs731450	4	3490328	T	C	0.025	0.003	1.6e-13	2.1e-04	82.6
Glucose Metabolism	TG:HDL	rs4450871	4	4990298	G	A	-0.020	0.003	2.6e-10	1.5e-04	60.6
Glucose Metabolism	TG:HDL	rs71603401	4	18034463	G	A	0.038	0.004	3.3e-16	2.5e-04	101.2
Glucose Metabolism	TG:HDL	rs73243877	4	26047616	G	A	0.044	0.003	5.6e-26	4.2e-04	168.7
Glucose Metabolism	TG:HDL	rs1055582	4	39700173	T	C	-0.020	0.003	4.1e-10	1.5e-04	59.3
Glucose Metabolism	TG:HDL	rs7684939	4	55509189	A	G	-0.023	0.003	6.5e-13	2.0e-04	78.5
Glucose Metabolism	TG:HDL	rs7675217	4	77413142	C	G	0.019	0.003	1.6e-09	1.4e-04	55.3
Glucose Metabolism	TG:HDL	rs1471251	4	87976359	T	A	0.046	0.003	9.8e-47	7.8e-04	313.0
Glucose Metabolism	TG:HDL	rs447990	4	88033352	G	T	0.023	0.003	7.9e-13	1.9e-04	77.9
Glucose Metabolism	TG:HDL	rs116417281	4	88385025	C	T	-0.032	0.004	5.9e-12	1.8e-04	71.9
Glucose Metabolism	TG:HDL	rs987469	4	89706643	G	C	0.019	0.003	6.3e-10	1.4e-04	58.0
Glucose Metabolism	TG:HDL	rs1126673	4	100045616	T	C	0.021	0.003	9.0e-10	1.4e-04	57.0
Glucose Metabolism	TG:HDL	rs13135092	4	103198082	G	A	0.064	0.005	1.3e-29	4.8e-04	194.1
Glucose Metabolism	TG:HDL	rs114816312	4	110638824	T	C	0.191	0.015	7.7e-26	4.2e-04	167.8
Glucose Metabolism	TG:HDL	rs11100083	4	157682598	C	T	-0.021	0.003	1.3e-08	1.2e-04	49.0
Glucose Metabolism	TG:HDL	rs185406435	5	53301571	G	A	0.030	0.004	1.9e-09	1.4e-04	54.8
Glucose Metabolism	TG:HDL	rs40270	5	55804552	C	A	0.052	0.003	2.3e-43	7.2e-04	289.5
Glucose Metabolism	TG:HDL	rs9687846	5	55861894	A	G	0.064	0.003	2.4e-59	1.0e-03	400.8
Glucose Metabolism	TG:HDL	rs10052346	5	78472599	T	G	-0.020	0.003	4.8e-10	1.5e-04	58.8
Glucose Metabolism	TG:HDL	rs115912456	5	82815158	G	A	-0.047	0.006	2.6e-09	1.3e-04	53.9
Glucose Metabolism	TG:HDL	rs1045241	5	118729286	T	C	-0.030	0.003	2.4e-17	2.7e-04	109.0
Glucose Metabolism	TG:HDL	rs253942	5	131320462	T	C	0.040	0.005	2.2e-09	1.4e-04	54.4
Glucose Metabolism	TG:HDL	rs72801474	5	132444128	A	G	-0.035	0.004	7.5e-11	1.6e-04	64.4
Glucose Metabolism	TG:HDL	rs11134475	5	156399950	G	A	0.027	0.003	2.1e-16	2.5e-04	102.5
Glucose Metabolism	TG:HDL	rs2914224	5	157998764	C	T	0.033	0.003	3.1e-18	2.9e-04	115.2
Glucose Metabolism	TG:HDL	rs4134963	6	20486798	T	C	-0.025	0.003	7.8e-10	1.4e-04	57.4
Glucose Metabolism	TG:HDL	rs115806990	6	29785700	A	G	0.088	0.013	1.5e-08	1.2e-04	48.7
Glucose Metabolism	TG:HDL	rs34794906	6	31237858	C	T	0.047	0.003	5.5e-39	6.4e-04	259.1
Glucose Metabolism	TG:HDL	rs10947210	6	31417742	C	T	0.064	0.005	1.1e-27	4.5e-04	180.6
Glucose Metabolism	TG:HDL	rs79118723	6	31446920	T	C	0.057	0.008	1.8e-09	1.4e-04	54.9
Glucose Metabolism	TG:HDL	rs78593564	6	31906828	A	G	0.124	0.010	5.4e-25	4.0e-04	161.9
Glucose Metabolism	TG:HDL	rs75483390	6	32585749	A	G	0.100	0.007	4.0e-34	5.6e-04	225.3
Glucose Metabolism	TG:HDL	rs2621366	6	32768980	A	C	0.021	0.003	1.1e-09	1.4e-04	56.5
Glucose Metabolism	TG:HDL	rs185139895	6	34177853	A	G	0.066	0.006	6.0e-18	2.8e-04	113.2
Glucose Metabolism	TG:HDL	rs9472125	6	43756169	T	C	-0.070	0.004	4.4e-41	6.8e-04	273.7
Glucose Metabolism	TG:HDL	rs9472136	6	43810021	T	C	-0.020	0.003	5.7e-10	1.5e-04	58.4
Glucose Metabolism	TG:HDL	rs2749024	6	52630587	A	T	-0.022	0.003	2.8e-11	1.7e-04	67.3
Glucose Metabolism	TG:HDL	rs62427983	6	107441254	C	T	-0.019	0.003	7.5e-09	1.3e-04	50.7
Glucose Metabolism	TG:HDL	rs2806381	6	109245554	T	C	0.023	0.003	7.1e-10	1.4e-04	57.7
Glucose Metabolism	TG:HDL	rs577721086	6	127440047	C	T	0.065	0.006	6.5e-19	3.0e-04	119.8
Glucose Metabolism	TG:HDL	rs9375694	6	130356608	A	G	0.023	0.003	3.0e-11	1.7e-04	67.1
Glucose Metabolism	TG:HDL	rs679582	6	139831180	A	G	-0.043	0.003	7.5e-40	6.6e-04	265.1
Glucose Metabolism	TG:HDL	rs1281955	6	153459444	A	T	-0.019	0.003	1.2e-09	1.4e-04	56.1

Phenotype	Trait	SNP	Chr	Position (GRCh37/hg19)	EA	NEA	BETA	SE	P-value	R2	F
Glucose Metabolism	TG:HDL	rs2450977	6	160713848	A	G	-0.025	0.003	1.2e-10	1.6e-04	63.1
Glucose Metabolism	TG:HDL	rs10455872	6	161010118	G	A	-0.045	0.005	5.3e-15	2.3e-04	92.9
Glucose Metabolism	TG:HDL	rs140570886	6	161013013	C	T	-0.127	0.010	6.6e-23	3.7e-04	147.5
Glucose Metabolism	TG:HDL	rs11751347	6	161092438	T	C	0.067	0.004	3.5e-38	6.3e-04	253.5
Glucose Metabolism	TG:HDL	rs73029270	6	164123095	G	A	-0.030	0.004	9.8e-11	1.6e-04	63.6
Glucose Metabolism	TG:HDL	rs34184329	7	1008307	T	C	0.028	0.004	2.1e-09	1.4e-04	54.5
Glucose Metabolism	TG:HDL	rs852388	7	5574239	C	G	0.025	0.003	2.4e-10	1.5e-04	60.9
Glucose Metabolism	TG:HDL	rs6968865	7	17287269	T	A	0.024	0.003	4.3e-14	2.2e-04	86.6
Glucose Metabolism	TG:HDL	rs6461354	7	17914600	T	C	0.029	0.003	1.1e-19	3.1e-04	125.3
Glucose Metabolism	TG:HDL	rs7786102	7	25965759	A	G	-0.029	0.003	1.2e-16	2.6e-04	104.1
Glucose Metabolism	TG:HDL	rs1406754	7	26396198	T	G	-0.022	0.003	6.4e-12	1.8e-04	71.7
Glucose Metabolism	TG:HDL	rs498475	7	28256240	A	G	-0.018	0.003	3.1e-08	1.2e-04	46.5
Glucose Metabolism	TG:HDL	rs2070971	7	44197583	T	G	0.031	0.004	1.1e-11	1.7e-04	70.1
Glucose Metabolism	TG:HDL	rs117603660	7	71954045	A	G	-0.107	0.012	1.2e-12	1.9e-04	76.7
Glucose Metabolism	TG:HDL	rs566263305	7	72028755	A	G	-0.143	0.019	1.4e-09	1.4e-04	55.7
Glucose Metabolism	TG:HDL	rs35867424	7	72032358	A	G	-0.074	0.008	7.4e-15	2.3e-04	91.9
Glucose Metabolism	TG:HDL	rs549069127	7	72731431	A	G	0.204	0.030	3.7e-08	1.1e-04	46.1
Glucose Metabolism	TG:HDL	rs142001788	7	72833107	T	C	0.094	0.012	9.1e-10	1.4e-04	57.0
Glucose Metabolism	TG:HDL	rs55747707	7	73037366	A	G	-0.129	0.003	1.0e-200	4.1e-03	1657.5
Glucose Metabolism	TG:HDL	rs2178026	7	73062019	G	C	0.032	0.004	2.4e-10	1.5e-04	61.0
Glucose Metabolism	TG:HDL	rs111914893	7	74002323	T	C	0.041	0.006	1.5e-08	1.2e-04	48.7
Glucose Metabolism	TG:HDL	rs2302429	7	75614777	A	G	0.023	0.003	9.2e-09	1.2e-04	50.1
Glucose Metabolism	TG:HDL	rs41301433	7	75615439	G	C	-0.135	0.019	6.9e-09	1.3e-04	51.0
Glucose Metabolism	TG:HDL	rs10260148	7	130430969	T	C	0.043	0.003	9.5e-35	5.7e-04	229.6
Glucose Metabolism	TG:HDL	rs157936	7	130585623	G	T	-0.019	0.003	2.0e-08	1.2e-04	47.9
Glucose Metabolism	TG:HDL	rs535241194	7	150296609	G	A	0.028	0.003	6.4e-13	2.0e-04	78.5
Glucose Metabolism	TG:HDL	rs2948294	8	8094961	G	A	-0.025	0.003	3.6e-15	2.3e-04	94.0
Glucose Metabolism	TG:HDL	rs10095103	8	9226095	C	T	0.030	0.003	5.8e-21	3.3e-04	134.0
Glucose Metabolism	TG:HDL	rs7460226	8	10197718	G	A	-0.023	0.003	1.1e-12	1.9e-04	76.9
Glucose Metabolism	TG:HDL	rs28588745	8	10647044	T	A	0.049	0.003	7.7e-37	6.1e-04	244.2
Glucose Metabolism	TG:HDL	rs10108052	8	11617763	A	G	0.022	0.003	1.1e-10	1.6e-04	63.3
Glucose Metabolism	TG:HDL	rs7819276	8	11755804	C	T	0.029	0.003	6.5e-19	3.0e-04	119.8
Glucose Metabolism	TG:HDL	rs1495741	8	18272881	A	G	-0.040	0.003	1.3e-26	4.3e-04	173.1
Glucose Metabolism	TG:HDL	rs539412189	8	19458595	G	A	0.193	0.024	4.1e-11	1.6e-04	66.1
Glucose Metabolism	TG:HDL	rs10088203	8	19622809	T	C	-0.032	0.003	3.0e-21	3.4e-04	136.0
Glucose Metabolism	TG:HDL	rs558679944	8	19634363	G	A	-0.273	0.021	1.9e-26	4.3e-04	171.9
Glucose Metabolism	TG:HDL	rs144473554	8	19639879	A	T	-0.181	0.024	1.8e-09	1.4e-04	54.9
Glucose Metabolism	TG:HDL	rs3758060	8	19673973	C	T	0.208	0.014	2.2e-32	5.3e-04	213.1
Glucose Metabolism	TG:HDL	rs539118782	8	19676524	A	G	-0.230	0.034	3.1e-08	1.2e-04	46.6
Glucose Metabolism	TG:HDL	rs187013686	8	19784052	T	C	-0.286	0.020	3.6e-31	5.1e-04	204.8
Glucose Metabolism	TG:HDL	rs254	8	19811897	G	C	-0.173	0.004	1.0e-200	5.7e-03	2291.7
Glucose Metabolism	TG:HDL	rs144578061	8	19813878	C	A	-0.127	0.015	2.0e-12	1.9e-04	75.2
Glucose Metabolism	TG:HDL	rs3289	8	19823192	C	T	0.237	0.008	4.3e-133	2.3e-03	915.4
Glucose Metabolism	TG:HDL	rs186998446	8	19865643	A	T	-0.263	0.021	2.3e-24	3.9e-04	157.6
Glucose Metabolism	TG:HDL	rs75609851	8	19871080	A	G	-0.299	0.013	1.7e-78	1.3e-03	534.4
Glucose Metabolism	TG:HDL	rs551447383	8	19880070	C	T	-0.318	0.023	2.9e-30	4.9e-04	198.5
Glucose Metabolism	TG:HDL	rs546061549	8	19902004	C	T	-0.305	0.040	4.8e-10	1.5e-04	58.9
Glucose Metabolism	TG:HDL	rs181349205	8	19939605	C	G	0.308	0.015	2.4e-61	1.0e-03	414.7
Glucose Metabolism	TG:HDL	rs183300997	8	19952828	C	T	0.086	0.012	2.1e-08	1.2e-04	47.7
Glucose Metabolism	TG:HDL	rs77675968	8	19959983	C	T	0.066	0.007	7.2e-14	2.1e-04	85.1
Glucose Metabolism	TG:HDL	rs181703090	8	19970086	T	C	0.092	0.012	1.2e-10	1.6e-04	63.0
Glucose Metabolism	TG:HDL	rs150564454	8	19972140	A	G	-0.180	0.012	5.4e-32	5.2e-04	210.5
Glucose Metabolism	TG:HDL	rs147972269	8	20026384	C	T	-0.160	0.018	1.8e-12	1.9e-04	75.5
Glucose Metabolism	TG:HDL	rs2081687	8	59388565	C	T	-0.024	0.003	7.5e-13	1.9e-04	78.1
Glucose Metabolism	TG:HDL	rs17215671	8	64646646	A	C	0.021	0.003	3.3e-09	1.3e-04	53.2
Glucose Metabolism	TG:HDL	rs13269725	8	72459889	G	A	0.042	0.005	7.6e-13	1.9e-04	78.0
Glucose Metabolism	TG:HDL	rs2017657	8	73426371	T	G	-0.017	0.003	4.7e-08	1.1e-04	45.3
Glucose Metabolism	TG:HDL	rs2737220	8	116637685	C	T	-0.023	0.003	1.8e-12	1.9e-04	75.5
Glucose Metabolism	TG:HDL	rs72647336	8	126445055	A	G	0.109	0.006	1.6e-50	8.4e-04	339.5
Glucose Metabolism	TG:HDL	rs28601761	8	126500031	G	C	-0.117	0.003	1.0e-200	5.0e-03	2025.1
Glucose Metabolism	TG:HDL	rs17420217	8	126543638	C	T	0.069	0.009	1.9e-10	1.5e-04	61.7
Glucose Metabolism	TG:HDL	rs1031497	8	126591392	C	T	0.028	0.003	3.5e-19	3.0e-04	121.7

Phenotype	Trait	SNP	Chr	Position (GRCh37/hg19)	EA	NEA	BETA	SE	P-value	R2	F
Glucose Metabolism	TG:HDL	rs60987469	8	144303753	T	C	-0.030	0.003	8.2e-14	2.1e-04	84.7
Glucose Metabolism	TG:HDL	rs1016565	9	1032567	A	G	0.022	0.003	2.0e-10	1.5e-04	61.5
Glucose Metabolism	TG:HDL	rs296886	9	86592026	G	A	-0.024	0.003	1.8e-10	1.5e-04	61.7
Glucose Metabolism	TG:HDL	rs10797119	9	92202495	C	T	0.023	0.003	2.6e-13	2.0e-04	81.2
Glucose Metabolism	TG:HDL	rs2131919	9	95283887	G	A	0.026	0.003	4.2e-10	1.5e-04	59.3
Glucose Metabolism	TG:HDL	rs62565259	9	102162570	T	C	-0.025	0.003	3.8e-09	1.3e-04	52.8
Glucose Metabolism	TG:HDL	rs7024300	9	107589567	T	C	-0.064	0.008	1.0e-11	1.7e-04	70.3
Glucose Metabolism	TG:HDL	rs188308962	9	107620797	G	A	0.257	0.032	4.0e-11	1.6e-04	66.3
Glucose Metabolism	TG:HDL	rs2275543	9	107651174	C	T	0.037	0.004	6.2e-13	2.0e-04	78.6
Glucose Metabolism	TG:HDL	rs1044531	9	117098650	T	C	-0.032	0.005	1.5e-08	1.2e-04	48.7
Glucose Metabolism	TG:HDL	rs2519093	9	136141870	T	C	-0.030	0.003	2.0e-13	2.0e-04	82.0
Glucose Metabolism	TG:HDL	rs150770378	10	52616492	A	G	0.145	0.014	1.4e-17	2.7e-04	110.6
Glucose Metabolism	TG:HDL	rs11510917	10	61403131	G	A	-0.026	0.003	8.1e-10	1.4e-04	57.3
Glucose Metabolism	TG:HDL	rs4454603	10	65012750	T	C	-0.037	0.003	2.0e-32	5.3e-04	213.5
Glucose Metabolism	TG:HDL	rs3829126	10	74714177	T	G	0.031	0.004	1.2e-08	1.2e-04	49.2
Glucose Metabolism	TG:HDL	rs1475366	10	94781857	C	T	-0.035	0.004	1.3e-10	1.6e-04	62.7
Glucose Metabolism	TG:HDL	rs6583855	10	94843032	C	A	-0.035	0.003	5.0e-28	4.5e-04	183.0
Glucose Metabolism	TG:HDL	rs11187498	10	95290777	C	T	0.036	0.003	8.7e-17	2.6e-04	105.2
Glucose Metabolism	TG:HDL	rs577525	10	99769388	C	T	0.024	0.003	9.4e-14	2.1e-04	84.3
Glucose Metabolism	TG:HDL	rs2803619	10	113934384	C	G	0.039	0.003	5.6e-29	4.7e-04	189.6
Glucose Metabolism	TG:HDL	rs4573621	10	113987548	G	A	0.034	0.003	9.5e-17	2.6e-04	104.9
Glucose Metabolism	TG:HDL	rs740746	10	115792787	A	G	-0.029	0.003	2.6e-16	2.5e-04	101.9
Glucose Metabolism	TG:HDL	rs11199845	10	122992475	T	C	-0.018	0.003	5.0e-09	1.3e-04	51.9
Glucose Metabolism	TG:HDL	rs12793175	11	10384303	A	G	0.023	0.003	3.4e-08	1.2e-04	46.3
Glucose Metabolism	TG:HDL	rs10832027	11	13357183	A	G	0.024	0.003	8.7e-13	1.9e-04	77.6
Glucose Metabolism	TG:HDL	rs192849769	11	14451992	C	T	-0.288	0.029	5.6e-16	2.5e-04	99.6
Glucose Metabolism	TG:HDL	rs79634051	11	14561945	C	G	-0.055	0.008	5.5e-09	1.3e-04	51.7
Glucose Metabolism	TG:HDL	rs10835211	11	27701365	A	G	0.029	0.003	8.0e-16	2.4e-04	98.5
Glucose Metabolism	TG:HDL	rs77403571	11	45913607	A	G	-0.052	0.005	4.6e-15	2.3e-04	93.3
Glucose Metabolism	TG:HDL	rs80078546	11	46195220	G	C	-0.044	0.006	1.6e-10	1.5e-04	62.1
Glucose Metabolism	TG:HDL	rs112487171	11	47160969	T	A	-0.064	0.007	2.3e-13	2.0e-04	81.6
Glucose Metabolism	TG:HDL	rs326222	11	47259668	C	T	0.053	0.003	1.0e-53	9.0e-04	361.7
Glucose Metabolism	TG:HDL	rs145758003	11	47414372	A	G	-0.251	0.025	9.2e-16	2.4e-04	98.1
Glucose Metabolism	TG:HDL	rs147216895	11	47431477	T	C	-0.243	0.025	2.1e-15	2.4e-04	95.6
Glucose Metabolism	TG:HDL	rs145276599	11	48444711	C	T	-0.044	0.004	1.4e-18	2.9e-04	117.6
Glucose Metabolism	TG:HDL	rs11039884	11	48644829	C	A	0.063	0.009	2.7e-09	1.3e-04	53.8
Glucose Metabolism	TG:HDL	rs113221947	11	50016058	A	G	-0.040	0.004	4.5e-16	2.5e-04	100.2
Glucose Metabolism	TG:HDL	rs78297458	11	50611510	C	T	-0.072	0.009	2.1e-11	1.7e-04	68.2
Glucose Metabolism	TG:HDL	rs4625441	11	51196033	C	T	-0.032	0.004	9.9e-12	1.7e-04	70.4
Glucose Metabolism	TG:HDL	rs74966104	11	54867854	G	A	-0.036	0.004	1.6e-12	1.9e-04	75.9
Glucose Metabolism	TG:HDL	rs12421131	11	56034587	G	A	-0.034	0.004	1.8e-11	1.7e-04	68.6
Glucose Metabolism	TG:HDL	rs10792091	11	57119156	C	T	0.026	0.004	7.1e-09	1.3e-04	50.9
Glucose Metabolism	TG:HDL	rs102275	11	61557803	C	T	0.077	0.003	3.8e-120	2.0e-03	825.0
Glucose Metabolism	TG:HDL	rs17156442	11	61614023	T	C	0.059	0.006	1.4e-15	2.4e-04	96.8
Glucose Metabolism	TG:HDL	rs3802932	11	63988045	A	G	0.071	0.005	1.7e-26	4.3e-04	172.4
Glucose Metabolism	TG:HDL	rs10750766	11	65473798	A	C	0.029	0.003	5.0e-17	2.7e-04	106.8
Glucose Metabolism	TG:HDL	rs4930352	11	66066993	T	G	-0.021	0.003	1.3e-10	1.6e-04	62.8
Glucose Metabolism	TG:HDL	rs11228374	11	68597805	A	T	0.023	0.003	6.2e-12	1.8e-04	71.8
Glucose Metabolism	TG:HDL	rs7110856	11	109968072	C	A	0.020	0.003	1.3e-08	1.2e-04	49.1
Glucose Metabolism	TG:HDL	rs117847213	11	111421906	G	A	-0.055	0.007	5.6e-12	1.8e-04	72.1
Glucose Metabolism	TG:HDL	rs148308118	11	116269843	G	A	0.289	0.039	1.8e-09	1.4e-04	54.9
Glucose Metabolism	TG:HDL	rs528868277	11	116472039	T	C	0.330	0.032	1.2e-16	2.6e-04	104.1
Glucose Metabolism	TG:HDL	rs541471464	11	116519130	A	G	0.349	0.032	6.4e-19	3.0e-04	119.9
Glucose Metabolism	TG:HDL	rs2105610	11	116535126	C	T	0.081	0.004	3.7e-55	9.2e-04	371.7
Glucose Metabolism	TG:HDL	rs149473698	11	116550869	T	C	0.321	0.029	6.9e-20	3.1e-04	126.6
Glucose Metabolism	TG:HDL	rs145716904	11	116552753	T	C	0.236	0.015	2.7e-35	5.8e-04	233.4
Glucose Metabolism	TG:HDL	rs71480317	11	116572169	C	T	-0.073	0.009	4.0e-11	1.6e-04	66.2
Glucose Metabolism	TG:HDL	rs11216103	11	116574488	A	G	0.254	0.005	1.0e-200	5.7e-03	2289.7
Glucose Metabolism	TG:HDL	rs6589563	11	116590787	A	G	0.259	0.005	1.0e-200	7.3e-03	2960.9
Glucose Metabolism	TG:HDL	rs111683933	11	116642049	C	T	-0.099	0.011	2.2e-14	2.2e-04	88.6
Glucose Metabolism	TG:HDL	rs45611741	11	116663128	T	C	0.633	0.015	1.0e-200	4.4e-03	1788.3
Glucose Metabolism	TG:HDL	rs142172482	11	116672392	C	T	0.268	0.017	1.9e-39	6.5e-04	262.3

Phenotype	Trait	SNP	Chr	Position (GRCh37/hg19)	EA	NEA	BETA	SE	P-value	R2	F
Glucose Metabolism	TG:HDL	rs2239013	11	116693353	T	C	-0.066	0.005	3.5e-25	4.1e-04	163.2
Glucose Metabolism	TG:HDL	rs147448028	11	116693647	A	G	0.315	0.024	5.3e-26	4.2e-04	168.9
Glucose Metabolism	TG:HDL	rs138326449	11	116701354	A	G	-1.755	0.035	1.0e-200	6.4e-03	2574.4
Glucose Metabolism	TG:HDL	rs7941912	11	116815762	A	G	-0.121	0.007	7.7e-43	7.1e-04	285.9
Glucose Metabolism	TG:HDL	rs116995028	11	116999072	T	C	0.233	0.011	9.0e-63	1.1e-03	424.7
Glucose Metabolism	TG:HDL	rs570337877	11	117098448	T	C	-0.062	0.009	2.1e-08	1.2e-04	47.8
Glucose Metabolism	TG:HDL	rs116987336	11	117175658	A	G	0.216	0.008	9.8e-107	1.8e-03	731.4
Glucose Metabolism	TG:HDL	rs539572120	11	117193201	G	A	0.225	0.033	4.9e-08	1.1e-04	45.2
Glucose Metabolism	TG:HDL	rs1003081	11	118913993	T	C	0.022	0.003	5.3e-12	1.8e-04	72.3
Glucose Metabolism	TG:HDL	rs1622638	11	121800971	A	G	0.021	0.003	1.7e-10	1.5e-04	62.0
Glucose Metabolism	TG:HDL	rs11612	12	6728165	A	G	0.028	0.003	1.8e-11	1.7e-04	68.6
Glucose Metabolism	TG:HDL	rs11045171	12	20470199	G	A	-0.041	0.003	1.8e-25	4.1e-04	165.2
Glucose Metabolism	TG:HDL	rs12369443	12	20582651	G	A	-0.025	0.003	2.3e-10	1.5e-04	61.0
Glucose Metabolism	TG:HDL	rs7969341	12	21385604	G	A	0.023	0.003	3.9e-08	1.1e-04	45.9
Glucose Metabolism	TG:HDL	rs10842703	12	26456188	T	A	0.022	0.003	9.7e-10	1.4e-04	56.8
Glucose Metabolism	TG:HDL	rs11052977	12	34035030	C	A	-0.018	0.003	1.0e-08	1.2e-04	49.9
Glucose Metabolism	TG:HDL	rs2122982	12	57781893	A	G	-0.041	0.003	2.1e-29	4.8e-04	192.4
Glucose Metabolism	TG:HDL	rs10783828	12	57861484	G	A	-0.025	0.003	3.9e-14	2.2e-04	86.9
Glucose Metabolism	TG:HDL	rs11113118	12	107199142	A	G	0.023	0.003	2.3e-10	1.5e-04	61.1
Glucose Metabolism	TG:HDL	rs149793040	12	109661672	G	A	-0.393	0.029	8.9e-28	4.5e-04	181.2
Glucose Metabolism	TG:HDL	rs7298135	12	109698566	A	T	0.025	0.003	5.0e-10	1.5e-04	58.8
Glucose Metabolism	TG:HDL	rs4766578	12	111904371	A	T	-0.022	0.003	7.2e-12	1.8e-04	71.3
Glucose Metabolism	TG:HDL	rs4075625	12	122610593	T	A	0.023	0.003	9.7e-09	1.2e-04	50.0
Glucose Metabolism	TG:HDL	rs597365	12	123185538	T	G	-0.045	0.004	5.9e-24	3.8e-04	154.7
Glucose Metabolism	TG:HDL	rs7133378	12	124409502	A	G	-0.046	0.003	4.3e-43	7.1e-04	287.7
Glucose Metabolism	TG:HDL	rs825453	12	124508758	T	A	0.045	0.003	6.1e-45	7.5e-04	300.5
Glucose Metabolism	TG:HDL	rs117285912	12	124795283	A	G	0.046	0.007	4.0e-08	1.1e-04	45.8
Glucose Metabolism	TG:HDL	rs61941660	12	125319181	C	T	0.041	0.005	1.4e-13	2.1e-04	83.1
Glucose Metabolism	TG:HDL	rs34505073	13	50237255	A	G	-0.061	0.009	2.2e-08	1.2e-04	47.6
Glucose Metabolism	TG:HDL	rs12863082	13	71824582	C	T	0.021	0.003	2.3e-08	1.2e-04	47.4
Glucose Metabolism	TG:HDL	rs7140110	13	114544024	C	T	0.031	0.003	1.1e-19	3.1e-04	125.2
Glucose Metabolism	TG:HDL	rs75073260	13	114599617	G	T	-0.036	0.004	2.3e-15	2.4e-04	95.3
Glucose Metabolism	TG:HDL	rs7157785	14	64235556	T	G	0.027	0.003	5.7e-10	1.4e-04	58.3
Glucose Metabolism	TG:HDL	rs17126047	14	90033046	C	T	-0.018	0.003	3.7e-08	1.1e-04	46.0
Glucose Metabolism	TG:HDL	rs61993685	14	100765823	C	T	-0.038	0.005	1.3e-10	1.6e-04	62.7
Glucose Metabolism	TG:HDL	rs45490496	14	105272678	T	A	0.019	0.003	6.6e-09	1.3e-04	51.1
Glucose Metabolism	TG:HDL	rs275182	15	39446211	G	A	-0.024	0.004	4.3e-08	1.1e-04	45.6
Glucose Metabolism	TG:HDL	rs7167078	15	41972392	G	C	-0.025	0.003	7.2e-14	2.1e-04	85.1
Glucose Metabolism	TG:HDL	rs184334219	15	42721845	A	G	0.114	0.009	8.6e-23	3.6e-04	146.7
Glucose Metabolism	TG:HDL	rs139974673	15	44027885	C	T	0.197	0.008	2.5e-88	1.5e-03	602.9
Glucose Metabolism	TG:HDL	rs1657926	15	57199592	C	T	-0.021	0.003	1.2e-08	1.2e-04	49.4
Glucose Metabolism	TG:HDL	rs1601935	15	58671765	T	G	0.025	0.003	8.0e-14	2.1e-04	84.8
Glucose Metabolism	TG:HDL	rs12440800	15	61960302	T	A	0.021	0.003	5.7e-09	1.3e-04	51.5
Glucose Metabolism	TG:HDL	rs7170462	15	63345622	A	G	0.034	0.003	1.0e-17	2.8e-04	111.5
Glucose Metabolism	TG:HDL	rs62012774	15	63868811	T	C	0.028	0.003	1.2e-10	1.6e-04	63.1
Glucose Metabolism	TG:HDL	rs4776793	15	66872114	T	C	0.022	0.003	2.0e-11	1.7e-04	68.3
Glucose Metabolism	TG:HDL	rs12924608	16	950007	C	T	-0.022	0.003	5.0e-12	1.8e-04	72.4
Glucose Metabolism	TG:HDL	rs1684608	16	4676852	A	C	0.022	0.003	3.7e-08	1.1e-04	46.1
Glucose Metabolism	TG:HDL	rs12928099	16	15150505	A	C	-0.039	0.003	1.0e-29	4.8e-04	194.7
Glucose Metabolism	TG:HDL	rs3814883	16	29994922	T	C	0.025	0.003	2.5e-15	2.4e-04	95.2
Glucose Metabolism	TG:HDL	rs10871454	16	31048079	T	C	-0.019	0.003	3.9e-09	1.3e-04	52.7
Glucose Metabolism	TG:HDL	rs11863743	16	56633321	T	C	-0.035	0.004	1.0e-11	1.7e-04	70.3
Glucose Metabolism	TG:HDL	rs34056275	16	56772710	A	G	-0.229	0.033	2.5e-08	1.2e-04	47.1
Glucose Metabolism	TG:HDL	rs563558487	16	56920865	A	G	-0.128	0.017	2.0e-09	1.4e-04	54.6
Glucose Metabolism	TG:HDL	rs144536013	16	56940341	T	G	0.215	0.027	1.1e-10	1.6e-04	63.1
Glucose Metabolism	TG:HDL	rs1138429	16	56942921	T	A	0.066	0.004	2.1e-40	6.7e-04	268.9
Glucose Metabolism	TG:HDL	rs72786786	16	56985514	A	G	-0.151	0.003	1.0e-200	7.2e-03	2928.1
Glucose Metabolism	TG:HDL	rs187468344	16	57007040	T	C	0.118	0.017	3.9e-08	1.1e-04	45.9
Glucose Metabolism	TG:HDL	rs562924383	16	57014640	T	C	-0.163	0.022	1.4e-09	1.4e-04	55.6
Glucose Metabolism	TG:HDL	rs185197208	16	57055635	A	G	0.183	0.017	1.1e-18	2.9e-04	118.1
Glucose Metabolism	TG:HDL	rs4783968	16	57072004	A	G	0.026	0.003	4.8e-14	2.1e-04	86.3
Glucose Metabolism	TG:HDL	rs113563886	16	67947158	C	T	-0.048	0.004	1.5e-22	3.6e-04	145.0

Phenotype	Trait	SNP	Chr	Position (GRCh37/hg19)	EA	NEA	BETA	SE	P-value	R2	F
Glucose Metabolism	TG:HDL	rs862320	16	69651866	T	C	-0.022	0.003	1.4e-11	1.7e-04	69.3
Glucose Metabolism	TG:HDL	rs12924886	16	72075593	T	A	0.032	0.003	7.7e-16	2.5e-04	98.6
Glucose Metabolism	TG:HDL	rs66710707	16	81528621	G	A	-0.036	0.003	1.7e-24	3.9e-04	158.5
Glucose Metabolism	TG:HDL	rs79311290	16	85150163	G	A	0.037	0.004	5.4e-12	1.8e-04	72.2
Glucose Metabolism	TG:HDL	rs12927305	16	88006413	T	C	0.022	0.003	8.0e-12	1.8e-04	71.0
Glucose Metabolism	TG:HDL	rs34345377	17	489296	G	C	0.024	0.003	1.9e-09	1.4e-04	54.8
Glucose Metabolism	TG:HDL	rs200489612	17	7106378	A	G	-0.172	0.019	2.0e-13	2.0e-04	82.0
Glucose Metabolism	TG:HDL	rs11651783	17	7447910	T	C	0.026	0.003	5.8e-12	1.8e-04	72.0
Glucose Metabolism	TG:HDL	rs13342397	17	17460926	C	T	0.054	0.005	1.8e-16	2.6e-04	103.0
Glucose Metabolism	TG:HDL	rs11658786	17	37815899	A	G	-0.020	0.003	8.4e-10	1.4e-04	57.2
Glucose Metabolism	TG:HDL	rs12945575	17	40713071	T	C	0.031	0.003	1.6e-17	2.7e-04	110.2
Glucose Metabolism	TG:HDL	rs62078657	17	41896541	A	G	-0.030	0.004	1.5e-10	1.5e-04	62.2
Glucose Metabolism	TG:HDL	rs72836561	17	41926126	T	C	0.203	0.007	2.0e-112	1.9e-03	771.1
Glucose Metabolism	TG:HDL	rs552316287	17	41977548	C	T	0.324	0.028	5.2e-21	3.3e-04	134.3
Glucose Metabolism	TG:HDL	rs74456742	17	42191796	A	G	-0.068	0.007	8.2e-16	2.4e-04	98.5
Glucose Metabolism	TG:HDL	rs17618704	17	46221010	G	T	-0.039	0.005	3.2e-10	1.5e-04	60.1
Glucose Metabolism	TG:HDL	rs4399576	17	47009835	A	G	0.022	0.003	1.7e-12	1.9e-04	75.7
Glucose Metabolism	TG:HDL	rs12943264	17	47418698	C	T	0.022	0.003	5.1e-12	1.8e-04	72.4
Glucose Metabolism	TG:HDL	rs8178824	17	64224775	T	C	-0.059	0.007	1.3e-10	1.6e-04	62.7
Glucose Metabolism	TG:HDL	rs62084237	17	65854807	A	G	0.035	0.003	2.2e-17	2.7e-04	109.3
Glucose Metabolism	TG:HDL	rs112001035	17	66823805	A	G	0.050	0.005	5.2e-14	2.1e-04	86.0
Glucose Metabolism	TG:HDL	rs766752	17	68424292	A	G	-0.017	0.003	4.0e-08	1.1e-04	45.8
Glucose Metabolism	TG:HDL	rs56257305	17	74207943	G	A	0.037	0.005	3.7e-10	1.5e-04	59.7
Glucose Metabolism	TG:HDL	rs11077363	17	76397309	T	C	-0.028	0.003	6.3e-18	2.8e-04	113.0
Glucose Metabolism	TG:HDL	rs67429374	18	100812	G	A	-0.020	0.003	1.7e-08	1.2e-04	48.3
Glucose Metabolism	TG:HDL	rs8088001	18	287705	G	T	-0.040	0.005	5.4e-11	1.6e-04	65.3
Glucose Metabolism	TG:HDL	rs35321365	18	19905141	T	C	-0.021	0.003	5.1e-11	1.6e-04	65.5
Glucose Metabolism	TG:HDL	rs149615216	18	47106028	T	C	-0.093	0.012	9.1e-10	1.4e-04	57.0
Glucose Metabolism	TG:HDL	rs2187375	18	47172283	G	A	-0.036	0.003	1.3e-18	2.9e-04	117.9
Glucose Metabolism	TG:HDL	rs11874381	18	47203408	A	G	-0.018	0.003	3.1e-08	1.2e-04	46.5
Glucose Metabolism	TG:HDL	rs41292412	18	56118358	T	C	0.104	0.012	8.4e-13	1.9e-04	77.7
Glucose Metabolism	TG:HDL	rs11664369	18	57739072	T	C	0.029	0.003	5.4e-16	2.5e-04	99.7
Glucose Metabolism	TG:HDL	rs78274649	18	58053794	A	G	-0.067	0.009	2.2e-09	1.3e-04	54.3
Glucose Metabolism	TG:HDL	rs1799816	19	7125518	T	C	-0.129	0.015	1.1e-11	1.7e-04	70.1
Glucose Metabolism	TG:HDL	rs12608575	19	7201504	C	T	-0.027	0.003	1.6e-14	2.2e-04	89.5
Glucose Metabolism	TG:HDL	rs62112763	19	7220013	G	C	0.030	0.003	1.9e-21	3.4e-04	137.4
Glucose Metabolism	TG:HDL	rs2115108	19	7963976	C	T	-0.020	0.003	1.0e-09	1.4e-04	56.6
Glucose Metabolism	TG:HDL	rs117188729	19	8415076	A	T	-0.082	0.007	2.4e-19	3.1e-04	122.8
Glucose Metabolism	TG:HDL	rs116843064	19	8429323	A	G	-0.260	0.009	5.6e-116	2.0e-03	795.9
Glucose Metabolism	TG:HDL	rs111831594	19	11294359	G	A	0.038	0.005	1.1e-11	1.7e-04	70.2
Glucose Metabolism	TG:HDL	rs188247550	19	19396616	T	C	-0.135	0.012	4.6e-21	3.3e-04	134.7
Glucose Metabolism	TG:HDL	rs10401969	19	19407718	C	T	-0.101	0.005	2.4e-65	1.1e-03	442.6
Glucose Metabolism	TG:HDL	rs10426801	19	19849201	T	A	-0.040	0.005	2.6e-09	1.3e-04	53.9
Glucose Metabolism	TG:HDL	rs62102718	19	33891013	T	A	0.030	0.003	6.3e-18	2.8e-04	113.1
Glucose Metabolism	TG:HDL	rs45512696	19	35550878	T	C	0.029	0.003	5.6e-12	1.8e-04	72.1
Glucose Metabolism	TG:HDL	rs4760	19	44153100	G	A	0.026	0.004	3.2e-09	1.3e-04	53.2
Glucose Metabolism	TG:HDL	rs147297605	19	45111582	C	G	-0.367	0.034	3.8e-18	2.8e-04	114.6
Glucose Metabolism	TG:HDL	rs140824606	19	45311084	A	G	0.060	0.009	2.2e-08	1.2e-04	47.6
Glucose Metabolism	TG:HDL	rs4081918	19	45373739	G	A	-0.042	0.004	7.2e-15	2.3e-04	92.0
Glucose Metabolism	TG:HDL	rs483082	19	45416178	T	G	0.109	0.003	4.6e-192	3.3e-03	1327.3
Glucose Metabolism	TG:HDL	rs187408586	19	45422921	G	A	0.128	0.017	9.8e-10	1.4e-04	56.8
Glucose Metabolism	TG:HDL	rs34934360	19	46398989	G	A	0.034	0.004	5.2e-12	1.8e-04	72.3
Glucose Metabolism	TG:HDL	rs12150914	19	47563418	C	T	0.020	0.003	1.3e-09	1.4e-04	55.9
Glucose Metabolism	TG:HDL	rs4002471	19	49215095	T	C	0.020	0.003	1.2e-10	1.6e-04	63.0
Glucose Metabolism	TG:HDL	rs434124	19	54809336	G	C	-0.030	0.003	5.3e-16	2.5e-04	99.8
Glucose Metabolism	TG:HDL	rs12610709	19	56102362	A	G	0.029	0.003	3.3e-12	1.8e-04	73.6
Glucose Metabolism	TG:HDL	rs11670474	19	57488280	G	A	0.018	0.003	1.4e-08	1.2e-04	48.8
Glucose Metabolism	TG:HDL	rs144033177	20	571467	C	A	0.080	0.010	4.2e-10	1.5e-04	59.2
Glucose Metabolism	TG:HDL	rs6088461	20	32905161	T	G	0.019	0.003	4.4e-09	1.3e-04	52.3
Glucose Metabolism	TG:HDL	rs2207132	20	39142516	A	G	0.050	0.007	1.2e-08	1.2e-04	49.4
Glucose Metabolism	TG:HDL	rs1800961	20	43042364	T	C	0.066	0.007	2.7e-13	2.0e-04	81.1
Glucose Metabolism	TG:HDL	rs6073958	20	44551855	C	T	0.094	0.003	2.3e-125	2.1e-03	861.5

Phenotype	Trait	SNP	Chr	Position (GRCh37/hg19)	EA	NEA	BETA	SE	P-value	R2	F
Glucose Metabolism	TG:HDL	rs6104456	20	44718231	A	G	-0.019	0.003	3.0e-09	1.3e-04	53.4
Glucose Metabolism	TG:HDL	rs55966194	20	45599090	G	C	-0.024	0.003	4.5e-12	1.8e-04	72.8
Glucose Metabolism	TG:HDL	rs865034	20	51261615	C	T	0.019	0.003	1.5e-08	1.2e-04	48.6
Glucose Metabolism	TG:HDL	rs6123685	20	55836040	A	G	-0.023	0.003	9.6e-11	1.6e-04	63.6
Glucose Metabolism	TG:HDL	rs2039098	20	56112882	T	C	0.019	0.003	2.4e-09	1.3e-04	54.0
Glucose Metabolism	TG:HDL	rs185799410	20	57466093	T	G	0.056	0.008	2.6e-08	1.2e-04	47.1
Glucose Metabolism	TG:HDL	rs8126001	20	62711459	T	C	-0.020	0.003	1.1e-10	1.6e-04	63.3
Glucose Metabolism	TG:HDL	rs2223041	21	16422603	T	C	0.018	0.003	5.0e-08	1.1e-04	45.1
Glucose Metabolism	TG:HDL	rs4820324	22	38599857	C	G	0.032	0.003	2.7e-24	3.9e-04	157.1
Glucose Metabolism	TG:HDL	rs2071887	22	38879010	A	T	0.026	0.003	9.2e-15	2.3e-04	91.2
Adiposity Markers	BMI	rs2803298	1	1846073	T	G	0.012	0.002	2.6e-15	6.7e-05	61.9
Adiposity Markers	BMI	rs1460943	1	72813129	T	C	0.026	0.001	8.1e-77	3.5e-04	358.3
Adiposity Markers	BMI	rs13303360	1	203488344	A	G	0.014	0.002	8.1e-11	5.2e-05	41.3
Adiposity Markers	BMI	rs7551794	1	97397047	A	T	-0.014	0.002	1.8e-20	9.2e-05	85.9
Adiposity Markers	BMI	rs6688826	1	80812329	T	C	-0.010	0.002	6.8e-10	4.0e-05	37.5
Adiposity Markers	BMI	rs4950078	1	96257675	T	G	-0.015	0.002	2.1e-18	8.1e-05	74.8
Adiposity Markers	BMI	rs16826068	1	39791876	C	G	0.019	0.002	1.4e-20	9.4e-05	87.4
Adiposity Markers	BMI	rs645482	1	191386639	A	G	0.009	0.002	1.7e-08	3.3e-05	30.6
Adiposity Markers	BMI	rs11577094	1	38026600	T	C	0.018	0.003	4.6e-12	4.9e-05	49.5
Adiposity Markers	BMI	rs1937443	1	66469643	C	G	-0.010	0.002	1.2e-11	4.9e-05	46.2
Adiposity Markers	BMI	rs6700816	1	174883994	A	T	0.029	0.004	3.7e-11	4.7e-05	43.7
Adiposity Markers	BMI	rs11588897	1	177392910	T	C	0.017	0.003	7.9e-11	4.4e-05	41.3
Adiposity Markers	BMI	rs12146022	1	171451959	A	T	-0.012	0.002	9.5e-10	4.1e-05	38.4
Adiposity Markers	BMI	rs7550711	1	110082886	T	C	0.061	0.004	2.4e-43	1.9e-04	190.3
Adiposity Markers	BMI	rs3922650	1	177766694	A	C	0.009	0.001	8.8e-11	4.3e-05	44.1
Adiposity Markers	BMI	rs2791149	1	163579955	T	C	-0.011	0.002	4.8e-08	3.8e-05	28.6
Adiposity Markers	BMI	rs2984618	1	47690438	T	G	0.017	0.001	9.1e-32	1.4e-04	142.3
Adiposity Markers	BMI	rs6661316	1	210095527	T	C	0.013	0.001	1.7e-18	7.8e-05	79.7
Adiposity Markers	BMI	rs7535528	1	2444414	A	G	-0.015	0.002	2.9e-21	9.9e-05	87.9
Adiposity Markers	BMI	rs880554	1	19954014	T	G	-0.010	0.002	4.7e-09	3.9e-05	36.0
Adiposity Markers	BMI	rs12033257	1	112318484	A	G	0.014	0.002	5.7e-20	8.7e-05	79.9
Adiposity Markers	BMI	rs11578857	1	34285381	T	C	-0.008	0.002	1.9e-08	3.3e-05	30.6
Adiposity Markers	BMI	rs4593875	1	50591851	A	G	0.016	0.002	1.8e-19	8.4e-05	78.0
Adiposity Markers	BMI	rs518785	1	111359802	A	G	0.010	0.002	3.0e-08	3.2e-05	29.6
Adiposity Markers	BMI	rs7526754	1	209233764	T	C	-0.010	0.002	2.8e-10	4.3e-05	39.8
Adiposity Markers	BMI	rs3766430	1	54730651	T	C	-0.010	0.001	2.2e-13	5.4e-05	55.2
Adiposity Markers	BMI	rs997474	1	201645609	C	G	0.013	0.002	5.6e-09	3.8e-05	34.9
Adiposity Markers	BMI	rs657452	1	49589847	A	G	0.018	0.002	4.4e-34	1.4e-04	139.2
Adiposity Markers	BMI	rs11120813	1	7062993	A	G	0.009	0.002	3.1e-08	3.2e-05	29.6
Adiposity Markers	BMI	rs7520706	1	101751884	T	G	0.011	0.002	1.8e-08	3.5e-05	32.3
Adiposity Markers	BMI	rs16864515	1	171435542	A	C	0.017	0.003	1.0e-11	4.8e-05	44.8
Adiposity Markers	BMI	rs1874129	1	235700023	T	G	-0.014	0.002	1.3e-10	4.5e-05	41.1
Adiposity Markers	BMI	rs946824	1	243684019	T	C	0.018	0.002	6.2e-16	6.9e-05	64.7
Adiposity Markers	BMI	rs4971079	1	155130391	A	G	-0.009	0.001	2.4e-09	3.6e-05	36.9
Adiposity Markers	BMI	rs12564992	1	174478100	A	G	-0.019	0.002	1.3e-16	6.8e-05	69.7
Adiposity Markers	BMI	rs11165643	1	96924097	T	C	0.018	0.001	6.9e-37	1.6e-04	167.1
Adiposity Markers	BMI	rs17014370	1	209540091	A	G	0.015	0.002	6.0e-12	5.0e-05	47.1
Adiposity Markers	BMI	rs7549758	1	34935035	T	C	-0.011	0.002	4.7e-10	4.3e-05	40.1
Adiposity Markers	BMI	rs11118308	1	219633869	A	G	0.011	0.001	8.1e-14	5.6e-05	57.3
Adiposity Markers	BMI	rs11208660	1	65983626	T	C	0.021	0.003	2.9e-16	6.5e-05	66.5
Adiposity Markers	BMI	rs4920608	1	17319287	T	C	-0.011	0.002	1.4e-11	5.0e-05	43.9
Adiposity Markers	BMI	rs11802147	1	108001307	A	G	0.013	0.002	5.8e-16	7.0e-05	65.0
Adiposity Markers	BMI	rs4653942	1	228505204	A	G	-0.011	0.002	3.2e-09	3.3e-05	34.0
Adiposity Markers	BMI	rs12040651	1	29551744	A	T	0.016	0.003	3.9e-09	3.6e-05	33.4
Adiposity Markers	BMI	rs2275426	1	46487552	A	G	0.010	0.001	2.4e-13	5.4e-05	55.2
Adiposity Markers	BMI	rs10920678	1	190239907	A	G	0.017	0.001	6.8e-32	1.4e-04	144.0
Adiposity Markers	BMI	rs2791643	1	11207269	T	C	-0.013	0.002	1.3e-13	5.8e-05	54.1
Adiposity Markers	BMI	rs4357530	1	151103153	A	G	-0.013	0.002	1.6e-17	7.2e-05	74.0
Adiposity Markers	BMI	rs823075	1	205774897	T	C	-0.010	0.002	1.5e-10	4.4e-05	41.0
Adiposity Markers	BMI	rs2235549	1	23391775	T	G	-0.018	0.002	7.2e-21	9.8e-05	91.8
Adiposity Markers	BMI	rs4653017	1	33776728	T	C	0.012	0.002	2.3e-13	5.7e-05	53.5
Adiposity Markers	BMI	rs543874	1	177889480	A	G	-0.045	0.002	7.8e-141	6.1e-04	619.5

Phenotype	Trait	SNP	Chr	Position (GRCh37/hg19)	EA	NEA	BETA	SE	P-value	R2	F
Adiposity Markers	BMI	rs4844686	1	208701114	T	C	-0.008	0.002	3.8e-08	3.3e-05	30.6
Adiposity Markers	BMI	rs2615075	1	225934295	A	G	-0.009	0.002	1.5e-08	3.3e-05	30.9
Adiposity Markers	BMI	rs8024	1	201845575	A	C	0.022	0.002	1.4e-45	2.1e-04	197.8
Adiposity Markers	BMI	rs17011146	1	222113648	A	G	0.010	0.002	4.9e-08	3.0e-05	28.3
Adiposity Markers	BMI	rs2401138	1	8704059	C	G	0.011	0.002	3.6e-13	5.7e-05	52.8
Adiposity Markers	BMI	rs9324379	1	98421965	A	C	-0.016	0.002	3.7e-15	6.8e-05	63.2
Adiposity Markers	BMI	rs11264483	1	156406381	C	G	0.014	0.002	9.4e-18	8.0e-05	73.3
Adiposity Markers	BMI	rs542941394	1	23372114	A	C	-1.420	0.246	8.3e-09	1.5e-04	33.2
Adiposity Markers	BMI	rs10801377	1	195028770	C	G	0.012	0.002	9.8e-16	6.9e-05	64.0
Adiposity Markers	BMI	rs7523668	1	42408159	A	G	-0.009	0.002	3.4e-10	4.2e-05	39.3
Adiposity Markers	BMI	rs6577584	1	6715390	T	G	-0.014	0.002	3.1e-18	7.8e-05	73.3
Adiposity Markers	BMI	rs7551758	1	52274078	T	G	0.008	0.002	3.4e-08	3.2e-05	29.9
Adiposity Markers	BMI	rs12021727	1	79371641	C	G	0.009	0.002	2.7e-08	3.3e-05	30.6
Adiposity Markers	BMI	rs2104404	1	156256913	A	C	0.010	0.002	1.9e-08	3.5e-05	32.7
Adiposity Markers	BMI	rs12066815	1	107883043	A	G	-0.016	0.002	1.1e-18	8.7e-05	81.0
Adiposity Markers	BMI	rs4970712	1	92993547	A	C	0.013	0.002	2.2e-13	5.1e-05	52.2
Adiposity Markers	BMI	rs10923724	1	119546842	T	C	-0.014	0.001	3.9e-22	9.5e-05	97.2
Adiposity Markers	BMI	rs4950824	1	202077489	A	T	-0.010	0.002	1.6e-10	4.3e-05	39.8
Adiposity Markers	BMI	rs2481665	1	62594677	T	C	0.014	0.001	2.7e-24	1.0e-04	105.8
Adiposity Markers	BMI	rs1891215	1	7727854	T	C	-0.012	0.002	1.5e-14	6.3e-05	58.8
Adiposity Markers	BMI	rs1439524	1	242531619	A	G	-0.013	0.002	1.9e-08	3.1e-05	31.9
Adiposity Markers	BMI	rs4258241	1	84437929	A	G	0.010	0.002	1.1e-09	4.7e-05	36.0
Adiposity Markers	BMI	rs10873947	1	77986516	A	G	-0.013	0.001	4.8e-19	8.0e-05	82.3
Adiposity Markers	BMI	rs10913118	1	175954755	A	C	-0.010	0.002	2.3e-11	4.6e-05	47.2
Adiposity Markers	BMI	rs870855	1	80009414	T	G	-0.008	0.002	4.1e-08	3.3e-05	30.6
Adiposity Markers	BMI	rs905938	1	154991389	T	C	-0.013	0.002	1.6e-15	6.0e-05	61.2
Adiposity Markers	BMI	rs1203632	1	14087114	A	C	0.011	0.002	3.3e-10	4.1e-05	38.1
Adiposity Markers	BMI	rs3010840	1	221954870	A	C	-0.008	0.001	1.1e-08	3.3e-05	33.5
Adiposity Markers	BMI	rs4568876	1	32152518	T	C	0.011	0.002	7.9e-14	6.1e-05	56.8
Adiposity Markers	BMI	rs12035349	1	77557339	A	G	-0.019	0.002	5.2e-16	7.1e-05	65.4
Adiposity Markers	BMI	rs17424278	1	57846394	A	C	-0.015	0.002	5.2e-10	4.3e-05	40.1
Adiposity Markers	BMI	rs11579790	1	154279183	A	C	0.013	0.002	1.4e-08	3.3e-05	33.3
Adiposity Markers	BMI	rs6593668	1	96033966	T	C	-0.012	0.002	7.2e-10	4.1e-05	38.4
Adiposity Markers	BMI	rs11208428	1	64809520	T	G	-0.009	0.002	4.3e-10	4.1e-05	38.4
Adiposity Markers	BMI	rs1730851	1	107596443	A	G	0.013	0.002	9.2e-16	6.7e-05	63.0
Adiposity Markers	BMI	rs2404715	1	57008778	T	C	0.015	0.002	5.7e-09	3.4e-05	34.6
Adiposity Markers	BMI	rs2024766	1	184653633	T	C	-0.008	0.002	1.7e-08	3.4e-05	31.4
Adiposity Markers	BMI	rs17096289	1	75592165	A	G	0.009	0.002	1.8e-08	3.4e-05	32.1
Adiposity Markers	BMI	rs3789586	1	51054322	T	C	0.013	0.002	4.5e-11	4.8e-05	44.9
Adiposity Markers	BMI	rs1497675	1	104820780	T	C	0.008	0.002	3.6e-08	3.2e-05	29.9
Adiposity Markers	BMI	rs12749978	1	91129376	T	C	0.015	0.003	4.0e-09	3.7e-05	34.2
Adiposity Markers	BMI	rs10489526	1	115293527	A	C	0.011	0.002	4.5e-10	4.3e-05	40.1
Adiposity Markers	BMI	rs17391694	1	78623626	T	C	0.032	0.002	1.8e-49	2.2e-04	218.2
Adiposity Markers	BMI	rs4639733	1	166038967	A	G	0.008	0.002	3.2e-08	3.2e-05	29.9
Adiposity Markers	BMI	rs284227	1	82379446	T	C	-0.013	0.002	2.8e-15	6.0e-05	61.2
Adiposity Markers	BMI	rs10803392	1	15876758	T	C	-0.010	0.002	1.3e-10	4.4e-05	40.6
Adiposity Markers	BMI	rs12566985	1	75002193	A	G	-0.017	0.001	6.5e-33	1.4e-04	147.4
Adiposity Markers	BMI	rs2000	1	173457834	A	G	-0.026	0.004	9.6e-09	3.6e-05	33.1
Adiposity Markers	BMI	rs1009188	1	197257090	T	C	0.013	0.002	9.4e-16	6.6e-05	62.1
Adiposity Markers	BMI	rs7534091	1	118864616	A	G	-0.012	0.002	8.2e-13	5.1e-05	51.7
Adiposity Markers	BMI	rs535533	2	166959505	T	C	-0.009	0.002	3.0e-09	3.8e-05	35.2
Adiposity Markers	BMI	rs4672221	2	57919517	A	T	-0.011	0.002	3.7e-12	6.0e-05	46.4
Adiposity Markers	BMI	rs10930502	2	172890588	A	G	0.013	0.002	2.3e-15	6.2e-05	63.0
Adiposity Markers	BMI	rs1551131	2	119536407	T	C	0.009	0.002	2.2e-09	3.9e-05	36.0
Adiposity Markers	BMI	rs12990552	2	29733405	T	C	-0.009	0.002	4.1e-09	3.6e-05	33.8
Adiposity Markers	BMI	rs10204807	2	23810290	A	G	-0.011	0.002	5.4e-10	4.0e-05	37.3
Adiposity Markers	BMI	rs972112	2	50828633	A	C	-0.009	0.002	3.3e-08	3.1e-05	29.3
Adiposity Markers	BMI	rs2111877	2	159388014	T	C	-0.013	0.002	8.3e-10	4.0e-05	37.7
Adiposity Markers	BMI	rs2192771	2	40509154	T	C	-0.010	0.002	1.4e-08	3.3e-05	31.2
Adiposity Markers	BMI	rs12622653	2	99546355	A	G	-0.010	0.002	1.4e-08	3.5e-05	32.1
Adiposity Markers	BMI	rs12615742	2	37995727	T	C	-0.009	0.002	5.5e-09	3.3e-05	33.6
Adiposity Markers	BMI	rs3821031	2	219295249	T	C	0.012	0.002	1.4e-14	6.4e-05	59.8

Phenotype	Trait	SNP	Chr	Position (GRCh37/hg19)	EA	NEA	BETA	SE	P-value	R2	F
Adiposity Markers	BMI	rs4482463	2	205375909	A	C	-0.028	0.003	7.5e-25	1.2e-04	107.5
Adiposity Markers	BMI	rs17551974	2	142293146	A	C	-0.013	0.002	1.4e-11	5.1e-05	47.5
Adiposity Markers	BMI	rs12692738	2	165558252	T	C	-0.013	0.002	7.2e-15	5.9e-05	60.3
Adiposity Markers	BMI	rs7566185	2	60091601	A	G	-0.009	0.002	1.9e-08	3.2e-05	29.9
Adiposity Markers	BMI	rs4671328	2	58935282	T	G	0.022	0.002	1.6e-48	2.3e-04	213.2
Adiposity Markers	BMI	rs3771076	2	188277123	A	T	-0.009	0.002	2.1e-09	3.9e-05	36.0
Adiposity Markers	BMI	rs4480977	2	215405343	T	G	0.010	0.001	1.8e-11	4.6e-05	47.0
Adiposity Markers	BMI	rs7601895	2	55281901	C	G	0.015	0.002	1.8e-20	9.3e-05	86.7
Adiposity Markers	BMI	rs7578902	2	50707394	A	G	0.012	0.002	1.1e-15	6.8e-05	62.9
Adiposity Markers	BMI	rs7583473	2	58101921	T	C	0.015	0.003	3.2e-08	3.3e-05	31.3
Adiposity Markers	BMI	rs501964	2	45138941	T	C	-0.021	0.003	1.6e-10	1.9e-04	40.1
Adiposity Markers	BMI	rs1227929	2	162720101	T	G	-0.009	0.002	3.6e-09	3.8e-05	35.2
Adiposity Markers	BMI	rs6705916	2	135397569	T	C	0.008	0.002	2.5e-08	3.3e-05	30.6
Adiposity Markers	BMI	rs1453118	2	236896678	T	C	0.011	0.002	2.2e-12	5.3e-05	49.0
Adiposity Markers	BMI	rs11885913	2	209198000	T	C	-0.013	0.002	4.1e-08	3.1e-05	28.9
Adiposity Markers	BMI	rs7580766	2	42939351	A	G	-0.010	0.002	1.0e-11	4.9e-05	45.3
Adiposity Markers	BMI	rs12611517	2	228977034	A	T	0.013	0.002	2.4e-17	7.5e-05	70.1
Adiposity Markers	BMI	rs17041983	2	546610	T	C	0.029	0.003	1.5e-26	1.2e-04	114.6
Adiposity Markers	BMI	rs980329	2	60285100	T	C	-0.015	0.002	8.1e-17	7.8e-05	72.8
Adiposity Markers	BMI	rs17327461	2	35512183	T	C	0.011	0.001	1.4e-14	6.0e-05	61.7
Adiposity Markers	BMI	rs262255	2	180903586	T	C	0.009	0.002	3.5e-10	4.2e-05	39.3
Adiposity Markers	BMI	rs10497870	2	203970283	A	G	0.012	0.001	6.4e-16	6.6e-05	67.5
Adiposity Markers	BMI	rs4850962	2	101801651	T	C	0.009	0.001	6.2e-10	4.0e-05	40.4
Adiposity Markers	BMI	rs4372836	2	28973883	T	C	0.013	0.002	1.4e-16	6.8e-05	69.4
Adiposity Markers	BMI	rs2044469	2	174961488	A	G	-0.012	0.002	2.7e-13	5.8e-05	54.4
Adiposity Markers	BMI	rs6739199	2	105452326	T	C	0.016	0.002	1.3e-22	9.8e-05	91.9
Adiposity Markers	BMI	rs698838	2	44738763	T	C	0.011	0.002	1.2e-11	4.8e-05	43.9
Adiposity Markers	BMI	rs13396415	2	142831758	A	C	-0.012	0.002	3.4e-09	3.8e-05	35.4
Adiposity Markers	BMI	rs1421609	2	15841288	A	G	-0.010	0.002	1.7e-09	3.8e-05	35.3
Adiposity Markers	BMI	rs12712767	2	41703383	A	G	0.010	0.002	1.2e-11	5.1e-05	47.2
Adiposity Markers	BMI	rs17437781	2	165424837	A	G	0.008	0.002	2.2e-08	3.4e-05	31.4
Adiposity Markers	BMI	rs6543021	2	98318644	A	G	0.016	0.003	1.2e-08	3.5e-05	32.7
Adiposity Markers	BMI	rs7607465	2	131955247	C	G	0.013	0.002	1.6e-08	3.6e-05	32.8
Adiposity Markers	BMI	rs13432055	2	56603985	T	C	-0.010	0.002	1.2e-09	3.6e-05	36.8
Adiposity Markers	BMI	rs7567655	2	28273595	A	G	-0.025	0.004	1.3e-10	4.0e-05	40.8
Adiposity Markers	BMI	rs10929925	2	6155557	A	C	-0.014	0.001	1.2e-23	1.0e-04	104.3
Adiposity Markers	BMI	rs12692596	2	161265910	T	C	0.010	0.002	5.3e-11	4.3e-05	43.6
Adiposity Markers	BMI	rs6722981	2	63916722	T	C	-0.009	0.002	1.8e-08	3.4e-05	31.6
Adiposity Markers	BMI	rs17247324	2	2195519	A	G	-0.013	0.002	3.9e-10	4.3e-05	40.3
Adiposity Markers	BMI	rs11902450	2	12845368	T	C	0.017	0.002	4.3e-13	5.5e-05	51.4
Adiposity Markers	BMI	rs972540	2	207244783	A	G	-0.013	0.002	3.1e-16	6.6e-05	67.0
Adiposity Markers	BMI	rs17041868	2	111894720	T	C	0.017	0.003	4.2e-09	3.4e-05	35.2
Adiposity Markers	BMI	rs4954647	2	139859202	T	G	0.008	0.002	3.4e-08	3.3e-05	30.6
Adiposity Markers	BMI	rs17734264	2	24719710	T	C	-0.019	0.002	7.5e-28	1.2e-04	115.0
Adiposity Markers	BMI	rs6542924	2	100893113	A	C	-0.016	0.002	1.6e-22	9.6e-05	89.7
Adiposity Markers	BMI	rs13417156	2	62848319	T	C	-0.012	0.002	2.3e-16	6.9e-05	68.3
Adiposity Markers	BMI	rs13396535	2	145223872	T	C	-0.012	0.002	4.1e-09	3.7e-05	34.9
Adiposity Markers	BMI	rs2119753	2	151224579	A	G	0.009	0.002	2.8e-09	3.9e-05	36.0
Adiposity Markers	BMI	rs535643789	2	25564215	C	G	-0.044	0.008	4.6e-08	1.4e-04	29.8
Adiposity Markers	BMI	rs1446585	2	136407479	A	G	0.013	0.002	1.2e-15	6.6e-05	61.0
Adiposity Markers	BMI	rs4851029	2	104159785	T	G	-0.011	0.002	1.9e-13	5.8e-05	53.8
Adiposity Markers	BMI	rs2203452	2	227094758	A	G	0.027	0.003	2.5e-17	3.3e-04	71.2
Adiposity Markers	BMI	rs10932099	2	206058350	T	C	0.009	0.002	6.1e-10	4.1e-05	38.4
Adiposity Markers	BMI	rs10172053	2	211644647	T	G	-0.016	0.002	6.5e-16	7.3e-05	68.3
Adiposity Markers	BMI	rs7421089	2	211988412	T	C	0.013	0.002	8.7e-15	6.1e-05	56.7
Adiposity Markers	BMI	rs11125450	2	53058194	A	C	-0.009	0.001	3.5e-09	3.6e-05	36.9
Adiposity Markers	BMI	rs10164798	2	69565345	T	C	0.010	0.002	2.2e-10	4.3e-05	40.1
Adiposity Markers	BMI	rs11676199	2	145626553	A	G	-0.019	0.003	1.5e-10	4.4e-05	41.1
Adiposity Markers	BMI	rs1083485	2	147950717	T	C	0.013	0.002	2.6e-16	8.4e-05	65.0
Adiposity Markers	BMI	rs2216931	2	181599070	A	C	0.017	0.002	7.8e-28	1.3e-04	116.9
Adiposity Markers	BMI	rs1011406	2	60666075	C	G	0.016	0.002	6.5e-12	5.0e-05	46.6
Adiposity Markers	BMI	rs4673613	2	212264642	T	C	-0.012	0.002	1.4e-14	6.2e-05	58.1

Phenotype	Trait	SNP	Chr	Position (GRCh37/hg19)	EA	NEA	BETA	SE	P-value	R2	F
Adiposity Markers	BMI	rs3770176	2	222317545	T	C	-0.010	0.002	1.3e-08	3.3e-05	30.9
Adiposity Markers	BMI	rs11676272	2	25141538	A	G	-0.029	0.001	4.0e-94	4.5e-04	435.0
Adiposity Markers	BMI	rs13400686	2	200289152	A	G	-0.012	0.002	2.1e-08	3.4e-05	32.1
Adiposity Markers	BMI	rs4639527	2	416815	A	G	-0.015	0.002	1.5e-21	9.5e-05	89.1
Adiposity Markers	BMI	rs930295	2	50233352	A	C	0.018	0.002	5.1e-20	9.2e-05	85.6
Adiposity Markers	BMI	rs6710871	2	143960593	A	G	0.020	0.002	7.3e-21	9.1e-05	88.0
Adiposity Markers	BMI	rs6744911	2	166208049	A	G	0.011	0.002	8.7e-11	4.4e-05	41.1
Adiposity Markers	BMI	rs4676084	2	110010962	A	G	0.010	0.002	5.5e-11	4.6e-05	42.7
Adiposity Markers	BMI	rs6436887	2	230589014	A	G	-0.012	0.002	3.0e-08	3.3e-05	31.3
Adiposity Markers	BMI	rs2539672	2	59877551	T	C	0.013	0.002	1.1e-12	5.2e-05	48.3
Adiposity Markers	BMI	rs17203016	2	208255518	A	G	-0.015	0.002	2.7e-16	6.6e-05	67.6
Adiposity Markers	BMI	rs1521886	2	202940099	T	C	0.009	0.002	2.3e-08	3.2e-05	29.6
Adiposity Markers	BMI	rs10193397	2	142538872	A	T	0.009	0.002	7.2e-09	3.6e-05	33.6
Adiposity Markers	BMI	rs2029085	2	187207393	A	G	-0.017	0.002	1.2e-12	5.4e-05	50.8
Adiposity Markers	BMI	rs12477088	2	67841326	T	C	0.015	0.002	1.0e-22	1.0e-04	97.4
Adiposity Markers	BMI	rs2346882	2	47015813	A	G	-0.012	0.002	2.0e-13	5.7e-05	53.5
Adiposity Markers	BMI	rs1263627	2	207977253	A	T	-0.012	0.002	2.6e-12	5.1e-05	47.5
Adiposity Markers	BMI	rs4954638	2	137435455	A	C	0.009	0.002	2.4e-08	3.2e-05	29.9
Adiposity Markers	BMI	rs731834	2	113957198	A	C	0.010	0.002	1.7e-11	5.2e-05	45.3
Adiposity Markers	BMI	rs17271838	2	232639176	T	C	-0.010	0.002	1.6e-09	3.5e-05	35.3
Adiposity Markers	BMI	rs12714199	2	86812549	T	C	-0.014	0.002	9.0e-22	9.7e-05	90.9
Adiposity Markers	BMI	rs1460678	2	164584156	C	G	-0.016	0.002	8.9e-11	4.5e-05	42.2
Adiposity Markers	BMI	rs6720868	2	230663576	T	C	0.013	0.002	1.4e-16	7.2e-05	67.0
Adiposity Markers	BMI	rs11884795	2	133519504	A	G	0.011	0.002	4.7e-11	4.4e-05	44.7
Adiposity Markers	BMI	rs16825786	2	198794681	A	C	-0.016	0.002	2.9e-18	8.6e-05	80.0
Adiposity Markers	BMI	rs7575118	2	182653725	T	C	0.012	0.002	1.2e-08	3.5e-05	32.1
Adiposity Markers	BMI	rs6750646	2	193872415	T	C	-0.011	0.002	1.5e-08	3.4e-05	31.7
Adiposity Markers	BMI	rs934515	2	79482643	A	G	0.015	0.002	2.8e-11	4.7e-05	44.3
Adiposity Markers	BMI	rs2901767	2	65680641	T	C	-0.015	0.003	1.9e-08	3.5e-05	32.0
Adiposity Markers	BMI	rs7599312	2	213413231	A	G	-0.019	0.002	8.2e-31	1.3e-04	136.6
Adiposity Markers	BMI	rs10931158	2	185809565	T	C	0.018	0.003	2.0e-08	1.5e-04	31.3
Adiposity Markers	BMI	rs7576796	2	236770736	A	G	-0.013	0.002	6.7e-14	5.9e-05	54.6
Adiposity Markers	BMI	rs805330	2	54148331	A	C	-0.009	0.002	2.1e-09	3.8e-05	35.2
Adiposity Markers	BMI	rs6713781	2	40291940	C	G	-0.011	0.002	3.0e-13	5.8e-05	53.8
Adiposity Markers	BMI	rs1658807	2	200767512	T	C	0.013	0.002	4.7e-12	4.9e-05	45.4
Adiposity Markers	BMI	rs11695013	2	157057487	T	C	-0.011	0.002	3.9e-13	5.7e-05	52.8
Adiposity Markers	BMI	rs3770890	2	36657992	T	G	-0.029	0.005	1.4e-09	3.7e-05	36.5
Adiposity Markers	BMI	rs11677466	2	232982257	A	T	0.015	0.003	6.6e-09	3.3e-05	32.8
Adiposity Markers	BMI	rs6547385	2	81948355	T	C	-0.010	0.002	5.5e-10	4.0e-05	37.5
Adiposity Markers	BMI	rs10933597	2	241346265	A	T	0.010	0.002	1.1e-10	4.3e-05	40.6
Adiposity Markers	BMI	rs12615199	2	51825253	A	T	0.011	0.002	4.1e-09	3.5e-05	32.9
Adiposity Markers	BMI	rs908194	2	220198247	A	G	0.017	0.002	2.6e-17	7.8e-05	73.1
Adiposity Markers	BMI	rs7561278	2	48954905	T	C	0.017	0.002	1.5e-20	9.8e-05	91.3
Adiposity Markers	BMI	rs6433242	2	171593276	C	G	0.009	0.002	2.8e-08	3.2e-05	29.6
Adiposity Markers	BMI	rs6545714	2	59307725	A	G	-0.019	0.001	1.1e-39	1.8e-04	180.3
Adiposity Markers	BMI	rs12468863	2	26940294	T	C	-0.014	0.001	2.4e-21	9.2e-05	94.4
Adiposity Markers	BMI	rs6734363	2	635200	A	G	0.056	0.002	1.3e-197	8.5e-04	865.6
Adiposity Markers	BMI	rs2366643	3	159736485	T	C	-0.010	0.002	1.4e-11	4.9e-05	45.3
Adiposity Markers	BMI	rs514816	3	161478145	A	G	0.011	0.002	1.3e-11	5.6e-05	43.1
Adiposity Markers	BMI	rs4856831	3	66957468	C	G	-0.014	0.002	6.7e-10	4.1e-05	38.7
Adiposity Markers	BMI	rs500216	3	193598283	A	G	-0.010	0.002	3.8e-10	4.0e-05	37.5
Adiposity Markers	BMI	rs12491006	3	16812479	A	T	0.017	0.003	1.4e-08	3.8e-05	33.2
Adiposity Markers	BMI	rs2710323	3	52815905	T	C	-0.015	0.001	2.4e-25	1.1e-04	111.8
Adiposity Markers	BMI	rs7646851	3	60906362	T	G	0.011	0.002	6.7e-09	3.7e-05	34.7
Adiposity Markers	BMI	rs2569993	3	12926096	T	C	-0.010	0.002	8.7e-11	4.4e-05	41.4
Adiposity Markers	BMI	rs33503	3	42427957	A	G	-0.016	0.002	2.9e-18	8.5e-05	79.0
Adiposity Markers	BMI	rs1799923	3	42306294	A	G	-0.022	0.002	8.4e-22	1.0e-04	93.2
Adiposity Markers	BMI	rs583173	3	173161912	A	G	0.009	0.002	6.2e-09	3.5e-05	33.1
Adiposity Markers	BMI	rs16852344	3	168194499	T	G	0.018	0.003	5.8e-10	4.0e-05	37.6
Adiposity Markers	BMI	rs12488237	3	56114861	T	C	-0.022	0.003	2.7e-12	4.8e-05	48.6
Adiposity Markers	BMI	rs11921432	3	35117776	T	C	-0.020	0.002	1.3e-17	7.9e-05	73.0
Adiposity Markers	BMI	rs925018	3	62713143	C	G	-0.014	0.002	1.3e-20	8.6e-05	88.4

Phenotype	Trait	SNP	Chr	Position (GRCh37/hg19)	EA	NEA	BETA	SE	P-value	R2	F
Adiposity Markers	BMI	rs1523768	3	77667044	A	G	-0.012	0.002	5.5e-16	6.7e-05	68.3
Adiposity Markers	BMI	rs936519	3	164275061	T	C	-0.009	0.002	7.1e-10	4.1e-05	38.4
Adiposity Markers	BMI	rs1801282	3	12393125	C	G	-0.020	0.002	2.9e-20	8.3e-05	85.1
Adiposity Markers	BMI	rs6765484	3	50041313	T	C	-0.021	0.001	1.3e-51	2.3e-04	235.8
Adiposity Markers	BMI	rs4017425	3	44028764	T	C	-0.012	0.002	3.7e-15	6.5e-05	60.8
Adiposity Markers	BMI	rs9851256	3	141177845	T	C	0.012	0.002	6.1e-15	6.3e-05	59.1
Adiposity Markers	BMI	rs16834431	3	123285856	T	C	0.012	0.002	1.9e-10	4.2e-05	39.2
Adiposity Markers	BMI	rs4624596	3	119571541	T	C	0.011	0.002	6.3e-09	3.7e-05	34.7
Adiposity Markers	BMI	rs17727959	3	70034728	A	G	-0.011	0.002	1.7e-10	4.2e-05	39.6
Adiposity Markers	BMI	rs2279440	3	9518511	T	C	0.015	0.002	4.0e-12	5.1e-05	47.1
Adiposity Markers	BMI	rs6804842	3	25106437	A	G	-0.015	0.001	1.2e-25	1.1e-04	114.8
Adiposity Markers	BMI	rs9816226	3	185834499	A	T	-0.033	0.002	1.3e-69	3.0e-04	303.5
Adiposity Markers	BMI	rs2682406	3	158026489	A	T	-0.011	0.002	1.2e-13	5.9e-05	54.8
Adiposity Markers	BMI	rs9812561	3	135762510	T	C	0.010	0.002	4.4e-10	4.1e-05	38.3
Adiposity Markers	BMI	rs17535749	3	10027724	A	G	0.013	0.002	2.4e-08	3.0e-05	30.7
Adiposity Markers	BMI	rs11720848	3	53783697	A	G	-0.015	0.002	1.4e-16	7.0e-05	65.8
Adiposity Markers	BMI	rs12633841	3	11630252	T	G	0.012	0.002	1.9e-10	4.6e-05	42.6
Adiposity Markers	BMI	rs10510757	3	51410064	C	G	0.031	0.005	3.7e-10	4.1e-05	39.3
Adiposity Markers	BMI	rs39654	3	173095123	A	G	-0.014	0.002	9.1e-22	9.9e-05	92.2
Adiposity Markers	BMI	rs4456860	3	180840488	T	C	0.013	0.002	5.0e-13	5.8e-05	54.6
Adiposity Markers	BMI	rs4133027	3	28624327	T	C	0.009	0.002	3.4e-08	3.2e-05	29.6
Adiposity Markers	BMI	rs12152350	3	176900551	A	G	-0.009	0.001	1.7e-09	3.7e-05	37.7
Adiposity Markers	BMI	rs17747268	3	71055582	A	C	-0.026	0.004	9.7e-09	3.6e-05	32.9
Adiposity Markers	BMI	rs936032	3	176324455	T	C	0.010	0.002	7.1e-09	3.5e-05	32.6
Adiposity Markers	BMI	rs11711337	3	21794904	A	T	-0.010	0.002	4.4e-09	3.6e-05	33.2
Adiposity Markers	BMI	rs9829442	3	166190518	T	C	-0.012	0.002	2.1e-08	3.4e-05	31.0
Adiposity Markers	BMI	rs2306272	3	66434643	T	C	-0.010	0.002	3.6e-09	3.6e-05	33.2
Adiposity Markers	BMI	rs6808814	3	116852469	T	C	0.011	0.002	2.1e-10	4.2e-05	38.9
Adiposity Markers	BMI	rs9814633	3	41310470	A	G	0.009	0.002	1.2e-08	3.4e-05	31.6
Adiposity Markers	BMI	rs4857968	3	20714580	A	G	-0.012	0.002	2.0e-14	5.7e-05	58.1
Adiposity Markers	BMI	rs9817496	3	107343437	T	C	0.012	0.002	6.9e-10	4.1e-05	38.4
Adiposity Markers	BMI	rs1006896	3	88104411	A	C	0.024	0.002	4.2e-24	1.1e-04	101.7
Adiposity Markers	BMI	rs2972504	3	115483341	A	G	-0.013	0.002	1.9e-09	3.9e-05	36.0
Adiposity Markers	BMI	rs355777	3	154034950	C	G	0.013	0.002	1.8e-18	8.2e-05	76.3
Adiposity Markers	BMI	rs17019905	3	82588812	C	G	-0.016	0.002	9.4e-16	7.2e-05	67.4
Adiposity Markers	BMI	rs9855260	3	116993247	T	C	0.011	0.002	9.1e-11	4.3e-05	40.4
Adiposity Markers	BMI	rs1248842	3	84882896	A	G	0.042	0.007	5.1e-09	1.6e-04	34.3
Adiposity Markers	BMI	rs17824625	3	102322040	A	G	0.014	0.002	9.4e-10	3.9e-05	36.5
Adiposity Markers	BMI	rs4682718	3	44226332	A	G	-0.013	0.002	2.5e-11	5.0e-05	46.1
Adiposity Markers	BMI	rs4616635	3	64702275	C	G	-0.011	0.002	7.2e-12	4.6e-05	46.4
Adiposity Markers	BMI	rs2884792	3	35704421	A	G	0.010	0.002	7.9e-11	4.4e-05	40.6
Adiposity Markers	BMI	rs7611238	3	195072918	A	G	0.011	0.002	8.1e-14	6.0e-05	55.8
Adiposity Markers	BMI	rs6764533	3	196088464	A	G	0.011	0.002	1.0e-11	4.8e-05	44.7
Adiposity Markers	BMI	rs6801848	3	185499057	C	G	0.009	0.002	3.0e-08	3.0e-05	30.2
Adiposity Markers	BMI	rs7612590	3	129054229	T	C	-0.014	0.002	1.2e-08	3.4e-05	31.6
Adiposity Markers	BMI	rs6781254	3	80649139	T	C	0.012	0.002	3.1e-14	5.7e-05	57.2
Adiposity Markers	BMI	rs16851483	3	141275436	T	G	0.037	0.003	1.1e-35	1.6e-04	153.8
Adiposity Markers	BMI	rs253664	3	138113353	T	C	0.013	0.002	1.5e-12	4.8e-05	49.0
Adiposity Markers	BMI	rs471976	3	170448937	T	C	0.010	0.002	2.3e-08	3.2e-05	30.2
Adiposity Markers	BMI	rs4683096	3	45374496	T	G	0.010	0.002	7.2e-11	4.6e-05	42.7
Adiposity Markers	BMI	rs1454687	3	94038085	C	G	0.018	0.002	1.4e-32	1.5e-04	139.2
Adiposity Markers	BMI	rs11915371	3	70539559	A	C	-0.014	0.002	1.2e-14	6.6e-05	61.4
Adiposity Markers	BMI	rs6786862	3	59905247	C	G	0.024	0.004	7.4e-09	3.6e-05	32.9
Adiposity Markers	BMI	rs17619661	3	114402087	T	C	-0.021	0.003	6.6e-15	6.6e-05	60.5
Adiposity Markers	BMI	rs4858802	3	47641011	T	C	0.012	0.002	2.5e-13	5.7e-05	53.5
Adiposity Markers	BMI	rs11920002	3	131616345	T	C	-0.025	0.003	3.5e-18	8.2e-05	76.7
Adiposity Markers	BMI	rs779206	3	118023515	A	G	-0.012	0.002	4.5e-12	5.2e-05	48.2
Adiposity Markers	BMI	rs7631156	3	131751628	A	G	0.022	0.002	5.7e-44	2.0e-04	189.1
Adiposity Markers	BMI	rs262976	3	183443403	A	G	0.011	0.002	2.8e-12	5.2e-05	49.0
Adiposity Markers	BMI	rs3752904	3	183996068	T	C	-0.013	0.001	3.1e-21	9.0e-05	91.6
Adiposity Markers	BMI	rs12696038	3	156295676	A	C	0.016	0.002	7.5e-14	6.0e-05	55.9
Adiposity Markers	BMI	rs10804719	3	147117260	A	G	-0.012	0.002	1.8e-08	3.5e-05	32.7

Phenotype	Trait	SNP	Chr	Position (GRCh37/hg19)	EA	NEA	BETA	SE	P-value	R2	F
Adiposity Markers	BMI	rs7374289	3	38633071	T	C	0.009	0.001	4.2e-10	4.0e-05	41.3
Adiposity Markers	BMI	rs7640424	3	107820063	T	C	-0.012	0.002	5.3e-14	5.5e-05	56.2
Adiposity Markers	BMI	rs6443750	3	181329682	T	C	-0.016	0.002	1.6e-18	7.2e-05	72.7
Adiposity Markers	BMI	rs963349	3	173681840	C	G	-0.011	0.002	1.9e-11	4.7e-05	43.4
Adiposity Markers	BMI	rs13078960	3	85807590	T	G	-0.022	0.002	2.2e-34	1.4e-04	146.7
Adiposity Markers	BMI	rs940338	3	61226761	A	C	0.026	0.002	3.2e-37	1.8e-04	170.3
Adiposity Markers	BMI	rs3773192	3	78660056	T	C	-0.012	0.002	2.0e-13	5.7e-05	53.2
Adiposity Markers	BMI	rs9845966	3	13433158	T	G	0.010	0.001	1.7e-11	4.7e-05	47.0
Adiposity Markers	BMI	rs16829530	3	178160703	A	G	0.023	0.004	4.2e-10	4.3e-05	39.3
Adiposity Markers	BMI	rs7433415	3	78467922	T	C	-0.014	0.002	3.0e-09	3.6e-05	34.0
Adiposity Markers	BMI	rs11130638	3	58405947	A	G	-0.010	0.002	3.4e-11	4.3e-05	43.6
Adiposity Markers	BMI	rs3849570	3	81792112	A	C	0.013	0.002	2.7e-17	7.4e-05	72.8
Adiposity Markers	BMI	rs9875219	3	117597695	T	G	0.010	0.002	3.7e-10	4.1e-05	38.3
Adiposity Markers	BMI	rs6809307	3	156862041	T	C	0.013	0.002	2.1e-14	6.1e-05	56.7
Adiposity Markers	BMI	rs1371838	3	29789779	T	C	-0.008	0.002	2.3e-08	3.4e-05	31.4
Adiposity Markers	BMI	rs11925953	3	142796422	T	C	-0.009	0.002	1.1e-09	3.9e-05	36.8
Adiposity Markers	BMI	rs1452075	3	62481063	T	C	0.013	0.002	9.4e-16	6.3e-05	64.0
Adiposity Markers	BMI	rs1554194	3	8138041	C	G	0.011	0.002	2.0e-12	5.3e-05	49.0
Adiposity Markers	BMI	rs4266190	3	86159976	T	G	-0.012	0.002	3.6e-15	6.6e-05	61.9
Adiposity Markers	BMI	rs9714342	3	90428286	T	C	-0.014	0.002	2.1e-16	8.2e-05	64.9
Adiposity Markers	BMI	rs2222098	3	5986390	A	C	0.012	0.002	1.3e-10	4.2e-05	39.2
Adiposity Markers	BMI	rs1849477	3	18245719	A	G	-0.013	0.002	1.4e-08	3.4e-05	31.5
Adiposity Markers	BMI	rs2255248	3	15754849	T	C	-0.008	0.001	1.0e-08	3.4e-05	34.3
Adiposity Markers	BMI	rs8192675	3	170724883	T	C	-0.015	0.002	3.1e-22	9.1e-05	92.6
Adiposity Markers	BMI	rs2200847	3	1212658	A	G	0.009	0.002	5.7e-09	3.6e-05	33.6
Adiposity Markers	BMI	rs1436348	3	104612668	A	G	-0.013	0.002	4.0e-19	8.4e-05	78.6
Adiposity Markers	BMI	rs2564944	3	53148455	C	G	0.011	0.002	5.2e-12	5.0e-05	46.4
Adiposity Markers	BMI	rs2124499	3	123093541	C	G	-0.012	0.002	1.2e-14	5.9e-05	59.8
Adiposity Markers	BMI	rs9827823	3	84221774	T	C	0.016	0.002	4.5e-16	7.3e-05	68.1
Adiposity Markers	BMI	rs6795301	3	141754558	A	G	-0.011	0.002	2.6e-08	3.4e-05	31.7
Adiposity Markers	BMI	rs16833232	3	182262873	T	C	-0.013	0.002	1.1e-15	6.7e-05	63.0
Adiposity Markers	BMI	rs4677252	3	73358567	T	C	0.010	0.002	1.9e-09	3.8e-05	35.3
Adiposity Markers	BMI	rs9829078	3	88446373	T	C	-0.009	0.002	8.4e-10	4.0e-05	37.6
Adiposity Markers	BMI	rs2322977	3	5368801	T	C	-0.010	0.002	2.3e-10	3.9e-05	39.8
Adiposity Markers	BMI	rs4273371	3	108119071	T	C	-0.010	0.001	1.8e-11	4.5e-05	46.0
Adiposity Markers	BMI	rs12630999	3	136300927	A	G	0.018	0.002	3.4e-24	1.1e-04	109.6
Adiposity Markers	BMI	rs10516497	4	103942714	A	C	0.013	0.002	5.3e-12	4.7e-05	48.2
Adiposity Markers	BMI	rs2175327	4	67406985	T	C	-0.010	0.002	1.2e-08	3.4e-05	31.9
Adiposity Markers	BMI	rs1455137	4	145986668	A	C	-0.011	0.002	2.3e-12	4.9e-05	49.9
Adiposity Markers	BMI	rs17507682	4	80620882	T	G	0.012	0.002	7.7e-09	3.6e-05	33.8
Adiposity Markers	BMI	rs10938397	4	45182527	A	G	-0.033	0.001	1.4e-116	5.3e-04	542.2
Adiposity Markers	BMI	rs16869307	4	20181554	T	G	-0.017	0.002	1.5e-14	6.3e-05	59.0
Adiposity Markers	BMI	rs6811330	4	164230038	T	G	0.009	0.002	4.6e-08	3.1e-05	28.7
Adiposity Markers	BMI	rs1437842	4	173597016	A	G	-0.011	0.002	4.1e-13	5.7e-05	52.8
Adiposity Markers	BMI	rs6845022	4	68171620	T	G	0.008	0.002	2.8e-08	3.3e-05	30.6
Adiposity Markers	BMI	rs11132137	4	183525697	T	C	-0.010	0.002	3.5e-08	3.4e-05	30.9
Adiposity Markers	BMI	rs2522501	4	105648283	A	C	-0.008	0.002	3.1e-08	3.3e-05	30.6
Adiposity Markers	BMI	rs12505133	4	26883909	T	C	-0.010	0.002	2.5e-08	3.3e-05	30.0
Adiposity Markers	BMI	rs4565118	4	180179877	A	C	-0.009	0.002	1.5e-08	3.9e-05	30.2
Adiposity Markers	BMI	rs6841761	4	25423538	T	G	-0.013	0.001	1.0e-20	8.7e-05	88.9
Adiposity Markers	BMI	rs2192158	4	55505360	A	G	0.012	0.002	6.0e-16	7.0e-05	65.1
Adiposity Markers	BMI	rs495323	4	152824343	T	C	-0.013	0.002	1.1e-09	4.9e-05	37.7
Adiposity Markers	BMI	rs1020073	4	36758646	A	G	0.012	0.002	6.4e-09	3.7e-05	34.2
Adiposity Markers	BMI	rs4392496	4	154442505	A	C	-0.009	0.002	9.3e-10	4.1e-05	38.4
Adiposity Markers	BMI	rs326889	4	112713436	T	C	-0.013	0.002	8.9e-17	7.4e-05	69.4
Adiposity Markers	BMI	rs797090	4	140867514	A	G	0.012	0.002	7.1e-15	6.5e-05	60.8
Adiposity Markers	BMI	rs12645001	4	96018337	A	G	-0.011	0.002	1.6e-11	4.8e-05	44.7
Adiposity Markers	BMI	rs4699650	4	99466330	A	G	0.009	0.002	2.2e-08	3.2e-05	30.2
Adiposity Markers	BMI	rs3796606	4	81194090	A	G	0.008	0.001	5.0e-09	3.5e-05	36.0
Adiposity Markers	BMI	rs16858004	4	45098080	T	C	0.014	0.002	2.5e-10	4.3e-05	39.9
Adiposity Markers	BMI	rs4834272	4	113313986	T	C	-0.010	0.002	2.0e-11	4.4e-05	45.3
Adiposity Markers	BMI	rs6818414	4	16600664	T	C	-0.010	0.002	2.1e-10	4.3e-05	40.1

Phenotype	Trait	SNP	Chr	Position (GRCh37/hg19)	EA	NEA	BETA	SE	P-value	R2	F
Adiposity Markers	BMI	rs10517806	4	165284262	C	G	-0.009	0.002	2.9e-10	4.2e-05	39.3
Adiposity Markers	BMI	rs2583401	4	102204529	T	C	0.015	0.002	6.3e-14	6.2e-05	57.8
Adiposity Markers	BMI	rs17019336	4	145333609	A	T	-0.013	0.002	4.5e-15	6.1e-05	62.1
Adiposity Markers	BMI	rs346928	4	53415541	T	G	0.015	0.002	4.5e-10	4.3e-05	40.1
Adiposity Markers	BMI	rs17698720	4	28549589	A	G	0.012	0.002	4.4e-09	3.7e-05	34.3
Adiposity Markers	BMI	rs1037814	4	88049850	T	C	-0.008	0.001	4.3e-08	3.0e-05	31.0
Adiposity Markers	BMI	rs3914628	4	147438019	T	C	0.015	0.002	1.7e-13	5.6e-05	57.0
Adiposity Markers	BMI	rs17630894	4	171532023	T	G	0.014	0.002	4.0e-10	4.2e-05	39.3
Adiposity Markers	BMI	rs1346841	4	65651730	A	G	-0.011	0.002	3.9e-13	5.7e-05	52.8
Adiposity Markers	BMI	rs6835302	4	16861755	T	C	-0.009	0.002	1.1e-08	3.5e-05	32.9
Adiposity Markers	BMI	rs964488	4	136113074	C	G	0.008	0.002	2.5e-08	3.3e-05	30.6
Adiposity Markers	BMI	rs1397802	4	60233749	A	C	0.010	0.002	7.4e-11	4.4e-05	40.6
Adiposity Markers	BMI	rs13147493	4	89742179	T	C	-0.008	0.001	8.2e-09	3.4e-05	34.3
Adiposity Markers	BMI	rs10017371	4	120293776	A	G	-0.011	0.002	5.9e-12	5.0e-05	46.4
Adiposity Markers	BMI	rs13110266	4	162129844	A	G	-0.012	0.001	1.6e-16	6.9e-05	71.0
Adiposity Markers	BMI	rs1531583	4	744972	T	G	-0.021	0.004	1.1e-08	3.8e-05	33.4
Adiposity Markers	BMI	rs4864201	4	130731284	T	C	0.014	0.002	3.4e-20	8.3e-05	84.6
Adiposity Markers	BMI	rs7669124	4	31019816	A	G	0.011	0.002	1.4e-13	5.9e-05	54.8
Adiposity Markers	BMI	rs1813006	4	103001649	T	G	0.051	0.003	7.9e-53	2.7e-04	237.0
Adiposity Markers	BMI	rs6854583	4	115053885	A	C	0.009	0.002	7.5e-10	4.0e-05	37.6
Adiposity Markers	BMI	rs6829208	4	52728640	T	C	-0.010	0.002	3.5e-09	3.4e-05	35.3
Adiposity Markers	BMI	rs4148155	4	89054667	A	G	0.018	0.002	1.8e-14	5.7e-05	58.6
Adiposity Markers	BMI	rs13152233	4	143076850	A	T	0.013	0.002	4.0e-08	3.3e-05	30.5
Adiposity Markers	BMI	rs3857068	4	95026494	C	G	0.010	0.002	1.9e-10	4.3e-05	40.1
Adiposity Markers	BMI	rs2271391	4	140787695	T	C	-0.010	0.002	5.1e-11	4.5e-05	42.2
Adiposity Markers	BMI	rs1403846	4	119101723	T	C	0.010	0.002	3.1e-08	3.0e-05	30.0
Adiposity Markers	BMI	rs1903579	4	91253956	C	G	0.010	0.002	2.3e-11	4.7e-05	43.6
Adiposity Markers	BMI	rs750090	4	152931436	T	C	0.010	0.002	2.0e-09	3.8e-05	35.3
Adiposity Markers	BMI	rs1511864	4	34831561	T	C	-0.010	0.002	1.6e-09	4.0e-05	36.7
Adiposity Markers	BMI	rs4270551	4	30703243	A	C	0.017	0.002	5.6e-14	6.1e-05	56.6
Adiposity Markers	BMI	rs1296328	4	137083193	A	C	0.017	0.002	8.7e-29	1.3e-04	124.0
Adiposity Markers	BMI	rs4235396	4	18575715	A	G	-0.012	0.002	1.4e-13	5.8e-05	54.4
Adiposity Markers	BMI	rs1655069	4	35923989	A	G	0.011	0.002	2.8e-11	4.6e-05	43.1
Adiposity Markers	BMI	rs6834210	4	94436697	A	T	-0.014	0.002	2.6e-14	5.9e-05	55.1
Adiposity Markers	BMI	rs2768950	4	49064487	A	G	0.010	0.002	2.1e-09	3.6e-05	36.0
Adiposity Markers	BMI	rs7672868	4	516398	A	G	0.014	0.002	7.5e-09	3.7e-05	32.3
Adiposity Markers	BMI	rs13105040	4	172201889	T	C	0.010	0.002	7.2e-09	3.8e-05	35.3
Adiposity Markers	BMI	rs3775820	4	20540540	A	G	-0.009	0.002	3.1e-08	3.2e-05	30.2
Adiposity Markers	BMI	rs10033843	4	77028783	A	G	0.013	0.002	6.6e-13	5.7e-05	53.0
Adiposity Markers	BMI	rs1976613	4	179339331	C	G	0.009	0.002	3.3e-09	3.8e-05	35.2
Adiposity Markers	BMI	rs11724872	4	144140665	T	C	-0.009	0.001	6.4e-11	4.3e-05	44.1
Adiposity Markers	BMI	rs2051559	4	3298800	T	C	-0.014	0.002	1.3e-10	4.4e-05	41.1
Adiposity Markers	BMI	rs3209570	4	38699657	A	G	-0.015	0.002	7.1e-22	1.0e-04	93.4
Adiposity Markers	BMI	rs4895231	5	119388659	C	G	0.012	0.002	3.2e-15	6.8e-05	62.9
Adiposity Markers	BMI	rs325220	5	98304564	A	G	-0.011	0.002	8.0e-12	4.5e-05	46.4
Adiposity Markers	BMI	rs1582931	5	122657199	A	G	-0.012	0.002	1.8e-16	7.1e-05	66.2
Adiposity Markers	BMI	rs9327000	5	115709156	A	C	0.010	0.002	8.6e-11	4.4e-05	41.4
Adiposity Markers	BMI	rs6866004	5	122042452	A	G	-0.008	0.002	1.8e-08	3.4e-05	31.4
Adiposity Markers	BMI	rs4865796	5	53272664	A	G	-0.011	0.002	3.2e-11	4.2e-05	43.1
Adiposity Markers	BMI	rs2112347	5	75015242	T	G	0.026	0.002	1.8e-69	3.0e-04	309.8
Adiposity Markers	BMI	rs13157978	5	80899408	C	G	0.013	0.002	1.3e-15	6.6e-05	62.0
Adiposity Markers	BMI	rs13153873	5	37276128	C	G	0.012	0.002	1.4e-10	4.6e-05	42.6
Adiposity Markers	BMI	rs1404989	5	75184420	A	G	-0.016	0.002	1.0e-12	5.4e-05	50.2
Adiposity Markers	BMI	rs33730	5	108672755	A	T	-0.011	0.002	1.4e-12	5.3e-05	49.9
Adiposity Markers	BMI	rs10473333	5	43866596	A	T	0.022	0.003	8.0e-12	4.8e-05	47.7
Adiposity Markers	BMI	rs6452794	5	87823299	T	C	-0.017	0.002	2.7e-21	9.2e-05	86.1
Adiposity Markers	BMI	rs982305	5	164562193	A	G	-0.011	0.002	1.0e-12	5.4e-05	50.9
Adiposity Markers	BMI	rs13436896	5	139067098	A	G	0.014	0.002	4.5e-18	7.9e-05	73.3
Adiposity Markers	BMI	rs7714712	5	60732552	A	C	-0.012	0.002	1.4e-14	6.1e-05	57.2
Adiposity Markers	BMI	rs261076	5	169304372	A	G	-0.011	0.002	7.2e-10	4.3e-05	40.1
Adiposity Markers	BMI	rs11739877	5	105876806	T	C	0.010	0.002	8.2e-10	3.9e-05	36.0
Adiposity Markers	BMI	rs919223	5	153271230	T	C	0.010	0.002	8.0e-10	3.9e-05	36.7

Phenotype	Trait	SNP	Chr	Position (GRCh37/hg19)	EA	NEA	BETA	SE	P-value	R2	F
Adiposity Markers	BMI	rs10068261	5	84365798	T	C	0.010	0.002	1.1e-10	4.5e-05	41.8
Adiposity Markers	BMI	rs254035	5	103936343	A	T	0.009	0.002	2.3e-09	3.8e-05	35.2
Adiposity Markers	BMI	rs7724203	5	61119308	T	C	0.013	0.002	1.2e-08	3.5e-05	32.4
Adiposity Markers	BMI	rs12652212	5	88808594	A	G	-0.012	0.001	4.4e-18	7.6e-05	77.2
Adiposity Markers	BMI	rs7730247	5	131781288	A	C	-0.010	0.002	1.5e-08	3.0e-05	30.9
Adiposity Markers	BMI	rs4703529	5	81129876	A	G	-0.012	0.002	1.9e-08	3.4e-05	31.6
Adiposity Markers	BMI	rs12189178	5	50914726	T	C	0.034	0.004	2.0e-17	7.2e-05	73.1
Adiposity Markers	BMI	rs6879326	5	59208302	T	C	-0.009	0.002	3.9e-09	3.7e-05	34.4
Adiposity Markers	BMI	rs34415	5	107428786	T	C	-0.023	0.002	2.8e-31	1.4e-04	128.8
Adiposity Markers	BMI	rs4912637	5	141720261	A	G	-0.010	0.002	3.0e-10	4.1e-05	38.3
Adiposity Markers	BMI	rs4532349	5	77473165	A	G	0.012	0.002	1.1e-12	4.9e-05	49.8
Adiposity Markers	BMI	rs6864049	5	124330522	A	G	-0.012	0.001	1.9e-16	6.8e-05	69.8
Adiposity Markers	BMI	rs1465406	5	148116306	A	C	0.010	0.002	9.5e-09	3.4e-05	31.5
Adiposity Markers	BMI	rs323742	5	86769015	A	T	-0.031	0.005	2.9e-11	4.8e-05	44.1
Adiposity Markers	BMI	rs2118793	5	152509939	A	C	-0.009	0.002	8.9e-10	4.0e-05	37.6
Adiposity Markers	BMI	rs698147	5	3513485	A	G	0.010	0.002	6.6e-11	4.5e-05	41.8
Adiposity Markers	BMI	rs782978	5	43146329	T	C	-0.015	0.002	5.0e-18	7.8e-05	72.8
Adiposity Markers	BMI	rs4509057	5	86165391	T	C	0.010	0.002	9.3e-12	5.0e-05	46.2
Adiposity Markers	BMI	rs112560558	5	7581938	A	G	-0.021	0.004	3.9e-09	1.7e-04	35.7
Adiposity Markers	BMI	rs2112457	5	86087631	T	C	-0.013	0.002	6.1e-11	4.6e-05	43.3
Adiposity Markers	BMI	rs6865920	5	167389006	A	T	0.013	0.002	6.2e-10	4.2e-05	39.1
Adiposity Markers	BMI	rs4701523	5	26139138	A	T	-0.017	0.003	1.6e-08	3.3e-05	31.1
Adiposity Markers	BMI	rs4912652	5	142888684	T	C	-0.010	0.002	5.3e-11	4.6e-05	42.7
Adiposity Markers	BMI	rs1822489	5	112542527	A	G	0.010	0.002	3.9e-09	3.8e-05	35.3
Adiposity Markers	BMI	rs13189692	5	136569448	A	G	-0.010	0.002	5.1e-11	4.6e-05	42.7
Adiposity Markers	BMI	rs7715256	5	153537893	T	G	-0.016	0.001	1.8e-30	1.3e-04	135.6
Adiposity Markers	BMI	rs17056301	5	158271680	T	C	-0.011	0.002	3.0e-11	4.6e-05	43.4
Adiposity Markers	BMI	rs2292144	5	168213385	T	C	-0.016	0.002	6.4e-10	4.3e-05	39.4
Adiposity Markers	BMI	rs10073659	5	107954472	T	C	-0.023	0.004	5.2e-09	3.7e-05	33.6
Adiposity Markers	BMI	rs4640754	5	27186133	A	T	-0.011	0.002	2.8e-11	4.5e-05	41.9
Adiposity Markers	BMI	rs2190788	5	144484261	T	G	0.012	0.002	1.2e-14	6.3e-05	59.1
Adiposity Markers	BMI	rs17695092	5	173337853	T	G	0.012	0.002	5.3e-14	5.5e-05	55.3
Adiposity Markers	BMI	rs350924	5	165850050	A	T	0.009	0.002	1.4e-09	4.0e-05	37.6
Adiposity Markers	BMI	rs2973564	5	73151033	A	G	0.009	0.002	2.2e-08	3.2e-05	29.9
Adiposity Markers	BMI	rs2053046	5	168198967	T	C	0.010	0.002	2.8e-08	3.5e-05	32.6
Adiposity Markers	BMI	rs6556301	5	176527577	T	G	-0.013	0.002	2.3e-17	7.5e-05	74.0
Adiposity Markers	BMI	rs1994652	5	67206742	T	C	0.009	0.002	9.4e-09	3.6e-05	33.1
Adiposity Markers	BMI	rs7730898	5	170459675	A	G	0.015	0.002	3.8e-21	8.7e-05	89.1
Adiposity Markers	BMI	rs329122	5	133864599	A	G	-0.012	0.001	6.8e-18	7.5e-05	77.2
Adiposity Markers	BMI	rs41290587	5	151045923	T	C	0.122	0.018	5.0e-11	2.0e-04	43.2
Adiposity Markers	BMI	rs12519652	5	165243019	T	C	0.008	0.002	4.8e-08	3.0e-05	30.6
Adiposity Markers	BMI	rs265180	5	4244462	T	C	0.009	0.002	6.8e-09	3.5e-05	32.9
Adiposity Markers	BMI	rs3811958	5	32772043	A	G	0.009	0.002	1.3e-08	3.2e-05	32.3
Adiposity Markers	BMI	rs2282802	5	139712239	A	G	0.011	0.002	8.9e-14	5.9e-05	54.8
Adiposity Markers	BMI	rs353259	5	148771844	T	G	-0.008	0.002	3.7e-08	3.4e-05	31.4
Adiposity Markers	BMI	rs7736910	5	63040773	A	G	-0.016	0.002	5.5e-25	1.1e-04	106.8
Adiposity Markers	BMI	rs6555747	5	166885759	A	C	-0.009	0.002	2.3e-08	3.4e-05	31.6
Adiposity Markers	BMI	rs455938	5	66198859	A	G	-0.013	0.002	7.8e-15	6.3e-05	58.5
Adiposity Markers	BMI	rs12522567	5	64087023	T	G	0.010	0.001	6.1e-12	4.9e-05	50.0
Adiposity Markers	BMI	rs7700874	5	95808483	T	C	0.010	0.002	8.5e-12	5.0e-05	46.2
Adiposity Markers	BMI	rs1501672	5	87963761	A	G	-0.026	0.002	1.2e-36	1.6e-04	166.4
Adiposity Markers	BMI	rs6886740	5	63928254	T	C	0.011	0.002	1.4e-11	4.8e-05	44.7
Adiposity Markers	BMI	rs17669543	5	113987780	A	G	0.011	0.002	1.1e-09	3.8e-05	35.3
Adiposity Markers	BMI	rs6235	5	95728898	C	G	-0.016	0.002	1.1e-20	8.9e-05	83.1
Adiposity Markers	BMI	rs1363695	5	130378027	T	C	-0.011	0.002	1.2e-10	4.3e-05	40.1
Adiposity Markers	BMI	rs159032	5	94206202	T	C	0.010	0.002	5.6e-09	3.9e-05	36.0
Adiposity Markers	BMI	rs2305736	5	167946076	A	G	0.011	0.002	1.2e-09	3.6e-05	36.7
Adiposity Markers	BMI	rs246551	5	71829971	A	T	0.011	0.002	8.8e-10	3.8e-05	35.3
Adiposity Markers	BMI	rs459552	5	112176756	A	T	-0.012	0.002	7.3e-12	4.5e-05	45.8
Adiposity Markers	BMI	rs1837269	5	95859144	T	C	-0.017	0.002	1.2e-29	1.4e-04	128.4
Adiposity Markers	BMI	rs980888	5	111293695	T	G	-0.010	0.002	2.2e-10	4.3e-05	39.8
Adiposity Markers	BMI	rs6885199	5	92574633	A	G	0.024	0.004	9.5e-09	3.6e-05	32.5

Phenotype	Trait	SNP	Chr	Position (GRCh37/hg19)	EA	NEA	BETA	SE	P-value	R2	F
Adiposity Markers	BMI	rs3822683	5	96080883	A	G	0.014	0.002	1.6e-14	6.1e-05	57.1
Adiposity Markers	BMI	rs11744049	5	137637183	T	C	-0.015	0.002	3.8e-14	6.3e-05	59.0
Adiposity Markers	BMI	rs4382152	5	106379106	T	C	0.010	0.002	8.4e-11	4.5e-05	41.8
Adiposity Markers	BMI	rs885624	5	107300655	T	C	-0.012	0.002	1.8e-12	5.1e-05	47.4
Adiposity Markers	BMI	rs4703019	5	102053558	A	G	0.009	0.001	6.3e-10	3.9e-05	39.5
Adiposity Markers	BMI	rs2453763	5	92376460	A	T	0.011	0.002	1.1e-13	6.0e-05	55.8
Adiposity Markers	BMI	rs765875	6	143185683	T	C	-0.012	0.002	9.1e-17	7.3e-05	68.3
Adiposity Markers	BMI	rs6915002	6	54028069	T	C	0.009	0.002	9.5e-09	3.4e-05	32.1
Adiposity Markers	BMI	rs17789218	6	100600097	T	C	-0.012	0.002	3.7e-12	4.6e-05	46.6
Adiposity Markers	BMI	rs12203240	6	24749104	T	C	0.018	0.003	2.9e-10	3.9e-05	38.5
Adiposity Markers	BMI	rs17805532	6	18497348	T	C	0.023	0.003	2.3e-12	5.3e-05	48.6
Adiposity Markers	BMI	rs9395885	6	53745477	T	C	0.017	0.003	4.3e-10	4.1e-05	38.7
Adiposity Markers	BMI	rs1768950	6	70220355	C	G	-0.011	0.002	2.0e-11	4.7e-05	44.2
Adiposity Markers	BMI	rs11753081	6	20705590	T	G	0.014	0.002	4.0e-13	5.0e-05	50.5
Adiposity Markers	BMI	rs11757081	6	33459420	A	T	-0.016	0.002	4.2e-21	1.0e-04	94.2
Adiposity Markers	BMI	rs9344003	6	81159931	A	G	0.015	0.002	1.9e-10	4.2e-05	39.6
Adiposity Markers	BMI	rs11760037	6	40590979	T	C	-0.022	0.004	2.8e-08	3.4e-05	31.2
Adiposity Markers	BMI	rs877446	6	39031039	A	G	-0.010	0.002	4.3e-12	5.1e-05	48.1
Adiposity Markers	BMI	rs12210292	6	44925293	T	C	0.010	0.002	4.6e-09	3.6e-05	33.4
Adiposity Markers	BMI	rs17757975	6	38214150	T	C	0.016	0.002	4.7e-14	6.4e-05	59.5
Adiposity Markers	BMI	rs12663742	6	97792735	A	G	-0.012	0.002	1.8e-16	7.2e-05	67.2
Adiposity Markers	BMI	rs9357429	6	44046143	T	C	-0.012	0.002	3.3e-08	3.3e-05	30.2
Adiposity Markers	BMI	rs998584	6	43757896	A	C	-0.014	0.002	5.4e-21	8.8e-05	87.1
Adiposity Markers	BMI	rs13203153	6	97374850	A	G	-0.011	0.002	1.7e-08	3.5e-05	32.9
Adiposity Markers	BMI	rs16870458	6	10545728	A	G	0.011	0.002	2.6e-09	3.3e-05	34.0
Adiposity Markers	BMI	rs2357760	6	120213880	A	G	0.013	0.002	1.3e-18	7.7e-05	78.6
Adiposity Markers	BMI	rs13201877	6	137675541	A	G	-0.013	0.002	1.1e-09	3.5e-05	36.0
Adiposity Markers	BMI	rs13191362	6	163033350	A	G	0.022	0.002	6.6e-24	1.0e-04	102.7
Adiposity Markers	BMI	rs9382487	6	55203369	C	G	-0.010	0.002	7.3e-11	4.6e-05	42.7
Adiposity Markers	BMI	rs820082	6	35031441	T	C	-0.015	0.002	5.3e-18	7.6e-05	71.3
Adiposity Markers	BMI	rs12191738	6	73224090	T	C	-0.011	0.002	1.6e-08	3.5e-05	32.9
Adiposity Markers	BMI	rs209139	6	28863483	A	C	0.020	0.003	3.8e-11	4.2e-05	42.9
Adiposity Markers	BMI	rs10498767	6	46363557	C	G	-0.011	0.002	9.6e-14	6.0e-05	55.8
Adiposity Markers	BMI	rs9478671	6	155987825	A	G	-0.011	0.002	6.0e-10	4.3e-05	40.1
Adiposity Markers	BMI	rs7760082	6	21919387	A	G	-0.011	0.002	3.8e-12	5.1e-05	47.3
Adiposity Markers	BMI	rs7758921	6	50064897	T	C	0.021	0.004	2.0e-09	3.5e-05	35.3
Adiposity Markers	BMI	rs9364687	6	163817911	T	G	-0.011	0.001	4.2e-15	6.3e-05	64.0
Adiposity Markers	BMI	rs2875762	6	124925032	C	G	0.014	0.002	9.0e-15	6.8e-05	63.1
Adiposity Markers	BMI	rs9367368	6	13189275	T	C	0.011	0.002	1.0e-11	4.5e-05	45.6
Adiposity Markers	BMI	rs2221824	6	23877024	T	C	-0.009	0.002	6.0e-09	3.5e-05	33.1
Adiposity Markers	BMI	rs2228213	6	12124855	A	G	-0.016	0.002	4.8e-25	1.0e-04	106.8
Adiposity Markers	BMI	rs768023	6	108876002	A	G	0.015	0.001	8.3e-27	1.2e-04	119.4
Adiposity Markers	BMI	rs1265079	6	31112108	T	G	0.011	0.002	1.0e-12	5.3e-05	49.0
Adiposity Markers	BMI	rs12173930	6	78186434	T	C	0.012	0.002	4.7e-09	3.7e-05	34.3
Adiposity Markers	BMI	rs1057691	6	34299232	A	G	-0.013	0.002	1.2e-08	3.3e-05	31.2
Adiposity Markers	BMI	rs2178899	6	31606756	A	T	0.024	0.002	3.7e-28	1.2e-04	122.0
Adiposity Markers	BMI	rs6569648	6	130349119	T	C	-0.014	0.002	1.7e-17	6.9e-05	70.8
Adiposity Markers	BMI	rs9320823	6	98429337	T	C	-0.015	0.002	1.3e-23	1.1e-04	100.0
Adiposity Markers	BMI	rs1546856	6	5606417	C	G	0.011	0.002	1.4e-10	4.2e-05	39.4
Adiposity Markers	BMI	rs3846783	6	166395367	A	C	0.014	0.002	9.4e-11	4.6e-05	42.2
Adiposity Markers	BMI	rs2781668	6	131897278	T	C	0.016	0.002	7.2e-16	6.7e-05	61.7
Adiposity Markers	BMI	rs6901756	6	41825590	T	C	0.015	0.002	6.8e-12	4.8e-05	49.0
Adiposity Markers	BMI	rs1324110	6	93913200	C	G	-0.008	0.001	5.3e-09	3.5e-05	36.0
Adiposity Markers	BMI	rs1327259	6	51177811	A	G	0.015	0.002	1.3e-23	1.1e-04	101.3
Adiposity Markers	BMI	rs17630640	6	164107529	A	G	-0.014	0.002	6.2e-10	4.0e-05	37.7
Adiposity Markers	BMI	rs2880652	6	69900571	A	G	0.012	0.002	1.9e-08	3.4e-05	31.6
Adiposity Markers	BMI	rs6922607	6	142703483	A	G	-0.012	0.002	8.7e-10	4.1e-05	37.9
Adiposity Markers	BMI	rs2236027	6	127768950	A	C	-0.008	0.002	4.6e-08	3.2e-05	29.9
Adiposity Markers	BMI	rs2256216	6	147471730	A	G	-0.010	0.002	1.3e-10	4.5e-05	42.2
Adiposity Markers	BMI	rs9294260	6	83433228	A	G	0.012	0.001	9.7e-16	6.6e-05	67.5
Adiposity Markers	BMI	rs6942222	6	107873783	A	T	-0.009	0.002	1.2e-08	3.5e-05	32.9
Adiposity Markers	BMI	rs707889	6	26095931	A	G	-0.013	0.002	1.5e-15	6.1e-05	62.1

Phenotype	Trait	SNP	Chr	Position (GRCh37/hg19)	EA	NEA	BETA	SE	P-value	R2	F
Adiposity Markers	BMI	rs2744974	6	34579431	T	C	0.024	0.002	1.1e-56	2.5e-04	258.1
Adiposity Markers	BMI	rs1116221	6	30071330	T	C	0.011	0.002	2.9e-12	4.7e-05	48.1
Adiposity Markers	BMI	rs486359	6	160774441	C	G	0.010	0.001	2.7e-12	5.0e-05	50.0
Adiposity Markers	BMI	rs5875652	6	39019672	T	G	-0.020	0.003	9.0e-10	1.7e-04	37.1
Adiposity Markers	BMI	rs11755280	6	118117246	T	C	0.010	0.002	1.8e-08	3.4e-05	31.9
Adiposity Markers	BMI	rs10499276	6	154309808	T	C	0.016	0.002	1.8e-14	5.9e-05	60.2
Adiposity Markers	BMI	rs10948367	6	47585615	A	G	0.010	0.002	6.6e-09	3.5e-05	32.6
Adiposity Markers	BMI	rs12213055	6	27330685	T	C	-0.012	0.002	3.7e-10	4.3e-05	39.9
Adiposity Markers	BMI	rs156126	6	104810083	T	C	-0.016	0.002	8.8e-19	8.1e-05	75.4
Adiposity Markers	BMI	rs9463175	6	9510030	T	C	-0.010	0.002	1.9e-11	4.5e-05	45.3
Adiposity Markers	BMI	rs16883089	6	73658053	T	C	-0.011	0.002	1.2e-09	4.1e-05	38.7
Adiposity Markers	BMI	rs1179907	6	90324849	A	G	-0.012	0.002	2.2e-11	4.6e-05	42.6
Adiposity Markers	BMI	rs9374412	6	113973972	A	T	0.012	0.002	9.5e-10	4.1e-05	37.9
Adiposity Markers	BMI	rs1853639	6	87606842	A	G	-0.010	0.002	1.1e-09	3.9e-05	36.0
Adiposity Markers	BMI	rs17681686	6	40367138	C	G	0.021	0.002	7.1e-41	1.9e-04	177.2
Adiposity Markers	BMI	rs11754747	6	141494602	T	C	0.012	0.002	3.5e-13	5.2e-05	53.2
Adiposity Markers	BMI	rs4712972	6	25772047	A	G	0.013	0.002	8.4e-11	4.3e-05	44.2
Adiposity Markers	BMI	rs4593336	6	86181931	A	G	0.008	0.002	4.9e-08	2.9e-05	29.9
Adiposity Markers	BMI	rs9688431	6	73922654	T	C	0.021	0.003	7.3e-12	4.6e-05	47.2
Adiposity Markers	BMI	rs1159974	6	126090277	T	C	-0.012	0.002	1.1e-15	6.7e-05	62.9
Adiposity Markers	BMI	rs1043784	6	7881931	T	C	-0.014	0.002	3.3e-09	3.7e-05	35.0
Adiposity Markers	BMI	rs9394767	6	41159905	A	G	-0.012	0.002	1.9e-12	5.1e-05	47.4
Adiposity Markers	BMI	rs1885728	6	5977833	A	G	0.009	0.002	3.3e-09	3.7e-05	34.5
Adiposity Markers	BMI	rs742900	6	42624555	C	G	-0.011	0.002	2.2e-13	5.8e-05	53.8
Adiposity Markers	BMI	rs2207139	6	50845490	A	G	-0.038	0.002	1.0e-92	3.9e-04	402.1
Adiposity Markers	BMI	rs2756117	6	153066908	A	G	-0.013	0.002	3.5e-08	3.3e-05	30.2
Adiposity Markers	BMI	rs7748644	6	101166809	T	G	-0.011	0.002	5.3e-14	6.0e-05	55.8
Adiposity Markers	BMI	rs2185027	6	153381622	A	C	-0.014	0.002	7.3e-19	7.5e-05	76.6
Adiposity Markers	BMI	rs78421949	6	56681464	A	G	-0.039	0.007	4.4e-08	1.4e-04	30.4
Adiposity Markers	BMI	rs2622274	6	64240516	T	G	-0.012	0.002	6.7e-16	7.0e-05	65.1
Adiposity Markers	BMI	rs1736904	6	28219270	A	G	0.010	0.002	4.5e-08	3.4e-05	31.5
Adiposity Markers	BMI	rs6962185	7	93135055	A	G	0.013	0.001	1.1e-20	9.1e-05	90.2
Adiposity Markers	BMI	rs10271989	7	131606162	A	G	-0.010	0.002	5.5e-09	3.6e-05	33.2
Adiposity Markers	BMI	rs17149254	7	76634463	T	C	0.025	0.002	4.5e-36	1.8e-04	155.0
Adiposity Markers	BMI	rs16875962	7	32514776	T	C	0.009	0.002	4.8e-09	3.7e-05	34.4
Adiposity Markers	BMI	rs10275044	7	1273845	A	T	-0.012	0.002	4.1e-10	4.6e-05	39.2
Adiposity Markers	BMI	rs329269	7	35074130	C	G	0.010	0.002	5.0e-11	4.8e-05	44.4
Adiposity Markers	BMI	rs866279	7	6451481	A	G	-0.016	0.002	5.6e-12	5.0e-05	46.6
Adiposity Markers	BMI	rs7779853	7	39057741	A	G	-0.008	0.001	2.3e-08	3.2e-05	32.7
Adiposity Markers	BMI	rs2690722	7	46070751	C	G	-0.009	0.002	1.5e-09	3.7e-05	37.6
Adiposity Markers	BMI	rs11761324	7	77432914	A	G	-0.010	0.002	5.0e-10	4.0e-05	37.4
Adiposity Markers	BMI	rs9638713	7	14645949	A	G	0.031	0.005	4.2e-11	4.5e-05	44.5
Adiposity Markers	BMI	rs6947240	7	150657209	A	G	-0.013	0.002	2.2e-15	6.1e-05	62.1
Adiposity Markers	BMI	rs6462772	7	37591968	A	G	0.009	0.002	1.5e-09	3.9e-05	36.8
Adiposity Markers	BMI	rs550619961	7	42949132	T	G	3.568	0.652	4.4e-08	1.4e-04	30.0
Adiposity Markers	BMI	rs1030015	7	78139581	T	G	0.010	0.002	1.3e-11	4.9e-05	45.3
Adiposity Markers	BMI	rs10245306	7	158029340	C	G	0.010	0.002	4.8e-10	4.0e-05	37.4
Adiposity Markers	BMI	rs13240600	7	99064466	A	G	0.015	0.002	2.6e-14	5.9e-05	55.5
Adiposity Markers	BMI	rs587671008	7	74532707	A	G	-21.219	3.881	4.6e-08	1.4e-04	29.9
Adiposity Markers	BMI	rs849135	7	28196413	A	G	0.012	0.001	2.5e-16	6.7e-05	68.7
Adiposity Markers	BMI	rs12706005	7	114672181	T	C	-0.015	0.002	9.3e-11	4.4e-05	41.2
Adiposity Markers	BMI	rs2866719	7	70106061	T	C	0.010	0.002	4.4e-11	4.3e-05	44.4
Adiposity Markers	BMI	rs2021885	7	107627314	T	C	-0.009	0.002	6.7e-09	3.6e-05	33.6
Adiposity Markers	BMI	rs10499694	7	50614173	A	G	0.013	0.001	5.5e-19	7.9e-05	81.0
Adiposity Markers	BMI	rs1048303	7	100804140	T	C	-0.010	0.002	4.3e-11	4.7e-05	43.6
Adiposity Markers	BMI	rs10263780	7	19778086	A	G	-0.014	0.002	1.1e-09	4.5e-05	35.5
Adiposity Markers	BMI	rs2396625	7	113028634	A	T	-0.017	0.002	6.0e-30	1.4e-04	131.5
Adiposity Markers	BMI	rs4722398	7	3125220	T	C	0.015	0.002	3.3e-12	5.2e-05	48.4
Adiposity Markers	BMI	rs896183	7	127831580	A	G	0.009	0.002	9.1e-09	3.3e-05	33.6
Adiposity Markers	BMI	rs17145750	7	73026378	T	C	0.011	0.002	2.2e-08	3.0e-05	30.2
Adiposity Markers	BMI	rs972283	7	130466854	A	G	0.009	0.001	8.0e-10	3.8e-05	38.6
Adiposity Markers	BMI	rs2952635	7	157508424	A	G	-0.012	0.002	1.4e-10	4.5e-05	41.9

Phenotype	Trait	SNP	Chr	Position (GRCh37/hg19)	EA	NEA	BETA	SE	P-value	R2	F
Adiposity Markers	BMI	rs12534783	7	147662972	A	G	-0.012	0.002	1.3e-12	5.3e-05	49.0
Adiposity Markers	BMI	rs10236427	7	4379718	T	C	0.011	0.002	3.2e-09	3.9e-05	36.7
Adiposity Markers	BMI	rs4307239	7	24354300	A	G	-0.010	0.002	3.5e-12	5.2e-05	48.1
Adiposity Markers	BMI	rs11772246	7	71603692	T	C	0.015	0.002	3.6e-15	6.8e-05	63.2
Adiposity Markers	BMI	rs11767191	7	48817965	T	C	0.009	0.002	1.3e-08	3.4e-05	32.1
Adiposity Markers	BMI	rs1357685	7	109200331	T	C	0.009	0.002	1.4e-09	3.9e-05	36.8
Adiposity Markers	BMI	rs11971098	7	26699061	A	G	-0.016	0.003	1.6e-09	3.8e-05	35.6
Adiposity Markers	BMI	rs1852006	7	77829768	A	G	-0.016	0.002	2.4e-25	1.1e-04	103.8
Adiposity Markers	BMI	rs13227433	7	74094721	T	G	-0.015	0.002	2.0e-17	8.4e-05	75.8
Adiposity Markers	BMI	rs6463489	7	5542513	T	C	0.016	0.002	2.8e-12	4.9e-05	49.6
Adiposity Markers	BMI	rs17207196	7	75101065	T	C	-0.023	0.002	1.2e-51	2.6e-04	237.2
Adiposity Markers	BMI	rs799449	7	44784697	T	C	0.012	0.002	2.0e-16	7.3e-05	68.3
Adiposity Markers	BMI	rs2283093	7	126721231	T	C	0.011	0.002	6.4e-09	3.8e-05	35.3
Adiposity Markers	BMI	rs6461115	7	2103668	A	G	0.014	0.002	6.6e-16	6.3e-05	64.0
Adiposity Markers	BMI	rs485865	7	71734051	A	G	-0.015	0.002	5.2e-09	3.6e-05	33.6
Adiposity Markers	BMI	rs274628	7	86265855	A	C	-0.010	0.002	1.1e-10	4.3e-05	39.8
Adiposity Markers	BMI	rs10229027	7	69799863	T	C	-0.013	0.002	9.8e-15	6.3e-05	58.5
Adiposity Markers	BMI	rs6460898	7	12252893	A	G	-0.009	0.002	7.1e-09	3.6e-05	33.6
Adiposity Markers	BMI	rs6968554	7	17287106	A	G	-0.009	0.002	5.3e-09	3.4e-05	34.4
Adiposity Markers	BMI	rs1348442	7	121960407	T	G	-0.013	0.002	1.7e-17	7.0e-05	71.7
Adiposity Markers	BMI	rs2237403	7	39448936	T	C	-0.012	0.002	1.3e-14	6.3e-05	59.1
Adiposity Markers	BMI	rs199848680	7	74569925	A	G	-19.526	3.374	7.2e-09	1.6e-04	33.5
Adiposity Markers	BMI	rs2396679	7	113349793	C	G	-0.013	0.002	1.0e-17	7.8e-05	72.8
Adiposity Markers	BMI	rs215669	7	32378979	A	G	-0.013	0.002	9.0e-17	7.4e-05	69.4
Adiposity Markers	BMI	rs2299383	7	103418846	T	C	0.016	0.001	4.6e-29	1.3e-04	132.2
Adiposity Markers	BMI	rs1814170	7	138794149	A	T	0.018	0.002	5.4e-14	5.9e-05	54.8
Adiposity Markers	BMI	rs3800649	7	137424509	A	G	0.013	0.002	2.6e-14	6.0e-05	55.8
Adiposity Markers	BMI	rs2045293	7	114350102	T	C	0.014	0.002	2.0e-19	8.8e-05	82.2
Adiposity Markers	BMI	rs10236956	7	67649847	T	G	0.009	0.002	1.1e-09	4.0e-05	37.6
Adiposity Markers	BMI	rs10259490	7	49616420	A	G	-0.012	0.002	1.1e-14	6.4e-05	59.8
Adiposity Markers	BMI	rs1431659	8	73439070	A	G	0.018	0.002	1.6e-25	1.2e-04	108.4
Adiposity Markers	BMI	rs1394	8	9511654	A	G	-0.013	0.002	6.5e-19	7.9e-05	79.8
Adiposity Markers	BMI	rs7833077	8	124145256	C	G	-0.011	0.002	2.4e-11	4.6e-05	43.1
Adiposity Markers	BMI	rs1899898	8	93240419	T	C	0.012	0.002	3.9e-14	5.9e-05	55.3
Adiposity Markers	BMI	rs7004189	8	76600419	T	C	-0.011	0.002	3.3e-09	3.9e-05	36.0
Adiposity Markers	BMI	rs2952131	8	17010298	A	G	-0.008	0.001	1.6e-08	3.3e-05	33.5
Adiposity Markers	BMI	rs6988769	8	135810390	C	G	0.010	0.002	6.6e-10	3.9e-05	36.0
Adiposity Markers	BMI	rs881301	8	38332318	T	C	-0.009	0.002	1.2e-09	3.9e-05	36.8
Adiposity Markers	BMI	rs1461858	8	14386985	A	C	0.009	0.002	3.4e-08	3.2e-05	29.9
Adiposity Markers	BMI	rs10099330	8	143383694	A	G	-0.012	0.002	1.7e-14	6.3e-05	58.8
Adiposity Markers	BMI	rs2196618	8	85089437	A	G	-0.013	0.002	1.1e-15	6.7e-05	62.1
Adiposity Markers	BMI	rs6996603	8	28117924	T	C	0.012	0.002	3.1e-14	6.3e-05	58.8
Adiposity Markers	BMI	rs4831563	8	14096310	A	T	-0.013	0.002	5.9e-18	7.9e-05	74.0
Adiposity Markers	BMI	rs12550299	8	6325442	C	G	0.014	0.002	6.7e-10	4.0e-05	37.7
Adiposity Markers	BMI	rs2959592	8	17002725	T	C	0.014	0.002	1.4e-14	5.8e-05	57.9
Adiposity Markers	BMI	rs17326685	8	3564244	A	G	-0.009	0.002	6.3e-09	3.5e-05	32.9
Adiposity Markers	BMI	rs1658820	8	4288577	T	G	0.011	0.002	8.6e-10	3.9e-05	36.0
Adiposity Markers	BMI	rs800537	8	116721984	T	C	0.017	0.002	2.0e-24	1.1e-04	101.2
Adiposity Markers	BMI	rs11781699	8	118863061	T	C	-0.013	0.002	4.0e-12	5.0e-05	50.6
Adiposity Markers	BMI	rs6469351	8	112353205	T	C	0.011	0.002	2.3e-11	4.6e-05	43.1
Adiposity Markers	BMI	rs17069831	8	4137396	T	C	-0.010	0.002	7.8e-10	4.0e-05	36.7
Adiposity Markers	BMI	rs11781222	8	23389571	T	C	0.017	0.002	2.7e-16	6.7e-05	68.7
Adiposity Markers	BMI	rs7003816	8	106433801	A	G	-0.011	0.002	5.2e-10	3.9e-05	36.7
Adiposity Markers	BMI	rs16907771	8	81415556	T	G	0.033	0.004	1.4e-16	7.5e-05	69.3
Adiposity Markers	BMI	rs6472125	8	64752533	A	G	0.011	0.002	2.4e-13	5.7e-05	52.8
Adiposity Markers	BMI	rs16906845	8	138215228	A	G	-0.024	0.003	3.9e-15	6.6e-05	61.4
Adiposity Markers	BMI	rs2059527	8	25654048	A	T	-0.009	0.002	8.0e-09	3.5e-05	32.3
Adiposity Markers	BMI	rs10081605	8	2936443	A	G	0.009	0.002	1.1e-08	3.4e-05	31.6
Adiposity Markers	BMI	rs330064	8	9163534	T	C	-0.011	0.002	4.4e-13	5.6e-05	51.8
Adiposity Markers	BMI	rs1982441	8	28021769	T	G	0.017	0.002	3.9e-14	6.2e-05	57.6
Adiposity Markers	BMI	rs12546331	8	87505968	T	C	0.010	0.002	1.0e-11	4.9e-05	45.3
Adiposity Markers	BMI	rs12545740	8	33455300	A	C	0.010	0.002	6.4e-12	5.1e-05	47.2

Phenotype	Trait	SNP	Chr	Position (GRCh37/hg19)	EA	NEA	BETA	SE	P-value	R2	F
Adiposity Markers	BMI	rs4599845	8	116593598	T	C	0.011	0.001	4.0e-14	5.8e-05	59.5
Adiposity Markers	BMI	rs12681792	8	62054463	A	C	0.014	0.002	3.3e-14	6.0e-05	55.9
Adiposity Markers	BMI	rs4917229	8	143130579	T	C	0.008	0.002	2.7e-08	3.3e-05	30.6
Adiposity Markers	BMI	rs12675063	8	132879047	A	T	-0.016	0.002	2.1e-12	4.9e-05	50.3
Adiposity Markers	BMI	rs4299971	8	105338752	A	G	-0.009	0.002	1.2e-09	3.9e-05	36.8
Adiposity Markers	BMI	rs2622555	8	56290486	T	C	0.012	0.002	9.0e-09	3.6e-05	33.2
Adiposity Markers	BMI	rs2954021	8	126482077	A	G	-0.012	0.002	3.5e-15	6.5e-05	60.8
Adiposity Markers	BMI	rs2616192	8	20668624	T	G	0.012	0.002	1.5e-13	5.7e-05	53.5
Adiposity Markers	BMI	rs7837587	8	8378992	T	C	-0.014	0.001	1.4e-22	9.6e-05	98.6
Adiposity Markers	BMI	rs4733037	8	27093495	T	C	0.009	0.002	3.2e-09	3.6e-05	33.8
Adiposity Markers	BMI	rs4240673	8	10787612	T	C	0.015	0.001	3.5e-27	1.2e-04	121.0
Adiposity Markers	BMI	rs4269528	8	67197341	T	G	0.013	0.002	2.0e-14	6.1e-05	56.7
Adiposity Markers	BMI	rs4841659	8	11828200	T	C	0.013	0.002	5.4e-17	7.4e-05	69.4
Adiposity Markers	BMI	rs305289	8	137587070	T	C	0.011	0.002	1.6e-10	4.2e-05	39.4
Adiposity Markers	BMI	rs2994298	8	131342873	A	G	0.010	0.002	2.6e-08	3.2e-05	30.2
Adiposity Markers	BMI	rs1362910	8	30856464	A	G	0.013	0.002	4.9e-18	7.7e-05	76.3
Adiposity Markers	BMI	rs12680842	8	95582606	A	G	0.015	0.002	1.0e-23	1.0e-04	101.3
Adiposity Markers	BMI	rs13265882	8	15565257	T	C	-0.010	0.002	2.0e-11	4.9e-05	45.3
Adiposity Markers	BMI	rs1351724	8	85519223	T	G	-0.012	0.002	5.4e-12	4.9e-05	45.9
Adiposity Markers	BMI	rs10808810	8	77235096	T	C	-0.019	0.002	1.9e-36	1.7e-04	157.1
Adiposity Markers	BMI	rs17316246	8	74749152	A	T	0.015	0.002	1.7e-10	4.3e-05	40.3
Adiposity Markers	BMI	rs10504290	8	60153096	A	G	0.013	0.002	1.7e-10	4.4e-05	40.7
Adiposity Markers	BMI	rs4739235	8	21287105	A	G	-0.012	0.002	3.6e-09	3.9e-05	36.0
Adiposity Markers	BMI	rs2978563	8	62216873	C	G	0.010	0.002	1.9e-10	4.3e-05	39.8
Adiposity Markers	BMI	rs7826312	8	32400115	T	C	-0.012	0.001	1.3e-16	6.9e-05	71.0
Adiposity Markers	BMI	rs1470764	8	101950038	A	G	0.010	0.002	5.5e-12	5.0e-05	47.2
Adiposity Markers	BMI	rs2174367	8	89408196	T	G	-0.010	0.002	6.6e-10	4.0e-05	37.5
Adiposity Markers	BMI	rs1350558	8	34186054	A	G	-0.011	0.002	4.3e-13	5.4e-05	50.8
Adiposity Markers	BMI	rs10104250	8	23431192	T	G	-0.017	0.003	5.8e-09	3.7e-05	34.4
Adiposity Markers	BMI	rs10756260	9	11585651	T	C	-0.009	0.002	1.6e-08	3.3e-05	30.6
Adiposity Markers	BMI	rs7023745	9	10408553	T	C	-0.012	0.002	7.0e-09	3.7e-05	33.8
Adiposity Markers	BMI	rs1484375	9	109067561	A	G	0.013	0.002	7.5e-13	5.2e-05	49.0
Adiposity Markers	BMI	rs10733682	9	129460914	A	G	0.015	0.001	1.3e-24	1.1e-04	110.2
Adiposity Markers	BMI	rs6477694	9	111932342	T	C	-0.012	0.002	1.6e-16	6.7e-05	68.3
Adiposity Markers	BMI	rs3922980	9	27803737	T	G	-0.013	0.002	1.9e-17	7.7e-05	71.7
Adiposity Markers	BMI	rs10812830	9	28446608	T	C	0.016	0.002	9.0e-16	6.0e-05	61.7
Adiposity Markers	BMI	rs4741188	9	11887262	C	G	0.011	0.002	5.6e-14	6.1e-05	56.8
Adiposity Markers	BMI	rs7042263	9	127039017	T	C	0.010	0.002	6.8e-12	5.0e-05	47.2
Adiposity Markers	BMI	rs483752	9	27612918	T	C	0.010	0.002	1.2e-08	4.3e-05	33.4
Adiposity Markers	BMI	rs449807	9	118965749	A	T	0.009	0.002	3.6e-10	4.2e-05	39.3
Adiposity Markers	BMI	rs1154659	9	28906917	A	G	-0.014	0.002	6.9e-14	6.3e-05	58.8
Adiposity Markers	BMI	rs7042372	9	6959840	A	G	0.012	0.002	3.1e-13	5.6e-05	52.6
Adiposity Markers	BMI	rs10735567	9	86712580	A	G	0.009	0.002	7.1e-09	3.6e-05	32.9
Adiposity Markers	BMI	rs1853413	9	1642998	C	G	0.010	0.002	3.8e-10	4.1e-05	38.3
Adiposity Markers	BMI	rs7039543	9	122920438	T	C	-0.009	0.002	1.8e-08	3.3e-05	30.6
Adiposity Markers	BMI	rs10818839	9	126324625	A	G	0.013	0.002	1.4e-09	3.9e-05	36.5
Adiposity Markers	BMI	rs1187352	9	87293457	T	C	-0.012	0.002	1.1e-13	5.7e-05	53.5
Adiposity Markers	BMI	rs12380880	9	88912765	T	C	-0.013	0.002	2.4e-12	5.2e-05	48.2
Adiposity Markers	BMI	rs10757893	9	29599001	A	G	0.010	0.002	3.7e-11	4.4e-05	44.4
Adiposity Markers	BMI	rs10760279	9	126105291	T	G	0.012	0.002	6.5e-15	6.3e-05	58.1
Adiposity Markers	BMI	rs10738374	9	14645900	C	G	0.009	0.002	5.7e-09	3.6e-05	33.6
Adiposity Markers	BMI	rs4989244	9	102100348	A	G	-0.009	0.002	9.3e-10	4.0e-05	37.6
Adiposity Markers	BMI	rs4878458	9	31003975	T	C	-0.012	0.002	4.4e-10	4.3e-05	39.9
Adiposity Markers	BMI	rs2418393	9	118390987	A	G	-0.011	0.002	1.3e-11	4.6e-05	44.2
Adiposity Markers	BMI	rs17277467	9	13939884	T	C	-0.014	0.002	3.8e-14	6.2e-05	57.4
Adiposity Markers	BMI	rs580809	9	101518442	T	C	-0.010	0.002	1.8e-09	3.5e-05	35.3
Adiposity Markers	BMI	rs7033259	9	20780016	A	T	-0.009	0.002	1.3e-09	3.9e-05	36.8
Adiposity Markers	BMI	rs4740619	9	15634326	T	C	0.018	0.001	2.1e-35	1.5e-04	158.0
Adiposity Markers	BMI	rs716387	9	73900251	T	C	0.011	0.002	6.9e-13	5.5e-05	51.8
Adiposity Markers	BMI	rs2570306	9	8843382	C	G	-0.010	0.002	1.4e-08	3.3e-05	30.9
Adiposity Markers	BMI	rs10984756	9	122651784	C	G	-0.016	0.002	2.6e-10	4.5e-05	41.7
Adiposity Markers	BMI	rs3025360	9	136481205	A	G	-0.014	0.002	7.4e-09	3.3e-05	32.6

Phenotype	Trait	SNP	Chr	Position (GRCh37/hg19)	EA	NEA	BETA	SE	P-value	R2	F
Adiposity Markers	BMI	rs12337316	9	37148105	A	G	-0.014	0.002	1.0e-20	9.5e-05	88.4
Adiposity Markers	BMI	rs2378100	9	80513323	T	C	0.009	0.002	7.4e-09	3.6e-05	33.6
Adiposity Markers	BMI	rs12555274	9	22136440	C	G	-0.021	0.004	7.6e-09	1.5e-04	33.1
Adiposity Markers	BMI	rs4740383	9	133783566	A	G	0.014	0.002	3.8e-18	7.9e-05	72.2
Adiposity Markers	BMI	rs7018588	9	96382449	A	G	0.012	0.002	1.2e-10	4.4e-05	40.6
Adiposity Markers	BMI	rs7863807	9	124625886	A	G	-0.009	0.002	1.7e-08	3.3e-05	30.9
Adiposity Markers	BMI	rs7045925	9	96475306	T	C	0.013	0.002	8.2e-16	6.4e-05	65.0
Adiposity Markers	BMI	rs10757259	9	2198033	A	C	-0.010	0.002	1.7e-08	3.7e-05	33.2
Adiposity Markers	BMI	rs4237263	9	76818745	T	C	0.010	0.002	1.8e-09	3.8e-05	35.3
Adiposity Markers	BMI	rs10118866	9	10119157	T	G	0.012	0.002	7.5e-12	4.8e-05	44.4
Adiposity Markers	BMI	rs10989574	9	104399139	T	C	-0.010	0.002	1.1e-10	4.4e-05	41.0
Adiposity Markers	BMI	rs7852958	9	138015521	T	C	0.010	0.002	2.1e-09	3.6e-05	33.9
Adiposity Markers	BMI	rs6477515	9	109184462	T	C	0.008	0.002	4.4e-08	3.2e-05	29.9
Adiposity Markers	BMI	rs10448285	9	129397014	T	C	0.010	0.002	2.4e-10	4.2e-05	39.1
Adiposity Markers	BMI	rs11792069	9	140646121	A	G	0.014	0.002	1.0e-11	5.3e-05	46.9
Adiposity Markers	BMI	rs7026534	9	134907263	T	G	0.010	0.002	1.2e-09	3.9e-05	36.8
Adiposity Markers	BMI	rs7871866	9	131027982	C	G	0.018	0.002	4.2e-18	8.3e-05	76.8
Adiposity Markers	BMI	rs995804	9	118644810	A	G	-0.009	0.002	4.1e-10	4.1e-05	38.4
Adiposity Markers	BMI	rs12552443	9	120663383	A	G	0.010	0.002	1.4e-11	4.9e-05	45.3
Adiposity Markers	BMI	rs10962549	9	16719445	T	C	0.018	0.002	1.6e-18	8.2e-05	76.6
Adiposity Markers	BMI	rs684639	9	79312579	A	T	-0.010	0.002	4.6e-12	5.1e-05	48.1
Adiposity Markers	BMI	rs10990303	9	98410405	T	C	-0.010	0.002	3.3e-09	3.3e-05	33.4
Adiposity Markers	BMI	rs13296052	9	104047273	A	G	-0.009	0.002	1.3e-08	3.4e-05	31.6
Adiposity Markers	BMI	rs10991925	9	94177640	T	C	0.013	0.002	3.9e-14	5.9e-05	54.9
Adiposity Markers	BMI	rs1999433	9	81371441	T	C	-0.011	0.002	8.3e-14	6.0e-05	55.8
Adiposity Markers	BMI	rs7030895	9	92189513	A	C	-0.012	0.002	1.0e-15	6.9e-05	64.0
Adiposity Markers	BMI	rs7031064	9	14455076	A	G	0.010	0.002	3.4e-11	4.6e-05	42.7
Adiposity Markers	BMI	rs9407671	9	15882615	A	T	-0.010	0.002	6.6e-09	3.5e-05	32.7
Adiposity Markers	BMI	rs4743395	9	103117247	C	G	-0.016	0.002	1.5e-23	1.0e-04	96.3
Adiposity Markers	BMI	rs1928295	9	120378483	T	C	0.014	0.001	1.1e-22	9.6e-05	98.6
Adiposity Markers	BMI	rs10971712	9	33820938	T	C	-0.022	0.002	1.7e-20	9.0e-05	84.0
Adiposity Markers	BMI	rs11138313	9	82240968	A	G	0.017	0.002	1.4e-12	5.3e-05	48.4
Adiposity Markers	BMI	rs2482080	9	117869684	T	G	-0.011	0.002	2.0e-13	5.8e-05	53.8
Adiposity Markers	BMI	rs10968576	9	28414339	A	G	-0.024	0.002	6.4e-58	2.5e-04	260.3
Adiposity Markers	BMI	rs10965780	9	23341715	C	G	-0.012	0.002	2.1e-14	6.3e-05	58.8
Adiposity Markers	BMI	rs10867775	9	84183227	A	C	0.011	0.002	2.0e-12	5.2e-05	48.1
Adiposity Markers	BMI	rs10828315	10	22835923	C	G	-0.009	0.002	2.9e-08	3.2e-05	30.2
Adiposity Markers	BMI	rs11017772	10	132954247	T	C	-0.011	0.002	1.4e-08	3.6e-05	34.0
Adiposity Markers	BMI	rs6585419	10	118556539	A	G	-0.009	0.002	6.4e-09	3.6e-05	33.6
Adiposity Markers	BMI	rs10761785	10	65318766	T	G	-0.011	0.001	3.3e-15	6.3e-05	64.0
Adiposity Markers	BMI	rs12218544	10	124947286	C	G	-0.015	0.002	3.5e-10	4.3e-05	40.1
Adiposity Markers	BMI	rs11201987	10	88111573	A	G	-0.012	0.002	3.4e-09	3.9e-05	36.0
Adiposity Markers	BMI	rs12259464	10	53680099	A	G	0.010	0.002	1.0e-11	4.9e-05	45.3
Adiposity Markers	BMI	rs10752309	10	13562076	T	C	0.011	0.002	3.7e-09	3.7e-05	34.1
Adiposity Markers	BMI	rs12411886	10	104685299	A	C	0.032	0.003	4.4e-35	1.4e-04	147.7
Adiposity Markers	BMI	rs12762034	10	33969931	T	C	-0.022	0.003	3.5e-15	6.7e-05	62.3
Adiposity Markers	BMI	rs4919556	10	103333271	T	C	0.011	0.002	7.0e-13	5.4e-05	50.9
Adiposity Markers	BMI	rs7084454	10	21821274	A	G	0.020	0.002	3.5e-36	1.7e-04	153.1
Adiposity Markers	BMI	rs11199264	10	122057157	A	G	-0.011	0.002	3.4e-09	3.3e-05	33.5
Adiposity Markers	BMI	rs2631681	10	93032943	T	C	-0.009	0.002	4.6e-09	3.4e-05	34.4
Adiposity Markers	BMI	rs1250552	10	81058027	A	G	-0.009	0.001	5.8e-10	3.9e-05	39.5
Adiposity Markers	BMI	rs529196140	10	104949907	A	G	11.437	1.932	3.2e-09	1.6e-04	35.1
Adiposity Markers	BMI	rs7087215	10	131120672	T	G	0.009	0.002	4.6e-08	3.2e-05	29.6
Adiposity Markers	BMI	rs7903146	10	114758349	T	C	-0.023	0.002	3.2e-48	2.0e-04	208.4
Adiposity Markers	BMI	rs7893571	10	16750129	T	G	0.011	0.002	2.3e-11	4.7e-05	43.9
Adiposity Markers	BMI	rs11001296	10	76873427	T	C	-0.011	0.002	6.7e-10	4.2e-05	39.4
Adiposity Markers	BMI	rs17636031	10	126594078	T	C	-0.015	0.002	7.8e-21	8.7e-05	87.9
Adiposity Markers	BMI	rs7899106	10	87410904	A	G	-0.034	0.003	1.5e-24	1.0e-04	103.7
Adiposity Markers	BMI	rs17113518	10	102620555	T	C	0.016	0.002	7.9e-12	4.6e-05	46.7
Adiposity Markers	BMI	rs845084	10	125220036	A	G	0.014	0.002	7.5e-16	6.8e-05	63.1
Adiposity Markers	BMI	rs9418706	10	128822842	A	G	-0.011	0.002	2.7e-08	3.4e-05	29.5
Adiposity Markers	BMI	rs3004929	10	34414865	A	G	0.009	0.002	2.1e-09	3.9e-05	36.0

Phenotype	Trait	SNP	Chr	Position (GRCh37/hg19)	EA	NEA	BETA	SE	P-value	R2	F
Adiposity Markers	BMI	rs11001963	10	78760959	T	C	0.011	0.002	4.1e-13	5.7e-05	52.8
Adiposity Markers	BMI	rs1993414	10	63403235	T	G	0.017	0.003	9.5e-09	3.3e-05	33.3
Adiposity Markers	BMI	rs17094222	10	102395440	T	C	-0.019	0.002	6.5e-26	1.0e-04	106.8
Adiposity Markers	BMI	rs4880341	10	133992689	T	C	-0.014	0.002	1.5e-20	9.2e-05	85.9
Adiposity Markers	BMI	rs12776880	10	19776828	A	T	0.011	0.002	1.8e-10	4.2e-05	38.9
Adiposity Markers	BMI	rs6602411	10	10264200	T	C	0.012	0.002	3.3e-09	3.9e-05	36.6
Adiposity Markers	BMI	rs10752040	10	16111499	A	C	-0.011	0.002	1.7e-10	4.2e-05	39.4
Adiposity Markers	BMI	rs7087685	10	118616537	A	G	0.013	0.002	1.0e-15	6.7e-05	62.1
Adiposity Markers	BMI	rs12779707	10	115857076	T	C	0.011	0.002	8.2e-10	3.9e-05	36.7
Adiposity Markers	BMI	rs7089920	10	120489545	T	C	-0.015	0.002	7.2e-10	4.0e-05	37.0
Adiposity Markers	BMI	rs10827381	10	34850203	A	G	-0.010	0.002	2.3e-11	4.8e-05	44.4
Adiposity Markers	BMI	rs12411523	10	99862731	T	C	-0.016	0.002	4.3e-23	1.1e-04	98.8
Adiposity Markers	BMI	rs12254400	10	18555510	A	C	0.014	0.002	4.1e-10	4.1e-05	38.2
Adiposity Markers	BMI	rs10824218	10	76421216	A	T	0.011	0.002	5.4e-14	6.1e-05	56.8
Adiposity Markers	BMI	rs575498477	10	105102102	T	C	10.285	1.870	3.8e-08	1.4e-04	30.2
Adiposity Markers	BMI	rs942093	10	77626488	C	G	-0.012	0.002	8.8e-14	5.7e-05	53.2
Adiposity Markers	BMI	rs7903784	10	77219944	T	C	-0.011	0.002	1.7e-08	3.5e-05	32.9
Adiposity Markers	BMI	rs10829164	10	27318370	T	C	0.014	0.002	7.9e-12	5.0e-05	47.0
Adiposity Markers	BMI	rs4929923	11	8639200	T	C	-0.017	0.002	7.4e-30	1.3e-04	128.4
Adiposity Markers	BMI	rs7122903	11	129305889	T	C	0.011	0.002	1.1e-09	3.8e-05	35.3
Adiposity Markers	BMI	rs7124681	11	47529947	A	C	0.026	0.001	1.6e-74	3.4e-04	347.6
Adiposity Markers	BMI	rs11226914	11	105941504	T	G	-0.034	0.006	2.8e-08	3.4e-05	30.7
Adiposity Markers	BMI	rs477895	11	64048912	T	C	0.014	0.002	1.6e-12	5.4e-05	50.4
Adiposity Markers	BMI	rs10840606	11	2234690	A	G	-0.015	0.002	2.6e-14	6.6e-05	59.3
Adiposity Markers	BMI	rs7950748	11	122762432	A	T	-0.011	0.002	1.6e-09	3.7e-05	34.7
Adiposity Markers	BMI	rs11227306	11	65578672	A	C	0.014	0.002	8.4e-21	9.5e-05	88.4
Adiposity Markers	BMI	rs2509324	11	94245740	A	G	-0.008	0.002	2.6e-08	3.3e-05	30.6
Adiposity Markers	BMI	rs7123876	11	72444583	T	C	-0.011	0.002	1.8e-10	3.9e-05	39.6
Adiposity Markers	BMI	rs1378529	11	106877375	T	C	0.009	0.002	3.8e-10	4.2e-05	39.3
Adiposity Markers	BMI	rs6591407	11	56914157	A	C	-0.011	0.002	1.9e-08	3.0e-05	30.5
Adiposity Markers	BMI	rs10892501	11	119818622	A	G	-0.010	0.002	4.7e-10	4.0e-05	37.5
Adiposity Markers	BMI	rs1789167	11	69486244	A	G	-0.015	0.002	1.4e-21	9.7e-05	89.1
Adiposity Markers	BMI	rs2440885	11	70563286	A	G	0.009	0.002	9.9e-10	3.9e-05	36.8
Adiposity Markers	BMI	rs2510709	11	127032222	A	G	0.008	0.002	3.8e-08	3.2e-05	29.9
Adiposity Markers	BMI	rs10894670	11	133221987	A	C	-0.009	0.001	3.2e-10	4.0e-05	41.3
Adiposity Markers	BMI	rs7118690	11	87486602	A	G	-0.010	0.002	1.8e-09	3.8e-05	35.3
Adiposity Markers	BMI	rs7091	11	866794	A	G	0.010	0.002	1.1e-11	5.0e-05	46.2
Adiposity Markers	BMI	rs893006	11	64365796	A	C	0.015	0.002	1.4e-20	8.4e-05	85.6
Adiposity Markers	BMI	rs10838464	11	45425995	T	C	-0.012	0.002	7.9e-14	5.8e-05	54.4
Adiposity Markers	BMI	rs11024614	11	18326758	T	C	0.010	0.002	2.4e-11	4.8e-05	44.4
Adiposity Markers	BMI	rs1765131	11	30404538	C	G	-0.015	0.002	2.1e-21	9.4e-05	87.9
Adiposity Markers	BMI	rs654829	11	69306395	A	T	-0.012	0.002	9.1e-14	6.4e-05	59.1
Adiposity Markers	BMI	rs7944782	11	130795698	T	G	-0.014	0.002	1.2e-19	8.8e-05	82.2
Adiposity Markers	BMI	rs2862996	11	43653833	T	G	-0.019	0.002	5.9e-38	1.6e-04	167.3
Adiposity Markers	BMI	rs11030104	11	27684517	A	G	0.038	0.002	1.4e-103	4.4e-04	450.4
Adiposity Markers	BMI	rs11533200	11	131955594	A	G	-0.013	0.002	1.7e-14	6.1e-05	56.7
Adiposity Markers	BMI	rs1318241	11	77930792	T	C	-0.012	0.002	2.4e-09	3.6e-05	37.3
Adiposity Markers	BMI	rs2155645	11	112912947	T	C	-0.011	0.002	1.0e-11	4.4e-05	45.0
Adiposity Markers	BMI	rs7120873	11	49459474	T	C	0.016	0.002	4.6e-12	5.1e-05	47.8
Adiposity Markers	BMI	rs1400759	11	110965115	A	C	-0.009	0.002	8.5e-09	3.5e-05	32.3
Adiposity Markers	BMI	rs570463	11	28739318	A	C	-0.014	0.002	1.0e-17	7.7e-05	72.2
Adiposity Markers	BMI	rs1440480	11	134290032	A	G	-0.009	0.002	1.2e-08	3.5e-05	31.6
Adiposity Markers	BMI	rs10736605	11	132693854	A	G	-0.009	0.002	8.5e-10	4.0e-05	37.6
Adiposity Markers	BMI	rs1440978	11	76476280	T	C	-0.011	0.002	1.9e-09	3.7e-05	34.1
Adiposity Markers	BMI	rs349088	11	84814393	A	C	-0.012	0.002	1.1e-15	6.9e-05	64.0
Adiposity Markers	BMI	rs10741329	11	89997796	A	G	0.012	0.002	4.8e-14	6.0e-05	56.2
Adiposity Markers	BMI	rs636049	11	68667198	A	C	-0.010	0.002	8.9e-10	3.6e-05	36.8
Adiposity Markers	BMI	rs2054117	11	11796897	T	G	-0.010	0.002	1.1e-10	4.4e-05	41.0
Adiposity Markers	BMI	rs6578300	11	2876284	A	G	-0.010	0.002	1.6e-10	4.0e-05	40.6
Adiposity Markers	BMI	rs17120344	11	116996345	A	G	0.018	0.003	5.7e-09	3.5e-05	33.1
Adiposity Markers	BMI	rs10832642	11	16566705	T	G	0.012	0.002	2.3e-10	4.5e-05	42.2
Adiposity Markers	BMI	rs12364470	11	134601012	T	G	-0.017	0.002	1.1e-18	8.1e-05	81.9

Phenotype	Trait	SNP	Chr	Position (GRCh37/hg19)	EA	NEA	BETA	SE	P-value	R2	F
Adiposity Markers	BMI	rs11220135	11	125451422	A	G	-0.012	0.002	4.6e-09	3.7e-05	34.2
Adiposity Markers	BMI	rs10832778	11	17394073	C	G	-0.013	0.002	2.3e-17	7.1e-05	71.7
Adiposity Markers	BMI	rs573455	11	117267884	A	G	-0.008	0.001	9.4e-09	3.3e-05	33.5
Adiposity Markers	BMI	rs7127212	11	118953202	T	C	0.013	0.002	2.0e-17	7.7e-05	71.7
Adiposity Markers	BMI	rs2512885	11	131467794	T	C	-0.011	0.001	8.0e-16	6.5e-05	66.3
Adiposity Markers	BMI	rs9326370	11	65992008	T	C	0.009	0.002	2.9e-09	3.8e-05	35.2
Adiposity Markers	BMI	rs1048932	11	115044850	A	C	-0.017	0.001	4.1e-31	1.4e-04	140.6
Adiposity Markers	BMI	rs2367873	11	116376840	A	G	-0.012	0.002	6.6e-10	4.5e-05	39.2
Adiposity Markers	BMI	rs4757142	11	13325695	A	G	0.015	0.002	5.4e-23	1.0e-04	97.4
Adiposity Markers	BMI	rs7933205	11	97831073	A	G	-0.010	0.002	4.3e-09	3.5e-05	32.7
Adiposity Markers	BMI	rs6277	11	113283459	A	G	-0.010	0.002	1.6e-11	5.1e-05	44.4
Adiposity Markers	BMI	rs7120113	11	46934369	T	C	0.017	0.002	7.0e-12	4.5e-05	46.2
Adiposity Markers	BMI	rs2605603	11	93221105	A	G	-0.011	0.001	4.6e-14	5.7e-05	58.4
Adiposity Markers	BMI	rs1973716	11	43946579	C	G	0.013	0.002	4.6e-12	5.0e-05	46.8
Adiposity Markers	BMI	rs7950488	11	121902073	T	C	-0.012	0.002	1.1e-14	6.2e-05	58.1
Adiposity Markers	BMI	rs4923597	11	29250910	A	G	-0.013	0.002	1.9e-17	7.7e-05	71.7
Adiposity Markers	BMI	rs7938266	11	14709324	A	G	0.008	0.001	3.0e-09	3.5e-05	36.0
Adiposity Markers	BMI	rs2513670	11	114962918	C	G	0.011	0.002	1.6e-09	3.7e-05	34.7
Adiposity Markers	BMI	rs198448	11	61500487	T	C	-0.009	0.002	4.5e-09	3.7e-05	34.4
Adiposity Markers	BMI	rs6589939	11	122518525	A	G	-0.010	0.002	8.1e-12	4.9e-05	46.2
Adiposity Markers	BMI	rs11246136	11	371265	A	C	-0.019	0.003	1.5e-13	6.1e-05	53.4
Adiposity Markers	BMI	rs7480395	11	133667674	A	G	-0.009	0.002	5.4e-09	3.6e-05	33.6
Adiposity Markers	BMI	rs223058	11	32125855	A	G	-0.011	0.002	2.9e-11	4.6e-05	43.1
Adiposity Markers	BMI	rs4936511	11	120150059	A	C	0.010	0.002	3.9e-08	3.1e-05	28.8
Adiposity Markers	BMI	rs7975187	12	60964108	A	G	-0.011	0.002	1.1e-09	4.1e-05	38.7
Adiposity Markers	BMI	rs11056875	12	16470918	T	C	0.013	0.002	4.4e-09	3.8e-05	35.4
Adiposity Markers	BMI	rs651548	12	99560183	A	G	0.013	0.002	5.8e-16	6.9e-05	64.0
Adiposity Markers	BMI	rs730560	12	57938565	A	G	0.010	0.002	6.4e-11	4.3e-05	43.6
Adiposity Markers	BMI	rs2470397	12	2106116	T	C	-0.012	0.002	3.1e-10	4.3e-05	40.6
Adiposity Markers	BMI	rs1426449	12	114066082	A	G	0.010	0.002	2.7e-09	4.0e-05	37.5
Adiposity Markers	BMI	rs11066871	12	114432613	T	C	-0.019	0.003	3.0e-10	4.2e-05	39.3
Adiposity Markers	BMI	rs1819844	12	68205604	A	G	0.014	0.002	9.5e-14	5.3e-05	54.3
Adiposity Markers	BMI	rs822688	12	53493387	T	C	0.012	0.002	3.8e-08	3.3e-05	30.8
Adiposity Markers	BMI	rs2657879	12	56865338	A	G	0.010	0.002	1.7e-08	3.0e-05	30.0
Adiposity Markers	BMI	rs12831874	12	17774918	T	G	-0.014	0.002	4.9e-09	3.7e-05	34.5
Adiposity Markers	BMI	rs1038196	12	66343400	C	G	-0.009	0.002	7.4e-09	3.6e-05	33.6
Adiposity Markers	BMI	rs3741989	12	113646310	C	G	0.017	0.003	6.3e-09	3.6e-05	34.0
Adiposity Markers	BMI	rs11170468	12	39430048	A	C	0.014	0.002	1.2e-16	6.7e-05	68.8
Adiposity Markers	BMI	rs12316047	12	108393845	A	G	-0.015	0.002	4.2e-18	7.8e-05	73.2
Adiposity Markers	BMI	rs10777193	12	90142883	A	G	0.012	0.002	3.3e-12	4.6e-05	46.6
Adiposity Markers	BMI	rs2359979	12	62260866	A	C	0.011	0.002	1.0e-09	3.8e-05	35.3
Adiposity Markers	BMI	rs1371088	12	90660733	A	G	0.011	0.002	8.2e-11	4.3e-05	40.1
Adiposity Markers	BMI	rs7961979	12	121671261	A	C	0.016	0.002	6.0e-12	4.9e-05	46.0
Adiposity Markers	BMI	rs3768	12	124499839	T	C	0.016	0.002	3.0e-16	7.4e-05	69.2
Adiposity Markers	BMI	rs3730074	12	49167683	A	C	0.013	0.002	4.1e-10	4.4e-05	40.7
Adiposity Markers	BMI	rs11614340	12	133426483	T	C	-0.010	0.002	1.0e-10	4.4e-05	41.4
Adiposity Markers	BMI	rs11115176	12	82465797	T	C	0.012	0.002	3.5e-12	4.7e-05	48.2
Adiposity Markers	BMI	rs2272002	12	53817916	A	T	0.013	0.002	2.5e-10	4.5e-05	41.6
Adiposity Markers	BMI	rs11168936	12	49645240	T	C	-0.011	0.002	1.1e-12	5.3e-05	49.9
Adiposity Markers	BMI	rs12422552	12	14413931	C	G	-0.012	0.002	2.6e-12	5.1e-05	47.4
Adiposity Markers	BMI	rs10745888	12	100852410	A	G	0.025	0.004	2.1e-08	3.2e-05	31.6
Adiposity Markers	BMI	rs2269828	12	47471439	A	G	-0.009	0.002	1.6e-08	3.1e-05	31.6
Adiposity Markers	BMI	rs1488074	12	16902621	T	C	0.011	0.002	5.4e-12	4.6e-05	47.3
Adiposity Markers	BMI	rs2160428	12	97588490	T	C	0.009	0.002	4.1e-08	3.0e-05	30.2
Adiposity Markers	BMI	rs17709991	12	116065057	T	C	-0.010	0.002	7.7e-10	3.7e-05	37.5
Adiposity Markers	BMI	rs1562131	12	23587505	A	C	-0.012	0.002	3.4e-10	4.1e-05	37.9
Adiposity Markers	BMI	rs2712643	12	99638276	A	C	-0.012	0.002	1.9e-12	4.8e-05	49.0
Adiposity Markers	BMI	rs4357727	12	28444922	T	C	-0.010	0.002	8.1e-09	4.6e-05	35.3
Adiposity Markers	BMI	rs4148866	12	123425575	T	C	0.009	0.002	1.4e-08	3.3e-05	30.2
Adiposity Markers	BMI	rs2970843	12	19232231	A	G	-0.012	0.002	8.7e-12	4.8e-05	45.2
Adiposity Markers	BMI	rs2891403	12	113137572	A	G	-0.013	0.002	5.5e-15	6.0e-05	61.0
Adiposity Markers	BMI	rs6606686	12	110903380	C	G	-0.015	0.002	3.7e-23	9.8e-05	100.0

Phenotype	Trait	SNP	Chr	Position (GRCh37/hg19)	EA	NEA	BETA	SE	P-value	R2	F
Adiposity Markers	BMI	rs2279574	12	89745477	A	C	-0.013	0.002	1.1e-18	8.6e-05	76.3
Adiposity Markers	BMI	rs11045237	12	20579083	A	C	-0.011	0.002	2.1e-08	3.5e-05	31.7
Adiposity Markers	BMI	rs2733287	12	41880909	A	C	-0.016	0.002	3.3e-26	1.2e-04	111.0
Adiposity Markers	BMI	rs11611246	12	939480	T	G	0.022	0.002	4.6e-36	1.5e-04	154.9
Adiposity Markers	BMI	rs1701704	12	56412487	T	G	0.012	0.002	7.4e-14	5.9e-05	54.4
Adiposity Markers	BMI	rs1904558	12	68048608	A	T	0.008	0.002	2.8e-08	3.3e-05	30.6
Adiposity Markers	BMI	rs11065079	12	120752247	T	C	-0.031	0.005	1.2e-08	3.6e-05	32.6
Adiposity Markers	BMI	rs4759075	12	54667285	T	C	0.011	0.001	2.7e-15	6.4e-05	65.1
Adiposity Markers	BMI	rs6539064	12	103706754	C	G	0.020	0.002	1.3e-31	1.4e-04	132.9
Adiposity Markers	BMI	rs10774708	12	109893156	A	G	-0.008	0.001	3.3e-09	3.5e-05	36.0
Adiposity Markers	BMI	rs10842240	12	24060075	C	G	0.018	0.002	2.1e-16	7.2e-05	67.7
Adiposity Markers	BMI	rs11109097	12	97928437	T	C	-0.010	0.002	2.3e-09	3.9e-05	36.0
Adiposity Markers	BMI	rs10773049	12	124506631	T	C	-0.012	0.002	9.2e-16	6.3e-05	64.0
Adiposity Markers	BMI	rs12369179	12	122963550	T	C	-0.034	0.003	2.0e-36	1.7e-04	157.6
Adiposity Markers	BMI	rs7132908	12	50263148	A	G	0.030	0.002	2.4e-82	4.0e-04	351.6
Adiposity Markers	BMI	rs7968390	12	118414697	A	G	0.009	0.002	5.0e-09	3.5e-05	33.1
Adiposity Markers	BMI	rs11105839	12	91237920	A	T	-0.011	0.002	1.8e-12	4.9e-05	49.9
Adiposity Markers	BMI	rs11065987	12	112072424	A	G	0.014	0.001	1.3e-21	9.2e-05	94.4
Adiposity Markers	BMI	rs10772055	12	33379440	C	G	-0.018	0.002	2.8e-18	7.8e-05	79.2
Adiposity Markers	BMI	rs650198	12	69674595	T	C	-0.011	0.002	9.4e-11	4.4e-05	41.1
Adiposity Markers	BMI	rs12372279	12	116931167	A	G	0.009	0.002	4.9e-10	4.1e-05	38.4
Adiposity Markers	BMI	rs7958189	12	17233108	T	G	0.011	0.002	4.6e-13	5.9e-05	54.8
Adiposity Markers	BMI	rs7135240	12	103535020	A	G	0.008	0.002	1.9e-08	3.4e-05	31.4
Adiposity Markers	BMI	rs2600930	12	42920717	A	G	0.010	0.002	9.5e-11	4.4e-05	41.4
Adiposity Markers	BMI	rs2036312	12	117623380	T	C	-0.011	0.002	3.3e-13	5.7e-05	52.8
Adiposity Markers	BMI	rs1326099	13	59888054	T	G	-0.010	0.002	1.7e-10	4.3e-05	39.8
Adiposity Markers	BMI	rs7325099	13	28104496	A	C	-0.010	0.002	8.4e-09	3.4e-05	31.9
Adiposity Markers	BMI	rs9538141	13	59178258	A	G	0.017	0.002	1.3e-28	1.3e-04	122.5
Adiposity Markers	BMI	rs9546831	13	85304309	A	T	-0.008	0.002	1.8e-08	3.4e-05	31.4
Adiposity Markers	BMI	rs7333719	13	53628742	A	G	-0.012	0.002	9.7e-10	4.0e-05	37.8
Adiposity Markers	BMI	rs2479958	13	111984244	A	G	0.013	0.002	1.9e-16	7.0e-05	65.0
Adiposity Markers	BMI	rs9514131	13	104090848	T	G	-0.014	0.002	1.3e-09	4.0e-05	37.1
Adiposity Markers	BMI	rs9603697	13	40783323	T	C	0.014	0.002	5.8e-19	8.3e-05	77.7
Adiposity Markers	BMI	rs1045411	13	31033232	T	C	-0.013	0.002	1.4e-14	5.8e-05	58.5
Adiposity Markers	BMI	rs1417860	13	57044574	C	G	0.010	0.002	2.4e-10	4.3e-05	40.1
Adiposity Markers	BMI	rs9317220	13	62686690	T	C	0.009	0.001	7.9e-11	4.3e-05	43.2
Adiposity Markers	BMI	rs7996551	13	89193033	A	T	0.014	0.002	3.0e-09	3.6e-05	34.0
Adiposity Markers	BMI	rs2590544	13	94124153	T	C	-0.010	0.002	5.0e-09	3.5e-05	32.7
Adiposity Markers	BMI	rs17053630	13	36776501	T	C	0.023	0.004	4.3e-10	4.2e-05	39.7
Adiposity Markers	BMI	rs9634445	13	98123032	A	G	0.010	0.002	2.3e-09	3.9e-05	36.0
Adiposity Markers	BMI	rs1458260	13	54508367	A	G	0.015	0.002	2.2e-12	5.3e-05	49.7
Adiposity Markers	BMI	rs7334078	13	99120484	T	C	0.012	0.002	1.0e-13	5.7e-05	53.2
Adiposity Markers	BMI	rs1933437	13	28624294	A	G	-0.014	0.002	6.4e-20	8.8e-05	82.2
Adiposity Markers	BMI	rs9540411	13	65985590	A	G	0.009	0.002	6.3e-10	4.1e-05	38.4
Adiposity Markers	BMI	rs1444899	13	35664764	C	G	-0.009	0.002	1.0e-08	3.4e-05	32.1
Adiposity Markers	BMI	rs12429545	13	54102206	A	G	0.029	0.002	2.0e-43	1.9e-04	194.7
Adiposity Markers	BMI	rs9571687	13	67472713	A	C	-0.012	0.002	8.0e-15	6.3e-05	59.1
Adiposity Markers	BMI	rs9544915	13	36230485	T	C	0.014	0.002	1.8e-11	4.9e-05	45.7
Adiposity Markers	BMI	rs9559022	13	108013654	A	G	-0.011	0.002	2.9e-08	3.2e-05	29.7
Adiposity Markers	BMI	rs629443	13	76386075	T	G	0.010	0.002	7.8e-09	3.2e-05	32.6
Adiposity Markers	BMI	rs9595908	13	33184288	T	C	0.014	0.002	4.3e-20	8.3e-05	84.6
Adiposity Markers	BMI	rs7994913	13	78465097	A	C	-0.011	0.002	1.5e-12	5.3e-05	49.9
Adiposity Markers	BMI	rs7323	13	28009031	C	G	-0.017	0.002	4.1e-24	1.0e-04	97.7
Adiposity Markers	BMI	rs9560114	13	112187882	A	T	0.014	0.002	4.0e-16	6.8e-05	63.1
Adiposity Markers	BMI	rs543683227	13	58761425	G	I	0.029	0.005	5.2e-10	1.8e-04	38.3
Adiposity Markers	BMI	rs9527455	13	56465597	A	C	-0.013	0.002	4.4e-13	5.5e-05	51.4
Adiposity Markers	BMI	rs1927790	13	96922191	T	C	-0.014	0.001	1.2e-23	1.0e-04	104.3
Adiposity Markers	BMI	rs9531756	13	85897010	T	C	0.009	0.002	2.6e-08	3.2e-05	30.2
Adiposity Markers	BMI	rs9602892	13	86535600	T	C	-0.012	0.002	2.7e-16	7.2e-05	67.2
Adiposity Markers	BMI	rs9540493	13	66205704	A	G	0.014	0.002	5.4e-20	8.2e-05	83.4
Adiposity Markers	BMI	rs9553993	13	27906727	A	G	-0.013	0.002	2.3e-15	6.6e-05	61.2
Adiposity Markers	BMI	rs1441264	13	79580919	A	G	0.016	0.002	3.5e-27	1.2e-04	119.5

Phenotype	Trait	SNP	Chr	Position (GRCh37/hg19)	EA	NEA	BETA	SE	P-value	R2	F
Adiposity Markers	BMI	rs4517716	14	83013876	C	G	-0.011	0.002	3.0e-10	3.9e-05	39.4
Adiposity Markers	BMI	rs2295124	14	23755598	A	G	-0.010	0.002	4.3e-10	4.1e-05	37.4
Adiposity Markers	BMI	rs4900714	14	47302219	T	G	0.015	0.002	2.4e-22	1.0e-04	93.4
Adiposity Markers	BMI	rs4982256	14	35816310	A	G	-0.015	0.003	2.3e-08	3.6e-05	32.4
Adiposity Markers	BMI	rs12147845	14	101144596	T	C	0.015	0.002	8.3e-11	4.4e-05	41.2
Adiposity Markers	BMI	rs226000	14	30488699	T	C	0.012	0.002	7.6e-10	3.6e-05	36.6
Adiposity Markers	BMI	rs2412107	14	65426216	T	G	0.011	0.002	2.8e-09	3.6e-05	34.0
Adiposity Markers	BMI	rs7158822	14	103887868	T	C	-0.013	0.002	2.0e-17	7.4e-05	69.1
Adiposity Markers	BMI	rs10144318	14	53189731	A	C	0.008	0.001	3.5e-08	3.1e-05	31.8
Adiposity Markers	BMI	rs217671	14	62360464	A	G	-0.012	0.002	8.0e-14	5.7e-05	53.2
Adiposity Markers	BMI	rs8022717	14	60111221	A	G	-0.009	0.002	2.9e-08	3.2e-05	29.6
Adiposity Markers	BMI	rs1176865	14	41279543	A	G	0.016	0.003	3.2e-08	3.3e-05	30.6
Adiposity Markers	BMI	rs1958898	14	40886886	C	G	-0.013	0.002	3.3e-12	5.0e-05	46.8
Adiposity Markers	BMI	rs6420893	14	63360617	A	G	-0.010	0.002	1.3e-09	4.0e-05	37.5
Adiposity Markers	BMI	rs1188157	14	56454074	T	C	0.009	0.002	3.0e-09	3.8e-05	35.2
Adiposity Markers	BMI	rs1951455	14	91512339	T	C	-0.014	0.002	4.1e-18	7.7e-05	71.8
Adiposity Markers	BMI	rs12879626	14	34721134	T	G	-0.010	0.002	4.0e-11	4.7e-05	43.6
Adiposity Markers	BMI	rs2160077	14	92428410	A	G	0.008	0.001	7.1e-09	3.4e-05	34.3
Adiposity Markers	BMI	rs12878084	14	21945629	T	C	-0.013	0.002	3.7e-10	4.2e-05	39.5
Adiposity Markers	BMI	rs1057035	14	95554142	T	C	-0.010	0.002	1.1e-09	3.9e-05	36.0
Adiposity Markers	BMI	rs915367	14	101585751	A	G	0.009	0.002	1.5e-08	3.4e-05	32.1
Adiposity Markers	BMI	rs929328	14	73077854	A	G	-0.009	0.002	3.8e-09	3.8e-05	35.2
Adiposity Markers	BMI	rs17103222	14	66499845	A	G	0.010	0.002	3.1e-08	3.2e-05	29.6
Adiposity Markers	BMI	rs7161194	14	101529005	A	G	0.018	0.002	1.3e-27	1.2e-04	118.4
Adiposity Markers	BMI	rs17105272	14	77529783	T	C	0.012	0.002	4.3e-13	5.6e-05	52.6
Adiposity Markers	BMI	rs10450952	14	97707982	T	C	0.030	0.005	1.0e-08	3.5e-05	32.3
Adiposity Markers	BMI	rs12589840	14	51720038	A	G	0.010	0.002	3.6e-08	3.1e-05	29.0
Adiposity Markers	BMI	rs10132584	14	62595982	C	G	0.010	0.002	2.1e-10	4.3e-05	40.1
Adiposity Markers	BMI	rs8006285	14	42910684	A	C	-0.010	0.002	6.2e-10	3.9e-05	36.0
Adiposity Markers	BMI	rs2239647	14	33292743	A	C	0.018	0.002	1.2e-33	1.6e-04	144.0
Adiposity Markers	BMI	rs12880708	14	47218052	A	G	0.009	0.002	1.1e-09	4.0e-05	37.6
Adiposity Markers	BMI	rs8009710	14	88330588	T	G	0.014	0.002	1.0e-14	6.1e-05	57.1
Adiposity Markers	BMI	rs9989141	14	94006257	T	C	0.017	0.002	1.0e-28	1.3e-04	125.4
Adiposity Markers	BMI	rs17686657	14	97408661	T	C	0.025	0.004	2.2e-08	3.6e-05	31.5
Adiposity Markers	BMI	rs10132280	14	25928179	A	C	-0.022	0.002	2.2e-42	1.8e-04	185.6
Adiposity Markers	BMI	rs17668186	14	82891741	T	C	-0.013	0.002	4.8e-08	3.2e-05	29.5
Adiposity Markers	BMI	rs12885454	14	29736838	A	C	-0.018	0.002	2.1e-33	1.4e-04	147.2
Adiposity Markers	BMI	rs7144011	14	79940383	T	G	0.028	0.002	2.9e-55	2.3e-04	236.8
Adiposity Markers	BMI	rs3902951	14	69789755	T	G	-0.012	0.002	2.1e-12	4.9e-05	49.0
Adiposity Markers	BMI	rs6574100	14	73323682	A	T	0.011	0.002	1.2e-13	5.9e-05	54.8
Adiposity Markers	BMI	rs3803286	14	103246470	A	G	0.016	0.002	1.5e-24	1.1e-04	101.3
Adiposity Markers	BMI	rs2221298	14	79510587	T	C	0.013	0.002	1.2e-18	8.6e-05	79.8
Adiposity Markers	BMI	rs4082793	14	99700080	T	C	-0.012	0.002	3.0e-16	7.1e-05	66.2
Adiposity Markers	BMI	rs12595158	15	62316035	T	C	-0.038	0.005	3.2e-16	6.7e-05	67.2
Adiposity Markers	BMI	rs340025	15	60908307	T	C	-0.013	0.001	7.3e-19	8.0e-05	82.3
Adiposity Markers	BMI	rs2243790	15	53092620	A	G	0.015	0.002	4.2e-13	5.9e-05	54.8
Adiposity Markers	BMI	rs8036796	15	38118873	T	C	0.009	0.002	4.0e-10	4.1e-05	38.4
Adiposity Markers	BMI	rs2871866	15	99221888	T	C	0.011	0.002	2.6e-09	4.0e-05	37.3
Adiposity Markers	BMI	rs17236194	15	59002755	T	C	-0.016	0.002	1.2e-14	5.7e-05	58.8
Adiposity Markers	BMI	rs11856579	15	78012688	A	G	-0.013	0.002	1.5e-15	6.7e-05	62.1
Adiposity Markers	BMI	rs2577947	15	42037570	T	C	-0.016	0.002	2.1e-16	7.5e-05	70.0
Adiposity Markers	BMI	rs11636611	15	36391965	T	C	0.011	0.002	4.4e-14	6.0e-05	55.8
Adiposity Markers	BMI	rs8027745	15	73631288	A	G	-0.020	0.003	2.4e-12	5.4e-05	49.5
Adiposity Markers	BMI	rs11635675	15	63793238	T	G	0.011	0.002	5.3e-13	5.3e-05	49.9
Adiposity Markers	BMI	rs12437900	15	78134138	A	C	0.009	0.002	2.7e-09	3.7e-05	34.5
Adiposity Markers	BMI	rs12913596	15	47663870	A	G	-0.020	0.002	1.7e-15	6.6e-05	62.1
Adiposity Markers	BMI	rs7180542	15	66040030	T	C	-0.012	0.002	3.4e-10	4.4e-05	41.2
Adiposity Markers	BMI	rs10518803	15	55475264	T	C	0.030	0.005	1.3e-09	4.1e-05	37.5
Adiposity Markers	BMI	rs7162964	15	59949433	T	C	-0.009	0.002	2.6e-08	3.3e-05	30.9
Adiposity Markers	BMI	rs2899663	15	61194234	A	G	-0.009	0.001	1.2e-09	3.7e-05	37.7
Adiposity Markers	BMI	rs11853395	15	44234776	A	C	0.021	0.004	5.9e-09	3.6e-05	33.7
Adiposity Markers	BMI	rs7181039	15	44179112	A	G	0.018	0.003	1.2e-11	4.9e-05	45.8

Phenotype	Trait	SNP	Chr	Position (GRCh37/hg19)	EA	NEA	BETA	SE	P-value	R2	F
Adiposity Markers	BMI	rs12594043	15	27034988	C	G	0.010	0.002	3.1e-11	4.7e-05	43.6
Adiposity Markers	BMI	rs8028925	15	83634125	A	G	-0.030	0.005	4.4e-09	3.7e-05	34.2
Adiposity Markers	BMI	rs8036171	15	95266810	A	C	0.013	0.002	1.8e-18	7.6e-05	77.4
Adiposity Markers	BMI	rs266274	15	67287430	T	C	-0.010	0.002	3.6e-10	4.1e-05	38.3
Adiposity Markers	BMI	rs4774921	15	57584896	A	G	0.013	0.002	9.5e-10	3.8e-05	38.9
Adiposity Markers	BMI	rs12899905	15	47142090	T	C	-0.010	0.002	8.0e-10	3.7e-05	38.3
Adiposity Markers	BMI	rs11634726	15	79465894	A	G	-0.012	0.002	3.4e-14	6.4e-05	59.8
Adiposity Markers	BMI	rs7496626	15	35861857	T	C	0.011	0.002	1.1e-08	3.0e-05	31.1
Adiposity Markers	BMI	rs6496247	15	98259953	A	T	0.008	0.002	2.1e-08	3.4e-05	31.4
Adiposity Markers	BMI	rs17486278	15	78867482	A	C	0.009	0.002	1.2e-08	3.2e-05	32.9
Adiposity Markers	BMI	rs150353	15	89928189	T	G	-0.010	0.002	5.1e-11	4.6e-05	42.7
Adiposity Markers	BMI	rs16946314	15	92572682	A	G	-0.014	0.002	5.5e-14	5.7e-05	53.5
Adiposity Markers	BMI	rs5742914	15	74286929	T	C	0.015	0.002	1.3e-10	4.7e-05	43.1
Adiposity Markers	BMI	rs7164727	15	73093991	T	C	0.017	0.002	3.9e-29	1.2e-04	126.9
Adiposity Markers	BMI	rs17200912	15	66730307	T	C	-0.013	0.002	1.1e-13	5.9e-05	55.4
Adiposity Markers	BMI	rs12904649	15	46203229	T	C	-0.010	0.002	1.2e-08	3.3e-05	31.2
Adiposity Markers	BMI	rs2063732	15	81985577	A	G	0.009	0.002	1.4e-08	3.3e-05	30.9
Adiposity Markers	BMI	rs7170980	15	51956056	A	G	0.013	0.001	2.4e-20	8.7e-05	88.9
Adiposity Markers	BMI	rs12908404	15	29848687	A	G	-0.014	0.002	8.8e-09	3.5e-05	32.1
Adiposity Markers	BMI	rs13329567	15	68104367	T	C	-0.029	0.002	7.0e-69	2.9e-04	301.1
Adiposity Markers	BMI	rs2715423	15	99511873	A	G	-0.011	0.002	2.3e-11	4.6e-05	42.6
Adiposity Markers	BMI	rs12439798	15	46584787	T	G	0.014	0.002	3.2e-19	8.7e-05	81.0
Adiposity Markers	BMI	rs12914623	15	80993570	C	G	-0.015	0.002	1.1e-18	8.0e-05	74.8
Adiposity Markers	BMI	rs7172627	15	31877690	A	G	-0.010	0.002	3.3e-12	5.1e-05	48.1
Adiposity Markers	BMI	rs1877139	15	53457526	A	G	-0.028	0.003	3.0e-20	9.0e-05	83.3
Adiposity Markers	BMI	rs9927139	16	77281628	C	G	-0.014	0.002	4.5e-10	4.0e-05	37.6
Adiposity Markers	BMI	rs4843708	16	87857939	T	C	0.009	0.002	6.9e-10	4.0e-05	37.6
Adiposity Markers	BMI	rs2908871	16	49055361	C	G	-0.013	0.002	4.7e-18	8.2e-05	76.3
Adiposity Markers	BMI	rs2307022	16	68381978	A	G	0.013	0.002	7.1e-19	7.8e-05	79.8
Adiposity Markers	BMI	rs205149	16	25645124	T	C	-0.009	0.002	5.1e-09	3.7e-05	34.4
Adiposity Markers	BMI	rs756717	16	72996162	A	G	-0.014	0.002	3.4e-21	9.0e-05	92.2
Adiposity Markers	BMI	rs3865188	16	82650717	A	T	0.008	0.002	4.6e-08	3.2e-05	29.2
Adiposity Markers	BMI	rs3814883	16	29994922	T	C	0.022	0.002	4.9e-50	2.4e-04	221.0
Adiposity Markers	BMI	rs889398	16	69556715	T	C	-0.018	0.001	2.0e-37	1.7e-04	170.9
Adiposity Markers	BMI	rs7200919	16	67316600	A	G	0.009	0.001	1.3e-09	3.8e-05	38.6
Adiposity Markers	BMI	rs4888167	16	81736324	T	C	0.012	0.002	1.4e-13	6.0e-05	52.3
Adiposity Markers	BMI	rs12447501	16	49727981	T	C	-0.010	0.002	1.3e-08	3.3e-05	30.9
Adiposity Markers	BMI	rs2917705	16	64339094	A	G	-0.014	0.002	2.1e-10	4.4e-05	41.1
Adiposity Markers	BMI	rs17667675	16	72201200	T	C	-0.016	0.002	4.0e-13	5.5e-05	51.5
Adiposity Markers	BMI	rs9302652	16	53865975	T	C	-0.025	0.002	1.7e-54	2.4e-04	244.1
Adiposity Markers	BMI	rs12447655	16	20225352	A	G	0.018	0.003	9.5e-10	4.0e-05	37.2
Adiposity Markers	BMI	rs12922346	16	82438337	C	G	0.014	0.002	5.0e-16	6.8e-05	63.1
Adiposity Markers	BMI	rs2516739	16	2097158	A	G	-0.015	0.002	2.4e-18	7.3e-05	72.2
Adiposity Markers	BMI	rs7206608	16	82872628	C	G	-0.014	0.002	5.0e-19	8.3e-05	77.7
Adiposity Markers	BMI	rs9652638	16	6498714	T	C	-0.009	0.002	1.6e-09	3.9e-05	36.8
Adiposity Markers	BMI	rs9931967	16	20375351	T	G	0.015	0.002	8.1e-25	1.1e-04	104.0
Adiposity Markers	BMI	rs11639988	16	19944363	A	G	0.031	0.002	2.9e-53	2.4e-04	243.4
Adiposity Markers	BMI	rs455527	16	89644001	T	C	0.021	0.003	1.1e-11	4.7e-05	47.2
Adiposity Markers	BMI	rs194809	16	23804956	A	G	0.014	0.002	1.2e-12	5.5e-05	51.2
Adiposity Markers	BMI	rs879620	16	4015729	T	C	0.024	0.002	2.3e-59	2.8e-04	264.6
Adiposity Markers	BMI	rs977540	16	9724750	A	G	0.013	0.002	4.3e-14	5.5e-05	56.7
Adiposity Markers	BMI	rs2342892	16	24540806	T	G	0.010	0.002	2.1e-11	4.8e-05	44.4
Adiposity Markers	BMI	rs3888190	16	28889486	A	C	0.028	0.001	3.5e-86	3.9e-04	400.0
Adiposity Markers	BMI	rs214249	16	348687	T	G	0.013	0.002	3.7e-17	7.7e-05	71.7
Adiposity Markers	BMI	rs37060	16	58566304	A	G	0.010	0.002	2.4e-09	3.4e-05	34.6
Adiposity Markers	BMI	rs7186893	16	24806420	T	G	-0.015	0.002	3.7e-19	8.2e-05	76.8
Adiposity Markers	BMI	rs8054140	16	3572694	T	G	0.017	0.002	7.4e-24	1.1e-04	100.0
Adiposity Markers	BMI	rs4786518	16	1799407	A	G	-0.014	0.002	3.8e-13	6.1e-05	53.5
Adiposity Markers	BMI	rs7206790	16	53797908	C	G	-0.063	0.002	1.0e-200	1.7e-03	1752.8
Adiposity Markers	BMI	rs2906808	16	82330933	A	G	-0.014	0.002	1.0e-08	3.5e-05	32.6
Adiposity Markers	BMI	rs2058527	16	6704749	T	G	-0.012	0.002	3.8e-12	5.0e-05	46.6
Adiposity Markers	BMI	rs7189122	16	56471410	T	C	-0.016	0.002	5.7e-16	7.3e-05	68.3

Phenotype	Trait	SNP	Chr	Position (GRCh37/hg19)	EA	NEA	BETA	SE	P-value	R2	F
Adiposity Markers	BMI	rs929866	16	72038363	T	C	0.012	0.002	1.1e-15	6.4e-05	65.1
Adiposity Markers	BMI	rs2107118	16	24310282	A	G	0.011	0.002	5.3e-13	5.5e-05	49.9
Adiposity Markers	BMI	rs6497676	16	10180223	A	G	-0.009	0.002	1.5e-08	3.4e-05	32.1
Adiposity Markers	BMI	rs40425	16	9403613	A	G	-0.010	0.002	3.4e-10	4.0e-05	37.4
Adiposity Markers	BMI	rs4985155	16	15129459	A	G	0.011	0.002	2.1e-13	5.3e-05	53.8
Adiposity Markers	BMI	rs12443767	16	53638340	T	C	0.032	0.004	1.1e-13	5.6e-05	56.1
Adiposity Markers	BMI	rs2962449	16	62838736	T	C	0.012	0.002	3.2e-15	6.5e-05	60.8
Adiposity Markers	BMI	rs3112581	16	52571533	A	G	-0.011	0.002	5.7e-13	5.5e-05	50.9
Adiposity Markers	BMI	rs921471	16	8289396	A	G	0.009	0.002	4.9e-09	3.6e-05	33.6
Adiposity Markers	BMI	rs12446456	16	4922201	T	C	0.013	0.002	1.2e-17	7.8e-05	72.8
Adiposity Markers	BMI	rs437115	16	4156423	T	C	0.008	0.002	2.5e-08	3.4e-05	31.4
Adiposity Markers	BMI	rs4888536	16	76761728	A	C	-0.013	0.002	1.9e-11	5.0e-05	46.8
Adiposity Markers	BMI	rs929840	16	70045908	A	C	0.014	0.002	6.9e-21	9.5e-05	88.4
Adiposity Markers	BMI	rs1549293	16	31141993	T	C	-0.019	0.002	6.6e-37	1.6e-04	160.4
Adiposity Markers	BMI	rs1382482	16	7103790	T	G	0.009	0.002	8.8e-09	3.5e-05	32.9
Adiposity Markers	BMI	rs11076032	16	54383284	T	G	-0.015	0.003	1.1e-08	3.6e-05	33.7
Adiposity Markers	BMI	rs1094924	16	65950125	C	G	-0.009	0.002	1.3e-09	4.0e-05	37.6
Adiposity Markers	BMI	rs8081869	17	27360140	C	G	-0.013	0.002	1.1e-08	3.4e-05	31.5
Adiposity Markers	BMI	rs7223850	17	35064820	A	G	-0.012	0.002	8.1e-14	5.7e-05	53.2
Adiposity Markers	BMI	rs4295	17	61556298	C	G	0.009	0.002	1.8e-08	3.3e-05	30.9
Adiposity Markers	BMI	rs2033717	17	75719833	T	G	-0.009	0.002	7.6e-09	3.6e-05	33.6
Adiposity Markers	BMI	rs1000940	17	5283252	A	G	-0.014	0.002	2.1e-19	7.8e-05	79.9
Adiposity Markers	BMI	rs4076427	17	79087537	C	G	-0.013	0.002	2.4e-15	6.6e-05	61.0
Adiposity Markers	BMI	rs12602912	17	65870073	T	C	0.015	0.002	1.2e-17	7.0e-05	70.4
Adiposity Markers	BMI	rs9901595	17	42305699	A	G	0.012	0.002	1.1e-13	5.7e-05	53.5
Adiposity Markers	BMI	rs1285245	17	77796889	C	G	-0.011	0.002	8.9e-12	4.8e-05	44.7
Adiposity Markers	BMI	rs4986044	17	21261560	T	C	-0.017	0.001	7.1e-33	1.4e-04	147.4
Adiposity Markers	BMI	rs3826408	17	7101292	T	C	0.010	0.001	4.1e-12	4.8e-05	49.0
Adiposity Markers	BMI	rs242556	17	44002250	A	T	-0.014	0.002	2.9e-13	5.6e-05	51.2
Adiposity Markers	BMI	rs757608	17	59497277	A	G	-0.009	0.002	1.3e-08	3.2e-05	32.9
Adiposity Markers	BMI	rs7209235	17	73759552	A	G	-0.012	0.002	6.6e-14	5.7e-05	53.2
Adiposity Markers	BMI	rs12938991	17	32262916	A	G	0.010	0.002	4.6e-09	3.6e-05	33.2
Adiposity Markers	BMI	rs8080252	17	79027296	A	G	0.013	0.002	1.1e-08	3.5e-05	33.3
Adiposity Markers	BMI	rs12150665	17	34914787	T	C	0.018	0.002	7.4e-35	1.4e-04	142.4
Adiposity Markers	BMI	rs16958933	17	9801370	A	G	-0.011	0.002	9.1e-10	4.2e-05	39.6
Adiposity Markers	BMI	rs16955959	17	6493468	T	C	-0.025	0.004	1.8e-08	3.4e-05	31.1
Adiposity Markers	BMI	rs377623	17	4918458	A	C	0.012	0.002	1.9e-13	5.3e-05	53.5
Adiposity Markers	BMI	rs16954324	17	46324584	T	C	0.023	0.002	2.9e-21	8.8e-05	90.2
Adiposity Markers	BMI	rs8070454	17	38160754	T	C	-0.010	0.002	1.5e-10	4.0e-05	41.0
Adiposity Markers	BMI	rs2953016	17	29494693	C	G	0.011	0.002	1.7e-10	4.2e-05	39.4
Adiposity Markers	BMI	rs312750	17	68343539	A	G	0.011	0.001	2.1e-14	6.0e-05	61.7
Adiposity Markers	BMI	rs8075273	17	61728881	A	C	-0.012	0.002	7.0e-14	5.4e-05	55.3
Adiposity Markers	BMI	rs2288276	17	46670422	C	G	0.031	0.005	4.9e-10	1.8e-04	38.9
Adiposity Markers	BMI	rs11654648	17	45913653	T	C	-0.019	0.003	1.4e-10	4.0e-05	41.0
Adiposity Markers	BMI	rs12939549	17	78611724	A	G	0.017	0.001	1.1e-32	1.4e-04	145.7
Adiposity Markers	BMI	rs4791806	17	7729184	A	G	0.012	0.002	9.2e-10	4.0e-05	36.6
Adiposity Markers	BMI	rs7342828	17	55700956	T	G	0.010	0.002	1.7e-09	3.9e-05	36.0
Adiposity Markers	BMI	rs1075901	17	15943910	T	C	-0.011	0.001	5.0e-14	5.7e-05	58.4
Adiposity Markers	BMI	rs11649804	17	17696755	A	C	-0.009	0.002	6.3e-09	3.5e-05	33.1
Adiposity Markers	BMI	rs12941944	17	41653440	T	G	0.013	0.002	1.7e-09	4.0e-05	37.7
Adiposity Markers	BMI	rs11652097	17	45316717	T	C	0.009	0.002	4.1e-09	3.4e-05	34.4
Adiposity Markers	BMI	rs3923783	17	1843189	A	C	-0.019	0.002	9.0e-23	1.1e-04	100.0
Adiposity Markers	BMI	rs4239020	17	80176641	T	C	0.011	0.002	3.1e-13	5.4e-05	53.8
Adiposity Markers	BMI	rs12453412	17	53478118	A	G	-0.009	0.002	7.9e-09	3.6e-05	33.6
Adiposity Markers	BMI	rs8080112	17	9968168	T	C	0.008	0.002	2.7e-08	3.2e-05	29.9
Adiposity Markers	BMI	rs11077666	17	71135882	A	C	0.009	0.002	9.2e-10	4.0e-05	36.8
Adiposity Markers	BMI	rs9910424	17	55799993	T	C	-0.010	0.001	1.8e-11	4.5e-05	46.0
Adiposity Markers	BMI	rs7219230	17	39286180	T	C	0.011	0.002	3.2e-12	5.1e-05	47.3
Adiposity Markers	BMI	rs7217226	17	2136065	T	G	-0.012	0.002	9.3e-16	6.4e-05	65.1
Adiposity Markers	BMI	rs11649864	17	56093061	A	G	0.015	0.003	7.3e-09	3.8e-05	34.2
Adiposity Markers	BMI	rs1552009	17	71751656	T	C	0.010	0.002	2.6e-11	4.8e-05	44.4
Adiposity Markers	BMI	rs7218676	17	26316949	C	G	0.012	0.002	5.4e-11	4.5e-05	41.5

Phenotype	Trait	SNP	Chr	Position (GRCh37/hg19)	EA	NEA	BETA	SE	P-value	R2	F
Adiposity Markers	BMI	rs11079849	17	47090785	T	C	-0.018	0.002	1.5e-30	1.4e-04	130.8
Adiposity Markers	BMI	rs1038088	17	28074563	T	G	-0.011	0.001	4.4e-14	5.7e-05	58.4
Adiposity Markers	BMI	rs11079691	17	65362552	A	T	-0.010	0.002	3.7e-08	3.5e-05	31.9
Adiposity Markers	BMI	rs4790285	17	1651697	T	C	0.011	0.002	1.3e-09	4.1e-05	38.7
Adiposity Markers	BMI	rs8081039	17	75995829	T	C	0.021	0.003	1.0e-10	4.5e-05	41.3
Adiposity Markers	BMI	rs8068804	17	3985864	A	G	0.009	0.002	6.0e-09	3.4e-05	34.4
Adiposity Markers	BMI	rs3930349	17	31475545	A	C	-0.012	0.002	1.9e-12	5.1e-05	47.5
Adiposity Markers	BMI	rs156400	18	23178900	A	C	-0.008	0.002	2.5e-08	3.3e-05	30.6
Adiposity Markers	BMI	rs12604770	18	36534402	C	G	-0.016	0.002	9.3e-11	4.5e-05	41.7
Adiposity Markers	BMI	rs954018	18	42598463	A	G	-0.012	0.002	1.1e-14	5.7e-05	58.1
Adiposity Markers	BMI	rs7243357	18	56883319	T	G	0.020	0.002	1.1e-27	1.1e-04	115.3
Adiposity Markers	BMI	rs8089514	18	69224478	A	T	0.014	0.002	1.5e-17	7.6e-05	71.2
Adiposity Markers	BMI	rs9964756	18	50441817	T	G	-0.015	0.002	1.2e-10	4.4e-05	41.2
Adiposity Markers	BMI	rs2012927	18	63297672	A	G	0.014	0.002	9.3e-20	8.3e-05	84.6
Adiposity Markers	BMI	rs2027667	18	938932	T	C	-0.009	0.002	5.8e-09	3.6e-05	33.6
Adiposity Markers	BMI	rs1461527	18	51562666	C	G	0.010	0.002	6.6e-09	3.5e-05	32.6
Adiposity Markers	BMI	rs642666	18	41021213	A	G	0.009	0.002	7.8e-09	3.5e-05	32.9
Adiposity Markers	BMI	rs8087237	18	12834359	A	C	0.009	0.001	2.3e-10	4.0e-05	41.3
Adiposity Markers	BMI	rs414676	18	30691787	T	C	-0.018	0.003	6.3e-10	4.0e-05	37.2
Adiposity Markers	BMI	rs12970626	18	76479152	A	G	0.009	0.002	1.0e-08	3.4e-05	32.1
Adiposity Markers	BMI	rs11874286	18	36164249	A	G	-0.012	0.002	1.3e-13	5.5e-05	56.2
Adiposity Markers	BMI	rs4940569	18	60765014	A	G	0.010	0.002	7.7e-12	5.0e-05	46.2
Adiposity Markers	BMI	rs6567160	18	57829135	T	C	-0.051	0.002	1.0e-200	8.9e-04	914.2
Adiposity Markers	BMI	rs610634	18	58156977	T	C	0.013	0.002	8.3e-12	5.2e-05	48.3
Adiposity Markers	BMI	rs2229616	18	58039276	T	C	-0.099	0.005	2.2e-80	3.7e-04	362.5
Adiposity Markers	BMI	rs891387	18	21103909	T	C	0.021	0.002	6.1e-44	2.0e-04	190.4
Adiposity Markers	BMI	rs948486	18	31267711	T	C	-0.012	0.002	1.6e-15	6.7e-05	62.9
Adiposity Markers	BMI	rs7239114	18	45921214	A	G	0.011	0.002	5.5e-13	5.1e-05	51.8
Adiposity Markers	BMI	rs11150911	18	73498528	A	C	0.011	0.002	8.8e-13	5.0e-05	50.8
Adiposity Markers	BMI	rs8097672	18	1839601	A	T	-0.022	0.002	1.6e-25	1.2e-04	110.8
Adiposity Markers	BMI	rs346271	18	39972779	A	C	-0.013	0.002	2.6e-11	4.9e-05	46.1
Adiposity Markers	BMI	rs17283867	18	73202388	A	C	0.009	0.002	2.1e-08	3.3e-05	30.9
Adiposity Markers	BMI	rs497785	18	76747412	A	G	-0.024	0.003	1.3e-14	6.3e-05	58.9
Adiposity Markers	BMI	rs8092503	18	52479487	A	G	-0.016	0.002	5.3e-21	8.6e-05	87.5
Adiposity Markers	BMI	rs10438964	18	42950629	T	C	-0.013	0.002	6.4e-14	5.9e-05	54.9
Adiposity Markers	BMI	rs477805	18	7543307	A	G	-0.013	0.002	4.1e-12	5.0e-05	46.8
Adiposity Markers	BMI	rs11872122	18	56893257	A	C	0.025	0.004	3.2e-08	3.4e-05	31.3
Adiposity Markers	BMI	rs559231	18	39644247	T	G	0.012	0.002	2.3e-15	6.7e-05	62.9
Adiposity Markers	BMI	rs1426284	18	58738938	A	G	0.036	0.004	1.5e-17	7.8e-05	71.3
Adiposity Markers	BMI	rs9965170	18	44788274	A	G	-0.009	0.002	2.4e-10	4.2e-05	39.3
Adiposity Markers	BMI	rs12454712	18	60845884	T	C	-0.014	0.002	3.3e-20	8.6e-05	81.0
Adiposity Markers	BMI	rs1158805	18	40736590	A	C	-0.013	0.002	1.4e-17	7.8e-05	72.8
Adiposity Markers	BMI	rs540418	18	24260982	A	G	-0.011	0.002	1.4e-10	4.1e-05	38.7
Adiposity Markers	BMI	rs9304502	18	24498214	A	G	-0.010	0.002	1.9e-08	3.5e-05	32.7
Adiposity Markers	BMI	rs1583123	18	58206046	A	C	0.011	0.002	2.5e-11	4.6e-05	43.1
Adiposity Markers	BMI	rs16940823	18	22137319	A	C	-0.016	0.002	7.0e-16	6.8e-05	63.2
Adiposity Markers	BMI	rs2012353	19	32899293	A	G	-0.011	0.002	7.8e-09	3.1e-05	31.9
Adiposity Markers	BMI	rs7258998	19	51771284	A	G	-0.011	0.002	1.1e-12	5.0e-05	50.8
Adiposity Markers	BMI	rs79712071	19	2221892	A	C	0.047	0.008	1.5e-08	1.5e-04	31.7
Adiposity Markers	BMI	rs17724992	19	18454825	A	G	0.018	0.002	1.5e-27	1.2e-04	119.6
Adiposity Markers	BMI	rs10421621	19	30920085	C	G	0.009	0.002	7.9e-09	3.5e-05	32.3
Adiposity Markers	BMI	rs454282	19	30599783	A	G	-0.011	0.002	3.0e-09	3.8e-05	35.3
Adiposity Markers	BMI	rs17513613	19	30286822	T	C	-0.017	0.002	3.1e-28	1.2e-04	122.5
Adiposity Markers	BMI	rs2965195	19	19463032	A	G	-0.014	0.002	1.1e-13	5.6e-05	57.4
Adiposity Markers	BMI	rs10518269	19	31028666	T	C	-0.018	0.002	7.7e-19	8.6e-05	80.1
Adiposity Markers	BMI	rs3826705	19	42637232	T	C	-0.013	0.002	1.4e-08	3.6e-05	33.4
Adiposity Markers	BMI	rs29938	19	34311481	T	C	-0.015	0.002	2.0e-23	9.8e-05	100.0
Adiposity Markers	BMI	rs12609744	19	12994140	T	C	-0.011	0.002	2.7e-11	4.5e-05	41.9
Adiposity Markers	BMI	rs12979351	19	51304404	C	G	0.016	0.003	1.3e-08	3.9e-05	33.4
Adiposity Markers	BMI	rs3764625	19	49649051	T	G	0.009	0.002	3.3e-09	3.7e-05	34.4
Adiposity Markers	BMI	rs895330	19	4060707	C	G	0.017	0.002	4.2e-19	8.7e-05	81.0
Adiposity Markers	BMI	rs741923	19	4448843	A	G	-0.009	0.002	5.9e-09	3.7e-05	34.4

Phenotype	Trait	SNP	Chr	Position (GRCh37/hg19)	EA	NEA	BETA	SE	P-value	R2	F
Adiposity Markers	BMI	rs6857	19	45392254	T	C	-0.028	0.002	1.1e-42	2.1e-04	193.2
Adiposity Markers	BMI	rs1800437	19	46181392	C	G	-0.035	0.002	7.1e-81	3.8e-04	375.9
Adiposity Markers	BMI	rs3810291	19	47569003	A	G	0.026	0.002	1.6e-66	3.1e-04	305.1
Adiposity Markers	BMI	rs143430880	19	46180976	A	G	0.223	0.037	1.8e-09	1.7e-04	36.2
Adiposity Markers	BMI	rs10403445	19	13940959	C	G	0.010	0.002	9.8e-09	3.4e-05	31.5
Adiposity Markers	BMI	rs11672894	19	45801951	T	C	0.010	0.002	1.3e-09	3.6e-05	36.8
Adiposity Markers	BMI	rs2965109	19	45225345	T	C	0.008	0.002	2.8e-08	3.0e-05	30.6
Adiposity Markers	BMI	rs7258722	19	18808915	A	T	-0.021	0.002	5.5e-45	2.1e-04	199.8
Adiposity Markers	BMI	rs3786915	19	33987424	T	G	-0.010	0.002	7.8e-09	3.6e-05	33.2
Adiposity Markers	BMI	rs12981256	19	1865901	A	G	0.015	0.002	1.2e-22	1.0e-04	96.0
Adiposity Markers	BMI	rs7259012	19	18321273	A	G	-0.009	0.002	6.2e-09	3.5e-05	33.1
Adiposity Markers	BMI	rs3092209	20	45489808	A	T	-0.009	0.002	8.3e-10	4.1e-05	38.4
Adiposity Markers	BMI	rs2145272	20	6626218	A	G	0.018	0.002	1.2e-31	1.3e-04	136.1
Adiposity Markers	BMI	rs6029180	20	39178923	A	G	-0.011	0.002	1.6e-12	5.2e-05	49.0
Adiposity Markers	BMI	rs4911442	20	33355046	A	G	0.015	0.002	4.7e-12	4.5e-05	46.5
Adiposity Markers	BMI	rs6069436	20	54443569	T	C	0.010	0.002	1.5e-10	4.4e-05	41.0
Adiposity Markers	BMI	rs6050446	20	25195509	A	G	-0.034	0.004	7.9e-16	6.5e-05	65.1
Adiposity Markers	BMI	rs6055745	20	8312279	A	T	-0.011	0.002	2.5e-10	4.1e-05	38.1
Adiposity Markers	BMI	rs6010675	20	62468726	T	C	0.020	0.003	2.3e-09	3.8e-05	35.6
Adiposity Markers	BMI	rs17806379	20	51107290	T	C	-0.026	0.002	3.8e-41	2.0e-04	187.3
Adiposity Markers	BMI	rs6010784	20	61540319	T	C	0.011	0.001	1.0e-14	6.1e-05	61.7
Adiposity Markers	BMI	rs2425241	20	35018412	T	C	-0.019	0.003	1.0e-10	4.6e-05	42.5
Adiposity Markers	BMI	rs2243930	20	54147462	A	G	0.014	0.002	3.1e-15	5.8e-05	59.6
Adiposity Markers	BMI	rs2143624	20	49781718	A	G	0.010	0.002	2.3e-10	4.0e-05	41.0
Adiposity Markers	BMI	rs2425024	20	33844938	A	C	-0.010	0.002	8.6e-11	4.4e-05	41.4
Adiposity Markers	BMI	rs6130360	20	42010996	A	G	0.016	0.002	6.0e-15	6.6e-05	61.7
Adiposity Markers	BMI	rs8123881	20	15819495	A	G	-0.018	0.002	2.0e-17	6.8e-05	70.0
Adiposity Markers	BMI	rs2423668	20	12430673	T	C	0.012	0.002	2.3e-14	7.3e-05	56.2
Adiposity Markers	BMI	rs6020179	20	48604276	T	C	-0.014	0.002	3.9e-10	4.2e-05	38.8
Adiposity Markers	BMI	rs1884389	20	1410582	T	C	-0.009	0.002	1.8e-09	3.9e-05	36.0
Adiposity Markers	BMI	rs4813852	20	871830	A	C	-0.008	0.002	1.9e-08	3.1e-05	31.4
Adiposity Markers	BMI	rs742748	20	39293397	T	C	-0.010	0.002	4.0e-11	4.4e-05	44.4
Adiposity Markers	BMI	rs6031858	20	43528962	T	G	0.010	0.002	5.5e-09	3.5e-05	32.6
Adiposity Markers	BMI	rs947088	20	17171373	T	G	0.012	0.002	9.5e-13	5.3e-05	49.8
Adiposity Markers	BMI	rs2425840	20	44904838	A	C	-0.012	0.002	2.9e-15	6.8e-05	62.9
Adiposity Markers	BMI	rs4813224	20	16546846	T	C	-0.010	0.002	5.3e-09	3.6e-05	33.2
Adiposity Markers	BMI	rs2224359	20	9072462	A	C	-0.011	0.002	1.2e-08	3.6e-05	33.5
Adiposity Markers	BMI	rs615568	20	3008775	T	G	-0.011	0.002	2.1e-12	5.2e-05	49.0
Adiposity Markers	BMI	rs368380	20	14814090	T	C	-0.009	0.002	7.1e-09	3.5e-05	33.1
Adiposity Markers	BMI	rs6512302	20	62691550	C	G	0.013	0.002	5.1e-13	5.3e-05	49.8
Adiposity Markers	BMI	rs6142067	20	32556572	T	C	0.013	0.002	4.9e-18	8.0e-05	75.1
Adiposity Markers	BMI	rs1056776	20	30782543	C	G	-0.014	0.002	1.9e-12	5.5e-05	51.1
Adiposity Markers	BMI	rs6019483	20	47495656	A	T	-0.015	0.002	3.1e-13	5.9e-05	54.8
Adiposity Markers	BMI	rs6068337	20	51393458	A	T	-0.011	0.002	4.4e-09	3.7e-05	34.0
Adiposity Markers	BMI	rs2876175	20	9872828	T	C	-0.009	0.002	1.1e-09	3.9e-05	36.8
Adiposity Markers	BMI	rs1409818	20	21381121	T	C	0.018	0.002	1.9e-12	5.2e-05	49.0
Adiposity Markers	BMI	rs2525482	20	51790871	T	C	-0.015	0.003	3.6e-08	3.2e-05	30.2
Adiposity Markers	BMI	rs2064044	21	22119890	A	C	-0.011	0.002	1.8e-08	3.3e-05	30.5
Adiposity Markers	BMI	rs427943	21	46570896	A	C	-0.016	0.002	4.8e-25	1.1e-04	109.6
Adiposity Markers	BMI	rs2836961	21	40627020	A	C	-0.009	0.002	2.8e-09	3.5e-05	36.0
Adiposity Markers	BMI	rs11088452	21	40206402	T	C	0.011	0.002	3.3e-11	4.6e-05	43.1
Adiposity Markers	BMI	rs2832157	21	30415629	A	G	-0.011	0.002	2.3e-10	4.0e-05	41.1
Adiposity Markers	BMI	rs762147	21	39238610	A	G	-0.011	0.002	3.0e-10	4.1e-05	38.1
Adiposity Markers	BMI	rs4818751	21	46440385	A	C	-0.009	0.002	1.1e-09	4.0e-05	37.6
Adiposity Markers	BMI	rs13047416	21	40309436	C	G	0.017	0.002	2.2e-26	1.2e-04	108.9
Adiposity Markers	BMI	rs207464	21	25198321	T	G	-0.009	0.002	1.1e-09	3.7e-05	37.6
Adiposity Markers	BMI	rs11909493	21	44467691	A	G	-0.012	0.002	9.3e-09	3.6e-05	33.2
Adiposity Markers	BMI	rs2298330	21	38852728	A	G	-0.014	0.002	1.6e-09	3.7e-05	38.2
Adiposity Markers	BMI	rs2838006	21	42653567	T	C	-0.013	0.002	1.5e-16	7.2e-05	67.0
Adiposity Markers	BMI	rs2262241	21	32646060	T	G	0.013	0.002	4.7e-08	3.2e-05	29.5
Adiposity Markers	BMI	rs2837398	21	41427168	A	C	-0.009	0.002	7.2e-10	4.1e-05	37.6
Adiposity Markers	BMI	rs8142495	22	40581822	A	G	0.016	0.002	1.4e-26	1.1e-04	113.8

Phenotype	Trait	SNP	Chr	Position (GRCh37/hg19)	EA	NEA	BETA	SE	P-value	R2	F
Adiposity Markers	BMI	rs138289	22	32182708	A	T	0.009	0.002	7.5e-09	3.6e-05	33.6
Adiposity Markers	BMI	rs713706	22	27443484	T	C	0.009	0.002	5.4e-10	4.1e-05	38.4
Adiposity Markers	BMI	rs4646312	22	19948337	T	C	0.010	0.002	1.5e-10	4.4e-05	41.0
Adiposity Markers	BMI	rs619015	22	20142379	C	G	-0.009	0.002	4.8e-09	3.7e-05	34.5
Adiposity Markers	BMI	rs13053342	22	48871624	A	G	0.012	0.002	3.7e-15	6.3e-05	59.1
Adiposity Markers	BMI	rs4823173	22	44328730	A	G	-0.011	0.002	2.1e-08	3.4e-05	31.9
Adiposity Markers	BMI	rs132645	22	36548406	A	G	-0.012	0.002	1.0e-08	3.5e-05	33.1
Adiposity Markers	BMI	rs136817	22	40034609	T	C	0.011	0.002	4.5e-08	3.2e-05	30.2
Adiposity Markers	BMI	rs6002677	22	42701419	A	G	-0.009	0.002	1.8e-09	3.7e-05	34.5
Adiposity Markers	BMI	rs738140	22	41884954	A	G	0.012	0.002	1.5e-14	6.3e-05	59.1
Adiposity Markers	BMI	rs9616409	22	49422624	T	G	0.011	0.002	3.6e-09	3.6e-05	34.0
Adiposity Markers	BMI	rs5750508	22	38303155	T	C	-0.011	0.002	1.2e-11	5.2e-05	48.1
Adiposity Markers	BMI	rs3819663	22	33965639	A	G	-0.009	0.002	1.3e-08	3.4e-05	32.1
Adiposity Markers	BMI	rs9610561	22	37064528	A	G	0.010	0.002	4.5e-09	3.6e-05	33.2
Adiposity Markers	BMI	rs395379	22	18212923	A	G	-0.014	0.002	4.5e-12	5.2e-05	48.3
Adiposity Markers	BMI	rs4275	22	26921725	A	G	-0.009	0.002	1.0e-09	3.9e-05	36.8
Adiposity Markers	WHR	rs717795	1	2970464	C	T	0.020	0.002	6.9e-18	1.2e-04	72.6
Adiposity Markers	WHR	rs6688233	1	9335745	T	C	0.021	0.002	9.6e-24	1.6e-04	100.0
Adiposity Markers	WHR	rs2076600	1	17337391	A	G	-0.012	0.002	1.5e-09	5.9e-05	37.2
Adiposity Markers	WHR	rs2235529	1	22450487	T	C	-0.018	0.002	1.3e-13	8.5e-05	53.0
Adiposity Markers	WHR	rs2298632	1	23710475	T	C	-0.016	0.002	5.0e-19	1.2e-04	83.1
Adiposity Markers	WHR	rs807067	1	26197063	T	C	0.011	0.002	1.2e-10	6.0e-05	41.9
Adiposity Markers	WHR	rs547976	1	29503214	A	G	-0.011	0.002	3.1e-08	4.5e-05	31.1
Adiposity Markers	WHR	rs645383	1	36373823	G	C	0.019	0.003	1.2e-08	5.2e-05	32.5
Adiposity Markers	WHR	rs11205773	1	51284905	C	T	-0.019	0.003	2.3e-09	5.8e-05	36.4
Adiposity Markers	WHR	rs875868	1	65539253	C	A	-0.012	0.002	6.9e-11	6.8e-05	42.6
Adiposity Markers	WHR	rs598104	1	86266109	T	A	0.014	0.002	5.4e-14	9.2e-05	57.9
Adiposity Markers	WHR	rs236319	1	94054382	C	T	-0.011	0.002	4.5e-08	5.3e-05	30.5
Adiposity Markers	WHR	rs11164653	1	103464210	C	T	0.013	0.002	2.9e-13	8.7e-05	54.6
Adiposity Markers	WHR	rs11579145	1	110501597	A	G	-0.010	0.002	2.8e-08	4.8e-05	30.2
Adiposity Markers	WHR	rs3789615	1	114941326	T	C	-0.012	0.002	7.3e-12	7.1e-05	48.2
Adiposity Markers	WHR	rs7534091	1	118864616	G	A	-0.017	0.002	1.4e-18	1.2e-04	81.0
Adiposity Markers	WHR	rs10923724	1	119546842	T	C	0.035	0.002	1.2e-89	6.0e-04	416.6
Adiposity Markers	WHR	rs11204762	1	150999737	G	A	-0.014	0.002	3.4e-11	7.1e-05	44.4
Adiposity Markers	WHR	rs951240	1	154793763	G	A	-0.012	0.002	3.1e-09	5.7e-05	36.0
Adiposity Markers	WHR	rs905938	1	154991389	C	T	-0.024	0.002	1.3e-35	2.1e-04	147.6
Adiposity Markers	WHR	rs2274319	1	156450873	C	T	-0.012	0.002	5.3e-10	5.9e-05	37.3
Adiposity Markers	WHR	rs1249558	1	160424761	A	T	-0.013	0.002	3.1e-11	7.1e-05	44.2
Adiposity Markers	WHR	rs7530102	1	163760257	A	T	0.012	0.002	1.5e-10	6.7e-05	42.2
Adiposity Markers	WHR	rs2279469	1	164740405	C	G	0.013	0.002	7.9e-13	8.5e-05	52.2
Adiposity Markers	WHR	rs6426912	1	165321568	C	T	-0.019	0.003	6.2e-11	7.2e-05	43.4
Adiposity Markers	WHR	rs17345907	1	170174046	G	T	0.022	0.003	1.5e-10	6.1e-05	42.2
Adiposity Markers	WHR	rs10919388	1	170372503	C	A	0.033	0.002	9.7e-66	4.6e-04	307.2
Adiposity Markers	WHR	rs10919477	1	170773792	A	C	0.020	0.004	3.7e-08	4.8e-05	29.9
Adiposity Markers	WHR	rs912299	1	172187250	A	G	-0.014	0.002	4.3e-14	9.5e-05	59.6
Adiposity Markers	WHR	rs714515	1	172352990	A	G	-0.028	0.002	6.5e-59	3.8e-04	263.6
Adiposity Markers	WHR	rs6425326	1	175198598	C	T	0.011	0.002	6.5e-09	5.0e-05	34.7
Adiposity Markers	WHR	rs2504489	1	176346512	T	A	-0.011	0.002	4.3e-09	5.4e-05	34.0
Adiposity Markers	WHR	rs10913257	1	176778610	T	G	-0.011	0.002	2.4e-09	5.6e-05	35.3
Adiposity Markers	WHR	rs6658424	1	200049302	A	T	-0.014	0.002	2.9e-13	8.1e-05	51.1
Adiposity Markers	WHR	rs6427992	1	203102563	C	G	0.010	0.002	1.2e-08	5.3e-05	33.4
Adiposity Markers	WHR	rs2821231	1	203518382	C	T	-0.012	0.002	4.9e-10	6.8e-05	40.8
Adiposity Markers	WHR	rs3851294	1	205130413	G	A	0.026	0.003	9.0e-17	1.1e-04	68.7
Adiposity Markers	WHR	rs4951583	1	212416078	T	C	-0.013	0.002	2.1e-13	8.7e-05	54.6
Adiposity Markers	WHR	rs2791550	1	219655369	G	T	0.037	0.002	3.3e-93	6.1e-04	422.5
Adiposity Markers	WHR	rs11118352	1	219756559	T	G	-0.027	0.002	3.4e-33	2.3e-04	141.9
Adiposity Markers	WHR	rs715300	1	221290755	T	C	0.014	0.002	1.1e-13	9.2e-05	57.4
Adiposity Markers	WHR	rs6604731	1	224051439	T	C	-0.011	0.002	2.8e-08	4.7e-05	29.7
Adiposity Markers	WHR	rs10799424	1	227755475	A	T	-0.013	0.002	3.1e-09	5.4e-05	33.9
Adiposity Markers	WHR	rs10803236	1	236191170	G	A	0.010	0.002	2.9e-08	4.7e-05	29.4
Adiposity Markers	WHR	rs11893688	2	9695282	T	C	0.014	0.002	7.7e-13	8.9e-05	52.8
Adiposity Markers	WHR	rs6432188	2	11499032	C	T	-0.014	0.002	2.3e-10	5.6e-05	38.7

Phenotype	Trait	SNP	Chr	Position (GRCh37/hg19)	EA	NEA	BETA	SE	P-value	R2	F
Adiposity Markers	WHR	rs711869	2	13073967	A	G	-0.019	0.002	2.0e-24	1.7e-04	106.8
Adiposity Markers	WHR	rs17509001	2	24021231	C	T	0.016	0.002	5.1e-11	6.0e-05	41.5
Adiposity Markers	WHR	rs7589318	2	25378372	A	G	-0.016	0.002	1.2e-18	1.2e-04	81.0
Adiposity Markers	WHR	rs13406302	2	37874850	C	A	0.014	0.002	2.2e-12	7.3e-05	50.5
Adiposity Markers	WHR	rs11676137	2	37941348	C	G	0.011	0.002	1.2e-08	5.2e-05	32.9
Adiposity Markers	WHR	rs6752964	2	43754655	C	T	0.019	0.003	1.7e-11	6.6e-05	45.6
Adiposity Markers	WHR	rs17326656	2	48962291	T	G	0.015	0.002	5.8e-13	8.5e-05	53.1
Adiposity Markers	WHR	rs6717480	2	58210522	A	C	0.012	0.002	1.6e-11	7.3e-05	45.9
Adiposity Markers	WHR	rs13028903	2	59951465	T	C	0.010	0.002	9.3e-09	5.3e-05	33.4
Adiposity Markers	WHR	rs13406620	2	66028199	T	C	0.015	0.002	1.5e-10	6.5e-05	40.6
Adiposity Markers	WHR	rs6719672	2	66234202	G	A	0.026	0.002	1.7e-29	2.1e-04	130.8
Adiposity Markers	WHR	rs11897119	2	66772000	C	T	0.014	0.002	5.7e-16	1.0e-04	69.8
Adiposity Markers	WHR	rs2062204	2	67472545	C	A	-0.016	0.002	2.6e-14	9.0e-05	56.6
Adiposity Markers	WHR	rs7598832	2	67853013	T	C	-0.018	0.002	1.1e-24	1.5e-04	103.4
Adiposity Markers	WHR	rs749458	2	96795857	C	T	0.013	0.002	2.5e-08	4.8e-05	30.0
Adiposity Markers	WHR	rs9678859	2	100288478	G	A	-0.019	0.002	8.0e-16	1.1e-04	66.8
Adiposity Markers	WHR	rs3761706	2	111899881	A	G	0.032	0.004	1.7e-18	1.2e-04	75.3
Adiposity Markers	WHR	rs13405741	2	111913056	C	T	-0.020	0.003	1.5e-11	7.5e-05	46.7
Adiposity Markers	WHR	rs4849294	2	114619997	T	C	0.013	0.002	1.8e-13	8.8e-05	55.4
Adiposity Markers	WHR	rs332105	2	119444229	A	G	-0.014	0.002	4.9e-14	9.3e-05	57.9
Adiposity Markers	WHR	rs2509903	2	158514510	C	T	0.018	0.002	3.4e-13	7.3e-05	50.7
Adiposity Markers	WHR	rs6743277	2	158519713	C	T	-0.064	0.009	5.4e-14	9.9e-05	56.7
Adiposity Markers	WHR	rs270960	2	161714729	C	A	-0.011	0.002	9.0e-10	5.5e-05	38.0
Adiposity Markers	WHR	rs10195252	2	165513091	C	T	-0.032	0.002	8.4e-78	5.2e-04	363.2
Adiposity Markers	WHR	rs1509097	2	165737931	T	C	-0.015	0.002	7.0e-18	1.2e-04	72.2
Adiposity Markers	WHR	rs6433219	2	171421125	A	G	0.016	0.002	2.8e-15	1.0e-04	65.6
Adiposity Markers	WHR	rs12052537	2	172369108	A	C	0.014	0.002	5.0e-10	6.4e-05	39.9
Adiposity Markers	WHR	rs1569135	2	188115398	G	A	-0.020	0.002	3.0e-33	2.1e-04	145.4
Adiposity Markers	WHR	rs1921913	2	191749543	G	C	-0.018	0.003	4.8e-08	4.7e-05	29.4
Adiposity Markers	WHR	rs3732083	2	207041053	C	T	0.011	0.002	1.7e-10	6.4e-05	40.1
Adiposity Markers	WHR	rs1250259	2	216300482	A	T	-0.016	0.002	1.0e-15	1.1e-04	67.2
Adiposity Markers	WHR	rs2373078	2	218392555	T	C	0.021	0.003	1.1e-13	8.8e-05	55.0
Adiposity Markers	WHR	rs3731861	2	219191256	C	T	0.011	0.002	1.4e-09	5.5e-05	34.7
Adiposity Markers	WHR	rs2602680	2	225066513	G	T	-0.012	0.002	1.0e-10	6.6e-05	41.5
Adiposity Markers	WHR	rs1522811	2	226992454	A	C	-0.013	0.002	1.6e-09	6.0e-05	37.7
Adiposity Markers	WHR	rs12466434	2	239674329	T	C	0.010	0.002	2.4e-08	4.8e-05	30.0
Adiposity Markers	WHR	rs2270894	3	9975386	G	C	0.014	0.002	1.1e-08	5.5e-05	32.6
Adiposity Markers	WHR	rs2595002	3	11373700	A	G	-0.015	0.002	3.3e-10	6.5e-05	40.8
Adiposity Markers	WHR	rs598747	3	12112010	A	G	-0.021	0.002	6.8e-17	1.1e-04	71.9
Adiposity Markers	WHR	rs709151	3	12454999	T	C	-0.014	0.002	8.4e-14	8.8e-05	55.1
Adiposity Markers	WHR	rs17819328	3	12489342	G	T	0.025	0.002	4.8e-46	3.0e-04	211.1
Adiposity Markers	WHR	rs1286769	3	25585166	A	G	-0.010	0.002	1.6e-08	4.8e-05	33.2
Adiposity Markers	WHR	rs10212473	3	33872984	G	A	0.012	0.002	5.7e-09	5.5e-05	34.8
Adiposity Markers	WHR	rs11129657	3	35636709	C	T	0.015	0.002	2.3e-11	7.1e-05	44.6
Adiposity Markers	WHR	rs174829	3	37535577	G	A	0.014	0.002	6.3e-13	9.2e-05	54.3
Adiposity Markers	WHR	rs740838	3	43764315	A	C	-0.043	0.006	9.0e-14	9.1e-05	55.2
Adiposity Markers	WHR	rs9855938	3	46957298	A	G	0.015	0.002	1.6e-17	1.1e-04	73.2
Adiposity Markers	WHR	rs6446204	3	49034879	C	T	0.021	0.002	1.2e-24	1.5e-04	107.1
Adiposity Markers	WHR	rs7652260	3	50571585	G	C	0.015	0.002	5.8e-10	6.1e-05	37.9
Adiposity Markers	WHR	rs2276824	3	52637486	G	C	-0.022	0.002	8.9e-38	2.5e-04	170.5
Adiposity Markers	WHR	rs17052727	3	53070914	C	T	-0.021	0.003	2.1e-12	7.8e-05	48.1
Adiposity Markers	WHR	rs6768738	3	53270849	C	G	0.017	0.002	1.6e-13	8.6e-05	53.4
Adiposity Markers	WHR	rs17289049	3	57187036	G	A	0.019	0.003	2.0e-10	5.9e-05	40.5
Adiposity Markers	WHR	rs2371767	3	64718258	C	G	-0.040	0.002	1.0e-100	6.6e-04	447.7
Adiposity Markers	WHR	rs1612708	3	64888938	A	G	0.012	0.002	1.9e-11	7.3e-05	45.9
Adiposity Markers	WHR	rs7609819	3	64901626	C	T	-0.015	0.002	2.2e-12	8.1e-05	51.0
Adiposity Markers	WHR	rs7651129	3	66474954	G	A	-0.019	0.003	2.6e-08	4.9e-05	30.9
Adiposity Markers	WHR	rs17311057	3	78841789	T	C	0.015	0.002	1.7e-12	8.5e-05	52.6
Adiposity Markers	WHR	rs17775314	3	99255124	G	A	0.019	0.003	4.5e-09	5.8e-05	35.6
Adiposity Markers	WHR	rs12489030	3	99696469	G	A	0.022	0.004	6.5e-10	6.5e-05	38.4
Adiposity Markers	WHR	rs16853606	3	107270460	G	A	0.015	0.002	1.2e-10	6.9e-05	43.1
Adiposity Markers	WHR	rs12496583	3	123121286	G	A	0.011	0.002	1.6e-08	4.8e-05	33.5

Phenotype	Trait	SNP	Chr	Position (GRCh37/hg19)	EA	NEA	BETA	SE	P-value	R2	F
Adiposity Markers	WHR	rs1979527	3	127400781	A	C	0.013	0.002	2.4e-09	5.9e-05	37.1
Adiposity Markers	WHR	rs6795831	3	129341403	C	A	-0.035	0.002	4.9e-53	3.6e-04	228.9
Adiposity Markers	WHR	rs4688833	3	129667868	C	A	0.020	0.002	3.1e-16	1.1e-04	66.7
Adiposity Markers	WHR	rs10935182	3	136137422	A	G	0.011	0.002	1.4e-09	5.8e-05	36.7
Adiposity Markers	WHR	rs34905952	3	138104635	A	G	0.021	0.003	1.2e-15	1.2e-04	64.6
Adiposity Markers	WHR	rs9844972	3	150097635	C	G	0.033	0.004	2.5e-19	1.4e-04	82.0
Adiposity Markers	WHR	rs10049088	3	156797648	T	C	-0.029	0.002	1.5e-59	3.8e-04	259.6
Adiposity Markers	WHR	rs998749	3	168972802	G	A	-0.014	0.002	6.2e-16	9.9e-05	66.9
Adiposity Markers	WHR	rs9647379	3	171785168	C	G	-0.015	0.002	5.1e-16	1.0e-04	62.3
Adiposity Markers	WHR	rs4686696	3	185516520	A	G	0.015	0.002	5.7e-17	1.0e-04	71.3
Adiposity Markers	WHR	rs522833	3	187677380	G	C	0.013	0.002	6.0e-09	5.3e-05	33.3
Adiposity Markers	WHR	rs11724804	4	965779	A	G	-0.017	0.002	7.6e-21	1.4e-04	89.2
Adiposity Markers	WHR	rs3121419	4	3232257	T	C	-0.011	0.002	8.5e-09	5.4e-05	34.1
Adiposity Markers	WHR	rs4450871	4	4990298	G	A	-0.017	0.002	3.2e-18	1.3e-04	76.3
Adiposity Markers	WHR	rs11722554	4	5016883	A	G	-0.033	0.005	1.3e-11	8.2e-05	46.5
Adiposity Markers	WHR	rs917681	4	13247539	T	C	0.012	0.002	7.6e-10	5.3e-05	36.6
Adiposity Markers	WHR	rs4586926	4	15375527	C	A	-0.012	0.002	1.3e-10	6.3e-05	39.2
Adiposity Markers	WHR	rs2137236	4	26080766	C	A	0.021	0.002	5.1e-19	1.2e-04	76.6
Adiposity Markers	WHR	rs6853254	4	26352363	G	T	-0.017	0.002	4.0e-20	1.3e-04	83.9
Adiposity Markers	WHR	rs10462028	4	56298300	A	G	0.019	0.002	2.9e-23	1.6e-04	98.9
Adiposity Markers	WHR	rs2167750	4	89730074	T	C	0.027	0.002	5.5e-53	3.7e-04	231.7
Adiposity Markers	WHR	rs4693978	4	89854167	C	A	0.012	0.002	6.1e-12	7.6e-05	47.5
Adiposity Markers	WHR	rs1813006	4	103001649	T	G	-0.031	0.004	6.4e-14	9.5e-05	56.1
Adiposity Markers	WHR	rs4464576	4	106177882	A	G	-0.015	0.002	9.9e-12	7.2e-05	45.3
Adiposity Markers	WHR	rs1511022	4	120126233	T	C	0.017	0.003	6.4e-10	6.2e-05	39.2
Adiposity Markers	WHR	rs7680787	4	122624065	C	T	-0.014	0.002	1.2e-14	8.5e-05	58.8
Adiposity Markers	WHR	rs303084	4	124066948	A	G	0.019	0.002	2.3e-19	1.1e-04	79.3
Adiposity Markers	WHR	rs11726981	4	125220708	C	A	0.015	0.002	1.3e-13	8.8e-05	55.5
Adiposity Markers	WHR	rs4256191	4	145240011	A	C	-0.012	0.002	1.1e-09	5.8e-05	36.6
Adiposity Markers	WHR	rs789351	4	145868370	T	C	0.019	0.002	5.0e-27	1.8e-04	121.0
Adiposity Markers	WHR	rs1425486	4	157683685	T	C	-0.012	0.002	2.9e-11	6.3e-05	43.7
Adiposity Markers	WHR	rs2333496	4	177609609	T	C	0.011	0.002	7.1e-10	5.8e-05	40.1
Adiposity Markers	WHR	rs2459732	5	4027324	T	C	-0.013	0.002	6.3e-13	8.3e-05	52.2
Adiposity Markers	WHR	rs3792751	5	32773314	T	C	0.014	0.002	1.4e-14	8.2e-05	57.1
Adiposity Markers	WHR	rs299615	5	34042097	A	G	-0.015	0.002	1.7e-09	5.6e-05	35.0
Adiposity Markers	WHR	rs4293945	5	38736142	T	A	-0.012	0.002	1.7e-10	6.8e-05	42.2
Adiposity Markers	WHR	rs6874524	5	53343529	C	T	-0.013	0.002	8.9e-10	6.2e-05	38.9
Adiposity Markers	WHR	rs7721054	5	54890802	C	T	-0.017	0.002	1.6e-19	1.1e-04	79.1
Adiposity Markers	WHR	rs459193	5	55806751	G	A	-0.028	0.002	4.0e-47	3.1e-04	214.1
Adiposity Markers	WHR	rs11948950	5	55876283	A	G	0.028	0.003	1.5e-21	1.4e-04	92.6
Adiposity Markers	WHR	rs13186194	5	60795485	C	T	0.010	0.002	7.0e-09	4.6e-05	32.1
Adiposity Markers	WHR	rs6867518	5	64679283	C	T	0.018	0.003	1.9e-11	7.1e-05	44.4
Adiposity Markers	WHR	rs2545401	5	66348255	C	G	0.010	0.002	2.1e-08	5.2e-05	32.7
Adiposity Markers	WHR	rs4704389	5	76446103	G	A	-0.012	0.002	1.7e-12	7.6e-05	52.3
Adiposity Markers	WHR	rs188703	5	82834299	A	G	0.010	0.002	2.6e-08	5.2e-05	32.7
Adiposity Markers	WHR	rs17154889	5	102305065	A	C	-0.016	0.002	3.9e-16	1.1e-04	69.2
Adiposity Markers	WHR	rs1045241	5	118729286	T	C	-0.018	0.002	3.5e-22	1.4e-04	94.8
Adiposity Markers	WHR	rs17764730	5	127357526	T	C	-0.016	0.002	9.7e-15	9.5e-05	59.5
Adiposity Markers	WHR	rs867150	5	130743668	A	G	0.021	0.004	1.5e-08	5.3e-05	32.2
Adiposity Markers	WHR	rs11747001	5	132412299	G	A	-0.017	0.002	7.7e-18	1.1e-04	74.0
Adiposity Markers	WHR	rs6881034	5	134405482	C	T	0.014	0.002	7.9e-12	7.4e-05	46.2
Adiposity Markers	WHR	rs10055995	5	137698299	T	C	-0.011	0.002	1.9e-09	5.8e-05	36.7
Adiposity Markers	WHR	rs33999	5	141973349	G	C	-0.014	0.002	4.1e-13	8.3e-05	51.2
Adiposity Markers	WHR	rs13154197	5	142086694	G	A	0.022	0.003	1.1e-15	1.1e-04	66.4
Adiposity Markers	WHR	rs10463416	5	148572915	A	G	0.014	0.002	9.3e-14	9.2e-05	57.9
Adiposity Markers	WHR	rs2343813	5	149878840	T	C	0.019	0.003	8.6e-10	6.1e-05	38.4
Adiposity Markers	WHR	rs17703354	5	157986357	C	T	0.020	0.003	1.2e-09	6.0e-05	37.5
Adiposity Markers	WHR	rs7725967	5	171829850	C	T	0.011	0.002	5.1e-09	5.6e-05	35.4
Adiposity Markers	WHR	rs12657303	5	172142551	A	C	-0.011	0.002	7.9e-10	6.4e-05	40.1
Adiposity Markers	WHR	rs6861681	5	173362458	A	G	0.028	0.002	4.2e-49	3.0e-04	209.5
Adiposity Markers	WHR	rs6556301	5	176527577	T	G	0.021	0.002	2.0e-29	2.0e-04	131.0
Adiposity Markers	WHR	rs1445845	5	178507090	A	G	-0.010	0.002	3.5e-08	4.8e-05	30.0

Phenotype	Trait	SNP	Chr	Position (GRCh37/hg19)	EA	NEA	BETA	SE	P-value	R2	F
Adiposity Markers	WHR	rs11746028	5	180645425	T	C	-0.014	0.002	2.0e-09	5.5e-05	34.5
Adiposity Markers	WHR	rs2745581	6	1598227	A	G	0.011	0.002	1.7e-08	5.0e-05	30.5
Adiposity Markers	WHR	rs1294410	6	6738752	C	T	0.031	0.002	2.3e-69	4.2e-04	294.7
Adiposity Markers	WHR	rs6939362	6	7206383	A	C	-0.012	0.002	2.8e-11	7.0e-05	43.7
Adiposity Markers	WHR	rs6932767	6	14595873	T	G	0.015	0.002	4.3e-12	7.8e-05	49.0
Adiposity Markers	WHR	rs7744833	6	20581828	G	A	-0.012	0.002	1.4e-09	6.0e-05	37.9
Adiposity Markers	WHR	rs2294823	6	22596987	T	C	0.012	0.002	2.7e-09	5.9e-05	37.3
Adiposity Markers	WHR	rs13193424	6	25943646	C	G	-0.024	0.003	3.5e-16	1.2e-04	65.6
Adiposity Markers	WHR	rs4452638	6	27229265	A	G	-0.023	0.003	2.9e-17	1.1e-04	70.7
Adiposity Markers	WHR	rs4947336	6	28576143	A	T	-0.026	0.003	1.3e-18	1.3e-04	78.5
Adiposity Markers	WHR	rs2523395	6	29702510	A	G	0.010	0.002	1.0e-08	4.9e-05	33.9
Adiposity Markers	WHR	rs2523729	6	30137387	A	G	-0.026	0.003	1.1e-22	1.4e-04	97.7
Adiposity Markers	WHR	rs9264647	6	31238613	G	A	0.025	0.002	6.6e-41	2.8e-04	177.3
Adiposity Markers	WHR	rs2769	6	31321882	A	G	0.015	0.002	2.1e-09	6.2e-05	36.0
Adiposity Markers	WHR	rs9268153	6	32264179	T	C	-0.022	0.002	4.0e-22	1.5e-04	94.9
Adiposity Markers	WHR	rs12194148	6	32444198	T	G	0.023	0.002	3.8e-41	2.8e-04	189.5
Adiposity Markers	WHR	rs12214804	6	34188866	T	C	-0.042	0.003	5.0e-41	2.9e-04	179.2
Adiposity Markers	WHR	rs2814998	6	34625211	T	C	-0.017	0.002	4.9e-17	9.8e-05	67.9
Adiposity Markers	WHR	rs7751693	6	35695274	A	G	0.031	0.005	2.0e-11	6.7e-05	45.4
Adiposity Markers	WHR	rs13198178	6	41702227	C	G	0.031	0.004	1.2e-16	1.1e-04	69.3
Adiposity Markers	WHR	rs998584	6	43757896	A	C	0.049	0.002	1.2e-170	1.2e-03	820.7
Adiposity Markers	WHR	rs9472138	6	43811762	T	C	-0.021	0.002	4.1e-28	1.7e-04	117.6
Adiposity Markers	WHR	rs1934328	6	45465753	T	A	0.010	0.002	2.7e-08	5.0e-05	31.5
Adiposity Markers	WHR	rs12527712	6	80916967	T	C	0.034	0.003	6.8e-25	1.7e-04	105.5
Adiposity Markers	WHR	rs1902066	6	81346033	C	T	0.018	0.002	2.4e-23	1.6e-04	100.0
Adiposity Markers	WHR	rs13191833	6	81744850	C	T	-0.015	0.002	3.7e-10	6.1e-05	38.0
Adiposity Markers	WHR	rs1010437	6	85098237	A	G	-0.013	0.002	1.0e-12	7.4e-05	51.4
Adiposity Markers	WHR	rs9341990	6	85391568	G	A	-0.015	0.002	9.5e-17	1.1e-04	70.4
Adiposity Markers	WHR	rs2503100	6	100613915	G	A	0.024	0.002	4.9e-25	1.7e-04	104.2
Adiposity Markers	WHR	rs11757455	6	112522852	A	G	-0.022	0.004	2.2e-09	5.7e-05	35.7
Adiposity Markers	WHR	rs1322757	6	126216025	A	G	0.011	0.002	3.5e-08	5.1e-05	31.7
Adiposity Markers	WHR	rs2326201	6	126386403	C	G	0.012	0.002	7.5e-12	7.6e-05	47.5
Adiposity Markers	WHR	rs7753502	6	127205732	C	T	-0.021	0.002	1.3e-33	2.2e-04	149.7
Adiposity Markers	WHR	rs72959041	6	127454893	A	G	0.162	0.004	1.0e-200	2.5e-03	1362.3
Adiposity Markers	WHR	rs77097175	6	127472589	A	C	0.046	0.006	7.2e-15	1.1e-04	59.8
Adiposity Markers	WHR	rs9388766	6	130354855	C	T	0.017	0.002	4.0e-20	1.2e-04	80.1
Adiposity Markers	WHR	rs11154680	6	132779502	C	T	0.023	0.003	8.8e-12	7.6e-05	46.9
Adiposity Markers	WHR	rs6940715	6	133601845	C	A	-0.022	0.003	6.1e-14	8.7e-05	54.8
Adiposity Markers	WHR	rs634869	6	139831757	C	T	-0.023	0.002	2.6e-40	2.6e-04	181.5
Adiposity Markers	WHR	rs541091	6	160770552	A	G	-0.020	0.002	3.0e-26	2.0e-04	109.7
Adiposity Markers	WHR	rs2158828	7	14227425	G	A	0.014	0.002	3.4e-09	5.2e-05	34.5
Adiposity Markers	WHR	rs3807947	7	20424889	G	T	-0.014	0.002	7.5e-14	9.2e-05	57.9
Adiposity Markers	WHR	rs2391168	7	25862790	A	C	0.032	0.002	2.4e-49	3.3e-04	227.9
Adiposity Markers	WHR	rs2122823	7	25939161	T	C	0.021	0.002	1.8e-22	1.4e-04	97.2
Adiposity Markers	WHR	rs1534696	7	26397239	A	C	-0.024	0.002	3.2e-44	3.0e-04	206.0
Adiposity Markers	WHR	rs17437657	7	27249617	A	G	-0.037	0.003	2.1e-29	2.0e-04	127.1
Adiposity Markers	WHR	rs17502051	7	27305382	G	T	0.033	0.006	5.4e-09	5.6e-05	34.3
Adiposity Markers	WHR	rs849139	7	28179396	T	C	-0.013	0.002	4.1e-13	7.3e-05	50.6
Adiposity Markers	WHR	rs849140	7	28183702	C	T	-0.016	0.002	1.9e-19	1.2e-04	85.3
Adiposity Markers	WHR	rs12112380	7	30935900	C	T	0.021	0.004	1.7e-08	4.6e-05	31.7
Adiposity Markers	WHR	rs7811609	7	32930597	T	C	-0.012	0.002	4.9e-11	7.2e-05	45.2
Adiposity Markers	WHR	rs13223034	7	42701627	T	C	-0.013	0.002	3.7e-12	7.8e-05	49.0
Adiposity Markers	WHR	rs3110697	7	45955029	G	A	-0.012	0.002	1.1e-11	7.5e-05	46.7
Adiposity Markers	WHR	rs10441122	7	46604621	A	G	0.012	0.002	2.4e-09	5.6e-05	34.3
Adiposity Markers	WHR	rs2529411	7	50738482	C	G	-0.011	0.002	2.8e-09	6.1e-05	36.0
Adiposity Markers	WHR	rs7797307	7	68686127	C	G	-0.022	0.004	4.7e-08	4.8e-05	29.7
Adiposity Markers	WHR	rs55747707	7	73037366	A	G	-0.024	0.002	3.0e-25	1.9e-04	103.4
Adiposity Markers	WHR	rs10264590	7	77357748	G	A	-0.018	0.002	8.3e-23	1.5e-04	94.5
Adiposity Markers	WHR	rs917191	7	80570871	C	G	0.014	0.002	5.1e-14	9.4e-05	58.8
Adiposity Markers	WHR	rs13234914	7	84577411	G	A	0.013	0.002	1.3e-11	7.2e-05	45.4
Adiposity Markers	WHR	rs2023772	7	93131162	C	A	-0.012	0.002	1.4e-11	6.6e-05	45.8
Adiposity Markers	WHR	rs421168	7	101693884	A	G	-0.012	0.002	9.2e-11	7.1e-05	44.4

Phenotype	Trait	SNP	Chr	Position (GRCh37/hg19)	EA	NEA	BETA	SE	P-value	R2	F
Adiposity Markers	WHR	rs1144	7	104756355	C	T	0.015	0.002	5.6e-17	1.0e-04	69.4
Adiposity Markers	WHR	rs10953555	7	107581346	C	T	-0.010	0.002	3.0e-08	5.0e-05	31.5
Adiposity Markers	WHR	rs11766345	7	107611650	T	G	-0.031	0.003	1.5e-23	1.6e-04	99.4
Adiposity Markers	WHR	rs38911	7	116895163	A	G	0.013	0.002	1.5e-13	8.7e-05	54.6
Adiposity Markers	WHR	rs10249651	7	117064625	C	T	0.015	0.002	1.2e-16	9.6e-05	66.7
Adiposity Markers	WHR	rs2222543	7	120902185	C	G	-0.015	0.002	1.6e-15	1.1e-04	66.7
Adiposity Markers	WHR	rs7776617	7	121065112	T	A	-0.013	0.002	4.4e-10	6.5e-05	41.0
Adiposity Markers	WHR	rs2268382	7	130027037	C	A	-0.011	0.002	3.3e-08	4.9e-05	30.5
Adiposity Markers	WHR	rs1364422	7	130445981	T	C	0.016	0.002	1.1e-16	9.8e-05	68.3
Adiposity Markers	WHR	rs357438	7	138023288	C	G	-0.011	0.002	3.8e-09	5.9e-05	36.0
Adiposity Markers	WHR	rs7827182	8	8380471	C	G	-0.017	0.002	3.9e-19	1.3e-04	76.3
Adiposity Markers	WHR	rs330078	8	9155685	T	C	0.012	0.002	8.7e-10	6.0e-05	36.6
Adiposity Markers	WHR	rs13268133	8	9731470	C	T	0.012	0.002	2.5e-10	6.4e-05	39.9
Adiposity Markers	WHR	rs4321967	8	10641407	T	C	0.014	0.002	1.9e-16	1.0e-04	70.8
Adiposity Markers	WHR	rs804282	8	11611743	T	G	-0.010	0.002	1.3e-08	5.2e-05	32.7
Adiposity Markers	WHR	rs4841662	8	11843758	A	G	-0.015	0.002	1.4e-18	1.2e-04	79.9
Adiposity Markers	WHR	rs4316157	8	12632951	T	C	0.011	0.002	3.5e-09	5.5e-05	34.7
Adiposity Markers	WHR	rs15285	8	19824667	T	C	-0.011	0.002	4.4e-08	4.2e-05	28.6
Adiposity Markers	WHR	rs9644033	8	23610639	T	A	-0.022	0.002	3.4e-26	1.8e-04	111.8
Adiposity Markers	WHR	rs2012485	8	25374910	T	C	-0.029	0.004	2.7e-11	6.6e-05	44.3
Adiposity Markers	WHR	rs13257034	8	25774337	T	C	0.015	0.002	3.2e-15	9.4e-05	59.0
Adiposity Markers	WHR	rs7835016	8	25889501	A	C	0.012	0.002	1.2e-08	5.3e-05	33.2
Adiposity Markers	WHR	rs2874670	8	26245658	A	G	0.010	0.002	2.1e-08	5.1e-05	32.1
Adiposity Markers	WHR	rs12543555	8	69562014	G	A	0.014	0.002	1.8e-10	5.7e-05	39.3
Adiposity Markers	WHR	rs1018015	8	71919570	T	C	-0.012	0.002	6.9e-10	6.1e-05	38.6
Adiposity Markers	WHR	rs16937474	8	72086197	T	A	0.016	0.003	3.1e-08	4.8e-05	29.9
Adiposity Markers	WHR	rs4738141	8	72469742	G	A	0.026	0.002	9.6e-41	2.6e-04	171.6
Adiposity Markers	WHR	rs1455813	8	75995961	G	A	0.012	0.002	2.3e-09	5.5e-05	34.2
Adiposity Markers	WHR	rs7835845	8	89338155	T	C	0.015	0.002	2.8e-14	8.5e-05	59.0
Adiposity Markers	WHR	rs10087445	8	98936178	T	C	-0.013	0.002	3.7e-08	4.7e-05	29.3
Adiposity Markers	WHR	rs7003062	8	121358527	C	T	0.012	0.002	2.1e-11	7.7e-05	47.5
Adiposity Markers	WHR	rs10808546	8	126495818	T	C	-0.016	0.002	2.0e-20	1.3e-04	87.5
Adiposity Markers	WHR	rs13256367	8	128334900	C	A	-0.018	0.002	6.1e-22	1.5e-04	91.8
Adiposity Markers	WHR	rs2060145	8	129807905	T	C	0.012	0.002	3.9e-09	5.4e-05	33.6
Adiposity Markers	WHR	rs10100533	8	135706219	G	A	-0.014	0.002	8.4e-15	1.0e-04	63.1
Adiposity Markers	WHR	rs10963067	9	17367946	A	C	-0.021	0.003	5.9e-11	6.8e-05	42.7
Adiposity Markers	WHR	rs3731246	9	21971989	G	C	0.016	0.003	1.1e-08	4.7e-05	32.7
Adiposity Markers	WHR	rs16907277	9	93972658	G	A	0.023	0.003	9.1e-15	9.9e-05	61.8
Adiposity Markers	WHR	rs10992414	9	95448381	C	A	0.022	0.002	1.1e-21	1.5e-04	94.0
Adiposity Markers	WHR	rs2398893	9	96758342	G	A	-0.015	0.002	6.8e-16	9.3e-05	64.8
Adiposity Markers	WHR	rs7854560	9	98382950	T	C	0.012	0.002	5.9e-10	5.4e-05	37.3
Adiposity Markers	WHR	rs4743305	9	101760026	C	T	-0.011	0.002	1.1e-09	6.2e-05	38.7
Adiposity Markers	WHR	rs2777795	9	107672365	A	G	-0.027	0.003	2.7e-20	1.3e-04	87.3
Adiposity Markers	WHR	rs10991433	9	107726918	C	T	0.032	0.003	2.5e-29	1.8e-04	126.6
Adiposity Markers	WHR	rs12003255	9	107883159	C	T	-0.047	0.004	1.7e-25	1.8e-04	108.6
Adiposity Markers	WHR	rs12684047	9	111972671	A	T	-0.015	0.002	6.9e-11	6.6e-05	41.2
Adiposity Markers	WHR	rs4978959	9	113597938	T	C	0.011	0.002	3.3e-08	5.1e-05	31.7
Adiposity Markers	WHR	rs10980802	9	113918856	G	A	0.015	0.002	5.3e-18	1.2e-04	73.2
Adiposity Markers	WHR	rs10817896	9	119232655	T	C	0.013	0.002	3.3e-10	6.3e-05	39.7
Adiposity Markers	WHR	rs6478851	9	131561110	A	G	0.012	0.002	1.8e-09	6.0e-05	37.8
Adiposity Markers	WHR	rs7086377	10	3596526	G	T	-0.012	0.002	6.9e-11	7.0e-05	43.7
Adiposity Markers	WHR	rs12774134	10	4963327	T	C	-0.019	0.003	2.0e-12	7.1e-05	48.5
Adiposity Markers	WHR	rs7350438	10	5643511	T	C	-0.012	0.002	3.6e-10	5.9e-05	37.3
Adiposity Markers	WHR	rs1494204	10	27904321	T	C	-0.015	0.002	8.1e-16	1.1e-04	66.7
Adiposity Markers	WHR	rs12770117	10	30360965	G	A	-0.021	0.004	2.0e-08	5.2e-05	32.0
Adiposity Markers	WHR	rs2907794	10	32391109	A	G	-0.011	0.002	1.3e-08	4.6e-05	31.9
Adiposity Markers	WHR	rs11592754	10	32619572	C	A	0.021	0.002	3.2e-18	1.1e-04	74.0
Adiposity Markers	WHR	rs734187	10	33484696	A	G	-0.012	0.002	3.6e-09	5.6e-05	34.9
Adiposity Markers	WHR	rs1474921	10	34165699	A	G	0.016	0.002	2.0e-16	1.1e-04	66.6
Adiposity Markers	WHR	rs10761602	10	63813802	T	G	-0.012	0.002	1.4e-10	6.8e-05	42.2
Adiposity Markers	WHR	rs9415106	10	77295957	A	G	0.014	0.002	9.8e-13	8.0e-05	50.4
Adiposity Markers	WHR	rs780159	10	80907147	G	A	0.016	0.002	3.3e-20	1.2e-04	82.0

Phenotype	Trait	SNP	Chr	Position (GRCh37/hg19)	EA	NEA	BETA	SE	P-value	R2	F
Adiposity Markers	WHR	rs1250584	10	80976442	T	G	0.011	0.002	1.0e-10	6.7e-05	44.2
Adiposity Markers	WHR	rs10887759	10	89603424	A	G	0.017	0.002	1.1e-12	7.5e-05	52.0
Adiposity Markers	WHR	rs11186914	10	94062631	A	T	0.010	0.002	9.2e-09	5.3e-05	33.4
Adiposity Markers	WHR	rs11187537	10	95346805	C	G	0.016	0.002	2.4e-15	9.9e-05	60.2
Adiposity Markers	WHR	rs1223583	10	95756500	G	A	0.011	0.002	5.9e-11	6.3e-05	43.4
Adiposity Markers	WHR	rs61886306	10	95903461	C	T	0.014	0.002	2.2e-08	5.6e-05	30.9
Adiposity Markers	WHR	rs28408682	10	104403310	G	A	0.012	0.002	1.8e-10	7.5e-05	41.2
Adiposity Markers	WHR	rs4073980	10	114746580	G	C	0.020	0.002	1.5e-25	1.9e-04	106.4
Adiposity Markers	WHR	rs4751628	10	119315825	T	G	-0.013	0.002	6.2e-13	8.7e-05	54.6
Adiposity Markers	WHR	rs2254069	10	122875589	A	G	0.025	0.003	2.5e-20	1.4e-04	84.4
Adiposity Markers	WHR	rs1138714	11	825110	G	A	-0.015	0.002	3.4e-16	1.1e-04	67.6
Adiposity Markers	WHR	rs11042077	11	8825966	A	G	0.012	0.002	5.0e-11	6.8e-05	42.6
Adiposity Markers	WHR	rs12419064	11	10320608	G	A	0.014	0.002	4.5e-14	1.0e-04	55.1
Adiposity Markers	WHR	rs2957678	11	10407955	C	T	0.011	0.002	4.5e-10	6.2e-05	38.7
Adiposity Markers	WHR	rs7932891	11	10921512	G	A	-0.014	0.002	1.4e-12	7.9e-05	49.7
Adiposity Markers	WHR	rs2970332	11	14360435	A	G	0.016	0.002	1.0e-13	8.8e-05	55.2
Adiposity Markers	WHR	rs7928810	11	17372443	A	C	-0.011	0.002	1.4e-10	5.7e-05	39.4
Adiposity Markers	WHR	rs3114693	11	26127790	T	A	-0.010	0.002	2.3e-08	5.0e-05	30.0
Adiposity Markers	WHR	rs2301250	11	32458219	A	G	-0.012	0.002	5.6e-11	7.2e-05	45.2
Adiposity Markers	WHR	rs7114403	11	36361607	A	T	0.013	0.002	1.2e-12	7.9e-05	49.8
Adiposity Markers	WHR	rs34312154	11	47470345	A	G	0.019	0.003	3.2e-10	7.1e-05	39.3
Adiposity Markers	WHR	rs2513064	11	62172631	G	T	-0.012	0.002	4.1e-12	6.7e-05	46.7
Adiposity Markers	WHR	rs2509967	11	62312786	G	C	0.020	0.002	1.4e-26	1.8e-04	109.7
Adiposity Markers	WHR	rs2155324	11	62930682	G	A	-0.032	0.006	9.8e-09	5.3e-05	32.7
Adiposity Markers	WHR	rs2845885	11	63869062	T	C	-0.039	0.004	4.1e-28	1.7e-04	118.0
Adiposity Markers	WHR	rs3741378	11	65408937	T	C	-0.022	0.002	1.1e-19	1.2e-04	80.3
Adiposity Markers	WHR	rs7947391	11	66186882	G	A	0.010	0.002	1.7e-08	4.4e-05	30.2
Adiposity Markers	WHR	rs11263432	11	69247354	C	T	-0.019	0.002	2.4e-24	1.6e-04	101.1
Adiposity Markers	WHR	rs667515	11	69449076	C	G	0.011	0.002	2.2e-09	5.8e-05	36.0
Adiposity Markers	WHR	rs2186955	11	74375475	C	T	-0.012	0.002	1.5e-08	4.5e-05	31.3
Adiposity Markers	WHR	rs10502148	11	111647998	T	C	-0.023	0.002	5.9e-36	2.6e-04	163.3
Adiposity Markers	WHR	rs10891490	11	112885527	C	T	-0.010	0.002	8.9e-09	5.0e-05	34.6
Adiposity Markers	WHR	rs7943309	11	116773653	A	G	0.027	0.005	9.6e-09	4.8e-05	33.5
Adiposity Markers	WHR	rs7914	11	119180316	A	G	0.012	0.002	2.4e-08	5.1e-05	31.6
Adiposity Markers	WHR	rs747249	11	130271647	G	A	-0.011	0.002	1.3e-09	5.7e-05	36.0
Adiposity Markers	WHR	rs7300366	12	6652227	A	G	-0.011	0.002	2.5e-08	4.3e-05	29.7
Adiposity Markers	WHR	rs34322	12	12879570	C	T	-0.010	0.002	2.7e-08	4.9e-05	30.9
Adiposity Markers	WHR	rs7959150	12	26428063	G	A	0.018	0.002	2.2e-19	1.3e-04	81.0
Adiposity Markers	WHR	rs11048456	12	26463082	T	C	-0.034	0.002	1.0e-69	4.7e-04	324.0
Adiposity Markers	WHR	rs4964058	12	27522766	A	G	0.011	0.002	2.8e-10	6.3e-05	39.4
Adiposity Markers	WHR	rs11051005	12	30783475	G	A	-0.016	0.002	6.4e-16	9.1e-05	63.2
Adiposity Markers	WHR	rs2200155	12	33734935	A	G	-0.014	0.002	1.3e-13	8.4e-05	52.8
Adiposity Markers	WHR	rs12372031	12	34788305	C	G	-0.013	0.002	9.7e-13	8.6e-05	53.8
Adiposity Markers	WHR	rs11181857	12	38413255	G	C	-0.013	0.002	7.9e-13	8.6e-05	53.8
Adiposity Markers	WHR	rs10880823	12	45986738	C	T	0.013	0.002	5.7e-10	6.3e-05	39.5
Adiposity Markers	WHR	rs2544028	12	48216430	T	A	0.011	0.002	1.1e-09	6.3e-05	39.4
Adiposity Markers	WHR	rs1443512	12	54342684	C	A	-0.031	0.002	1.2e-53	3.4e-04	237.2
Adiposity Markers	WHR	rs2241938	12	54392449	C	T	-0.026	0.002	5.8e-26	1.8e-04	114.7
Adiposity Markers	WHR	rs1872636	12	54536534	G	C	0.012	0.002	4.0e-09	5.6e-05	34.8
Adiposity Markers	WHR	rs2293413	12	56096816	C	T	-0.011	0.002	5.0e-09	5.4e-05	34.0
Adiposity Markers	WHR	rs2277339	12	57146069	G	T	0.022	0.003	2.3e-14	9.7e-05	59.7
Adiposity Markers	WHR	rs10784510	12	66422753	C	T	-0.015	0.002	9.1e-16	8.9e-05	61.5
Adiposity Markers	WHR	rs10878367	12	66436097	A	T	0.019	0.002	5.8e-21	1.5e-04	90.2
Adiposity Markers	WHR	rs711076	12	77905207	C	T	0.012	0.002	1.5e-11	6.7e-05	46.7
Adiposity Markers	WHR	rs10745659	12	94092690	G	C	0.013	0.002	1.8e-12	7.9e-05	49.8
Adiposity Markers	WHR	rs7311622	12	98772975	T	C	-0.010	0.002	1.1e-08	5.3e-05	33.4
Adiposity Markers	WHR	rs2195243	12	102922986	C	G	-0.012	0.002	1.1e-08	4.7e-05	32.1
Adiposity Markers	WHR	rs4433659	12	107082496	T	G	0.014	0.002	6.9e-14	8.8e-05	55.1
Adiposity Markers	WHR	rs3764002	12	108618630	T	C	-0.018	0.002	1.9e-19	1.2e-04	78.3
Adiposity Markers	WHR	rs10850127	12	113512945	T	C	0.013	0.002	8.2e-11	7.0e-05	42.2
Adiposity Markers	WHR	rs1896313	12	115395280	C	T	0.010	0.002	8.6e-09	4.9e-05	33.9
Adiposity Markers	WHR	rs11043307	12	122494845	T	C	0.017	0.002	4.1e-14	8.9e-05	55.9

Phenotype	Trait	SNP	Chr	Position (GRCh37/hg19)	EA	NEA	BETA	SE	P-value	R2	F
Adiposity Markers	WHR	rs6489156	12	123100397	T	C	-0.011	0.002	3.3e-08	5.0e-05	31.4
Adiposity Markers	WHR	rs601339	12	123174743	G	A	-0.025	0.002	1.5e-27	2.0e-04	121.0
Adiposity Markers	WHR	rs11057239	12	123768246	T	C	0.042	0.005	7.6e-17	1.0e-04	68.9
Adiposity Markers	WHR	rs10744161	12	124231355	G	A	-0.011	0.002	5.6e-09	5.6e-05	35.3
Adiposity Markers	WHR	rs7133378	12	124409502	A	G	-0.039	0.002	4.0e-98	6.6e-04	459.9
Adiposity Markers	WHR	rs863750	12	124505444	T	C	0.037	0.002	4.2e-101	6.9e-04	481.4
Adiposity Markers	WHR	rs7954697	12	125335253	A	C	0.010	0.002	1.9e-08	4.8e-05	33.2
Adiposity Markers	WHR	rs7973997	12	128756938	T	C	0.011	0.002	2.2e-08	5.0e-05	31.1
Adiposity Markers	WHR	rs12823266	12	131441717	G	A	-0.013	0.002	2.4e-10	6.4e-05	38.9
Adiposity Markers	WHR	rs12828318	12	133766122	G	A	-0.017	0.002	3.8e-14	8.3e-05	56.6
Adiposity Markers	WHR	rs1547149	13	22476288	G	A	0.012	0.002	6.6e-11	6.7e-05	41.9
Adiposity Markers	WHR	rs1340819	13	29145323	C	A	-0.010	0.002	2.2e-08	4.6e-05	32.1
Adiposity Markers	WHR	rs1360485	13	31031884	T	C	0.011	0.002	8.0e-10	5.2e-05	36.0
Adiposity Markers	WHR	rs10507524	13	44684600	C	T	0.018	0.003	2.7e-09	5.5e-05	34.5
Adiposity Markers	WHR	rs2475837	13	50535863	T	C	0.019	0.003	1.2e-12	7.5e-05	51.2
Adiposity Markers	WHR	rs1638703	13	51088356	C	G	0.014	0.002	1.4e-11	7.0e-05	43.8
Adiposity Markers	WHR	rs797486	13	51221618	A	C	0.037	0.003	4.4e-46	3.0e-04	205.8
Adiposity Markers	WHR	rs17067999	13	78163787	T	C	-0.016	0.003	1.4e-10	6.3e-05	39.8
Adiposity Markers	WHR	rs1577099	13	93894245	T	C	0.010	0.002	3.1e-08	4.8e-05	30.2
Adiposity Markers	WHR	rs9557378	13	100747566	A	G	0.011	0.002	2.5e-08	5.1e-05	31.9
Adiposity Markers	WHR	rs664532	13	110932363	C	T	-0.013	0.002	1.4e-12	9.0e-05	49.7
Adiposity Markers	WHR	rs61964262	13	110937654	T	C	-0.015	0.003	7.5e-09	6.0e-05	32.8
Adiposity Markers	WHR	rs4773173	13	111025118	G	A	-0.015	0.002	4.2e-17	1.1e-04	72.2
Adiposity Markers	WHR	rs3210043	14	23779877	A	C	-0.015	0.002	2.2e-09	6.1e-05	37.0
Adiposity Markers	WHR	rs12435790	14	35154381	G	A	-0.021	0.004	1.2e-09	6.2e-05	37.4
Adiposity Markers	WHR	rs1253682	14	52463153	T	C	0.010	0.002	1.7e-09	5.4e-05	37.4
Adiposity Markers	WHR	rs17125001	14	52541199	T	C	0.026	0.005	4.6e-08	4.9e-05	29.9
Adiposity Markers	WHR	rs7160860	14	54339493	C	T	0.014	0.002	7.6e-09	5.3e-05	33.5
Adiposity Markers	WHR	rs1190982	14	58815839	C	T	-0.016	0.002	2.5e-16	1.1e-04	70.0
Adiposity Markers	WHR	rs1254319	14	60903757	A	G	-0.012	0.002	7.1e-11	5.8e-05	40.6
Adiposity Markers	WHR	rs4902632	14	69149428	T	A	-0.020	0.002	2.4e-16	1.2e-04	70.1
Adiposity Markers	WHR	rs2810073	14	71383848	C	T	0.012	0.002	1.9e-09	6.8e-05	36.6
Adiposity Markers	WHR	rs862031	14	74991855	G	A	0.012	0.002	7.2e-12	6.9e-05	47.5
Adiposity Markers	WHR	rs7492628	14	91547136	G	C	0.023	0.002	4.9e-32	2.3e-04	145.3
Adiposity Markers	WHR	rs8003238	14	98345604	A	G	0.013	0.002	2.6e-12	7.9e-05	49.8
Adiposity Markers	WHR	rs12441543	15	31689543	A	G	-0.019	0.002	8.7e-22	1.4e-04	89.3
Adiposity Markers	WHR	rs1826947	15	36105651	A	G	-0.010	0.002	7.8e-09	4.5e-05	31.5
Adiposity Markers	WHR	rs2701523	15	37384284	G	A	-0.013	0.002	3.2e-10	6.6e-05	41.6
Adiposity Markers	WHR	rs2289328	15	40705417	A	G	0.015	0.002	3.5e-11	6.5e-05	44.8
Adiposity Markers	WHR	rs1105881	15	42072530	G	C	0.016	0.002	1.1e-16	1.1e-04	67.4
Adiposity Markers	WHR	rs12908370	15	47905717	C	T	0.011	0.002	1.9e-08	5.1e-05	31.7
Adiposity Markers	WHR	rs17703883	15	51530097	C	T	0.015	0.002	7.3e-13	8.0e-05	50.3
Adiposity Markers	WHR	rs17648151	15	51694635	A	G	-0.037	0.007	2.2e-08	5.1e-05	31.4
Adiposity Markers	WHR	rs12595496	15	56528806	G	A	0.026	0.003	1.0e-23	1.4e-04	97.7
Adiposity Markers	WHR	rs4775348	15	61313142	A	T	0.018	0.003	1.2e-08	5.3e-05	33.3
Adiposity Markers	WHR	rs12440695	15	62435156	C	T	0.010	0.002	6.3e-09	4.8e-05	32.7
Adiposity Markers	WHR	rs2058914	15	63831984	A	G	-0.012	0.002	1.6e-10	5.6e-05	38.6
Adiposity Markers	WHR	rs1440372	15	67033151	C	T	0.016	0.002	9.5e-16	9.6e-05	66.6
Adiposity Markers	WHR	rs12442323	15	67654919	T	C	-0.015	0.002	1.7e-11	8.3e-05	45.9
Adiposity Markers	WHR	rs750460	15	74241506	A	G	-0.013	0.002	4.8e-13	8.3e-05	52.2
Adiposity Markers	WHR	rs59888683	15	79049464	T	C	0.014	0.002	1.8e-09	6.4e-05	35.0
Adiposity Markers	WHR	rs1516797	15	89410314	G	T	-0.011	0.002	2.8e-08	5.0e-05	30.5
Adiposity Markers	WHR	rs8030277	15	94029153	T	A	0.013	0.002	6.0e-12	7.7e-05	48.3
Adiposity Markers	WHR	rs13379908	15	96176302	C	T	0.013	0.002	3.7e-08	4.9e-05	30.7
Adiposity Markers	WHR	rs13379794	15	98367100	A	G	0.011	0.002	4.3e-09	5.9e-05	36.7
Adiposity Markers	WHR	rs2684787	15	99505062	T	C	0.013	0.002	4.7e-10	6.1e-05	38.3
Adiposity Markers	WHR	rs11641742	16	427784	A	G	0.011	0.002	1.7e-08	5.2e-05	30.5
Adiposity Markers	WHR	rs258281	16	2191734	G	A	0.014	0.002	5.0e-09	5.4e-05	33.1
Adiposity Markers	WHR	rs12325187	16	3364997	G	C	-0.013	0.002	8.3e-10	5.8e-05	36.6
Adiposity Markers	WHR	rs3747577	16	4415696	G	C	-0.021	0.002	3.3e-26	1.8e-04	115.6
Adiposity Markers	WHR	rs876476	16	11150248	A	G	0.011	0.002	1.3e-08	5.0e-05	31.4
Adiposity Markers	WHR	rs7102	16	11642242	C	T	0.012	0.002	1.4e-10	5.9e-05	40.8

Phenotype	Trait	SNP	Chr	Position (GRCh37/hg19)	EA	NEA	BETA	SE	P-value	R2	F
Adiposity Markers	WHR	rs2047937	16	49864791	T	C	-0.015	0.002	7.3e-18	1.1e-04	74.8
Adiposity Markers	WHR	rs8054299	16	53498655	G	C	-0.015	0.002	5.8e-14	9.6e-05	59.0
Adiposity Markers	WHR	rs16957415	16	67418957	G	A	-0.031	0.004	3.3e-13	8.4e-05	53.0
Adiposity Markers	WHR	rs400223	16	73809928	T	C	0.010	0.002	9.2e-09	5.4e-05	33.4
Adiposity Markers	WHR	rs17767383	16	79744401	A	G	0.010	0.002	2.3e-08	4.7e-05	32.7
Adiposity Markers	WHR	rs2925979	16	81534790	C	T	-0.026	0.002	7.3e-46	2.8e-04	194.5
Adiposity Markers	WHR	rs7198287	16	85258191	T	C	-0.014	0.002	1.0e-09	6.2e-05	38.8
Adiposity Markers	WHR	rs1169644	16	86537032	A	G	0.012	0.002	3.9e-08	4.8e-05	30.2
Adiposity Markers	WHR	rs8076112	17	1612199	C	A	-0.014	0.002	4.1e-09	5.7e-05	35.0
Adiposity Markers	WHR	rs7225453	17	3960620	C	T	0.015	0.002	2.7e-10	6.3e-05	39.7
Adiposity Markers	WHR	rs727428	17	7537792	C	T	-0.016	0.002	4.3e-18	1.2e-04	77.0
Adiposity Markers	WHR	rs1561	17	8263334	T	C	0.022	0.004	9.3e-10	5.5e-05	38.1
Adiposity Markers	WHR	rs12936587	17	17543722	A	G	-0.018	0.002	9.2e-27	1.7e-04	117.1
Adiposity Markers	WHR	rs2280777	17	18041507	T	C	-0.012	0.002	5.0e-11	6.9e-05	43.0
Adiposity Markers	WHR	rs7213608	17	21279289	T	C	-0.012	0.002	3.1e-09	5.8e-05	36.6
Adiposity Markers	WHR	rs8076739	17	27714587	T	C	-0.010	0.002	2.7e-09	5.1e-05	35.3
Adiposity Markers	WHR	rs3087591	17	29630970	G	A	-0.011	0.002	1.0e-09	5.2e-05	36.0
Adiposity Markers	WHR	rs8070260	17	34866915	G	A	-0.010	0.002	3.4e-08	4.8e-05	30.2
Adiposity Markers	WHR	rs3744070	17	36924339	A	G	0.016	0.003	3.5e-08	4.9e-05	30.8
Adiposity Markers	WHR	rs2293158	17	40447558	C	T	0.015	0.002	3.6e-14	8.9e-05	54.8
Adiposity Markers	WHR	rs9905939	17	40905383	C	T	-0.012	0.002	5.8e-11	6.9e-05	43.0
Adiposity Markers	WHR	rs10445337	17	44067400	C	T	0.017	0.002	4.1e-16	9.7e-05	67.1
Adiposity Markers	WHR	rs4794033	17	47358481	A	G	-0.019	0.003	8.7e-11	6.7e-05	41.8
Adiposity Markers	WHR	rs8074638	17	53731579	A	G	0.016	0.002	2.1e-11	7.4e-05	46.6
Adiposity Markers	WHR	rs227733	17	54769174	A	C	0.013	0.002	7.5e-11	6.5e-05	41.0
Adiposity Markers	WHR	rs757608	17	59497277	G	A	-0.019	0.002	1.1e-26	1.7e-04	116.2
Adiposity Markers	WHR	rs730213	17	59502635	A	G	0.013	0.002	6.6e-13	8.5e-05	53.0
Adiposity Markers	WHR	rs2854152	17	61986027	G	A	-0.013	0.002	3.9e-12	7.8e-05	49.0
Adiposity Markers	WHR	rs4247361	17	65396360	A	G	-0.014	0.002	4.4e-13	8.4e-05	52.0
Adiposity Markers	WHR	rs8066985	17	68453345	G	A	-0.023	0.002	2.0e-40	2.6e-04	179.9
Adiposity Markers	WHR	rs1029645	17	70295760	A	G	-0.013	0.002	7.2e-13	8.2e-05	51.4
Adiposity Markers	WHR	rs9909443	17	73308346	T	C	-0.014	0.002	1.2e-10	6.9e-05	43.2
Adiposity Markers	WHR	rs10512606	17	74211208	C	A	0.028	0.003	2.4e-18	1.1e-04	75.5
Adiposity Markers	WHR	rs2376585	17	76417883	C	T	0.018	0.002	5.5e-14	9.2e-05	57.9
Adiposity Markers	WHR	rs4239275	17	79923718	C	T	-0.012	0.002	1.7e-10	6.2e-05	40.8
Adiposity Markers	WHR	rs3810068	18	2846499	C	T	-0.023	0.002	5.8e-33	2.5e-04	144.0
Adiposity Markers	WHR	rs614841	18	2850631	G	C	0.011	0.002	3.3e-09	5.2e-05	35.3
Adiposity Markers	WHR	rs7235010	18	20724810	A	G	0.019	0.002	5.6e-19	1.1e-04	78.4
Adiposity Markers	WHR	rs1893781	18	42443643	A	C	0.018	0.003	3.3e-10	6.2e-05	39.1
Adiposity Markers	WHR	rs1026186	18	42741410	A	G	-0.011	0.002	2.2e-09	5.5e-05	34.7
Adiposity Markers	WHR	rs1539871	18	45467806	G	A	-0.010	0.002	3.9e-08	4.8e-05	30.2
Adiposity Markers	WHR	rs7242873	18	46885025	G	A	0.024	0.003	2.5e-14	8.2e-05	57.2
Adiposity Markers	WHR	rs12454712	18	60845884	C	T	-0.017	0.002	1.3e-20	1.4e-04	89.2
Adiposity Markers	WHR	rs12459350	19	2176586	G	A	-0.014	0.002	2.4e-16	9.9e-05	68.8
Adiposity Markers	WHR	rs8887	19	4502201	C	T	0.011	0.002	3.2e-09	6.0e-05	36.7
Adiposity Markers	WHR	rs3786680	19	7183551	T	C	-0.021	0.003	1.1e-12	7.3e-05	50.5
Adiposity Markers	WHR	rs4542783	19	8642160	C	T	-0.010	0.002	2.0e-08	4.8e-05	30.9
Adiposity Markers	WHR	rs889129	19	10070954	A	T	-0.020	0.003	9.3e-12	7.9e-05	46.6
Adiposity Markers	WHR	rs737338	19	11347657	T	C	0.026	0.004	3.9e-09	5.5e-05	34.2
Adiposity Markers	WHR	rs10418336	19	13115480	A	G	-0.033	0.005	7.4e-13	8.1e-05	50.5
Adiposity Markers	WHR	rs3745348	19	17212410	T	C	0.014	0.002	9.5e-14	7.7e-05	53.5
Adiposity Markers	WHR	rs439223	19	18126453	A	G	0.020	0.003	6.3e-09	5.5e-05	34.3
Adiposity Markers	WHR	rs12608504	19	18389135	G	A	-0.026	0.002	3.1e-47	3.0e-04	205.4
Adiposity Markers	WHR	rs11670392	19	18607780	T	C	-0.012	0.002	4.0e-08	4.8e-05	30.2
Adiposity Markers	WHR	rs73001065	19	19460541	C	G	0.022	0.004	3.7e-09	6.3e-05	35.0
Adiposity Markers	WHR	rs12461296	19	33774591	A	C	-0.013	0.002	3.3e-08	5.0e-05	30.0
Adiposity Markers	WHR	rs7251505	19	33802542	A	G	-0.046	0.003	1.3e-51	3.3e-04	230.0
Adiposity Markers	WHR	rs3786897	19	33893008	G	A	0.028	0.002	3.0e-58	3.9e-04	269.3
Adiposity Markers	WHR	rs4420638	19	45422946	G	A	-0.023	0.002	6.8e-22	1.5e-04	91.0
Adiposity Markers	WHR	rs73942938	19	46162346	C	T	0.015	0.002	4.5e-09	6.2e-05	34.1
Adiposity Markers	WHR	rs838145	19	49248730	A	G	-0.012	0.002	2.4e-10	6.7e-05	40.8
Adiposity Markers	WHR	rs2864436	19	55986637	C	T	0.013	0.002	3.4e-10	6.8e-05	40.7

Phenotype	Trait	SNP	Chr	Position (GRCh37/hg19)	EA	NEA	BETA	SE	P-value	R2	F
Adiposity Markers	WHR	rs6084945	20	549364	T	C	0.012	0.002	3.8e-08	5.2e-05	31.3
Adiposity Markers	WHR	rs6053604	20	5664999	C	A	0.014	0.002	3.8e-09	5.6e-05	34.5
Adiposity Markers	WHR	rs2145272	20	6626218	A	G	-0.025	0.002	4.0e-43	2.7e-04	188.3
Adiposity Markers	WHR	rs170986	20	6760431	A	C	0.015	0.003	1.5e-08	5.4e-05	31.1
Adiposity Markers	WHR	rs6040229	20	10883131	G	A	0.011	0.002	5.3e-10	6.2e-05	38.7
Adiposity Markers	WHR	rs6047259	20	21098319	T	C	0.010	0.002	4.3e-09	4.8e-05	33.4
Adiposity Markers	WHR	rs143384	20	34025756	G	A	-0.018	0.002	9.9e-25	1.6e-04	109.6
Adiposity Markers	WHR	rs6124221	20	38881281	A	G	-0.013	0.002	3.1e-10	6.3e-05	39.1
Adiposity Markers	WHR	rs910071	20	39213710	T	C	-0.017	0.002	6.3e-19	1.3e-04	81.0
Adiposity Markers	WHR	rs1997833	20	39690342	C	T	0.014	0.002	1.6e-13	7.6e-05	52.8
Adiposity Markers	WHR	rs2236519	20	45529571	A	G	0.030	0.002	4.1e-58	3.9e-04	246.0
Adiposity Markers	WHR	rs6066130	20	45569541	A	G	-0.021	0.002	3.7e-32	2.2e-04	138.7
Adiposity Markers	WHR	rs17789520	20	48839705	A	G	0.017	0.003	4.6e-08	5.0e-05	30.4
Adiposity Markers	WHR	rs6096940	20	51034311	G	A	-0.016	0.002	1.7e-17	1.1e-04	70.0
Adiposity Markers	WHR	rs910382	20	51699189	A	G	-0.019	0.002	2.8e-25	1.7e-04	107.9
Adiposity Markers	WHR	rs1293398	20	51781745	T	C	0.021	0.003	4.5e-10	6.3e-05	39.2
Adiposity Markers	WHR	rs1328757	20	56135199	T	C	0.014	0.002	3.4e-15	9.8e-05	61.4
Adiposity Markers	WHR	rs6062344	20	62696024	T	C	-0.015	0.002	6.2e-16	1.2e-04	68.5
Adiposity Markers	WHR	rs2898237	21	36763769	G	A	-0.012	0.002	7.5e-11	6.2e-05	43.0
Adiposity Markers	WHR	rs2836179	21	39544159	A	G	-0.017	0.002	7.2e-20	1.4e-04	86.1
Adiposity Markers	WHR	rs6518232	21	46788299	C	A	-0.011	0.002	4.4e-08	5.1e-05	31.7
Adiposity Markers	WHR	rs2839108	21	47535248	T	C	-0.015	0.002	1.7e-13	8.9e-05	55.5
Adiposity Markers	WHR	rs8142329	22	27647289	G	A	0.016	0.003	1.6e-10	5.9e-05	40.3
Adiposity Markers	WHR	rs2283847	22	28181399	T	C	0.012	0.002	1.2e-10	6.2e-05	41.5
Adiposity Markers	WHR	rs6005861	22	29154237	G	A	0.041	0.005	2.7e-14	8.5e-05	58.2
Adiposity Markers	WHR	rs2294239	22	29449477	G	A	-0.024	0.002	4.0e-44	2.9e-04	204.3
Adiposity Markers	WHR	rs134630	22	29480742	G	A	0.010	0.002	4.5e-08	4.8e-05	30.0
Adiposity Markers	WHR	rs13058399	22	30694428	G	A	0.020	0.003	3.6e-13	8.6e-05	53.8
Adiposity Markers	WHR	rs6518754	22	32097775	C	T	0.010	0.002	3.6e-09	5.1e-05	35.3
Adiposity Markers	WHR	rs4821387	22	35669441	G	A	-0.015	0.002	4.0e-15	9.3e-05	58.2
Adiposity Markers	WHR	rs4821897	22	39835587	G	A	-0.012	0.002	9.9e-09	5.0e-05	31.6
Adiposity Markers	WHR	rs801593	22	47195050	C	G	-0.012	0.002	6.7e-10	6.1e-05	37.8
Smoking Status	CigDay	rs2072659	1	154548521	G	C	-0.036	0.005	1.7e-12	1.8e-04	46.6
Smoking Status	CigDay	rs2084533	3	16872929	T	C	0.017	0.003	1.2e-08	1.2e-04	32.1
Smoking Status	CigDay	rs7431710	3	48935583	A	G	-0.017	0.003	1.8e-09	1.4e-04	36.3
Smoking Status	CigDay	rs11725618	4	67053769	C	T	0.019	0.003	4.7e-09	1.3e-04	34.4
Smoking Status	CigDay	rs787362	4	67904931	A	T	0.015	0.003	4.5e-08	1.1e-04	29.9
Smoking Status	CigDay	rs806798	6	26214473	C	T	-0.016	0.003	2.5e-08	1.2e-04	30.9
Smoking Status	CigDay	rs215600	7	32333642	A	G	-0.025	0.003	1.1e-17	2.8e-04	73.5
Smoking Status	CigDay	rs73229090	8	27442127	A	C	0.028	0.004	2.4e-10	1.5e-04	39.8
Smoking Status	CigDay	rs58379124	8	42579203	C	T	0.034	0.003	9.0e-25	3.9e-04	103.7
Smoking Status	CigDay	rs790564	8	64604218	C	A	-0.020	0.003	4.0e-11	1.7e-04	43.7
Smoking Status	CigDay	rs75596189	9	136468701	T	C	0.033	0.004	4.5e-14	2.1e-04	56.6
Smoking Status	CigDay	rs3025383	9	136502369	C	T	-0.029	0.004	2.2e-16	2.5e-04	66.2
Smoking Status	CigDay	rs7951365	11	16377044	C	T	0.020	0.003	6.6e-11	1.6e-04	42.4
Smoking Status	CigDay	rs75494138	11	46465361	T	C	0.029	0.005	1.5e-08	1.2e-04	31.8
Smoking Status	CigDay	rs7928017	11	113448762	A	C	-0.016	0.003	3.1e-09	1.3e-04	34.7
Smoking Status	CigDay	rs632811	15	59155050	G	A	-0.019	0.003	1.0e-08	1.5e-04	33.6
Smoking Status	CigDay	rs8034191	15	78806023	C	T	0.091	0.003	1.0e-200	3.7e-03	962.7
Smoking Status	CigDay	rs184659738	15	79051716	A	G	-0.092	0.012	1.2e-14	2.2e-04	56.6
Smoking Status	CigDay	rs117541407	15	79057455	A	C	-0.047	0.007	2.7e-11	1.6e-04	42.3
Smoking Status	CigDay	rs8034658	15	79165441	T	G	-0.026	0.003	1.4e-16	2.6e-04	67.9
Smoking Status	CigDay	rs2386571	16	52074123	C	A	-0.016	0.003	1.0e-08	1.2e-04	32.7
Smoking Status	CigDay	rs4785587	16	89772619	A	G	-0.017	0.003	1.3e-09	1.4e-04	36.5
Smoking Status	CigDay	rs895330	19	4060707	G	C	-0.020	0.004	2.7e-08	1.1e-04	30.2
Smoking Status	CigDay	rs147893869	19	41321074	G	C	-0.092	0.009	3.5e-24	4.0e-04	104.3
Smoking Status	CigDay	rs11671669	19	41324392	A	G	-0.080	0.008	1.8e-22	3.7e-04	95.2
Smoking Status	CigDay	rs56113850	19	41353107	C	T	0.056	0.003	1.1e-81	1.4e-03	370.3
Smoking Status	CigDay	rs115994486	19	41511306	A	G	-0.084	0.015	1.6e-08	1.3e-04	33.5
Smoking Status	CigDay	rs707265	19	41524087	G	A	-0.017	0.003	4.6e-09	1.4e-04	35.0
Smoking Status	CigDay	rs2424888	20	31047533	A	G	0.017	0.003	2.8e-09	1.3e-04	35.1
Smoking Status	CigDay	rs2273500	20	61986949	C	T	0.035	0.004	2.5e-18	2.9e-04	76.0

Phenotype	Trait	SNP	Chr	Position (GRCh37/hg19)	EA	NEA	BETA	SE	P-value	R2	F
Smoking Status	Lifetime smk	rs1149335	1	7530341	C	T	0.008	0.001	3.6e-08	6.6e-05	30.4
Smoking Status	Lifetime smk	rs4949465	1	32178489	T	C	0.012	0.002	1.7e-08	6.9e-05	31.8
Smoking Status	Lifetime smk	rs11210887	1	44076019	G	A	-0.011	0.002	9.6e-14	1.2e-04	55.5
Smoking Status	Lifetime smk	rs12137221	1	50182298	C	T	0.011	0.002	1.6e-10	8.8e-05	40.9
Smoking Status	Lifetime smk	rs11208774	1	66410109	C	T	0.010	0.001	5.9e-12	1.0e-04	47.4
Smoking Status	Lifetime smk	rs7546987	1	73848505	C	T	-0.012	0.001	2.1e-16	1.5e-04	67.6
Smoking Status	Lifetime smk	rs7553348	1	75005067	G	A	-0.010	0.001	5.2e-12	1.0e-04	47.6
Smoking Status	Lifetime smk	rs1008078	1	91189731	C	T	0.009	0.001	8.2e-11	9.1e-05	42.2
Smoking Status	Lifetime smk	rs1931262	1	96174912	T	C	0.008	0.001	3.5e-08	6.6e-05	30.4
Smoking Status	Lifetime smk	rs11801576	1	99425374	T	C	0.010	0.002	2.3e-10	8.7e-05	40.2
Smoking Status	Lifetime smk	rs9435341	1	107616641	T	C	-0.008	0.001	1.3e-08	7.0e-05	32.3
Smoking Status	Lifetime smk	rs10918701	1	162090536	G	A	-0.008	0.001	2.1e-08	6.8e-05	31.4
Smoking Status	Lifetime smk	rs62107261	2	422144	T	C	-0.021	0.003	8.2e-11	9.1e-05	42.2
Smoking Status	Lifetime smk	rs2867112	2	651349	T	G	-0.015	0.002	4.8e-15	1.3e-04	61.3
Smoking Status	Lifetime smk	rs12469094	2	22967228	A	C	-0.008	0.001	2.1e-08	6.8e-05	31.4
Smoking Status	Lifetime smk	rs62136969	2	44359876	G	C	-0.025	0.004	6.4e-10	8.3e-05	38.2
Smoking Status	Lifetime smk	rs7569203	2	45154418	A	C	0.011	0.002	7.4e-13	1.1e-04	51.4
Smoking Status	Lifetime smk	rs4671357	2	60136176	T	C	0.009	0.001	1.1e-11	1.0e-04	46.1
Smoking Status	Lifetime smk	rs1553933	2	60509080	C	T	0.008	0.001	2.7e-08	6.7e-05	30.9
Smoking Status	Lifetime smk	rs12151765	2	62777035	A	G	-0.013	0.002	2.3e-09	7.7e-05	35.7
Smoking Status	Lifetime smk	rs11123962	2	104157011	T	G	0.009	0.001	1.6e-10	8.8e-05	40.9
Smoking Status	Lifetime smk	rs62155874	2	105973094	A	G	0.017	0.002	5.2e-16	1.4e-04	65.7
Smoking Status	Lifetime smk	rs3811038	2	113240183	T	C	0.010	0.002	8.9e-10	8.1e-05	37.6
Smoking Status	Lifetime smk	rs1858152	2	145874327	G	A	0.009	0.001	6.6e-11	9.2e-05	42.6
Smoking Status	Lifetime smk	rs2890772	2	146175106	G	T	0.014	0.001	2.1e-22	2.0e-04	94.8
Smoking Status	Lifetime smk	rs62175972	2	161362830	T	C	-0.022	0.004	1.7e-08	6.9e-05	31.8
Smoking Status	Lifetime smk	rs3769949	2	166199284	T	A	0.008	0.001	2.5e-09	7.7e-05	35.5
Smoking Status	Lifetime smk	rs13009008	2	174043233	A	G	-0.009	0.001	4.6e-09	7.4e-05	34.3
Smoking Status	Lifetime smk	rs4432411	2	182069004	G	T	0.010	0.002	6.8e-11	9.2e-05	42.6
Smoking Status	Lifetime smk	rs56059523	2	204123832	T	C	0.013	0.002	2.1e-09	7.7e-05	35.8
Smoking Status	Lifetime smk	rs9870893	3	16863267	T	G	0.009	0.001	1.3e-09	7.9e-05	36.7
Smoking Status	Lifetime smk	rs6778080	3	49317338	T	C	-0.011	0.002	1.3e-12	1.1e-04	50.4
Smoking Status	Lifetime smk	rs9309773	3	77593147	T	C	-0.008	0.001	1.9e-08	6.8e-05	31.6
Smoking Status	Lifetime smk	rs78526269	3	83602635	T	C	-0.010	0.002	1.5e-09	7.9e-05	36.6
Smoking Status	Lifetime smk	rs421983	3	84892866	T	C	-0.009	0.001	3.3e-10	8.5e-05	39.5
Smoking Status	Lifetime smk	rs1549979	3	85460131	C	T	-0.008	0.001	3.7e-08	6.6e-05	30.3
Smoking Status	Lifetime smk	rs2117762	3	107832118	T	C	0.009	0.001	4.5e-11	9.4e-05	43.4
Smoking Status	Lifetime smk	rs62280815	3	131073888	A	G	0.011	0.002	1.7e-08	6.9e-05	31.8
Smoking Status	Lifetime smk	rs9842947	3	157412246	C	T	0.009	0.001	3.1e-09	7.6e-05	35.1
Smoking Status	Lifetime smk	rs35780153	4	2845274	C	T	-0.009	0.002	1.6e-09	7.9e-05	36.4
Smoking Status	Lifetime smk	rs61796681	4	23678196	A	T	0.013	0.002	4.2e-08	6.5e-05	30.0
Smoking Status	Lifetime smk	rs317021	4	35418368	T	A	0.012	0.002	1.1e-10	9.0e-05	41.7
Smoking Status	Lifetime smk	rs72678864	4	112422145	G	A	-0.012	0.002	1.6e-11	9.8e-05	45.4
Smoking Status	Lifetime smk	rs17314804	4	140946828	C	T	-0.009	0.001	3.9e-09	7.5e-05	34.7
Smoking Status	Lifetime smk	rs17576594	4	147952241	G	A	-0.011	0.002	1.7e-12	1.1e-04	49.8
Smoking Status	Lifetime smk	rs13106038	4	154688164	G	A	-0.009	0.001	2.2e-09	7.7e-05	35.8
Smoking Status	Lifetime smk	rs11948770	5	13246336	T	C	0.010	0.002	4.9e-10	8.4e-05	38.7
Smoking Status	Lifetime smk	rs71627581	5	43161351	G	A	-0.013	0.002	1.6e-09	7.9e-05	36.4
Smoking Status	Lifetime smk	rs2068701	5	50814632	A	G	-0.008	0.001	2.3e-09	7.7e-05	35.7
Smoking Status	Lifetime smk	rs6891239	5	87769693	C	T	-0.008	0.001	2.9e-08	6.6e-05	30.8
Smoking Status	Lifetime smk	rs72784111	5	106631814	T	C	-0.014	0.002	7.6e-09	7.2e-05	33.4
Smoking Status	Lifetime smk	rs329122	5	133864599	G	A	-0.010	0.001	7.3e-12	1.0e-04	46.9
Smoking Status	Lifetime smk	rs1363374	5	165187776	C	T	-0.008	0.001	6.4e-09	7.3e-05	33.7
Smoking Status	Lifetime smk	rs13178466	5	166748010	C	T	-0.008	0.001	4.8e-08	6.4e-05	29.8
Smoking Status	Lifetime smk	rs986391	5	166993972	G	A	-0.011	0.001	9.4e-15	1.3e-04	60.0
Smoking Status	Lifetime smk	rs11134664	5	170324778	G	A	0.009	0.001	5.0e-10	8.4e-05	38.7
Smoking Status	Lifetime smk	rs1059490	6	26171250	T	C	-0.010	0.001	2.0e-11	9.7e-05	45.0
Smoking Status	Lifetime smk	rs3734431	6	52843429	A	G	0.015	0.003	2.0e-08	6.8e-05	31.5
Smoking Status	Lifetime smk	rs7775552	6	67475569	A	G	0.008	0.001	2.8e-09	7.6e-05	35.3
Smoking Status	Lifetime smk	rs10945142	6	69519309	G	A	0.009	0.002	2.9e-08	6.6e-05	30.8
Smoking Status	Lifetime smk	rs459809	6	111653167	T	C	-0.013	0.002	2.6e-12	1.1e-04	49.0
Smoking Status	Lifetime smk	rs1108879	7	3556132	G	A	-0.009	0.001	1.3e-10	8.9e-05	41.2

Phenotype	Trait	SNP	Chr	Position (GRCh37/hg19)	EA	NEA	BETA	SE	P-value	R2	F
Smoking Status	Lifetime smk	rs1860225	7	32263711	A	G	0.011	0.001	3.0e-15	1.3e-04	62.3
Smoking Status	Lifetime smk	rs11762736	7	96630637	A	G	-0.009	0.001	1.7e-09	7.8e-05	36.3
Smoking Status	Lifetime smk	rs6962772	7	99081730	A	G	-0.011	0.002	7.8e-09	7.2e-05	33.3
Smoking Status	Lifetime smk	rs10282292	7	111092478	C	T	-0.009	0.001	5.9e-10	8.3e-05	38.4
Smoking Status	Lifetime smk	rs8180817	7	114047542	G	C	-0.008	0.001	5.2e-09	7.4e-05	34.1
Smoking Status	Lifetime smk	rs1358393	7	115086427	G	T	-0.011	0.001	3.4e-14	1.2e-04	57.5
Smoking Status	Lifetime smk	rs10250850	7	117545340	C	A	0.010	0.001	6.8e-14	1.2e-04	56.1
Smoking Status	Lifetime smk	rs4731925	7	132664757	C	T	0.008	0.001	2.6e-08	6.7e-05	31.0
Smoking Status	Lifetime smk	rs35169606	8	9604066	T	G	-0.009	0.001	1.2e-09	8.0e-05	36.9
Smoking Status	Lifetime smk	rs11786713	8	10838413	C	G	-0.008	0.001	4.3e-08	6.5e-05	30.0
Smoking Status	Lifetime smk	rs11783093	8	27425349	C	T	-0.016	0.002	1.2e-16	1.5e-04	68.6
Smoking Status	Lifetime smk	rs2062882	8	91839576	G	A	0.008	0.001	1.1e-08	7.0e-05	32.6
Smoking Status	Lifetime smk	rs71532325	8	95627966	C	T	-0.008	0.001	2.3e-08	6.8e-05	31.2
Smoking Status	Lifetime smk	rs7047015	9	3151423	C	G	0.008	0.001	2.4e-09	7.7e-05	35.6
Smoking Status	Lifetime smk	rs7024957	9	82444254	C	T	-0.009	0.001	9.6e-10	8.1e-05	37.4
Smoking Status	Lifetime smk	rs1246263	9	86762039	C	G	0.009	0.002	4.5e-09	7.4e-05	34.4
Smoking Status	Lifetime smk	rs1927902	9	120518991	T	C	-0.009	0.002	2.4e-08	6.7e-05	31.2
Smoking Status	Lifetime smk	rs1359358	9	122073899	C	T	-0.009	0.001	2.7e-10	8.6e-05	39.9
Smoking Status	Lifetime smk	rs6478697	9	127889460	T	G	-0.008	0.001	5.3e-09	7.4e-05	34.1
Smoking Status	Lifetime smk	rs2416963	9	128241414	C	T	0.011	0.001	5.0e-15	1.3e-04	61.3
Smoking Status	Lifetime smk	rs113382419	9	136463019	C	A	0.028	0.002	3.0e-37	3.5e-04	162.7
Smoking Status	Lifetime smk	rs1112278	10	8803133	A	G	0.010	0.002	2.8e-10	8.6e-05	39.8
Smoking Status	Lifetime smk	rs2675638	10	63576286	G	A	-0.008	0.001	1.3e-09	8.0e-05	36.8
Smoking Status	Lifetime smk	rs11000477	10	74730322	C	T	-0.008	0.001	3.3e-08	6.6e-05	30.5
Smoking Status	Lifetime smk	rs12266933	10	92912588	G	A	0.013	0.002	6.1e-09	7.3e-05	33.8
Smoking Status	Lifetime smk	rs7087984	10	104380686	G	A	-0.008	0.001	3.4e-09	7.6e-05	35.0
Smoking Status	Lifetime smk	rs28606370	10	104670832	C	A	0.013	0.001	1.1e-18	1.7e-04	77.9
Smoking Status	Lifetime smk	rs11023819	11	16011483	A	G	-0.008	0.001	1.5e-09	7.9e-05	36.5
Smoking Status	Lifetime smk	rs56030184	11	17067849	C	T	-0.010	0.002	2.0e-09	7.8e-05	36.0
Smoking Status	Lifetime smk	rs35891966	11	20129311	G	A	-0.016	0.003	6.9e-09	7.3e-05	33.6
Smoking Status	Lifetime smk	rs17309874	11	27667236	G	A	0.011	0.002	9.7e-13	1.1e-04	50.9
Smoking Status	Lifetime smk	rs4391802	11	28674592	A	G	-0.010	0.002	1.4e-11	9.9e-05	45.6
Smoking Status	Lifetime smk	rs112282219	11	46632809	G	A	0.023	0.004	3.8e-11	9.4e-05	43.7
Smoking Status	Lifetime smk	rs7127712	11	112874603	A	T	0.015	0.001	9.0e-27	2.5e-04	114.7
Smoking Status	Lifetime smk	rs551337	11	121546188	T	G	0.008	0.001	2.1e-08	6.8e-05	31.4
Smoking Status	Lifetime smk	rs74086911	12	50015942	G	A	-0.015	0.003	2.1e-08	6.8e-05	31.4
Smoking Status	Lifetime smk	rs7297175	12	56473808	T	C	0.008	0.001	6.6e-09	7.3e-05	33.6
Smoking Status	Lifetime smk	rs1405451	12	75417818	G	A	-0.011	0.002	6.1e-11	9.2e-05	42.8
Smoking Status	Lifetime smk	rs12831617	12	84758368	C	T	0.009	0.002	1.9e-08	6.8e-05	31.6
Smoking Status	Lifetime smk	rs9571652	13	67338075	T	C	-0.008	0.001	1.0e-08	7.1e-05	32.8
Smoking Status	Lifetime smk	rs7333559	13	100546450	G	A	-0.011	0.002	3.2e-10	8.6e-05	39.6
Smoking Status	Lifetime smk	rs860326	14	57342912	C	T	-0.008	0.001	2.7e-09	7.6e-05	35.4
Smoking Status	Lifetime smk	rs850269	14	57401178	T	C	-0.008	0.001	3.1e-08	6.6e-05	30.7
Smoking Status	Lifetime smk	rs72723308	14	77485365	C	A	-0.008	0.001	5.8e-09	7.3e-05	33.9
Smoking Status	Lifetime smk	rs77653640	14	98643863	G	A	0.010	0.002	3.3e-08	6.6e-05	30.5
Smoking Status	Lifetime smk	rs3742365	14	104198251	T	C	0.011	0.001	2.5e-14	1.3e-04	58.1
Smoking Status	Lifetime smk	rs11622651	14	104620077	G	A	-0.011	0.002	1.2e-12	1.1e-04	50.5
Smoking Status	Lifetime smk	rs35175834	15	47680815	G	A	0.016	0.002	4.6e-22	2.0e-04	93.3
Smoking Status	Lifetime smk	rs8039398	15	47730870	T	C	0.011	0.001	2.7e-14	1.3e-04	57.9
Smoking Status	Lifetime smk	rs4887111	15	74028285	A	G	-0.008	0.001	1.4e-08	6.9e-05	32.1
Smoking Status	Lifetime smk	rs938682	15	78896547	G	A	0.021	0.002	1.9e-37	3.5e-04	163.5
Smoking Status	Lifetime smk	rs12438734	15	79052135	T	C	-0.014	0.003	4.2e-08	6.5e-05	30.1
Smoking Status	Lifetime smk	rs35755781	15	97514655	G	A	0.009	0.001	2.1e-09	7.8e-05	35.9
Smoking Status	Lifetime smk	rs2017500	15	99196112	G	A	0.008	0.001	6.7e-09	7.3e-05	33.6
Smoking Status	Lifetime smk	rs4984916	16	749057	G	A	-0.009	0.002	3.4e-08	6.6e-05	30.5
Smoking Status	Lifetime smk	rs6497754	16	24710801	G	A	0.009	0.002	9.8e-09	7.1e-05	32.9
Smoking Status	Lifetime smk	rs889398	16	69556715	C	T	-0.009	0.001	6.3e-11	9.2e-05	42.7
Smoking Status	Lifetime smk	rs112178027	17	27564013	C	T	0.011	0.002	1.6e-09	7.9e-05	36.4
Smoking Status	Lifetime smk	rs732083	17	37834367	G	A	-0.008	0.001	1.5e-08	6.9e-05	32.1
Smoking Status	Lifetime smk	rs28635466	17	46983820	G	A	-0.008	0.002	2.0e-08	6.8e-05	31.5
Smoking Status	Lifetime smk	rs9889262	17	47398070	T	A	0.008	0.001	4.0e-08	6.5e-05	30.2
Smoking Status	Lifetime smk	rs67596067	17	50333733	G	A	0.009	0.001	1.2e-09	8.0e-05	37.0

Phenotype	Trait	SNP	Chr	Position (GRCh37/hg19)	EA	NEA	BETA	SE	P-value	R2	F
Smoking Status	Lifetime smk	rs62098013	18	50863861	G	A	0.009	0.001	4.1e-09	7.5e-05	34.6
Smoking Status	Lifetime smk	rs71367545	18	77576337	G	A	0.010	0.002	1.4e-09	7.9e-05	36.7
Smoking Status	Lifetime smk	rs12986231	19	18469017	T	C	-0.009	0.002	1.8e-08	6.8e-05	31.7
Smoking Status	Lifetime smk	rs4814873	20	19616429	C	T	-0.010	0.002	2.9e-09	7.6e-05	35.2
Smoking Status	Lifetime smk	rs6050215	20	24948737	C	T	0.026	0.004	5.7e-11	9.3e-05	42.9
Smoking Status	Lifetime smk	rs845984	20	26137161	G	A	0.025	0.004	8.9e-10	8.1e-05	37.6
Smoking Status	Lifetime smk	rs6057527	20	29513209	C	G	-0.010	0.002	2.2e-10	8.7e-05	40.3
Smoking Status	Lifetime smk	rs6119897	20	31145415	G	A	0.013	0.002	3.6e-15	1.3e-04	61.9
Smoking Status	Lifetime smk	rs12480534	20	44761485	T	G	0.009	0.002	9.0e-09	7.1e-05	33.1
Smoking Status	Lifetime smk	rs348811	20	59029492	G	A	0.008	0.001	1.3e-08	7.0e-05	32.3
Smoking Status	Lifetime smk	rs45577732	20	61983934	C	G	0.027	0.003	1.5e-26	2.5e-04	113.7
Smoking Status	Lifetime smk	rs6089904	20	62018289	A	T	-0.020	0.003	1.2e-08	7.0e-05	32.5
Smoking Status	Lifetime smk	rs147412694	21	40702786	G	A	0.012	0.002	2.9e-09	7.6e-05	35.3
Smoking Status	Lifetime smk	rs2838834	21	46665208	C	T	0.009	0.002	6.3e-10	8.3e-05	38.2
Smoking Status	Lifetime smk	rs136233	22	31212410	A	G	0.010	0.002	1.8e-08	6.8e-05	31.7
Smoking Status	Lifetime smk	rs202645	22	41798520	A	G	0.010	0.002	3.9e-09	7.5e-05	34.7
Smoking Status	SmkInit	rs301805	1	8481016	G	T	0.010	0.002	2.8e-09	5.2e-05	32.7
Smoking Status	SmkInit	rs3001723	1	44037685	A	G	0.015	0.002	8.1e-18	9.4e-05	59.4
Smoking Status	SmkInit	rs1343432	1	49396989	C	G	0.010	0.002	2.0e-08	3.9e-05	24.3
Smoking Status	SmkInit	rs6669839	1	50625979	T	C	0.012	0.002	3.4e-09	5.1e-05	31.8
Smoking Status	SmkInit	rs2186122	1	66470206	T	A	0.013	0.002	3.6e-13	7.7e-05	48.8
Smoking Status	SmkInit	rs7555507	1	73766037	T	C	-0.011	0.002	1.1e-11	5.9e-05	37.2
Smoking Status	SmkInit	rs2344508	1	74999713	A	G	-0.009	0.002	8.8e-10	4.3e-05	27.5
Smoking Status	SmkInit	rs2050586	1	87905828	C	G	-0.009	0.002	3.0e-08	3.9e-05	24.7
Smoking Status	SmkInit	rs12042107	1	91196176	C	T	-0.009	0.002	4.2e-10	3.7e-05	23.5
Smoking Status	SmkInit	rs12027999	1	154206358	C	T	-0.014	0.003	5.8e-10	4.5e-05	28.7
Smoking Status	SmkInit	rs2046850	1	210304319	T	C	-0.011	0.002	3.0e-08	3.7e-05	23.7
Smoking Status	SmkInit	rs6728726	2	623976	C	T	0.016	0.002	6.7e-14	7.1e-05	45.2
Smoking Status	SmkInit	rs1004787	2	45159091	A	G	0.012	0.002	5.3e-17	7.7e-05	48.5
Smoking Status	SmkInit	rs10490159	2	51341259	T	C	0.012	0.002	2.6e-09	6.6e-05	41.8
Smoking Status	SmkInit	rs1518393	2	58171220	C	A	0.009	0.002	2.0e-08	3.8e-05	23.8
Smoking Status	SmkInit	rs7585579	2	60024857	G	C	0.008	0.002	1.9e-09	3.4e-05	19.6
Smoking Status	SmkInit	rs1029984	2	60526747	T	G	0.010	0.002	6.3e-09	5.2e-05	32.7
Smoking Status	SmkInit	rs266047	2	104088751	A	G	-0.013	0.002	3.4e-16	9.8e-05	56.7
Smoking Status	SmkInit	rs35702515	2	137542847	T	G	0.011	0.002	2.4e-09	3.9e-05	24.8
Smoking Status	SmkInit	rs6756883	2	146015334	A	G	0.009	0.002	3.2e-09	4.1e-05	26.0
Smoking Status	SmkInit	rs13030994	2	146143090	A	G	0.016	0.002	3.6e-24	1.3e-04	79.6
Smoking Status	SmkInit	rs1445649	2	155682556	C	T	0.010	0.002	1.7e-11	4.6e-05	28.9
Smoking Status	SmkInit	rs12474587	2	162802993	T	G	0.011	0.002	1.3e-14	6.1e-05	38.5
Smoking Status	SmkInit	rs6433897	2	182034448	C	T	0.010	0.002	3.2e-08	3.8e-05	24.2
Smoking Status	SmkInit	rs2107300	2	200937901	G	C	-0.013	0.003	3.3e-08	4.5e-05	28.5
Smoking Status	SmkInit	rs4674993	2	226332033	G	A	-0.011	0.002	1.3e-08	4.1e-05	25.9
Smoking Status	SmkInit	rs11721059	3	5725560	T	C	0.009	0.002	2.2e-08	4.0e-05	25.6
Smoking Status	SmkInit	rs11716779	3	47606215	G	A	0.008	0.002	4.9e-08	2.7e-05	17.4
Smoking Status	SmkInit	rs12632110	3	50224225	G	A	-0.010	0.002	4.8e-10	4.7e-05	29.8
Smoking Status	SmkInit	rs2276825	3	52886605	C	T	0.010	0.002	2.4e-08	3.9e-05	24.5
Smoking Status	SmkInit	rs11712680	3	75009019	C	A	-0.013	0.002	3.5e-09	5.0e-05	31.4
Smoking Status	SmkInit	rs6788098	3	85624131	T	A	-0.013	0.002	1.9e-17	7.5e-05	47.4
Smoking Status	SmkInit	rs7644190	3	85905418	C	G	-0.010	0.002	4.6e-11	4.8e-05	29.9
Smoking Status	SmkInit	rs9866543	3	85981081	C	T	0.011	0.002	2.2e-08	3.4e-05	21.1
Smoking Status	SmkInit	rs1154693	3	117804154	G	A	0.015	0.002	3.1e-11	5.4e-05	34.5
Smoking Status	SmkInit	rs292071	4	28456089	C	T	0.010	0.002	4.1e-09	4.3e-05	27.1
Smoking Status	SmkInit	rs993700	4	67825894	C	T	-0.012	0.002	1.5e-09	5.0e-05	31.7
Smoking Status	SmkInit	rs1160685	4	94052854	G	C	0.010	0.002	7.2e-09	4.9e-05	30.8
Smoking Status	SmkInit	rs13136052	4	139711726	T	G	-0.008	0.002	3.4e-08	3.4e-05	21.2
Smoking Status	SmkInit	rs1355334	4	140887493	A	T	0.010	0.002	9.6e-11	4.8e-05	30.3
Smoking Status	SmkInit	rs10001365	4	147797214	A	G	-0.011	0.002	6.7e-12	5.5e-05	34.7
Smoking Status	SmkInit	rs6893752	5	60374912	G	A	-0.010	0.002	3.3e-09	4.1e-05	25.8
Smoking Status	SmkInit	rs4571506	5	87756918	T	C	-0.013	0.002	1.1e-14	8.0e-05	50.7
Smoking Status	SmkInit	rs27003	5	94202167	C	T	0.012	0.002	2.6e-10	6.1e-05	38.4
Smoking Status	SmkInit	rs12186738	5	103816655	T	G	-0.013	0.002	3.4e-11	4.7e-05	29.5
Smoking Status	SmkInit	rs17534089	5	106829219	G	A	-0.015	0.003	5.0e-10	5.6e-05	35.2

Phenotype	Trait	SNP	Chr	Position (GRCh37/hg19)	EA	NEA	BETA	SE	P-value	R2	F
Smoking Status	SmkInit	rs1385108	5	154839646	T	C	0.012	0.002	3.0e-09	5.4e-05	34.2
Smoking Status	SmkInit	rs11135030	5	157743295	T	C	0.008	0.002	4.4e-09	2.9e-05	18.2
Smoking Status	SmkInit	rs4044321	5	166989513	G	A	-0.013	0.002	6.1e-14	7.9e-05	50.1
Smoking Status	SmkInit	rs222449	6	52916062	T	A	-0.012	0.002	1.1e-08	4.3e-05	27.3
Smoking Status	SmkInit	rs10498846	6	67405337	T	C	0.009	0.002	6.6e-09	4.1e-05	25.9
Smoking Status	SmkInit	rs10945141	6	69470709	A	G	0.008	0.002	4.2e-08	2.7e-05	17.2
Smoking Status	SmkInit	rs12662631	6	93842446	T	C	0.011	0.002	4.3e-08	5.3e-05	33.4
Smoking Status	SmkInit	rs9401770	6	98748008	A	G	0.011	0.002	3.5e-12	4.6e-05	29.2
Smoking Status	SmkInit	rs846799	6	101270345	G	A	-0.009	0.002	3.9e-09	4.2e-05	26.5
Smoking Status	SmkInit	rs3800227	6	108994161	G	A	0.010	0.002	1.9e-08	4.0e-05	25.0
Smoking Status	SmkInit	rs240963	6	111644332	C	T	-0.018	0.002	2.2e-17	8.3e-05	52.3
Smoking Status	SmkInit	rs4236259	7	1708080	G	T	-0.011	0.002	3.4e-12	6.3e-05	40.1
Smoking Status	SmkInit	rs4721112	7	1889466	C	T	-0.010	0.002	1.9e-09	4.4e-05	27.5
Smoking Status	SmkInit	rs4549681	7	3406478	G	A	-0.010	0.002	2.3e-10	5.0e-05	28.8
Smoking Status	SmkInit	rs12112638	7	69735251	G	A	-0.011	0.002	1.3e-09	4.6e-05	28.9
Smoking Status	SmkInit	rs11768481	7	96629103	A	C	-0.010	0.002	7.0e-10	4.5e-05	28.5
Smoking Status	SmkInit	rs12333760	7	99185406	C	T	-0.013	0.002	1.4e-09	4.5e-05	28.5
Smoking Status	SmkInit	rs10233018	7	117523709	G	A	0.013	0.002	2.7e-14	8.8e-05	55.6
Smoking Status	SmkInit	rs10279261	7	133589846	A	G	-0.010	0.002	5.0e-09	4.6e-05	29.2
Smoking Status	SmkInit	rs1565735	8	27426077	A	T	-0.018	0.002	3.4e-17	9.5e-05	60.1
Smoking Status	SmkInit	rs13261666	8	59814666	T	G	-0.011	0.002	3.9e-14	6.5e-05	41.2
Smoking Status	SmkInit	rs12545053	8	65073605	G	A	0.010	0.002	2.4e-08	5.1e-05	32.4
Smoking Status	SmkInit	rs2631024	8	91995577	G	A	-0.011	0.002	1.2e-08	4.4e-05	27.8
Smoking Status	SmkInit	rs6471291	8	92678041	G	T	-0.008	0.002	3.2e-08	3.3e-05	20.7
Smoking Status	SmkInit	rs1899896	8	93201036	T	C	0.012	0.002	1.0e-11	6.0e-05	38.3
Smoking Status	SmkInit	rs4543592	9	3014254	C	T	0.009	0.002	7.5e-10	4.0e-05	25.1
Smoking Status	SmkInit	rs7024924	9	8282399	C	T	0.011	0.002	4.0e-08	3.2e-05	20.3
Smoking Status	SmkInit	rs17234745	9	11081182	C	T	-0.011	0.003	1.5e-08	2.7e-05	17.1
Smoking Status	SmkInit	rs2378662	9	86707289	A	G	0.009	0.002	4.2e-09	3.7e-05	23.5
Smoking Status	SmkInit	rs10905461	10	8803551	C	T	-0.011	0.002	7.3e-09	4.2e-05	26.7
Smoking Status	SmkInit	rs10159545	10	21766969	G	C	0.013	0.002	1.8e-12	7.3e-05	45.9
Smoking Status	SmkInit	rs7921378	10	63674885	C	G	-0.013	0.002	8.3e-13	7.8e-05	49.3
Smoking Status	SmkInit	rs7901883	10	103186838	A	G	-0.010	0.002	8.0e-09	3.4e-05	21.4
Smoking Status	SmkInit	rs12356821	10	104563808	C	G	0.014	0.003	6.3e-15	4.8e-05	30.3
Smoking Status	SmkInit	rs9423279	10	125680419	G	C	-0.008	0.002	3.2e-08	2.9e-05	18.4
Smoking Status	SmkInit	rs4523689	11	7950797	G	A	-0.008	0.002	1.5e-08	2.8e-05	17.5
Smoking Status	SmkInit	rs6265	11	27679916	T	C	-0.014	0.002	3.8e-12	6.2e-05	39.1
Smoking Status	SmkInit	rs4275621	11	28652996	G	A	-0.009	0.002	3.1e-09	3.8e-05	24.2
Smoking Status	SmkInit	rs7929518	11	85980958	G	A	0.011	0.002	1.6e-08	4.5e-05	28.4
Smoking Status	SmkInit	rs117695734	11	111992273	G	A	-0.027	0.005	3.8e-10	5.1e-05	32.3
Smoking Status	SmkInit	rs7938812	11	112911004	G	T	0.018	0.002	2.7e-33	1.7e-04	105.6
Smoking Status	SmkInit	rs11057005	12	16748721	G	A	-0.010	0.002	4.8e-09	5.3e-05	33.4
Smoking Status	SmkInit	rs4759228	12	56508409	C	G	-0.010	0.002	3.6e-08	4.1e-05	26.0
Smoking Status	SmkInit	rs7969559	12	69655167	G	A	-0.010	0.002	7.3e-10	3.8e-05	24.0
Smoking Status	SmkInit	rs1971318	12	121389500	T	C	0.013	0.002	7.1e-09	4.2e-05	26.7
Smoking Status	SmkInit	rs3904512	13	38357471	A	G	-0.009	0.002	3.2e-09	3.8e-05	24.0
Smoking Status	SmkInit	rs9540729	13	66947124	T	A	-0.008	0.002	3.8e-08	3.1e-05	19.8
Smoking Status	SmkInit	rs7322872	13	100548329	T	C	-0.011	0.002	3.6e-09	3.8e-05	24.3
Smoking Status	SmkInit	rs76214862	14	29500130	C	A	-0.012	0.002	4.0e-08	4.4e-05	27.9
Smoking Status	SmkInit	rs74011413	15	47659864	T	C	0.015	0.003	6.1e-10	5.1e-05	32.2
Smoking Status	SmkInit	rs962950	15	47685416	T	A	0.012	0.002	1.8e-12	6.2e-05	39.4
Smoking Status	SmkInit	rs1435741	15	47935843	A	G	0.013	0.002	2.6e-16	9.0e-05	56.7
Smoking Status	SmkInit	rs12441907	15	83922387	A	C	-0.013	0.002	1.1e-10	5.1e-05	32.1
Smoking Status	SmkInit	rs7197072	16	717085	T	C	-0.010	0.002	2.8e-09	3.6e-05	22.9
Smoking Status	SmkInit	rs12923427	16	17575065	T	C	-0.010	0.002	4.4e-08	3.2e-05	20.3
Smoking Status	SmkInit	rs4785836	16	65604652	C	T	-0.010	0.002	2.3e-08	4.5e-05	28.2
Smoking Status	SmkInit	rs11658881	17	2072949	G	A	0.009	0.002	2.4e-08	4.4e-05	27.6
Smoking Status	SmkInit	rs11078713	17	7795972	G	A	-0.008	0.002	2.2e-08	3.4e-05	21.3
Smoking Status	SmkInit	rs7224742	17	30657058	T	C	-0.008	0.002	1.4e-08	3.1e-05	19.7
Smoking Status	SmkInit	rs11873164	18	42659922	T	C	-0.014	0.002	1.3e-08	4.7e-05	29.6
Smoking Status	SmkInit	rs6508144	18	50026142	G	C	-0.009	0.002	8.0e-09	3.8e-05	24.1
Smoking Status	SmkInit	rs62098013	18	50863861	A	G	0.011	0.002	3.7e-08	5.4e-05	34.0

Phenotype	Trait	SNP	Chr	Position (GRCh37/hg19)	EA	NEA	BETA	SE	P-value	R2	F
Smoking Status	SmkInit	rs11872397	18	72535282	A	G	-0.012	0.002	1.4e-09	5.4e-05	33.9
Smoking Status	SmkInit	rs76608582	19	4474725	A	C	-0.027	0.005	1.9e-09	5.3e-05	33.6
Smoking Status	SmkInit	rs6050446	20	25195509	G	A	0.032	0.005	1.6e-08	5.7e-05	35.7
Smoking Status	SmkInit	rs1555445	20	31175258	T	A	0.009	0.002	3.7e-09	3.7e-05	23.1
Smoking Status	SmkInit	rs56820925	20	54387374	T	C	-0.010	0.002	1.7e-08	5.6e-05	31.9
Smoking Status	SmkInit	rs117143374	21	40555561	C	T	0.013	0.003	2.8e-08	3.3e-05	21.1
Smoking Status	SmkInit	rs134529	22	28781758	C	T	-0.008	0.002	4.8e-08	3.1e-05	19.8
Drinking Status	DrnkWk	rs10753661	1	165119792	A	G	-0.011	0.002	4.2e-08	5.5e-05	29.2
Drinking Status	DrnkWk	rs28680958	1	173848808	A	G	-0.014	0.002	9.8e-09	6.1e-05	32.9
Drinking Status	DrnkWk	rs1260326	2	27730940	C	T	0.023	0.002	3.3e-33	2.6e-04	141.3
Drinking Status	DrnkWk	rs528301	2	45154908	A	G	0.016	0.002	1.2e-15	1.2e-04	64.0
Drinking Status	DrnkWk	rs6739804	2	63269604	C	T	-0.013	0.002	4.7e-10	7.2e-05	38.5
Drinking Status	DrnkWk	rs4233567	2	144272376	T	C	-0.013	0.002	3.8e-10	7.3e-05	39.1
Drinking Status	DrnkWk	rs28732378	3	85403892	G	A	-0.016	0.002	2.2e-14	1.1e-04	56.4
Drinking Status	DrnkWk	rs28712821	4	39413780	A	G	0.028	0.002	1.1e-46	3.8e-04	203.7
Drinking Status	DrnkWk	rs16854020	4	42117559	A	G	0.018	0.003	4.8e-10	7.2e-05	38.8
Drinking Status	DrnkWk	rs144502847	4	98452892	A	T	-0.170	0.019	9.2e-19	1.6e-04	79.2
Drinking Status	DrnkWk	rs1727284	4	98979582	G	C	-0.229	0.035	5.1e-11	1.0e-04	42.3
Drinking Status	DrnkWk	rs71612659	4	99691047	A	G	-0.046	0.005	8.6e-25	1.9e-04	104.0
Drinking Status	DrnkWk	rs192787596	4	99738259	A	T	-0.325	0.044	4.2e-13	1.2e-04	55.8
Drinking Status	DrnkWk	rs1229984	4	100239319	C	T	0.209	0.007	1.0e-200	1.8e-03	964.4
Drinking Status	DrnkWk	rs192550	4	100286188	T	G	0.041	0.007	1.3e-09	6.9e-05	37.0
Drinking Status	DrnkWk	rs114112910	4	100313350	G	A	0.044	0.008	1.9e-08	5.9e-05	31.5
Drinking Status	DrnkWk	rs148926542	4	101307196	G	A	-0.179	0.027	2.5e-08	9.5e-05	43.0
Drinking Status	DrnkWk	rs13135092	4	103198082	G	A	-0.034	0.004	2.1e-19	1.5e-04	81.4
Drinking Status	DrnkWk	rs331939	4	143654889	A	G	-0.012	0.002	4.5e-09	6.4e-05	34.1
Drinking Status	DrnkWk	rs4916723	5	87854395	C	A	-0.012	0.002	8.1e-09	6.2e-05	33.4
Drinking Status	DrnkWk	rs55872084	5	155902003	T	G	0.013	0.002	2.0e-08	6.0e-05	32.0
Drinking Status	DrnkWk	rs10085696	7	69783020	G	A	-0.016	0.002	1.2e-10	7.7e-05	41.3
Drinking Status	DrnkWk	rs2299409	7	103812171	A	G	-0.010	0.002	4.8e-08	5.5e-05	29.3
Drinking Status	DrnkWk	rs6951574	7	153489744	C	T	0.014	0.002	4.4e-11	8.1e-05	43.4
Drinking Status	DrnkWk	rs28601761	8	126500031	G	C	0.012	0.002	7.6e-09	6.2e-05	33.3
Drinking Status	DrnkWk	rs55932213	9	108755622	G	A	0.013	0.002	1.8e-08	5.9e-05	31.5
Drinking Status	DrnkWk	rs2049045	11	27694241	C	G	-0.014	0.003	4.0e-08	5.5e-05	29.8
Drinking Status	DrnkWk	rs4752999	11	47428565	T	C	-0.015	0.002	2.0e-12	9.2e-05	49.1
Drinking Status	DrnkWk	rs4309187	11	113412443	C	A	0.015	0.002	1.4e-12	9.4e-05	50.3
Drinking Status	DrnkWk	rs17542254	11	113655696	G	A	0.013	0.002	9.0e-10	7.0e-05	37.5
Drinking Status	DrnkWk	rs7106546	11	116079170	T	G	0.013	0.002	1.3e-08	6.2e-05	33.2
Drinking Status	DrnkWk	rs682011	11	121544285	C	T	0.011	0.002	1.5e-08	6.0e-05	32.1
Drinking Status	DrnkWk	rs1387766	12	92081800	A	G	-0.011	0.002	4.8e-08	5.5e-05	29.8
Drinking Status	DrnkWk	rs34704785	13	68117681	T	C	-0.011	0.002	4.5e-08	5.3e-05	28.4
Drinking Status	DrnkWk	rs1123285	14	57274519	G	C	-0.013	0.002	1.4e-09	6.9e-05	37.3
Drinking Status	DrnkWk	rs6573197	14	58724208	T	C	-0.012	0.002	9.3e-09	6.1e-05	33.0
Drinking Status	DrnkWk	rs28929474	14	94844947	T	C	-0.048	0.007	2.4e-11	8.2e-05	44.0
Drinking Status	DrnkWk	rs17177078	16	24810681	T	C	-0.025	0.004	9.5e-10	6.8e-05	36.6
Drinking Status	DrnkWk	rs153106	16	28526897	C	T	-0.014	0.002	3.6e-12	9.1e-05	48.9
Drinking Status	DrnkWk	rs3814877	16	30042677	T	G	-0.011	0.002	1.1e-08	6.1e-05	32.6
Drinking Status	DrnkWk	rs79616692	16	72338507	C	G	0.019	0.003	2.4e-09	6.8e-05	36.4
Drinking Status	DrnkWk	rs11860773	16	73912503	C	T	-0.016	0.003	8.4e-10	7.1e-05	38.1
Drinking Status	DrnkWk	rs13332432	16	85721809	G	C	0.014	0.002	5.9e-11	7.9e-05	42.0
Drinking Status	DrnkWk	rs34121753	17	7733833	G	A	0.011	0.002	1.4e-08	5.9e-05	31.7
Drinking Status	DrnkWk	rs76640332	17	44189858	A	G	-0.022	0.002	1.5e-18	1.4e-04	76.7
Drinking Status	DrnkWk	rs838145	19	49248730	A	G	-0.016	0.002	3.9e-16	1.2e-04	66.1
Drinking Status	DrnkWk	rs6106989	20	25027630	A	G	0.011	0.002	3.8e-08	5.7e-05	30.7
Physical Activity	MVPA	rs6427178	1	169095082	A	G	0.023	0.004	1.7e-08	6.4e-05	31.2
Physical Activity	MVPA	rs1160545	2	100832269	T	C	0.025	0.004	1.7e-09	7.6e-05	36.9
Physical Activity	MVPA	rs336620	3	18628793	G	C	-0.024	0.004	4.0e-08	6.3e-05	30.5
Physical Activity	MVPA	rs7613360	3	49916710	T	C	-0.025	0.004	2.8e-09	7.1e-05	34.6
Physical Activity	MVPA	rs1691471	3	85011013	T	C	0.038	0.004	1.7e-19	1.7e-04	81.4
Physical Activity	MVPA	rs2290338	3	85362101	C	T	0.026	0.004	7.3e-11	7.3e-05	43.4
Physical Activity	MVPA	rs2668196	3	165502709	T	A	0.023	0.004	2.1e-08	5.4e-05	32.2
Physical Activity	MVPA	rs4865512	5	50661601	G	A	-0.024	0.004	7.7e-09	6.7e-05	32.7

Phenotype	Trait	SNP	Chr	Position (GRCh37/hg19)	EA	NEA	BETA	SE	P-value	R2	F
Physical Activity	MVPA	rs4546329	5	60589739	T	C	-0.018	0.003	1.4e-08	5.3e-05	32.0
Physical Activity	MVPA	rs370935521	6	26770791	T	C	0.043	0.007	8.2e-09	7.5e-05	33.5
Physical Activity	MVPA	rs13201721	6	141799534	C	T	-0.025	0.004	1.8e-10	7.8e-05	40.6
Physical Activity	MVPA	rs12357890	10	99762693	G	A	-0.022	0.004	4.8e-08	6.2e-05	30.1
Physical Activity	MVPA	rs1424751	11	57479732	C	G	-0.024	0.004	2.5e-08	6.9e-05	31.0
Physical Activity	MVPA	rs1625595	11	66078129	T	C	-0.021	0.003	1.9e-11	7.5e-05	44.3
Physical Activity	MVPA	rs568546	11	107321156	T	C	0.024	0.004	5.9e-09	6.9e-05	33.4
Physical Activity	MVPA	rs385301	17	19806828	T	C	-0.028	0.005	1.6e-09	7.9e-05	36.5
Physical Activity	MVPA	rs9903845	17	50291181	A	C	-0.020	0.003	6.1e-09	5.7e-05	34.6

$R^2 = \frac{2\beta^2 EAF(1-EAF)}{2\beta^2 EAF(1-EAF)+(se)^2(2N)EAF(1-EAF)}$, where EAF is the effect allele frequency, β is the effect estimate of the SNP on the corresponding phenotype, and N is the number of individuals in the GWAS analysis (Shim 2015, PLoS One;10(4): e0120758).

$F = \frac{R^2(N-2)}{1-R^2}$, where R2 is the variance of the corresponding phenotype explained by the specific SNP (as explained above) and N the number of individuals in the GWAS analysis (Palmer 2012, Stat Methods Med Res;21(3):223-42).

Supplementary Table 2 Genetic variants used as instruments to proxy SBP-lowering drug targets

Drug Target	SNP	Chr	Position (GRCh37)	EA	NEA	BETA	SE	P-value	R2	F
**ACE inhibitors**										
ACEs	rs4291	17	61554194	A	T	-0.284	0.031	8.6e-20	1.1e-04	82.8
**Beta Blockers**										
ADRB1	rs1801253	10	115805056	C	G	0.463	0.034	2.8e-41	2.4e-04	180.8
ADRB1	rs460718	10	115721364	A	G	-0.276	0.032	1.4e-17	9.9e-05	72.8
ADRB1	rs11196549	10	115707298	A	G	0.688	0.078	1.6e-18	1.0e-04	77.1
ADRB1	rs11196597	10	115788094	A	G	0.286	0.046	4.2e-10	5.3e-05	38.9
ADRB1	rs4359161	10	115826508	A	G	-0.266	0.039	9.5e-12	6.3e-05	46.3
ADRB1	rs17875473	10	115800294	T	C	0.328	0.055	2.7e-09	4.8e-05	35.4
**Calcium channel blockers**										
CACNA1D	rs3821843	3	53558012	A	G	0.337	0.034	6.6e-24	1.4e-04	101.4
CACNA1D	rs114987861	3	53605712	A	G	0.529	0.096	3.4e-08	4.1e-05	30.5
CACNA1D	rs113210396	3	53612327	T	G	-0.434	0.077	1.8e-08	4.3e-05	31.7
CACNA1D	rs7340705	3	53734443	T	C	-0.242	0.032	4.9e-14	7.7e-05	56.7
CACNB2	rs1888693	10	18440444	A	G	0.386	0.032	4.7e-34	2.0e-04	148.1
CACNB2	rs7076319	10	18459450	A	G	-0.321	0.034	5.1e-21	1.2e-04	88.6
CACNB2	rs61278674	10	18481737	A	G	-0.330	0.054	1.0e-09	5.1e-05	37.3
CACNB2	rs1779209	10	18514561	T	C	0.274	0.034	4.2e-16	9.1e-05	66.3
CACNB2	rs10828399	10	18553968	A	G	-0.195	0.030	1.1e-10	5.6e-05	41.6
CACNB2	rs10828452	10	18592450	A	T	0.305	0.039	4.2e-15	8.4e-05	61.6
CACNB2	rs10828542	10	18627285	A	G	0.182	0.031	5.2e-09	4.6e-05	34.1
CACNB2	rs12780039	10	18678987	C	G	0.285	0.047	1.3e-09	5.0e-05	36.8
CACNB2	rs79253631	10	18694223	A	G	-0.777	0.139	2.3e-08	4.3e-05	31.2
CACNB2	rs112133583	10	18695681	T	C	-0.555	0.097	1.2e-08	4.4e-05	32.5
CACNB2	rs7909027	10	18695892	T	C	-0.331	0.032	2.0e-25	1.5e-04	108.5
CACNB2	rs11014170	10	18710991	A	G	-0.670	0.115	5.6e-09	4.6e-05	34.0
CACNB2	rs7923191	10	18727901	A	G	-0.369	0.038	1.1e-22	1.3e-04	96.3
CACNB2	rs12258967	10	18727959	C	G	0.633	0.034	1.1e-78	4.8e-04	352.3
CACNB2	rs72786098	10	18729855	A	G	-0.503	0.088	1.2e-08	4.4e-05	32.5
CACNB2	rs1998822	10	18755664	A	G	-0.196	0.034	1.1e-08	4.5e-05	32.6
CACNB2	rs2488136	10	18334521	A	G	0.226	0.033	1.2e-11	6.2e-05	45.8
CACNB3	rs150857355	12	49209340	C	G	0.941	0.112	5.2e-17	9.6e-05	70.3
CACNA1C	rs2239046	12	2434419	A	G	0.208	0.032	9.6e-11	5.6e-05	41.8
CACNA1C	rs714277	12	2514270	T	C	0.199	0.033	2.4e-09	4.8e-05	35.6

$R^2 = \frac{2\beta^2 EAF(1-EAF)}{2\beta^2 EAF(1-EAF)+(se)^2(2N)EAF(1-EAF)}$, where EAF is the effect allele frequency, β is the effect estimate of the SNP on the SBP, and N the number of individuals in the GWAS analysis (Shim 2015, PLoS One;10(4):e0120758).

$F = \frac{R^2(N-2)}{1-R^2}$, where R2 is the variance of SBP explained by the specific SNP (as explained above) and N the number of individuals in the GWAS analysis (Palmer 2012, Stat Methods Med Res;21(3):223-42).

Supplementary Table 3 Genetic variants used as instruments to proxy lipid-lowering drug targets

Drug Target	SNP	Chr	Position (GRCh37)	EA	NEA	BETA	SE	P-value	R2	F
**LDL-lowering Targets**										
HMGCR	rs150415361	5	74536192	A	G	0.078	0.011	1.0e-13	4.7e-05	55.3
HMGCR	rs1308220	5	74537301	A	G	-0.049	0.005	1.1e-23	8.5e-05	100.6
HMGCR	rs142156764	5	74537548	A	T	0.037	0.006	2.5e-10	3.3e-05	40.0
HMGCR	rs16878747	5	74560349	C	T	0.043	0.007	3.4e-10	3.2e-05	39.4
HMGCR	rs10051965	5	74560487	T	C	0.048	0.001	1.0e-200	8.6e-04	1055.7
HMGCR	rs11741997	5	74560542	G	A	-0.039	0.006	8.0e-12	4.3e-05	46.8
HMGCR	rs72633961	5	74564190	G	A	0.068	0.002	1.0e-200	1.3e-03	1631.6
HMGCR	rs113988305	5	74569590	A	G	0.042	0.008	1.9e-08	2.6e-05	31.6
HMGCR	rs72768351	5	74582374	G	A	-0.040	0.006	4.8e-13	4.3e-05	52.3
HMGCR	rs138636245	5	74589365	T	C	0.069	0.009	6.1e-15	5.0e-05	60.9
HMGCR	rs75144964	5	74597689	G	C	-0.040	0.006	1.7e-11	3.8e-05	45.3
HMGCR	rs35122945	5	74610293	C	A	-0.037	0.003	1.0e-35	1.3e-04	155.6
HMGCR	rs6895057	5	74624234	A	G	0.047	0.002	1.7e-185	6.8e-04	843.1
HMGCR	rs75240579	5	74624484	T	C	-0.040	0.004	5.5e-27	9.5e-05	115.7
HMGCR	rs17238568	5	74631071	T	G	0.043	0.003	4.5e-52	1.9e-04	230.5
HMGCR	rs17244939	5	74631096	C	A	-0.043	0.006	2.8e-12	4.0e-05	48.8
HMGCR	rs536947347	5	74633133	A	G	-0.049	0.008	8.1e-09	2.7e-05	33.3
HMGCR	rs115169875	5	74633264	A	G	-0.034	0.005	2.7e-13	4.4e-05	53.4
HMGCR	rs551145289	5	74670594	T	C	0.098	0.013	3.7e-14	5.3e-05	57.3
HMGCR	rs78587954	5	74677170	G	C	0.053	0.003	1.1e-77	2.8e-04	348.0
HMGCR	rs144083983	5	74683306	T	C	-0.042	0.003	2.4e-44	1.6e-04	195.1
HMGCR	rs180978629	5	74688599	T	C	0.066	0.012	2.3e-08	2.6e-05	31.2
HMGCR	rs181668591	5	74689045	T	C	0.060	0.007	6.5e-17	5.7e-05	69.8
HMGCR	rs114253542	5	74692810	C	T	0.076	0.009	1.9e-18	6.3e-05	76.8
HMGCR	rs145888305	5	74697794	G	A	0.066	0.011	4.3e-09	2.9e-05	34.5
HMGCR	rs114872747	5	74698750	C	T	0.088	0.013	6.1e-12	4.1e-05	47.3
HMGCR	rs114200677	5	74701484	C	T	0.063	0.010	9.2e-10	3.1e-05	37.5
HMGCR	rs180755046	5	74703989	A	C	-0.042	0.006	1.4e-11	3.8e-05	45.7
HMGCR	rs75944831	5	74720611	G	A	-0.048	0.008	9.0e-10	3.1e-05	37.5
HMGCR	rs151000110	5	74725216	A	G	0.067	0.003	1.6e-103	3.8e-04	466.5
HMGCR	rs116153450	5	74729433	A	C	-0.036	0.004	3.5e-23	8.0e-05	98.4
HMGCR	rs10473973	5	74730306	C	T	0.046	0.007	6.0e-10	3.1e-05	38.3
HMGCR	rs140048772	5	74746415	T	A	0.086	0.013	1.1e-11	3.9e-05	46.2
HMGCR	rs62366598	5	74749147	T	C	-0.031	0.003	2.8e-24	8.7e-05	103.3
HMGCR	rs200823803	5	74753738	C	T	0.055	0.006	3.9e-20	7.9e-05	84.5
PCSK9	rs11588211	1	55406482	T	C	-0.317	0.015	2.6e-101	4.7e-04	456.4
PCSK9	rs7549203	1	55413361	C	A	0.010	0.001	2.2e-12	4.0e-05	49.3
PCSK9	rs12043403	1	55431933	C	T	-0.024	0.002	3.0e-23	8.1e-05	98.6
PCSK9	rs57428252	1	55434931	T	G	-0.022	0.002	3.2e-20	6.9e-05	84.9
PCSK9	rs11206497	1	55436790	C	T	0.010	0.001	2.3e-12	4.0e-05	49.2
PCSK9	rs149341284	1	55442993	T	C	-0.070	0.012	3.2e-09	3.2e-05	35.0
PCSK9	rs1549564	1	55447270	G	A	0.014	0.002	3.6e-17	5.8e-05	71.0
PCSK9	rs561554246	1	55453057	G	A	-0.429	0.052	1.7e-16	1.7e-04	67.9
PCSK9	rs12123143	1	55453856	C	T	0.013	0.002	5.6e-09	4.5e-05	34.0
PCSK9	rs145391209	1	55462672	A	G	-0.044	0.006	7.3e-13	4.2e-05	51.4
PCSK9	rs183549858	1	55465741	A	C	0.058	0.010	3.1e-09	2.9e-05	35.1
PCSK9	rs55637835	1	55466303	T	C	-0.017	0.002	2.5e-14	4.7e-05	58.1
PCSK9	rs17111474	1	55479967	T	C	0.011	0.002	4.5e-12	4.1e-05	47.9
PCSK9	rs2479396	1	55482091	A	G	0.011	0.002	8.2e-13	4.5e-05	51.2

Drug Target	SNP	Chr	Position (GRCh37)	EA	NEA	BETA	SE	P-value	R2	F
PCSK9	rs146480899	1	55483477	G	A	-0.036	0.005	1.9e-12	4.0e-05	49.6
PCSK9	rs34232196	1	55489542	T	C	-0.066	0.002	1.0e-200	1.3e-03	1630.2
PCSK9	rs374459115	1	55489968	A	G	0.068	0.007	5.1e-24	9.5e-05	102.1
PCSK9	rs111835689	1	55490282	T	C	0.040	0.002	4.3e-81	3.0e-04	363.6
PCSK9	rs80085738	1	55495299	A	G	-0.041	0.007	2.6e-09	2.9e-05	35.4
PCSK9	rs185586206	1	55495731	T	C	-0.119	0.018	1.1e-10	3.7e-05	41.7
PCSK9	rs2479417	1	55495744	C	T	-0.014	0.001	2.2e-22	7.9e-05	94.7
PCSK9	rs2495494	1	55497136	G	C	-0.023	0.003	2.2e-13	4.5e-05	53.8
PCSK9	rs149502401	1	55500970	T	C	-0.145	0.024	1.9e-09	3.6e-05	36.1
PCSK9	rs72660548	1	55500978	G	C	0.070	0.005	6.2e-46	1.6e-04	202.4
PCSK9	rs142236283	1	55502146	A	G	-0.053	0.005	7.4e-31	1.1e-04	133.4
PCSK9	rs150893545	1	55502286	T	A	0.069	0.009	3.1e-15	5.1e-05	62.2
PCSK9	rs28385700	1	55504401	T	C	0.051	0.009	5.7e-09	2.8e-05	33.9
PCSK9	rs2479409	1	55504650	A	G	-0.043	0.001	5.3e-189	7.2e-04	859.2
PCSK9	rs75777418	1	55510892	G	A	0.156	0.013	4.0e-34	1.2e-04	148.3
PCSK9	rs28385707	1	55512018	T	A	0.075	0.009	7.4e-18	6.1e-05	74.1
PCSK9	rs10888897	1	55513061	C	T	0.036	0.001	1.2e-132	5.1e-04	600.3
PCSK9	rs41294821	1	55513183	T	C	-0.043	0.005	1.7e-17	5.9e-05	72.5
PCSK9	rs7546522	1	55516713	T	C	-0.022	0.002	1.9e-28	1.0e-04	122.3
PCSK9	rs148195424	1	55518374	T	C	-0.555	0.043	8.5e-38	2.4e-04	165.1
PCSK9	rs2479413	1	55518682	T	C	-0.050	0.002	1.0e-200	8.9e-04	1046.8
PCSK9	rs41294825	1	55519068	T	A	-0.041	0.003	7.2e-34	1.2e-04	147.2
PCSK9	rs45566638	1	55519863	T	C	0.059	0.007	2.3e-16	5.5e-05	67.4
PCSK9	rs45508296	1	55520547	G	A	-0.065	0.005	2.0e-33	1.2e-04	145.1
PCSK9	rs150119739	1	55520938	A	G	0.070	0.004	6.8e-82	3.0e-04	367.3
PCSK9	rs11587071	1	55522674	T	C	-0.045	0.002	1.2e-122	4.5e-04	554.5
PCSK9	rs41297885	1	55526840	G	C	-0.041	0.004	7.8e-27	9.3e-05	115.0
PCSK9	rs539259897	1	55529944	C	G	-0.701	0.101	4.0e-12	1.2e-04	48.1
PCSK9	rs28385715	1	55532142	G	T	0.046	0.005	1.3e-20	7.2e-05	86.6
PCSK9	rs192276219	1	55532511	G	A	-0.041	0.007	3.2e-08	2.5e-05	30.6
PCSK9	rs145372536	1	55532710	A	G	0.138	0.015	9.5e-21	7.3e-05	87.3
PCSK9	rs77011887	1	55534271	T	C	0.046	0.006	8.2e-16	5.3e-05	64.8
PCSK9	rs10493176	1	55538552	G	T	-0.071	0.003	1.1e-175	6.5e-04	798.1
PCSK9	rs138856381	1	55542694	T	C	-0.063	0.008	3.4e-15	5.1e-05	62.0
PCSK9	rs190720101	1	55544698	C	T	-0.125	0.016	2.5e-15	5.2e-05	62.6
PCSK9	rs138981210	1	55547334	T	C	0.102	0.008	8.9e-38	1.4e-04	165.0
PCSK9	rs371621780	1	55548992	A	G	-0.554	0.029	4.6e-82	4.6e-04	368.1
PCSK9	rs142318450	1	55561224	G	A	-0.085	0.011	1.1e-15	5.3e-05	64.3
PCSK9	rs181149007	1	55562928	T	G	-0.592	0.082	3.8e-13	1.3e-04	52.7
PCSK9	rs115460230	1	55566699	T	C	-0.052	0.009	9.5e-10	3.1e-05	37.4
PCSK9	rs111488799	1	55578169	G	A	0.100	0.008	5.4e-40	1.4e-04	175.2
PCSK9	rs115465289	1	55580914	A	G	-0.041	0.004	6.2e-22	7.5e-05	92.6
PCSK9	rs139471498	1	55584033	C	T	0.085	0.012	1.8e-13	4.5e-05	54.3
PCSK9	rs191448950	1	55584844	A	G	-0.414	0.008	1.0e-200	2.1e-03	2611.3
PCSK9	rs575331971	1	55596509	G	A	-0.358	0.055	1.1e-10	1.0e-04	41.6
PCSK9	rs574121437	1	55600247	A	G	0.129	0.022	3.4e-09	8.6e-05	35.0
PCSK9	rs553629879	1	55602330	C	T	0.136	0.015	2.9e-19	6.8e-05	80.5
PCSK9	rs368908265	1	55608515	C	T	-0.105	0.015	9.6e-13	4.7e-05	50.9
PCSK9	rs572849884	1	55617670	C	T	-0.447	0.047	6.9e-22	1.7e-04	92.4
PCSK9	rs564951008	1	55624448	C	T	-0.431	0.061	1.1e-12	1.3e-04	50.7
PCSK9	rs149489863	1	55626128	A	G	-0.542	0.032	6.5e-64	2.9e-04	284.8

Drug Target	SNP	Chr	Position (GRCh37)	EA	NEA	BETA	SE	P-value	R2	F
PCSK9	rs17111688	1	55626676	A	G	0.087	0.004	1.5e-122	4.5e-04	553.9
PCSK9	rs147863227	1	55626872	T	C	0.092	0.009	2.3e-22	7.7e-05	94.6
LDLR	rs527288769	19	11101019	G	A	-0.142	0.026	2.8e-08	3.7e-05	30.8
LDLR	rs548746023	19	11104727	C	T	-0.406	0.047	2.6e-18	1.9e-04	76.1
LDLR	rs537285153	19	11105963	A	G	-0.317	0.055	1.1e-08	8.2e-05	32.7
LDLR	rs73013153	19	11113281	T	C	-0.193	0.008	8.9e-135	5.0e-04	610.1
LDLR	rs12983316	19	11114352	G	A	0.043	0.002	6.6e-107	4.0e-04	482.1
LDLR	rs536693949	19	11116576	G	A	-0.302	0.037	6.5e-16	9.6e-05	65.3
LDLR	rs563243095	19	11117282	A	G	0.156	0.022	4.7e-13	6.6e-05	52.3
LDLR	rs116945003	19	11119233	A	G	0.083	0.008	7.7e-23	8.0e-05	96.8
LDLR	rs148724334	19	11122019	C	G	0.043	0.006	2.4e-13	4.4e-05	53.6
LDLR	rs567474873	19	11122469	A	G	-0.111	0.020	2.6e-08	4.0e-05	31.0
LDLR	rs73013159	19	11122710	T	G	-0.129	0.003	1.0e-200	1.5e-03	1816.9
LDLR	rs557024333	19	11125383	T	C	-0.250	0.044	1.6e-08	4.8e-05	31.9
LDLR	rs566795598	19	11125636	A	G	-0.456	0.069	3.3e-11	6.6e-05	44.0
LDLR	rs78527782	19	11126142	A	G	0.063	0.007	2.5e-18	6.2e-05	76.2
LDLR	rs118115977	19	11128365	T	C	0.039	0.006	2.1e-10	3.3e-05	40.4
LDLR	rs192566697	19	11131652	T	G	-0.209	0.033	2.2e-10	5.7e-05	40.2
LDLR	rs564402674	19	11132291	A	G	0.115	0.013	2.0e-19	7.0e-05	81.2
LDLR	rs557281937	19	11134700	T	C	0.082	0.010	4.2e-16	5.5e-05	66.1
LDLR	rs56101423	19	11135078	T	C	0.137	0.021	1.2e-10	5.2e-05	41.5
LDLR	rs186362541	19	11136304	C	T	-0.077	0.009	1.8e-17	5.9e-05	72.3
LDLR	rs552839111	19	11139483	G	A	-0.442	0.026	6.5e-64	7.1e-04	284.7
LDLR	rs545861602	19	11140494	T	C	-0.353	0.064	4.5e-08	5.1e-05	29.9
LDLR	rs144108046	19	11140707	T	C	-0.506	0.091	2.5e-08	5.1e-05	31.0
LDLR	rs4804562	19	11147615	A	G	-0.042	0.001	1.5e-174	6.5e-04	792.8
LDLR	rs562146065	19	11148274	A	G	-0.181	0.031	8.6e-09	4.6e-05	33.1
LDLR	rs140808844	19	11151059	G	A	-0.033	0.004	1.1e-21	7.5e-05	91.5
LDLR	rs192107518	19	11153695	A	G	0.259	0.043	1.2e-09	4.3e-05	37.0
LDLR	rs189076912	19	11155283	A	C	0.133	0.020	1.2e-11	6.3e-05	46.0
LDLR	rs568637145	19	11156755	T	C	-0.235	0.010	7.3e-118	4.4e-04	532.5
LDLR	rs11668554	19	11157919	G	A	0.010	0.002	1.2e-09	3.3e-05	37.0
LDLR	rs55948246	19	11159889	C	T	-0.068	0.002	1.0e-200	1.4e-03	1752.5
LDLR	rs544502327	19	11162544	T	C	-0.481	0.062	8.5e-15	1.5e-04	60.2
LDLR	rs78549246	19	11162983	A	T	0.090	0.013	1.3e-12	5.3e-05	50.3
LDLR	rs1529728	19	11163897	C	T	-0.047	0.001	1.0e-200	9.3e-04	1141.5
LDLR	rs138914633	19	11165610	T	G	-0.315	0.021	7.0e-51	2.0e-04	225.0
LDLR	rs534203832	19	11166422	A	C	0.160	0.027	4.9e-09	4.5e-05	34.2
LDLR	rs139591415	19	11167316	A	G	-0.363	0.039	2.9e-20	1.4e-04	85.1
LDLR	rs185646448	19	11167424	A	G	-0.108	0.017	6.6e-10	4.1e-05	38.1
LDLR	rs36049922	19	11169305	C	T	0.044	0.004	2.5e-28	1.0e-04	121.8
LDLR	rs199690115	19	11169419	A	G	-0.408	0.047	4.9e-18	1.9e-04	74.9
LDLR	rs545160301	19	11171813	A	G	-0.219	0.033	5.5e-11	6.2e-05	43.0
LDLR	rs78218585	19	11174496	G	C	-0.070	0.005	1.9e-51	1.9e-04	227.7
LDLR	rs11671812	19	11176337	T	C	0.054	0.003	6.7e-57	2.1e-04	252.7
LDLR	rs371899114	19	11177496	A	G	0.085	0.012	8.8e-13	4.3e-05	51.1
LDLR	rs113194249	19	11180058	C	T	0.090	0.010	3.5e-20	7.0e-05	84.7
LDLR	rs139232176	19	11181171	A	G	-0.222	0.012	2.0e-75	2.8e-04	337.6
LDLR	rs143539721	19	11181404	A	G	0.079	0.009	1.5e-19	7.7e-05	81.8
LDLR	rs10423733	19	11185919	C	T	-0.131	0.002	1.0e-200	4.0e-03	4956.9
LDLR	rs74940106	19	11189641	G	A	-0.489	0.086	1.5e-08	4.6e-05	32.0

Drug Target	SNP	Chr	Position (GRCh37)	EA	NEA	BETA	SE	P-value	R2	F
LDLR	rs142274292	19	11191624	T	C	0.106	0.008	8.5e-41	1.5e-04	178.9
LDLR	rs550066154	19	11192327	C	T	0.185	0.034	4.6e-08	4.6e-05	29.9
LDLR	rs112159161	19	11192603	T	C	-0.200	0.006	1.0e-200	9.3e-04	1144.8
LDLR	rs17249120	19	11199496	A	G	-0.163	0.013	8.2e-39	1.4e-04	169.8
LDLR	rs17242353	19	11200806	T	C	0.109	0.004	2.5e-144	5.3e-04	654.0
LDLR	rs17242367	19	11204627	T	C	0.028	0.003	1.8e-22	7.8e-05	95.1
LDLR	rs537829044	19	11204927	C	T	-0.469	0.080	4.2e-09	5.4e-05	34.5
LDLR	rs17248748	19	11206040	T	C	-0.075	0.006	4.9e-41	1.5e-04	179.9
LDLR	rs572948750	19	11207345	A	G	-0.362	0.043	4.1e-17	9.6e-05	70.7
LDLR	rs73015030	19	11207516	A	G	-0.133	0.004	1.0e-200	8.9e-04	1096.5
LDLR	rs17242402	19	11208397	T	C	0.114	0.010	2.8e-28	1.1e-04	121.6
LDLR	rs560115066	19	11209990	G	T	0.073	0.005	2.3e-58	2.8e-04	259.3
LDLR	rs10423288	19	11215846	C	T	0.076	0.006	3.7e-35	1.3e-04	153.1
LDLR	rs537582537	19	11217096	T	C	-0.246	0.026	4.4e-22	9.5e-05	93.3
LDLR	rs180760728	19	11219281	C	T	-0.345	0.008	1.0e-200	1.5e-03	1737.0
LDLR	rs568336016	19	11219624	T	C	0.231	0.036	1.2e-10	1.1e-04	41.5
LDLR	rs570504499	19	11220517	A	C	-0.349	0.058	2.0e-09	9.2e-05	35.9
LDLR	rs562512066	19	11220961	G	T	-0.220	0.032	3.2e-12	1.1e-04	48.6
LDLR	rs55792959	19	11221289	T	C	-0.098	0.011	3.5e-20	7.0e-05	84.7
LDLR	rs192390193	19	11221487	A	G	0.114	0.013	5.1e-18	6.2e-05	74.8
LDLR	rs180876220	19	11223083	G	C	0.107	0.010	1.5e-28	1.0e-04	122.8
LDLR	rs6413505	19	11224157	T	C	0.080	0.008	4.3e-24	8.4e-05	102.5
LDLR	rs2738447	19	11227480	C	A	0.056	0.001	1.0e-200	1.3e-03	1570.0
LDLR	rs17248882	19	11227525	A	G	0.117	0.020	7.5e-09	3.3e-05	33.4
LDLR	rs527357541	19	11227855	A	C	0.216	0.034	2.1e-10	1.0e-04	40.3
LDLR	rs180773846	19	11228794	A	G	0.072	0.007	4.1e-25	8.8e-05	107.2
LDLR	rs191531000	19	11229495	T	C	0.100	0.014	2.6e-12	4.6e-05	49.0
LDLR	rs62129100	19	11230672	G	A	0.021	0.003	2.5e-11	5.1e-05	44.5
LDLR	rs527424716	19	11231402	A	G	-0.179	0.028	1.6e-10	4.1e-05	40.8
LDLR	rs147223423	19	11232261	T	A	0.100	0.007	4.9e-45	1.6e-04	198.3
LDLR	rs45508991	19	11233886	T	C	0.105	0.007	1.5e-47	1.7e-04	209.8
LDLR	rs181994650	19	11235610	T	C	0.074	0.011	4.8e-11	5.1e-05	43.2
LDLR	rs116778995	19	11238996	T	C	-0.258	0.017	2.3e-52	2.0e-04	231.8
LDLR	rs542873596	19	11239005	C	A	-0.449	0.029	5.1e-55	6.1e-04	243.9
LDLR	rs137992968	19	11239696	T	C	-0.093	0.005	1.8e-76	3.2e-04	342.4
LDLR	rs17249001	19	11241428	A	G	0.071	0.003	8.5e-133	4.9e-04	601.1
LDLR	rs56270417	19	11242011	A	G	-0.258	0.030	1.1e-17	7.9e-05	73.4
LDLR	rs3826810	19	11242133	A	G	0.038	0.003	5.3e-28	9.8e-05	120.3
LDLR	rs17249029	19	11242330	A	G	0.075	0.013	1.0e-08	2.8e-05	32.8
LDLR	rs72658875	19	11243219	T	C	-0.306	0.053	6.1e-09	5.3e-05	33.8
LDLR	rs143587805	19	11243542	T	A	0.063	0.007	4.0e-21	7.3e-05	88.9
LDLR	rs147540853	19	11248104	A	G	-0.091	0.004	4.6e-100	3.7e-04	450.7
LDLR	rs74862316	19	11248782	T	C	-0.208	0.018	4.4e-31	1.6e-04	134.4
LDLR	rs531359724	19	11250445	A	G	-0.323	0.045	9.7e-13	6.3e-05	50.9
LDLR	rs143026258	19	11251500	C	T	-0.375	0.047	1.5e-15	1.6e-04	63.6
LDLR	rs185482271	19	11253692	T	C	-0.128	0.013	5.1e-24	9.5e-05	102.1
LDLR	rs4804147	19	11256285	G	A	0.031	0.001	5.5e-104	3.8e-04	468.7
LDLR	rs184755907	19	11256675	T	C	-0.147	0.025	2.7e-09	4.2e-05	35.4
LDLR	rs111622889	19	11259273	T	C	0.045	0.006	1.8e-15	5.8e-05	63.3
LDLR	rs553556107	19	11259571	T	C	0.085	0.009	2.3e-20	7.0e-05	85.5
LDLR	rs139774348	19	11261445	A	G	0.074	0.009	2.8e-15	5.2e-05	62.4

Drug Target	SNP	Chr	Position (GRCh37)	EA	NEA	BETA	SE	P-value	R2	F
LDLR	rs551676793	19	11264052	A	G	-0.251	0.045	2.3e-08	7.8e-05	31.2
LDLR	rs11671872	19	11265020	A	G	-0.064	0.001	1.0e-200	1.6e-03	1951.0
LDLR	rs566140164	19	11265225	G	C	-0.535	0.026	5.1e-97	6.3e-04	436.6
LDLR	rs11879968	19	11269423	A	T	0.052	0.008	2.0e-10	3.9e-05	40.5
LDLR	rs182348385	19	11272998	G	C	0.091	0.016	7.3e-09	3.1e-05	33.4
LDLR	rs189143685	19	11274168	T	C	0.049	0.007	7.3e-13	4.2e-05	51.4
LDLR	rs148079581	19	11274668	C	A	0.092	0.009	5.0e-27	9.5e-05	115.9
LDLR	rs143979605	19	11277575	T	A	0.132	0.022	1.6e-09	3.2e-05	36.4
LDLR	rs111731690	19	11277922	T	G	0.024	0.003	1.0e-12	4.1e-05	50.8
LDLR	rs570974999	19	11280249	T	C	0.090	0.014	1.4e-10	3.5e-05	41.2
LDLR	rs117339792	19	11282298	A	G	-0.085	0.006	3.1e-43	1.6e-04	190.1
LDLR	rs4804149	19	11284028	C	T	0.024	0.002	9.6e-50	1.9e-04	219.8
LDLR	rs562896829	19	11284299	G	A	-0.246	0.042	5.7e-09	3.8e-05	33.9
LDLR	rs379309	19	11284302	T	C	-0.025	0.001	1.3e-69	2.6e-04	310.9
LDLR	rs139198665	19	11284392	T	C	0.061	0.005	1.8e-37	1.3e-04	163.6
LDLR	rs193296317	19	11284560	T	C	-0.149	0.022	6.4e-12	4.5e-05	47.2
LDLR	rs61734799	19	11287412	T	C	0.064	0.010	9.7e-11	3.5e-05	41.9
LDLR	rs151111259	19	11294226	A	G	-0.199	0.036	4.2e-08	4.4e-05	30.1
LDLR	rs550611465	19	11295752	A	C	-0.436	0.061	1.2e-12	1.2e-04	50.4
LDLR	rs76288880	19	11302336	G	A	0.026	0.002	1.3e-25	9.0e-05	109.4
LDLR	rs7249669	19	11302904	A	G	0.022	0.004	3.1e-09	2.9e-05	35.1
LDLR	rs185317898	19	11304297	A	G	-0.395	0.065	1.2e-09	9.1e-05	36.9
LDLR	rs754528	19	11305245	C	T	0.019	0.002	7.6e-14	4.5e-05	55.9
LDLR	rs12980863	19	11309871	T	C	0.022	0.001	4.5e-55	2.0e-04	244.3
LDLR	rs146220810	19	11309953	T	C	-0.096	0.015	7.9e-11	4.0e-05	42.3
LDLR	rs200393834	19	11311386	A	G	0.108	0.016	4.9e-12	4.2e-05	47.7
LDLR	rs143466522	19	11318472	A	G	-0.053	0.009	1.2e-09	3.0e-05	37.0
LDLR	rs200307398	19	11323999	T	C	-0.176	0.011	1.3e-54	2.5e-04	242.1
LDLR	rs34243815	19	11324965	T	C	-0.023	0.003	2.6e-15	5.2e-05	62.5
LDLR	rs564214041	19	11330355	G	A	0.083	0.015	1.5e-08	3.4e-05	32.1
LDLR	rs139347491	19	11334329	T	A	-0.137	0.011	6.6e-36	1.3e-04	156.5
LDLR	rs8101801	19	11335477	A	C	-0.048	0.003	1.3e-43	1.6e-04	191.8
LDLR	rs73506650	19	11340498	C	T	0.022	0.003	6.6e-16	5.3e-05	65.2
NPC1L1	rs141530948	7	44511663	A	G	-0.056	0.009	3.0e-09	2.9e-05	35.2
NPC1L1	rs7798185	7	44570717	A	C	0.034	0.002	4.4e-78	2.9e-04	349.8
NPC1L1	rs77826622	7	44572346	C	T	0.031	0.005	2.3e-10	3.3e-05	40.2
NPC1L1	rs139659653	7	44578747	A	G	-0.200	0.027	9.6e-14	7.4e-05	55.4
NPC1L1	rs2073547	7	44582331	G	A	0.043	0.002	5.4e-91	5.0e-04	409.0
NPC1L1	rs77517259	7	44586770	T	C	0.050	0.007	5.1e-12	3.9e-05	47.6
NPC1L1	rs217385	7	44602187	T	G	-0.029	0.001	1.0e-93	3.4e-04	421.6
NPC1L1	rs118187778	7	44612914	G	A	0.025	0.004	7.5e-09	2.7e-05	33.4
NPC1L1	rs191955602	7	44629606	T	C	0.057	0.010	2.1e-09	2.9e-05	35.9
NPC1L1	rs79836087	7	44635997	A	G	-0.022	0.003	3.7e-10	3.2e-05	39.2
NPC1L1	rs79854399	7	44640315	T	C	-0.039	0.005	8.1e-14	4.6e-05	55.8
**TG-lowering Targets**										
LPL	rs1826418	8	19664490	G	A	-0.020	0.001	4.9e-47	1.7e-04	207.4
LPL	rs148903487	8	19667872	G	A	-0.130	0.013	7.3e-24	8.5e-05	101.4
LPL	rs3899015	8	19668707	T	C	-0.052	0.002	4.6e-146	5.3e-04	662.0
LPL	rs3758060	8	19673973	C	T	0.138	0.007	2.3e-77	2.8e-04	346.5
LPL	rs539118782	8	19676524	A	G	-0.168	0.025	1.8e-11	4.2e-05	45.2
LPL	rs2278615	8	19678314	G	A	-0.028	0.003	3.0e-21	7.2e-05	89.5

Drug Target	SNP	Chr	Position (GRCh37)	EA	NEA	BETA	SE	P-value	R2	F
LPL	rs201621947	8	19680410	A	G	0.159	0.012	8.0e-41	1.5e-04	179.0
LPL	rs149859209	8	19685593	A	G	0.183	0.016	1.3e-30	1.6e-04	132.3
LPL	rs76769796	8	19686315	G	A	-0.035	0.002	2.0e-65	2.3e-04	291.7
LPL	rs183878912	8	19688373	T	C	0.046	0.007	1.7e-12	4.0e-05	49.9
LPL	rs559477267	8	19692824	A	G	-0.254	0.042	2.1e-09	8.8e-05	35.9
LPL	rs146521119	8	19695072	T	C	0.043	0.006	3.0e-11	3.6e-05	44.2
LPL	rs77312736	8	19714837	A	G	0.014	0.002	1.5e-09	2.9e-05	36.6
LPL	rs56373382	8	19717573	A	T	0.020	0.003	9.7e-13	4.1e-05	50.9
LPL	rs11998681	8	19722560	T	G	0.043	0.002	2.0e-146	5.3e-04	663.6
LPL	rs6988864	8	19726742	G	A	0.011	0.002	3.5e-13	4.2e-05	52.9
LPL	rs545516154	8	19729354	C	T	0.268	0.034	3.6e-15	1.6e-04	61.9
LPL	rs144907154	8	19734622	T	C	-0.185	0.019	1.2e-21	1.1e-04	91.4
LPL	rs73607783	8	19742204	A	T	-0.068	0.002	1.0e-200	1.2e-03	1548.0
LPL	rs564004042	8	19742935	G	T	0.260	0.037	3.2e-12	1.2e-04	48.5
LPL	rs578043912	8	19745762	C	T	-0.220	0.033	3.9e-11	5.8e-05	43.7
LPL	rs190299239	8	19748090	G	A	-0.311	0.049	2.9e-10	4.9e-05	39.7
LPL	rs11204081	8	19748122	G	A	0.023	0.001	1.1e-62	2.2e-04	279.2
LPL	rs138215942	8	19748180	A	C	-0.272	0.046	2.2e-09	3.6e-05	35.7
LPL	rs1441774	8	19751755	T	A	-0.023	0.004	1.2e-10	3.4e-05	41.5
LPL	rs80041799	8	19753467	T	C	-0.076	0.007	1.1e-31	1.1e-04	137.2
LPL	rs145280633	8	19753600	C	T	0.043	0.007	9.5e-11	3.4e-05	41.9
LPL	rs182725791	8	19761630	C	T	-0.099	0.015	1.0e-11	3.8e-05	46.3
LPL	rs150799387	8	19766707	A	G	0.173	0.006	4.2e-198	7.2e-04	901.0
LPL	rs142084074	8	19768150	A	G	-0.153	0.006	7.4e-142	5.2e-04	642.7
LPL	rs3988301	8	19770379	G	T	-0.087	0.002	1.0e-200	1.6e-03	1963.2
LPL	rs373010541	8	19771395	T	C	0.109	0.015	4.0e-13	4.5e-05	52.7
LPL	rs559883181	8	19773545	A	G	-0.358	0.047	4.5e-14	7.8e-05	56.9
LPL	rs55737222	8	19775271	C	T	0.288	0.037	9.7e-15	6.8e-05	60.0
LPL	rs182184672	8	19776058	A	G	0.072	0.012	8.0e-09	2.7e-05	33.3
LPL	rs187013686	8	19784052	T	C	-0.175	0.009	2.5e-85	3.1e-04	383.1
LPL	rs190118115	8	19793581	T	C	0.226	0.005	1.0e-200	1.5e-03	1713.0
LPL	rs371696649	8	19804097	A	G	-0.102	0.008	1.6e-34	1.2e-04	150.2
LPL	rs73667470	8	19807341	C	A	0.156	0.005	1.9e-187	6.8e-04	852.1
LPL	rs536076610	8	19807477	C	T	0.378	0.045	3.8e-17	1.7e-04	70.9
LPL	rs78326602	8	19809220	A	G	0.076	0.010	2.6e-13	4.4e-05	53.5
LPL	rs531593252	8	19809855	C	T	-0.172	0.015	2.5e-29	1.0e-04	126.4
LPL	rs144578061	8	19813878	C	A	-0.072	0.008	2.6e-19	6.5e-05	80.7
LPL	rs277	8	19814403	C	T	0.029	0.002	2.2e-60	2.1e-04	268.6
LPL	rs286	8	19815256	T	A	-0.171	0.003	1.0e-200	3.7e-03	4655.0
LPL	rs301	8	19816934	C	T	-0.112	0.002	1.0e-200	3.9e-03	4826.6
LPL	rs308	8	19817476	G	T	-0.155	0.005	1.0e-200	8.7e-04	1084.5
LPL	rs554540115	8	19818084	C	T	0.228	0.039	4.6e-09	5.2e-05	34.4
LPL	rs323	8	19819221	C	A	0.047	0.008	7.7e-09	2.7e-05	33.3
LPL	rs3289	8	19823192	C	T	0.155	0.004	1.0e-200	1.0e-03	1276.9
LPL	rs113831503	8	19825055	T	C	-0.036	0.004	1.8e-20	6.9e-05	86.0
LPL	rs80085105	8	19825297	C	T	0.086	0.008	4.5e-27	9.4e-05	116.1
LPL	rs564058250	8	19825501	C	A	-0.250	0.044	9.3e-09	8.0e-05	33.0
LPL	rs2197089	8	19826373	A	G	-0.057	0.001	1.0e-200	1.4e-03	1743.5
LPL	rs548372218	8	19827638	A	G	-0.240	0.039	6.2e-10	5.4e-05	38.3
LPL	rs554780319	8	19828313	T	C	-0.066	0.010	2.7e-11	4.6e-05	44.4
LPL	rs548617076	8	19828740	C	T	0.134	0.015	2.1e-18	6.6e-05	76.6

Drug Target	SNP	Chr	Position (GRCh37)	EA	NEA	BETA	SE	P-value	R2	F
LPL	rs531537695	8	19829850	C	A	0.307	0.025	2.6e-35	3.8e-04	153.7
LPL	rs182091484	8	19832700	A	G	0.113	0.012	1.5e-22	7.8e-05	95.5
LPL	rs148681769	8	19835621	A	T	0.057	0.006	1.7e-19	6.7e-05	81.6
LPL	rs117303935	8	19837269	T	C	-0.037	0.003	2.1e-32	1.1e-04	140.5
LPL	rs186147937	8	19841219	C	T	-0.142	0.025	1.2e-08	2.8e-05	32.5
LPL	rs578074162	8	19842473	G	C	0.253	0.023	2.5e-29	3.2e-04	126.3
LPL	rs147011441	8	19843748	A	G	0.045	0.005	5.8e-18	6.0e-05	74.6
LPL	rs566546099	8	19844470	G	A	-0.069	0.011	1.1e-09	3.0e-05	37.2
LPL	rs142288236	8	19845612	T	C	0.073	0.006	6.2e-37	1.3e-04	161.2
LPL	rs181174350	8	19849398	C	T	0.055	0.007	5.8e-15	4.9e-05	61.0
LPL	rs11992311	8	19853146	T	C	0.070	0.003	5.9e-105	3.8e-04	473.2
LPL	rs117579447	8	19856331	G	A	0.088	0.010	5.5e-18	6.1e-05	74.7
LPL	rs76623698	8	19856983	C	A	-0.060	0.009	2.9e-11	3.6e-05	44.2
LPL	rs537482174	8	19864081	G	T	-0.202	0.031	1.1e-10	1.0e-04	41.6
LPL	rs561588511	8	19864490	T	A	0.408	0.066	6.0e-10	6.0e-05	38.3
LPL	rs188443653	8	19867720	C	A	-0.175	0.008	3.9e-102	3.7e-04	460.2
LPL	rs144824668	8	19869789	A	G	0.190	0.016	1.1e-32	1.7e-04	141.7
LPL	rs7005359	8	19870693	G	A	-0.079	0.002	1.0e-200	1.5e-03	1908.0
LPL	rs559942143	8	19873411	A	G	-0.261	0.021	2.0e-34	1.7e-04	149.7
LPL	rs10105418	8	19875365	A	G	0.147	0.004	1.0e-200	9.1e-04	1135.9
LPL	rs186172167	8	19876610	T	C	-0.227	0.015	5.8e-53	1.9e-04	234.6
LPL	rs181241315	8	19876663	T	C	-0.344	0.025	2.3e-44	2.1e-04	195.2
LPL	rs184386132	8	19879054	A	G	-0.197	0.010	6.1e-94	3.4e-04	422.6
LPL	rs186879193	8	19881560	C	A	0.204	0.036	2.1e-08	8.1e-05	31.4
LPL	rs555409362	8	19886369	T	C	0.255	0.020	4.9e-37	1.4e-04	161.6
LPL	rs141570430	8	19888343	T	C	-0.133	0.005	2.0e-130	4.7e-04	590.2
LPL	rs187544997	8	19890641	G	C	0.063	0.006	3.1e-24	8.3e-05	103.1
LPL	rs4557718	8	19890654	C	T	0.051	0.002	7.6e-135	5.0e-04	610.5
LPL	rs184748159	8	19890770	G	A	-0.336	0.044	1.5e-14	6.4e-05	59.1
LPL	rs555211558	8	19893301	A	G	0.098	0.015	2.6e-11	5.2e-05	44.5
LPL	rs192184585	8	19893903	T	C	0.043	0.007	6.2e-09	2.7e-05	33.8
LPL	rs530229037	8	19896484	C	T	0.101	0.013	5.5e-15	5.0e-05	61.1
LPL	rs73612169	8	19901072	T	A	-0.069	0.006	5.0e-28	9.6e-05	120.4
LPL	rs138773389	8	19902884	C	T	0.038	0.007	3.3e-08	2.5e-05	30.5
LPL	rs138966397	8	19910304	A	T	-0.080	0.007	3.7e-32	1.1e-04	139.4
LPL	rs189702906	8	19910774	T	C	-0.218	0.010	1.1e-103	3.8e-04	467.3
LPL	rs184674402	8	19912601	A	G	-0.203	0.027	2.3e-14	6.2e-05	58.2
LPL	rs189463635	8	19912670	A	G	-0.683	0.110	6.1e-10	6.1e-05	38.3
LPL	rs118045108	8	19913833	T	C	0.047	0.005	1.6e-21	7.3e-05	90.8
LPL	rs577319938	8	19914917	A	T	0.122	0.017	9.6e-13	6.4e-05	50.9
LPL	rs78376313	8	19917471	C	T	0.041	0.004	1.5e-26	9.1e-05	113.7
LPL	rs35014088	8	19918553	A	G	-0.039	0.002	8.2e-56	2.0e-04	247.7
APOC3	rs61905081	11	116600564	A	G	0.207	0.003	1.0e-200	4.8e-03	6072.2
APOC3	rs150549687	11	116601477	T	A	-0.078	0.009	4.5e-17	5.7e-05	70.5
APOC3	rs144453220	11	116602214	A	G	0.246	0.034	7.3e-13	5.6e-05	51.5
APOC3	rs567222600	11	116604964	A	G	0.515	0.055	9.2e-21	1.1e-04	87.3
APOC3	rs11216122	11	116609540	T	G	-0.036	0.003	1.6e-29	1.0e-04	127.3
APOC3	rs577035642	11	116610693	T	C	-0.072	0.011	1.2e-11	4.6e-05	46.0
APOC3	rs377491137	11	116619390	T	A	0.252	0.006	1.0e-200	1.3e-03	1643.7
APOC3	rs74496689	11	116621623	A	G	-0.065	0.005	1.2e-33	1.2e-04	146.1
APOC3	rs79112951	11	116621972	T	C	-0.024	0.004	1.8e-11	3.6e-05	45.2

Drug Target	SNP	Chr	Position (GRCh37)	EA	NEA	BETA	SE	P-value	R2	F
APOC3	rs74368849	11	116622299	A	G	0.286	0.043	3.7e-11	4.9e-05	43.8
APOC3	rs192269528	11	116622683	A	G	0.255	0.037	2.8e-12	6.8e-05	48.8
APOC3	rs184421176	11	116626792	T	C	0.153	0.012	1.4e-36	1.4e-04	159.5
APOC3	rs193108398	11	116627177	A	G	0.243	0.008	1.0e-200	7.6e-04	941.2
APOC3	rs17519079	11	116629153	A	G	-0.029	0.003	1.2e-25	8.7e-05	109.5
APOC3	rs150233369	11	116629555	A	G	-0.049	0.005	1.1e-25	8.8e-05	109.7
APOC3	rs187117860	11	116631789	A	T	-0.075	0.010	1.2e-13	4.6e-05	55.0
APOC3	rs117106280	11	116634837	C	G	-0.039	0.007	6.3e-09	2.7e-05	33.7
APOC3	rs79610135	11	116635951	C	T	-0.036	0.004	1.8e-18	6.2e-05	76.9
APOC3	rs547886066	11	116637571	T	G	-0.053	0.007	2.4e-13	4.3e-05	53.6
APOC3	rs1268353	11	116639692	T	C	-0.067	0.001	1.0e-200	1.8e-03	2193.0
APOC3	rs545950926	11	116641187	G	C	0.123	0.017	2.2e-12	4.3e-05	49.3
APOC3	rs148028577	11	116642755	A	G	-0.033	0.005	2.0e-11	3.6e-05	45.0
APOC3	rs543000923	11	116645079	A	T	0.409	0.062	3.2e-11	1.1e-04	44.0
APOC3	rs74773964	11	116649759	C	T	-0.044	0.005	1.5e-17	5.8e-05	72.7
APOC3	rs139636218	11	116650295	C	T	0.441	0.009	1.0e-200	1.9e-03	2352.0
APOC3	rs560442486	11	116652153	G	T	0.274	0.020	1.3e-44	2.1e-04	196.4
APOC3	rs185082164	11	116654375	A	G	0.303	0.020	9.0e-53	2.7e-04	233.7
APOC3	rs562775669	11	116654745	T	C	0.338	0.038	1.2e-18	8.3e-05	77.7
APOC3	rs531357809	11	116658494	G	A	0.376	0.065	8.7e-09	4.5e-05	33.1
APOC3	rs138452085	11	116660001	A	G	0.338	0.048	2.0e-12	5.4e-05	49.5
APOC3	rs142958146	11	116660008	G	A	0.280	0.008	1.0e-200	9.4e-04	1164.6
APOC3	rs143292359	11	116661001	A	G	0.329	0.034	5.2e-22	8.9e-05	93.0
APOC3	rs3135507	11	116661488	T	C	0.081	0.004	4.4e-80	2.9e-04	359.0
APOC3	rs9804646	11	116665079	T	C	-0.060	0.002	1.1e-131	4.8e-04	595.9
APOC3	rs45555231	11	116665120	C	G	-0.040	0.006	3.4e-12	3.9e-05	48.5
APOC3	rs17520254	11	116665553	G	C	-0.076	0.003	1.9e-151	5.5e-04	686.8
APOC3	rs543178884	11	116665745	C	T	0.295	0.031	2.5e-21	8.4e-05	89.9
APOC3	rs182936182	11	116668970	A	G	0.238	0.019	9.8e-35	1.6e-04	151.1
APOC3	rs116886525	11	116671391	T	C	0.127	0.008	1.6e-57	2.1e-04	255.5
APOC3	rs140697471	11	116671875	T	G	0.222	0.024	3.5e-21	8.9e-05	89.2
APOC3	rs142172482	11	116672392	C	T	0.198	0.010	1.9e-83	3.0e-04	374.4
APOC3	rs61905132	11	116673464	T	C	0.228	0.005	1.0e-200	2.1e-03	2562.8
APOC3	rs189930576	11	116674440	T	C	0.330	0.039	5.7e-17	1.8e-04	70.0
APOC3	rs117246585	11	116677356	T	C	-0.051	0.007	1.5e-14	4.7e-05	59.1
APOC3	rs549729213	11	116677825	T	C	0.464	0.083	2.0e-08	4.4e-05	31.5
APOC3	rs61905133	11	116678191	G	A	-0.027	0.004	2.1e-13	4.3e-05	54.0
APOC3	rs75542613	11	116679155	A	G	-0.059	0.003	1.4e-102	3.7e-04	462.3
APOC3	rs7927820	11	116682156	A	G	-0.093	0.002	1.0e-200	1.7e-03	2154.3
APOC3	rs75318540	11	116683862	T	C	-0.044	0.005	9.2e-21	7.0e-05	87.3
APOC3	rs139069259	11	116684082	A	G	0.276	0.027	6.8e-24	9.7e-05	101.6
APOC3	rs534039500	11	116691054	T	C	0.243	0.017	1.0e-44	2.1e-04	196.8
APOC3	rs571827425	11	116691410	G	A	-0.094	0.013	2.5e-13	4.4e-05	53.5
APOC3	rs147448028	11	116693647	A	G	0.241	0.015	1.6e-58	2.2e-04	260.1
APOC3	rs546829656	11	116698320	C	T	-0.128	0.020	1.1e-10	4.4e-05	41.7
APOC3	rs2727788	11	116698963	A	C	-0.046	0.002	2.1e-188	6.9e-04	856.5
APOC3	rs10790164	11	116699389	G	A	-0.231	0.005	1.0e-200	2.7e-03	2255.3
APOC3	rs12721078	11	116699395	A	C	-0.072	0.004	1.7e-80	2.9e-04	360.9
APOC3	rs148295370	11	116700487	T	C	-0.109	0.020	2.9e-08	3.8e-05	30.8
APOC3	rs138326449	11	116701354	A	G	-1.125	0.018	1.0e-200	3.5e-03	4105.4
APOC3	rs12721098	11	116702229	T	C	-0.052	0.008	4.4e-11	3.5e-05	43.4

Drug Target	SNP	Chr	Position (GRCh37)	EA	NEA	BETA	SE	P-value	R2	F
APOC3	rs4225	11	116703671	T	G	-0.064	0.001	1.0e-200	1.6e-03	1932.3
APOC3	rs12721031	11	116704505	T	C	-0.049	0.005	2.5e-27	9.4e-05	117.3
APOC3	rs555819148	11	116705405	A	G	0.277	0.037	4.6e-14	6.2e-05	56.9
APOC3	rs140301504	11	116708750	C	T	-0.077	0.010	9.3e-16	5.3e-05	64.6
APOC3	rs183737298	11	116712329	A	G	0.190	0.018	2.0e-25	1.3e-04	108.6
APOC3	rs551151056	11	116716353	G	A	0.205	0.026	9.0e-16	7.3e-05	64.6
APOC3	rs143186980	11	116719611	G	A	0.053	0.007	4.3e-13	4.2e-05	52.5
APOC3	rs548104945	11	116723154	G	A	0.365	0.034	4.2e-26	1.3e-04	111.7
APOC3	rs117221732	11	116725659	A	G	0.208	0.010	5.6e-104	3.8e-04	468.7
APOC3	rs181873174	11	116726640	A	G	-0.090	0.010	1.5e-18	7.9e-05	77.3
APOC3	rs78427244	11	116727524	A	G	0.274	0.023	8.9e-33	1.4e-04	142.2
APOC3	rs183167801	11	116728169	T	C	0.235	0.020	2.3e-31	1.1e-04	135.7
APOC3	rs144882902	11	116728517	A	G	0.369	0.068	4.9e-08	5.5e-05	29.8
APOC3	rs555934108	11	116729796	A	C	0.305	0.033	3.6e-20	1.2e-04	84.6
APOC3	rs187537072	11	116731872	C	T	-0.114	0.018	5.1e-10	4.5e-05	38.6
APOC3	rs535916709	11	116740548	A	T	0.269	0.042	1.4e-10	6.2e-05	41.2
APOC3	rs142839861	11	116741712	T	C	0.305	0.034	2.5e-19	2.1e-04	80.8
APOC3	rs191449055	11	116747547	T	C	0.953	0.137	3.2e-12	7.8e-05	48.6
APOC3	rs535885869	11	116751878	C	A	-0.125	0.020	1.6e-10	4.4e-05	40.9
APOC3	rs541798108	11	116754415	T	C	1.534	0.252	1.2e-09	5.9e-05	37.0
APOC3	rs139733873	11	116755560	G	A	0.228	0.025	1.3e-20	9.9e-05	86.6
APOC3	rs556278194	11	116757869	G	C	0.280	0.039	8.8e-13	5.5e-05	51.1
APOC3	rs1473177	11	116759332	C	T	0.068	0.002	1.0e-200	7.7e-04	966.4
APOC3	rs571732812	11	116763627	C	A	0.372	0.065	8.3e-09	8.0e-05	33.2
APOC3	rs182599193	11	116767481	T	C	0.264	0.035	9.2e-14	8.1e-05	55.5
APOC3	rs595137	11	116771356	T	C	-0.093	0.005	4.4e-88	3.2e-04	395.7
APOC3	rs34144542	11	116774447	G	A	-0.039	0.003	1.3e-45	1.6e-04	200.9
APOC3	rs188316032	11	116778084	A	C	-0.145	0.027	5.0e-08	2.7e-05	29.7
APOC3	rs139523029	11	116785447	C	T	0.205	0.016	1.2e-37	1.4e-04	164.4
APOC3	rs187830550	11	116786300	T	C	0.284	0.039	4.6e-13	7.1e-05	52.3
APOC3	rs115968150	11	116796713	A	G	1.920	0.303	2.4e-10	1.7e-04	40.1
APOC3	rs112701434	11	116797442	C	T	-0.039	0.004	6.2e-28	9.6e-05	120.0
APOC3	rs11216190	11	116797684	C	T	-0.039	0.002	3.2e-60	2.1e-04	267.9
APOC3	rs117459305	11	116800073	C	A	0.929	0.065	8.6e-46	2.7e-04	201.7
**HDL-raising Targets**										
CETP	rs562392336	16	56895768	A	G	0.149	0.019	6.3e-15	1.6e-04	60.8
CETP	rs146619351	16	56896702	G	A	0.110	0.006	2.8e-77	2.8e-04	346.1
CETP	rs140123059	16	56896892	A	G	0.143	0.020	3.5e-13	6.0e-05	52.9
CETP	rs200271933	16	56897620	A	G	-0.265	0.028	7.4e-21	8.8e-05	87.8
CETP	rs184151024	16	56898043	T	C	0.148	0.015	2.1e-22	1.3e-04	94.8
CETP	rs117987946	16	56899183	T	C	0.142	0.021	1.4e-11	4.0e-05	45.7
CETP	rs534822153	16	56899880	C	T	-0.066	0.008	4.4e-15	5.1e-05	61.5
CETP	rs12918664	16	56900519	T	C	-0.012	0.002	1.8e-14	4.8e-05	58.7
CETP	rs13306673	16	56900931	T	C	-0.081	0.002	1.0e-200	8.6e-04	1072.5
CETP	rs141938931	16	56901445	G	A	-0.316	0.013	1.6e-136	5.1e-04	618.1
CETP	rs199852748	16	56903757	T	C	-0.168	0.016	9.6e-27	9.5e-05	114.6
CETP	rs12597428	16	56905458	G	T	0.043	0.002	1.7e-143	5.3e-04	650.2
CETP	rs142679083	16	56906568	T	C	0.112	0.015	4.3e-14	5.6e-05	57.0
CETP	rs138286718	16	56907225	C	T	-0.122	0.015	1.1e-15	5.3e-05	64.2
CETP	rs11640954	16	56908884	A	G	0.060	0.002	1.0e-200	9.4e-04	1157.1
CETP	rs62035948	16	56910293	A	G	-0.028	0.005	1.9e-08	2.6e-05	31.6

Drug Target	SNP	Chr	Position (GRCh37)	EA	NEA	BETA	SE	P-value	R2	F
CETP	rs116383747	16	56910637	A	G	0.166	0.018	2.2e-21	8.2e-05	90.2
CETP	rs79844654	16	56910906	A	C	-0.043	0.006	5.6e-14	4.6e-05	56.5
CETP	rs139238742	16	56910936	A	G	-0.094	0.010	6.0e-22	7.6e-05	92.7
CETP	rs79968526	16	56911069	C	T	-0.064	0.005	3.8e-40	1.4e-04	175.9
CETP	rs191444600	16	56911264	C	T	0.090	0.003	1.0e-159	5.9e-04	724.7
CETP	rs577613896	16	56911313	T	A	0.153	0.013	3.5e-32	1.7e-04	139.4
CETP	rs13306688	16	56911856	T	G	0.061	0.010	9.4e-10	3.1e-05	37.5
CETP	rs569288154	16	56912293	A	G	-0.178	0.022	3.4e-16	1.8e-04	66.6
CETP	rs551114142	16	56912801	T	C	0.192	0.024	8.4e-16	7.6e-05	64.8
CETP	rs72786758	16	56912904	A	G	-0.110	0.014	2.3e-14	4.9e-05	58.2
CETP	rs566327653	16	56913973	T	C	0.304	0.033	1.9e-20	1.7e-04	85.9
CETP	rs181454960	16	56914011	T	C	0.144	0.008	9.8e-66	2.4e-04	293.2
CETP	rs148931404	16	56914455	A	G	0.131	0.005	1.0e-137	5.1e-04	623.7
CETP	rs111273057	16	56915269	T	C	0.120	0.008	2.2e-49	1.8e-04	218.2
CETP	rs141724276	16	56915407	T	C	0.060	0.005	5.3e-34	1.2e-04	147.8
CETP	rs180875104	16	56917168	A	G	-0.085	0.009	3.5e-22	7.7e-05	93.8
CETP	rs12929471	16	56920483	A	G	0.303	0.016	2.5e-76	3.6e-04	341.8
CETP	rs537342415	16	56921471	A	G	-0.203	0.034	1.9e-09	3.8e-05	36.1
CETP	rs144580397	16	56922293	A	G	-0.117	0.019	1.7e-09	3.4e-05	36.3
CETP	rs190351409	16	56922750	A	G	-0.296	0.019	9.6e-56	3.0e-04	247.3
CETP	rs140770781	16	56925500	T	C	-0.141	0.011	1.9e-37	1.4e-04	163.6
CETP	rs181112562	16	56927284	G	A	0.102	0.017	3.0e-09	3.9e-05	35.2
CETP	rs75346962	16	56929843	C	T	-0.050	0.005	1.4e-22	7.7e-05	95.6
CETP	rs141890420	16	56931816	A	G	-0.614	0.097	2.1e-10	5.8e-05	40.4
CETP	rs3794655	16	56937329	T	C	-0.062	0.002	2.8e-200	7.3e-04	911.0
CETP	rs187721921	16	56937864	T	C	0.305	0.047	1.2e-10	4.7e-05	41.4
CETP	rs28651544	16	56939533	G	A	0.162	0.027	2.9e-09	3.7e-05	35.3
CETP	rs1138429	16	56942921	T	A	-0.091	0.002	1.0e-200	1.3e-03	1576.0
CETP	rs188168278	16	56943760	A	G	0.233	0.028	8.0e-17	1.0e-04	69.4
CETP	rs59515242	16	56945049	A	C	-0.080	0.002	1.0e-200	1.6e-03	2035.9
CETP	rs4567697	16	56945619	G	T	-0.028	0.001	1.0e-86	3.2e-04	389.5
CETP	rs138696161	16	56945988	A	G	-0.116	0.006	2.2e-76	2.8e-04	342.0
CETP	rs544208203	16	56946026	A	G	0.189	0.025	4.3e-14	7.8e-05	57.0
CETP	rs527804384	16	56946148	T	G	0.189	0.016	5.1e-34	2.3e-04	147.8
CETP	rs117738782	16	56946502	C	T	-0.139	0.008	4.2e-70	2.5e-04	313.2
CETP	rs549191237	16	56946653	A	G	0.393	0.066	3.0e-09	9.3e-05	35.2
CETP	rs139928813	16	56947066	C	A	0.216	0.015	2.9e-46	1.7e-04	203.9
CETP	rs188179537	16	56947092	A	G	0.174	0.009	5.0e-78	2.8e-04	349.6
CETP	rs139828223	16	56947893	G	C	-0.209	0.015	2.7e-46	1.7e-04	204.0
CETP	rs184498979	16	56952262	A	G	-0.182	0.013	4.6e-45	1.6e-04	198.4
CETP	rs189063017	16	56952461	T	C	0.189	0.026	2.8e-13	5.9e-05	53.3
CETP	rs148973971	16	56952721	A	G	0.150	0.015	3.3e-23	8.4e-05	98.5
CETP	rs530299667	16	56954020	A	C	-0.557	0.087	1.7e-10	1.1e-04	40.7
CETP	rs191128095	16	56954865	A	C	0.181	0.020	1.5e-19	1.1e-04	81.7
CETP	rs571668051	16	56955279	T	G	0.171	0.016	2.8e-26	1.0e-04	112.5
CETP	rs565370927	16	56955728	T	C	-0.390	0.071	4.0e-08	4.1e-05	30.1
CETP	rs554574125	16	56956104	C	G	-0.133	0.011	2.3e-33	1.2e-04	144.9
CETP	rs576324892	16	56956123	G	A	-0.164	0.015	5.7e-28	1.2e-04	120.2
CETP	rs12149263	16	56957517	T	C	0.053	0.005	3.9e-30	1.1e-04	130.1
CETP	rs116985036	16	56957844	T	G	-0.178	0.013	7.2e-44	1.6e-04	192.9
CETP	rs75429044	16	56960147	A	G	0.072	0.002	1.0e-200	8.8e-04	1099.0

Drug Target	SNP	Chr	Position (GRCh37)	EA	NEA	BETA	SE	P-value	R2	F
CETP	rs76021218	16	56960805	T	C	0.108	0.009	7.3e-31	1.1e-04	133.4
CETP	rs146571693	16	56962029	C	T	0.220	0.011	2.1e-83	3.1e-04	374.3
CETP	rs563586405	16	56962610	A	G	-0.126	0.013	6.2e-21	7.5e-05	88.1
CETP	rs113286621	16	56969016	A	G	0.168	0.007	6.2e-116	4.3e-04	523.6
CETP	rs568111235	16	56969850	A	T	0.167	0.009	2.2e-84	4.0e-04	378.7
CETP	rs74402821	16	56969977	C	T	-0.118	0.020	5.3e-09	4.1e-05	34.1
CETP	rs72786781	16	56970210	A	T	0.161	0.005	1.0e-200	1.0e-03	1243.9
CETP	rs557755532	16	56971498	A	G	0.100	0.018	3.6e-08	3.0e-05	30.3
CETP	rs557774831	16	56971678	T	C	0.231	0.040	7.5e-09	8.9e-05	33.4
CETP	rs115291165	16	56972197	T	C	-0.236	0.026	3.1e-20	8.9e-05	84.9
CETP	rs550183772	16	56973744	C	T	-0.296	0.015	2.0e-89	3.4e-04	401.8
CETP	rs573155644	16	56976064	A	C	0.192	0.033	3.3e-09	9.3e-05	35.0
CETP	rs184418117	16	56976191	G	A	0.225	0.035	1.8e-10	1.1e-04	40.6
CETP	rs377449652	16	56976743	T	C	-0.199	0.024	1.2e-16	7.8e-05	68.5
CETP	rs572336695	16	56977783	A	G	-0.235	0.021	8.5e-29	1.5e-04	124.0
CETP	rs11864751	16	56978148	T	C	-0.029	0.002	5.1e-78	2.8e-04	349.5
CETP	rs150266133	16	56980193	T	C	-0.115	0.011	1.1e-23	8.3e-05	100.6
CETP	rs146957515	16	56980553	T	C	0.186	0.016	5.3e-33	1.2e-04	143.2
CETP	rs139646866	16	56982584	T	C	-0.199	0.021	2.4e-22	8.0e-05	94.6
CETP	rs247615	16	56984763	G	A	-0.079	0.002	1.0e-200	1.6e-03	2033.4
CETP	rs116448878	16	56984905	G	A	0.411	0.055	1.0e-13	6.6e-05	55.3
CETP	rs72786786	16	56985514	A	G	0.212	0.002	1.0e-200	1.5e-02	18575.5
CETP	rs76277974	16	56986207	A	G	-0.169	0.025	3.7e-11	4.5e-05	43.8
CETP	rs142500745	16	56986531	T	C	-0.272	0.041	3.3e-11	4.8e-05	44.0
CETP	rs575963958	16	56986548	A	G	0.223	0.027	1.7e-16	7.3e-05	68.0
CETP	rs7205692	16	56986914	G	A	-0.084	0.002	1.0e-200	1.5e-03	1899.0
CETP	rs564283356	16	56988061	A	T	0.252	0.011	1.3e-113	4.8e-04	512.9
CETP	rs147464145	16	56988503	T	C	0.180	0.007	4.3e-161	6.0e-04	731.0
CETP	rs139884539	16	56989246	T	C	0.215	0.038	1.1e-08	4.3e-05	32.7
CETP	rs247619	16	56990827	A	G	0.446	0.038	2.1e-32	1.6e-04	140.5
CETP	rs143785930	16	56991452	T	C	0.279	0.046	9.9e-10	6.1e-05	37.3
CETP	rs148808285	16	56991645	T	C	-0.103	0.008	5.3e-34	1.2e-04	147.8
CETP	rs140806876	16	56992473	T	C	-0.409	0.038	3.2e-27	1.2e-04	116.8
CETP	rs12920974	16	56993025	T	G	-0.136	0.002	1.0e-200	6.4e-03	7893.3
CETP	rs548921324	16	56993350	C	G	-0.519	0.091	1.3e-08	6.4e-05	32.4
CETP	rs541129597	16	56993748	A	G	-0.259	0.044	5.2e-09	4.8e-05	34.1
CETP	rs552467014	16	56994779	C	G	0.372	0.047	3.2e-15	8.1e-05	62.2
CETP	rs142464301	16	56996985	T	C	-0.555	0.051	5.5e-28	1.7e-04	120.2
CETP	rs544927894	16	56997218	A	G	-0.293	0.036	1.7e-16	9.0e-05	68.0
CETP	rs114908369	16	56998269	A	C	-1.204	0.159	4.0e-14	9.8e-05	57.2
CETP	rs9929488	16	56998572	C	G	-0.163	0.002	1.0e-200	8.7e-03	10557.7
CETP	rs35619327	16	57000221	T	C	0.128	0.008	1.0e-58	2.1e-04	261.0
CETP	rs573857010	16	57000986	A	G	0.207	0.033	4.3e-10	4.0e-05	39.0
CETP	rs9936680	16	57002152	C	T	0.040	0.003	1.6e-54	1.9e-04	241.8
CETP	rs140268708	16	57002258	T	C	0.126	0.019	2.0e-11	4.4e-05	45.0
CETP	rs12708972	16	57004892	T	C	0.143	0.009	2.1e-52	2.0e-04	232.0
CETP	rs35915925	16	57005363	A	G	0.234	0.019	1.9e-36	1.3e-04	159.0
CETP	rs148654654	16	57006147	T	C	0.185	0.009	2.0e-94	3.5e-04	424.8
CETP	rs187766748	16	57006715	G	A	-0.200	0.017	3.1e-30	1.2e-04	130.6
CETP	rs187468344	16	57007040	T	C	-0.182	0.010	1.3e-74	2.7e-04	333.9
CETP	rs158477	16	57007610	A	G	-0.039	0.001	1.5e-163	6.2e-04	742.3

Drug Target	SNP	Chr	Position (GRCh37)	EA	NEA	BETA	SE	P-value	R2	F
CETP	rs11644475	16	57007652	G	A	0.112	0.004	6.6e-158	5.8e-04	716.4
CETP	rs181381869	16	57008092	A	G	-0.159	0.009	8.7e-78	2.8e-04	348.5
CETP	rs199796310	16	57008377	A	C	-0.382	0.064	2.0e-09	6.6e-05	36.0
CETP	rs79137138	16	57008905	A	G	0.184	0.033	2.4e-08	3.2e-05	31.2
CETP	rs575342274	16	57009135	C	A	-0.056	0.005	2.8e-28	1.3e-04	121.6
CETP	rs545858676	16	57009137	C	A	-0.093	0.005	4.0e-65	3.2e-04	290.3
CETP	rs289716	16	57009376	A	T	-0.106	0.001	1.0e-200	4.1e-03	5032.1
CETP	rs72771489	16	57010117	A	G	-0.139	0.010	2.5e-44	1.6e-04	195.0
CETP	rs56208677	16	57010232	T	C	0.149	0.003	1.0e-200	2.4e-03	2904.3
CETP	rs71387147	16	57010382	G	T	-0.071	0.003	2.4e-155	5.8e-04	704.6
CETP	rs117427818	16	57010486	T	C	-0.294	0.003	1.0e-200	6.6e-03	8155.9
CETP	rs142980129	16	57010519	T	G	0.163	0.008	3.2e-86	3.1e-04	387.2
CETP	rs12720897	16	57010994	G	T	0.027	0.003	1.2e-24	8.5e-05	105.1
CETP	rs538999658	16	57011110	T	C	0.294	0.047	3.6e-10	1.1e-04	39.3
CETP	rs551343692	16	57011265	T	C	0.277	0.040	6.2e-12	1.3e-04	47.3
CETP	rs567823047	16	57011350	G	A	0.569	0.096	2.6e-09	5.2e-05	35.5
CETP	rs534053619	16	57011643	G	T	0.182	0.026	4.0e-12	5.6e-05	48.1
CETP	rs891144	16	57011936	T	C	0.072	0.007	2.3e-25	8.8e-05	108.3
CETP	rs138298866	16	57012038	T	C	0.225	0.024	3.3e-20	1.3e-04	84.8
CETP	rs114203109	16	57012936	A	G	-0.592	0.094	3.6e-10	6.2e-05	39.3
CETP	rs183876127	16	57014035	A	G	-0.067	0.011	8.8e-10	3.2e-05	37.6
CETP	rs12708983	16	57014411	C	T	0.146	0.004	1.0e-200	1.2e-03	1447.9
CETP	rs562924383	16	57014640	T	C	0.164	0.009	8.8e-68	2.5e-04	302.6
CETP	rs12149409	16	57016359	C	G	0.265	0.028	1.3e-20	1.1e-04	86.6
CETP	rs12720917	16	57019392	C	T	0.076	0.002	1.0e-200	1.1e-03	1370.7
CETP	rs184617400	16	57019498	T	G	-0.258	0.024	4.4e-26	1.1e-04	111.6
CETP	rs289745	16	57019532	A	C	0.010	0.002	2.2e-10	3.7e-05	40.2
CETP	rs17369163	16	57020327	G	C	-0.028	0.002	7.6e-40	1.4e-04	174.5
CETP	rs12934552	16	57021433	G	A	-0.051	0.002	2.3e-142	5.2e-04	645.0
CETP	rs151200656	16	57022978	T	C	-0.310	0.028	1.9e-29	1.1e-04	126.9
CETP	rs80327887	16	57023450	T	G	0.044	0.003	4.9e-43	1.5e-04	189.1
CETP	rs289748	16	57025063	G	A	-0.037	0.001	4.5e-153	5.7e-04	694.2
CETP	rs572003930	16	57027754	T	C	0.214	0.022	5.2e-22	1.2e-04	93.0
CETP	rs72773105	16	57028758	G	A	-0.050	0.005	5.2e-21	7.1e-05	88.4
CETP	rs193288094	16	57029577	T	C	-0.192	0.022	2.4e-18	7.5e-05	76.4
CETP	rs145283538	16	57030107	T	G	-0.037	0.006	7.0e-09	2.7e-05	33.5
CETP	rs7187261	16	57031716	T	C	0.073	0.003	2.7e-134	4.9e-04	608.0
CETP	rs76496464	16	57033129	T	C	-0.076	0.012	7.5e-10	3.1e-05	37.9
CETP	rs533720015	16	57038480	G	A	-0.234	0.025	9.7e-21	1.9e-04	87.2
CETP	rs570194437	16	57042128	T	C	-0.404	0.034	1.0e-32	1.6e-04	141.8
CETP	rs149233811	16	57042182	C	T	-0.056	0.009	6.8e-11	3.5e-05	42.6
CETP	rs182457758	16	57043340	T	C	0.105	0.008	1.4e-44	1.6e-04	196.2
CETP	rs148594549	16	57044209	G	A	-0.094	0.012	3.0e-15	5.1e-05	62.3
CETP	rs1167513	16	57045159	C	T	-0.033	0.003	2.9e-30	1.1e-04	130.7
CETP	rs76933585	16	57046069	G	T	-0.043	0.006	4.4e-11	3.5e-05	43.4
CETP	rs191020702	16	57047121	T	C	-0.128	0.015	2.3e-17	8.5e-05	71.8
CETP	rs289703	16	57048118	T	C	-0.038	0.002	2.9e-128	4.8e-04	580.2
CETP	rs75911530	16	57049137	A	G	-0.150	0.004	1.0e-200	1.0e-03	1287.2
CETP	rs200512949	16	57049518	G	A	0.039	0.002	5.7e-71	3.0e-04	317.2
CETP	rs538640172	16	57050024	A	G	0.171	0.024	1.3e-12	1.3e-04	50.3
CETP	rs548254268	16	57051780	G	A	0.140	0.026	4.7e-08	3.0e-05	29.8

Drug Target	SNP	Chr	Position (GRCh37)	EA	NEA	BETA	SE	P-value	R2	F
CETP	rs118117916	16	57053694	G	A	0.098	0.007	2.1e-48	1.7e-04	213.7
CETP	rs185197208	16	57055635	A	G	-0.261	0.008	1.0e-200	9.1e-04	1116.3
CETP	rs562072315	16	57057828	A	G	-0.097	0.016	1.9e-09	4.5e-05	36.1
CETP	rs529751629	16	57058959	A	C	0.201	0.030	2.7e-11	5.7e-05	44.4
CETP	rs56132500	16	57059241	C	G	0.052	0.003	1.9e-75	2.7e-04	337.7
CETP	rs291040	16	57061189	C	T	-0.033	0.001	9.9e-113	4.1e-04	508.9
CETP	rs150403117	16	57063268	T	C	0.143	0.008	3.3e-64	2.4e-04	286.1
CETP	rs535495813	16	57064470	G	A	-0.147	0.021	4.2e-12	5.6e-05	48.0
CETP	rs538493159	16	57064743	A	G	0.195	0.017	6.7e-29	1.6e-04	124.4
CETP	rs187522671	16	57064903	C	T	-0.191	0.027	2.2e-12	6.7e-05	49.3
CETP	rs544795174	16	57066576	A	G	-0.406	0.066	9.4e-10	1.0e-04	37.4
CETP	rs183616684	16	57069414	C	G	-0.174	0.027	8.4e-11	4.3e-05	42.2
CETP	rs116267571	16	57069449	A	G	0.019	0.003	8.3e-11	3.4e-05	42.2
CETP	rs118063294	16	57069661	A	G	0.296	0.044	1.4e-11	7.4e-05	45.7
CETP	rs78325822	16	57069803	T	C	-0.041	0.005	6.9e-19	6.4e-05	78.8
CETP	rs568271305	16	57071647	G	C	-0.139	0.022	2.9e-10	3.9e-05	39.8
CETP	rs4784754	16	57071963	T	C	-0.029	0.002	8.4e-63	2.3e-04	279.7
CETP	rs55771374	16	57073950	T	C	0.083	0.008	5.3e-24	8.4e-05	102.1
CETP	rs145546591	16	57074963	G	C	0.077	0.006	2.5e-43	1.5e-04	190.4
CETP	rs572221855	16	57075207	G	A	0.270	0.024	7.0e-29	2.1e-04	124.3
CETP	rs184175799	16	57075607	T	C	0.171	0.031	4.3e-08	7.9e-05	30.0
CETP	rs201866815	16	57075911	G	T	-0.311	0.040	7.4e-15	1.6e-04	60.5
CETP	rs35885237	16	57076404	A	G	-0.011	0.002	1.0e-11	3.7e-05	46.3
CETP	rs76888401	16	57077179	G	A	-0.053	0.003	1.5e-64	2.3e-04	287.8
CETP	rs573224371	16	57078042	T	C	-0.151	0.022	1.3e-11	5.2e-05	45.8
CETP	rs200883407	16	57080525	T	C	0.196	0.032	5.7e-10	8.6e-05	38.4
CETP	rs72780015	16	57081998	G	C	-0.020	0.002	5.5e-21	7.2e-05	88.4
CETP	rs75719766	16	57082024	T	C	0.046	0.004	2.2e-33	1.2e-04	145.0
CETP	rs11648592	16	57083232	A	G	-0.072	0.005	6.8e-46	1.6e-04	202.2
CETP	rs73546877	16	57089786	T	C	-0.020	0.004	3.1e-08	2.5e-05	30.6
CETP	rs72775169	16	57090582	C	T	0.041	0.003	1.7e-43	1.6e-04	191.3
CETP	rs117587884	16	57091999	A	G	0.046	0.005	2.7e-18	6.2e-05	76.1
CETP	rs1627144	16	57092406	T	C	0.015	0.001	6.2e-27	9.4e-05	115.5
CETP	rs574604244	16	57093152	T	C	-0.097	0.018	4.6e-08	2.6e-05	29.9
CETP	rs146644349	16	57093472	A	G	-0.077	0.012	1.8e-11	3.7e-05	45.2
CETP	rs200695496	16	57095493	C	T	-0.156	0.028	3.0e-08	3.3e-05	30.7
CETP	rs79546662	16	57096098	T	C	-0.054	0.004	1.2e-53	1.9e-04	237.7
CETP	rs191876338	16	57097149	A	G	0.132	0.024	2.6e-08	3.9e-05	31.0
CETP	rs142611813	16	57097274	T	C	-0.055	0.006	1.5e-19	6.6e-05	81.7
CETP	rs190152429	16	57098236	C	T	0.096	0.008	3.1e-30	1.1e-04	130.5
CETP	rs181828544	16	57100168	A	G	0.075	0.012	8.9e-10	3.1e-05	37.5
CETP	rs78360542	16	57100637	A	C	-0.104	0.013	7.0e-17	5.8e-05	69.7
CETP	rs75688907	16	57101903	A	G	0.027	0.002	3.3e-38	1.4e-04	167.0
CETP	rs564055199	16	57106823	T	C	0.130	0.022	2.1e-09	4.7e-05	35.9
CETP	rs7202086	16	57110037	A	G	-0.013	0.002	1.7e-10	3.3e-05	40.8
CETP	rs75003587	16	57113341	G	A	0.014	0.002	4.0e-10	3.2e-05	39.1
CETP	rs35756276	16	57114671	A	G	-0.038	0.003	2.8e-36	1.3e-04	158.2
CETP	rs154045	16	57114924	G	A	-0.018	0.001	1.9e-36	1.3e-04	158.9

$R^2 = \frac{2\beta^2 EAF(1-EAF)}{2\beta^2 EAF(1-EAF)+(se)^2(2N)EAF(1-EAF)}$, where EAF is the effect allele frequency, β is the effect estimate of the SNP on the corresponding phenotype, and N is the number of individuals in the GWAS analysis (Shim 2015, PLoS One;10(4): e0120758).

Supplementary Table 4 Genetic variants used as instruments to proxy glucose-lowering drug targets

Drug Target	SNP	Chr	Position (GRCh37)	EA	NEA	BETA	SE	P-value	R2	F
ABCC8	rs61880293	11	17376498	C	T	0.058	0.009	8.1e-10	3.6e-05	37.7
ABCC8	rs10832783	11	17401134	A	G	0.055	0.009	4.6e-09	3.3e-05	34.4
ABCC8	rs11604345	11	17405442	A	G	-0.035	0.006	1.8e-09	3.5e-05	36.0
ABCC8	rs5215	11	17408630	T	C	-0.068	0.005	3.4e-48	2.0e-04	211.8
GLP1R	rs10305420	6	39016636	T	C	-0.032	0.005	2.7e-11	4.3e-05	45.2
GLP1R	rs41273118	6	39072334	A	T	-0.032	0.005	6.1e-09	3.2e-05	33.9
PPARG	rs11712085	3	12240611	C	T	-0.085	0.013	2.0e-10	3.9e-05	40.6
PPARG	rs7637403	3	12242488	A	G	-0.074	0.007	1.0e-24	1.0e-04	105.6
PPARG	rs59101559	3	12274676	C	T	-0.046	0.008	6.6e-09	3.3e-05	34.1
PPARG	rs9826367	3	12294202	G	A	-0.040	0.005	1.8e-18	7.3e-05	76.4
PPARG	rs2347100	3	12315139	G	C	0.054	0.007	2.9e-14	5.5e-05	57.0
PPARG	rs11709077	3	12336507	A	G	-0.102	0.007	6.8e-47	2.0e-04	204.3
PPARG	rs150535373	3	12410328	A	G	-0.140	0.019	5.4e-13	5.0e-05	52.0
PPARG	rs1152008	3	12487612	G	T	-0.026	0.005	1.7e-08	3.0e-05	31.5
PPARG	rs73029216	3	12501943	A	G	0.039	0.007	2.8e-08	2.9e-05	30.6

$R^2 = \frac{2\beta^2 EAF(1-EAF)}{2\beta^2 EAF(1-EAF)+(se)^2(2N)EAF(1-EAF)}$, where EAF is the effect allele frequency, β is the effect estimate of the SNP on type2 diabetes, and N is the number of individuals in the GWAS analysis (Shim 2015, PLoS One;10(4): e0120758).

$F = \frac{R^2(N-2)}{1-R^2}$, where R2 is the variance of type2 diabetes explained by the specific SNP (as explained above) and N is the number of individuals in the GWAS analysis (Palmer 2012, Stat Methods Med Res;21(3):223-42).

Supplementary Table 5 Genetic variants used as instruments to proxy anti-obesity drug targets

Drug Target	SNP	Chr	Position (GRCh37)	EA	NEA	BETA	SE	P-value	R2	F
GLP1R	rs877446	6	39031039	A	G	-0.010	0.002	4.3e-12	5.1e-05	48.1
GLP1R	rs9357297	6	39009846	T	C	0.024	0.003	2.1e-13	2.4e-04	52.5
GIPR	rs3816046	19	46118127	T	C	0.010	0.002	1.3e-08	3.6e-05	31.9
GIPR	rs11671664	19	46172278	A	G	-0.023	0.002	2.3e-22	9.3e-05	92.6
GIPR	rs1800437	19	46181392	C	G	-0.035	0.002	7.1e-81	3.8e-04	375.9
GIPR	rs78784145	19	46207586	A	G	-0.047	0.007	6.5e-10	1.8e-04	38.6
GIPR	rs7253390	19	46188626	A	C	0.010	0.002	1.1e-09	3.7e-05	36.0
GIPR	rs143430880	19	46180976	A	G	0.223	0.037	1.8e-09	1.7e-04	36.2
GIPR	rs12972158	19	46158473	T	C	0.012	0.002	2.0e-10	4.2e-05	41.9
GIPR	rs73050374	19	46146269	A	G	0.048	0.008	3.5e-10	1.8e-04	39.7

$R^2 = \frac{2\beta^2 EAF(1-EAF)}{2\beta^2 EAF(1-EAF)+(se)^2(2N)EAF(1-EAF)}$, where EAF is the effect allele frequency, β is the effect estimate of the SNP on BMI, and N is the number of individuals in the GWAS analysis (Shim 2015, PLoS One;10(4): e0120758).

$F = \frac{R^2(N-2)}{1-R^2}$, where R2 is the variance of BMI explained by the specific SNP (as explained above) and N is the number of individuals in the GWAS analysis (Palmer 2012, Stat Methods Med Res;21(3):223-42).

Supplementary Table 6 Statistical power estimates for MR analyses

Exposures	R2	LS	MRI-LS	WMH	MD	FA
**Risk factors**						
DBP	6.6%	100%	100%	100%	100%	100%
PP	5.3%	100%	100%	100%	100%	100%
SBP	6.9%	100%	100%	100%	100%	100%
HDL	14.2%	100%	100%	100%	100%	100%
LDL	13.7%	100%	100%	100%	100%	100%
TC	12.7%	100%	100%	100%	100%	100%
TG	10.2%	100%	100%	100%	100%	100%
HbA1c	7.4%	100%	100%	100%	100%	100%
T2D	3.3%	100%	100%	99%	100%	100%
TG:HDL ratio	16.1%	100%	100%	100%	100%	100%
BMI	8.7%	100%	85.2%	100%	100%	100%
WHR	5.7%	100%	100%	100%	100%	100%
CigDay	1.05%	93.1%	100%	100%	100%	49.9%
Lifetime smk	1.3%	100%	100%	100%	100%	100%
SmkInit	0.58%	100%	5.8%	100%	100%	100%
DrnkWk	0.6%	99.8%	100%	100%	83.6%	100%
MVPA	0.13%	100%	99.5%	100%	100%	78.6%
**Drug Targets**						
ACEs	0.01%	8.7%	-	4.2%	47.5%	33.3%
BBs	0.06%	6%	10.7%	21%	99.6%	64.8%
CCBs	0.22%	21.7%	10.8%	60%	100%	100%
APOC3	3.4%	100%	96.7%	99.1%	100%	100%
CETP	8.7%	100%	100%	100%	100%	100%
HMGCR	0.53%	100%	100%	92.8%	100%	100%
LDLR	3%	100%	100%	100%	100%	100%
LPL	3.08%	100%	100%	100%	100%	100%
NPC1L1	0.14%	100%	100%	100%	100%	100%
PCSK9	1.31%	100%	100%	100%	100%	100%
ABCC8	0.03%	100%	99.6%	68.6%	100%	100%
GLP1R	0.008%	100%	100%	100%	100%	100%
PPARG	0.06%	99.9%	30.1%	82.1%	100%	100%
GIPR	0.11%	100%	100%	99.7%	100%	100%
GLP1R	0.03%	100%	100%	100%	100%	100%

Statistical power was also calculated using the online web tool (https://sb452.shinyapps.io/power/).

Abbreviations: R2 indicates the proportion of variance in the exposure explained by genetic instruments; LS, lacunar stroke in GIGASTROKE; MRI-LS, MRI-confirmed lacunar stroke; WMH, white matter hyperintensity; FA, fractional anisotropy; MD, mean diffusivity; DBP, diastolic blood pressure; PP, pulse pressure; SBP, systolic blood pressure; HDL, high density lipoprotein; LDL, low density lipoprotein; TC, total cholesterol; TG, triglycerides; T2D, type 2 diabetes; HbA1c, haemoglobin A1C; TG:HDL, triglycerides to high density lipoprotein ratio; BMI, body mass index; WHR, waist to hip ratio; Lifetime smk, life time smoking index; SmkInit, smoking initiation; DrnkWk, drinks per week; and MVPA, moderate-to-vigorous intensity physical activity during leisure time; ACEs, angiotensin-converting enzyme inhibitors; BBs, beta blockers; CCBs, calcium channel blockers; APOC3, Apolipoprotein C-III inhibitors; CETP, Cholesteryl Ester Transfer Protein inhibitors; HMGCR, HMG-CoA reductase inhibitors; *LDLR*, LDL Receptor; *LPL*, lipoprotein lipase; NPC1L1, NPC1 Like Intracellular Cholesterol Transporter 1 inhibitors; PCSK9, proprotein convertase subtilisin/kexin type 9 inhibitors; ABCC8, ATP binding cassette subfamily C member 8; GLP1R, glucagon like peptide 1 receptor; PPARG, peroxisome proliferator activated receptor gamma; GIPR, gastric inhibitory polypeptide receptor.

Supplementary Table 7 Sensitivity analyses for the Mendelian randomization associations of genetically predicted cardiovascular risk factors with lacunar stroke

Exposure	Outcome	SNPs	Inverse Variance Weighted						MR Egger						Weighted Median		
			OR (95% CI)	P	q	Q statistic			OR (95% CI)	P	q	Egger's Int			OR (95% CI)	P	q
						Q	P	q				Int	P	q			
**Trait: Blood Pressure**																	
DBP	LS	663	1.06 (1.05 - 1.07)	<0.001	<0.001	939.6	<0.001	<0.001	1.07 (1.03 - 1.11)	<0.001	0.002	-0.002	0.571	0.832	1.07 (1.05 - 1.09)	<0.001	<0.001
	MRI_LS	415	1.06 (1.04 - 1.09)	<0.001	<0.001	460.2	0.058	0.070	1.11 (1.04 - 1.18)	0.001	0.013	-0.008	0.149	0.665	1.08 (1.04 - 1.12)	<0.001	<0.001
PP	LS	533	1.04 (1.02 - 1.05)	<0.001	<0.001	736.7	<0.001	<0.001	1.05 (1.01 - 1.08)	0.005	0.032	-0.002	0.521	0.832	1.04 (1.02 - 1.06)	<0.001	<0.001
	MRI_LS	335	1.06 (1.04 - 1.08)	<0.001	<0.001	376.0	0.056	0.069	1.02 (0.97 - 1.08)	0.415	0.619	0.008	0.193	0.665	1.06 (1.03 - 1.10)	0.001	0.004
SBP	LS	710	1.04 (1.03 - 1.04)	<0.001	<0.001	925.6	<0.001	<0.001	1.04 (1.02 - 1.06)	<0.001	<0.001	-0.002	0.454	0.832	1.04 (1.03 - 1.05)	<0.001	<0.001
	MRI_LS	450	1.03 (1.01 - 1.04)	<0.001	0.001	536.4	0.003	0.004	1.06 (1.02 - 1.10)	0.006	0.036	-0.008	0.141	0.665	1.03 (1.01 - 1.05)	0.002	0.009
**Trait: Lipid Metabolism**																	
HDL	LS	762	0.78 (0.71 - 0.85)	<0.001	<0.001	894.0	0.001	0.001	0.85 (0.74 - 0.99)	0.033	0.140	-0.002	0.087	0.665	0.83 (0.68 - 1.02)	0.079	0.191
	MRI_LS	551	0.65 (0.55 - 0.77)	<0.001	<0.001	607.3	0.046	0.057	0.66 (0.52 - 0.85)	0.001	0.011	-0.001	0.782	0.936	0.74 (0.54 - 1.03)	0.073	0.191
LDL	LS	526	1.06 (0.95 - 1.18)	0.325	0.485	711.9	<0.001	<0.001	1.18 (0.99 - 1.40)	0.063	0.198	-0.003	0.108	0.665	0.98 (0.83 - 1.16)	0.820	0.890
	MRI_LS	380	1.32 (1.07 - 1.62)	0.009	0.027	450.6	0.007	0.009	1.54 (1.12 - 2.12)	0.008	0.042	-0.005	0.204	0.665	1.58 (1.12 - 2.22)	0.010	0.046
TC	LS	632	1.04 (0.93 - 1.16)	0.471	0.626	790.5	<0.001	<0.001	1.13 (0.95 - 1.34)	0.177	0.386	-0.002	0.250	0.684	1.05 (0.88 - 1.24)	0.590	0.727
	MRI_LS	455	1.14 (0.94 - 1.38)	0.183	0.339	496.8	0.081	0.094	1.17 (0.86 - 1.58)	0.312	0.568	-0.001	0.824	0.946	1.33 (0.98 - 1.82)	0.071	0.191
TG	LS	645	1.43 (1.27 - 1.60)	<0.001	<0.001	786.6	<0.001	<0.001	1.34 (1.10 - 1.62)	0.003	0.024	0.001	0.394	0.832	1.22 (1.01 - 1.49)	0.044	0.133
	MRI_LS	478	1.50 (1.22 - 1.86)	<0.001	0.001	541.8	0.021	0.028	1.39 (1.00 - 1.94)	0.052	0.187	0.002	0.558	0.832	1.12 (0.78 - 1.60)	0.544	0.719
**Trait: Glucose Metabolism**																	
HbA1c	LS	78	1.14 (0.74 - 1.74)	0.550	0.709	115.3	0.003	0.004	1.47 (0.68 - 3.17)	0.336	0.571	-0.005	0.445	0.832	1.20 (0.66 - 2.18)	0.550	0.719
	MRI_LS	50	2.44 (1.21 - 4.91)	0.013	0.036	42.2	0.742	0.769	1.98 (0.58 - 6.76)	0.280	0.554	0.004	0.689	0.901	1.46 (0.49 - 4.31)	0.496	0.691
T2D	LS	417	1.19 (1.13 - 1.25)	<0.001	<0.001	458.9	0.072	0.085	1.15 (1.02 - 1.30)	0.025	0.116	0.002	0.557	0.832	1.18 (1.07 - 1.30)	0.001	0.008
	MRI_LS	270	1.12 (1.01 - 1.24)	0.040	0.100	271.2	0.451	0.504	1.17 (0.89 - 1.53)	0.256	0.517	-0.002	0.717	0.907	1.18 (1.01 - 1.39)	0.041	0.130
TG:HDL	LS	245	1.23 (1.12 - 1.34)	<0.001	<0.001	318.2	0.001	0.002	1.08 (0.93 - 1.26)	0.294	0.567	0.006	0.040	0.563	1.10 (0.96 - 1.25)	0.175	0.367
	MRI_LS	174	1.28 (1.11 - 1.47)	0.001	0.003	170.9	0.531	0.579	1.12 (0.90 - 1.39)	0.325	0.568	0.008	0.126	0.665	1.24 (0.99 - 1.55)	0.063	0.178
**Trait: Adiposity Markers**																	
BMI	LS	1 229	1.22 (1.09 - 1.35)	<0.001	0.001	1449.3	<0.001	<0.001	1.12 (0.82 - 1.55)	0.476	0.698	0.001	0.610	0.855	1.14 (0.97 - 1.34)	0.109	0.237
	MRI_LS	827	0.98 (0.80 - 1.21)	0.876	0.955	947.8	0.002	0.003	1.01 (0.55 - 1.86)	0.974	0.988	0.000	0.926	0.966	0.92 (0.65 - 1.30)	0.642	0.779
WHR	LS	478	1.29 (1.13 - 1.47)	<0.001	0.001	526.0	0.060	0.072	1.16 (0.84 - 1.61)	0.368	0.602	0.002	0.502	0.832	1.28 (1.03 - 1.58)	0.027	0.098
	MRI_LS	303	1.35 (1.03 - 1.78)	0.029	0.078	358.4	0.014	0.019	0.90 (0.46 - 1.77)	0.764	0.902	0.008	0.196	0.665	1.16 (0.74 - 1.83)	0.512	0.694
**Trait: Smoking Status**																	
CigDay	LS	25	0.96 (0.62 - 1.48)	0.855	0.944	51.1	0.001	0.002	1.04 (0.47 - 2.29)	0.925	0.974	-0.003	0.816	0.946	1.04 (0.69 - 1.56)	0.846	0.899
	MRI_LS	17	0.87 (0.52 - 1.47)	0.613	0.755	4.3	0.998	0.998	0.73 (0.31 - 1.72)	0.484	0.698	0.008	0.614	0.855	0.78 (0.41 - 1.49)	0.452	0.663
Lifetime smk	LS	133	1.75 (1.29 - 2.38)	<0.001	0.001	176.3	0.006	0.008	1.87 (0.55 - 6.36)	0.319	0.568	-0.001	0.915	0.966	1.57 (1.05 - 2.34)	0.029	0.101
	MRI_LS	90	1.69 (1.00 - 2.85)	0.051	0.118	86.7	0.550	0.591	0.97 (0.09 - 10.13)	0.977	0.988	0.008	0.635	0.859	1.58 (0.73 - 3.39)	0.244	0.460
SmkInit	LS	103	1.28 (0.83 - 1.98)	0.269	0.439	128.5	0.039	0.050	10.46 (1.11 - 98.50)	0.043	0.165	-0.024	0.064	0.665	1.23 (0.68 - 2.20)	0.494	0.691
	MRI_LS	68	0.99 (0.45 - 2.16)	0.975	0.994	54.8	0.857	0.878	3.83 (0.07 - 204.51)	0.511	0.716	-0.015	0.498	0.832	0.88 (0.29 - 2.71)	0.827	0.890
**Trait: Drinking Status**																	
DrnkWk	LS	36	1.08 (0.58 - 1.98)	0.813	0.926	52.2	0.031	0.040	0.64 (0.09 - 4.35)	0.650	0.818	0.008	0.577	0.832	0.87 (0.40 - 1.87)	0.715	0.840
	MRI_LS	26	2.56 (1.00 - 6.56)	0.051	0.118	28.4	0.291	0.335	0.88 (0.07 - 10.43)	0.922	0.974	0.019	0.370	0.832	2.11 (0.58 - 7.75)	0.258	0.477
**Trait: Physical Activity**																	
MVPA	LS	13	0.54 (0.34 - 0.86)	0.009	0.027	10.0	0.613	0.651	0.99 (0.08 - 12.30)	0.995	0.995	-0.015	0.637	0.859	0.71 (0.37 - 1.39)	0.321	0.546
	MRI_LS	11	0.78 (0.33 - 1.84)	0.567	0.719	11.0	0.361	0.409	9.78 (0.14 - 690.51)	0.321	0.568	-0.064	0.264	0.703	1.38 (0.43 - 4.47)	0.589	0.727

Results are presented using GWAS data from LS in GIGASTROKE, and in a sensitivity analysis in a cohort of MRI-confirmed LS.
Abbreviations: SNPs, single nucleotide polymorphisms; OR, odds ratio; P, p-value; q, q-value (p-values after adjustment for multiple testing by false discovery rate (FDR)); Q, Cochran's Q statistics exploring heterogeneity across variants; Int, Egger's intercept estimate exploring pleiotropic effect; DBP, diastolic blood pressure; PP, pulse pressure; SBP, systolic blood pressure; HDL, high density lipoprotein; LDL, low density lipoprotein; TC, total cholesterol; TG, triglycerides; T2D, type 2 diabetes; HbA1c, haemoglobin A1C; TG:HDL, triglycerides to high density lipoprotein ratio; BMI, body mass index; WHR, waist to hip ratio; Lifetime smk, life time smoking index; SmkInit, smoking initiation; DrnkWk, drinks per week; and MVPA, moderate-to-vigorous intensity physical activity during leisure time.

Supplementary Table 8 Sensitivity analyses for the Mendelian randomization associations of genetically predicted cardiovascular risk factors with imaging markers of cSVD

Exposure	Outcome	SNPs	Inverse Variance Weighted						MR Egger						Weighted Median		
			Beta (95% CI)	P	q	Q statistic			Beta (95% CI)	P	q	Egger's Int			Beta (95% CI)	P	q
						Q	P	q				Int	P	q			
**Trait: Blood Pressure**																	
	WMH	701	0.02 (0.02 - 0.02)	<0.001	<0.001	1733.3	<0.001	<0.001	0.02 (0.01 - 0.04)	<0.001	0.002	-0.001	0.496	0.832	0.02 (0.02 - 0.03)	<0.001	<0.001
DBP	Lower FA	699	0.07 (0.05 - 0.09)	<0.001	<0.001	1419.7	<0.001	<0.001	0.10 (0.05 - 0.15)	<0.001	0.002	-0.005	0.232	0.681	0.07 (0.05 - 0.10)	<0.001	<0.001
	MD	699	0.09 (0.07 - 0.11)	<0.001	<0.001	1417.5	<0.001	<0.001	0.12 (0.07 - 0.18)	<0.001	<0.001	-0.006	0.203	0.665	0.10 (0.07 - 0.12)	<0.001	<0.001
	WMH	560	0.00 (-0.00 - 0.01)	0.095	0.198	1384.8	<0.001	<0.001	0.00 (-0.01 - 0.02)	0.533	0.719	0.000	0.981	0.981	0.01 (-0.00 - 0.01)	0.054	0.159
PP	Lower FA	557	0.02 (-0.00 - 0.04)	0.051	0.118	1143.3	<0.001	<0.001	0.01 (-0.04 - 0.06)	0.655	0.818	0.002	0.740	0.907	0.02 (-0.00 - 0.04)	0.085	0.198
	MD	557	0.03 (0.01 - 0.05)	0.001	0.005	1162.2	<0.001	<0.001	0.04 (-0.01 - 0.10)	0.105	0.288	-0.002	0.676	0.897	0.05 (0.02 - 0.07)	<0.001	0.001
	WMH	756	0.01 (0.01 - 0.01)	<0.001	<0.001	1756.5	<0.001	<0.001	0.01 (0.01 - 0.02)	<0.001	0.002	-0.001	0.179	0.665	0.01 (0.01 - 0.01)	<0.001	<0.001
SBP	Lower FA	752	0.04 (0.03 - 0.05)	<0.001	<0.001	1404.7	<0.001	<0.001	0.05 (0.02 - 0.08)	0.001	0.008	-0.003	0.502	0.832	0.05 (0.03 - 0.06)	<0.001	<0.001
	MD	752	0.06 (0.04 - 0.07)	<0.001	<0.001	1317.8	<0.001	<0.001	0.07 (0.04 - 0.10)	<0.001	<0.001	-0.005	0.207	0.665	0.07 (0.06 - 0.09)	<0.001	<0.001
**Trait: Lipid Metabolism**																	
	WMH	949	-0.02 (-0.05 - 0.01)	0.216	0.383	1588.2	<0.001	<0.001	-0.04 (-0.09 - 0.00)	0.055	0.187	0.001	0.140	0.665	-0.03 (-0.09 - 0.02)	0.202	0.389
HDL	Lower FA	883	-0.13 (-0.28 - 0.02)	0.081	0.177	1609.4	<0.001	<0.001	-0.25 (-0.46 - -0.03)	0.028	0.126	0.003	0.169	0.665	0.07 (-0.14 - 0.27)	0.515	0.694
	MD	883	-0.02 (-0.16 - 0.12)	0.783	0.926	1461.3	<0.001	<0.001	-0.15 (-0.36 - 0.06)	0.151	0.368	0.004	0.086	0.665	0.04 (-0.22 - 0.30)	0.775	0.866
	WMH	699	0.03 (-0.00 - 0.07)	0.063	0.140	1389.2	<0.001	<0.001	0.04 (-0.01 - 0.09)	0.091	0.257	0.000	0.577	0.832	0.05 (0.00 - 0.10)	0.039	0.126
LDL	Lower FA	655	0.14 (-0.03 - 0.30)	0.098	0.199	1280.7	<0.001	<0.001	0.25 (0.02 - 0.49)	0.035	0.140	-0.004	0.180	0.665	0.24 (0.02 - 0.46)	0.034	0.117
	MD	655	0.14 (-0.02 - 0.31)	0.084	0.179	1280.7	<0.001	<0.001	0.32 (0.09 - 0.56)	0.007	0.042	-0.006	0.040	0.563	0.26 (0.04 - 0.48)	0.019	0.079
	WMH	861	0.02 (-0.01 - 0.05)	0.256	0.426	1633.4	<0.001	<0.001	0.03 (-0.02 - 0.08)	0.214	0.444	0.000	0.514	0.832	0.04 (-0.00 - 0.08)	0.079	0.191
TC	Lower FA	802	0.02 (-0.14 - 0.19)	0.786	0.926	1621.0	<0.001	<0.001	0.03 (-0.22 - 0.28)	0.807	0.927	0.000	0.932	0.966	-0.04 (-0.26 - 0.18)	0.736	0.846
	MD	802	0.09 (-0.07 - 0.25)	0.279	0.448	1535.5	<0.001	<0.001	0.20 (-0.05 - 0.44)	0.112	0.296	-0.003	0.243	0.684	0.19 (-0.03 - 0.41)	0.089	0.199
	WMH	810	0.02 (-0.02 - 0.06)	0.309	0.485	1451.9	<0.001	<0.001	0.01 (-0.05 - 0.07)	0.696	0.857	0.000	0.738	0.907	-0.03 (-0.08 - 0.02)	0.289	0.511
TG	Lower FA	771	0.02 (-0.16 - 0.20)	0.823	0.926	1511.5	<0.001	<0.001	0.28 (-0.01 - 0.56)	0.059	0.191	-0.007	0.023	0.563	-0.07 (-0.32 - 0.18)	0.583	0.727
	MD	771	-0.01 (-0.18 - 0.17)	0.947	0.994	1346.2	<0.001	<0.001	0.25 (-0.02 - 0.53)	0.067	0.202	-0.007	0.015	0.563	-0.02 (-0.27 - 0.23)	0.890	0.911
**Trait: Glucose Metabolism**																	
	WMH	96	-0.00 (-0.14 - 0.14)	0.996	0.996	215.8	<0.001	<0.001	-0.01 (-0.27 - 0.26)	0.970	0.988	0.000	0.966	0.978	0.01 (-0.17 - 0.20)	0.890	0.911
HbA1c	Lower FA	93	-0.07 (-0.66 - 0.53)	0.828	0.926	163.4	<0.001	<0.001	-0.11 (-1.20 - 0.98)	0.840	0.952	0.001	0.920	0.966	-0.00 (-0.74 - 0.74)	1.000	1.000
	MD	93	-0.30 (-0.92 - 0.31)	0.332	0.486	172.9	<0.001	<0.001	-0.35 (-1.48 - 0.78)	0.549	0.728	0.001	0.928	0.966	-0.53 (-1.29 - 0.24)	0.177	0.367
	WMH	458	0.01 (-0.00 - 0.03)	0.128	0.251	803.8	<0.001	<0.001	-0.01 (-0.05 - 0.03)	0.573	0.749	0.001	0.159	0.665	-0.00 (-0.03 - 0.02)	0.880	0.911
T2D	Lower FA	450	0.05 (-0.03 - 0.13)	0.223	0.386	672.8	<0.001	<0.001	0.16 (-0.02 - 0.34)	0.078	0.229	-0.006	0.174	0.665	0.16 (0.04 - 0.29)	0.010	0.046
	MD	450	-0.04 (-0.12 - 0.04)	0.339	0.488	629.2	<0.001	<0.001	0.14 (-0.04 - 0.31)	0.128	0.329	-0.009	0.029	0.563	0.04 (-0.07 - 0.16)	0.460	0.663
	WMH	324	0.01 (-0.02 - 0.03)	0.593	0.741	552.7	<0.001	<0.001	-0.01 (-0.05 - 0.04)	0.805	0.927	0.001	0.457	0.832	-0.01 (-0.04 - 0.03)	0.756	0.857
TG:HDL	Lower FA	314	0.09 (-0.05 - 0.22)	0.207	0.375	609.1	<0.001	<0.001	0.15 (-0.07 - 0.36)	0.177	0.386	-0.003	0.466	0.832	0.07 (-0.10 - 0.25)	0.415	0.653
	MD	314	0.06 (-0.07 - 0.18)	0.389	0.542	542.7	<0.001	<0.001	0.13 (-0.07 - 0.34)	0.198	0.421	-0.004	0.336	0.832	0.02 (-0.16 - 0.20)	0.826	0.890
**Trait: Adiposity Markers**																	
	WMH	1 266	0.06 (0.02 - 0.10)	0.002	0.006	2093.3	<0.001	<0.001	0.02 (-0.09 - 0.13)	0.721	0.872	0.001	0.439	0.832	0.06 (0.01 - 0.11)	0.019	0.079
BMI	Lower FA	1 261	-0.24 (-0.41 - -0.06)	0.007	0.024	2033.6	<0.001	<0.001	-0.16 (-0.66 - 0.34)	0.533	0.719	-0.001	0.747	0.907	-0.07 (-0.30 - 0.17)	0.564	0.727
	MD	1 261	-0.41 (-0.57 - -0.25)	<0.001	<0.001	1761.5	<0.001	<0.001	-0.54 (-1.00 - -0.07)	0.024	0.116	0.002	0.576	0.832	-0.39 (-0.61 - -0.17)	0.001	0.004
	WMH	500	0.02 (-0.03 - 0.07)	0.373	0.528	1033.4	<0.001	<0.001	0.10 (-0.03 - 0.23)	0.138	0.346	-0.001	0.219	0.665	0.06 (-0.01 - 0.13)	0.078	0.191
WHR	Lower FA	500	0.09 (-0.15 - 0.34)	0.453	0.614	1080.0	<0.001	<0.001	0.11 (-0.50 - 0.72)	0.728	0.872	0.000	0.961	0.978	0.12 (-0.18 - 0.42)	0.423	0.654
	MD	500	0.18 (-0.07 - 0.42)	0.152	0.288	1043.0	<0.001	<0.001	0.29 (-0.32 - 0.89)	0.355	0.591	-0.002	0.703	0.905	0.17 (-0.13 - 0.46)	0.264	0.477
**Trait: Smoking Status**																	
	WMH	29	0.03 (-0.05 - 0.12)	0.455	0.614	27.4	0.495	0.546	-0.01 (-0.16 - 0.14)	0.900	0.974	0.002	0.509	0.832	-0.05 (-0.17 - 0.07)	0.451	0.663
CigDay	Lower FA	28	0.01 (-0.73 - 0.74)	0.982	0.994	97.5	<0.001	<0.001	0.11 (-1.21 - 1.43)	0.872	0.962	-0.004	0.856	0.958	-0.31 (-0.90 - 0.28)	0.307	0.532
	MD	28	-0.13 (-0.77 - 0.50)	0.680	0.826	71.4	<0.001	<0.001	0.48 (-0.62 - 1.59)	0.401	0.619	-0.025	0.198	0.665	-0.26 (-0.84 - 0.33)	0.390	0.625
	WMH	137	0.14 (0.03 - 0.24)	0.008	0.026	230.2	<0.001	<0.001	0.28 (-0.10 - 0.66)	0.156	0.369	-0.002	0.451	0.832	0.08 (-0.04 - 0.20)	0.198	0.389
Lifetime smk	Lower FA	137	0.54 (0.04 - 1.04)	0.036	0.092	258.4	<0.001	<0.001	1.83 (-0.01 - 3.67)	0.053	0.187	-0.020	0.155	0.665	0.76 (0.19 - 1.32)	0.008	0.045
	MD	137	0.37 (-0.11 - 0.84)	0.130	0.251	229.6	<0.001	<0.001	2.55 (0.83 - 4.26)	0.004	0.030	-0.034	0.011	0.563	0.50 (-0.07 - 1.08)	0.086	0.198
	WMH	109	0.05 (-0.09 - 0.19)	0.491	0.642	157.9	0.001	0.002	0.31 (-0.31 - 0.94)	0.328	0.568	-0.003	0.395	0.832	0.04 (-0.14 - 0.21)	0.686	0.821
SmkInit	Lower FA	109	0.08 (-0.66 - 0.83)	0.827	0.926	209.6	<0.001	<0.001	1.44 (-1.97 - 4.86)	0.410	0.619	-0.016	0.426	0.832	-0.38 (-1.22 - 0.46)	0.380	0.622
	MD	109	-0.05 (-0.77 - 0.67)	0.894	0.961	192.6	<0.001	<0.001	1.10 (-2.19 - 4.40)	0.514	0.716	-0.013	0.485	0.832	-0.03 (-0.89 - 0.83)	0.946	0.957
**Trait: Drinking Status**																	
	WMH	43	-0.06 (-0.19 - 0.06)	0.325	0.485	62.1	0.023	0.031	-0.01 (-0.20 - 0.18)	0.928	0.974	-0.002	0.462	0.832	0.06 (-0.09 - 0.21)	0.435	0.660
DrnkWk	Lower FA	41	0.55 (-0.37 - 1.47)	0.241	0.410	125.9	<0.001	<0.001	0.66 (-0.83 - 2.16)	0.391	0.615	-0.003	0.852	0.958	1.05 (0.25 - 1.86)	0.010	0.046
	MD	41	0.02 (-0.89 - 0.92)	0.968	0.994	121.1	<0.001	<0.001	-0.13 (-1.60 - 1.35)	0.867	0.962	0.004	0.806	0.946	0.40 (-0.41 - 1.22)	0.331	0.552
**Trait: Physical Activity**																	
	WMH	16	-0.20 (-0.39 - -0.02)	0.032	0.086	32.4	0.006	0.008	-0.25 (-1.22 - 0.72)	0.617	0.795	0.001	0.922	0.966	-0.23 (-0.43 - -0.03)	0.021	0.082
MVPA	Lower FA	16	0.04 (-0.56 - 0.63)	0.904	0.961	11.5	0.717	0.752	-1.35 (-4.25 - 1.55)	0.377	0.605	0.036	0.355	0.832	-0.14 (-0.91 - 0.63)	0.722	0.840
	MD	16	-0.31 (-0.91 - 0.30)	0.318	0.485	8.4	0.906	0.917	-2.18 (-5.10 - 0.74)	0.165	0.379	0.049	0.219	0.665	-0.53 (-1.32 - 0.26)	0.187	0.378

Results are presented using GWAS data from WMH, white matter hyperintensity; lower FA, fractional anisotropy; and MD, mean diffusivity. Abbreviations: SNPs, single nucleotide polymorphisms; OR, odds ratio; P, p-value; q, q-value (p-values after adjustment for multiple testing by false discovery rate (FDR)); Q, Cochran's Q statistics exploring heterogeneity across variants; Int, Egger's intercept estimate exploring pleiotropic effect; DBP, diastolic blood pressure; PP, pulse pressure; SBP, systolic blood pressure; HDL, high density lipoprotein; LDL, low density lipoprotein; TC, total cholesterol; TG, triglycerides; T2D, type 2 diabetes; HbA1c, haemoglobin A1C; TG:HDL, triglycerides to high density lipoprotein ratio; BMI, body mass index; WHR, waist to hip ratio; Lifetime smk, life time smoking index; SmkInit, smoking initiation; DrnkWk, drinks per week; and MVPA, moderate-to-vigorous intensity physical activity during leisure time.

Supplementary Table 9 Sensitivity analyses for the Mendelian randomization associations of genetically proxied antihypertensive, antihyperlipidemic, antihyperglycemic, and antiobesity therapies with lacunar stroke

Drug Target	Outcome	SNPs	Inverse Variance Weighted						MR Egger						Weighted Median		
			OR (95% CI)	P	q	Q statistic			OR (95% CI)	P	q	Egger's Int			OR (95% CI)	P	q
						Q	P	q				Int	P	q			
**Drug: Antihypertensives**																	
ACEs	LS	1	0.93 (0.80 - 1.08)	0.348	0.531	-	-	-	-	-	-	-	-	-	-	-	-
BBs	LS	5	0.98 (0.82 - 1.18)	0.865	0.865	19.4	0.001	0.045	0.89 (0.46 - 1.74)	0.763	0.907	0.033	0.784	1.000	1.01 (0.91 - 1.13)	0.818	0.933
	MRI_LS	2	1.06 (0.62 - 1.82)	0.826	0.838	4.9	0.027	0.363	-	-	-	-	-	-	-	-	-
CCBs	LS	19	0.97 (0.93 - 1.02)	0.228	0.418	27.2	0.075	0.460	0.97 (0.86 - 1.09)	0.619	0.891	0.002	0.926	1.000	0.96 (0.92 - 1.01)	0.161	0.396
	MRI_LS	14	0.97 (0.90 - 1.04)	0.408	0.599	11.8	0.543	0.839	1.06 (0.83 - 1.36)	0.660	0.905	-0.028	0.485	1.000	0.98 (0.88 - 1.08)	0.677	0.850
**Drug: Antihyperlipidemic**																	
APOC3	LS	19	1.15 (0.94 - 1.41)	0.184	0.379	13.3	0.771	0.931	0.99 (0.66 - 1.49)	0.967	0.984	0.013	0.422	1.000	1.11 (0.82 - 1.50)	0.492	0.675
	MRI_LS	25	0.96 (0.69 - 1.34)	0.825	0.838	23.4	0.499	0.839	1.10 (0.59 - 2.07)	0.764	0.907	-0.012	0.628	1.000	0.98 (0.63 - 1.51)	0.917	0.933
CETP	LS	49	0.84 (0.76 - 0.93)	0.001	0.015	47.2	0.504	0.839	0.82 (0.70 - 0.96)	0.016	0.162	0.003	0.660	1.000	0.83 (0.72 - 0.96)	0.013	0.107
	MRI_LS	49	0.75 (0.62 - 0.91)	0.003	0.035	43.1	0.673	0.889	0.66 (0.49 - 0.89)	0.009	0.126	0.013	0.276	1.000	0.75 (0.57 - 1.00)	0.050	0.269
HMGCR	LS	9	0.69 (0.47 - 1.02)	0.066	0.203	6.4	0.600	0.855	0.30 (0.04 - 2.06)	0.258	0.810	0.045	0.411	1.000	0.67 (0.40 - 1.11)	0.121	0.363
	MRI_LS	7	0.56 (0.29 - 1.11)	0.097	0.288	4.9	0.560	0.847	0.81 (0.03 - 24.76)	0.907	0.984	-0.019	0.842	1.000	0.58 (0.25 - 1.33)	0.197	0.433
LDLR	LS	25	0.92 (0.69 - 1.21)	0.549	0.643	42.9	0.010	0.229	0.99 (0.58 - 1.70)	0.985	0.985	-0.005	0.731	1.000	1.02 (0.76 - 1.36)	0.903	0.933
	MRI_LS	9	1.20 (0.73 - 1.97)	0.469	0.632	10.6	0.225	0.806	0.59 (0.24 - 1.47)	0.293	0.810	0.058	0.124	1.000	1.07 (0.56 - 2.03)	0.845	0.933
LPL	LS	24	0.74 (0.58 - 0.95)	0.019	0.097	30.3	0.141	0.639	0.77 (0.51 - 1.19)	0.256	0.810	-0.003	0.824	1.000	0.98 (0.72 - 1.33)	0.886	0.933
	MRI_LS	28	0.54 (0.39 - 0.74)	<0.001	0.006	23.8	0.640	0.888	0.48 (0.28 - 0.82)	0.013	0.149	0.009	0.597	1.000	0.68 (0.42 - 1.11)	0.123	0.363
NPC1L1	LS	5	0.34 (0.13 - 0.92)	0.034	0.149	6.6	0.157	0.667	0.04 (0.00 - 10.84)	0.339	0.810	0.076	0.495	1.000	0.39 (0.16 - 0.95)	0.038	0.251
	MRI_LS	7	0.25 (0.04 - 1.41)	0.116	0.311	11.6	0.071	0.460	23.93 (0.00 - 356912.30)	0.546	0.891	-0.157	0.388	1.000	0.50 (0.09 - 2.74)	0.423	0.623
PCSK9	LS	21	1.12 (0.80 - 1.57)	0.513	0.634	16.7	0.673	0.889	0.89 (0.49 - 1.60)	0.691	0.907	0.010	0.353	1.000	1.35 (0.83 - 2.21)	0.230	0.447
	MRI_LS	17	0.82 (0.47 - 1.44)	0.494	0.632	15.0	0.525	0.839	0.42 (0.10 - 1.70)	0.241	0.810	0.033	0.317	1.000	0.60 (0.27 - 1.31)	0.200	0.433
**Drug: Antihyperglycemic**																	
ABCC8	LS	3	0.22 (0.10 - 0.49)	<0.001	0.006	0.5	0.780	0.931	0.23 (0.01 - 8.62)	0.574	0.891	-0.003	0.975	1.000	0.22 (0.07 - 0.67)	0.008	0.074
	MRI_LS	4	0.59 (0.15 - 2.25)	0.436	0.605	8.8	0.032	0.363	0.71 (0.00 - 698.53)	0.931	0.984	-0.011	0.960	1.000	0.67 (0.27 - 1.67)	0.391	0.592
GLP1R	LS	1	2.44 (0.57 - 10.34)	0.227	0.531	-	-	-	-	-	-	-	-	-	-	-	-
	MRI_LS	1	4.74 (0.58 - 39.06)	0.148	0.531	-	-	-	-	-	-	-	-	-	-	-	-
PPARG	LS	8	0.78 (0.55 - 1.11)	0.172	0.376	1.0	0.994	0.994	0.65 (0.29 - 1.48)	0.347	0.810	0.011	0.644	1.000	0.78 (0.51 - 1.20)	0.264	0.458
	MRI_LS	8	0.89 (0.49 - 1.61)	0.702	0.760	7.1	0.418	0.839	1.32 (0.35 - 5.04)	0.698	0.907	-0.025	0.541	1.000	0.92 (0.43 - 1.96)	0.822	0.933
**Drug: Antiobesity**																	
GIPR	LS	5	0.10 (0.02 - 0.51)	0.006	0.042	5.4	0.251	0.814	0.11 (0.00 - 3.56)	0.300	0.810	-0.001	0.970	1.000	0.19 (0.03 - 1.19)	0.076	0.321
	MRI_LS	5	0.15 (0.02 - 1.03)	0.054	0.183	2.7	0.604	0.855	0.21 (0.01 - 5.67)	0.420	0.845	-0.009	0.826	1.000	0.09 (0.01 - 0.98)	0.048	0.269
GLP1R	LS	2	0.56 (0.11 - 2.86)	0.482	0.632	1.1	0.293	0.839	-	-	-	-	-	-	-	-	-
	MRI_LS	2	0.15 (0.01 - 1.93)	0.144	0.327	0.0	0.866	0.945	-	-	-	-	-	-	-	-	-

Results are presented using GWAS data from LS in GIGASTROKE, and in a sensitivity analysis in a cohort of MRI-confirmed LS.
Abbreviations*:* SNPs, single nucleotide polymorphisms; OR, odds ratio; P, p-value; q, q-value (p-values after adjustment for multiple testing by false discovery rate (FDR)); Q, Cochran's Q statistics exploring heterogeneity across variants; Int, Egger's intercept estimate exploring pleiotropic effect; ACEs, angiotensin-converting enzyme inhibitors; BBs, beta blockers; CCBs, calcium channel blockers; APOC3, Apolipoprotein C-III inhibitors; CETP, Cholesteryl Ester Transfer Protein inhibitors; HMGCR, HMG-CoA reductase inhibitors; *LDLR*, LDL Receptor; *LPL*, lipoprotein lipase; NPC1L1, NPC1 Like Intracellular Cholesterol Transporter 1 inhibitors; PCSK9, proprotein convertase subtilisin/kexin type 9 inhibitors; ABCC8, ATP binding cassette subfamily C member 8; GLP1R, glucagon like peptide 1 receptor; PPARG, peroxisome proliferator activated receptor gamma; GIPR, gastric inhibitory polypeptide receptor.

Supplementary Table 10 Sensitivity analyses for the Mendelian randomization associations of genetically proxied antihypertensive, antihyperlipidemic, antihyperglycemic, and antiobesity therapies with imaging markers of cSVD

			Inverse Variance Weighted						MR Egger						Weighted Median		
Drug Target	Outcome	SNPs	Beta (95% CI)	P	q	Q statistic			Beta (95% CI)	P	q	Egger's Int			Beta (95% CI)	P	q
						Q	P	q				Int	P	q			
**Drug: Antihypertensives**																	
	WMH	1	-0.01 (-0.06 - 0.03)	0.531	0.531	-	-	-	-	-	-	-	-	-	-	-	-
ACEs	MD	1	0.10 (-0.11 - 0.30)	0.358	0.531	-	-	-	-	-	-	-	-	-	-	-	-
	Lower FA	1	0.08 (-0.13 - 0.28)	0.468	0.531	-	-	-	-	-	-	-	-	-	-	-	-
	WMH	6	-0.02 (-0.05 - 0.01)	0.116	0.311	11.3	0.046	0.443	-0.09 (-0.16 - -0.01)	0.100	0.701	0.024	0.174	1.000	-0.03 (-0.06 - -0.01)	0.004	0.047
BBs	Lower FA	6	-0.05 (-0.13 - 0.04)	0.295	0.504	4.4	0.494	0.839	-0.07 (-0.37 - 0.22)	0.650	0.905	0.011	0.857	1.000	-0.06 (-0.16 - 0.04)	0.242	0.447
	MD	6	-0.10 (-0.19 - -0.01)	0.029	0.140	5.5	0.363	0.839	-0.22 (-0.53 - 0.09)	0.238	0.810	0.045	0.477	1.000	-0.12 (-0.23 - -0.01)	0.031	0.226
	WMH	24	-0.02 (-0.03 - -0.00)	0.007	0.047	40.2	0.015	0.247	-0.06 (-0.08 - -0.03)	0.001	0.053	0.014	0.009	0.615	-0.02 (-0.04 - -0.01)	0.001	0.027
CCBs	Lower FA	24	-0.10 (-0.15 - -0.04)	<0.001	0.008	32.6	0.088	0.460	-0.20 (-0.33 - -0.06)	0.008	0.126	0.036	0.120	1.000	-0.12 (-0.19 - -0.05)	0.001	0.015
	MD	24	-0.13 (-0.19 - -0.06)	<0.001	0.006	48.0	0.002	0.057	-0.25 (-0.41 - -0.09)	0.006	0.126	0.044	0.120	1.000	-0.18 (-0.25 - -0.11)	<0.001	<0.001
**Drug: Antihyperlipidemic**																	
	WMH	40	0.01 (-0.04 - 0.07)	0.597	0.688	42.5	0.321	0.839	0.04 (-0.05 - 0.14)	0.368	0.810	-0.003	0.461	1.000	0.04 (-0.03 - 0.12)	0.258	0.458
APOC3	Lower FA	32	-0.32 (-0.56 - -0.07)	0.012	0.070	26.8	0.680	0.889	-0.12 (-0.56 - 0.32)	0.591	0.891	-0.020	0.293	1.000	-0.33 (-0.67 - 0.01)	0.055	0.269
	MD	32	-0.37 (-0.62 - -0.12)	0.004	0.035	22.8	0.857	0.945	-0.37 (-0.81 - 0.07)	0.107	0.701	0.000	0.985	1.000	-0.28 (-0.65 - 0.08)	0.130	0.364
	WMH	100	-0.04 (-0.07 - -0.01)	0.012	0.070	110.4	0.205	0.792	-0.02 (-0.07 - 0.02)	0.343	0.810	-0.002	0.351	1.000	-0.04 (-0.08 - 0.01)	0.092	0.339
CETP	Lower FA	74	0.07 (-0.08 - 0.22)	0.361	0.572	90.2	0.084	0.460	0.06 (-0.17 - 0.29)	0.601	0.891	0.001	0.917	1.000	0.18 (-0.04 - 0.40)	0.114	0.363
	MD	74	0.09 (-0.05 - 0.23)	0.205	0.410	58.5	0.891	0.946	-0.01 (-0.21 - 0.20)	0.953	0.984	0.010	0.233	1.000	0.03 (-0.17 - 0.24)	0.754	0.927
	WMH	31	-0.02 (-0.13 - 0.09)	0.716	0.760	29.0	0.518	0.839	-0.01 (-0.51 - 0.48)	0.959	0.984	0.000	0.977	1.000	-0.05 (-0.19 - 0.10)	0.551	0.722
HMGCR	Lower FA	22	0.21 (-0.30 - 0.73)	0.414	0.599	9.0	0.989	0.994	0.40 (-1.94 - 2.74)	0.739	0.907	-0.009	0.873	1.000	0.09 (-0.58 - 0.76)	0.789	0.933
	MD	22	-0.33 (-0.85 - 0.19)	0.211	0.410	10.7	0.968	0.994	0.33 (-2.02 - 2.69)	0.784	0.907	-0.033	0.578	1.000	-0.50 (-1.17 - 0.17)	0.145	0.372
	WMH	57	0.04 (-0.01 - 0.10)	0.141	0.327	56.3	0.464	0.839	-0.03 (-0.13 - 0.07)	0.504	0.891	0.005	0.084	1.000	-0.05 (-0.13 - 0.03)	0.206	0.433
LDLR	Lower FA	46	0.08 (-0.18 - 0.34)	0.532	0.635	45.9	0.435	0.839	-0.04 (-0.52 - 0.43)	0.854	0.951	0.009	0.535	1.000	0.04 (-0.34 - 0.42)	0.826	0.933
	MD	46	0.05 (-0.21 - 0.30)	0.713	0.760	43.7	0.529	0.839	-0.15 (-0.61 - 0.32)	0.547	0.891	0.013	0.339	1.000	-0.19 (-0.57 - 0.18)	0.316	0.504
	WMH	54	0.05 (0.00 - 0.10)	0.035	0.149	55.1	0.394	0.839	0.06 (-0.02 - 0.14)	0.158	0.810	-0.001	0.817	1.000	0.06 (-0.00 - 0.13)	0.070	0.316
LPL	Lower FA	45	-0.14 (-0.37 - 0.09)	0.222	0.418	34.0	0.861	0.945	-0.28 (-0.66 - 0.11)	0.164	0.810	0.012	0.395	1.000	-0.13 (-0.47 - 0.20)	0.440	0.633
	MD	45	0.06 (-0.17 - 0.29)	0.620	0.703	34.1	0.859	0.945	-0.17 (-0.56 - 0.21)	0.384	0.810	0.020	0.150	1.000	0.00 (-0.32 - 0.33)	0.991	0.991
	WMH	10	-0.15 (-0.37 - 0.07)	0.183	0.379	5.6	0.781	0.931	-0.39 (-1.50 - 0.72)	0.510	0.891	0.008	0.676	1.000	-0.15 (-0.43 - 0.13)	0.302	0.495
NPC1L1	Lower FA	7	0.62 (-0.54 - 1.77)	0.296	0.504	7.6	0.269	0.830	4.32 (-1.59 - 10.22)	0.212	0.810	-0.127	0.267	1.000	0.88 (-0.53 - 2.28)	0.222	0.447
	MD	7	1.10 (0.06 - 2.13)	0.038	0.150	1.8	0.938	0.981	2.70 (-2.82 - 8.21)	0.382	0.810	-0.055	0.587	1.000	0.97 (-0.23 - 2.17)	0.112	0.363
	WMH	46	0.03 (-0.07 - 0.13)	0.524	0.635	61.4	0.052	0.445	0.11 (-0.06 - 0.28)	0.212	0.810	-0.004	0.278	1.000	0.01 (-0.11 - 0.13)	0.891	0.933
PCSK9	Lower FA	36	0.18 (-0.24 - 0.61)	0.402	0.599	36.0	0.422	0.839	0.11 (-0.67 - 0.90)	0.777	0.907	0.003	0.844	1.000	0.22 (-0.41 - 0.85)	0.490	0.675
	MD	36	0.33 (-0.09 - 0.75)	0.119	0.311	25.6	0.876	0.945	0.20 (-0.57 - 0.97)	0.608	0.891	0.006	0.694	1.000	0.34 (-0.29 - 0.96)	0.295	0.495
**Drug: Antihyperglycemic**																	
	WMH	4	-0.06 (-0.20 - 0.08)	0.428	0.605	2.4	0.502	0.839	-0.39 (-0.97 - 0.19)	0.321	0.810	0.019	0.369	1.000	-0.07 (-0.23 - 0.09)	0.374	0.581
ABCC8	Lower FA	4	-0.66 (-1.35 - 0.02)	0.058	0.188	3.1	0.371	0.839	-1.59 (-4.76 - 1.59)	0.430	0.845	0.054	0.615	1.000	-0.58 (-1.35 - 0.19)	0.138	0.371
	MD	4	-0.67 (-1.35 - 0.00)	0.051	0.183	1.9	0.601	0.855	-0.98 (-3.75 - 1.78)	0.558	0.891	0.018	0.841	1.000	-0.66 (-1.42 - 0.10)	0.087	0.339
	WMH	2	-0.24 (-0.67 - 0.19)	0.277	0.496	2.2	0.138	0.639	-	-	-	-	-	-	-	-	-
GLP1R	Lower FA	2	1.07 (-0.31 - 2.45)	0.127	0.321	0.6	0.425	0.839	-	-	-	-	-	-	-	-	-
	MD	2	0.61 (-0.78 - 1.99)	0.389	0.599	0.1	0.780	0.931	-	-	-	-	-	-	-	-	-
	WMH	9	0.05 (-0.05 - 0.15)	0.362	0.572	7.0	0.536	0.839	0.27 (0.04 - 0.50)	0.054	0.454	-0.014	0.071	1.000	0.03 (-0.10 - 0.17)	0.601	0.771
PPARG	Lower FA	9	-0.23 (-0.71 - 0.25)	0.352	0.572	7.0	0.533	0.839	-0.31 (-1.41 - 0.78)	0.590	0.891	0.005	0.871	1.000	-0.22 (-0.85 - 0.42)	0.505	0.677
	MD	9	-0.48 (-0.97 - 0.00)	0.051	0.183	5.3	0.727	0.931	-0.58 (-1.68 - 0.52)	0.339	0.810	0.006	0.861	1.000	-0.42 (-1.07 - 0.23)	0.205	0.433
**Drug: Antiobesity**																	
	WMH	7	-0.06 (-0.33 - 0.20)	0.640	0.714	2.7	0.842	0.945	-0.06 (-0.55 - 0.43)	0.826	0.937	0.000	0.978	1.000	0.02 (-0.30 - 0.35)	0.893	0.933
GIPR	Lower FA	7	-1.94 (-3.28 - -0.61)	0.004	0.037	6.8	0.344	0.839	-1.93 (-4.57 - 0.71)	0.211	0.810	0.000	0.991	1.000	-2.48 (-4.11 - -0.84)	0.003	0.044
	MD	7	-1.29 (-3.01 - 0.44)	0.144	0.327	11.2	0.082	0.460	-0.62 (-3.95 - 2.72)	0.732	0.907	-0.019	0.657	1.000	-1.05 (-2.79 - 0.70)	0.239	0.447
	WMH	2	0.19 (-0.35 - 0.73)	0.491	0.632	1.3	0.247	0.814	-	-	-	-	-	-	-	-	-
GLP1R	Lower FA	2	-0.96 (-3.74 - 1.83)	0.502	0.632	1.6	0.210	0.792	-	-	-	-	-	-	-	-	-
	MD	2	0.31 (-1.92 - 2.55)	0.784	0.821	0.7	0.405	0.839	-	-	-	-	-	-	-	-	-

Results are presented using GWAS data from WMH, white matter hyperintensity; lower FA, fractional anisotropy; and MD, mean diffusivity. Abbreviations*:* SNPs, single nucleotide polymorphisms; OR, odds ratio; P, p-value; q, q-value (p-values after adjustment for multiple testing by false discovery rate (FDR)); Q, Cochran's Q statistics exploring heterogeneity across variants; Int, Egger's intercept estimate exploring pleiotropic effect; ACEs, angiotensin-converting enzyme inhibitors; BBs, beta blockers; CCBs, calcium channel blockers; APOC3, Apolipoprotein C-III inhibitors; CETP, Cholesteryl Ester Transfer Protein inhibitors; HMGCR, HMG-CoA reductase inhibitors; *LDLR*, LDL Receptor; *LPL*, lipoprotein lipase; NPC1L1, NPC1 Like Intracellular Cholesterol Transporter 1 inhibitors; PCSK9, proprotein convertase subtilisin/kexin type 9 inhibitors; ABCC8, ATP binding cassette subfamily C member 8; GLP1R, glucagon like peptide 1 receptor; PPARG, peroxisome proliferator activated receptor gamma; GIPR, gastric inhibitory polypeptide receptor.

Supplementary Table 11 Results of colocalization analysis

Drug	Target gene	Outcome	No. of SNPs	PP.H0	PP.H1	PP.H2	PP.H3	PP.H4
Antihypertensive	CACNB2	WMH	2170	3.59E-72	1.48E-01	2.01E-71	8.31E-01	2.08E-02
		MD	2170	2.93E-73	1.21E-02	1.93E-72	7.87E-02	9.09E-01
		Lower FA	2170	1.91E-71	7.89E-01	2.84E-72	1.17E-01	9.39E-02
	CACNA1C	WMH	1170	7.78E-17	9.69E-01	1.78E-18	2.22E-02	8.92E-03
		MD	1170	7.82E-17	9.74E-01	1.60E-18	2.00E-02	6.37E-03
		Lower FA	1170	7.81E-17	9.72E-01	1.84E-18	2.29E-02	5.22E-03
	CACNA1D	WMH	2250	1.61E-04	9.28E-01	1.13E-05	6.53E-02	6.37E-03
		MD	2250	1.63E-04	9.36E-01	7.91E-06	4.55E-02	1.83E-02
		Lower FA	2250	1.59E-04	9.17E-01	1.27E-05	7.31E-02	9.29E-03
	ADRB1	MD	126	4.28E-34	9.75E-01	1.37E-36	3.09E-03	2.22E-02
Antihyperlipidemic	LPL	GIGASTROKE	451	0.00E+00	8.77E-01	0.00E+00	1.06E-01	1.67E-02
		MRI-confirmed LS	436	0.00E+00	7.60E-01	0.00E+00	5.43E-02	1.85E-01
		WMH	742	0.00E+00	9.73E-01	0.00E+00	1.71E-02	1.03E-02
	CETP	GIGASTROKE	249	0.00E+00	9.79E-01	0.00E+00	8.98E-03	1.19E-02
		MRI-confirmed LS	217	0.00E+00	8.19E-01	0.00E+00	1.51E-02	1.66E-01
		WMH	275	0.00E+00	9.78E-01	0.00E+00	1.46E-02	7.56E-03
	NPC1L1	GIGASTROKE	234	2.48E-86	9.05E-01	1.94E-87	7.07E-02	2.40E-02
		MD	327	2.71E-86	9.88E-01	1.62E-88	5.92E-03	5.97E-03
	APOC3	MD	390	0.00E+00	9.55E-01	0.00E+00	1.47E-02	3.07E-02
		Lower FA	390	0.00E+00	9.64E-01	0.00E+00	1.71E-02	1.88E-02
Antihyperglycemic	ABCC8	GIGASTROKE	419	2.31E-40	8.81E-01	8.95E-42	3.40E-02	8.51E-02
Antiobesity	GIPR	GIGASTROKE	379	1.66E-76	8.16E-01	1.24E-77	6.10E-02	1.23E-01
		MRI-confirmed LS	336	1.96E-76	9.65E-01	2.79E-78	1.37E-02	2.14E-02
		Lower FA	535	8.78E-77	8.62E-01	7.52E-78	7.38E-02	6.41E-02
